# A computational framework for defining and validating reproducible phenotyping algorithms of 313 diseases in the UK Biobank

**DOI:** 10.1038/s41598-025-05838-9

**Published:** 2025-07-09

**Authors:** Ana Torralbo, Jonathan M. Davitte, Damien C. Croteau-Chonka, Cai Ytsma, Chris Tomlinson, Natalie K. Fitzpatrick, Sheng-Chia Chung, Ghazaleh Fatemifar, Adrian S. Cortes, Tom G. Richardson, Matthew Barclay, Julia Carrasco-Zanini, Chris Finan, Harry Hemingway, Aroon D. Hingorani, Valerie Kuan, Claudia Langenberg, Georgios Lyratzopoulos, R. Thomas Lumbers, Maik Pietzner, Anoop D. Shah, Johan H. Thygesen, Natalie Zelenka, John C. Whittaker, Margaret G. Ehm, Spiros Denaxas

**Affiliations:** 1 Institute of Health Informatics, University College London, London, UK; 2 Department of Human Genetics and Genomics, GlaxoSmithKline, Collegeville, PA USA; 3 Department of Human Genetics and Genomics, GlaxoSmithKline, Cambridge, MA USA; 4 University College London Hospitals NIHR Biomedical Research Centre, University College London, London, UK; 5 Health Data Research, London, UK; 6 Institute of Cardiovascular Science, University College of London, London, UK; 7 Novo Nordisk Research Centre Oxford, Oxford, UK; 8 Department of Human Genetics and Genomics, GlaxoSmithKline, Heidelberg, Germany; 9 Department of Human Genetics and Genomics, GlaxoSmithKline, Stevenage, UK; 10 Epidemiology of Cancer Healthcare & Outcomes (ECHO), Dept. of Behavioural Science and Health, Institute of Epidemiology and Healthcare, University College London, London, UK; 11 Precision Healthcare University Research Institute, Queen Mary University London, London, UK; 12 UCL Hospitals Biomedical Research Center, London, UK; 13 Computational Medicine, Berlin Institute of Health at Charité – Universitätsmedizin Berlin, Berlin, Germany; 14 University College London Hospitals NHS Trust, London, UK; 15 MRC Biostatistics Unit, University of Cambridge, Cambridge, UK; 16 Department of Human Genetics and Genomics, GSK, Collegeville, PA USA; 17 Network.Bio, New York, NY USA; 18 British Heart Foundation Data Science Center, London, UK; 19 NIHR UCL Hospitals BRC, London, UK; 20 National and Kapodistrian University of Athens, NKUA, Athens, Greece

**Keywords:** Diseases, Cancer, Cardiovascular diseases, Endocrine system and metabolic diseases, Eye diseases, Gastrointestinal diseases, Immunological disorders, Rheumatic diseases

## Abstract

Accurate and reproducible phenotyping is essential for large-scale biomedical research. However, developing robust phenotype definitions in biobanks is challenging due to diverse data sources and varying medical ontologies. As a result, the current phenotyping landscape is fragmented. We developed a computational framework to harmonize electronic health record (EHR) data, participant questionnaires, and clinical registry information, defining 313 disease phenotypes among 502,356 UK Biobank (UKB) participants. Our method integrated four medical ontologies (Read v2, CTV3, ICD-10, OPCS-4) across seven data sources, including primary care, hospital admissions, cancer and death registries, and self-reported data on diseases, procedures, and medication. Phenotypes underwent multi-layered validation, assessing data source concordance, age-sex incidence and prevalence patterns, external comparison to a representative UK EHR dataset, modifiable risk factor associations, and genetic correlations with external genome-wide association studies (GWAS). Results indicated consistent disease distributions by age and sex, high correlation with non-selected general population data prevalence estimates, confirmed risk factor associations, and significant genetic correlations with external GWAS for nine of ten evaluated diseases. Our approach establishes comprehensive disease validation profiles, improving phenotype generalizability despite inherent UKB demographic biases. The modular, reproducible framework can be extended to additional diseases and populations, supporting federated analyses across diverse biobanks, and facilitating research in underrepresented populations.

## Introduction

Rapid advances in genotyping and sequencing technology combined with availability of large-scale electronic health record (EHR) data have increased the pace and scale of genomic research studies, leading to a numerous large-scale biobank resources that link phenotypic information collected from participants, including EHR and participant questionnaire data^1^. Examples of such biobanks include the UK Biobank (UKB) (United Kingdom, N ≅ 500,000 participants;^2^), East London Genes and Health (UK, target 100,000;^3^), Our Future Health (UK, target 5 M), Million Veteran Program (USA, N ≅ 1,000,000;^4^), All of Us (USA, N ≅ 723,000 currently;^1^), FinnGen (Finland, N ≅ 500,000;^5^), Estonian Biobank (Estonia, N ≅ 200,000;^6^) and others (International HundredK + Cohorts Consortium (IHCC) (ihccglobal.org)). A core component of such biobanks is data collected during healthcare encounters, which enable the longitudinal follow-up of participants and phenome-wide analyses for research. In the Global Biobank Meta-analysis Initiative^1^, the majority of biobanks make use of EHR as their primary method of phenotypic data collection and follow up of health-related outcomes.

The use of EHR data for research studies has enabled the study of disease in the context of current care paradigms. In the context of genetic research, EHR and different phenotyping methods can have a differential impact on the genetic results^7–9^. In addition, to perform accurate federated analyses across multiple such biobanks, portable and reproducible phenotyping approaches are required^10^.

In terms of disease phenotyping, the current landscape in the UK Biobank and many other biobanks is somewhat fragmented. Disease phenotypes are created in a non-standardized manner, often one disease at a time. Furthermore, case definitions often leverage data captured from a single source, such as participant medical history questionnaire data or administrative data using medical ontology or statistical classification codes (e.g., International Classification of Diseases tenth revision, ICD-10)^11–13^. The process also involves many ad hoc decisions being taken which are often not fully documented. Defining phenotyping algorithms that span multiple data sources is complex and time-consuming due to data fragmentation, the use of different medical ontologies, classification systems, and proprietary data fields. Variability in phenotyping methods hinder research reproducibility and generalizability of results^8^. As a result, it is challenging to implement trait definitions that are consistent and comparable both within and across biobanked resources and researchers have to spend significant resources in integrating data across sources.

Integrating records from different data providers and medical ontologies, as is the case for primary care data in the UKB, is time consuming and complex. Achieving functional integration of multiple data sources, at scale, would enable the development and use of systematic and reproducible disease phenotypes for research. Such phenotypes can enable the identification of cases using multiple data sources, characterization of disease subtypes, boost statistical power for genetic discovery, improve risk stratification, and increase the precision of genetic markers such as polygenic risk scores and prognostic models.

**Fig. 1:**
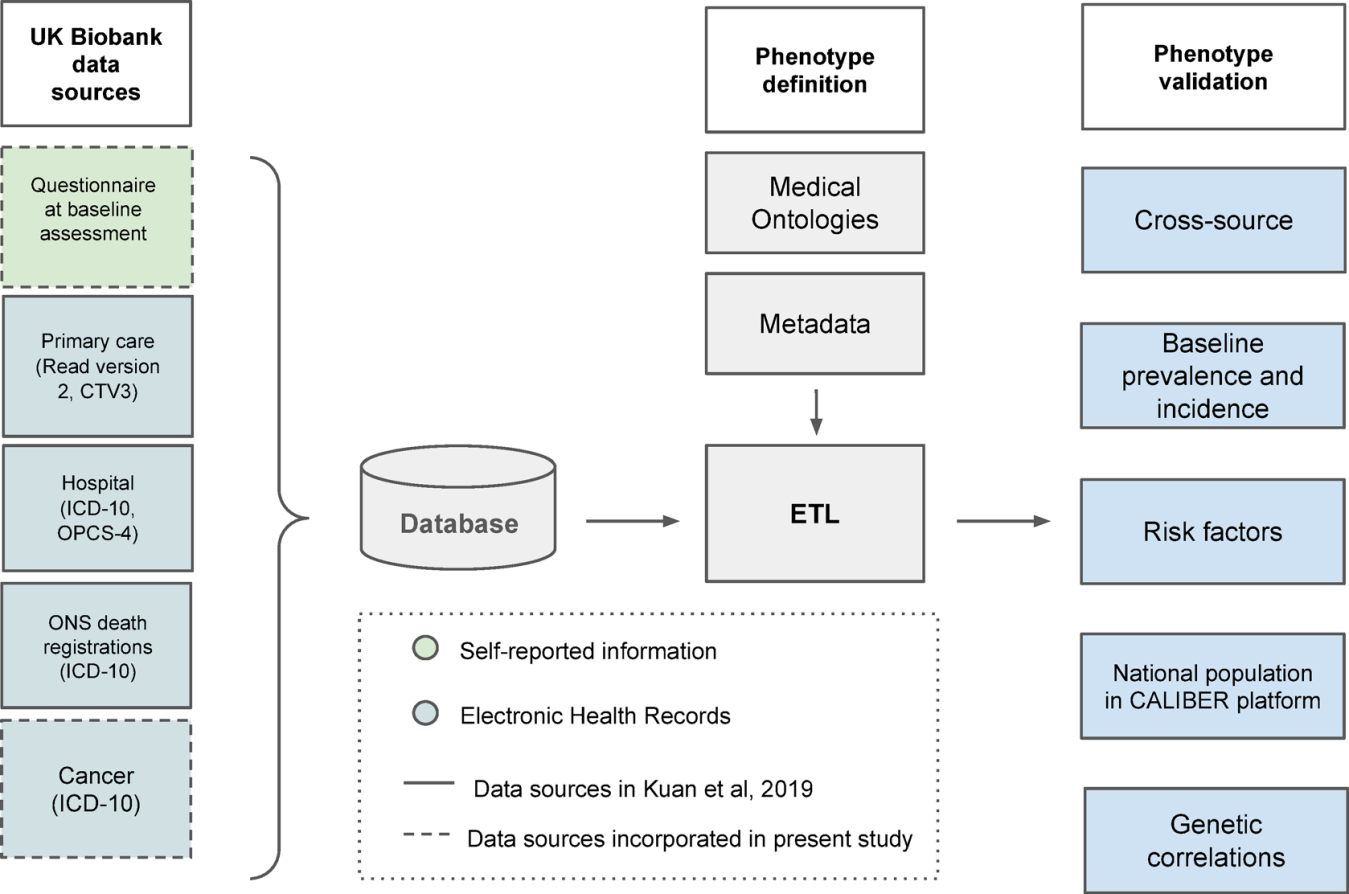
Overview of the phenotyping framework implementation in the UK Biobank, including (**a**) data sources (left), (**b**) Extract, Transform and Load (ETL) processes to obtain records based on the phenotype definition (middle), (**c**) phenotype validation layers.

In this paper, our main objective was to create a computational phenotyping framework and pipeline that integrates diverse EHR and non-EHR data sources to systematically and consistently define and validate disease phenotypes across a broad range of conditions. Our framework combines multiple data sources (e.g., longitudinal EHR, disease registry, and participant questionnaire data) and provides a robust approach for validating phenotype definitions through multiple layers of evidence (Fig. 1). This integrative approach aims to enhance disease characterization, improve accuracy, and facilitate future research in precision medicine and population health by enabling researchers to rapidly create disease phenotypes or test different definition conditions.

## Methods

### Data sources and medical ontologies

#### Baseline cohort

The UKB is a prospective study of ~ 500,000 participants aged 40–69 at entry and recruited in England, Wales and Scotland from the general population (http://www.ukbiobank.ac.uk). At baseline, participants were invited to assessment centres where detailed phenotypic data on their health and lifestyle was collected including lifestyle risk factors, anthropometric measurements, and past medical history. Genetic data, including imputed genotype, exome sequencing, and whole genome sequencing data^2^ is available for participants. All participants provided written informed consent and ethics approval was granted from the North West Multi-Centre Research Ethics Committee (06/MRE08/65). All methods were performed in accordance with the relevant guidelines and regulations. UKB data were obtained following approval of the protocol by the UKB Access Management Committee (https://bbams.ndph.ox.ac.uk/ams/) (Application Numbers 58356 and 20361).

#### EHR data sources

Participants are followed up across linked EHR sources that contain information on:

- primary care EHR (available for half the population by Vision/EMIS data providers for Scotland and Wales, and TPP/Vision for England);
- inpatient hospitalizations (from Hospital Episodes Statistics for England, Patients Episodes Database for Wales and General Acute Inpatient and Day care for Scotland);
- cancer registry data from the national cancer registries, and;
- mortality (provided by NHS Digital for England and Wales and NHS Central Register for Scotland).

Data sources were linked by the UKB using a unique healthcare patient identifier (the NHS number). Participants’ records were processed using an anonymized patient id created by the UKB (‘eid’). Data coverage varied by data providers and country of participant recruitment (Table S1).

#### Medical ontologies

Participant medical history and physiological data collected during the initial UKB assessment centre visit or in subsequent visits are recorded in a custom system. For EHR data, five different ontologies are used: (a) data from inpatient hospitalizations are recorded using the ICD-10 classification system while surgical procedures are recorded using the OPCS-4 classification of codes and procedures; (b) diagnoses drawn from primary care records are recorded using the Read version 2 and Clinical Terms version 3 (CTV3) medical ontologies; (c) cause of death information and cancer registrations were coded using ICD-10 (with some historic records using ICD-9).

#### Self-reported medical history and other questionnaire data

Past medical history collected during UKB baseline visit and validated by clinical research nurses (fields 20001 and 20002) was incorporated in relevant phenotypes as self-reported conditions. Additional fields collected through the baseline questionnaires related to individual phenotypes were also included in phenotype definitions (e.g. field 20544 related to mental health events diagnosed by a professional).

### Study design

The UKB cohort includes 502,356 participants. Participants were included in the current validation analyses when they met the analysis specific criteria (see Figure S1), including: primary care data availability (for the cross source representation) and a single General Practitioner (GP) registration (for the prevalence and incidence calculation), being registered in the UKB during the same period of an external general population comparison cohort (for the comparison with the reference population), or having genetic data availability (for the genetic validation).

The study follow-up period was defined using the individual baseline registration date on which participants attended the UKB assessment centre between 2006 and 2010 to the study end date, defined as the earliest end date across all data sources identified per country (31 March 2016, 31 October 2015, or 28 February 2016 for England, Scotland, or Wales, respectively, or an individual’s death date (see Tables S1 and S2 for data coverage and primary care data EHR software suppliers). 31 March 2016 was considered as the end date in all sources to derive the cross-source concordance and the median age of the phenotypes. Patients in England can only be registered with a single primary care general practitioner, but registration periods can be fragmented (e.g. when an individual moves outside the capture area of a surgery to register with another primary care practice). Participants with a single primary care registration period (such as participants in England from the Vision system) were considered to have a continuous registration period.

### Phenotyping

In this manuscript, we utilized 313 diseases that had been previously selected by clinician experts to reflect the disease burden and healthcare utilisation of the English population^14^ which are likely to be similar to those in countries with similar economies and population structures. Our research significantly extends the previous definitions of these diseases which were developed in an independent dataset in the following manner:

(a) the original primary care definitions were based on the Clinical Practice Datalink (CPRD) data that uses Read version 2; the algorithms in our framework have been translated to Clinical Terms Version 3 (CTV3) that enables their use in primary care practices that use the TPP software. This was a significant undertaking as CTV3 has more than 100,000 terms compared to Read version 2 and is significantly more extensive. This has wider implications as CTV3 is a component of the SNOMED-CT ontology which is now becoming the de facto standard for primary care EHR; (b) we enriched disease phenotype definitions by adding additional EHR derived fields from the cancer registration datasets in England, Scotland, and Wales; (c) we harmonized and integrated self-reported information from the cancer, non-cancer diseases, procedures, and medication fields collected from UK Biobank participants at recruitment.

For each condition, we created a list of diagnosis or procedure codes from medical ontologies used across all EHR sources. We manually identified relevant self-reported medical history (e.g. self-reported cancer and non-cancer illness) and custom data fields (e.g. “Mental health problems ever diagnosed by a professional”, “Ever been offered/sought treatment for anxiety”) recorded during the initial participant assessment. Primary care definitions translating Read version 2 to CTV3 were bootstrapped by utilizing the NHS TRUD forward mapping files initially. This was followed by manual curation to remove any incorrect or narrow-to-ultrabroad maps that were identified (an example of an incorrect map found in the mapping files and removed by our curation was Read version 2 ZV104 [V]Personal history of malignant neoplasm of ovary which was forward mapped to CTV3 ZV104 “[V]Personal history of malignant neoplasm of genital organ”). Disease registry-based definitions were created by utilizing the ICD-10 codes selected for defining hospital admissions and verifying their use in the respective field of the registry. Clinicians in the team reviewed the lists of diagnosis codes and removed incorrect terms or very broad terms (see Table S3 for an example phenotype) in addition to reviewing the results of the phenotyping validation analyses described below. In some cases, we created individual phenotypes in addition to the combined phenotype (e.g., hypothyroidism and hyperthyroidism within thyroid disorders). Medical ontology terms were further classified as prevalent (e.g. “H/O thyroid disorder”) or incident (e.g. “Nonsuppurative otitis media”) using regular expressions followed by manual review. Phenotypes were grouped and are reported in 16 disease categories broadly mirroring the ICD-10 structure (e.g., malignant neoplasms, digestive system diseases, cardiovascular diseases; Table S4).

We recreated a full cross-source longitudinal history of participants by linking EHR data pre-baseline, questionnaire and other data collected during baseline assessment, or EHR data corresponding to the study follow-up period and identifying all disease events. When temporal information about the event was recorded at a lower resolution between sources (e.g., year of event for self-reported illnesses) or a placeholder date was used by the UKB (e.g., event dates in the same year of a patient’s date of birth), we derived an artificial event date of January 1st of the same year. Records with UKB placeholder dates (such as 07 July 2037, used to flag future dates or other system default dates, or 01 January 1901, used when the original event date preceded the participant date of birth) were excluded from the analyses as the event date was unknown. Sex-dependent phenotypes (e.g., prostate cancer for men) were restricted to records for the relevant sex.

In all analyses, participants were identified as a case if they had at least one record of the phenotype in any of the data sources included in the phenotype definition. Our approach to case ascertainment was specifically designed to maximize case sensitivity (e.g. identify as many true cases as possible). Higher sensitivity is particularly useful for studies that link to diverse data from multiple sources and combine research and EHR data. Participants with no record of the phenotype were considered to be disease-free. The earliest event across all sources was selected for analyses as the first record of the disease.

### Statistical analysis

For each phenotype (n = 313), we created and characterised a disease cohort using descriptive statistics of key demographic factors (e.g., sex, assessment centre country, socioeconomic deprivation/Townsend score), and lifestyle risk factors (e.g., smoking status).

#### Incidence and prevalence

We identified the earliest phenotype event per source (primary care, hospital, cancer registration, death registry, or self-reported medical history) to calculate and report the percentage of cases that are captured in each data source, restricting to events up to 31 March 2026 due to differential data sources coverage. For every phenotype, we defined the median age at first diagnosis as the median age of all participants at their earliest event in any source. We defined crude prevalence at baseline as the proportion of participants in the relevant cohort who had a first event of the phenotype recorded before or at the UKB assessment date, and was stratified by sex. We defined crude incidence rate as the number of new cases during the study follow-up divided by the total person time in years at risk in the cohort, separately by sex and age groups in 5-year bands using age at UKB recruitment.

#### Comparison with external population

We validated the phenotyping algorithms in terms of their epidemiological plausibility by comparing their prevalence estimates in the UKB with the estimates published for an unselected representative external population. The external population was drawn from the CPRD GOLD^15^ accessed through the CALIBER resource^10,16^. We assessed the prevalence of disease in UKB using EHRs and we compared it with the prevalence in the national reference population reported in^14^.

To match the external comparator population, UKB participants were considered to have or have had a condition if they met the criteria of the phenotype before or during the 2010–2015 period. We calculated the UKB period prevalence of the disease and obtained sex-standardised estimates per age group using the 2013 European Standard Population^17^ in the 40–49, 50–59 and 60–69 age groups. Cases were identified in any of the UKB sources, or in EHR sources only (excluding participant questionnaire data or other data collected during the baseline assessment). Participants who died during this period were included in the period prevalence calculation. We generated sex-standardised period prevalence estimates for the phenotypes available in the reference population^14,22^ and for the age bands available in both data sets (Table S5–9).

We evaluated this comparison in two additional analyses: (a) EHR prevalence in the selected UKB cohort stratified by country (England, Wales, and Scotland) using the country where the participants attended the assessment centre to evaluate the prevalence pattern in England, given that only GP practices in England are included in the national reference population study, and; (b) EHR prevalence stratified by socioeconomic deprivation status using the participants’ Townsend deprivation scores from information collected at UKB recruitment and extracted from the baseline field 22189 and presented as quintiles, given that the UKB population is drawn from socio-economically deprived areas, unlike the external population.

#### Sensitivity analyses

To investigate the impact of including self-reported data in the phenotype definitions, we performed a sensitivity analysis where the prevalence in UKB, including self-reported diseases, was compared with the prevalence in the external population. We then compared the disease prevalence without self-reported diseases within the UKB population.

#### Modifiable risk factor associations

The replication of positive and negative associations with modifiable risk factors offer an additional validation layer in our framework. We calculated hazard ratios (HR) of body mass index (BMI), smoking status, and hypertension association with disease onset in Cox proportional hazards models adjusted by age and sex. In disease-specific models for each factor of interest, participants were censored at the first event of the phenotype of interest, end of study follow-up, or death, whichever came first. Missing data were represented by dummy regressors in the factor of interest (BMI, smoking). BMI was represented as a categorical variable, with levels defined as overweight (BMI = 25–29.9), obese (BMI ≥ 30) and underweight (BMI < 18.5) when compared to the reference category defined as healthy BMI (18.5–24.9). Risk factor status at baseline was extracted from the UKB baseline visit (field 21001 for BMI; field 20116 values 0–2 for smoking; field 20002 value 1072 for essential hypertension in addition to the diagnosis hypertension phenotype created with primary and secondary care records^14^. ‘Healthy’ BMI, smoking status ‘never’, or no hypertension (absence of hypertension records) were considered as reference values. HR values with *P* < 0.0002 were considered to show significant association with disease onset after correction for multiple-testing using the Bonferroni method (Tables S10–12).

#### Genetic correlation analyses

To further evaluate the biological robustness of the developed phenotypes, we performed genome-wide association studies (GWAS) in the UKB and compared the results with findings from other published GWAS that did not include UKB as a contributing cohort. We identified ten diseases (bipolar affective disorder, Crohn’s disease, type-2 diabetes, osteoarthritis, peripheral arterial disease (PAD), ovarian cancer, psoriasis, rheumatoid arthritis, schizophrenia, and ulcerative colitis) based on: (a) the presence of existing results from large-scale consortia that exclude the UKB; (b) that had approximately matching phenotypes, and; (c) with a significant proportion of cases contributed from primary care EHR, which are often excluded from the phenotype definitions.

Our UKB GWAS used high-quality genotypes derived from whole genome sequencing data identifying ~ 1.5 billion variants (i.e., single nucleotide polymorphisms (SNPs), indel variants, and structural variants) among 490,640 UKB participants (Li et al. under review). Case–control GWAS analyses of SNPs and small indels were run with REGENIE using a logistic regression model among participants of non-Finnish European (NFE) genetic ancestry, adjusting for age at baseline assessment, genetic sex, sequencing centre, and the first 20 genetic principal components of population substructure. The maximum cohort size across the ten phenotypes was 458,440 participants.

We used LD Score Regression (LDSC) (version 1.0.1) to calculate genetic correlations (*r*_g_) between pairs of GWAS results. Estimates were derived using a reference panel consisting of 1,181,916 genetic variants taken from a sample of *n* = 10,000 unrelated and randomly selected UKB participants of NFE ancestry.

### Shareable tools

Each phenotype is represented in a single, self-contained YAML file that contains all metadata and definitions required to extract relevant information from the database. We developed a phenotyping pipeline using Python version 3.8 and MySQL (Fig. 1). Data extraction was performed using SQL and analyses using Python and R. An interactive data portal with resources, results and visualisations was created using Jekyll. All definitions, code, and documentation are made available under an open source licence as the library ‘Pomegranate’ (https://github.com/spiros/pomegranate-ukbiobank), and in the Pomegranate portal (https://pomegranate.denaxaslab.org/).

## Results

We identified 502,356 eligible participants in UKB and extracted approximately 13.7 million events across all data sources. From these, we identified approximately 5.8 million ‘first’ events (earliest diagnosis date). We created a cohort of 231,303 participants (46%) that had data linked across all EHR sources to calculate the cross-source concordance and the median age at first event. Of these, 156,266 participants (31%) had a single and continuous primary care registration period and age at baseline between 40 and 69 years, and therefore, were included in the baseline prevalence and incidence estimations. A set of 199,373 participants (40%) had linked data across all sources and met the criteria of inclusion in the comparison cohort with the external population. A set of 425,816 participants (85%) of European ancestry had linked genotype data available for genetic analyses (Figure S1, S2; Table 1).

**Table 1:** Description at recruitment of the full UK Biobank population (n = 502,356), and of cases identified in every phenotype in the linked EHR cohort including primary care EHR (n = 213,303) and for a set of exemplar phenotypes.

		Full UKB cohort	Full linked UKB cohort	Actinic keratosis	Bipolar affective disorder	Bronchiectasis	Crohn’s disease	Type 2 diabetes	Osteoarthritis	Peripheral arterial disease	Psoriasis	Ovarian cancer	Raynaud’s disease	Rheumatoid arthritis	Schizophrenia	Ulcerative colitis
n		502,356	231,303	13,059	2145	3519	1579	20,336	64,711	4903	10,411	1306	4219	6376	1258	3369
Sex, n (%)	0	273,293 (54.4)	126,501 (54.7)	5587 (42.8)	1142 (53.2)	1987 (56.5)	867 (54.9)	8214 (40.4)	38,491 (59.5)	1773 (36.2)	5397 (51.8)	1306 (98.5)	2888 (68.5)	4268 (66.9)	575 (45.7)	1724 (51.2)
Sex, n (%)	1	229,062 (45.6)	104,802 (45.3)	7472 (57.2)	1003 (46.8)	1532 (43.5)	712 (45.1)	12,122 (59.6)	26,220 (40.5)	3130 (63.8)	5014 (48.2)	0 (0)	1331 (31.5)	2108 (33.1)	683 (54.3)	1645 (48.8)
Age_baseline_cat, n (%)	40–49	117,799 (23.6)	54,157 (23.5)	783 (6.1)	577 (27.1)	254 (7.3)	354 (22.5)	2433 (12.0)	6494 (10.1)	361 (7.4)	2163 (20.9)	163 (12.6)	904 (21.6)	830 (13.1)	381 (30.4)	662 (19.7)
Age_baseline_cat, n (%)	50–59	167,091 (33.4)	77,347 (33.6)	3171 (24.5)	734 (34.4)	869 (24.9)	544 (34.6)	6095 (30.2)	19,519 (30.4)	1154 (23.8)	3511 (33.9)	427 (33.1)	1330 (31.7)	1993 (31.5)	388 (30.9)	1075 (32.0)
Age_baseline_cat, n (%)	60–69	215,035 (43.0)	98,713 (42.9)	8972 (69.4)	821 (38.5)	2368 (67.8)	674 (42.9)	11,664 (57.8)	38,168 (59.5)	3332 (68.7)	4688 (45.2)	701 (54.3)	1958 (46.7)	3513 (55.4)	485 (38.7)	1618 (48.2)
BMI_cat, n (%)	1	162,348 (32.3)	72,895 (31.5)	4491 (34.4)	556 (25.9)	1211 (34.4)	524 (33.2)	1821 (9.0)	14,688 (22.7)	1183 (24.1)	2848 (27.4)	456 (34.9)	1944 (46.1)	1625 (25.5)	326 (25.9)	1043 (31.0)
BMI_cat, n (%)	2	212,057 (42.2)	97,932 (42.3)	5922 (45.3)	846 (39.4)	1362 (38.7)	656 (41.5)	6835 (33.6)	26,768 (41.4)	1849 (37.7)	4325 (41.5)	480 (36.8)	1533 (36.3)	2441 (38.3)	479 (38.1)	1459 (43.3)
BMI_cat, n (%)	3	122,220 (24.3)	57,928 (25.0)	2533 (19.4)	698 (32.5)	846 (24.0)	367 (23.2)	11,389 (56.0)	22,613 (34.9)	1756 (35.8)	3132 (30.1)	361 (27.6)	634 (15.0)	2208 (34.6)	417 (33.1)	811 (24.1)
BMI_cat, n (%)	4	2626 (0.5)	1131 (0.5)	57 (0.4)	17 (0.8)	53 (1.5)	22 (1.4)	28 (0.1)	193 (0.3)	40 (0.8)	41 (0.4)	5 (0.4)	76 (1.8)	29 (0.5)	16 (1.3)	23 (0.7)
BMI_cat, n (%)	5	3105 (0.6)	1417 (0.6)	56 (0.4)	28 (1.3)	47 (1.3)	10 (0.6)	263 (1.3)	449 (0.7)	75 (1.5)	65 (0.6)	4 (0.3)	32 (0.8)	73 (1.1)	20 (1.6)	33 (1.0)
Smoking, n (%)	0	273,446 (54.4)	126,378 (54.6)	6898 (52.8)	979 (45.6)	1678 (47.7)	703 (44.5)	9099 (44.7)	32,752 (50.6)	1370 (27.9)	4683 (45.0)	753 (57.7)	2228 (52.8)	2951 (46.3)	556 (44.2)	1542 (45.8)
Smoking, n (%)	1	173,001 (34.4)	79,405 (34.3)	5234 (40.1)	700 (32.6)	1444 (41.0)	621 (39.3)	8427 (41.4)	25,112 (38.8)	2152 (43.9)	4180 (40.1)	436 (33.4)	1524 (36.1)	2533 (39.7)	365 (29.0)	1457 (43.2)
Smoking, n (%)	2	52,960 (10.5)	24,300 (10.5)	869 (6.7)	449 (20.9)	363 (10.3)	246 (15.6)	2610 (12.8)	6451 (10.0)	1344 (27.4)	1495 (14.4)	113 (8.7)	445 (10.5)	841 (13.2)	325 (25.8)	352 (10.4)
Smoking, n (%)	3	2949 (0.6)	1220 (0.5)	58 (0.4)	17 (0.8)	34 (1.0)	9 (0.6)	200 (1.0)	396 (0.6)	37 (0.8)	53 (0.5)	4 (0.3)	22 (0.5)	51 (0.8)	12 (1.0)	18 (0.5)
Country, n (%)	E	445,716 (88.7)	185,694 (80.3)	10,627 (81.4)	1830 (85.3)	2952 (83.9)	1243 (78.7)	16,249 (79.9)	52,267 (80.8)	3826 (78.0)	8299 (79.7)	1044 (79.9)	3575 (84.7)	5139 (80.6)	1017 (80.8)	2700 (80.1)
Country, n (%)	S	35,836 (7.1)	27,203 (11.8)	755 (5.8)	178 (8.3)	367 (10.4)	203 (12.9)	2208 (10.9)	6847 (10.6)	654 (13.3)	1203 (11.6)	147 (11.3)	321 (7.6)	663 (10.4)	159 (12.6)	410 (12.2)
Country, n (%)	W	20,804 (4.1)	18,406 (8.0)	1677 (12.8)	137 (6.4)	200 (5.7)	133 (8.4)	1879 (9.2)	5597 (8.6)	423 (8.6)	909 (8.7)	115 (8.8)	323 (7.7)	574 (9.0)	82 (6.5)	259 (7.7)
Townsend_q, n (%)	least_depr	100,627 (20.1)	45,933 (19.9)	3142 (24.1)	283 (13.2)	637 (18.1)	269 (17.1)	2902 (14.3)	12,354 (19.1)	677 (13.8)	1973 (19.0)	240 (18.4)	867 (20.6)	1100 (17.3)	99 (7.9)	645 (19.2)
Townsend_q, n (%)	low_depr	100,067 (19.9)	45,813 (19.8)	3029 (23.2)	292 (13.7)	670 (19.1)	303 (19.2)	3286 (16.2)	12,725 (19.7)	786 (16.0)	1959 (18.8)	274 (21.0)	834 (19.8)	1118 (17.6)	125 (10.0)	626 (18.6)
Townsend_q, n (%)	medium	100,357 (20.0)	47,669 (20.6)	2884 (22.1)	371 (17.3)	725 (20.6)	305 (19.4)	3751 (18.5)	13,469 (20.8)	882 (18.0)	2075 (20.0)	298 (22.8)	853 (20.3)	1229 (19.3)	173 (13.8)	686 (20.4)
Townsend_q, n (%)	high_depr	100,340 (20.0)	46,716 (20.2)	2306 (17.7)	470 (22.0)	685 (19.5)	328 (20.8)	4304 (21.2)	12,899 (20.0)	1028 (21.0)	2167 (20.8)	275 (21.1)	850 (20.2)	1355 (21.3)	256 (20.4)	749 (22.2)
Townsend_q, n (%)	most_depr	100,341 (20.0)	44,828 (19.4)	1682 (12.9)	723 (33.8)	798 (22.7)	370 (23.5)	6051 (29.8)	13,184 (20.4)	1525 (31.1)	2223 (21.4)	219 (16.8)	805 (19.1)	1567 (24.6)	601 (47.9)	661 (19.6)

Sex 0: female, 1: male; 1: healthy BMI, 2:overweight, 3: obese, 4:underweight, 5:missing; 0: smoking status ‘never’; 1: smoking status ‘previous’; 2 smoking status ‘current’: 3: smoking status ‘missing’. Townsend deprivation score: greater values indicate greater indicator of socioeconomic deprivation.

### Counts and age distributions

Cases for 313 phenotypes from 16 categories were identified: benign neoplasms (number of phenotypes = 8); cancers (n = 43); cardiovascular (n = 38); digestive (n = 31); eye (n = 11); ear (n = 3); endocrine (n = 12); genitourinary (n = 23); haematological or immunological (n = 19); infections (n = 27); musculoskeletal (n = 25); neurological (n = 15); perinatal (n = 12); respiratory (n = 17); psychiatric (n = 16) and skin (n = 13) (Tables S4, S5). Median ages at the first event of the phenotype in the fully-linked cohort ranged from 1 years old for cerebral palsy to 71 years old for dementia (see Table S4 for median age at first record for the phenotypes). Ranked correlation with median ages reported in Kuan et al. 2019 table S7A was high (r = 0.9, *p* < 2.2e−16), although median ages were younger in UKB for B12 deficiency, unstable angina, glaucoma, primary stomach cancer, atrial fibrillation or dementia), and older for allergic rhinitis, dermatitis, endometriosis, Crohn’s disease, chronic fatigue, depression, schizophrenia, systemic sclerosis, pancreatitis or liver failure).

### Validation: cross-source representation

**Fig. 2:**
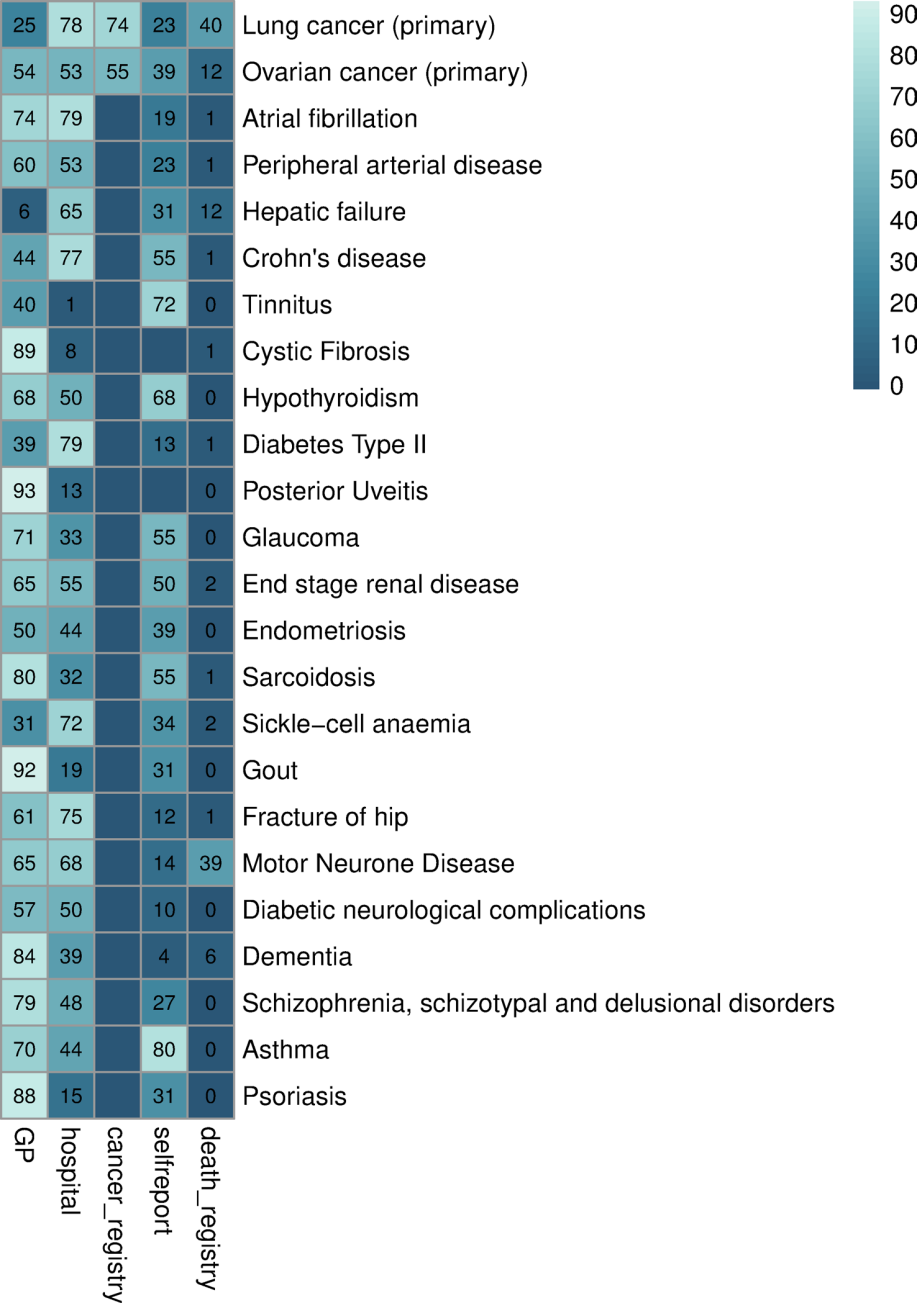
Proportion of patients (%) per phenotype identified in each source including example phenotypes with high case ascertainment in primary care (patients can appear in multiple sources). Cells with no digits denote sources not available in the phenotype definition.

The proportion of participants identified in every source per phenotype showed that phenotypes with cases most highly represented in primary care were vitamin B12 deficiency (92% of their cases), chronic sinusitis (95%) or rosacea (98%), Fig. 2; Table S4). Secondary care showed a high proportion of cases of dilated cardiomyopathy (84%), portal hypertension (96%), mesothelioma (90%) and primary or secondary cancers.The phenotypes most highly represented in the cancer registry were prostate cancer (93%), mesothelioma (90%), breast cancer (89%), colorectal cancer (85%). Phenotypes most highly captured in death records were mesothelioma (65%), pancreatic cancer (59%), lung cancer (40%) and motor neurone disease (39%). Self-reported sources captured cataract (88%), depression (78%) and tinnitus (71%) among others. Primary care had the highest proportion of cases for 50% of the phenotypes, including psoriasis, schizophrenia, alopecia areata, migraine or Raynaud’s disease (Table S4; Figure S3 to S17).

### Validation: baseline prevalence and incidence

**Fig. 3:**
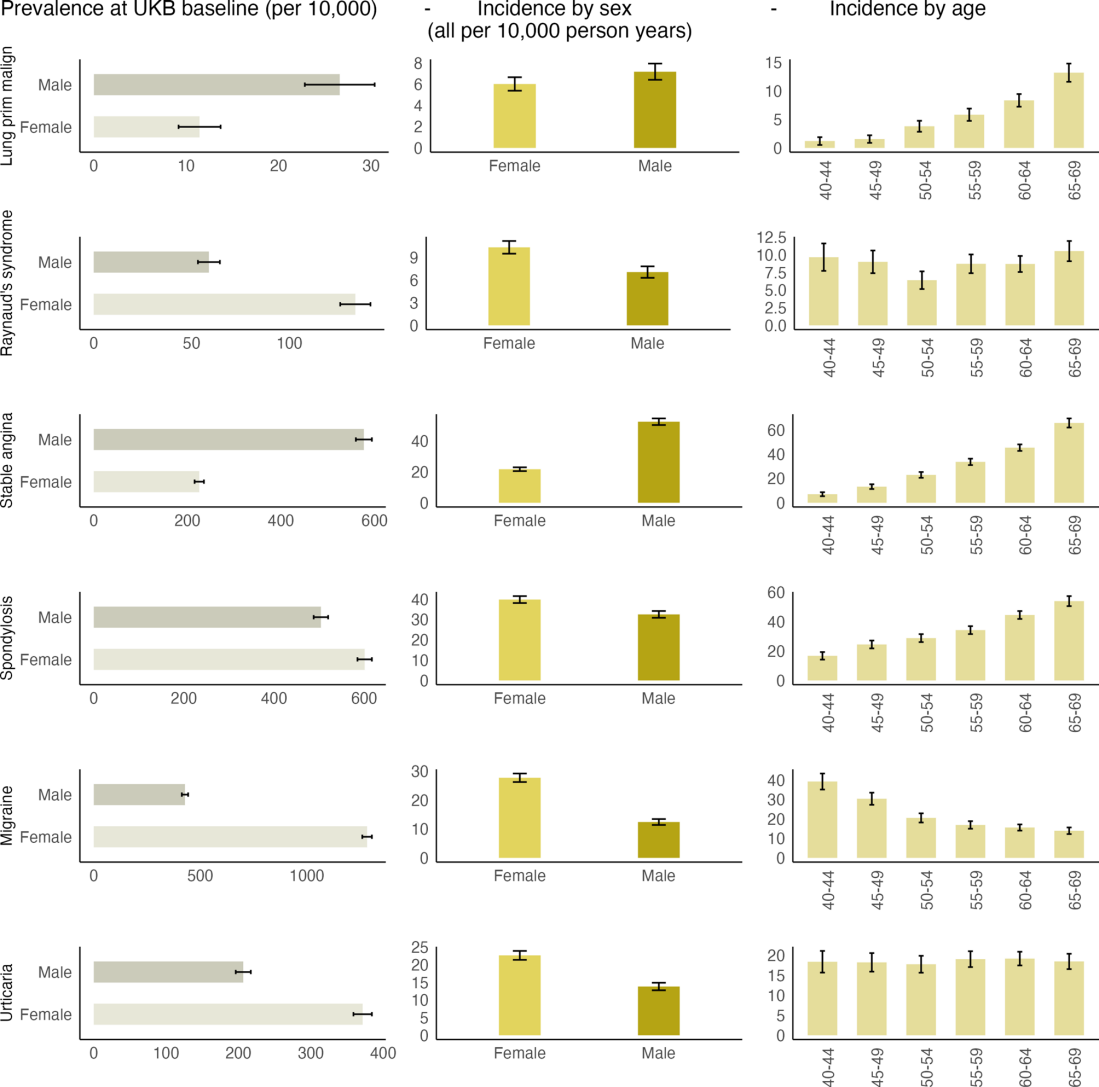
Baseline prevalence, incidence rate (per 10,000 person years) stratified by sex and age at UKB entry for exemplar diseases (error bars are 95% CI).

From the linked records of 156,266 participants and a single GP registration period, we observed highest baseline prevalence in depression at UKB entry (3687 per 10,000 in females; 2447 per 10,000 in males), allergic and chronic rhinitis (3008 and 2630 per 10,000), anxiety disorders (2092 and 1335 per 10,000) and hypertension (2008 and 2609 per 10,000). We observed highest incidence rates during follow up in enthesopathies and synovial disorders (166 per 10,000 for females and 180 per 10,000 for males), hypertension (142 per 10,000 for females and 210 per 10,000 for males), osteoarthritis (148 per 10,000 for females and 122 per 10,000 for males; unadjusted) (Table S5). Our prevalence and incidence estimates revealed age and sex variations per phenotype: cardiovascular diseases like heart failure, intracerebral haemorrhage and peripheral arterial disease were more prominent in males and increased with age. Migraine, spondylosis, Raynauds, urticaria and hypothyroidism were more prominent in females and showed variable age trends (Fig. 3, Figures S18–S20).

### Validation: prevalence comparison with external population

**Fig. 4:**
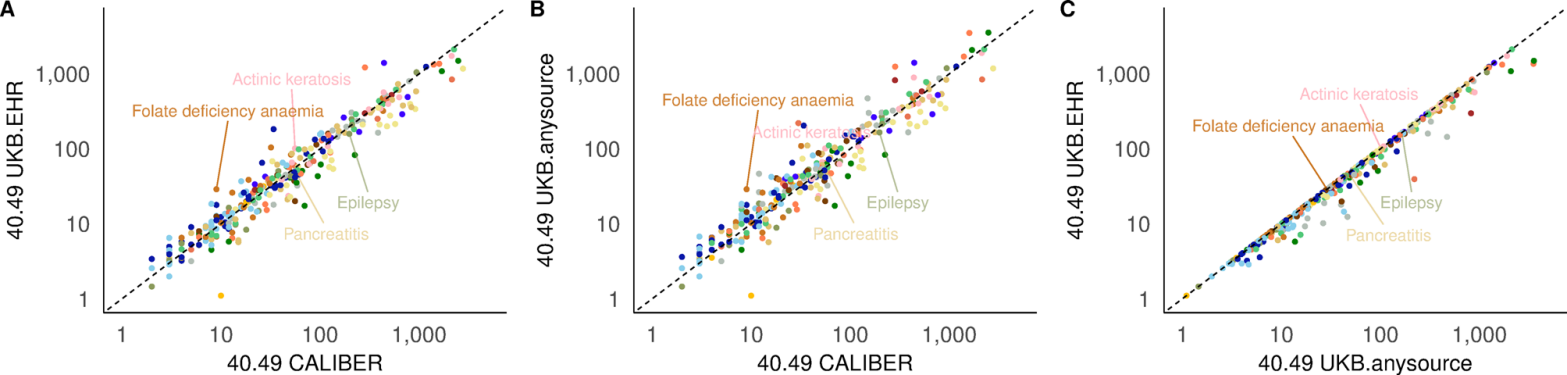
Log_10_ sex-standardised period prevalence for CALIBER and UKB in EHR sources (**A**), CALIBER and UKB in any source (EHR sources and diseases self-reported in the baseline questionnaires) (**B**), and UKB in EHR and UKB in any source (EHR including self-reported diseases) (**C**) for age group 40–49 at UKB baseline, and including example diseases. Colour denotes disease groups.

From 199,373 participants (Table S6) we found that ranked correlations between the CALIBER prevalence and the sex standardised prevalence in the UKB from EHR or all sources was very high (Spearman correlations > 0.95, *p* < 0.001 in all age groups). This was also the case between the prevalence in UKB from EHR versus from all sources (all Spearman correlations > 0.99, *p* < 0.001). Prevalence was comparable and showed fluctuations; it was higher in the CALIBER population (e.g., in asthma, cirrhosis, epilepsy, irritable bowel syndrome or leiomyoma), or in the UKB (e.g., actinic keratosis, ulcerative colitis, sinusitis or osteoporosis; 95% CI non overlapping in the three age bands in relevant pairs; Figs. 4). Including all the sources contributed to a moderate increase in prevalence in a set of phenotypes within the UKB; for instance glaucoma, rhinitis, migraine or rheumatoid arthritis; Table S7. The prevalence patterns between UKB EHR and CALIBER remain when we restrict the analysis to UKB participants in England (all r > 0.95, *p* < 0.001, Figure S21). This suggests that variations by country might not underlie these fluctuations in prevalence (UKB included patients from Wales and Scotland while CPRD captures only English population) (Table S8). Despite UKB participants being less socioeconomically deprived, unlike the CALIBER population, prevalence was highest in the most deprived quintile in the three age bands consistently in 65 phenotypes (20%), including asthma, epilepsy, depression and hypertension, while the prevalence was highest in the least deprived quintile in four phenotypes (1%): actinic keratosis, rosacea, malignant melanoma and other skin cancers (95% CI between relevant age band pairs non overlapping; Table S9).

### Validation: replication of associations with modifiable risk factors

**Fig. 5:**
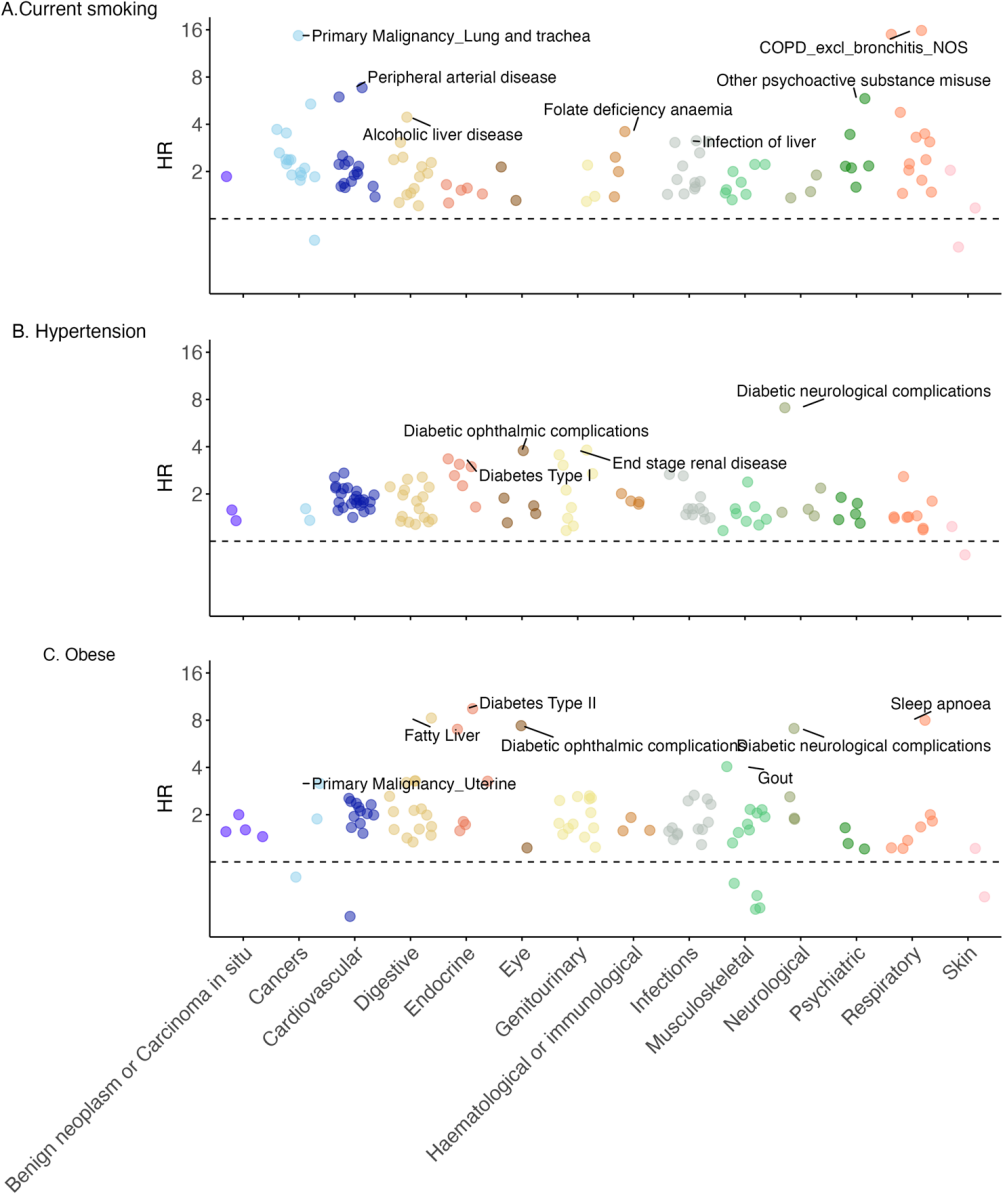
Baseline (UKB recruitment) risk factor associations with disease onset (all *P* < 0.0002) of current smoking (**A**), hypertension (**B**) and obese BMI (**C**).

We examined both positive and negative associations of modifiable risk factors with disease onset. In a cohort of 156,266 participants with linked records, we estimated sex-adjusted and age-adjusted hazard ratios to evaluate the association of smoking, hypertension, and BMI with disease onset. Risk factor-specific models were executed for diseases with at least 100 incident cases during follow-up (n phenotypes = 240; Fig. 5 and Tables S10 to S12; all results after correction for multiple testing described next).

We found 63 associations (25%) between previous smoking status at baseline and disease onset, including lung cancer, COPD, abdominal aortic aneurysm, ulcerative colitis, alcoholic liver disease, and primary oreo-pharyneal cancer. We identified 104 associations (42%) between current smoking status at baseline and disease onset after correction for multiple testing, including COPD, lung cancer, peripheral arterial disease, abdominal aortic aneurysm, and psychoactive substance misuse. 57 of these associations between current smoking status and incident disease were also identified above when the status at baseline was previous smoker (e.g., stable angina, atrial fibrillation, or sleep apnea; Table S10).

We identified 108 associations (45%) between hypertension at baseline and disease onset, with highest associations in diabetes-related neurological or eye complications, end-stage renal disease, glomerulonephritis, type 1 or type 2 diabetes, and chronic kidney disease (Table S11).

We observed 102 positive associations (42%) between obesity at baseline and disease onset after correction for multiple testing and these resembled known associations, including type 2 diabetes, fatty liver disease, sleep apnea, diabetes-related eye or neurological complications, and gout; the association was inverse for Raynaud’s disease. We found 42 associations (17%) between overweight status and disease onset after correction for multiple testing, including fatty liver disease, diabetes-related eye complications, sleep apnea, and gout; an inverse association was observed for osteoporosis. We found 13 associations (5%) between underweight status at baseline and disease onset after multiple testing correction, including Raynaud’s syndrome, osteoporosis, pulmonary fibrosis, chronic obstructive pulmonary disease (COPD), bronchiectasis, lower respiratory tract or urinary tract infections, and some anemias (Table S12).

Likewise, we observed negative associations between modifiable risk factors and disease onset which provided an additional layer of validity in terms of algorithmic accuracy. For example, we did not observe an association between smoking and endometriosis, coeliac disease or vitiligo, nor between obese or overweight BMI and bronchiectasis. Note that lack of association might be influenced by various factors including a low number of incident cases.

### Genetic correlation of phenotypes with external study data

Nine of the ten phenotypes for genetic validation showed significant genetic correlation with their matched published study. Genetic correlation (*r*_g_) ranged between 0.65 and 1.03 across the ten GWAS pairs (Table 2). Five of the ten phenotypes had a genetic correlation of approximately 1, indicating that the genetic structure of the phenotype trait in UKB is nearly perfectly aligned with the matching published study (type 2 diabetes, ulcerative colitis, PAD, Crohn’s disease, schizophrenia). Four phenotypes still had significant genetic correlations with *r*_g_ values largely around 0.80 (psoriasis, rheumatoid arthritis, bipolar affective disorder) or at 0.65 (osteoarthritis). While ovarian cancer had a comparable magnitude of correlation (*r*_g_ = 0.77), the very low heritability for the GWAS reference study of Phelan (2017) (*h*^2^_obs_ = 0.0048) resulted in a non-significant value (*p* = 0.29).

**Table 2:** Genetic validation of selected phenotypes, and the number of cases and controls included in the UK Biobank analysis and in the reference studies.

Phenotype	Published study	*r*_g_	SE	*P* value	UK biobank		Published study	
					# Cases	# Control	# Cases	# Controls
Diabetes, type 2	Scott et al.^45^	0.97	0.03	5.15E−231	37,189	421,251	26,676	132,532
Psoriasis	Tsoi et al.^46^	0.83	0.05	5.14E−63	14,844	443,596	10,588	22,806
Ulcerative colitis	de Lange et al.^47^	0.94	0.07	2.04E−40	6094	452,346	12,366	33,609
Rheumatoid arthritis	Okada et al.^48^	0.76	0.07	1.32E−30	11,961	446,479	14,361	43,923
Peripheral arterial disease	Klarin et al.^49^	1.03	0.09	1.66E−30	8984	449,456	24,009	150,983
Crohn’s disease	de Lange et al.^47^	0.95	0.08	1.84E−30	2992	455,448	12,194	28,072
Osteoarthritis	arcOGEN (2012)	0.65	0.06	2.13E−25	119,948	338,492	14,883	53,947
Schizophrenia	Lam et al.^50^	0.96	0.11	1.88E−17	2165	456,275	33,640	43,456
Bipolar affective disorder	Stahl et al.^51^	0.84	0.17	9.99E−07	3692	454,748	3421	22,155
Primary malignancy, ovarian	Phelan et al.^52^	0.77	0.73	2.93E−01	2375	246,224	29,396	68,502

*r*_g_, genetic correlation; SE, standard error.

### Validation profile

**Fig. 6:**
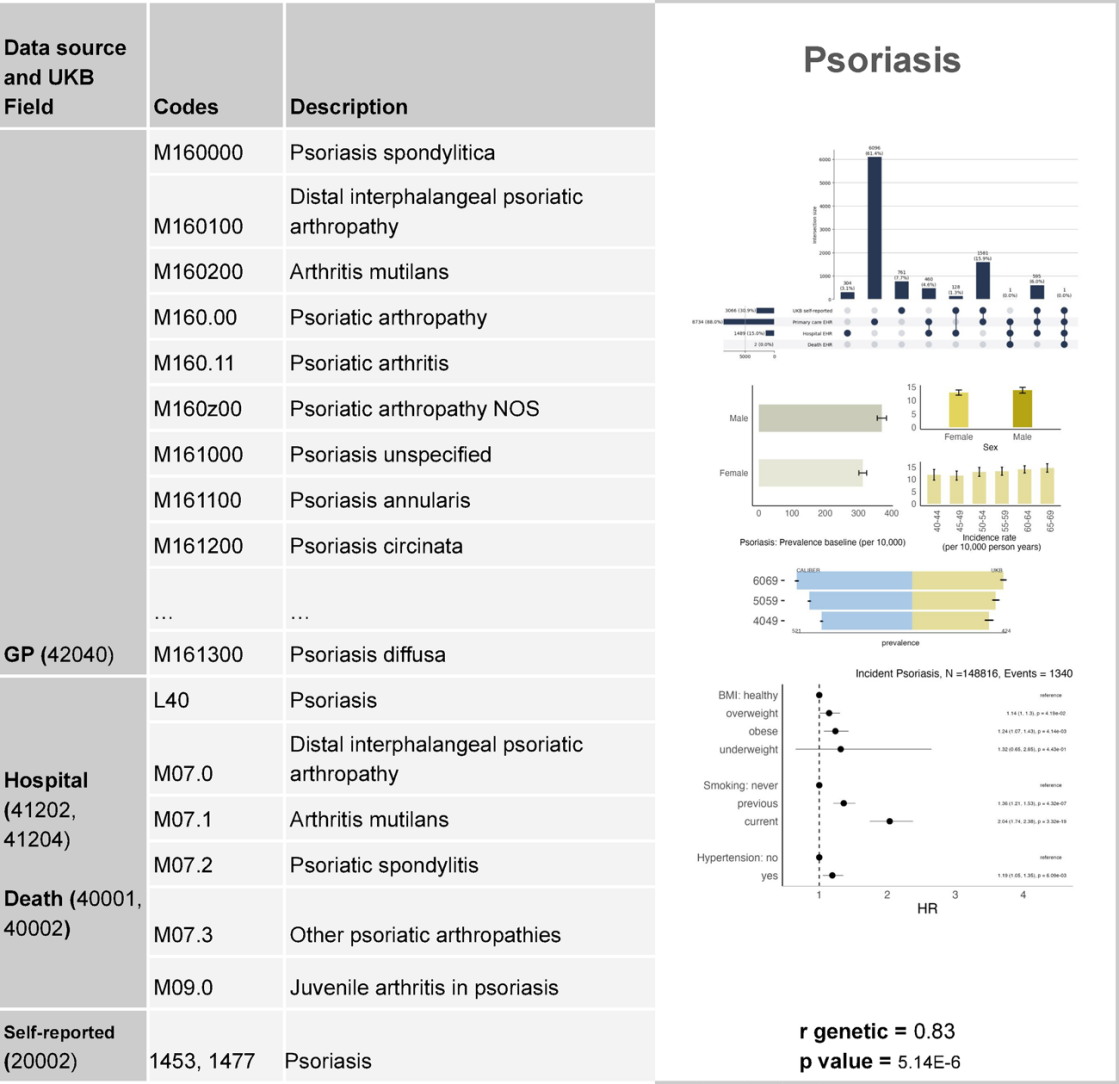
Validation profile for psoriasis.

**Fig. 7:**
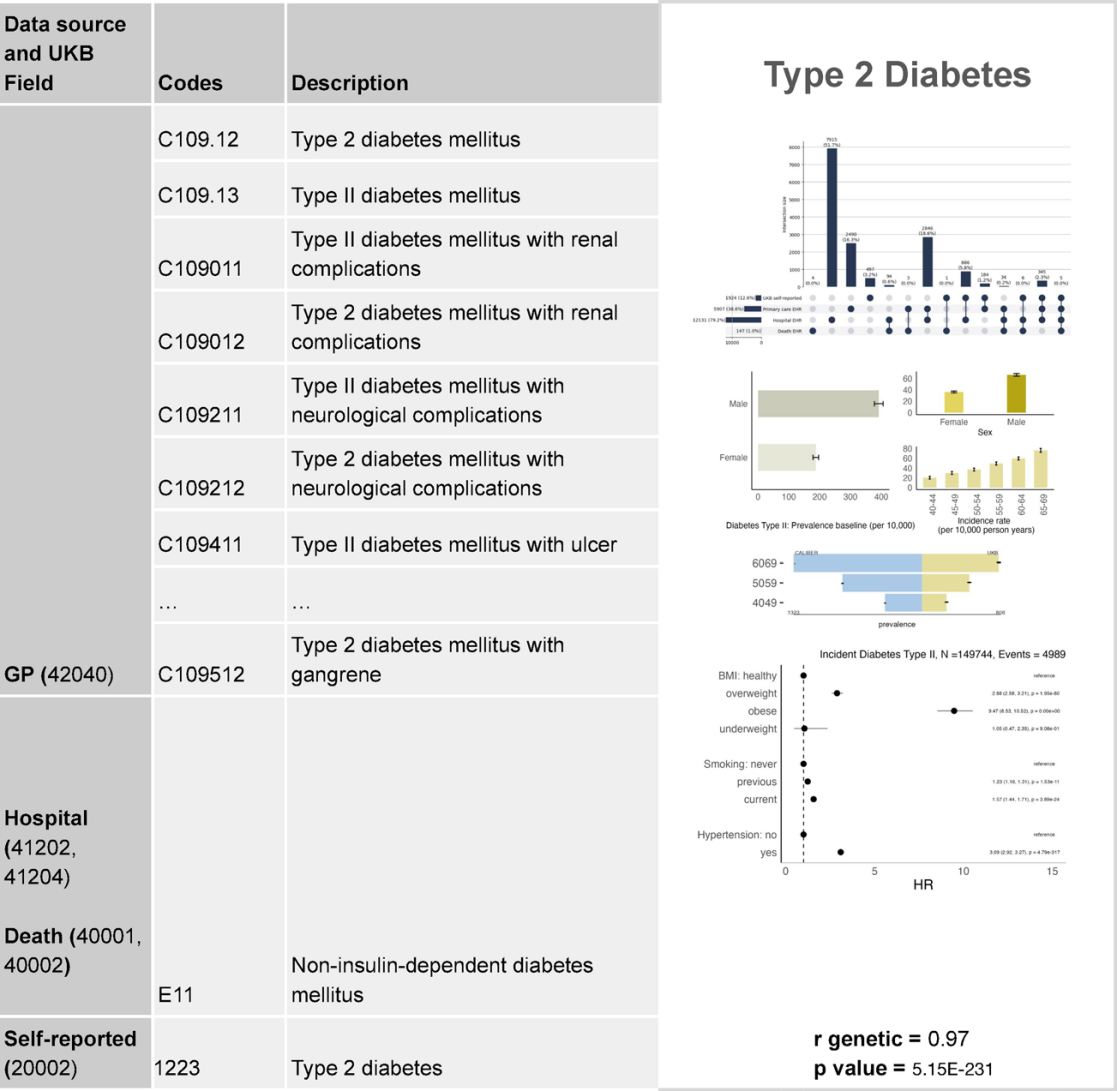
Validation profile for type 2 diabetes.

These analyses aimed to highlight whether the phenotype algorithm captured key characteristics of the disease. The integration of the 5 layers established the phenotype validation profile. This enabled a characterisation of the phenotypic population, and a comparative evaluation between phenotypes as a form of additional validation. Figures 6 and 7 display the profiles of psoriasis, and type 2 diabetes. Psoriasis patients were mostly identified in primary care, current smoking was a risk factor and patients were slightly more prevalent in the CALIBER study cohort. However, type 2 diabetes incidence increased with age more sharply, it was highly more prevalent in men and it was captured in hospitals more frequently; obesity and hypertension were associated risk factors, and the disease was more prevalent in the CALIBER cohort than in the UKB.

## Discussion

In this paper, we presented a novel computational phenotyping framework that was used to define 313 phenotypes in a contemporary biobank cohort by combining multiple EHR sources with medical history questionnaire and physical measure data gathered by UKB. We performed systematic validation at scale of these phenotypes through 5 layers of analyses and established disease specific validation profiles. Our study is novel to provide systematic validation methods simultaneously in a high number of phenotypes within the same framework, including a comparison with an English representative population. We made available our code in the open source library ‘Pomegranate’ (https://github.com/spiros/pomegranate-ukbiobank) to enable reproducible research in other EHR sources. We shared these disease phenotypes, metadata, results, visualisations and validation profiles in the Pomegranate portal (https://pomegranate.denaxaslab.org/).

Our analyses allowed us to identify disease cases within a range of ages consistent with the age distribution described with related phenotypes in a representative population in the CALIBER platform^14^. The fact that the UKB is composed of a middle-age selected population might prevent capture of conditions more common in elderly populations and affect case ascertainment and age patterns of perinatal or young age phenotypes where EHR coverage does not capture back to birth. Unlike the CALIBER study population 1, considered to be representative of the general population^15^, the UKB population was drawn from low socio-economic deprived areas^18–20^. UKB participants have lower indicators of health related characteristics, described as “a healthy volunteer effect”, that affects the generalizability of UKB research findings. However, we observed known age-based patterns in multiple phenotypes related to diseases with appearance in middle-age, with an increase of incidence by age in hypertension, pulmonary embolism and other cardiovascular diseases, bowel or lung cancer, type 2 diabetes and bronchiectasis^21^. We also observed less pronounced influences of age in other diseases, like vitiligo, hidradenitis suppurativa and rosacea^22^, suggesting that our phenotypes can capture key aspects of these diseases in this cohort. We identified known sex-based effects: higher frequency in males for heart failure, atrial fibrillation, dilated cardiomyopathy, and Parkinson’s; likewise for females in osteoporosis, migraine, thyroid disorders which further supports the internal validity of our phenotyping approach.

Our phenotypes allowed us to obtain disease prevalence comparable with the estimates of a national (English) unselected reference population^14^, with fluctuations possibly influenced by different demographic characteristics between the populations and health behaviours derived from them, rather than phenotype limitations^18–20,23^. Despite the fact that the UKB population has been drawn from a higher socioeconomic stratum, we observe the impact of socioeconomic deprivation on disease frequency in a number of phenotypes with highest prevalence in the most deprived group, like hypertension and asthma. The UKB and reference populations are unmatched in terms of demographics factors. 95% of participants in UKB self-identified as of white ethnicity whereas this group represents 68% of the CALIBER study population, and quintiles of Townsend are relative to each cohort such that high socio-economic deprivation in UKB might not represent the same level of high socio-economic deprivation in the national population. Hence results need to be interpreted within the context of the UKB population. Additionally, we identified higher prevalence in UKB for a number of phenotypes when the definitions include self-reported diseases (e.g. osteoporosis, glaucoma). This observation needs to be understood in the context of the differences in the data sources available as self-reported diseases and information from the cancer registry are not captured in the reference population study. Case ascertainment in cancers was in good agreement with their representation in the cancer registry. These comparisons strengthen this rich and systematic phenotyping and analytical approach. They highlight the importance of, on one hand combining multiple data sources to increase case ascertainment, and on the other hand, implement analytical procedures to evaluate information consistency.

Our approach to case ascertainment aimed to maximize the sensitivity of cases and as a result is potentially more prone to noise and misclassification problems that can be observed in EHR data. One of the primary reasons however that our framework provides multiple validation layers was to enable researchers to identify and characterise such potential issues. Non-deterministic phenotyping algorithms, such as machine learning and artificial intelligence methods, can potentially be utilized to further improve mitigation against such artifacts in EHR data. Additionally, it is important to note that all disease phenotypes that were examined in this study were defined in an adult population (the UK Biobank primarily recruited participants 40–69 years old). Researchers implementing our approach in younger populations or special groups such as paediatric cohorts or rare disease cohorts in the UK Biobank would face challenges related to lower disease incidence rates, limited longitudinal data capture from early life, incomplete phenotypic expression at younger ages, and potentially different medical ontologies or terminologies specific to paediatric or rare conditions.

Despite the observation of lower association between UKB baseline hypertension and cardiovascular mortality compared to a control surveyed England population^19^, our phenotypes reproduced known risk factors associations between smoking and COPD, lung cancer, PAD, and abdominal aortic aneurysm^24,25^, rheumatoid arthritis^26^, higher burden of respiratory infections^27,28^, tinnitus^29^ or psoriasis^30^, PAD^25^ or dermatitis^31^. We reproduced associations between obesity and incident fatty liver disease^32^, type-2 diabetes, sleep apnea^33^, rheumatoid arthritis^34^, and psoriasis^35,36^ and urticaria^37^. Note that although the case representation in these phenotypes was diverse, they were most frequently represented in EHR sources, hence associations are likely not driven by self-reported cases. Associations were reproduced between hypertension and incident diabetic eye or neurological complications, end-stage renal disease, type 1 diabetes or dermatitis^38^. We observed negative associations between smoking and endometriosis^39,40^, coeliac disease or vitiligo^36^, and between obese and overweight BMI and bronchiectasis. Note that these analyses do not intend to address causality but rather evaluate whether known associations are reproduced so they might implicate reverse causality or might involve additional factors as mediators.

Nine of the diseases selected for GWAS in UKB had significant genetic correlations with matched published GWAS, indicating that the phenotype pairs are measuring similar genetic architecture. Five of the phenotypes had a nearly perfect genetic correlation (*r*_g_ ≅ 1). While osteoarthritis and rheumatoid arthritis, psoriasis or bipolar affective disorder had significant genetic correlation results, their relatively lower estimates (*r*_g_ < 0.9) may indicate more heterogeneity in: (1) our phenotype definitions; (2) the external, published phenotype definitions, and/or; (3) both factors. We found that a higher number of patients were identified in self-reported sources of bipolar affective disorder compared to cases identified in EHR alone, which suggests that the UKB disease population might be more heterogeneous than that captured in the reference GWAS. Finally, the high but non-significant genetic correlation for the ovarian cancer GWAS illustrates that sufficient heritability in both sets of input summary statistics is necessary for a stable estimate.

Overall, although UKB is drawn from a wealthy population considered not representative of the general population^18^, we observed key age and sex patterns and high ranked correlations with age and prevalence in a reference population. We established risk factors associations, and identified participants with similar genetic architecture as studies in other biobanks. Despite the fact that interpretations should be specific to the UKB population, our study focused on these 313 phenotypes sheds light on where to be cautious about the generalisation of UKB findings. Given the relatively short follow up period that the UΚ Biobank provides, it’s important to acknowledge that our research has focused on identifying individual disease cases rather than disease subtypes based on chronicity e.g. slow or fast progressors of a particular disease.

Our novel and modular computational phenotyping framework, exemplified in this work in the UKB, allowed us to leverage the richness of biobank data. This framework contributed to capturing patients’ clinical diversity more comprehensively than when a single source is used, and enabled the discovery of novel markers of disease risk; see^41^ where they report the ability of protein signatures to predict the onset of 67 diseases. Capturing patients’ clinical diversity more comprehensively by consolidating phenotypes may lead to stronger genetic associations and merits further exploration in future studies.

By combining multiple sources and clinical events this framework goes beyond phenotypic approaches based on a single data source (e.g.^42,43^), and expands algorithmic approaches provided by the UKB, where first occurrences of clinical events were mapped to broad health outcomes (https://biobank.ndph.ox.ac.uk/showcase/refer.cgi?id=596). UKB internal phenotype methods provide algorithmic defined outcomes for a small set of diseases. We provide 313 phenotypes and we implement methods to identify prevalent and incident patients from multiple sources of data. UKB first occurrence phenotyping methods mapped events to 3 digits ICD codes then named as a broad disease definition. Our methods preserve the granularity of diagnoses in every source and allow disease cohorts to be researched as a whole or stratified within each source. Our framework expands other phenotyping approaches (Yeung et al. 2022; Neale lab, http://www.nealelab.is/uk-biobank/; PHESANT,^44^) by providing extensive phenotype validation and describing the phenotypic population.

Our multiple layers combined allowed us to establish the validation profile of the phenotype. This provided a detailed characterisation of the phenotypic population at scale, novel in the EHR phenotyping methods literature. They complement the benefits of robust and reproducible phenotyping methods, because they describe the disease population within the same process. This expands the information provided in phenotyping resources like HDR UK (https://phenotypes.healthdatagateway.org/), OpenCodelists (https://www.opencodelists.org/) and has potential to benefit the wider research community if the profile is incorporated in the phenotyping reporting standards in research papers or phenotype libraries.

Our approach is key in many aspects. The validation profile enables comparisons between populations, e.g., if a different age or sex distribution in a new phenotype cohort is accompanied by a different risk profile. The validation profile also enables one to observe the impact when a data source is not available in a new study, and whether the population captured has different characteristics if the differential source is related to disease severity. In the context of studying diseases at scale, the validation profiles enable us to observe trends to enhance our understanding of disease determinants, when groups of diseases are affected differently by age, sex or modifiable risk factors.

Finally, this framework enables the study of diseases systematically using reproducible methods across biobanks. The UK Biobank was chosen as a case study as it exemplifies the characteristics of most contemporary biobank research studies e.g. rich baseline phenotypic assessment information combined with linkages to cross-source EHR data for long-term health status follow-up of participants. Adapting the framework and methods described in this manuscript to additional data sources in other studies (e.g. Genes and Health, Our Future Health, both in the UK) would require manual mapping of the respective research fields of interest (e.g. self-reported diagnoses) and harmonization of ontologies used in EHR recording. This process while resource intensive could potentially be assisted by the fact that ICD-10 is one of the most common classification systems used to record information in hospital and CTV3 is a subset of SNOMED-CT which is a well-established international standard.

Our framework exemplified in the UKB, a flagship contemporary biobank study, including the comparison with a national English population, enables comparable biomedical research in underserved and across nations populations As most contemporary biobanks now heavily rely on multi-source linked electronic health record data for following up participants, our framework provides guidance on how to systematically create disease phenotypes and do case ascertainment in a semi-automatic way to enable harmonized research efforts across biobanks.

## Supplementary Information

Supplementary Information.

## Data Availability

UKB data were obtained following approval of the protocol by the UKB Access Management Committee (https://ams.ukbiobank.ac.uk/ams/) (Application Numbers 58356 and 20361). To request access UKB data, researchers need to apply directly to the UK Biobank, register in their access management system and submit a research study protocol https://www.ukbiobank.ac.uk/enable-your-research/apply-for-access. All definitions, code, and documentation are made available under an open source licence as the library ‘Pomegranate’ (https://github.com/spiros/pomegranateukbiobank), and in the Pomegranate portal (https://pomegranate.denaxaslab.org/).
