# Supplementary material for: A computational framework for defining and validating reproducible phenotyping algorithms of 313 diseases in the UK Biobank
Source: Sci Rep. 2025 Jul 9;15:24607. doi: 10.1038/s41598-025-05838-9 (PMC12241469; doi:10.1038/s41598-025-05838-9)
Supplement: Supplementary file 1 — Supplementary Information. [file 41598_2025_5838_MOESM1_ESM.zip › Supplementary/SuppInfo_SciReports_Torralbo_ComputationalFrameworkPhenotypingValidating303UKB1.pdf]

Supplementary Information

Figure S1. Flow diagram with steps to define the study population. From the total UK Biobank cohort, participants with full EHR linked data were selected to identify the representation of the diseases across all sources (orange box). From these, participants with a continuous single GP registration period and 40-69 years old at UKB baseline were selected to estimate baseline prevalence and disease incidence. From participants with liked EHR data, participants that matched the selection criteria of Kuan *et al.* (2019) CALIBER population were selected to evaluate phenotype prevalence. From the total UK Biobank cohort, participants with linked genetic information were selected to identify disease cases in relevant traits and perform genetic validation (dark orange box).

Figure S2. Participants distribution per data source over time from 1990 from the total number of participants with data linked in all data sources and phenotype events in the study.

Figure S3. Proportion of patients per phenotype identified in each source in the Cardiovascular group

Figure S4. Proportion of patients per phenotype identified in each source in the Digestive group

Figure S5. Proportion of patients per phenotype identified in each source in the Cancer group

Figure S6. Proportion of patients per phenotype identified in each source in the Benign neoplasm or Carcinoma group

Figure S7. Proportion of patients per phenotype identified in each source in the Ear group

Figure S8. Proportion of patients per phenotype identified in each source in the Endocrine group

Figure S9. Proportion of patients per phenotype identified in each source in the Eye group

Figure S10. Proportion of patients per phenotype identified in each source in the Genitourinary group

Figure S11. Proportion of patients per phenotype identified in each source in the Infections group

Figure S12. Proportion of patients per phenotype identified in each source in the Haematological group

Figure S13. Proportion of patients per phenotype identified in each source in the Musculoskeletal group 35

Figure S14. Proportion of patients per phenotype identified in each source in the Neurological group

Figure S15. Proportion of patients per phenotype identified in each source in the Psychiatric group

Figure S16. Proportion of patients per phenotype identified in each source in the Respiratory

Figure S17. Proportion of patients per phenotype identified in each source in the Skin groups

Figure S18. Baseline prevalence, incidence by age and sex for phenotypes in the Cardiovascular group

Figure S19. Baseline prevalence, incidence by age and sex for phenotypes in the Skin group

Figure S20. Baseline prevalence, incidence by age and sex for phenotypes in the Endocrine group

Figure S21. Log 10 -transformed sex-standardised period prevalence for UKB EHR participants from England recruitment centres and CALIBER (A), prevalence variation by country (England, Wales or Scotland) and prevalence by socioeconomic status (least and most deprived quintiles of Townsend scores at UK B entry) in 2_selected diseases for age bands 40-49, 50-59, 60-69.

Supplementary Tables:

Table S1. Linked EHR sources and the end of their coverage. Primary care EHR censoring dates for England data providers England Vision and England TPP respectively. Dates are specified as DD-MM-YYYY.

Table S2. Coding systems and data providers in primary care

Table S3. Codelists of the phenotypes and UKB Fields for ovarian cancer.

Table S4. Proportion of cases per source and median age at first record from patients with full linked data in all EHR sources from their events up to 31 March 2016 (N = 226,353). Patients could be represented in multiple sources. - indicates that the phenotypes did not include codes of that source. -- denotes N < 5

Table S5. Cases in the full UK Biobank (N = 502,356), cases with full linked EHR data (N = 231,303), prevalence at baseline, incidence rate by sex and age groups (per 10,000 person years) derived from participants with full linked EHR data and a single continuous primary care registration (N = 156,266). -- denotes N < 5

Table S6. Number of participants and cohort characteristics of the UKB cohort compared with the national external population (CALIBER)

Table S7. Sex-standardised period prevalence per 10,000 persons obtained in UKB from all sources, EHR sources and estimates reported for CALIBER in the Table S4 in Kuan et al, 2019, stratified on age on 1 April 2010; 95% CI in parenthesis

Table S8. Sex-standardised period prevalence per 10,000 persons obtained in UKB in any source, in UKB in EHR stratified by country of assessment centre

Table S9. Sex-standardised period prevalence per 10,000 persons obtained in UKB in any source, in UKB in EHR stratified by deprivation quintile at UKB entry

Table S10. Age and sex adjusted hazard ratio of smoking association with incident disease (reference value is smoking status = never); all $p < 0.0002$

Table S11. Age and sex adjusted hazard ratio of hypertension association with incident disease (reference value is no hypertension); all $p < 0.0002$

Table S12. Age and sex adjusted hazard ratio of BMI association with incident disease ($P < 0.002$) (reference value is healthy BMI)

Figure S1. Flow diagram with steps to define the study population. From the total UK Biobank cohort, participants with full EHR linked data were selected to identify the representation of the diseases across all sources (orange box). From these, participants with a continuous single GP registration period and 40-69 years old at UKB baseline were selected to estimate baseline prevalence and disease incidence. From participants with liked EHR data, participants that matched the selection criteria of Kuan *et al.* (2019) CALIBER population were selected to evaluate phenotype prevalence. From the total UK Biobank cohort, participants with linked genetic information were selected to identify disease cases in relevant traits and perform genetic validation (dark orange box).

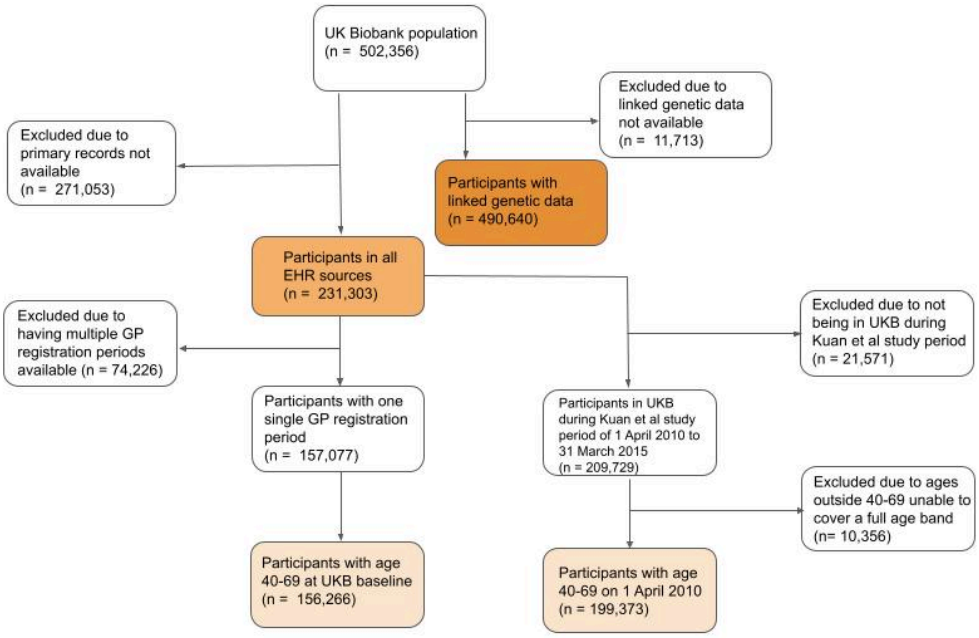

Figure S2. Participants distribution per data source over time from 1990 from the total number of participants with data linked in all data sources and phenotype events in the study.

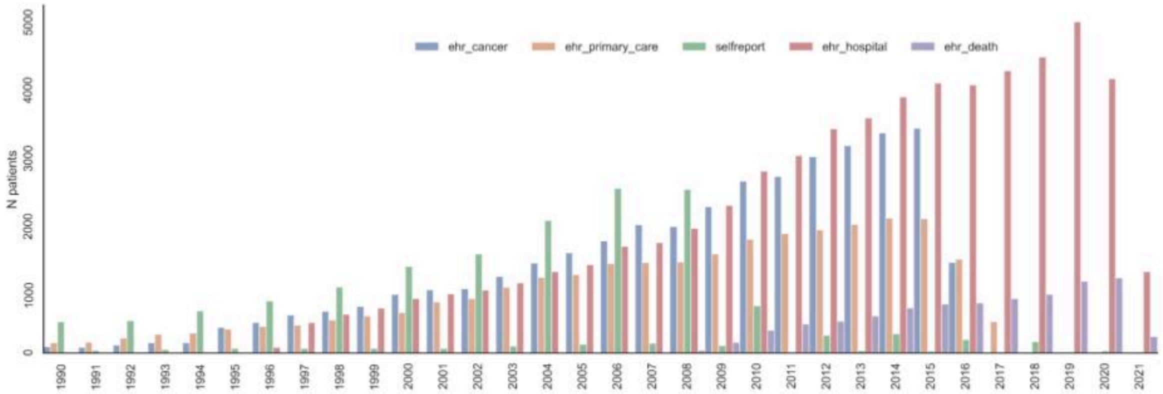

Figure S3. Proportion of patients per phenotype identified in each source in the Cardiovascular group

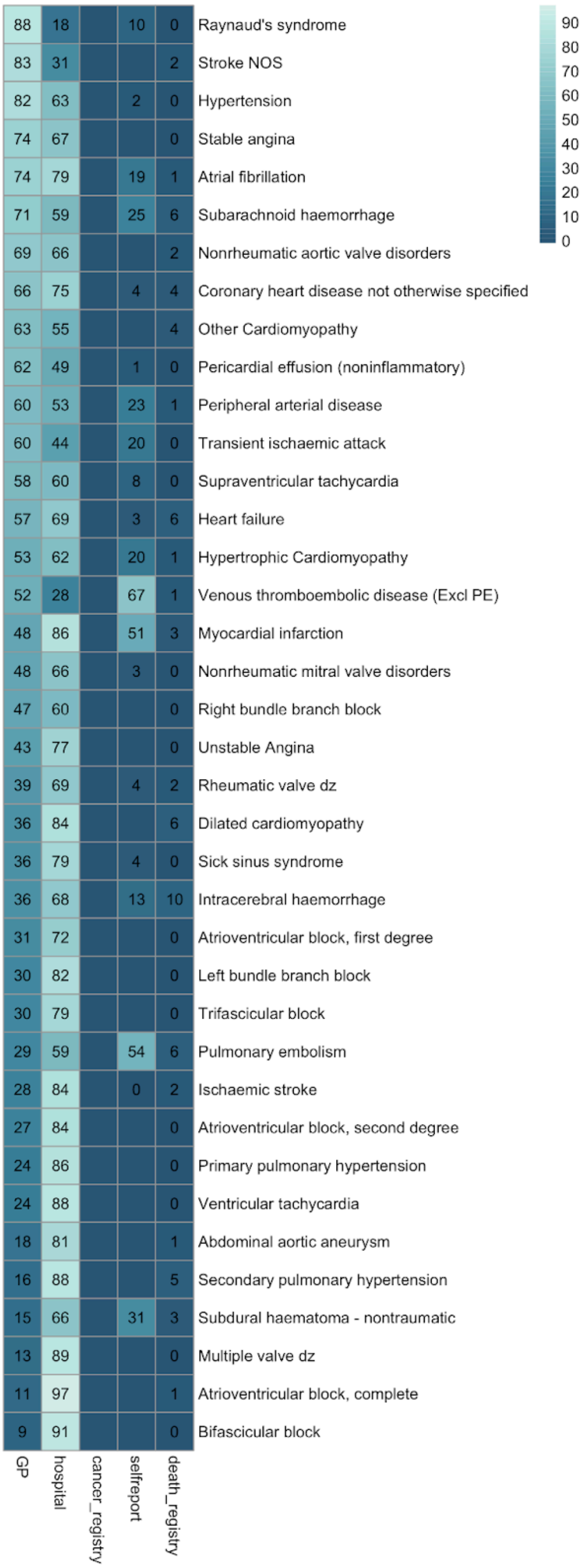

Figure S4. Proportion of patients per phenotype identified in each source in the Digestive group

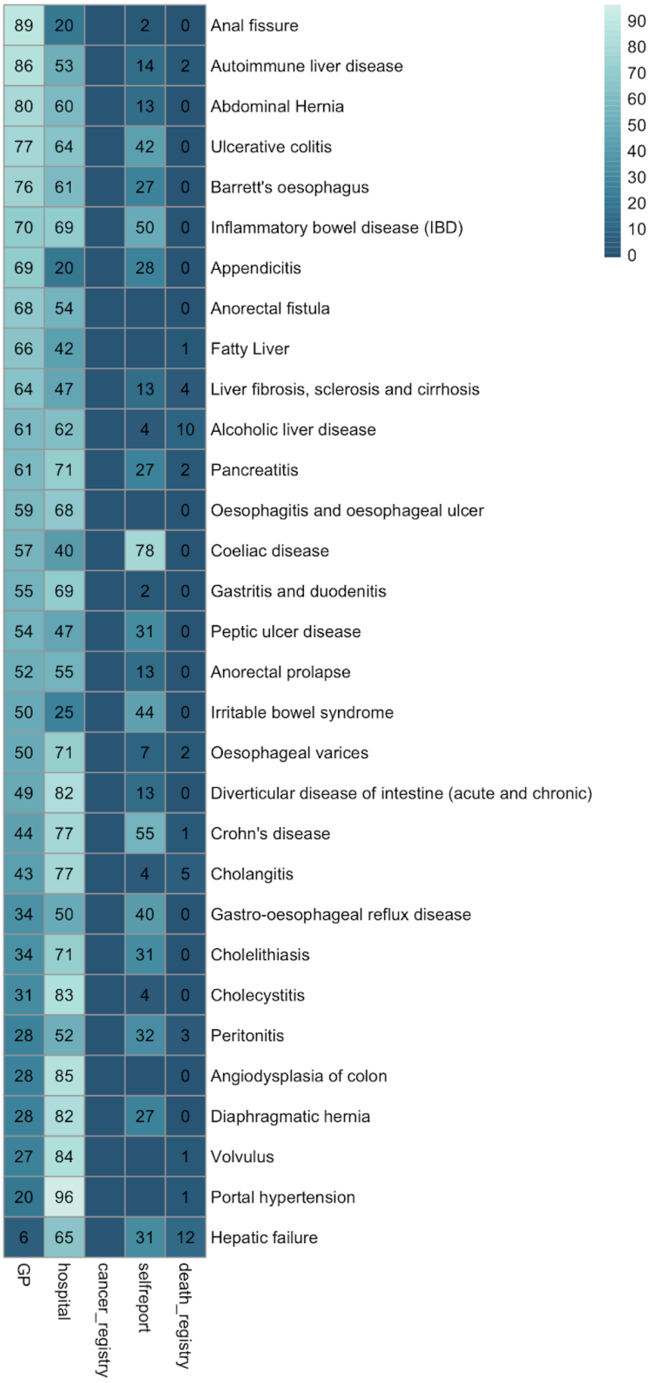

Figure S5. Proportion of patients per phenotype identified in each source in the Cancer group

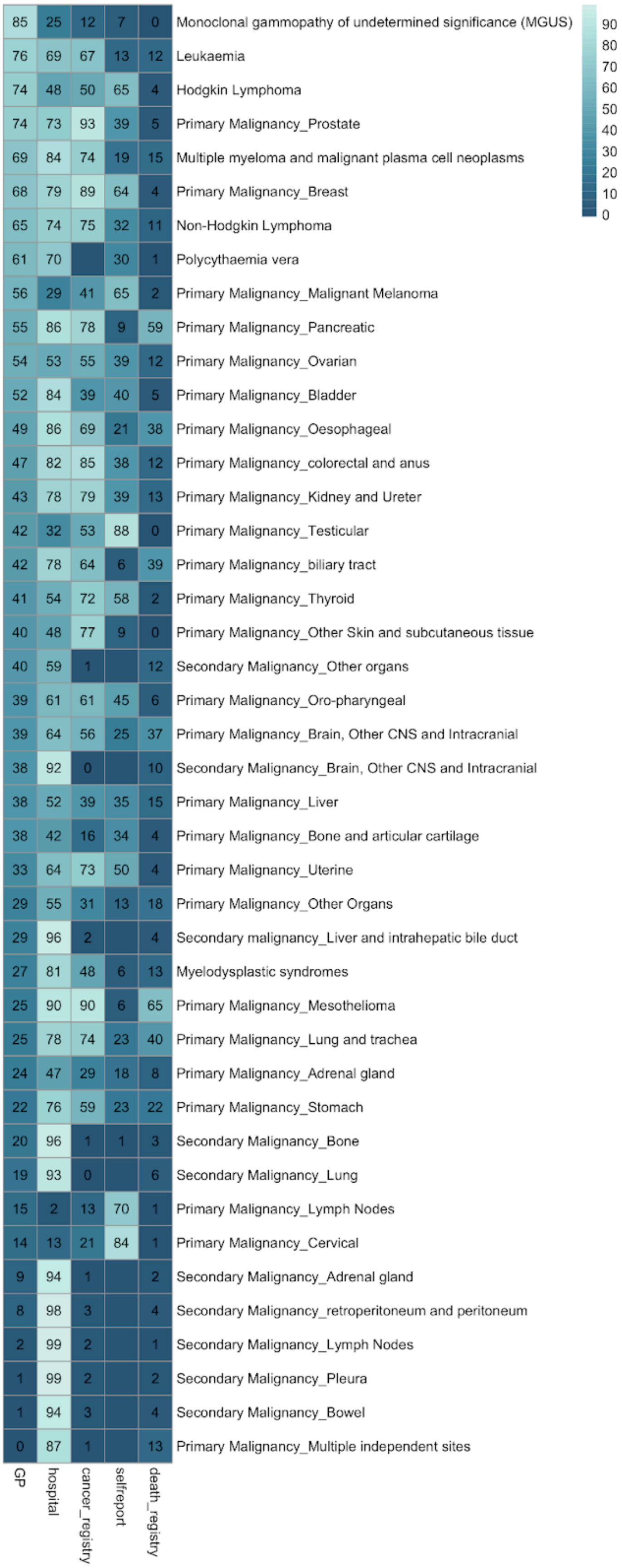

Figure S6. Proportion of patients per phenotype identified in each source in the Benign neoplasm or Carcinoma group

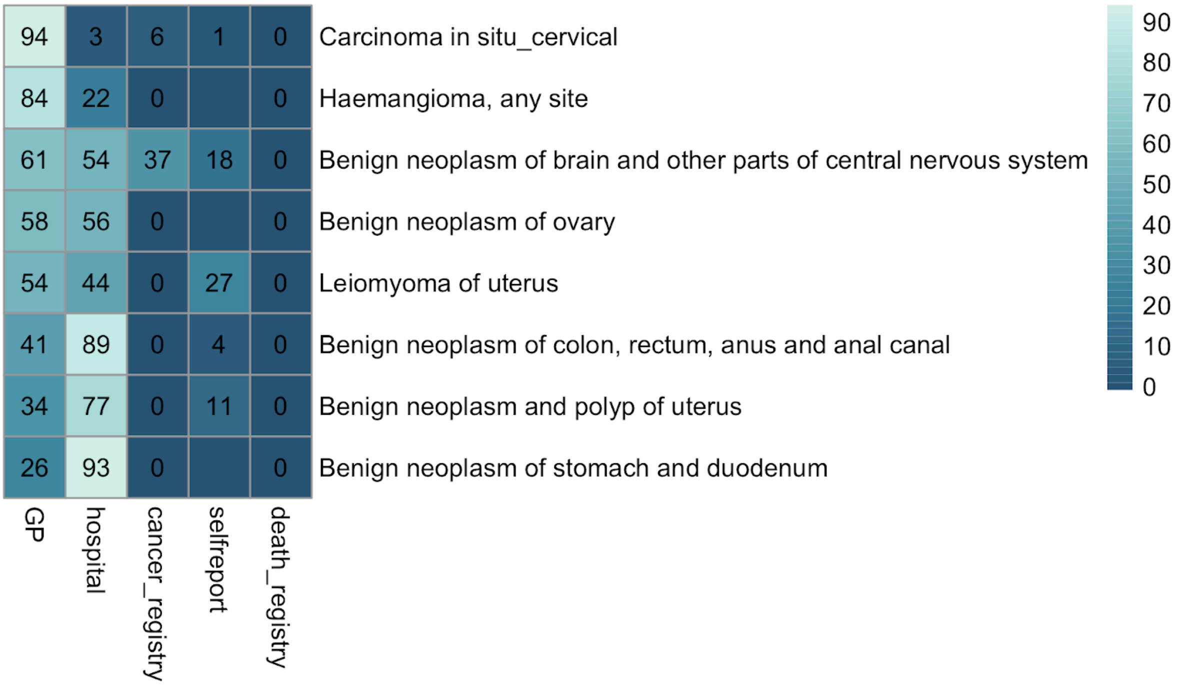

Figure S7. Proportion of patients per phenotype identified in each source in the Ear group

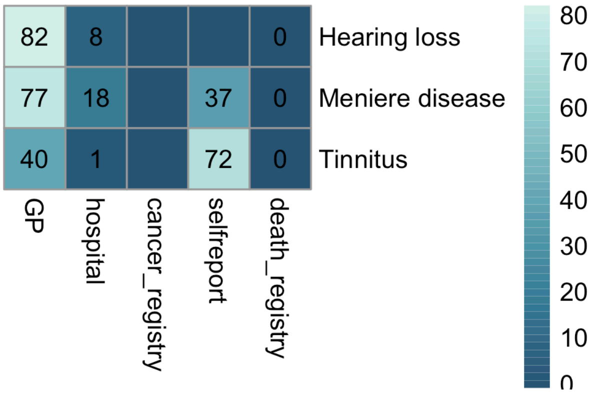

Figure S8. Proportion of patients per phenotype identified in each source in the Endocrine group

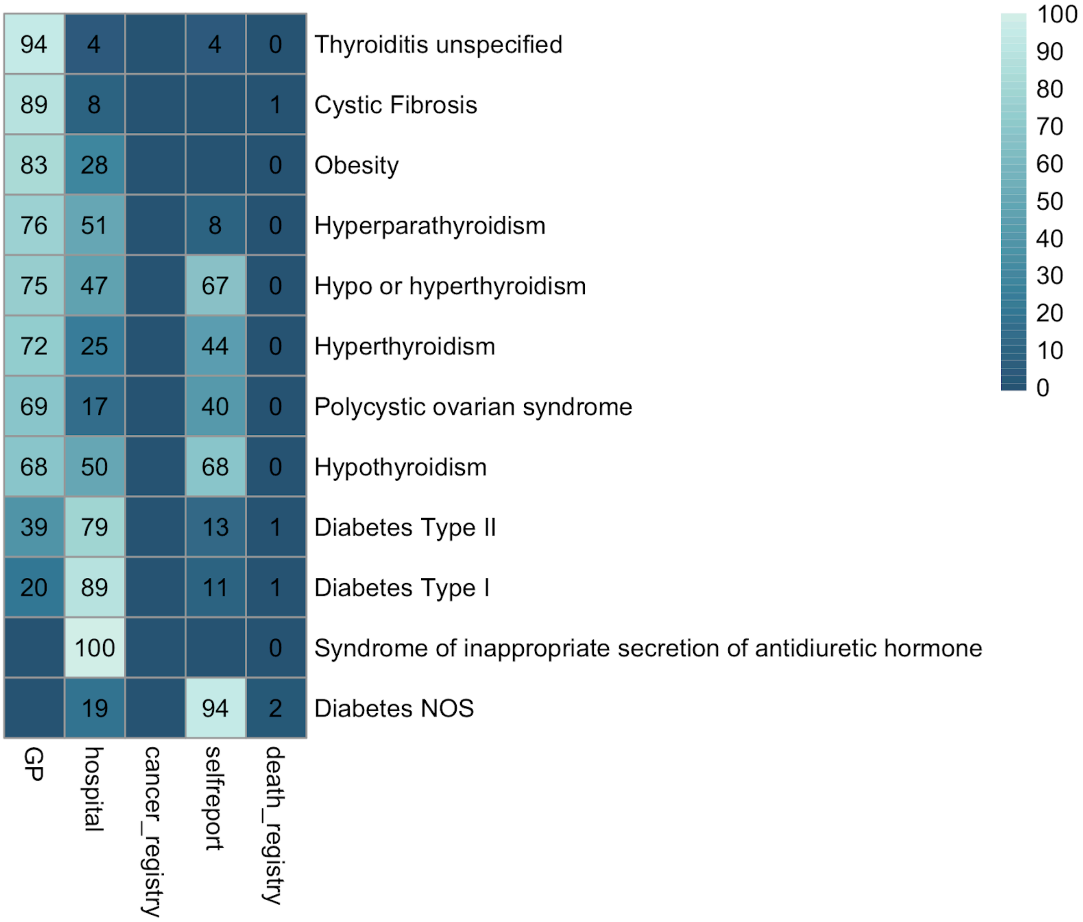

Figure S9. Proportion of patients per phenotype identified in each source in the Eye group

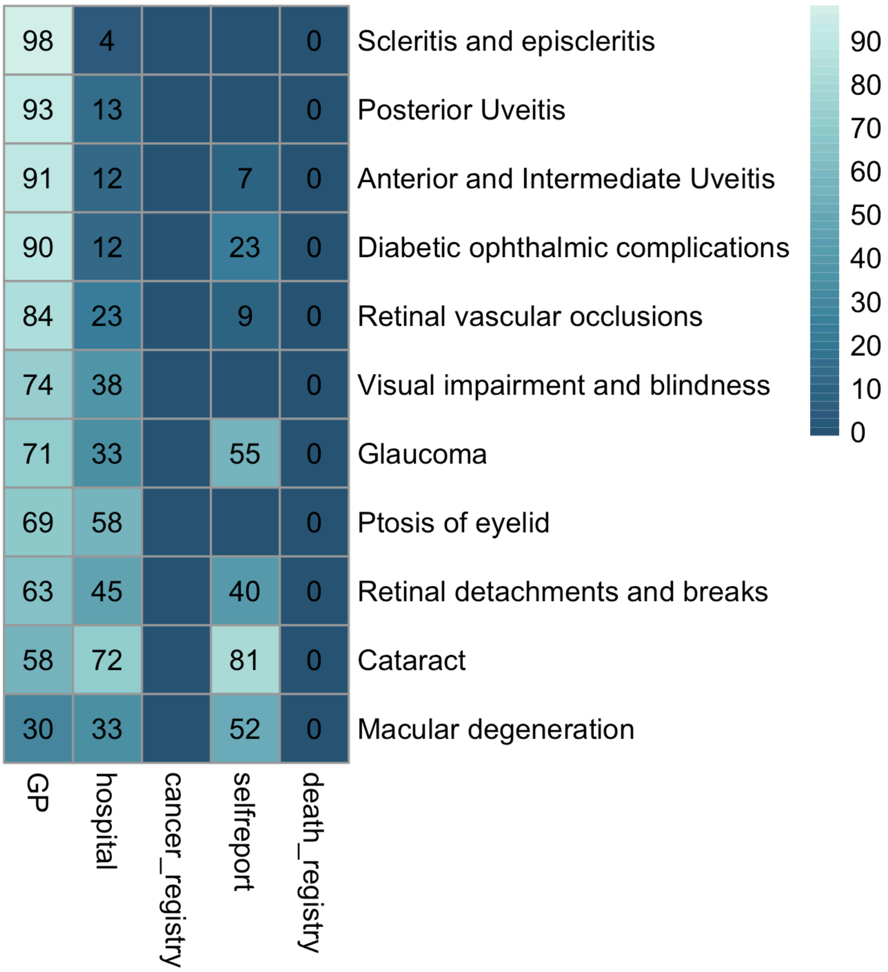

Figure S10. Proportion of patients per phenotype identified in each source in the Genitourinary group

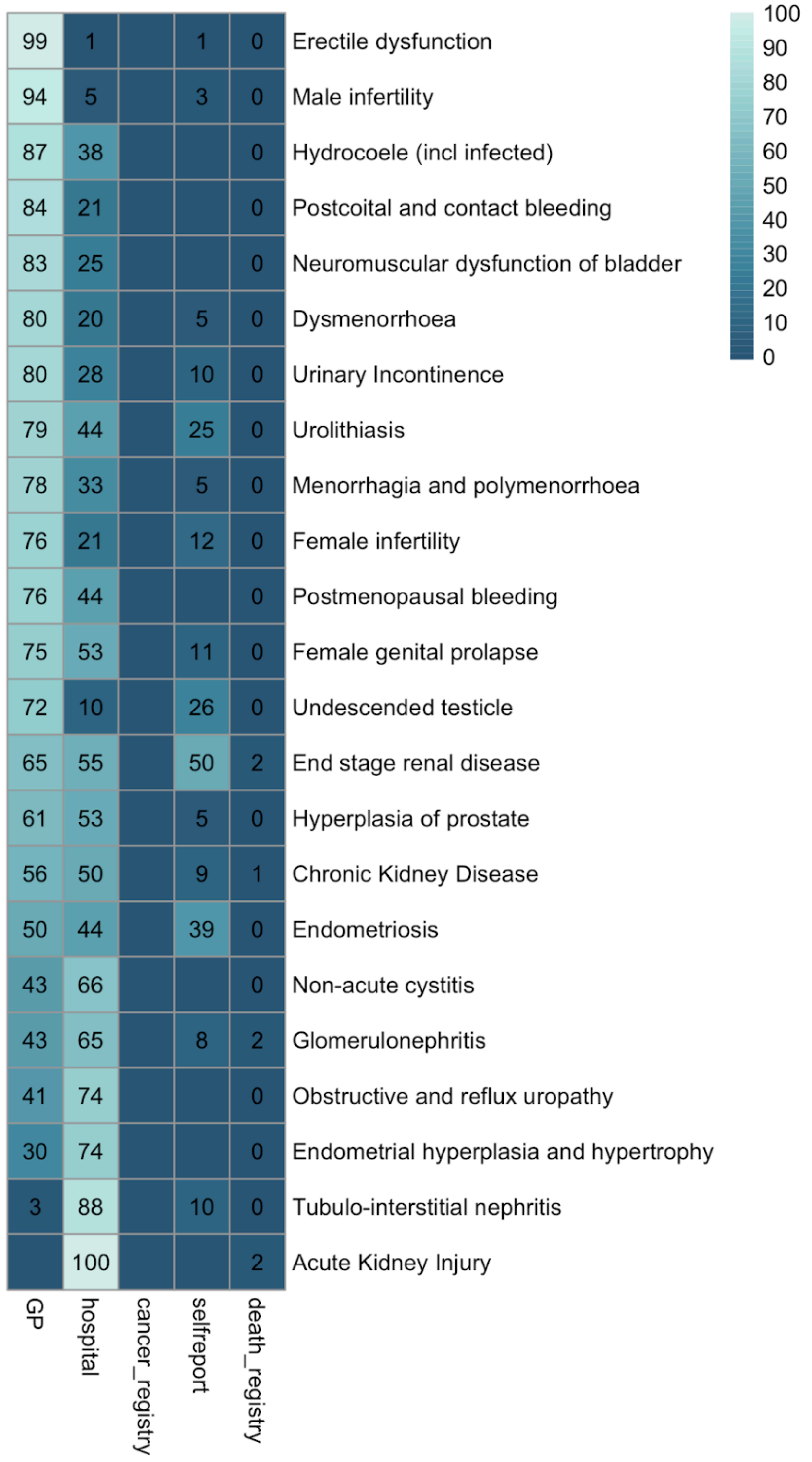

Figure S11. Proportion of patients per phenotype identified in each source in the Infections group

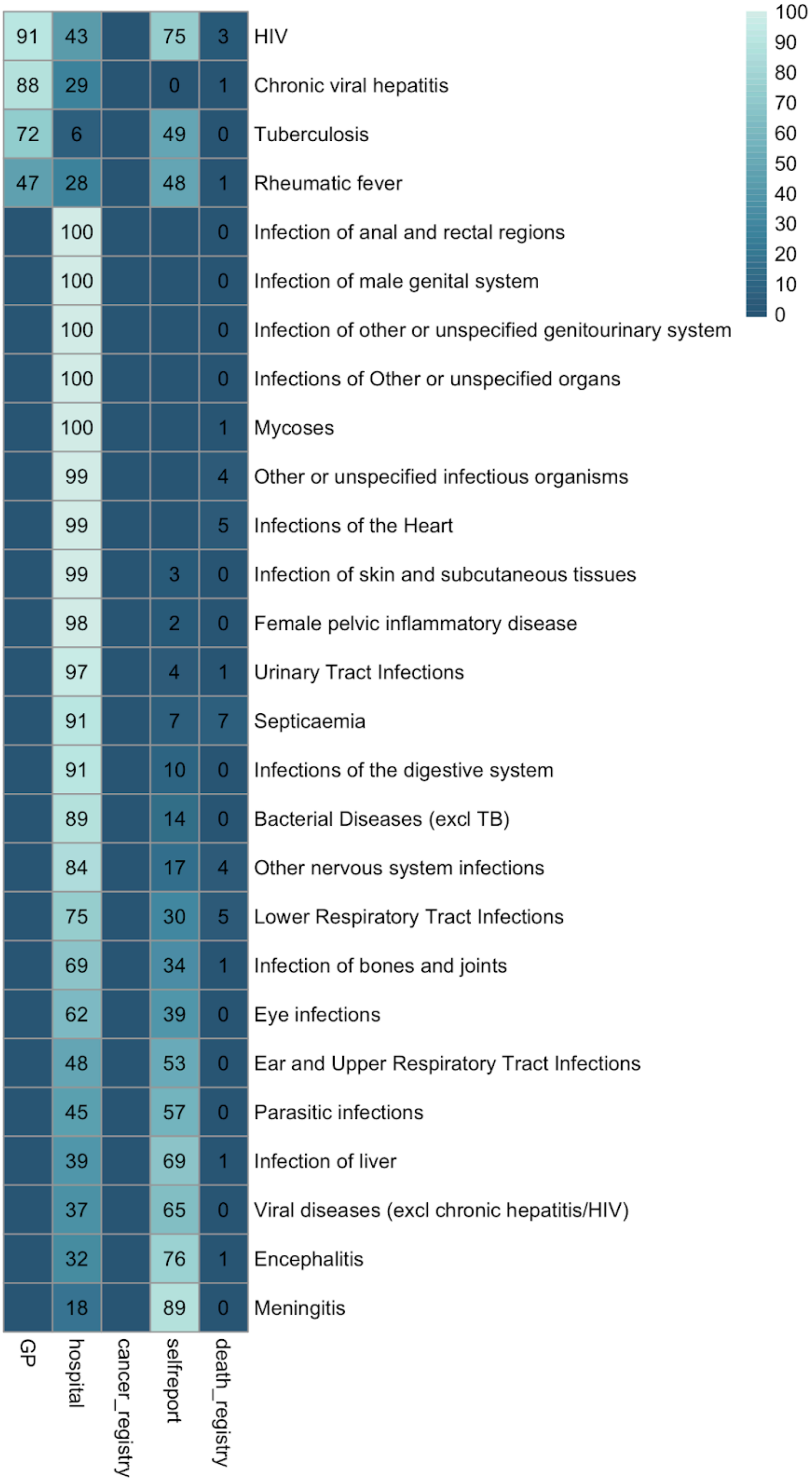

Figure S12. Proportion of patients per phenotype identified in each source in the Haematological group

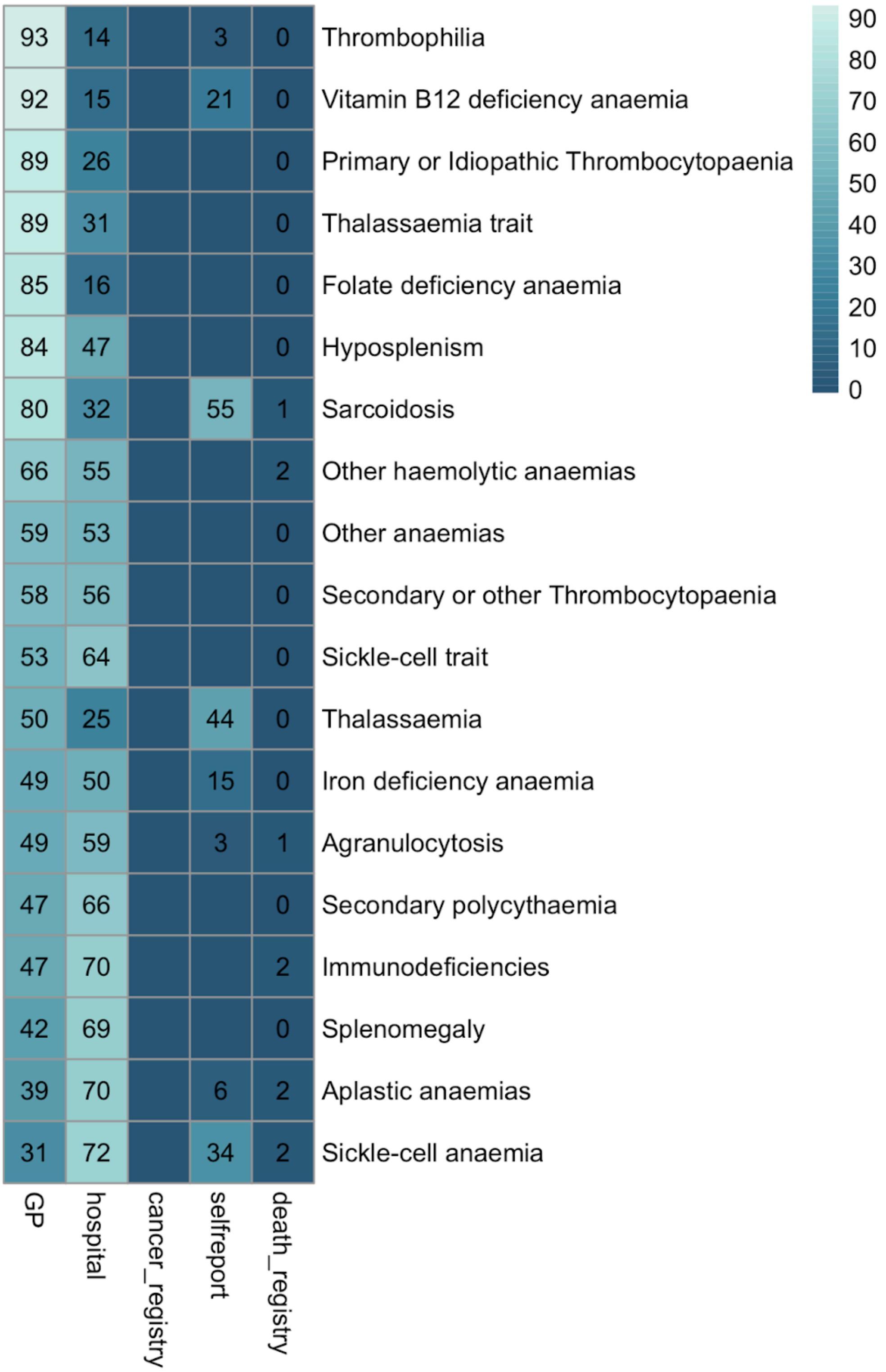

Figure S13. Proportion of patients per phenotype identified in each source in the Musculoskeletal group 35

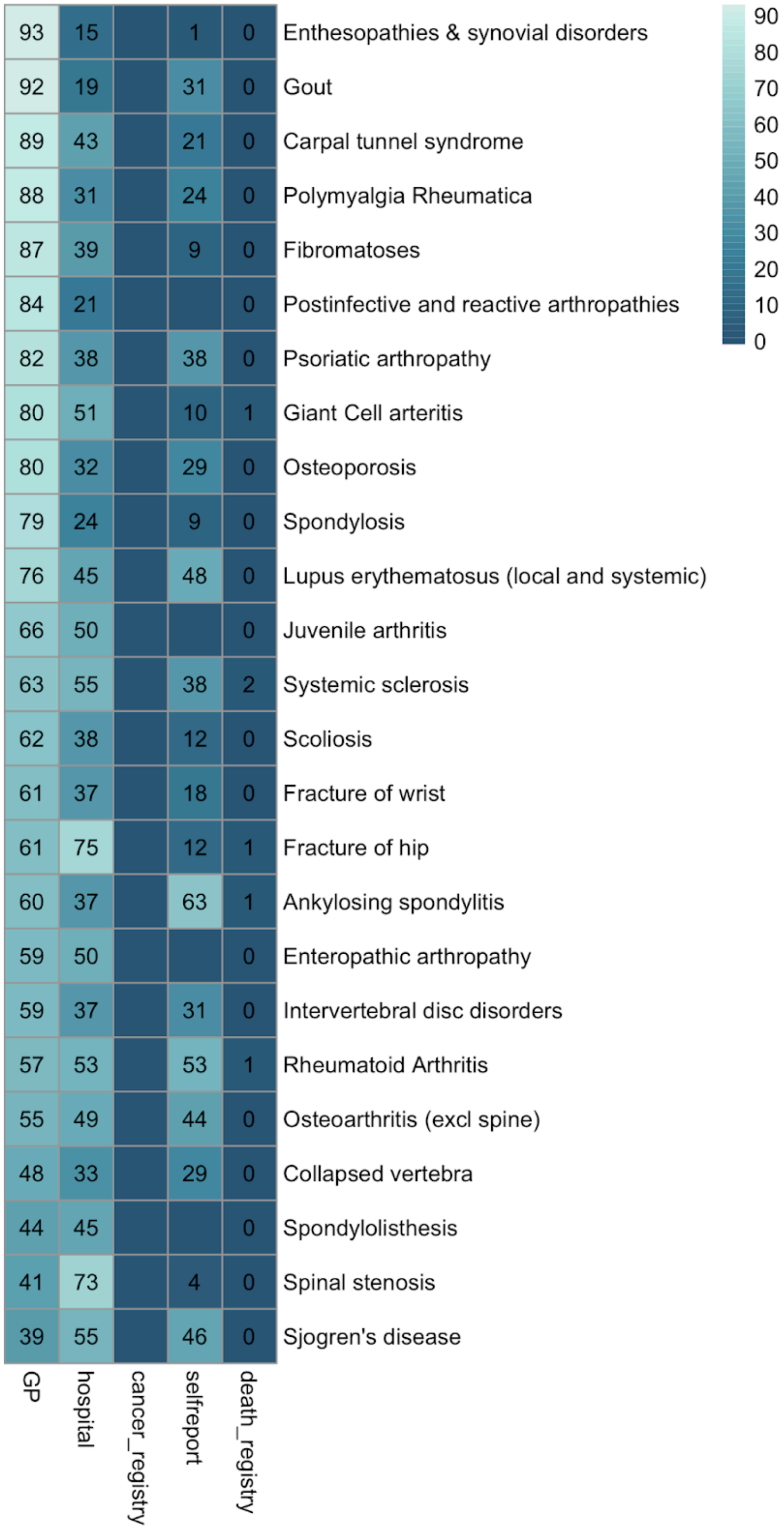

Figure S14. Proportion of patients per phenotype identified in each source in the Neurological group

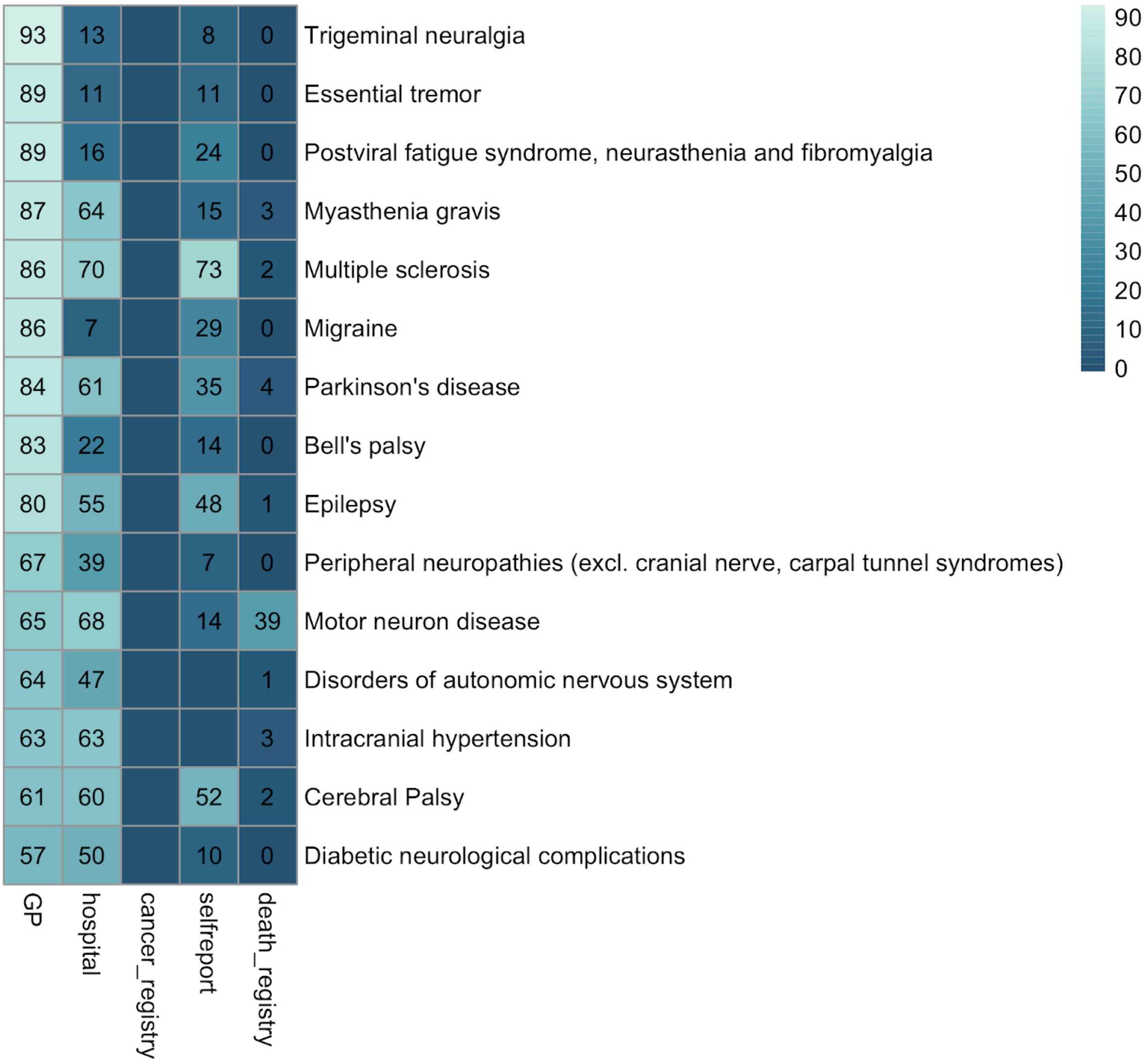

Figure S15. Proportion of patients per phenotype identified in each source in the Psychiatric group

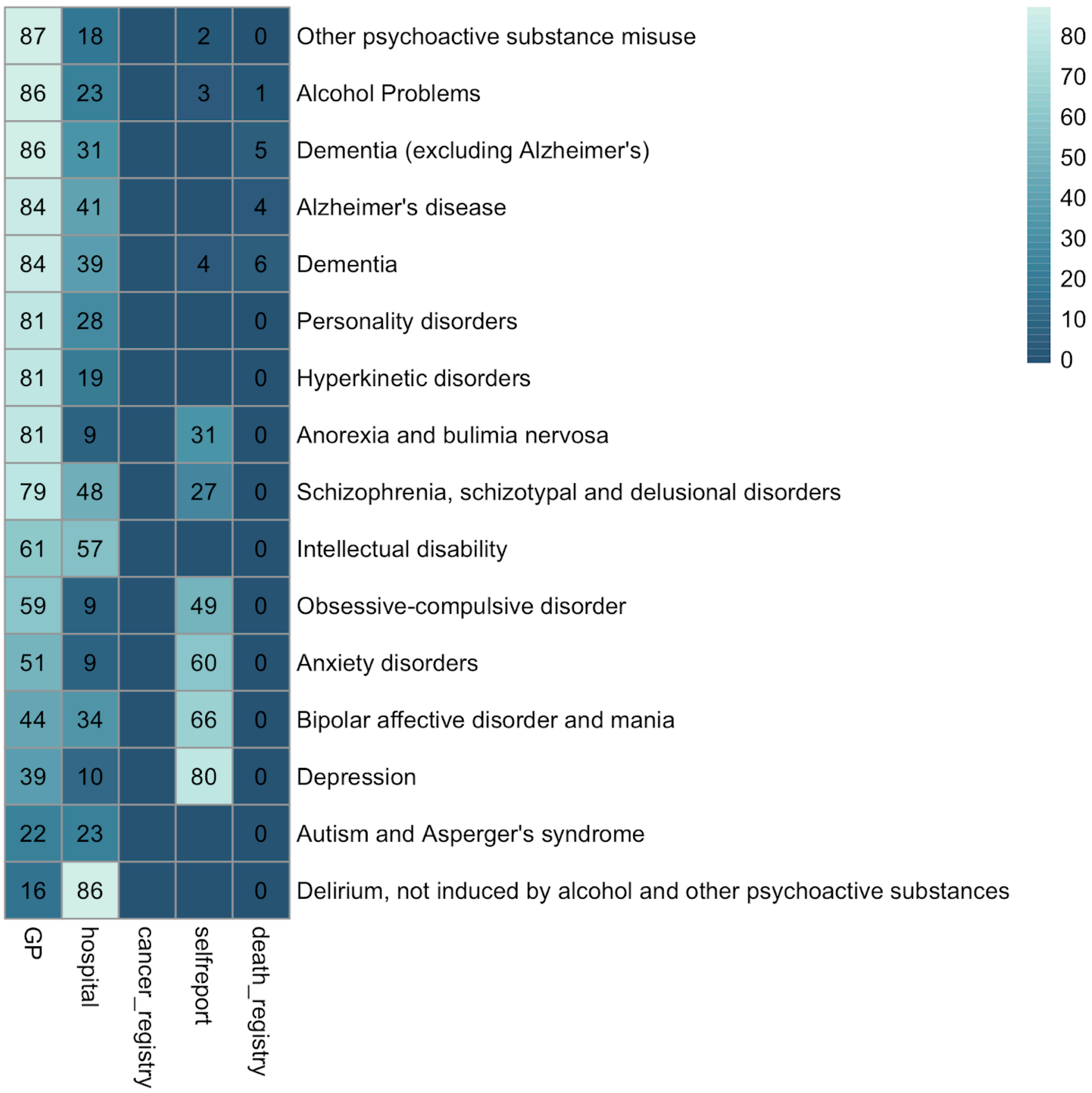

Figure S16. Proportion of patients per phenotype identified in each source in the Respiratory

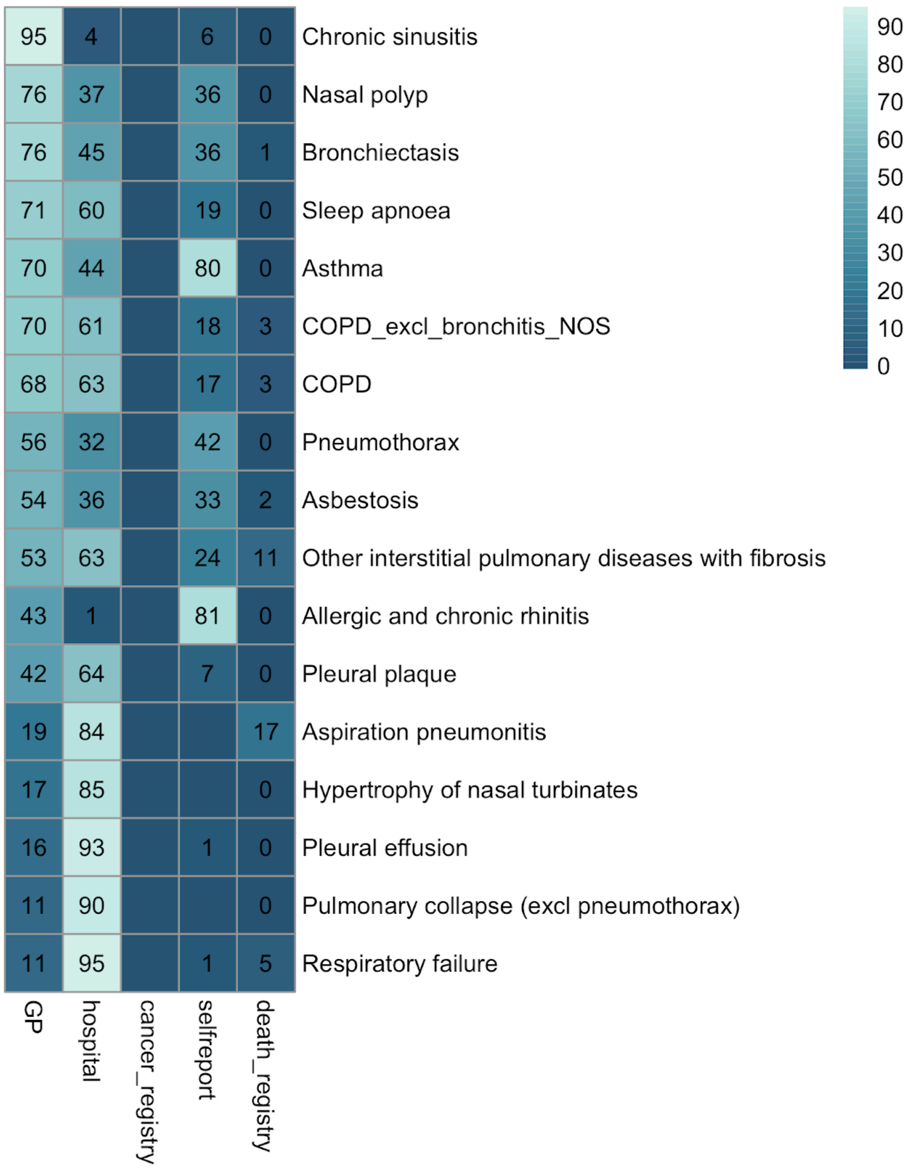

Figure S17. Proportion of patients per phenotype identified in each source in the Skin groups

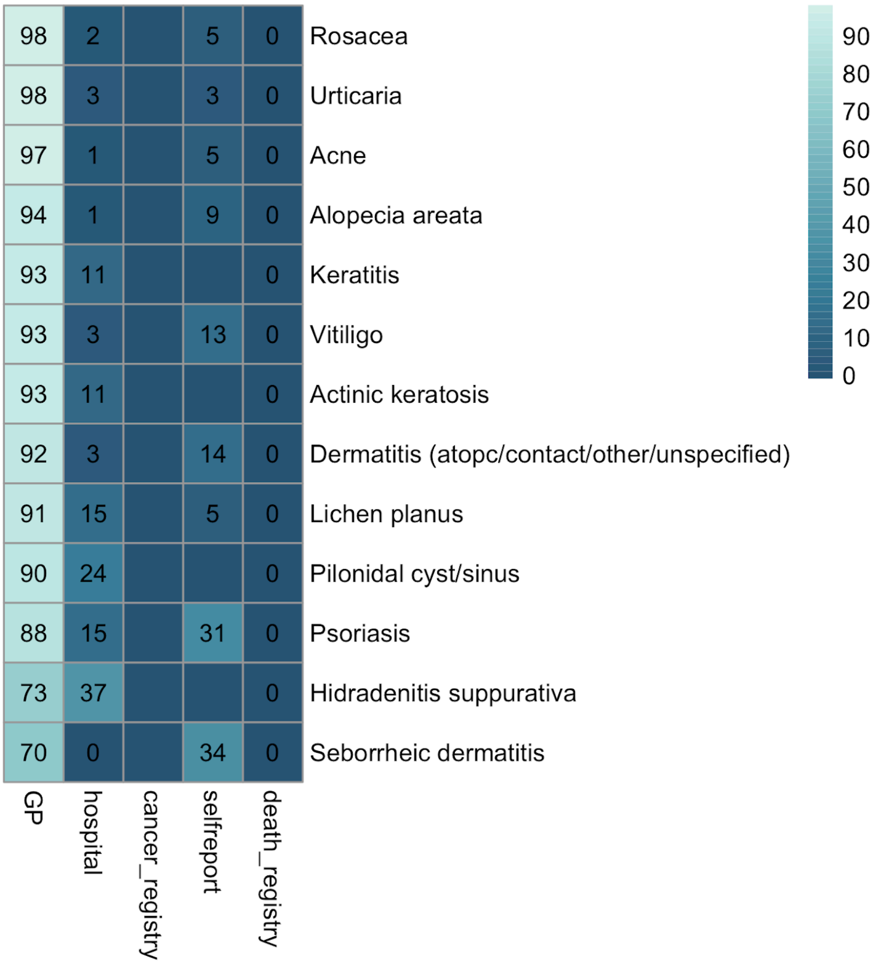

Figure S18. Baseline prevalence, incidence by age and sex for phenotypes in the Cardiovascular group

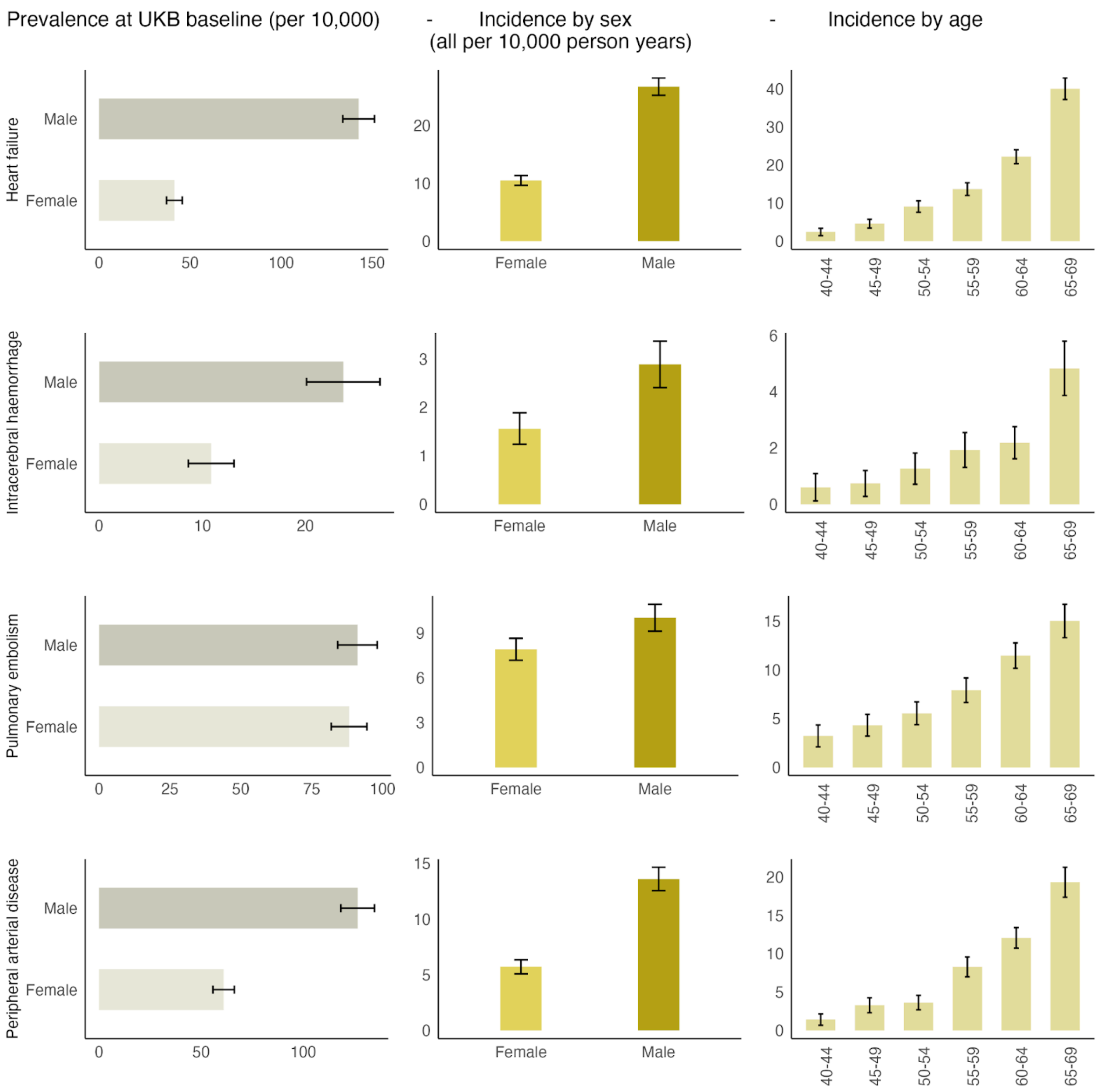

Figure S19. Baseline prevalence, incidence by age and sex for phenotypes in the Skin group

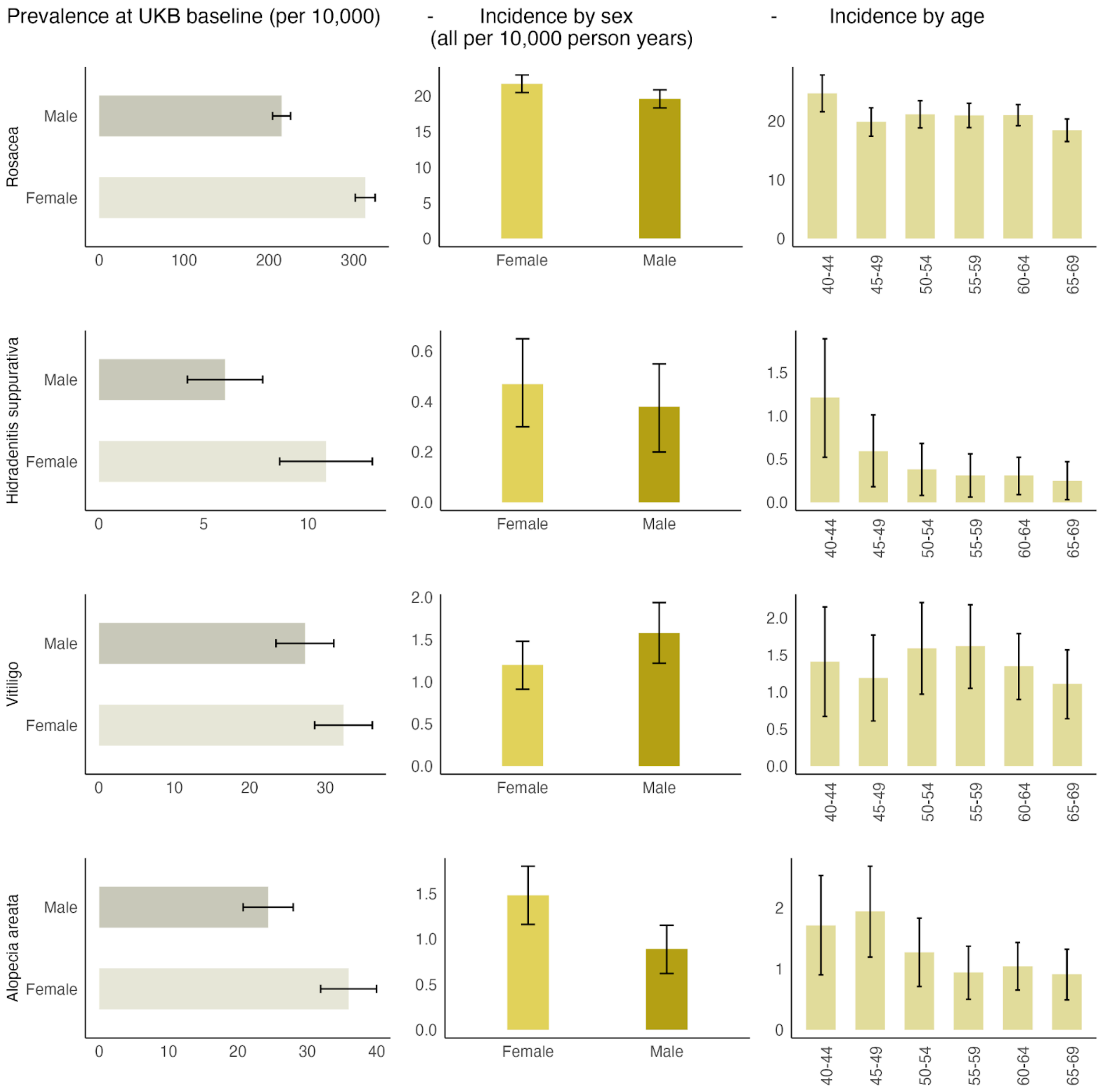

Figure S20. Baseline prevalence, incidence by age and sex for phenotypes in the Endocrine group

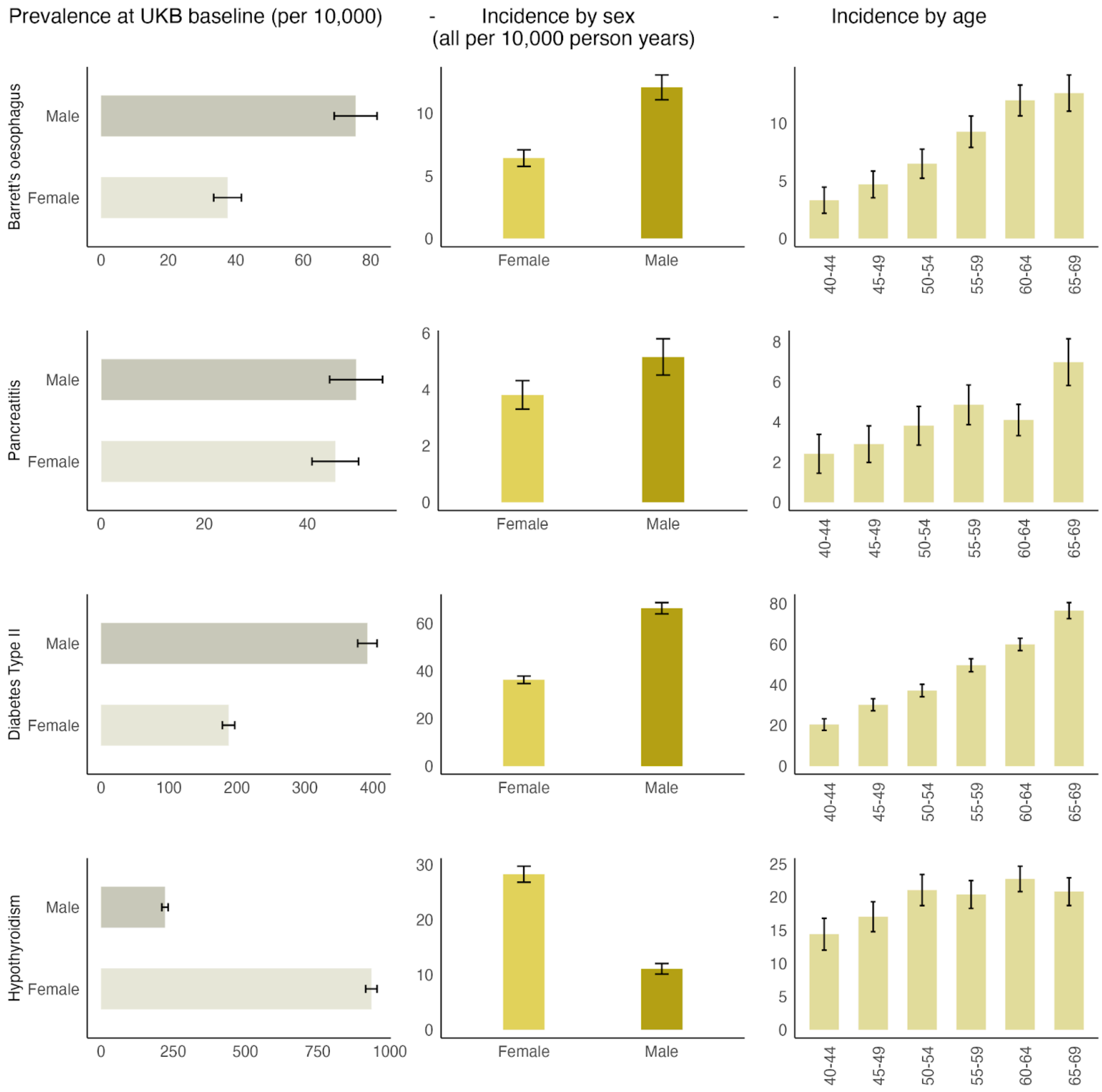

Figure S21. Log 10 -transformed sex-standardised period prevalence for UKB EHR participants from England recruitment centres and CALIBER (A), prevalence variation by country (England, Wales or Scotland) and prevalence by socioeconomic status (least and most deprived quintiles of Townsend scores at UK B entry) in 2_selected diseases for age bands 40-49, 50-59, 60-69.

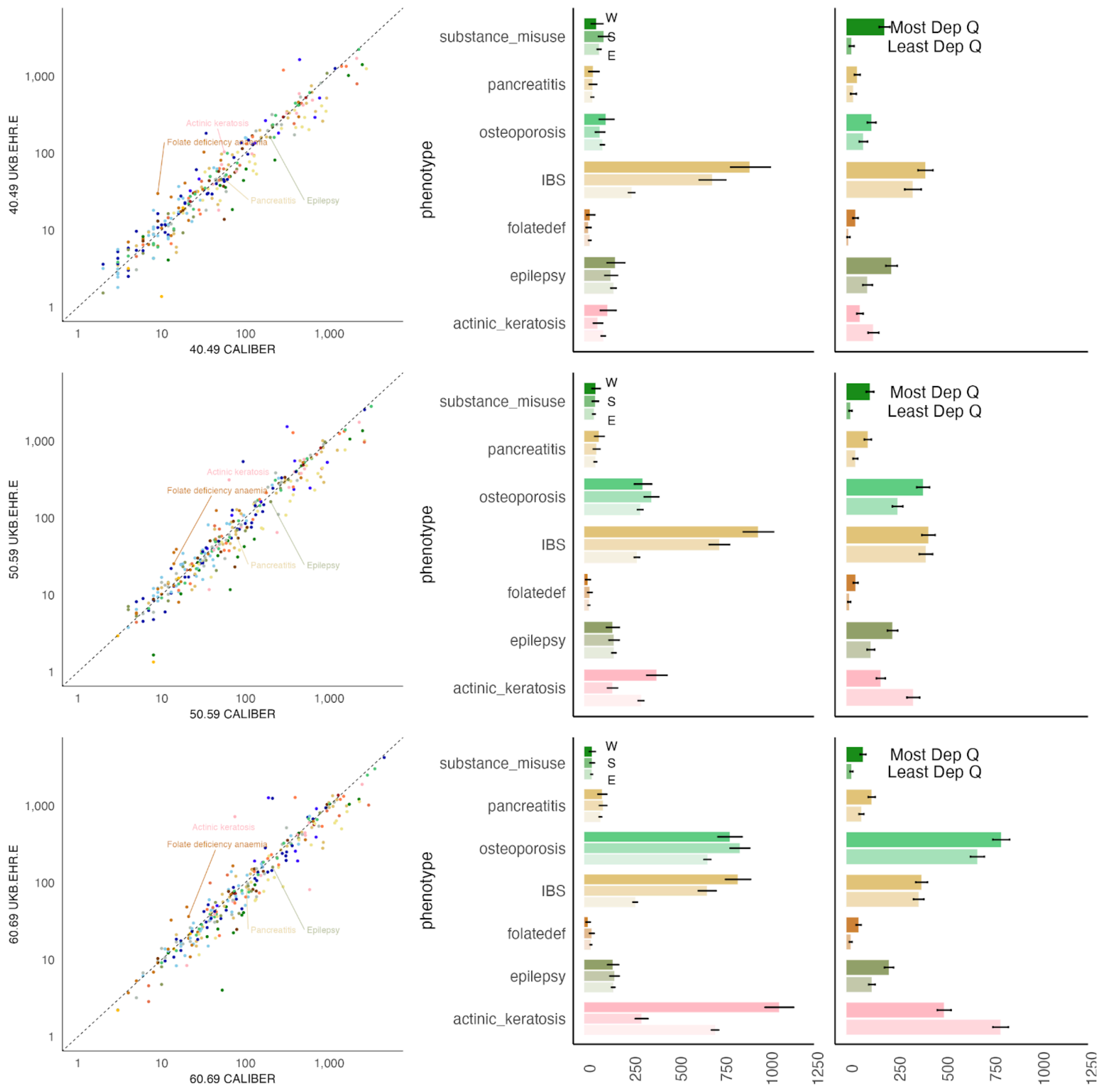

Supplementary Tables:

Table S1. Linked EHR sources and the end of their coverage. Primary care EHR censoring dates for England data providers England Vision and England TPP respectively. Dates are specified as DD-MM-YYYY.

	England	Scotland	Wales
Primary care	31-05-2017 / 31-05-2016	31-03-2017	31-08-2017
Death Registry	31-08-2020	31-08-2020	31-08-2020
HES	30-06-2020	31-08-2016	28-02-2016
Cancer Registry	31-03-2016	31-10-2015	31-12-2016

Table S2. Coding systems and data providers in primary care

	England	Scotland	Wales
GP data provider	Vision / TPP	EMIS / Vision	EMIS / Vision
Coding system	Read v2 / CTV3	Read v2	Read v2

Table S3. Codelists of the phenotypes and UKB Fields for ovarian cancer.

	**UK Field**	**Codes**	Description
**GP**	42040	B440.00	Malignant neoplasm of ovary
		B440.11	Cancer of ovary
		BB5j200	Endometrioid carcinoma
		BB5j500	Endometrioid adenofibroma, malignant
		BBQA100	Struma ovarii, malignant
		D212000	Anaemia in ovarian carcinoma
		XaBDS	Anaemia in ovarian carcinoma
**Hospital**	41202	C56	Malignant neoplasm of ovary
	41204	C56	Malignant neoplasm of ovary
**Death**	40001	C56	Malignant neoplasm of ovary
	40002	C56	Malignant neoplasm of ovary
**Cancer**	40006	C56	Malignant neoplasm of ovary
**Self-reported**	20002	1039 (value ovarian cancer)	

Table S4. Proportion of cases per source and median age at first record from patients with full linked data in all EHR sources from their events up to 31 March 2016 (N = 226,353). Patients could be represented in multiple sources. - indicates that the phenotypes did not include codes of that source – indicates N < 5

group	phenotype	phenotype_descr	neid_total	GP	hospital	selfreport	cancer_registry	death_registry	Median Age (all)	Median Age (female)	Median Age (male)
Benign neoplasm or Carcinoma in situ	cin_cervical	Carcinoma in situ_cervical	30766	94	3	1	6	0	49	49	n/a
Benign neoplasm or Carcinoma in situ	benign_colon	Benign neoplasm of colon, rectum, anus and anal canal	17037	41	89	4	0	0	62	61	62
Benign neoplasm or Carcinoma in situ	leiomyoma	Leiomyoma of uterus	13859	54	44	27	0	0	48	48	n/a
Benign neoplasm or Carcinoma in situ	benign_uterus	Benign neoplasm and polyp of uterus	6281	34	77	11	0	0	54	54	n/a
Benign neoplasm or Carcinoma in situ	benign_ovary	Benign neoplasm of ovary	5983	58	56	-	0	0	47	47	n/a
Benign neoplasm or Carcinoma in situ	haemangioma	Haemangioma, any site	4111	84	22	-	0	0	54	54	55
Benign neoplasm or Carcinoma in situ	benign_stomach	Benign neoplasm of stomach and duodenum	3451	26	93	-	0	0	63	63	63
Benign neoplasm or Carcinoma in situ	benign_brain	Benign neoplasm of brain and other parts of central nervous system	1285	61	54	18	37	0	55	54	56
Cancers	pri_skin	Primary Malignancy_Other Skin and subcutaneous tissue	13599	40	48	9	77	0	62	61	63
Cancers	pri_breast	Primary Malignancy_Breast	8523	68	79	64	89	4	55	55	62
Cancers	pri_prost	Primary Malignancy_Prostate	4661	74	73	39	93	5	65	n/a	65
Cancers	pri_melanoma	Primary Malignancy_Malignant Melanoma	3910	56	29	65	41	2	56	54	58

Cancers	sec_LN	Secondary Malignancy_Lymph Nodes	3404	2	99	-	2	1	62	61	65
Cancers	pri_bowel	Primary Malignancy_colorectal and anus	3276	47	82	38	85	12	62	61	62
Cancers	pri_other	Primary Malignancy_Other Organs	3042	29	55	13	31	18	61	61	62
Cancers	sec_liver	Secondary malignancy_Liver and intrahepatic bile duct	1546	29	96	-	2	4	66	65	67
Cancers	pri_lung	Primary Malignancy_Lung and trachea	1526	25	78	23	74	40	65	65	65
Cancers	pri_bladder	Primary Malignancy_Bladder	1453	52	84	40	39	5	63	60	63
Cancers	NHL	Non-Hodgkin Lymphoma	1439	65	74	32	75	11	61	61	61
Cancers	sec_other	Secondary Malignancy_Other organs	1391	40	59	-	1	12	65	63	67
Cancers	sec_bone	Secondary Malignancy_Bone	1325	20	96	1	1	3	66	65	68
Cancers	pri_uterine	Primary Malignancy_Uterine	1190	33	64	50	73	4	58	58	n/a
Cancers	sec_lung	Secondary Malignancy_Lung	1182	19	93	-	0	6	65	65	66
Cancers	pri_cervical	Primary Malignancy_Cervical	1163	14	13	84	21	1	37	37	n/a
Cancers	pri_ovarian	Primary Malignancy_Ovarian	1035	54	53	39	55	12	57	57	n/a
Cancers	pri_kidney	Primary Malignancy_Kidney and Ureter	884	43	78	39	79	13	62	61	62
Cancers	leukaemia	Leukaemia	851	76	69	13	67	12	63	63	63
Cancers	pri_oroph	Primary Malignancy_Oro-pharyngeal	844	39	61	45	61	6	58	57	58
Cancers	sec_peritoneum	Secondary Malignancy_retroperitoneum and peritoneum	811	8	98	-	3	4	65	65	66
Cancers	MGUS	Monoclonal gammopathy of undetermined significance (MGUS)	546	85	25	7	12	0	64	64	65

Cancers	sec_brain	Secondary Malignancy_Brain, Other CNS and Intracranial	507	38	92	-	0	10	65	65	67
Cancers	plasmacell	Multiple myeloma and malignant plasma cell neoplasms	481	69	84	19	74	15	65	65	65
Cancers	pri_brain	Primary Malignancy_Brain, Other CNS and Intracranial	475	39	64	25	56	37	59	57	61
Cancers	pri_testis	Primary Malignancy_Testicular	459	42	32	88	53	0	40	n/a	40
Cancers	pri_oesoph	Primary Malignancy_Oesophageal	449	49	86	21	69	38	66	64	66
Cancers	pri_stomach	Primary Malignancy_Stomach	430	22	76	23	59	22	64	62	65
Cancers	pri_thyroid	Primary Malignancy_Thyroid	413	41	54	58	72	2	53	52	56
Cancers	pri_pancr	Primary Malignancy_Pancreatic	403	55	86	9	78	59	66	66	66
Cancers	PCV	Polycythaemia vera	345	61	70	30	-	1	58	59	58
Cancers	sec_pleura	Secondary Malignancy_Pleura	341	1	99	-	2	2	66	65	67
Cancers	hodgkins	Hodgkin Lymphoma	330	74	48	65	50	4	41	37	43
Cancers	pri_liver	Primary Malignancy_Liver	234	38	52	35	39	15	64	63	65
Cancers	pri_biliary	Primary Malignancy_biliary tract	207	42	78	6	64	39	66	65	66
Cancers	MDS	Myelodysplastic syndromes	180	27	81	6	48	13	64	62	66
Cancers	pri_bone	Primary Malignancy_Bone and articular cartilage	174	38	42	34	16	4	54	52	56
Cancers	sec_adrenal	Secondary Malignancy_Adrenal gland	173	9	94	-	1	2	67	66	67
Cancers	sec_bowel	Secondary Malignancy_Bowel	140	1	94	-	3	4	66	66	67
Cancers	pri_mesothelioma	Primary Malignancy_Mesothelioma	127	25	90	6	90	65	69	69	69
Cancers	pri_LN	Primary Malignancy_Lymph Nodes	101	15	2	70	13	1	54	54	51

Cancers	pri_multindep	Primary Malignancy_Multiple independent sites	86	0	87	-	1	13	68	67	69
Cancers	pri_adrenal	Primary Malignancy_Adrenal gland	49	24	47	18	29	8	56	54	57
Cardiovascular	hypertension	Hypertension	74859	82	63	2	-	0	58	58	58
Cardiovascular	CHD_NOS	Coronary heart disease not otherwise specified	20217	66	75	4	-	4	61	62	61
Cardiovascular	stable_angina	Stable angina	14786	74	67	-	-	0	60	60	60
Cardiovascular	myocardial_infarction	Myocardial infarction	11507	48	86	51	-	3	58	59	57
Cardiovascular	AF	Atrial fibrillation	11265	74	79	19	-	1	64	65	64
Cardiovascular	vte_ex_pe	Venous thromboembolic disease (Excl PE)	7133	52	28	67	-	1	51	46	55
Cardiovascular	hf	Heart failure	5188	57	69	3	-	6	65	66	65
Cardiovascular	Stroke_NOS	Stroke NOS	5082	83	31	-	-	2	62	62	62
Cardiovascular	TIA	Transient ischaemic attack	4580	60	44	20	-	0	62	62	61
Cardiovascular	unstable_angina	Unstable Angina	3991	43	77	-	-	0	60	61	60
Cardiovascular	raynauds	Raynaud's syndrome	3829	88	18	10	-	0	53	51	57
Cardiovascular	PE	Pulmonary embolism	3768	29	59	54	-	6	57	55	59
Cardiovascular	peripheral_arterial_disease	Peripheral arterial disease	3757	60	53	23	-	1	61	60	61
Cardiovascular	Isch_stroke	Ischaemic stroke	2713	28	84	0	-	2	64	65	64
Cardiovascular	nonRh_aortic	Nonrheumatic aortic valve disorders	2413	69	66	-	-	2	64	65	64
Cardiovascular	SVT	Supraventricular tachycardia	2323	58	60	8	-	0	57	56	59
Cardiovascular	nonRh_mitral	Nonrheumatic mitral valve disorders	2104	48	66	3	-	0	61	60	62
Cardiovascular	RBBB	Right bundle branch block	1510	47	60	-	-	0	64	64	64
Cardiovascular	LBBB	Left bundle branch block	1299	30	82	-	-	0	66	65	66

Cardiovascular	mult_valve	Multiple valve dz	1102	13	89	-	-	0	67	66	67
Cardiovascular	Subarach	Subarachnoid haemorrhage	913	71	59	25	-	6	54	55	52
Cardiovascular	av_block_1	Atrioventricular block, first degree	879	31	72	-	-	0	66	66	67
Cardiovascular	cardiomyo_oth	Other Cardiomyopathy	874	63	55	-	-	4	62	62	62
Cardiovascular	AAA	Abdominal aortic aneurysm	845	18	81	-	-	1	66	65	66
Cardiovascular	pericardial_effusion	Pericardial effusion (noninflammatory)	820	62	49	1	-	0	58	61	56
Cardiovascular	Intracereb_haem	Intracerebral haemorrhage	765	36	68	13	-	10	62	62	62
Cardiovascular	VT	Ventricular tachycardia	612	24	88	-	-	0	63	59	63
Cardiovascular	Rh_valve	Rheumatic valve dz	590	39	69	4	-	2	60	57	63
Cardiovascular	dcm	Dilated cardiomyopathy	502	36	84	-	-	6	61	61	61
Cardiovascular	av_block_3	Atrioventricular block, complete	390	11	97	-	-	1	66	65	66
Cardiovascular	sick_sinus	Sick sinus syndrome	363	36	79	4	-	0	65	65	65
Cardiovascular	av_block_2	Atrioventricular block, second degree	334	27	84	-	-	0	65	64	65
Cardiovascular	prim_pulm_htn	Primary pulmonary hypertension	323	24	86	-	-	0	64	63	65
Cardiovascular	subdural_haem	Subdural haematoma - nontraumatic	280	15	66	31	-	3	62	61	64
Cardiovascular	sec_pulm_htn	Secondary pulmonary hypertension	266	16	88	-	-	5	68	68	68
Cardiovascular	hocm	Hypertrophic Cardiomyopathy	244	53	62	20	-	1	59	63	57
Cardiovascular	trifasc_block	Trifascicular block	61	30	79	-	-	0	68	68	69
Cardiovascular	bifasc_block	Bifascicular block	57	9	91	-	-	0	69	70	68
Digestive	GORD	Gastro-oesophageal reflux disease	31120	34	50	40	-	0	57	57	56

Digestive	gastritis_duodenitis	Gastritis and duodenitis	27274	55	69	2	-	0	57	57	57
Digestive	hernia_abdo	Abdominal Hernia	22712	80	60	13	-	0	55	53	55
Digestive	diverticuli	Diverticular disease of intestine (acute and chronic)	20721	49	82	13	-	0	62	62	62
Digestive	hernia_diaphragm	Diaphragmatic hernia	19689	28	82	27	-	0	58	58	58
Digestive	oesoph_ulc	Oesophagitis and oesophageal ulcer	19142	59	68	-	-	0	57	57	56
Digestive	cholelithiasis	Cholelithiasis	13790	34	71	31	-	0	56	55	59
Digestive	IBS	Irritable bowel syndrome	13216	50	25	44	-	0	48	48	48
Digestive	appendicitis	Appendicitis	11468	69	20	28	-	0	22	23	22
Digestive	ulcer_peptic	Peptic ulcer disease	9897	54	47	31	-	0	50	52	47
Digestive	anal_fissure	Anal fissure	6777	89	20	2	-	0	49	48	50
Digestive	cholecystitis	Cholecystitis	6443	31	83	4	-	0	58	56	60
Digestive	IBD	Inflammatory bowel disease (IBD)	3973	70	69	50	-	0	46	44	47
Digestive	ulc_colitis	Ulcerative colitis	3007	77	64	42	-	0	46	45	48
Digestive	fatty_liver	Fatty Liver	2905	66	42	-	-	1	61	61	60
Digestive	barretts	Barrett's oesophagus	2800	76	61	27	-	0	61	62	61
Digestive	coeliac	Coeliac disease	2723	57	40	78	-	0	54	53	55
Digestive	peritonitis	Peritonitis	2471	28	52	32	-	3	52	52	52
Digestive	pancreatitis	Pancreatitis	1948	61	71	27	-	2	57	56	57
Digestive	anorectal_fistula	Anorectal fistula	1695	68	54	-	-	0	49	48	50
Digestive	crohns	Crohn's disease	1360	44	77	55	-	1	47	46	48
Digestive	cirrhosis	Liver fibrosis, sclerosis and cirrhosis	1336	64	47	13	-	4	58	59	58
Digestive	anorectal_prolapse	Anorectal prolapse	1226	52	55	13	-	0	59	59	58

Digestive	liver_alc	Alcoholic liver disease	833	61	62	4	-	10	57	55	57
Digestive	cholangitis	Cholangitis	541	43	77	4	-	5	63	61	65
Digestive	varices	Oesophageal varices	523	50	71	7	-	2	59	57	60
Digestive	liver_fail	Hepatic failure	459	6	65	31	-	12	59	58	60
Digestive	volvulus	Volvulus	445	27	84	-	-	1	61	60	63
Digestive	autoimm_liver	Autoimmune liver disease	407	86	53	14	-	2	57	58	55
Digestive	portal_htn	Portal hypertension	348	20	96	-	-	1	62	62	62
Digestive	angiodysplasia_colon	Angiodysplasia of colon	333	28	85	-	-	0	66	66	66
Ear	deaf	Hearing loss	29183	82	8	-	-	0	60	59	60
Ear	tinnitus	Tinnitus	28230	40	1	72	-	0	58	58	59
Ear	meniere	Meniere disease	1845	77	18	37	-	0	52	52	53
Endocrine	obesity	Obesity	25195	83	28	-	-	0	56	56	57
Endocrine	thyroid	Hypo or hyperthyroidism	20293	75	47	67	-	0	52	51	56
Endocrine	hypothyroid	Hypothyroidism	17542	68	50	68	-	0	53	52	57
Endocrine	diabetes_t2	Diabetes Type II	15310	39	79	13	-	1	61	61	61
Endocrine	diabetes_nos	Diabetes NOS	11145	-	19	94	-	2	55	55	55
Endocrine	thyroid_nos	Thyroiditis unspecified	4621	94	4	4	-	0	52	51	54
Endocrine	hyperthyroid	Hyperthyroidism	4561	72	25	44	-	0	49	48	52
Endocrine	diabetes_t1	Diabetes Type I	2010	20	89	11	-	1	56	55	57
Endocrine	PTH	Hyperparathyroidism	1024	76	51	8	-	0	62	62	61
Endocrine	PCOS	Polycystic ovarian syndrome	730	69	17	40	-	0	33	33	n/a
Endocrine	CF	Cystic Fibrosis	120	89	8	-	-	1	45	44	61
Endocrine	SIADH	Syndrome of inappropriate secretion of antidiuretic hormone	103	-	100	-	-	0	67	66	67
Eye	cataract	Cataract	19160	58	72	81	-	0	65	65	64

Eye	diab_eye	Diabetic ophthalmic complications	8412	90	12	23	-	0	62	62	62
Eye	glaucoma	Glaucoma	7305	71	33	55	-	0	60	61	60
Eye	macula_degen	Macular degeneration	4369	30	33	52	-	0	65	65	64
Eye	retinal_detach	Retinal detachments and breaks	2969	63	45	40	-	0	58	58	58
Eye	ant_uveitis	Anterior and Intermediate Uveitis	2014	91	12	7	-	0	50	51	50
Eye	blind	Visual impairment and blindness	1579	74	38	-	-	0	58	57	58
Eye	ptosis	Ptosis of eyelid	1464	69	58	-	-	0	60	60	61
Eye	retinal_vasc_occl	Retinal vascular occlusions	1326	84	23	9	-	0	62	62	61
Eye	scleritis	Scleritis and episcleritis	709	98	4	-	-	0	52	52	51
Eye	post_uveitis	Posterior Uveitis	233	93	13	-	-	0	49	51	46
Genitourinary	menorrhagia	Menorrhagia and polymenorrhoea	20533	78	33	5	-	0	45	45	n/a
Genitourinary	ED	Erectile dysfunction	18519	99	1	1	-	0	60	n/a	60
Genitourinary	urine_incont	Urinary Incontinence	14178	80	28	10	-	0	55	54	62
Genitourinary	female_genital_prolapse	Female genital prolapse	12710	75	53	11	-	0	56	56	n/a
Genitourinary	BPH	Hyperplasia of prostate	12586	61	53	5	-	0	62	n/a	62
Genitourinary	PMB	Postmenopausal bleeding	11598	76	44	-	-	0	56	56	n/a
Genitourinary	urolithiasis	Urolithiasis	7786	79	44	25	-	0	50	51	50
Genitourinary	CKD	Chronic Kidney Disease	7155	56	50	9	-	1	65	65	66
Genitourinary	endometriosis	Endometriosis	5302	50	44	39	-	0	41	41	n/a
Genitourinary	dysmenorrhoea	Dysmenorrhoea	4809	80	20	5	-	0	41	41	n/a
Genitourinary	neuro_bladder	Neuromuscular dysfunction of bladder	4362	83	25	-	-	0	57	56	59

Genitourinary	AKI	Acute Kidney Injury	3355	-	100	-	-	2	67	67	68
Genitourinary	PCB	Postcoital and contact bleeding	3147	84	21	-	-	0	46	46	n/a
Genitourinary	female_infertility	Female infertility	2884	76	21	12	-	0	33	33	n/a
Genitourinary	obstr_reflux	Obstructive and reflux uropathy	2163	41	74	-	-	0	60	57	61
Genitourinary	hydrocele	Hydrocoele (incl infected)	2037	87	38	-	-	0	55	n/a	55
Genitourinary	GN	Glomerulonephritis	1586	43	65	8	-	2	56	55	58
Genitourinary	endometrial_hyper	Endometrial hyperplasia and hypertrophy	1556	30	74	-	-	0	51	51	n/a
Genitourinary	ESRD	End stage renal disease	919	65	55	50	-	2	59	59	59
Genitourinary	chr_cystitis	Non-acute cystitis	786	43	66	-	-	0	59	57	64
Genitourinary	TIN	Tubulo-interstitial nephritis	686	3	88	10	-	0	57	56	59
Genitourinary	male_infertility	Male infertility	660	94	5	3	-	0	36	n/a	36
Genitourinary	undescended_testis	Undescended testicle	396	72	10	26	-	0	13	n/a	13
Haematological or immunological	oth_anaemia	Other anaemias	14870	59	53	-	-	0	55	50	63
Haematological or immunological	IDA	Iron deficiency anaemia	10619	49	50	15	-	0	52	49	62
Haematological or immunological	b12_def	Vitamin B12 deficiency anaemia	3827	92	15	21	-	0	58	55	62
Haematological or immunological	agranulocytosis	Agranulocytosis	3315	49	59	3	-	1	59	58	62
Haematological or immunological	sec_oth_thrombocytopaenia	Secondary or other Thrombocytopaenia	1475	58	56	-	-	0	61	58	62
Haematological or immunological	sarcoid	Sarcoidosis	1016	80	32	55	-	1	40	42	40
Haematological or immunological	pri_thrombocytopaenia	Primary or Idiopathic Thrombocytopaenia	1009	89	26	-	-	0	57	53	60

Haematological or immunological	folatedef	Folate deficiency anaemia	832	85	16	-	-	0	61	58	63
Haematological or immunological	thrombophilia	Thrombophilia	817	93	14	3	-	0	54	53	56
Haematological or immunological	hyposplenism	Hyposplenism	539	84	47	-	-	0	46	48	45
Haematological or immunological	splenomegaly	Splenomegaly	378	42	69	-	-	0	60	58	61
Haematological or immunological	aplastic	Aplastic anaemias	374	39	70	6	-	2	61	57	64
Haematological or immunological	oth_haem_anaemia	Other haemolytic anaemias	279	66	55	-	-	2	53	51	54
Haematological or immunological	thal_trait	Thalassaemia trait	275	89	31	-	-	0	46	43	50
Haematological or immunological	2ry_polycythaemia	Secondary polycythaemia	253	47	66	-	-	0	59	59	59
Haematological or immunological	sickle_trait	Sickle-cell trait	205	53	64	-	-	0	48	48	48
Haematological or immunological	immunodef	Immunodeficiencies	176	47	70	-	-	2	60	58	61
Haematological or immunological	thala	Thalassaemia	173	50	25	44	-	0	43	42	47
Haematological or immunological	sickle_cell	Sickle-cell anaemia	64	31	72	34	-	2	47	43	47
Infections	bacterial	Bacterial Diseases (excl TB)	27957	-	89	14	-	0	56	55	58
Infections	oth_organisms	Other or unspecified infectious organisms	20611	-	99	-	-	4	62	61	63
Infections	lrti	Lower Respiratory Tract Infections	14677	-	75	30	-	5	59	58	60
Infections	oth_organs	Infections of Other or unspecified organs	11204	-	100	-	-	0	60	59	61

Infections	viral	Viral diseases (excl chronic hepatitis/HIV)	10693	-	37	65	-	0	12	15	11
Infections	uti	Urinary Tract Infections	9394	-	97	4	-	1	61	59	63
Infections	digestive	Infections of the digestive system	7897	-	91	10	-	0	62	62	62
Infections	ear_urti	Ear and Upper Respiratory Tract Infections	7556	-	48	53	-	0	35	32	38
Infections	skin	Infection of skin and subcutaneous tissues	7470	-	99	3	-	0	58	58	58
Infections	sepsis	Septicaemia	3002	-	91	7	-	7	64	63	65
Infections	PID	Female pelvic inflammatory disease	2582	-	98	2	-	0	48	48	n/a
Infections	TB	Tuberculosis	2479	72	6	49	-	0	17	16	18
Infections	mycoses	Mycoses	1799	-	100	-	-	1	64	63	64
Infections	rh_fever	Rheumatic fever	1552	47	28	48	-	1	15	14	16
Infections	liver	Infection of liver	1226	-	39	69	-	1	36	33	38
Infections	meningitis	Meningitis	1052	-	18	89	-	0	21	24	17
Infections	bone	Infection of bones and joints	1029	-	69	34	-	1	54	52	54
Infections	anorectal	Infection of anal and rectal regions	941	-	100	-	-	0	52	50	53
Infections	male_GU	Infection of male genital system	802	-	100	-	-	0	58	n/a	58
Infections	chr_hep	Chronic viral hepatitis	773	88	29	0	-	1	47	45	47
Infections	eye	Eye infections	603	-	62	39	-	0	56	57	56
Infections	parasitic	Parasitic infections	571	-	45	57	-	0	39	42	37
Infections	oth_gu	Infection of other or unspecified genitourinary system	482	-	100	-	-	0	50	50	58
Infections	oth_nerv_sys	Other nervous system infections	425	-	84	17	-	4	58	57	59

Infections	hiv	HIV	236	91	43	75	-	3	44	40	44
Infections	enceph	Encephalitis	194	-	32	76	-	1	42	38	44
Infections	heart	Infections of the Heart	172	-	99	-	-	5	60	58	61
Musculoskeletal	enthesopathy	Enthesopathies & synovial disorders	64813	93	15	1	-	0	54	54	54
Musculoskeletal	OA	Osteoarthritis (excl spine)	55149	55	49	44	-	0	57	57	57
Musculoskeletal	spondylosis	Spondylosis	19187	79	24	9	-	0	54	54	55
Musculoskeletal	intervert_disc	Intervertebral disc disorders	15915	59	37	31	-	0	50	51	49
Musculoskeletal	carpal_tunnel	Carpal tunnel syndrome	14490	89	43	21	-	0	54	53	56
Musculoskeletal	osteoporosis	Osteoporosis	13330	80	32	29	-	0	61	61	61
Musculoskeletal	gout	Gout	11422	92	19	31	-	0	57	62	56
Musculoskeletal	fracture_wrist	Fracture of wrist	8023	61	37	18	-	0	54	58	41
Musculoskeletal	fibromatosis	Fibromatoses	5650	87	39	9	-	0	61	61	60
Musculoskeletal	RhA	Rheumatoid Arthritis	5232	57	53	53	-	1	54	54	56
Musculoskeletal	spinal_stenosis	Spinal stenosis	3227	41	73	4	-	0	63	64	63
Musculoskeletal	PMR	Polymyalgia Rheumatica	2289	88	31	24	-	0	65	64	66
Musculoskeletal	scoliosis	Scoliosis	1778	62	38	12	-	0	50	53	46
Musculoskeletal	spondylolisthesis	Spondylolisthesis	1762	44	45	-	-	0	58	59	54
Musculoskeletal	fracture_hip	Fracture of hip	1608	61	75	12	-	1	62	65	58
Musculoskeletal	PSA	Psoriatic arthropathy	1129	82	38	38	-	0	50	51	49
Musculoskeletal	ank_spond	Ankylosing spondylitis	1078	60	37	63	-	1	41	46	38
Musculoskeletal	collapsed_vert	Collapsed vertebra	878	48	33	29	-	0	61	63	60
Musculoskeletal	SLE	Lupus erythematosus (local and systemic)	678	76	45	48	-	0	48	48	50
Musculoskeletal	sjogren	Sjogren's disease	578	39	55	46	-	0	56	56	56
Musculoskeletal	GCA	Giant Cell arteritis	493	80	51	10	-	1	65	64	66

Musculoskeletal	reactive	Postinfective and reactive arthropathies	277	84	21	-	-	0	48	52	40
Musculoskeletal	sys_sclerosis	Systemic sclerosis	215	63	55	38	-	2	56	56	54
Musculoskeletal	juv_arth	Juvenile arthritis	58	66	50	-	-	0	36	35	40
Musculoskeletal	entero_arthro	Enteropathic arthropathy	34	59	50	-	-	0	51	50	59
Neurological	migraine	Migraine	24481	86	7	29	-	0	42	41	43
Neurological	periph_neuro	Peripheral neuropathies (excl. cranial nerve, carpal tunnel syndromes)	8148	67	39	7	-	0	57	56	58
Neurological	chronic_fatigue	Postviral fatigue syndrome, neurasthenia and fibromyalgia	6629	89	16	24	-	0	50	50	49
Neurological	epilepsy	Epilepsy	3930	80	55	48	-	1	42	40	44
Neurological	trigem_neur	Trigeminal neuralgia	1925	93	13	8	-	0	57	55	59
Neurological	bells	Bell's palsy	1886	83	22	14	-	0	51	50	52
Neurological	Parkinsons	Parkinson's disease	1221	84	61	35	-	4	65	64	65
Neurological	MS	Multiple sclerosis	1177	86	70	73	-	2	43	43	43
Neurological	essential_tremor	Essential tremor	1010	89	11	11	-	0	63	63	63
Neurological	dm_neuro	Diabetic neurological complications	774	57	50	10	-	0	61	61	60
Neurological	autonomic_neuro	Disorders of autonomic nervous system	691	64	47	-	-	1	57	56	58
Neurological	MND	Motor neuron disease	190	65	68	14	-	39	64	64	64
Neurological	myasthenia	Myasthenia gravis	178	87	64	15	-	3	57	53	62
Neurological	cerebral_palsy	Cerebral Palsy	174	61	60	52	-	2	1	1	1
Neurological	intracranial_htn	Intracranial hypertension	95	63	63	-	-	3	47	46	51
Perinatal	congenital_septal	Congenital malformations of cardiac septa	593	70	54	-	-	1	49	48	51
Perinatal	prematurity	Prematurity	405	100	0	-	-	0	35	35	0

Perinatal	spina_bifida	Spina bifida	387	81	28	11	-	0	30	29	30
Perinatal	post_term	Post-term infant	275	100	0	-	-	0	36	37	0
Perinatal	PDA	Patent ductus arteriosus	68	97	15	-	-	0	10	13	8
Perinatal	LBW	Slow fetal growth or low birth weight	28	89	11	-	-	0	31	31	23
Perinatal	neo_jaundice	Neonatal jaundice (excl haemolytic dz of the newborn)	27	100	0	-	-	0	43	27	48
Perinatal	intrauterine_hypoxia	Intrauterine hypoxia	24	100	0	-	-	0	31	31	45
Perinatal	downs	Down's syndrome	19	74	37	-	-	0	40	40	52
Perinatal	sepsis_newborn	Bacterial sepsis of newborn	14	100	0	-	-	0	46	31	56
Perinatal	HBW	High birth weight	9	100	0	-	-	0	39	47	18
Perinatal	RDN	Respiratory distress of newborn	--	--	--		-	--	--	--	--
Psychiatric	depression	Depression	78992	39	10	80	-	0	53	52	54
Psychiatric	anxiety	Anxiety disorders	47642	51	9	60	-	0	52	52	52
Psychiatric	alc_problems	Alcohol Problems	11591	86	23	3	-	1	59	59	59
Psychiatric	BAD	Bipolar affective disorder and mania	2036	44	34	66	-	0	49	48	50
Psychiatric	dementia	Dementia	1834	84	39	4	-	6	71	71	70
Psychiatric	dementia_ex_Alz	Dementia (excluding Alzheimer's)	1663	86	31	-	-	5	71	72	70
Psychiatric	substance_misuse	Other psychoactive substance misuse	1282	87	18	2	-	0	51	53	50
Psychiatric	schizo	Schizophrenia, schizotypal and delusional disorders	1091	79	48	27	-	0	42	45	40
Psychiatric	ocd	Obsessive-compulsive disorder	1008	59	9	49	-	0	48	48	47
Psychiatric	alzheimer	Alzheimer's disease	773	84	41	-	-	4	70	70	70
Psychiatric	PD	Personality disorders	752	81	28	-	-	0	39	39	39

Psychiatric	eating_dz	Anorexia and bulimia nervosa	593	81	9	31	-	0	23	23	27
Psychiatric	delirium	Delirium, not induced by alcohol and other psychoactive substances	568	16	86	-	-	0	69	69	70
Psychiatric	intell_dz	Intellectual disability	227	61	57	-	-	0	50	50	50
Psychiatric	autism	Autism and Asperger's syndrome	148	22	23	-	-	0	51	51	51
Psychiatric	ADHD	Hyperkinetic disorders	26	81	19	-	-	0	51	49	52
Respiratory	allergic_rhinitis	Allergic and chronic rhinitis	71060	43	1	81	-	0	32	33	30
Respiratory	asthma	Asthma	36500	70	44	80	-	0	40	40	37
Respiratory	sinusitis	Chronic sinusitis	31060	95	4	6	-	0	51	51	52
Respiratory	COPD	COPD	9658	68	63	17	-	3	63	62	63
Respiratory	COPD_excl_bronchitis_NOS	COPD_excl_bronchitis_NOS	9388	70	61	18	-	3	63	62	63
Respiratory	nasal_polyp	Nasal polyp	5050	76	37	36	-	0	50	49	50
Respiratory	sleep_apnoea	Sleep apnoea	4712	71	60	19	-	0	57	58	57
Respiratory	pleural_effusion	Pleural effusion	3417	16	93	1	-	0	64	63	65
Respiratory	bronchiectasis	Bronchiectasis	2658	76	45	36	-	1	61	60	62
Respiratory	pneumothorax	Pneumothorax	1533	56	32	42	-	0	40	45	38
Respiratory	resp_failure	Respiratory failure	1379	11	95	1	-	5	66	66	66
Respiratory	pulm_collapse	Pulmonary collapse (excl pneumothorax)	1294	11	90	-	-	0	64	64	64
Respiratory	hyper_nasal_turbs	Hypertrophy of nasal turbinates	1111	17	85	-	-	0	52	53	51
Respiratory	pulm_fibrosis	Other interstitial pulmonary diseases with fibrosis	868	53	63	24	-	11	66	65	66
Respiratory	pleural_plaque	Pleural plaque	699	42	64	7	-	0	67	66	67
Respiratory	aspiration_pneumo	Aspiration pneumonitis	460	19	84	-	-	17	67	67	67

Respiratory	asbestosis	Asbestosis	265	54	36	33	-	2	64	62	64
Skin	dermatitis	Dermatitis (atopc/contact/other/unspecified)	47292	92	3	14	-	0	51	51	52
Skin	seb_derm	Seborrheic dermatitis	19438	70	0	34	-	0	49	48	50
Skin	actinic_keratosis	Actinic keratosis	11807	93	11	-	-	0	64	63	64
Skin	urticaria	Urticaria	10245	98	3	3	-	0	53	52	54
Skin	psoriasis	Psoriasis	9927	88	15	31	-	0	48	48	47
Skin	rosacea	Rosacea	9693	98	2	5	-	0	53	52	55
Skin	acne	Acne	5738	97	1	5	-	0	34	35	28
Skin	lichen_planus	Lichen planus	2808	91	15	5	-	0	56	57	53
Skin	keratitis	Keratitis	1780	93	11	-	-	0	53	54	52
Skin	pilonidal	Pilonidal cyst/sinus	1636	90	24	-	-	0	34	32	35
Skin	vitiligo	Vitiligo	936	93	3	13	-	0	49	49	50
Skin	alopecia_areata	Alopecia areata	930	94	1	9	-	0	46	47	42
Skin	hidradenitis	Hidradenitis suppurativa	341	73	37	-	-	0	48	48	49

Table S5. Cases in the full UK Biobank (N = 502,356), cases with full linked EHR data (N = 231,303), prevalence at baseline, incidence rate by sex and age groups (per 10,000 person years) derived from participants with full linked EHR data and a single continuous primary care registration (N = 156,266). – denotes N < 5

		N cases in full UKB cohort			N cases in full linked cohort			Prevalence at baseline (per 10,000)		Observed Incidence rate (per 10,000 person years)		Observed Incidence rate by age group (per 10,000 person years)					
	phenotype	Total	Female	Male	Total	Female	Male	Female	Male	Female	Male	40-44	45-49	50-54	55-59	60-64	65-69
Benign neoplasm or Carcinoma in situ	benign_brain	3163	1957	1206	1610	1009	601	38.66 (34.49,42.84)	26.74 (22.95,30.53)	3.25 (2.78,3.72)	2.66 (2.19,3.12)	1.71 (0.9,2.53)	2.31 (1.49,3.12)	1.91 (1.22,2.59)	3.39 (2.57,4.22)	2.93 (2.27,3.59)	4.59 (3.65,5.53)
Benign neoplasm or Carcinoma in situ	benign_colon	53843	24573	29270	25450	11699	13751	231.63 (221.51,241.75)	340.46 (327.16,353.76)	52.99 (51.06,54.91)	83.38 (80.71,86.06)	21.61 (18.7,24.52)	36.2 (32.95,39.45)	47.89 (44.41, 51.36)	68.07 (64.29,71.84)	85.84 (82.17,89.51)	101.17 (96.57,105.77)
Benign neoplasm or Carcinoma in situ	benign_ovary	11417	11417	0	6574	6574	0	352.92 (340.51,365.34)	0.0 (0.0,0.0)	14.68 (13.66,15.69)	0.0 (0.0,0.0)	13.32 (11.02, 15.62)	10.5 (8.75,12.25)	8.08 (6.65,9.5)	5.74 (4.66,6.82)	6.37 (5.4,7.35)	7.14 (5.95,8.32)
Benign neoplasm or Carcinoma in situ	benign_stomach	12650	7826	4824	5797	3641	2156	50.1 (45.35,54.85)	38.64 (34.09,43.19)	16.47 (15.41,17.53)	11.16 (10.21,12.11)	4.03 (2.78,5.28)	6.18 (4.85,7.5)	10.94 (9.31,12.58)	14.45 (12.75,16.16)	16.87 (15.29,18.46)	22.9 (20.78,25.01)
Benign neoplasm or	benign_uterus	13993	13993	0	6918	6918	0	334.77 (322.67,346.88)	0.0 (0.0,0.0)	21.42 (20.19,22.65)	0.0 (0.0,0.0)	12.5 (10.29, 14.71)	17.21 (14.97,19.44)	14.55 (12.65, 16.46)	11.02 (9.52,12.52)	8.87 (7.71,10.03)	8.32 (7.04,9.6)

Carcinoma in situ																	
Benign neoplasm or Carcinoma in situ	cin_cervical	34606	34606	0	30987	30987	0	2226.47 (2198.47,2254.46)	0.0 (0.0,0.0)	55.17 (52.96,57.38)	0.0 (0.0,0.0)	52.85 (47.98, 57.72)	47.47 (43.48,51.46)	38.68 (35.36, 42.0)	32.63 (29.89,35.37)	13.1 (11.61,14.58)	1.95 (1.3,2.59)
Benign neoplasm or Carcinoma in situ	haemangioma	6000	3548	2452	4504	2704	1800	123.65 (116.22,131.09)	95.9 (88.75,103.04)	9.19 (8.4,9.98)	7.71 (6.91,8.5)	6.92 (5.28,8.57)	8.64 (7.06,10.22)	7.38 (6.03,8.72)	8.44 (7.13,9.74)	9.28 (8.1,10.46)	9.2 (7.86,10.54)
Benign neoplasm or Carcinoma in situ	leiomyoma	26629	26629	0	14457	14457	0	876.18 (857.16,895.21)	0.0 (0.0,0.0)	30.32 (28.81,31.82)	0.0 (0.0,0.0)	37.87 (33.97, 41.76)	36.09 (32.8,39.39)	18.49 (16.3,20.68)	9.82 (8.38,11.26)	7.16 (6.1,8.21)	5.57 (4.51,6.63)
Cancers	MDS	810	314	496	339	136	203	1.89 (0.96,2.81)	2.66 (1.46,3.86)	0.42 (0.25,0.59)	1.14 (0.83,1.44)	0.3 (-0.04,0.64)	0.22 (-0.03,0.47)	0.38 (0.08,0.68)	0.88 (0.46,1.3)	0.54 (0.26,0.82)	1.76 (1.18,2.34)
Cancers	MGUS	1361	624	737	797	387	410	7.43 (5.59,9.26)	8.54 (6.4,10.68)	2.3 (1.91,2.7)	2.19 (1.77,2.61)	0.2 (-0.08,0.48)	0.96 (0.44,1.49)	1.08 (0.56,1.59)	1.3 (0.79,1.81)	3.38 (2.68,4.09)	4.53 (3.59,5.46)
Cancers	NHL	4199	1891	2308	1960	886	1074	25.58 (22.18,28.98)	35.56 (31.19,39.92)	3.81 (3.3,4.31)	5.85 (5.16,6.54)	1.51 (0.75,2.28)	1.86 (1.13,2.59)	2.54 (1.75,3.32)	4.48 (3.54,5.43)	6.63 (5.64,7.63)	7.83 (6.59,9.06)
Cancers	PCV	801	257	544	406	125	281	4.01 (2.66,5.35)	12.6 (10.0,15.2)	0.39 (0.22,0.55)	0.86 (0.6,1.13)	0.1 (-0.1,0.3)	0.07 (-0.07,0.22)	0.7 (0.28,1.11)	0.52 (0.2,0.84)	0.77 (0.43,1.1)	1.01 (0.56,1.45)
Cancers	hodgkins	794	369	425	372	179	193	9.67 (7.57,11.76)	10.92 (8.5,13.34)	0.46 (0.28,0.63)	0.61 (0.39,0.83)	0.0 (0.0,0.0)	0.45 (0.09,0.8)	0.63 (0.24,1.03)	0.42 (0.13,0.7)	0.58 (0.28,0.87)	0.8 (0.41,1.2)

Cancers	leukaemia	2651	1091	1560	1193	497	696	11.2 (8.95,13.45)	22.12 (18.67,25.56)	1.99 (1.62,2.35)	4.3 (3.71,4.9)	0.2 (-0.08,0.48)	1.04 (0.49,1.58)	2.47 (1.7,3.25)	2.03 (1.39,2.67)	4.43 (3.62,5.23)	5.44 (4.41,6.46)
Cancers	plasmacell	1632	685	947	789	329	460	5.19 (3.65,6.72)	8.4 (6.28,10.52)	1.42 (1.11,1.73)	2.46 (2.02,2.91)	0.4 (0.01,0.8)	0.59 (0.18,1.0)	0.95 (0.47,1.43)	1.35 (0.83,1.87)	3.04 (2.37,3.7)	3.32 (2.52,4.12)
Cancers	pri_LN	199	108	91	104	57	47	3.89 (2.56,5.22)	4.06 (2.58,5.54)	0.11 (0.02,0.19)	0.11 (0.01,0.2)	0.1 (-0.1,0.3)	0.07 (-0.07,0.22)	0.06 (-0.06,0.19)	0.1 (-0.04,0.25)	0.04 (-0.04,0.11)	0.25 (0.03,0.47)
Cancers	pri_adrenal	115	57	58	56	24	32	0.83 (0.21,1.44)	1.12 (0.34,1.9)	0.04 (-0.01,0.08)	0.17 (0.05,0.28)	0.2 (-0.08,0.48)	0.07 (-0.07,0.22)	0.06 (-0.06,0.19)	0.05 (-0.05,0.15)	0.04 (-0.04,0.11)	0.2 (0.0,0.4)
Cancers	pri_biliary	832	404	428	360	169	191	1.18 (0.45,1.91)	1.96 (0.93,2.99)	0.84 (0.6,1.08)	1.16 (0.85,1.46)	0.0 (0.0,0.0)	0.15 (-0.06,0.35)	0.57 (0.2,0.94)	0.94 (0.5,1.37)	1.38 (0.93,1.83)	1.91 (1.3,2.51)
Cancers	pri_bladder	4584	1161	3423	2109	561	1548	16.27 (13.56,18.98)	48.44 (43.35,53.53)	2.13 (1.75,2.51)	7.94 (7.14,8.75)	1.01 (0.38,1.63)	0.82 (0.33,1.3)	1.84 (1.17,2.51)	4.01 (3.12,4.91)	5.87 (4.93,6.8)	11.0 (9.53,12.46)
Cancers	pri_bone	442	200	242	201	94	107	4.72 (3.25,6.18)	5.32 (3.63,7.01)	0.19 (0.08,0.31)	0.44 (0.25,0.63)	0.1 (-0.1,0.3)	0.07 (-0.07,0.22)	0.13 (-0.05,0.3)	0.47 (0.16,0.77)	0.5 (0.23,0.77)	0.3 (0.06,0.54)
Cancers	pri_bowel	10350	4662	5688	4712	2142	2570	52.34 (47.48,57.19)	75.18 (68.84,81.51)	9.26 (8.46,10.05)	14.35 (13.27,15.44)	2.21 (1.29,3.14)	3.94 (2.88,5.0)	7.89 (6.5,9.28)	10.74 (9.27,12.21)	14.64 (13.16,16.11)	21.31 (19.27,23.36)
Cancers	pri_brain	1578	731	847	703	337	366	7.54 (5.7,9.39)	8.96 (6.77,11.15)	1.23 (0.94,1.52)	2.0 (1.6,2.4)	0.7 (0.18,1.23)	0.67 (0.23,1.1)	1.14 (0.61,1.67)	1.61 (1.04,2.18)	2.11 (1.55,2.67)	2.26 (1.6,2.92)
Cancers	pri_breast	23016	22846	170	10401	10334	67	431.9 (418.22,445.58)	3.36 (2.02,4.7)	37.24 (35.61,38.87)	0.27 (0.12,0.42)	14.92 (12.51,17.34)	19.13 (16.78,21.48)	16.91 (14.86,18.96)	19.93 (17.91,21.96)	24.24 (22.31,26.17)	19.76 (17.77,21.75)
Cancers	pri_cervical	2444	2444	0	1196	1196	0	85.34 (79.15,91.53)	0.0 (0.0,0.0)	0.64 (0.43,0.85)	0.0 (0.0,0.0)	0.3 (-0.04,0.65)	0.37 (0.05,0.7)	0.38 (0.08,0.69)	0.42 (0.13,0.71)	0.42 (0.17,0.67)	0.15 (-0.02,0.32)

Cancers	pri_kidney	2871	1042	1829	1353	483	870	8.84 (6.84,10.84)	24.36 (20.74,27.97)	2.06 (1.68,2.43)	4.52 (3.91,5.12)	1.01 (0.38,1.63)	1.56 (0.89,2.22)	1.96 (1.27,2.66)	2.97 (2.2,3.74)	4.39 (3.58,5.19)	4.94 (3.96,5.91)
Cancers	pri_liver	893	303	590	385	128	257	2.83 (1.7,3.96)	5.88 (4.1,7.66)	0.35 (0.2,0.51)	0.95 (0.67,1.22)	0.1 (-0.1,0.3)	0.0 (0.0,0.0)	0.38 (0.08,0.68)	0.52 (0.2,0.84)	0.92 (0.55,1.29)	1.21 (0.72,1.69)
Cancers	pri_lung	6303	2949	3354	2745	1305	1440	11.43 (9.16,13.71)	26.6 (22.82,30.38)	6.03 (5.4,6.67)	7.19 (6.43,7.96)	1.21 (0.52,1.89)	1.56 (0.89,2.22)	3.8 (2.84,4.77)	5.83 (4.75,6.91)	8.35 (7.24,9.46)	13.21 (11.61,14.81)
Cancers	pri_melanoma	9592	5187	4405	4567	2507	2060	126.72 (119.19,134.25)	107.23 (99.68,114.79)	6.67 (5.99,7.34)	7.97 (7.16,8.78)	3.24 (2.12,4.36)	4.03 (2.96,5.11)	5.95 (4.74,7.16)	6.84 (5.66,8.01)	9.45 (8.26,10.64)	10.09 (8.68,11.5)
Cancers	pri_mesothelioma	523	100	423	222	31	191	0.0 (0.0,0.0)	0.56 (0.01,1.11)	0.21 (0.09,0.33)	1.3 (0.98,1.63)	0.0 (0.0,0.0)	0.07 (-0.07,0.22)	0.0 (0.0,0.0)	0.42 (0.13,0.7)	0.77 (0.43,1.1)	2.26 (1.6,2.92)
Cancers	pri_multindep	1296	540	756	513	214	299	0.0 (0.0,0.0)	0.0 (0.0,0.0)	0.58 (0.38,0.78)	0.44 (0.25,0.63)	0.2 (-0.08,0.48)	0.15 (-0.06,0.35)	0.38 (0.08,0.68)	0.31 (0.06,0.56)	0.65 (0.34,0.96)	1.05 (0.6,1.5)
Cancers	pri_oesoph	1809	484	1325	815	229	586	2.36 (1.32,3.39)	9.8 (7.5,12.09)	0.91 (0.67,1.16)	3.31 (2.79,3.82)	0.3 (-0.04,0.64)	0.37 (0.05,0.7)	0.89 (0.42,1.35)	1.82 (1.22,2.42)	2.11 (1.55,2.67)	4.88 (3.91,5.85)
Cancers	pri_oroph	2467	878	1589	1111	405	706	15.44 (12.8,18.08)	30.24 (26.21,34.27)	1.39 (1.08,1.7)	2.89 (2.41,3.38)	0.5 (0.06,0.94)	1.86 (1.13,2.58)	1.33 (0.76,1.9)	2.4 (1.7,3.09)	2.46 (1.86,3.07)	2.77 (2.04,3.5)
Cancers	pri_other	9234	4624	4610	4284	2175	2109	52.22 (47.37,57.07)	61.32 (55.59,67.04)	9.67 (8.86,10.48)	11.4 (10.44,12.37)	2.52 (1.53,3.51)	4.16 (3.07,5.25)	6.55 (5.29,7.82)	9.3 (7.94,10.67)	14.03 (12.58,15.47)	18.31 (16.42,20.2)
Cancers	pri_ovarian	2677	2677	0	1306	1306	0	48.68 (44.0,53.37)	0.0 (0.0,0.0)	4.59 (4.03,5.15)	0.0 (0.0,0.0)	1.11 (0.45,1.76)	1.41 (0.78,2.04)	1.78 (1.12,2.43)	2.97 (2.2,3.74)	2.85 (2.2,3.5)	3.58 (2.75,4.41)
Cancers	pri_pancr	1898	873	1025	820	362	458	1.77 (0.87,2.66)	3.36 (2.02,4.7)	1.76 (1.41,2.1)	2.46 (2.02,2.91)	0.2 (-0.08,0.48)	0.37 (0.05,0.7)	1.39 (0.81,1.97)	1.3 (0.79,1.81)	3.07 (2.4,3.74)	4.17 (3.27,5.06)

Cancers	pri_prost	15157	0	15157	6715	0	6715	0.0 (0.0,0.0)	165.75 (156.39,175.11)	0.0 (0.0,0.0)	41.02 (39.18,42.87)	0.5 (0.06,0.94)	2.97 (2.05,3.89)	7.75 (6.37,9.12)	14.52 (12.81,16.23)	27.86 (25.82,29.91)	38.24 (35.48,41.0)
Cancers	pri_skin	36059	17357	18702	17194	8331	8863	257.68 (247.02,268.34)	292.73 (280.36,305.09)	39.78 (38.11,41.45)	50.79 (48.72,52.87)	12.3 (10.11, 14.49)	18.29 (15.99,20.59)	25.28 (22.77, 27.79)	39.07 (36.22,41.91)	60.54 (57.48,63.61)	82.13 (77.99,86.27)
Cancers	pri_stomach	1491	476	1015	686	228	458	4.48 (3.06,5.9)	8.12 (6.03,10.21)	0.88 (0.63,1.12)	2.84 (2.36,3.32)	0.2 (-0.08,0.48)	0.82 (0.33,1.3)	0.95 (0.47,1.43)	1.61 (1.04,2.18)	1.88 (1.35,2.41)	3.87 (3.01,4.73)
Cancers	pri_testis	994	0	994	471	0	471	0.0 (0.0,0.0)	41.44 (36.73,46.15)	0.0 (0.0,0.0)	0.61 (0.39,0.84)	0.6 (0.12,1.09)	0.59 (0.18,1.01)	0.13 (-0.05, 0.3)	0.1 (-0.04,0.25)	0.27 (0.07,0.47)	0.2 (0.0,0.4)
Cancers	pri_thyroid	1083	818	265	525	403	122	17.8 (14.96,20.64)	4.34 (2.81,5.87)	1.27 (0.97,1.56)	0.44 (0.25,0.63)	0.5 (0.06,0.94)	1.26 (0.66,1.86)	0.95 (0.47,1.43)	1.09 (0.63,1.56)	0.92 (0.55,1.29)	0.55 (0.23,0.88)
Cancers	pri_uterine	3349	3349	0	1537	1537	0	56.7 (51.65,61.75)	0.0 (0.0,0.0)	5.55 (4.94,6.17)	0.0 (0.0,0.0)	0.7 (0.18,1.23)	1.48 (0.83,2.14)	2.54 (1.75,3.32)	3.44 (2.61,4.27)	3.86 (3.1,4.61)	4.09 (3.2,4.98)
Cancers	sec_LN	12046	7312	4734	5470	3362	2108	61.65 (56.38,66.92)	22.68 (19.19,26.17)	16.03 (14.98,17.07)	12.75 (11.74,13.77)	6.05 (4.52,7.58)	9.24 (7.61,10.86)	10.51 (8.9,12.11)	14.08 (12.4,15.76)	18.41 (16.76,20.07)	20.96 (18.94,22.98)
Cancers	sec_adrenal	923	410	513	390	168	222	0.12 (-0.11,0.35)	0.0 (0.0,0.0)	0.67 (0.46,0.88)	1.45 (1.11,1.79)	0.2 (-0.08,0.48)	0.22 (-0.03,0.47)	0.57 (0.2,0.94)	1.04 (0.58,1.49)	1.46 (0.99,1.92)	1.76 (1.17,2.34)
Cancers	sec_bone	5964	2672	3292	2580	1118	1462	5.3 (3.76,6.85)	4.76 (3.16,6.36)	6.03 (5.39,6.67)	8.7 (7.86,9.53)	1.71 (0.9,2.52)	2.45 (1.61,3.28)	3.99 (3.0,4.97)	6.24 (5.12,7.36)	10.03 (8.81,11.24)	13.17 (11.57,14.76)
Cancers	sec_bowel	598	368	230	260	155	105	0.94 (0.29,1.6)	0.56 (0.01,1.11)	0.77 (0.54,1.0)	0.46 (0.27,0.66)	0.3 (-0.04,0.64)	0.44 (0.09,0.8)	0.38 (0.08,0.68)	0.52 (0.2,0.84)	0.65 (0.34,0.96)	1.2 (0.72,1.69)
Cancers	sec_brain	2155	1185	970	938	523	415	1.06 (0.37,1.75)	0.84 (0.17,1.51)	3.13 (2.67,3.59)	2.97 (2.48,3.46)	1.0 (0.38,1.63)	1.19 (0.6,1.77)	2.66 (1.85,3.46)	2.75 (2.01,3.49)	4.26 (3.47,5.05)	4.37 (3.45,5.28)

Cancers	sec_liver	7124	3653	3471	2988	1510	1478	5.89 (4.26,7.53)	6.58 (4.7,8.46)	7.47 (6.76,8.18)	9.27 (8.4,10.13)	2.01 (1.13,2.89)	3.63 (2.62,4.65)	5.57 (4.41,6.74)	7.43 (6.22,8.65)	11.18 (9.9,12.47)	13.77 (12.14,15.4)
Cancers	sec_lung	5175	2601	2574	2194	1067	1127	3.54 (2.27,4.8)	5.6 (3.86,7.33)	5.8 (5.17,6.43)	7.43 (6.66,8.21)	1.31 (0.6,2.02)	3.26 (2.3,4.23)	4.62 (3.56,5.68)	5.61 (4.56,6.67)	9.34 (8.16,10.51)	10.15 (8.75,11.55)
Cancers	sec_other	5653	3061	2592	2682	1463	1219	12.38 (10.01,14.74)	9.38 (7.13,11.62)	6.97 (6.28,7.65)	6.42 (5.7,7.14)	1.31 (0.6,2.02)	3.19 (2.24,4.15)	4.88 (3.79,5.97)	6.45 (5.32,7.59)	8.34 (7.23,9.45)	11.41 (9.93,12.9)
Cancers	sec_peritoneum	3524	2298	1226	1576	1026	550	5.19 (3.65,6.72)	0.7 (0.09,1.31)	5.03 (4.44,5.61)	3.53 (3.0,4.07)	1.01 (0.38,1.63)	2.3 (1.49,3.11)	3.17 (2.29,4.04)	4.37 (3.43,5.3)	5.53 (4.63,6.43)	6.78 (5.64,7.92)
Cancers	sec_pleura	1628	1056	572	709	443	266	1.18 (0.45,1.91)	0.28 (-0.11,0.67)	2.21 (1.83,2.6)	1.94 (1.54,2.33)	0.4 (0.01,0.8)	0.82 (0.33,1.3)	1.39 (0.81,1.97)	2.75 (2.01,3.49)	2.57 (1.96,3.19)	3.06 (2.29,3.83)
Cardiovascular	AAA	3464	653	2811	1501	287	1214	5.19 (3.65,6.72)	22.96 (19.45,26.47)	0.88 (0.64,1.12)	5.89 (5.2,6.58)	0.3 (-0.04,0.64)	0.52 (0.13,0.9)	0.63 (0.24,1.03)	2.13 (1.48,2.78)	4.65 (3.83,5.48)	7.41 (6.22,8.61)
Cardiovascular	AF	36619	13383	23236	17168	6310	10858	130.02 (122.4,137.64)	343.12 (329.77,356.47)	22.79 (21.54,24.05)	51.91 (49.81,54.01)	4.95 (3.56,6.33)	9.05 (7.43,10.66)	18.83 (16.67,20.98)	25.69 (23.4,27.98)	47.33 (44.64,50.03)	80.81 (76.72,84.91)
Cardiovascular	CHD_NOS	52194	16601	35593	25762	8469	17293	330.06 (318.03,342.08)	915.0 (893.85,936.14)	22.72 (21.46,23.98)	59.66 (57.34,61.98)	8.42 (6.61,10.23)	15.69 (13.55,17.82)	24.94 (22.43,27.45)	34.86 (32.14,37.58)	50.08 (47.22,52.93)	75.52 (71.4,79.64)
Cardiovascular	Intracereb_haem	2446	1085	1361	1175	491	684	10.84 (8.63,13.06)	23.66 (20.1,27.22)	1.56 (1.24,1.89)	2.89 (2.41,3.37)	0.6 (0.12,1.09)	0.74 (0.28,1.2)	1.27 (0.71,1.82)	1.93 (1.31,2.55)	2.19 (1.62,2.76)	4.83 (3.87,5.8)
Cardiovascular	Isch_stroke	9179	3550	5629	4466	1740	2726	30.29 (26.6,33.99)	67.06 (61.07,73.04)	6.14 (5.49,6.79)	12.96 (11.93,13.98)	2.32 (1.37,3.26)	2.53 (1.68,3.38)	5.72 (4.54,6.9)	7.32 (6.11,8.53)	10.37 (9.13,11.61)	20.5 (18.5,22.5)
Cardiovascular	LBBB	5353	2249	3104	2397	1036	1361	10.49 (8.31,12.67)	19.32 (16.1,22.54)	4.52 (3.97,5.08)	6.69 (5.95,7.43)	0.6 (0.12,1.09)	0.96 (0.44,1.49)	2.66 (1.86,3.47)	4.84 (3.86,5.83)	6.7 (5.71,7.7)	12.42 (10.87,13.97)

Cardiovascular	PE	11778	5928	5850	5493	2788	2705	88.17 (81.88,94.46)	91.14 (84.17,98.1)	7.91 (7.18,8.65)	10.04 (9.13,10.94)	3.23 (2.11,4.35)	4.32 (3.21,5.44)	5.55 (4.38,6.72)	7.92 (6.66,9.18)	11.47 (10.16,12.78)	15.03 (13.32,16.75)
Cardiovascular	RBBB	5656	1729	3927	2629	805	1824	11.79 (9.48,14.1)	31.78 (27.65,35.91)	3.4 (2.92,3.88)	8.9 (8.05,9.75)	1.91 (1.05,2.77)	2.3 (1.49,3.11)	3.17 (2.29,4.05)	5.37 (4.33,6.4)	7.01 (5.99,8.03)	11.57 (10.07,13.07)
Cardiovascular	Rh_valve	2317	1201	1116	1027	544	483	15.56 (12.91,18.21)	9.24 (7.01,11.47)	1.46 (1.15,1.77)	1.85 (1.47,2.24)	0.6 (0.12,1.09)	0.59 (0.18,1.0)	0.76 (0.33,1.19)	1.2 (0.71,1.69)	1.85 (1.32,2.37)	3.72 (2.88,4.57)
Cardiovascular	SVT	5733	3069	2664	3014	1672	1342	66.84 (61.35,72.32)	54.04 (48.66,59.41)	4.89 (4.31,5.46)	5.1 (4.46,5.75)	3.03 (1.94,4.11)	2.53 (1.68,3.38)	3.82 (2.85,4.79)	5.65 (4.59,6.72)	5.85 (4.91,6.78)	6.8 (5.65,7.95)
Cardiovascular	Stroke_NOS	8087	3296	4791	5530	2274	3256	140.39 (132.47,148.31)	248.77 (237.35,260.19)	2.57 (2.15,2.99)	4.71 (4.09,5.34)	0.51 (0.06,0.95)	0.82 (0.34,1.31)	1.98 (1.29,2.68)	2.64 (1.91,3.38)	4.05 (3.27,4.84)	8.48 (7.17,9.78)
Cardiovascular	Subarach	2129	1284	845	1130	682	448	27.82 (24.27,31.36)	24.08 (20.48,27.67)	1.83 (1.48,2.18)	1.79 (1.41,2.17)	1.01 (0.38,1.63)	1.63 (0.95,2.32)	1.59 (0.96,2.21)	2.14 (1.48,2.79)	1.62 (1.13,2.11)	2.47 (1.78,3.16)
Cardiovascular	TIA	11006	4738	6268	5913	2579	3334	80.04 (74.04,86.03)	137.89 (129.34,146.45)	10.05 (9.22,10.88)	14.7 (13.6,15.8)	2.42 (1.45,3.38)	4.31 (3.2,5.42)	6.11 (4.89,7.34)	9.93 (8.51,11.34)	16.35 (14.78,17.92)	24.2 (22.01,26.39)
Cardiovascular	VT	2129	532	1597	1010	262	748	6.37 (4.67,8.06)	17.08 (14.05,20.11)	1.02 (0.76,1.28)	3.5 (2.97,4.03)	0.5 (0.06,0.94)	1.19 (0.61,1.77)	1.27 (0.71,1.82)	1.82 (1.22,2.42)	2.88 (2.23,3.54)	3.67 (2.83,4.52)
Cardiovascular	av_block_1	4000	1101	2899	1842	527	1315	3.06 (1.89,4.24)	10.92 (8.5,13.34)	1.9 (1.54,2.26)	6.75 (6.01,7.49)	0.9 (0.31,1.5)	1.78 (1.07,2.49)	1.71 (1.07,2.36)	2.7 (1.97,3.44)	5.54 (4.63,6.44)	8.66 (7.37,9.96)
Cardiovascular	av_block_2	1452	475	977	654	212	442	2.12 (1.14,3.1)	5.18 (3.51,6.85)	0.72 (0.5,0.94)	2.23 (1.81,2.66)	0.5 (0.06,0.94)	0.82 (0.33,1.3)	0.51 (0.16,0.86)	1.14 (0.67,1.62)	1.61 (1.12,2.1)	2.96 (2.21,3.72)
Cardiovascular	av_block_3	1831	531	1300	777	217	560	3.3 (2.08,4.52)	6.86 (4.94,8.78)	0.77 (0.54,1.0)	2.17 (1.75,2.59)	0.4 (0.01,0.8)	0.37 (0.05,0.7)	0.44 (0.11,0.77)	1.14 (0.67,1.62)	1.31 (0.87,1.74)	3.77 (2.92,4.62)

Cardiovascular	bifasc_block	388	84	304	159	42	117	0.0 (0.0,0.0)	0.28 (-0.11,0.67)	0.14 (0.04,0.24)	0.55 (0.34,0.76)	0.0 (0.0,0.0)	0.07 (-0.07,0.22)	0.06 (-0.06, 0.19)	0.26 (0.03,0.49)	0.35 (0.12,0.57)	0.9 (0.49,1.32)
Cardiovascular	cardiomyo_oth	2753	921	1832	1320	451	869	7.19 (5.39,8.99)	21.98 (18.54,25.41)	1.86 (1.51,2.22)	4.35 (3.75,4.94)	0.7 (0.18,1.23)	1.26 (0.66,1.86)	2.22 (1.48,2.95)	2.65 (1.93,3.38)	3.62 (2.89,4.35)	5.44 (4.41,6.46)
Cardiovascular	dcm	1569	457	1112	706	207	499	2.95 (1.79,4.1)	16.52 (13.54,19.5)	0.97 (0.71,1.22)	2.7 (2.23,3.17)	0.2 (-0.08,0.48)	0.89 (0.39,1.39)	1.46 (0.86,2.05)	1.14 (0.67,1.62)	2.27 (1.69,2.85)	3.27 (2.47,4.06)
Cardiovascular	hf	17761	6236	11525	8626	2999	5627	41.37 (37.06,45.69)	142.51 (133.82,151.21)	10.48 (9.64,11.33)	26.69 (25.2,28.17)	2.42 (1.45,3.38)	4.61 (3.46,5.76)	9.1 (7.61,10.6)	13.7 (12.04,15.36)	22.23 (20.4,24.05)	40.09 (37.27,42.91)
Cardiovascular	hocm	724	255	469	340	136	204	3.65 (2.37,4.94)	7.56 (5.54,9.58)	0.61 (0.41,0.82)	0.95 (0.67,1.22)	0.2 (-0.08,0.48)	0.67 (0.23,1.1)	0.32 (0.04,0.59)	0.94 (0.5,1.37)	0.84 (0.49,1.2)	1.21 (0.72,1.69)
Cardiovascular	hypertension	167650	80028	87622	85056	41056	44000	2008.63 (1981.67,2035.59)	2609.47 (2577.27, 2641.68)	142.14 (138.59,145.7)	210.14 (205.16,215.12)	68.47 (63.1,73.84)	97.91 (92.26,103.57)	130.49 (124.22,136.76)	165.02 (158.33, 171.71)	219.73 (212.68,226.78)	317.03 (306.49,327.57)
Cardiovascular	mult_valve	7066	3049	4017	2797	1202	1595	9.55 (7.47,11.63)	12.32 (9.75,14.89)	3.29 (2.82,3.76)	6.98 (6.23,7.73)	0.7 (0.18,1.22)	1.19 (0.61,1.77)	1.65 (1.01,2.28)	4.84 (3.86,5.82)	5.7 (4.78,6.61)	11.5 (10.0,12.99)
Cardiovascular	myocardial_infarction	27582	7383	20199	12900	3515	9385	207.11 (197.53,216.69)	723.77 (704.76,742.77)	3.37 (2.89,3.86)	11.84 (10.82,12.86)	2.44 (1.46,3.41)	4.14 (3.05,5.23)	5.92 (4.7,7.13)	7.29 (6.06,8.52)	8.7 (7.53,9.86)	10.49 (9.0,11.98)
Cardiovascular	nonRh_aortic	7940	3036	4904	3806	1469	2337	28.64 (25.05,32.24)	54.74 (49.33,60.15)	5.82 (5.19,6.45)	11.69 (10.71,12.66)	1.21 (0.52,1.89)	2.38 (1.55,3.2)	3.3 (2.4,4.2)	6.79 (5.62,7.95)	10.44 (9.19,11.68)	19.59 (17.63,21.54)
Cardiovascular	nonRh_mitral	7042	3296	3746	3348	1630	1718	44.32 (39.85,48.79)	48.72 (43.61,53.82)	5.19 (4.6,5.79)	7.52 (6.74,8.3)	1.41 (0.67,2.15)	2.38 (1.55,3.2)	3.31 (2.41,4.21)	5.27 (4.24,6.3)	7.23 (6.19,8.26)	13.35 (11.74,14.97)

Cardiovascular	pericardial_effusion	2415	1071	1344	1226	519	707	10.37 (8.21,12.54)	29.26 (25.3,33.22)	2.2 (1.81,2.58)	2.62 (2.16,3.08)	0.8 (0.25,1.36)	1.56 (0.89,2.23)	2.09 (1.38,2.81)	1.93 (1.31,2.55)	3.19 (2.51,3.88)	3.37 (2.57,4.18)
Cardiovascular	peripheral_arterial_disease	9441	3347	6094	4903	1773	3130	60.94 (55.71,66.18)	126.55 (118.36,134.75)	5.72 (5.09,6.34)	13.6 (12.54,14.65)	1.41 (0.67,2.15)	3.28 (2.31,4.24)	3.63 (2.69,4.57)	8.29 (6.99,9.58)	12.06 (10.72,13.41)	19.32 (17.37,21.27)
Cardiovascular	prim_pulm_htn	823	427	396	392	206	186	4.72 (3.25,6.18)	4.9 (3.28,6.52)	1.23 (0.94,1.52)	1.35 (1.02,1.68)	0.4 (0.01,0.8)	0.3 (0.01,0.59)	0.13 (-0.05,0.3)	1.09 (0.62,1.56)	1.38 (0.93,1.83)	3.37 (2.56,4.17)
Cardiovascular	raynauds	6188	4270	1918	4218	2888	1330	133.55 (125.83,141.28)	58.8 (53.19,64.4)	10.34 (9.5,11.18)	7.06 (6.3,7.82)	9.67 (7.73,11.62)	9.01 (7.4,10.62)	6.41 (5.15,7.66)	8.73 (7.4,10.06)	8.71 (7.57,9.85)	10.54 (9.1,11.97)
Cardiovascular	sec_pulm_htn	1825	848	977	754	370	384	1.18 (0.45,1.91)	0.56 (0.01,1.11)	1.16 (0.88,1.44)	1.54 (1.18,1.89)	0.3 (-0.04,0.64)	0.59 (0.18,1.0)	0.57 (0.2,0.94)	0.83 (0.42,1.24)	1.77 (1.26,2.28)	2.86 (2.12,3.6)
Cardiovascular	sick_sinus	1302	556	746	645	271	374	4.6 (3.15,6.04)	7.7 (5.67,9.73)	1.05 (0.79,1.32)	1.71 (1.33,2.08)	0.1 (-0.1,0.3)	0.44 (0.09,0.8)	0.63 (0.24,1.03)	0.73 (0.35,1.11)	1.92 (1.39,2.45)	3.02 (2.25,3.78)
Cardiovascular	stable_angina	32804	11573	21231	17564	6150	11414	225.26 (215.28,235.25)	576.91 (559.81,594.01)	21.69 (20.47,22.92)	52.42 (50.28,54.56)	7.07 (5.41,8.72)	13.29 (11.33,15.25)	23.0 (20.6,25.39)	33.74 (31.09,36.39)	45.48 (42.8,48.16)	65.86 (62.1,69.62)
Cardiovascular	subdural_haem	953	322	631	437	149	288	4.6 (3.15,6.04)	7.28 (5.3,9.26)	0.53 (0.34,0.72)	1.16 (0.85,1.46)	0.4 (0.01,0.8)	0.22 (-0.03,0.47)	0.63 (0.24,1.03)	0.57 (0.23,0.91)	0.85 (0.49,1.2)	1.76 (1.18,2.34)
Cardiovascular	trifasc_block	359	63	296	158	33	125	0.0 (0.0,0.0)	0.7 (0.09,1.31)	0.11 (0.02,0.19)	0.42 (0.24,0.61)	0.0 (0.0,0.0)	0.07 (-0.07,0.22)	0.0 (0.0,0.0)	0.16 (-0.02,0.33)	0.35 (0.12,0.57)	0.65 (0.3,1.01)
Cardiovascular	unstable_angina	8927	2985	5942	4575	1532	3043	54.81 (49.84,59.78)	152.45 (143.47,161.44)	5.55 (4.94,6.17)	13.93 (12.86,15.0)	2.21 (1.29,3.14)	4.17 (3.08,5.26)	6.5 (5.24,7.76)	9.09 (7.73,10.44)	11.36 (10.05,12.66)	16.44 (14.63,18.24)
Cardiovascular	vte_ex_pe	16465	8908	7557	8112	4390	3722	238.11 (227.85,248.37)	196.97 (186.78,207.16)	8.59 (7.82,9.37)	13.21 (12.17,14.26)	3.97 (2.72,5.21)	7.06 (5.63,8.48)	7.22 (5.89,8.56)	10.31 (8.86,11.76)	12.82 (11.42,14.21)	17.07 (15.22,18.91)

Digestive	GORD	74365	41037	33328	39057	21809	17248	787.18 (769.06,805.31)	818.96 (798.85,839.07)	77.16 (74.76,79.57)	68.9 (66.41,71.39)	42.43 (38.26, 46.6)	54.5 (50.41,58.59)	66.24 (62.04, 70.44)	76.21 (72.09,80.32)	82.12 (78.42,85.81)	95.44 (90.83,100.05)
Digestive	IBD	8767	4549	4218	4472	2336	2136	134.85 (127.09,142.61)	144.47 (135.72,153.22)	3.78 (3.27,4.29)	4.89 (4.26,5.53)	3.35 (2.21,4.5)	3.83 (2.78,4.89)	4.24 (3.22,5.26)	4.48 (3.53,5.43)	4.76 (3.91,5.6)	4.29 (3.37,5.21)
Digestive	IBS	26707	19612	7095	14834	10906	3928	590.92 (575.05,606.79)	249.47 (238.03,260.91)	17.14 (16.03,18.25)	8.96 (8.1,9.83)	15.37 (12.87, 17.87)	13.41 (11.41,15.42)	13.57 (11.7,15.43)	13.92 (12.21,15.64)	13.33 (11.9,14.77)	11.55 (10.03,13.08)
Digestive	anal_fissure	8883	4434	4449	7030	3592	3438	191.55 (182.33,200.77)	211.53 (200.98,222.08)	9.61 (8.8,10.43)	12.56 (11.54,13.58)	14.65 (12.24, 17.05)	13.98 (11.95,16.0)	11.44 (9.75,13.13)	11.38 (9.85,12.9)	9.38 (8.19,10.57)	8.35 (7.07,9.63)
Digestive	angiodysplasia_colon	1346	644	702	619	300	319	3.77 (2.47,5.08)	2.66 (1.46,3.86)	1.0 (0.74,1.26)	1.56 (1.2,1.91)	0.1 (-0.1,0.3)	0.3 (0.01,0.59)	0.82 (0.38,1.27)	1.04 (0.58,1.49)	1.69 (1.19,2.19)	2.46 (1.77,3.15)
Digestive	anorectal_fistula	2938	1031	1907	1848	677	1171	36.07 (32.04,40.1)	75.32 (68.98,81.66)	1.57 (1.24,1.9)	3.35 (2.83,3.88)	3.33 (2.2,4.47)	2.91 (2.0,3.83)	3.12 (2.25,4.0)	2.46 (1.75,3.16)	1.81 (1.3,2.33)	1.61 (1.06,2.17)
Digestive	anorectal_prolapse	2876	2222	654	1506	1158	348	39.37 (35.16,43.59)	14.42 (11.64,17.2)	4.57 (4.02,5.13)	1.71 (1.34,2.08)	1.51 (0.75,2.27)	2.08 (1.31,2.85)	2.03 (1.33,2.73)	3.18 (2.38,3.98)	4.39 (3.59,5.2)	4.54 (3.6,5.48)
Digestive	appendicitis	18448	10336	8112	12085	6808	5277	492.26 (477.7,506.81)	462.12 (446.72,477.51)	6.39 (5.72,7.06)	5.37 (4.7,6.05)	7.89 (6.1,9.67)	4.89 (3.69,6.1)	6.25 (4.99,7.52)	6.52 (5.35,7.7)	5.27 (4.36,6.17)	5.66 (4.59,6.73)
Digestive	autoimm_liver	899	724	175	478	377	101	14.97 (12.37,17.57)	4.9 (3.28,6.52)	1.69 (1.35,2.03)	0.32 (0.16,0.48)	0.5 (0.06,0.94)	1.04 (0.49,1.58)	0.82 (0.38,1.27)	1.3 (0.79,1.81)	1.15 (0.74,1.57)	1.21 (0.72,1.69)
Digestive	barretts	8133	3115	5018	3908	1492	2416	37.48 (33.37,41.6)	75.46 (69.11,81.8)	6.43 (5.77,7.09)	12.08 (11.09,13.07)	3.32 (2.19,4.46)	4.69 (3.53,5.85)	6.49 (5.23,7.75)	9.26 (7.9,10.63)	11.98 (10.64,13.31)	12.61 (11.04,14.18)
Digestive	cholangitis	2155	949	1206	1022	452	570	7.31 (5.49,9.13)	8.4 (6.28,10.52)	1.79 (1.45,2.14)	2.55 (2.09,3.0)	0.6 (0.12,1.09)	0.82 (0.33,1.3)	1.52 (0.91,2.13)	1.87 (1.26,2.48)	2.69 (2.06,3.32)	3.82 (2.96,4.68)

Digestive	cholecystitis	16711	11041	5670	8365	5597	2768	198.27 (188.89,207.65)	89.46 (82.55,96.36)	18.5 (17.37,19.63)	13.04 (12.01,14.07)	12.29 (10.1,14.49)	12.76 (10.84,14.68)	14.66 (12.76,16.57)	14.64 (12.91,16.37)	16.78 (15.18,18.37)	21.46 (19.4,23.53)
Digestive	cholelithiasis	35015	24083	10932	17175	11914	5261	496.85 (482.23,511.48)	198.65 (188.42,208.88)	36.58 (34.96,38.2)	22.88 (21.5,24.26)	23.69 (20.63,26.75)	23.28 (20.67,25.89)	26.23 (23.66,28.8)	30.82 (28.28,33.36)	32.01 (29.78,34.24)	38.78 (35.96,41.61)
Digestive	cirrhosis	3512	1258	2254	1877	686	1191	18.39 (15.51,21.27)	38.64 (34.09,43.19)	2.64 (2.22,3.06)	5.33 (4.67,5.98)	1.91 (1.05,2.77)	2.38 (1.55,3.2)	4.0 (3.01,4.99)	4.28 (3.35,5.2)	4.47 (3.66,5.28)	4.53 (3.6,5.47)
Digestive	coeliac	6078	4169	1909	2926	1991	935	128.96 (121.37,136.55)	64.4 (58.53,70.26)	2.49 (2.08,2.91)	2.2 (1.78,2.63)	1.01 (0.39,1.64)	2.1 (1.32,2.88)	2.24 (1.5,2.98)	2.21 (1.54,2.87)	2.56 (1.94,3.17)	3.19 (2.4,3.98)
Digestive	crohns	3214	1776	1438	1579	867	712	46.21 (41.64,50.77)	44.94 (40.03,49.84)	1.64 (1.31,1.97)	1.84 (1.45,2.23)	1.82 (0.98,2.66)	1.42 (0.78,2.05)	1.65 (1.02,2.29)	1.93 (1.31,2.55)	1.97 (1.43,2.51)	1.46 (0.93,1.99)
Digestive	diverticuli	65365	35177	30188	30703	16635	14068	363.06 (350.47,375.65)	338.36 (325.1,351.62)	76.07 (73.73,78.4)	82.2 (79.55,84.86)	18.45 (15.77,21.13)	35.93 (32.7,39.16)	54.78 (51.07,58.49)	77.58 (73.53,81.63)	101.67 (97.63,105.7)	135.48 (130.07,140.89)
Digestive	fatty_liver	8070	3974	4096	4386	2106	2280	18.04 (15.18,20.89)	38.78 (34.22,43.34)	11.06 (10.2,11.93)	15.69 (14.56,16.82)	10.6 (8.57,12.63)	11.02 (9.25,12.8)	13.82 (11.98,15.66)	15.27 (13.52,17.02)	13.29 (11.88,14.69)	13.17 (11.57,14.77)
Digestive	gastritis_duodenitis	63275	34141	29134	33887	18261	15626	596.34 (580.41,612.28)	688.21 (669.64,706.77)	74.78 (72.44,77.12)	73.78 (71.22,76.34)	39.14 (35.15,43.12)	56.29 (52.15,60.43)	62.72 (58.67,66.77)	74.01 (70.0,78.02)	88.31 (84.51,92.11)	97.48 (92.87,102.09)
Digestive	hernia_abdo	47848	9867	37981	26250	5446	20804	229.74 (219.66,239.82)	1169.36 (1145.8,1192.93)	17.11 (16.02,18.2)	93.72 (90.75,96.7)	28.51 (25.11,31.91)	32.93 (29.78,36.07)	36.99 (33.9,40.08)	47.19 (43.99,50.38)	60.23 (57.1,63.37)	71.86 (67.89,75.82)
Digestive	hernia_diaphragm	56410	31945	24465	25789	14687	11102	476.93 (462.59,491.27)	454.84 (439.56,470.12)	56.28 (54.26,58.29)	50.76 (48.68,52.85)	21.99 (19.04,24.94)	34.89 (31.68,38.09)	41.89 (38.62,45.16)	53.45 (50.08,56.82)	66.26 (63.0,69.51)	78.19 (74.1,82.28)
Digestive	liver_alc	2141	466	1675	1107	234	873	7.19 (5.39,8.99)	32.62 (28.44,36.8)	0.7 (0.49,0.92)	4.37 (3.78,4.97)	1.91 (1.05,2.77)	1.86 (1.13,2.58)	2.41 (1.64,3.18)	2.81 (2.06,3.56)	2.46 (1.86,3.07)	2.36 (1.69,3.04)

Digestive	liver_fail	1728	661	1067	738	282	456	8.13 (6.22,10.05)	9.66 (7.38,11.94)	1.18 (0.9,1.46)	2.11 (1.69,2.52)	0.8 (0.25,1.36)	0.67 (0.23,1.1)	1.2 (0.66,1.74)	2.08 (1.44,2.72)	1.73 (1.22,2.23)	2.31 (1.64,2.98)
Digestive	oesoph_ulc	41498	20433	21065	22690	11317	11373	430.84 (417.18,444.51)	554.09 (537.32,570.87)	41.29 (39.57,43.01)	50.0 (47.92,52.09)	22.77 (19.76, 25.78)	30.89 (27.86,33.92)	37.55 (34.45, 40.65)	45.93 (42.8,49.05)	54.73 (51.78,57.68)	60.41 (56.84,63.99)
Digestive	pancreatitis	5099	2552	2547	2521	1279	1242	45.38 (40.86,49.91)	49.42 (44.28,54.56)	3.81 (3.31,4.32)	5.16 (4.52,5.81)	2.42 (1.45,3.39)	2.9 (1.99,3.81)	3.82 (2.85,4.78)	4.86 (3.87,5.85)	4.1 (3.32,4.88)	6.98 (5.82,8.15)
Digestive	peritonitis	7220	3541	3679	3415	1679	1736	58.7 (53.56,63.84)	77.14 (70.72,83.55)	4.92 (4.34,5.49)	6.16 (5.45,6.86)	3.23 (2.11,4.35)	3.43 (2.44,4.42)	5.16 (4.04,6.28)	4.98 (3.98,5.98)	6.31 (5.34,7.28)	7.66 (6.44,8.88)
Digestive	portal_htn	1393	455	938	625	208	417	2.12 (1.14,3.1)	5.6 (3.86,7.33)	0.84 (0.6,1.08)	2.1 (1.69,2.52)	0.6 (0.12,1.09)	0.74 (0.28,1.2)	1.27 (0.71,1.82)	1.71 (1.13,2.3)	1.57 (1.09,2.06)	1.91 (1.3,2.51)
Digestive	ulc_colitis	6523	3276	3247	3369	1724	1645	99.96 (93.27,106.65)	111.71 (104.01,119.42)	2.81 (2.37,3.24)	3.81 (3.25,4.37)	2.43 (1.46,3.4)	3.0 (2.07,3.92)	3.14 (2.26,4.01)	3.2 (2.4,4.0)	3.81 (3.06,4.57)	3.31 (2.5,4.11)
Digestive	ulcer_peptic	21297	9111	12186	11419	4793	6626	226.44 (216.43,236.45)	442.38 (427.3,457.46)	11.56 (10.67,12.46)	16.08 (14.91,17.25)	5.3 (3.86,6.75)	9.02 (7.4,10.64)	10.37 (8.76,11.98)	12.49 (10.88,14.1)	16.17 (14.59,17.75)	21.42 (19.33,23.52)
Digestive	varices	1523	590	933	740	320	420	9.9 (7.79,12.02)	9.38 (7.13,11.62)	1.21 (0.93,1.5)	2.19 (1.77,2.61)	0.5 (0.06,0.94)	0.82 (0.33,1.3)	2.22 (1.48,2.95)	1.4 (0.87,1.93)	1.84 (1.32,2.37)	2.36 (1.69,3.04)
Digestive	volvulus	1530	900	630	712	439	273	9.19 (7.15,11.23)	5.88 (4.1,7.66)	1.99 (1.62,2.35)	1.39 (1.05,1.72)	0.6 (0.12,1.09)	0.82 (0.33,1.3)	1.2 (0.66,1.74)	1.87 (1.26,2.48)	1.77 (1.26,2.28)	3.07 (2.3,3.84)
Ear	deaf	46964	21980	24984	31153	14798	16355	731.31 (713.79,748.83)	980.37 (958.57,1002.18)	54.98 (52.96,56.99)	72.92 (70.33,75.51)	26.95 (23.65, 30.25)	32.12 (29.01,35.24)	43.35 (39.97, 46.72)	59.44 (55.82,63.06)	77.69 (74.07,81.31)	108.27 (103.26,113.28)
Ear	meniere	3166	2021	1145	1977	1296	681	72.61 (66.9,78.33)	47.46 (42.42,52.5)	2.28 (1.89,2.68)	1.44 (1.1,1.78)	1.21 (0.52,1.89)	1.79 (1.07,2.5)	2.1 (1.38,2.82)	2.04 (1.4,2.68)	2.05 (1.5,2.6)	1.82 (1.23,2.42)

Ear	tinnitus	50806	24260	26546	28655	13817	14838	925.69 (906.19,945.19)	1257.98 (1233.66, 1282.3)	21.74 (20.47,23.01)	25.48 (23.94,27.02)	18.17 (15.43, 20.92)	18.94 (16.51,21.37)	25.96 (23.31, 28.61)	24.73 (22.37,27.09)	25.61 (23.53,27.68)	23.09 (20.83,25.35)
Endocrine	CF	147	122	25	125	109	16	7.54 (5.7,9.39)	1.12 (0.34,1.9)	0.07 (0.0,0.14)	0.04 (-0.02,0.1)	0.3 (-0.04,0.64)	0.0 (0.0,0.0)	0.0 (0.0,0.0)	0.05 (-0.05,0.15)	0.08 (-0.03,0.18)	0.0 (0.0,0.0)
Endocrine	PCOS	1287	1287	0	754	754	0	55.99 (50.97,61.01)	0.0 (0.0,0.0)	0.28 (0.14,0.42)	0.0 (0.0,0.0)	0.3 (-0.04,0.65)	0.15 (-0.06,0.36)	0.32 (0.04,0.6)	0.16 (-0.02,0.33)	0.0 (0.0,0.0)	0.15 (-0.02,0.32)
Endocrine	PTH	2528	1882	646	1360	1019	341	20.04 (17.03,23.05)	9.52 (7.26,11.78)	5.64 (5.02,6.26)	1.66 (1.3,2.03)	0.6 (0.12,1.09)	2.0 (1.25,2.76)	2.47 (1.7,3.25)	3.12 (2.33,3.91)	5.51 (4.6,6.41)	6.25 (5.15,7.35)
Endocrine	SIADH	582	280	302	254	116	138	0.59 (0.07,1.11)	1.26 (0.44,2.08)	0.39 (0.22,0.55)	0.72 (0.47,0.96)	0.0 (0.0,0.0)	0.0 (0.0,0.0)	0.51 (0.16,0.86)	0.36 (0.09,0.63)	0.81 (0.46,1.15)	1.0 (0.56,1.44)
Endocrine	diabetes_nos	26977	10226	16751	12005	4518	7487	281.14 (270.01,292.26)	586.01 (568.79,603.24)	4.54 (3.98,5.1)	9.04 (8.16,9.92)	2.96 (1.88,4.04)	5.16 (3.93,6.38)	6.22 (4.97,7.47)	5.74 (4.65,6.83)	8.1 (6.98,9.22)	8.46 (7.14,9.78)
Endocrine	diabetes_t1	5121	2190	2931	2388	1020	1368	41.49 (37.17,45.82)	61.88 (56.13,67.63)	2.82 (2.39,3.26)	5.53 (4.86,6.2)	3.03 (1.95,4.11)	2.83 (1.93,3.73)	2.67 (1.86,3.48)	3.81 (2.94,4.69)	4.64 (3.81,5.47)	5.96 (4.89,7.04)
Endocrine	diabetes_t2	42662	17251	25411	20336	8214	12122	187.54 (178.41,196.67)	391.7 (377.47,405.93)	36.37 (34.78,37.96)	66.54 (64.15,68.93)	20.49 (17.66, 23.32)	30.22 (27.25,33.18)	37.21 (34.15, 40.26)	49.67 (46.46,52.89)	59.97 (56.92,63.03)	76.63 (72.65,80.62)
Endocrine	hyperthyroid	8905	7080	1825	5054	4062	992	235.17 (224.97,245.36)	60.34 (54.66,66.02)	7.57 (6.85,8.3)	2.46 (2.01,2.9)	4.68 (3.33,6.03)	5.26 (4.03,6.49)	5.03 (3.91,6.14)	5.34 (4.3,6.38)	4.81 (3.96,5.66)	6.04 (4.95,7.13)
Endocrine	hypothyroid	39029	31552	7477	19351	15645	3706	935.59 (916.0,955.19)	221.05 (210.27,231.83)	28.31 (26.86,29.77)	11.11 (10.15,12.07)	14.42 (12.01, 16.82)	17.05 (14.8,19.3)	21.07 (18.74, 23.41)	20.41 (18.32,22.5)	22.75 (20.84,24.66)	20.84 (18.75,22.94)
Endocrine	obesity	50234	27810	22424	31139	17496	13643	688.76 (671.72,705.8)	581.25 (564.09,598.41)	61.4 (59.27,63.54)	64.85 (62.46,67.24)	56.57 (51.73, 61.41)	60.66 (56.34,64.98)	65.15 (60.98, 69.31)	67.21 (63.36,71.05)	62.35 (59.17,65.53)	62.89 (59.24,66.55)

Endocrine	thyroid	43851	35224	8627	22188	17798	4390	1103.92 (1082.83,1125.01)	278.45 (266.38,290.51)	28.92 (27.43,30.41)	11.31 (10.34,12.28)	16.76 (14.16, 19.37)	18.25 (15.91,20.6)	21.41 (19.04, 23.78)	19.87 (17.79,21.95)	22.82 (20.89,24.74)	20.83 (18.72,22.94)
Endocrine	thyroid_nos	5150	4263	887	4696	3875	821	280.08 (268.97,291.18)	70.0 (63.88,76.11)	1.47 (1.15,1.79)	0.7 (0.46,0.94)	1.42 (0.68,2.17)	1.66 (0.96,2.35)	1.09 (0.57,1.61)	0.85 (0.43,1.26)	1.22 (0.79,1.65)	0.72 (0.34,1.1)
Eye	ant_uveitis	2787	1478	1309	2161	1146	1015	57.64 (52.55,62.74)	71.12 (64.95,77.28)	3.11 (2.65,3.57)	2.42 (1.97,2.86)	2.02 (1.13,2.91)	2.69 (1.81,3.56)	2.99 (2.14,3.85)	2.83 (2.07,3.58)	3.33 (2.62,4.03)	2.38 (1.7,3.06)
Eye	blind	3547	1711	1836	2091	1001	1090	30.65 (26.93,34.37)	42.98 (38.18,47.78)	3.31 (2.84,3.79)	4.63 (4.02,5.25)	1.31 (0.6,2.02)	2.16 (1.37,2.94)	3.18 (2.3,4.06)	2.87 (2.11,3.63)	4.74 (3.91,5.58)	6.93 (5.77,8.08)
Eye	cataract	64203	37068	27135	30552	17592	12960	345.38 (333.09,357.67)	344.1 (330.74,357.47)	73.85 (71.55,76.14)	68.17 (65.76,70.58)	8.91 (7.05,10.78)	13.59 (11.61,15.57)	26.28 (23.73, 28.83)	52.71 (49.4,56.01)	98.23 (94.28,102.17)	172.27 (166.13,178.42)
Eye	diab_eye	13313	5066	8247	9050	3404	5646	98.9 (92.24,105.56)	216.43 (205.76,227.1)	19.67 (18.51,20.83)	40.45 (38.61,42.29)	11.77 (9.63,13.91)	17.96 (15.69,20.24)	23.72 (21.29, 26.14)	27.68 (25.31,30.06)	34.74 (32.44,37.03)	43.65 (40.69,46.61)
Eye	glaucoma	17587	8838	8749	8690	4405	4285	177.41 (168.52,186.29)	230.57 (219.56,241.58)	16.04 (14.99,17.1)	17.09 (15.9,18.28)	3.03 (1.95,4.12)	6.73 (5.34,8.12)	9.19 (7.68,10.69)	14.11 (12.41,15.81)	23.02 (21.14,24.89)	30.2 (27.73,32.67)
Eye	macula_degen	13203	7837	5366	6333	3705	2628	123.06 (115.64,130.48)	111.99 (104.28,119.71)	11.38 (10.49,12.26)	9.43 (8.55,10.3)	1.61 (0.82,2.4)	2.76 (1.87,3.65)	2.8 (1.97,3.63)	7.51 (6.28,8.74)	14.47 (12.99,15.94)	24.34 (22.14,26.54)
Eye	post_uveitis	280	153	127	244	132	112	6.6 (4.87,8.33)	7.84 (5.79,9.89)	0.4 (0.24,0.57)	0.23 (0.09,0.37)	0.4 (0.01,0.8)	0.22 (-0.03,0.47)	0.38 (0.08,0.68)	0.21 (0.0,0.41)	0.27 (0.07,0.47)	0.5 (0.19,0.81)
Eye	ptosis	3313	1999	1314	1815	1118	697	37.37 (33.26,41.47)	26.88 (23.08,30.68)	4.93 (4.35,5.5)	3.17 (2.66,3.67)	1.51 (0.75,2.27)	2.38 (1.55,3.2)	2.99 (2.13,3.84)	3.96 (3.07,4.86)	5.17 (4.29,6.04)	6.31 (5.21,7.42)
Eye	retinal_detach	7012	3030	3982	3535	1576	1959	66.13 (60.67,71.58)	101.64 (94.28,108.99)	6.09 (5.45,6.74)	8.35 (7.53,9.18)	3.13 (2.03,4.23)	4.17 (3.08,5.27)	6.05 (4.84,7.27)	9.77 (8.36,11.17)	8.21 (7.1,9.32)	8.0 (6.75,9.26)

Eye	retinal_vasc_occl	2497	1089	1408	1583	663	920	19.21 (16.27,22.16)	38.92 (34.35,43.48)	3.33 (2.85,3.8)	4.69 (4.08,5.31)	1.31 (0.6,2.02)	1.19 (0.61,1.77)	1.71 (1.07,2.36)	3.49 (2.66,4.33)	5.13 (4.26,6.0)	7.84 (6.61,9.08)
Eye	scleritis	785	517	268	735	489	246	24.64 (21.3,27.97)	15.82 (12.9,18.73)	1.2 (0.91,1.48)	0.72 (0.48,0.96)	1.31 (0.6,2.02)	1.19 (0.61,1.77)	1.27 (0.71,1.82)	0.36 (0.09,0.63)	1.04 (0.65,1.43)	0.96 (0.53,1.39)
Genitourinary	AKI	20544	8011	12533	9152	3524	5628	9.08 (7.05,11.1)	22.68 (19.19,26.17)	11.13 (10.26,12.0)	24.08 (22.68,25.48)	3.42 (2.27,4.57)	6.02 (4.71,7.33)	9.26 (7.76,10.77)	13.24 (11.61,14.86)	21.68 (19.89,23.47)	35.04 (32.43,37.65)
Genitourinary	BPH	30410	0	30410	16177	0	16177	0.0 (0.0,0.0)	603.09 (585.63,620.55)	0.0 (0.0,0.0)	90.31 (87.48,93.14)	5.34 (3.9,6.78)	12.31 (10.43,14.19)	21.54 (19.23,23.84)	37.6 (34.82,40.39)	53.81 (50.91,56.71)	73.49 (69.54,77.43)
Genitourinary	CKD	23280	11266	12014	11709	5665	6044	102.67 (95.89,109.45)	126.97 (118.76,135.18)	23.18 (21.91,24.44)	29.03 (27.48,30.58)	4.04 (2.79,5.3)	6.35 (5.0,7.7)	10.87 (9.24,12.51)	18.29 (16.36,20.21)	31.57 (29.39,33.75)	62.81 (59.26,66.36)
Genitourinary	ED	19869	0	19869	18983	0	18983	0.0 (0.0,0.0)	879.02 (858.25,899.78)	0.0 (0.0,0.0)	135.69 (132.14,139.24)	31.4 (27.88,34.92)	37.78 (34.46,41.11)	47.16 (43.7,50.62)	59.25 (55.71,62.8)	70.54 (67.18,73.89)	73.34 (69.4,77.28)
Genitourinary	ESRD	2587	963	1624	1285	484	801	15.91 (13.23,18.6)	30.94 (26.87,35.01)	1.37 (1.07,1.68)	2.53 (2.08,2.99)	0.7 (0.18,1.23)	0.67 (0.23,1.11)	1.46 (0.86,2.06)	1.51 (0.96,2.06)	2.54 (1.93,3.15)	3.22 (2.43,4.01)
Genitourinary	GN	3577	1659	1918	1762	825	937	36.31 (32.26,40.35)	47.46 (42.42,52.5)	3.56 (3.07,4.06)	4.78 (4.16,5.41)	1.71 (0.9,2.53)	1.49 (0.84,2.14)	2.42 (1.65,3.18)	3.71 (2.85,4.57)	5.09 (4.23,5.96)	7.58 (6.37,8.8)
Genitourinary	PCB	3980	3980	0	3194	3194	0	185.66 (176.57,194.74)	0.0 (0.0,0.0)	6.03 (5.38,6.67)	0.0 (0.0,0.0)	9.5 (7.57,11.43)	7.4 (5.93,8.86)	4.63 (3.56,5.7)	2.58 (1.85,3.3)	0.73 (0.4,1.06)	0.25 (0.03,0.47)
Genitourinary	PMB	19634	19634	0	12365	12365	0	628.17 (611.84,644.5)	0.0 (0.0,0.0)	40.88 (39.15,42.61)	0.0 (0.0,0.0)	6.45 (4.87,8.03)	24.44 (21.79,27.09)	42.11 (38.85,45.36)	24.82 (22.53,27.11)	16.57 (14.96,18.18)	13.65 (11.98,15.32)
Genitourinary	TIN	2561	1745	816	1141	770	371	18.62 (15.72,21.53)	8.54 (6.4,10.68)	2.39 (1.99,2.8)	1.37 (1.04,1.7)	1.31 (0.6,2.02)	1.78 (1.07,2.5)	2.09 (1.38,2.81)	1.87 (1.26,2.49)	2.08 (1.52,2.63)	2.06 (1.43,2.69)

Genitourinary	chr_cystitis	1840	1200	640	986	680	306	25.93 (22.51,29.36)	9.1 (6.89,11.31)	2.33 (1.93,2.72)	1.79 (1.41,2.17)	0.91 (0.31,1.5)	0.89 (0.39,1.39)	1.46 (0.86,2.05)	1.77 (1.17,2.36)	2.73 (2.1,3.37)	3.43 (2.61,4.24)
Genitourinary	dysmenorrhoea	5954	5954	0	4820	4820	0	326.99 (315.02,338.96)	0.0 (0.0,0.0)	3.33 (2.85,3.81)	0.0 (0.0,0.0)	9.96 (7.96,11.95)	4.6 (3.44,5.77)	1.3 (0.73,1.87)	0.37 (0.1,0.64)	0.0 (0.0,0.0)	0.0 (0.0,0.0)
Genitourinary	endometrial_hyper	3576	3576	0	1686	1686	0	90.06 (83.7,96.42)	0.0 (0.0,0.0)	4.6 (4.04,5.16)	0.0 (0.0,0.0)	3.94 (2.7,5.17)	4.55 (3.41,5.69)	2.93 (2.08,3.78)	2.2 (1.53,2.86)	1.43 (0.97,1.89)	1.71 (1.14,2.29)
Genitourinary	endometriosis	10023	10023	0	5488	5488	0	360.82 (348.27,373.37)	0.0 (0.0,0.0)	5.84 (5.2,6.48)	0.0 (0.0,0.0)	8.38 (6.56,10.21)	6.26 (4.9,7.61)	3.45 (2.52,4.38)	1.7 (1.11,2.28)	1.56 (1.08,2.04)	1.62 (1.06,2.19)
Genitourinary	female_genital_prolapse	24004	24004	0	13846	13846	0	616.26 (600.08,632.44)	0.0 (0.0,0.0)	54.02 (52.03,56.01)	0.0 (0.0,0.0)	16.01 (13.5,18.51)	19.95 (17.54,22.36)	24.69 (22.2,27.18)	26.63 (24.27,28.99)	34.38 (32.06,36.7)	37.43 (34.66,40.21)
Genitourinary	female_infertility	3907	3907	0	2884	2884	0	224.32 (214.36,234.29)	0.0 (0.0,0.0)	0.31 (0.16,0.45)	0.0 (0.0,0.0)	1.24 (0.54,1.94)	0.3 (0.01,0.6)	0.0 (0.0,0.0)	0.0 (0.0,0.0)	0.04 (-0.04,0.11)	0.0 (0.0,0.0)
Genitourinary	hydrocele	3570	0	3570	2305	0	2305	0.0 (0.0,0.0)	129.63 (121.34,137.93)	0.0 (0.0,0.0)	9.41 (8.53,10.29)	1.52 (0.75,2.28)	3.13 (2.18,4.08)	3.31 (2.41,4.21)	4.07 (3.17,4.98)	5.07 (4.21,5.94)	6.18 (5.08,7.28)
Genitourinary	male_infertility	716	0	716	661	0	661	0.0 (0.0,0.0)	57.12 (51.59,62.64)	0.0 (0.0,0.0)	0.53 (0.32,0.74)	1.41 (0.67,2.15)	0.52 (0.13,0.91)	0.19 (-0.03,0.41)	0.05 (-0.05,0.15)	0.0 (0.0,0.0)	0.0 (0.0,0.0)
Genitourinary	menorrhagia	29009	29009	0	20677	20677	0	1358.18 (1335.13,1381.24)	0.0 (0.0,0.0)	31.33 (29.76,32.91)	0.0 (0.0,0.0)	73.4 (67.8,79.0)	53.4 (49.25,57.55)	14.61 (12.61,16.62)	0.97 (0.51,1.43)	0.08 (-0.03,0.2)	0.1 (-0.04,0.25)
Genitourinary	neuro_bladder	5707	3472	2235	4588	2803	1785	118.11 (110.84,125.38)	95.34 (88.21,102.46)	12.14 (11.22,13.05)	9.65 (8.77,10.54)	5.26 (3.83,6.69)	7.71 (6.22,9.2)	8.72 (7.26,10.19)	11.75 (10.21,13.29)	12.22 (10.86,13.57)	15.67 (13.92,17.43)
Genitourinary	obstr_reflux	7049	2907	4142	3452	1462	1990	33.01 (29.15,36.87)	46.2 (41.22,51.17)	5.57 (4.96,6.19)	9.74 (8.85,10.63)	5.85 (4.34,7.35)	4.32 (3.21,5.43)	5.98 (4.77,7.19)	5.59 (4.53,6.64)	9.31 (8.13,10.48)	11.02 (9.55,12.48)

Genitourinary	undescended_testis	577	0	577	399	0	399	0.0 (0.0,0.0)	36.68 (32.25,41.11)	0.0 (0.0,0.0)	0.13 (0.03,0.23)	0.1 (-0.1,0.3)	0.0 (0.0,0.0)	0.06 (-0.06, 0.19)	0.05 (-0.05,0.15)	0.08 (-0.03,0.18)	0.05 (-0.05,0.15)
Genitourinary	urine_incont	21841	18605	3236	14897	12855	2042	600.82 (584.83,616.81)	80.78 (74.21,87.34)	49.2 (47.3,51.09)	12.75 (11.73,13.77)	25.79 (22.6,28.99)	27.49 (24.64,30.34)	27.84 (25.17, 30.5)	32.88 (30.25,35.5)	33.24 (30.97,35.52)	39.17 (36.34,42.0)
Genitourinary	urolithiasis	15901	5123	10778	8988	2938	6050	133.44 (125.72,141.16)	373.36 (359.46,387.27)	8.53 (7.77,9.3)	18.67 (17.41,19.92)	11.05 (8.97,13.14)	11.19 (9.39,13.0)	11.69 (9.98,13.39)	13.79 (12.11,15.48)	13.29 (11.86,14.71)	15.52 (13.76,17.28)
Haematological or immunological	2ry_polycythaemia	817	257	560	396	128	268	2.48 (1.42,3.53)	6.44 (4.58,8.3)	0.4 (0.24,0.57)	0.97 (0.69,1.25)	0.4 (0.01,0.8)	0.52 (0.13,0.9)	0.57 (0.2,0.94)	0.52 (0.2,0.84)	0.69 (0.37,1.01)	1.05 (0.6,1.51)
Haematological or immunological	IDA	27327	17455	9872	14454	9714	4740	387.46 (374.48,400.45)	109.89 (102.25,117.54)	27.73 (26.33,29.13)	23.7 (22.3,25.1)	20.66 (17.79, 23.53)	23.01 (20.4,25.62)	19.48 (17.26, 21.7)	21.19 (19.1,23.28)	26.88 (24.86,28.91)	38.63 (35.85,41.4)
Haematological or immunological	agranulocytosis	8320	4972	3348	4471	2772	1699	77.68 (71.77,83.59)	38.78 (34.22,43.34)	13.29 (12.33,14.24)	10.18 (9.27,11.09)	6.57 (4.97,8.17)	7.99 (6.48,9.51)	11.17 (9.51,12.82)	13.48 (11.83,15.12)	13.91 (12.47,15.35)	13.48 (11.86,15.1)
Haematological or immunological	aplastic	1390	580	810	669	286	383	3.77 (2.47,5.08)	4.62 (3.04,6.2)	0.72 (0.5,0.94)	1.94 (1.54,2.33)	0.6 (0.12,1.09)	0.22 (-0.03,0.47)	0.89 (0.42,1.35)	1.09 (0.62,1.56)	1.84 (1.32,2.36)	2.06 (1.43,2.69)
Haematological or immunological	b12_def	5674	3441	2233	4191	2441	1750	100.67 (93.95,107.38)	68.18 (62.14,74.21)	9.93 (9.11,10.76)	10.9 (9.96,11.84)	8.1 (6.33,9.88)	7.78 (6.29,9.28)	8.25 (6.82,9.67)	9.78 (8.38,11.19)	11.04 (9.75,12.32)	14.69 (12.99,16.39)
Haematological or immunological	folatedef	1435	715	720	1069	562	507	13.32 (10.87,15.77)	9.1 (6.89,11.31)	3.06 (2.61,3.52)	3.75 (3.2,4.3)	3.63 (2.44,4.81)	2.82 (1.92,3.72)	2.47 (1.7,3.25)	2.55 (1.84,3.26)	3.69 (2.95,4.43)	4.73 (3.77,5.68)

Haematological or immunological	hyposplenism	1227	580	647	659	327	332	16.03 (13.34,18.72)	21.42 (18.03,24.81)	0.62 (0.41,0.82)	0.7 (0.46,0.93)	0.3 (-0.04,0.64)	0.52 (0.13,0.9)	0.38 (0.08,0.68)	0.78 (0.39,1.18)	0.62 (0.31,0.92)	1.06 (0.6,1.51)
Haematological or immunological	immunodef	567	296	271	266	146	120	3.18 (1.98,4.38)	2.38 (1.25,3.51)	0.6 (0.4,0.8)	0.69 (0.46,0.93)	0.2 (-0.08,0.48)	0.82 (0.33,1.3)	0.38 (0.08,0.68)	0.52 (0.2,0.84)	0.92 (0.55,1.29)	0.7 (0.33,1.07)
Haematological or immunological	oth_anaemia	35331	21104	14227	19693	12420	7273	514.53 (499.67,529.4)	198.23 (188.01,208.45)	37.73 (36.08,39.38)	35.92 (34.19,37.65)	26.12 (22.88, 29.37)	27.43 (24.56,30.29)	24.94 (22.41, 27.46)	29.59 (27.1,32.07)	42.65 (40.08,45.22)	57.87 (54.44,61.31)
Haematological or immunological	oth_haem_anaemia	641	338	303	359	189	170	7.66 (5.8,9.52)	8.12 (6.03,10.21)	0.62 (0.41,0.82)	0.65 (0.42,0.88)	0.1 (-0.1,0.3)	0.59 (0.18,1.0)	0.38 (0.08,0.68)	0.57 (0.23,0.91)	0.65 (0.34,0.96)	1.16 (0.68,1.63)
Haematological or immunological	pri_thrombocytopaenia	1604	820	784	1138	583	555	27.94 (24.39,31.49)	23.94 (20.36,27.52)	1.87 (1.51,2.22)	3.14 (2.64,3.65)	1.01 (0.38,1.63)	1.41 (0.78,2.05)	1.78 (1.12,2.44)	2.5 (1.79,3.21)	2.96 (2.3,3.63)	3.68 (2.84,4.52)
Haematological or immunological	sarcoid	2187	1176	1011	1092	590	502	34.89 (30.92,38.86)	38.22 (33.69,42.74)	1.13 (0.85,1.4)	0.95 (0.67,1.23)	1.31 (0.6,2.02)	1.34 (0.72,1.96)	0.95 (0.47,1.43)	0.73 (0.35,1.11)	1.08 (0.68,1.48)	1.06 (0.61,1.51)
Haematological or immunological	sec_oth_thrombocytopaenia	4315	1855	2460	2232	992	1240	25.7 (22.29,29.1)	26.88 (23.08,30.68)	3.96 (3.45,4.48)	6.44 (5.72,7.16)	2.12 (1.21,3.02)	3.05 (2.11,3.98)	3.49 (2.57,4.41)	5.11 (4.1,6.12)	6.24 (5.28,7.21)	7.72 (6.49,8.94)
Haematological or immunological	sickle_cell	201	129	72	73	49	24	2.71 (1.6,3.82)	1.54 (0.63,2.45)	0.09 (0.01,0.16)	0.11 (0.01,0.2)	0.2 (-0.08,0.48)	0.22 (-0.03,0.47)	0.13 (-0.05, 0.3)	0.16 (-0.02,0.33)	0.0 (0.0,0.0)	0.0 (0.0,0.0)

Haematological or immunological	sickle_trait	584	415	169	234	168	66	8.25 (6.32,10.18)	3.92 (2.47,5.37)	0.6 (0.4,0.8)	0.32 (0.16,0.48)	0.5 (0.06,0.94)	1.04 (0.49,1.58)	0.44 (0.11,0.77)	0.47 (0.16,0.77)	0.38 (0.15,0.62)	0.2 (0.0,0.4)
Haematological or immunological	splenomegaly	1232	411	821	588	198	390	3.65 (2.37,4.94)	10.08 (7.75,12.41)	0.69 (0.47,0.9)	1.9 (1.5,2.29)	0.9 (0.31,1.5)	0.44 (0.09,0.8)	0.82 (0.38,1.27)	1.04 (0.58,1.5)	1.73 (1.22,2.23)	1.81 (1.22,2.4)
Haematological or immunological	thal_trait	473	288	185	290	168	122	10.73 (8.52,12.93)	8.82 (6.64,11.0)	0.33 (0.18,0.48)	0.4 (0.22,0.58)	0.5 (0.06,0.95)	0.59 (0.18,1.01)	0.63 (0.24,1.03)	0.31 (0.06,0.56)	0.19 (0.02,0.36)	0.2 (0.0,0.4)
Haematological or immunological	thala	409	259	150	196	124	72	6.6 (4.87,8.33)	4.2 (2.7,5.7)	0.11 (0.02,0.19)	0.15 (0.04,0.26)	0.1 (-0.1,0.3)	0.0 (0.0,0.0)	0.25 (0.01,0.5)	0.16 (-0.02,0.33)	0.12 (-0.02,0.25)	0.1 (-0.04,0.24)
Haematological or immunological	thrombophilia	1282	841	441	919	608	311	26.64 (23.17,30.11)	16.1 (13.16,19.04)	1.78 (1.43,2.13)	1.54 (1.19,1.89)	1.82 (0.98,2.65)	2.16 (1.37,2.94)	2.03 (1.33,2.73)	1.72 (1.13,2.3)	1.5 (1.03,1.97)	1.16 (0.68,1.63)
Infections	PID	6724	6724	0	2914	2914	0	137.44 (129.61,145.28)	0.0 (0.0,0.0)	7.5 (6.78,8.21)	0.0 (0.0,0.0)	7.85 (6.1,9.61)	5.49 (4.23,6.75)	4.1 (3.1,5.11)	2.51 (1.8,3.22)	3.32 (2.62,4.02)	3.63 (2.79,4.47)
Infections	TB	4119	2179	1940	2516	1342	1174	96.78 (90.19,103.37)	103.88 (96.44,111.31)	0.67 (0.46,0.89)	1.19 (0.88,1.5)	1.11 (0.45,1.77)	0.82 (0.34,1.3)	0.7 (0.29,1.11)	0.79 (0.39,1.19)	1.17 (0.75,1.58)	0.82 (0.42,1.22)
Infections	anorectal	2362	837	1525	1100	387	713	17.56 (14.75,20.38)	39.06 (34.48,43.63)	1.27 (0.97,1.56)	2.47 (2.02,2.92)	3.13 (2.03,4.23)	2.53 (1.68,3.38)	1.91 (1.22,2.59)	1.15 (0.67,1.63)	1.58 (1.09,2.06)	1.56 (1.01,2.11)
Infections	bacterial	80401	42601	37800	37324	19807	17517	679.8 (662.86,696.74)	668.33 (650.01,686.64)	67.55 (65.31,69.78)	78.38 (75.74,81.01)	46.66 (42.26,51.06)	46.44 (42.66,50.22)	59.49 (55.53,63.45)	65.46 (61.68,69.23)	81.77 (78.13,85.4)	109.38 (104.52,114.24)

Infections	bone	3204	1239	1965	1458	530	928	19.21 (16.27,22.16)	35.0 (30.67,39.33)	1.27 (0.97,1.56)	3.44 (2.91,3.97)	1.01 (0.38,1.63)	1.26 (0.66,1.86)	2.29 (1.54,3.03)	1.98 (1.35,2.61)	2.46 (1.86,3.07)	3.53 (2.7,4.35)
Infections	chr_hep	1361	571	790	848	407	441	26.17 (22.73,29.61)	31.08 (27.0,35.16)	0.55 (0.35,0.74)	1.1 (0.8,1.4)	1.21 (0.53,1.9)	0.89 (0.39,1.4)	1.14 (0.62,1.67)	0.83 (0.43,1.24)	0.73 (0.4,1.06)	0.3 (0.06,0.54)
Infections	digestive	29101	16190	12911	13742	7687	6055	87.82 (81.54,94.1)	86.94 (80.13,93.74)	32.06 (30.57,33.54)	32.03 (30.41,33.65)	18.79 (16.08,21.49)	21.51 (19.02,24.0)	28.93 (26.26,31.6)	31.82 (29.28,34.35)	34.9 (32.61,37.18)	44.88 (41.91,47.86)
Infections	ear_urti	17601	9781	7820	8655	4830	3825	262.51 (251.75,273.27)	254.79 (243.23,266.34)	9.3 (8.5,10.11)	9.19 (8.31,10.06)	10.45 (8.41,12.49)	7.77 (6.26,9.27)	8.93 (7.43,10.42)	9.58 (8.18,10.99)	9.28 (8.1,10.47)	9.55 (8.17,10.92)
Infections	enceph	530	269	261	230	112	118	6.13 (4.46,7.8)	6.44 (4.58,8.3)	0.12 (0.03,0.21)	0.23 (0.09,0.37)	0.0 (0.0,0.0)	0.15 (-0.06,0.35)	0.25 (0.01,0.5)	0.26 (0.03,0.49)	0.15 (0.0,0.3)	0.15 (-0.02,0.32)
Infections	eye	1864	973	891	851	464	387	16.62 (13.88,19.36)	14.98 (12.14,17.82)	1.06 (0.79,1.32)	1.35 (1.02,1.68)	0.6 (0.12,1.09)	0.67 (0.23,1.1)	1.14 (0.61,1.67)	0.99 (0.54,1.43)	1.5 (1.03,1.97)	1.66 (1.09,2.23)
Infections	heart	648	180	468	286	78	208	1.53 (0.7,2.37)	5.46 (3.75,7.17)	0.21 (0.09,0.33)	0.93 (0.65,1.2)	0.2 (-0.08,0.48)	0.07 (-0.07,0.22)	0.25 (0.01,0.5)	0.36 (0.09,0.63)	1.0 (0.61,1.38)	0.8 (0.41,1.2)
Infections	hiv	567	93	474	238	37	201	1.89 (0.96,2.81)	15.54 (12.65,18.43)	0.07 (0.0,0.14)	0.51 (0.3,0.71)	0.6 (0.12,1.09)	0.45 (0.09,0.8)	0.38 (0.08,0.68)	0.26 (0.03,0.49)	0.12 (-0.02,0.25)	0.1 (-0.04,0.24)
Infections	liver	3388	1515	1873	1431	673	758	38.9 (34.71,43.09)	47.46 (42.42,52.5)	0.9 (0.65,1.15)	1.33 (1.0,1.66)	0.71 (0.18,1.23)	0.89 (0.39,1.4)	1.53 (0.92,2.14)	1.31 (0.79,1.82)	0.85 (0.49,1.2)	1.21 (0.73,1.69)
Infections	lrti	51771	24291	27480	23416	11039	12377	280.19 (269.09,291.3)	347.18 (333.76,360.61)	35.15 (33.59,36.72)	51.8 (49.71,53.89)	17.73 (15.08,20.38)	19.3 (16.93,21.68)	26.49 (23.91,29.07)	38.39 (35.56,41.21)	52.09 (49.26,54.93)	76.94 (72.98,80.91)
Infections	male_GU	2520	0	2520	1127	0	1127	0.0 (0.0,0.0)	40.04 (35.41,44.67)	0.0 (0.0,0.0)	4.89 (4.26,5.52)	1.71 (0.9,2.53)	1.63 (0.95,2.32)	1.52 (0.91,2.13)	2.03 (1.39,2.67)	2.58 (1.96,3.19)	3.12 (2.34,3.9)

Infections	meningitis	2449	1285	1164	1092	559	533	37.13 (33.04,41.22)	45.64 (40.69,50.58)	0.37 (0.21,0.53)	0.4 (0.22,0.58)	0.4 (0.01,0.8)	0.45 (0.09,0.8)	0.19 (-0.03,0.41)	0.21 (0.0,0.41)	0.5 (0.23,0.77)	0.5 (0.19,0.82)
Infections	mycoses	7665	4046	3619	3431	1841	1590	20.39 (17.36,23.43)	18.9 (15.71,22.08)	6.91 (6.22,7.59)	7.62 (6.83,8.4)	3.53 (2.36,4.7)	3.19 (2.24,4.15)	5.39 (4.25,6.54)	5.99 (4.89,7.08)	9.01 (7.86,10.17)	12.15 (10.62,13.68)
Infections	oth_gu	1242	1111	131	616	554	62	23.34 (20.09,26.59)	0.7 (0.09,1.31)	1.25 (0.96,1.54)	0.21 (0.08,0.34)	1.01 (0.38,1.63)	0.97 (0.44,1.49)	0.82 (0.38,1.27)	0.62 (0.27,0.98)	0.61 (0.31,0.92)	0.85 (0.45,1.26)
Infections	oth_nerv_sys	1522	768	754	650	337	313	8.61 (6.63,10.58)	10.08 (7.75,12.41)	1.18 (0.9,1.46)	1.31 (0.98,1.63)	0.1 (-0.1,0.3)	1.11 (0.55,1.68)	1.27 (0.71,1.82)	1.09 (0.62,1.56)	1.69 (1.19,2.19)	1.41 (0.89,1.93)
Infections	oth_organisms	73703	36808	36895	34086	17226	16860	270.65 (259.73,281.57)	309.67 (296.96,322.37)	73.56 (71.28,75.84)	88.57 (85.83,91.32)	43.18 (39.02,47.34)	46.43 (42.73,50.13)	60.18 (56.28,64.08)	75.81 (71.84,79.79)	94.24 (90.41,98.07)	125.7 (120.62,130.78)
Infections	oth_organs	38595	19624	18971	17637	9020	8617	197.8 (188.43,207.17)	222.73 (211.91,233.55)	31.97 (30.48,33.46)	38.99 (37.18,40.79)	19.44 (16.67,22.21)	20.41 (17.97,22.85)	28.65 (25.97,31.32)	33.07 (30.47,35.68)	40.2 (37.73,42.68)	53.9 (50.62,57.19)
Infections	parasitic	1685	706	979	721	304	417	14.03 (11.51,16.55)	24.22 (20.61,27.82)	0.56 (0.37,0.76)	0.91 (0.64,1.18)	0.81 (0.25,1.36)	0.59 (0.18,1.01)	1.01 (0.52,1.51)	0.36 (0.09,0.63)	0.81 (0.46,1.15)	0.75 (0.37,1.14)
Infections	rh_fever	4029	2230	1799	1995	1140	855	61.53 (56.27,66.79)	44.24 (39.37,49.1)	1.45 (1.14,1.76)	1.8 (1.41,2.18)	0.7 (0.18,1.23)	0.59 (0.18,1.01)	0.76 (0.33,1.19)	1.31 (0.79,1.82)	1.82 (1.3,2.33)	3.45 (2.63,4.27)
Infections	sepsis	15510	6800	8710	6985	3079	3906	26.76 (23.28,30.23)	38.22 (33.69,42.74)	11.17 (10.3,12.04)	15.78 (14.65,16.92)	4.84 (3.47,6.21)	5.06 (3.85,6.26)	9.65 (8.12,11.19)	12.99 (11.38,14.6)	16.95 (15.37,18.54)	21.38 (19.34,23.42)
Infections	skin	22950	10419	12531	10732	4879	5853	117.41 (110.16,124.65)	190.25 (180.23,200.27)	16.95 (15.87,18.02)	26.45 (24.97,27.93)	15.34 (12.89,17.8)	17.29 (15.05,19.53)	18.66 (16.51,20.82)	20.6 (18.55,22.65)	23.48 (21.6,25.36)	26.67 (24.37,28.96)
Infections	uti	30468	17228	13240	14386	8190	6196	195.91 (186.59,205.24)	135.93 (127.44,144.43)	29.86 (28.42,31.3)	30.31 (28.73,31.89)	11.31 (9.21,13.42)	13.73 (11.73,15.72)	21.14 (18.85,23.44)	24.78 (22.53,27.03)	36.82 (34.46,39.18)	54.35 (51.05,57.64)

Infections	viral	29412	15746	13666	13891	7484	6407	391.94 (378.88,405.0)	399.4 (385.04,413.76)	10.49 (9.63,11.35)	11.64 (10.65,12.63)	7.68 (5.92,9.44)	9.1 (7.45,10.75)	10.86 (9.19,12.52)	11.66 (10.1,13.22)	11.19 (9.88,12.5)	13.2 (11.58,14.82)
Musculoskeletal	GCA	1311	932	379	710	508	202	9.78 (7.68,11.89)	5.18 (3.51,6.85)	2.39 (1.99,2.79)	1.22 (0.91,1.54)	0.2 (-0.08,0.48)	0.59 (0.18,1.0)	0.76 (0.33,1.19)	1.09 (0.62,1.56)	2.46 (1.86,3.06)	4.38 (3.46,5.3)
Musculoskeletal	OA	126211	75084	51127	64711	38491	26220	1691.42 (1666.19,1716.65)	1367.59 (1342.4,1392.79)	148.21 (144.66,151.77)	122.46 (119.01,125.92)	49.05 (44.6,53.51)	75.81 (70.97,80.65)	117.12 (111.41,122.84)	145.03 (139.04,151.03)	174.7 (168.82,180.58)	205.78 (198.18,213.37)
Musculoskeletal	PMR	5458	3553	1905	2959	1937	1022	47.62 (42.99,52.26)	23.8 (20.23,27.37)	9.68 (8.87,10.49)	7.35 (6.58,8.12)	0.7 (0.18,1.22)	1.71 (1.01,2.4)	4.7 (3.63,5.77)	6.73 (5.57,7.89)	10.91 (9.64,12.18)	19.35 (17.4,21.29)
Musculoskeletal	PSA	2256	1172	1084	1230	644	586	32.3 (28.48,36.12)	39.62 (35.01,44.22)	1.85 (1.5,2.21)	1.9 (1.51,2.29)	2.62 (1.61,3.63)	2.31 (1.49,3.12)	2.16 (1.44,2.89)	2.3 (1.62,2.97)	1.39 (0.93,1.84)	1.21 (0.73,1.69)
Musculoskeletal	RhA	12886	8697	4189	6376	4268	2108	194.97 (185.66,204.27)	111.43 (103.74,119.13)	10.35 (9.5,11.19)	6.82 (6.07,7.57)	3.35 (2.21,4.49)	6.6 (5.22,7.98)	7.07 (5.75,8.39)	9.32 (7.95,10.7)	9.64 (8.44,10.85)	12.5 (10.93,14.07)
Musculoskeletal	SLE	1338	1109	229	728	599	129	38.19 (34.04,42.34)	7.42 (5.42,9.42)	1.16 (0.88,1.44)	0.42 (0.24,0.61)	0.81 (0.25,1.36)	0.67 (0.23,1.11)	0.51 (0.16,0.86)	0.52 (0.2,0.84)	0.85 (0.49,1.2)	1.46 (0.93,1.99)
Musculoskeletal	ank_spond	2245	885	1360	1158	453	705	27.58 (24.05,31.11)	56.84 (51.32,62.35)	0.63 (0.43,0.84)	0.87 (0.6,1.13)	0.4 (0.01,0.8)	0.37 (0.05,0.7)	0.7 (0.29,1.11)	0.94 (0.51,1.37)	0.77 (0.43,1.11)	0.96 (0.53,1.39)
Musculoskeletal	carpal_tunnel	25353	17475	7878	15665	10870	4795	532.45 (517.34,547.56)	239.67 (228.45,250.88)	35.05 (33.46,36.64)	21.42 (20.09,22.76)	21.67 (18.74,24.6)	30.45 (27.44,33.45)	34.15 (31.2,37.1)	29.67 (27.17,32.17)	26.55 (24.51,28.59)	28.77 (26.35,31.19)
Musculoskeletal	collapsed_vert	2601	1449	1152	1354	740	614	16.15 (13.45,18.85)	20.3 (17.0,23.6)	2.89 (2.44,3.33)	2.85 (2.37,3.33)	0.3 (-0.04,0.64)	1.04 (0.49,1.58)	1.58 (0.96,2.21)	2.55 (1.84,3.26)	3.39 (2.68,4.09)	6.04 (4.96,7.12)

Musculoskeletal	entero_arthro	90	61	29	39	25	14	1.06 (0.37,1.75)	0.7 (0.09,1.31)	0.05 (-0.01,0.11)	0.06 (-0.01,0.13)	0.1 (-0.1,0.3)	0.07 (-0.07,0.22)	0.0 (0.0,0.0)	0.0 (0.0,0.0)	0.08 (-0.03,0.18)	0.1 (-0.04,0.24)
Musculoskeletal	enthesopathy	84711	45885	38826	67660	36230	31430	1689.42 (1664.2,1714.63)	1834.33 (1805.95,1862.71)	166.28 (162.5,170.07)	179.76 (175.41,184.12)	168.02 (159.28,176.75)	187.49 (179.38,195.59)	179.47 (172.03,186.91)	172.55 (165.82,179.28)	170.29 (164.5,176.09)	160.23 (153.82,166.64)
Musculoskeletal	fibromatosis	9774	3092	6682	6347	2125	4222	69.9 (64.29,75.51)	188.15 (178.19,198.12)	10.97 (10.1,11.83)	23.95 (22.54,25.36)	4.54 (3.21,5.86)	6.64 (5.26,8.01)	12.91 (11.13,14.69)	18.23 (16.31,20.16)	23.09 (21.22,24.96)	23.75 (21.57,25.92)
Musculoskeletal	fracture_hip	5863	3665	2198	2846	1738	1108	24.4 (21.08,27.72)	35.98 (31.59,40.37)	6.21 (5.56,6.86)	3.93 (3.36,4.49)	1.81 (0.97,2.65)	2.01 (1.25,2.76)	2.22 (1.49,2.96)	3.7 (2.84,4.56)	6.21 (5.25,7.17)	11.42 (9.93,12.91)
Musculoskeletal	fracture_wrist	15317	10732	4585	9229	6442	2787	251.9 (241.36,262.45)	208.31 (197.84,218.78)	25.03 (23.71,26.35)	6.0 (5.3,6.71)	7.18 (5.5,8.86)	7.72 (6.22,9.22)	16.29 (14.28,18.31)	18.11 (16.18,20.03)	19.28 (17.56,20.99)	21.27 (19.21,23.34)
Musculoskeletal	gout	19778	3251	16527	12383	2218	10165	65.07 (59.66,70.48)	573.13 (556.09,590.18)	10.92 (10.06,11.79)	46.17 (44.17,48.18)	14.48 (12.1,16.86)	16.62 (14.42,18.82)	19.65 (17.43,21.86)	24.87 (22.6,27.14)	32.9 (30.64,35.15)	38.04 (35.25,40.83)
Musculoskeletal	intervert_disc	32059	17207	14852	18140	9716	8424	431.08 (417.41,444.74)	512.1 (495.93,528.26)	27.65 (26.25,29.05)	27.22 (25.69,28.75)	23.53 (20.46,26.61)	24.65 (21.93,27.37)	24.66 (22.14,27.17)	27.55 (25.14,29.97)	30.06 (27.89,32.23)	30.13 (27.64,32.62)
Musculoskeletal	juv_arth	109	77	32	67	46	21	2.24 (1.23,3.25)	1.82 (0.83,2.81)	0.12 (0.03,0.21)	0.06 (-0.01,0.13)	0.0 (0.0,0.0)	0.22 (-0.03,0.47)	0.13 (-0.05,0.3)	0.05 (-0.05,0.15)	0.0 (0.0,0.0)	0.2 (0.0,0.4)
Musculoskeletal	osteoporosis	28657	23997	4660	15817	13342	2475	447.93 (434.01,461.85)	93.8 (86.73,100.86)	69.25 (67.01,71.49)	12.55 (11.53,13.56)	5.66 (4.18,7.14)	15.15 (13.06,17.24)	30.04 (27.31,32.77)	45.02 (41.96,48.08)	57.02 (54.04,60.01)	71.61 (67.74,75.48)
Musculoskeletal	reactive	390	159	231	299	120	179	6.13 (4.46,7.8)	12.74 (10.12,15.36)	0.21 (0.09,0.33)	0.46 (0.27,0.66)	0.0 (0.0,0.0)	0.3 (0.01,0.59)	0.51 (0.16,0.86)	0.36 (0.09,0.63)	0.42 (0.17,0.67)	0.2 (0.0,0.4)

Musculoskeletal	scoliosis	4249	3062	1187	2381	1667	714	59.41 (54.24,64.58)	39.06 (34.48,43.63)	4.63 (4.07,5.2)	1.88 (1.49,2.27)	1.31 (0.6,2.03)	1.86 (1.13,2.59)	1.97 (1.28,2.67)	2.82 (2.07,3.57)	4.52 (3.7,5.34)	5.61 (4.56,6.65)
Musculoskeletal	sjogren	1487	1326	161	755	670	85	27.47 (23.94,30.99)	2.38 (1.25,3.51)	1.74 (1.4,2.09)	0.36 (0.19,0.53)	0.7 (0.18,1.23)	0.74 (0.28,1.2)	1.33 (0.76,1.9)	1.2 (0.71,1.69)	1.04 (0.65,1.43)	1.41 (0.89,1.93)
Musculoskeletal	spinal_stenosis	10203	5427	4776	4818	2546	2272	41.26 (36.94,45.57)	52.92 (47.6,58.24)	11.96 (11.06,12.86)	13.22 (12.18,14.26)	3.73 (2.53,4.93)	4.54 (3.4,5.67)	7.05 (5.74,8.36)	11.77 (10.24,13.31)	15.68 (14.15,17.21)	23.48 (21.34,25.62)
Musculoskeletal	spondylolisthesis	4406	2850	1556	2293	1472	821	47.03 (42.43,51.64)	39.34 (34.75,43.93)	5.37 (4.77,5.98)	3.02 (2.53,3.52)	0.81 (0.25,1.36)	1.94 (1.19,2.68)	3.69 (2.74,4.63)	3.86 (2.98,4.74)	5.56 (4.66,6.47)	6.93 (5.77,8.08)
Musculoskeletal	spondylosis	33839	20217	13622	21976	13036	8940	600.58 (584.6,616.57)	503.56 (487.52,519.59)	39.86 (38.16,41.57)	32.55 (30.88,34.23)	16.79 (14.22, 19.36)	24.53 (21.85,27.21)	28.87 (26.16, 31.58)	34.31 (31.6,37.02)	44.63 (41.94,47.32)	54.08 (50.66,57.51)
Musculoskeletal	sys_sclerosis	486	404	82	253	211	42	7.66 (5.8,9.52)	2.24 (1.14,3.34)	0.86 (0.62,1.1)	0.21 (0.08,0.34)	0.3 (-0.04,0.64)	0.59 (0.18,1.0)	0.25 (0.01,0.5)	0.52 (0.2,0.84)	0.92 (0.55,1.29)	0.5 (0.19,0.81)
Neurological	MND	670	295	375	303	137	166	1.53 (0.7,2.37)	3.22 (1.9,4.54)	0.51 (0.32,0.69)	0.93 (0.65,1.2)	0.1 (-0.1,0.3)	0.37 (0.05,0.7)	0.38 (0.08,0.68)	0.57 (0.23,0.91)	0.65 (0.34,0.96)	1.66 (1.09,2.22)
Neurological	MS	2512	1815	697	1246	904	342	57.17 (52.1,62.24)	24.22 (20.61,27.82)	1.48 (1.17,1.8)	1.01 (0.73,1.3)	2.83 (1.78,3.87)	1.72 (1.01,2.42)	1.59 (0.97,2.22)	1.1 (0.63,1.56)	0.73 (0.4,1.06)	0.81 (0.41,1.2)
Neurological	Parkinsons	3812	1431	2381	1792	685	1107	12.97 (10.54,15.39)	24.78 (21.13,28.42)	3.41 (2.93,3.89)	6.06 (5.36,6.76)	0.4 (0.01,0.8)	1.04 (0.49,1.58)	1.14 (0.61,1.67)	4.48 (3.53,5.43)	5.78 (4.85,6.7)	10.56 (9.13,11.99)
Neurological	autonomic_neuro	1454	747	707	828	442	386	17.68 (14.85,20.51)	16.24 (13.29,19.19)	1.88 (1.53,2.24)	1.77 (1.39,2.15)	1.21 (0.52,1.89)	1.04 (0.49,1.58)	1.65 (1.02,2.28)	2.13 (1.48,2.79)	2.08 (1.52,2.63)	2.21 (1.56,2.87)
Neurological	bells	3173	1679	1494	2118	1121	997	58.11 (53.0,63.23)	56.84 (51.32,62.35)	2.72 (2.29,3.15)	3.81 (3.25,4.37)	3.14 (2.03,4.24)	3.5 (2.5,4.5)	2.8 (1.97,3.63)	3.03 (2.25,3.81)	3.17 (2.48,3.86)	3.64 (2.8,4.48)

Neurological	cerebral_palsy	327	136	191	192	74	118	5.07 (3.55,6.58)	8.96 (6.77,11.15)	0.12 (0.03,0.21)	0.08 (0.0,0.17)	0.1 (-0.1,0.3)	0.3 (0.01,0.59)	0.0 (0.0,0.0)	0.05 (-0.05,0.15)	0.04 (-0.04,0.11)	0.2 (0.0,0.4)
Neurological	chronic_fatigue	10456	8184	2272	7221	5551	1670	261.92 (251.18,272.67)	110.87 (103.2,118.55)	12.22 (11.3,13.15)	3.51 (2.98,4.05)	9.94 (7.97,11.92)	12.51 (10.6,14.42)	10.91 (9.26,12.56)	8.48 (7.16,9.79)	6.16 (5.2,7.12)	4.8 (3.83,5.77)
Neurological	dm_neuro	2014	679	1335	1033	340	693	8.61 (6.63,10.58)	24.92 (21.26,28.58)	1.41 (1.1,1.71)	2.97 (2.48,3.47)	0.5 (0.06,0.94)	1.48 (0.83,2.13)	1.14 (0.61,1.67)	2.13 (1.48,2.79)	2.65 (2.03,3.28)	3.42 (2.61,4.24)
Neurological	epilepsy	8832	4374	4458	4448	2221	2227	126.95 (119.42,134.49)	146.15 (137.35,154.95)	3.2 (2.74,3.67)	4.04 (3.46,4.61)	3.06 (1.97,4.16)	2.48 (1.64,3.33)	2.63 (1.82,3.43)	2.95 (2.18,3.73)	3.35 (2.64,4.05)	6.26 (5.16,7.37)
Neurological	essential_tremor	1757	854	903	1194	586	608	15.8 (13.12,18.47)	18.62 (15.46,21.78)	3.05 (2.59,3.5)	4.28 (3.69,4.87)	1.11 (0.45,1.76)	1.41 (0.78,2.04)	1.46 (0.86,2.05)	2.71 (1.97,3.44)	5.74 (4.82,6.66)	6.15 (5.06,7.24)
Neurological	intracranial_htn	206	140	66	118	88	30	3.77 (2.47,5.08)	0.84 (0.17,1.51)	0.18 (0.07,0.28)	0.21 (0.08,0.34)	0.2 (-0.08,0.48)	0.3 (0.01,0.59)	0.13 (-0.05,0.3)	0.31 (0.06,0.56)	0.15 (0.0,0.3)	0.1 (-0.04,0.24)
Neurological	migraine	36612	28209	8403	25138	19497	5641	1282.27 (1259.77,1304.77)	427.82 (412.98,442.66)	27.77 (26.3,29.25)	12.43 (11.41,13.46)	39.37 (35.24,43.5)	30.49 (27.36,33.62)	20.61 (18.24,22.97)	16.97 (15.03,18.92)	15.65 (14.06,17.25)	13.97 (12.27,15.68)
Neurological	myasthenia	433	209	224	219	107	112	4.95 (3.45,6.45)	4.48 (2.93,6.03)	0.44 (0.27,0.61)	0.51 (0.3,0.71)	0.0 (0.0,0.0)	0.07 (-0.07,0.22)	0.38 (0.08,0.68)	0.36 (0.09,0.63)	0.5 (0.23,0.77)	1.1 (0.64,1.57)
Neurological	periph_neuro	16229	8403	7826	9846	5106	4740	178.58 (169.67,187.5)	201.17 (190.87,211.47)	20.83 (19.63,22.03)	23.1 (21.71,24.48)	13.97 (11.63,16.31)	18.37 (16.06,20.68)	21.98 (19.64,24.32)	23.24 (21.05,25.42)	24.53 (22.6,26.46)	23.36 (21.2,25.51)
Neurological	trigem_neur	2655	1873	782	2052	1456	596	61.18 (55.93,66.43)	31.08 (27.0,35.16)	5.81 (5.18,6.44)	3.08 (2.58,3.58)	2.92 (1.86,3.99)	2.9 (1.99,3.81)	3.63 (2.69,4.57)	5.28 (4.25,6.31)	5.14 (4.27,6.01)	5.82 (4.76,6.88)
Perinatal	HBW	9	--	5	9	--	5	--	0.7 (0.09,1.31)	--	--	--	--	--	--	--	--

Perinatal	LBW	28	26	--	28	26	--	1.65 (0.79,2.51)	--	--	--	--	--	--	--	--	--
Perinatal	PDA	98	61	37	73	47	26	3.18 (1.98,4.38)	1.68 (0.73,2.63)	0.07 (0.0,0.14)	0.06 (-0.01,0.13)	0.0 (0.0,0.0)	0.0 (0.0,0.0)	0.13 (-0.05,0.3)	0.1 (-0.04,0.25)	0.04 (-0.04,0.11)	0.1 (-0.04,0.24)
Perinatal	RDN	--	--	0	--	--	0	--	0.0 (0.0,0.0)	--	--	--	--	--	--	--	--
Perinatal	congenital_septal	1350	699	651	712	377	335	17.92 (15.07,20.76)	16.94 (13.92,19.95)	0.92 (0.67,1.16)	1.22 (0.91,1.54)	0.81 (0.25,1.36)	1.49 (0.84,2.14)	1.01 (0.52,1.51)	1.04 (0.58,1.5)	0.96 (0.58,1.34)	1.06 (0.6,1.51)
Perinatal	downs	33	28	5	22	19	--	1.06 (0.37,1.75)	--	--	--	--	--	--	--	--	--
Perinatal	intrauterine_hypoxia	25	24	--	24	23	--	1.65 (0.79,2.51)	--	--	--	--	--	--	--	--	--
Perinatal	neo_jaundice	27	13	14	27	13	14	1.18 (0.45,1.91)	1.26 (0.44,2.08)	0.02 (-0.02,0.05)	0.0 (0.0,0.0)	0.0 (0.0,0.0)	0.0 (0.0,0.0)	0.0 (0.0,0.0)	0.0 (0.0,0.0)	0.0 (0.0,0.0)	0.05 (-0.05,0.15)
Perinatal	post_term	275	271	--	275	271	--	17.33 (14.53,20.13)	--	--	--	--	--	--	--	--	--
Perinatal	prematurity	406	392	14	406	392	14	25.93 (22.51,29.36)	1.12 (0.34,1.9)	0.09 (0.01,0.17)	0.0 (0.0,0.0)	0.3 (-0.04,0.65)	0.07 (-0.07,0.22)	0.0 (0.0,0.0)	0.0 (0.0,0.0)	0.04 (-0.04,0.11)	0.0 (0.0,0.0)
Perinatal	sepsis_newborn	15	8	7	15	8	7	0.59 (0.07,1.11)	0.7 (0.09,1.31)	0.02 (-0.02,0.05)	0.0 (0.0,0.0)	0.0 (0.0,0.0)	0.0 (0.0,0.0)	0.0 (0.0,0.0)	0.05 (-0.05,0.15)	0.0 (0.0,0.0)	0.0 (0.0,0.0)
Perinatal	spina_bifida	610	353	257	408	231	177	14.62 (12.05,17.19)	15.54 (12.65,18.43)	0.26 (0.13,0.4)	0.13 (0.03,0.23)	0.1 (-0.1,0.3)	0.59 (0.18,1.01)	0.19 (-0.03,0.41)	0.1 (-0.04,0.25)	0.27 (0.07,0.47)	0.0 (0.0,0.0)
Psychiatric	ADHD	58	25	33	39	17	22	0.59 (0.07,1.11)	0.56 (0.01,1.11)	0.07 (0.0,0.14)	0.08 (0.0,0.17)	0.3 (-0.04,0.64)	0.15 (-0.06,0.35)	0.0 (0.0,0.0)	0.05 (-0.05,0.15)	0.08 (-0.03,0.18)	0.0 (0.0,0.0)

Psychiatric	BAD	4159	2260	1899	2145	1142	1003	68.6 (63.05,74.16)	76.44 (70.05,82.82)	1.66 (1.33,2.0)	1.46 (1.12,1.81)	2.23 (1.3,3.16)	2.02 (1.26,2.78)	1.85 (1.18,2.52)	0.78 (0.39,1.18)	1.74 (1.23,2.25)	1.26 (0.77,1.76)
Psychiatric	PD	1152	597	555	824	415	409	22.28 (19.11,25.45)	26.46 (22.69,30.23)	0.58 (0.38,0.78)	0.91 (0.64,1.18)	1.21 (0.53,1.89)	1.64 (0.95,2.32)	0.95 (0.47,1.43)	0.31 (0.06,0.56)	0.46 (0.2,0.72)	0.45 (0.16,0.75)
Psychiatric	alc_problems	18528	5994	12534	13043	4567	8476	67.43 (61.92,72.93)	223.01 (212.18,233.84)	39.02 (37.38,40.66)	79.91 (77.32,82.51)	50.41 (45.95,54.87)	56.66 (52.59,60.73)	59.99 (56.11,63.87)	58.99 (55.51,62.48)	58.29 (55.32,61.27)	56.38 (53.04,59.73)
Psychiatric	alzheimer	3215	1660	1555	1613	834	779	1.3 (0.53,2.06)	1.12 (0.34,1.9)	3.43 (2.95,3.91)	4.11 (3.53,4.68)	0.1 (-0.1,0.3)	0.15 (-0.06,0.35)	0.63 (0.24,1.03)	1.71 (1.13,2.3)	4.99 (4.14,5.85)	10.76 (9.32,12.21)
Psychiatric	anxiety	91720	60308	31412	50728	33377	17351	2092.2 (2064.83,2119.58)	1335.26 (1310.31,1360.2)	51.59 (49.47,53.71)	27.97 (26.35,29.59)	52.37 (47.36,57.37)	43.07 (39.16,46.99)	39.45 (35.97,42.92)	36.82 (33.78,39.86)	36.87 (34.29,39.45)	40.71 (37.68,43.74)
Psychiatric	autism	430	128	302	197	56	141	2.36 (1.32,3.39)	7.7 (5.67,9.73)	0.05 (-0.01,0.11)	0.25 (0.11,0.4)	0.5 (0.06,0.94)	0.3 (0.01,0.59)	0.13 (-0.05,0.3)	0.0 (0.0,0.0)	0.08 (-0.03,0.18)	0.1 (-0.04,0.24)
Psychiatric	delirium	6148	2635	3513	2871	1213	1658	1.89 (0.96,2.81)	3.78 (2.35,5.21)	1.88 (1.52,2.24)	3.22 (2.71,3.73)	0.3 (-0.04,0.64)	0.67 (0.23,1.1)	0.82 (0.38,1.27)	1.61 (1.04,2.18)	2.8 (2.16,3.45)	6.58 (5.45,7.71)
Psychiatric	dementia	7046	3366	3680	3618	1730	1888	50.81 (46.02,55.59)	61.32 (55.59,67.04)	1.85 (1.5,2.21)	2.6 (2.14,3.06)	0.0 (0.0,0.0)	0.15 (-0.06,0.35)	0.57 (0.2,0.94)	1.1 (0.63,1.56)	2.24 (1.66,2.82)	7.02 (5.85,8.2)
Psychiatric	dementia_ex_Alz	5774	2692	3082	3124	1460	1664	49.98 (45.23,54.73)	60.34 (54.66,66.02)	1.06 (0.79,1.33)	1.82 (1.44,2.21)	0.0 (0.0,0.0)	0.15 (-0.06,0.35)	0.32 (0.04,0.59)	0.73 (0.35,1.11)	1.16 (0.74,1.57)	4.83 (3.86,5.81)
Psychiatric	depression	159349	102153	57196	80918	51989	28929	3687.2 (3654.73,3719.67)	2447.78 (2416.25,2479.31)	50.38 (48.05,52.71)	33.97 (32.06,35.89)	52.1 (46.59,57.62)	52.84 (48.04,57.65)	47.69 (43.44,51.94)	39.7 (36.21,43.19)	34.21 (31.52,36.9)	39.52 (36.34,42.69)
Psychiatric	eating_dz	843	790	53	599	561	38	42.08 (37.73,46.44)	3.64 (2.24,5.04)	0.21 (0.09,0.33)	0.02 (-0.02,0.06)	0.2 (-0.08,0.48)	0.3 (0.01,0.59)	0.06 (-0.06,0.19)	0.0 (0.0,0.0)	0.12 (-0.02,0.25)	0.15 (-0.02,0.32)

Psychiatric	intell_dz	557	220	337	290	115	175	2.95 (1.79,4.1)	6.16 (4.34,7.98)	0.49 (0.31,0.67)	0.65 (0.42,0.88)	0.91 (0.31,1.5)	0.89 (0.39,1.39)	0.7 (0.28,1.11)	0.47 (0.16,0.77)	0.38 (0.15,0.62)	0.4 (0.12,0.68)
Psychiatric	ocd	1769	1036	733	1042	626	416	43.5 (39.07,47.93)	35.14 (30.8,39.48)	0.51 (0.33,0.7)	0.44 (0.25,0.63)	0.81 (0.25,1.37)	0.3 (0.01,0.59)	1.02 (0.52,1.52)	0.37 (0.09,0.64)	0.38 (0.15,0.62)	0.25 (0.03,0.47)
Psychiatric	schizo	2514	1130	1384	1258	575	683	28.53 (24.94,32.12)	45.22 (40.3,50.14)	1.04 (0.77,1.3)	1.2 (0.89,1.52)	1.31 (0.6,2.03)	1.79 (1.07,2.5)	1.33 (0.76,1.91)	0.78 (0.39,1.18)	0.89 (0.52,1.25)	1.01 (0.57,1.45)
Psychiatric	substance_misuse	1968	794	1174	1389	612	777	25.93 (22.51,29.36)	40.18 (35.54,44.82)	2.03 (1.66,2.4)	3.19 (2.68,3.7)	2.23 (1.3,3.16)	3.28 (2.31,4.25)	2.61 (1.81,3.4)	2.76 (2.02,3.51)	2.54 (1.93,3.15)	2.01 (1.39,2.64)
Respiratory	COPD	26386	12165	14221	12816	6019	6797	143.69 (135.68,151.7)	216.57 (205.89,227.24)	27.21 (25.84,28.58)	37.8 (36.03,39.58)	9.3 (7.4,11.2)	10.23 (8.52,11.94)	20.55 (18.3,22.8)	29.89 (27.42,32.36)	41.76 (39.23,44.28)	57.85 (54.42,61.28)
Respiratory	COPD_excl_bronchitis_NOS	25577	11669	13908	12433	5784	6649	139.92 (132.02,147.82)	211.95 (201.39,222.51)	26.19 (24.84,27.53)	37.15 (35.39,38.91)	8.79 (6.94,10.64)	9.7 (8.04,11.37)	19.83 (17.62,22.04)	29.45 (27.0,31.91)	40.56 (38.07,43.05)	56.48 (53.09,59.86)
Respiratory	allergic_rhinitis	142237	82436	59801	71608	41428	30180	3007.76 (2976.9,3038.62)	2630.19 (2597.91,2662.48)	39.65 (37.69,41.62)	40.4 (38.28,42.52)	36.59 (31.93,41.25)	36.97 (32.97,40.96)	34.2 (30.72,37.69)	38.06 (34.76,41.35)	45.22 (42.2,48.24)	42.57 (39.28,45.87)
Respiratory	asbestosis	764	30	734	365	12	353	0.12 (-0.11,0.35)	12.46 (9.87,15.05)	0.05 (-0.01,0.11)	1.31 (0.98,1.63)	0.0 (0.0,0.0)	0.07 (-0.07,0.22)	0.06 (-0.06,0.19)	0.42 (0.13,0.7)	0.46 (0.2,0.72)	2.16 (1.52,2.81)
Respiratory	aspiration_pneumo	3113	1098	2015	1361	489	872	1.53 (0.7,2.37)	3.92 (2.47,5.37)	1.41 (1.1,1.71)	3.14 (2.63,3.64)	0.6 (0.12,1.09)	0.89 (0.39,1.39)	1.39 (0.81,1.98)	1.61 (1.04,2.18)	2.84 (2.19,3.49)	4.22 (3.31,5.12)
Respiratory	asthma	78588	45315	33273	38035	21996	16039	1435.27 (1411.68,1458.87)	1270.58 (1246.16,1295.0)	25.83 (24.4,27.26)	23.56 (22.07,25.04)	23.57 (20.27,26.86)	21.49 (18.8,24.18)	23.42 (20.83,26.0)	23.49 (21.15,25.82)	26.33 (24.21,28.44)	27.84 (25.35,30.33)
Respiratory	bronchiectasis	7087	3993	3094	3519	1987	1532	69.08 (63.5,74.65)	55.58 (50.13,61.03)	7.09 (6.39,7.78)	7.08 (6.32,7.84)	2.52 (1.53,3.51)	2.45 (1.62,3.29)	3.56 (2.63,4.49)	6.44 (5.3,7.57)	8.73 (7.59,9.87)	13.86 (12.21,15.5)

Respiratory	hyper_nasal_turbs	2600	1084	1516	1213	491	722	23.34 (20.09,26.59)	46.34 (41.36,51.32)	1.06 (0.79,1.32)	2.07 (1.66,2.48)	1.82 (0.98,2.66)	1.64 (0.95,2.32)	2.41 (1.65,3.18)	1.41 (0.88,1.94)	1.12 (0.71,1.52)	1.21 (0.72,1.69)
Respiratory	nasal_polyp	9093	3327	5766	5340	2059	3281	114.46 (107.3,121.62)	223.57 (212.73,234.41)	4.36 (3.81,4.9)	9.54 (8.65,10.43)	5.49 (4.03,6.96)	5.4 (4.15,6.65)	6.23 (4.99,7.47)	6.24 (5.12,7.37)	7.34 (6.28,8.39)	8.19 (6.92,9.47)
Respiratory	pleural_effusion	15356	6844	8512	6672	2937	3735	34.18 (30.26,38.11)	54.04 (48.66,59.41)	10.77 (9.92,11.63)	18.48 (17.25,19.71)	4.33 (3.04,5.63)	5.8 (4.51,7.09)	9.53 (8.01,11.06)	12.59 (11.0,14.18)	16.99 (15.4,18.58)	26.91 (24.62,29.2)
Respiratory	pleural_plaque	2454	257	2197	1197	121	1076	0.47 (0.01,0.93)	13.58 (10.88,16.28)	0.54 (0.35,0.74)	6.14 (5.43,6.84)	0.3 (-0.04,0.64)	0.3 (0.01,0.59)	1.33 (0.76,1.9)	1.92 (1.3,2.54)	3.34 (2.64,4.05)	8.56 (7.28,9.85)
Respiratory	pneumothorax	3708	1276	2432	1910	647	1263	26.88 (23.39,30.36)	78.68 (72.2,85.16)	1.39 (1.08,1.7)	3.2 (2.69,3.71)	0.81 (0.25,1.37)	1.42 (0.78,2.05)	1.46 (0.86,2.06)	2.04 (1.4,2.68)	2.16 (1.59,2.73)	4.29 (3.38,5.2)
Respiratory	pulm_collapse	7903	3715	4188	3329	1535	1794	12.38 (10.01,14.74)	17.08 (14.05,20.11)	4.68 (4.12,5.24)	6.58 (5.85,7.31)	2.01 (1.13,2.89)	2.82 (1.92,3.72)	4.12 (3.12,5.13)	4.74 (3.77,5.71)	6.93 (5.92,7.94)	9.27 (7.93,10.61)
Respiratory	pulm_fibrosis	3320	1292	2028	1553	619	934	10.73 (8.52,12.93)	20.02 (16.74,23.3)	2.39 (1.99,2.79)	4.09 (3.52,4.67)	0.5 (0.06,0.94)	0.82 (0.33,1.3)	1.27 (0.71,1.82)	1.56 (1.0,2.12)	4.43 (3.62,5.23)	7.51 (6.3,8.71)
Respiratory	resp_failure	7848	3262	4586	3486	1474	2012	5.19 (3.65,6.72)	11.9 (9.37,14.43)	4.8 (4.23,5.37)	9.4 (8.53,10.28)	1.81 (0.97,2.65)	2.97 (2.05,3.89)	3.93 (2.95,4.91)	5.83 (4.75,6.9)	8.85 (7.7,9.99)	12.93 (11.35,14.51)
Respiratory	sinusitis	36360	22867	13493	31811	20394	11417	1112.53 (1091.37,1133.69)	738.88 (719.7,758.07)	61.94 (59.74,64.13)	41.87 (39.94,43.8)	50.39 (45.72,55.07)	50.72 (46.69,54.74)	52.07 (48.28,55.85)	54.86 (51.34,58.38)	54.82 (51.8,57.85)	50.11 (46.82,53.4)
Respiratory	sleep_apnoea	11568	3486	8082	6027	1840	4187	44.2 (39.74,48.67)	163.51 (154.21,172.81)	7.94 (7.21,8.67)	20.47 (19.17,21.77)	11.87 (9.72,14.02)	14.25 (12.22,16.27)	15.08 (13.16,17.01)	14.74 (13.01,16.47)	13.67 (12.24,15.1)	11.61 (10.1,13.12)
Skin	acne	6076	3929	2147	5781	3770	2011	270.41 (259.5,281.33)	177.79 (168.1,187.48)	2.87 (2.43,3.32)	1.91 (1.51,2.3)	6.91 (5.23,8.59)	4.79 (3.6,5.98)	2.41 (1.64,3.19)	1.27 (0.76,1.78)	1.28 (0.85,1.72)	1.37 (0.85,1.88)

Skin	actinic_keratosis	16197	7041	9156	13059	5587	7472	156.07 (147.73,164.41)	238.13 (226.95,249.31)	32.9 (31.39,34.41)	60.76 (58.5,63.03)	10.4 (8.39,12.41)	17.44 (15.2,19.68)	26.15 (23.61,28.69)	39.73 (36.87,42.58)	62.67 (59.56,65.78)	83.03 (78.89,87.16)
Skin	alopecia_areata	1079	703	376	950	611	339	35.95 (31.92,39.98)	24.36 (20.74,27.97)	1.48 (1.16,1.8)	0.89 (0.62,1.15)	1.71 (0.9,2.53)	1.94 (1.19,2.68)	1.27 (0.71,1.83)	0.94 (0.5,1.37)	1.04 (0.65,1.43)	0.91 (0.49,1.32)
Skin	dermatitis	60208	34115	26093	48976	28067	20909	1555.51 (1531.12,1579.9)	1364.37 (1339.2,1389.55)	84.37 (81.73,87.01)	80.87 (78.07,83.66)	67.98 (62.35,73.6)	72.46 (67.48,77.45)	75.64 (70.93,80.34)	82.29 (77.86,86.73)	89.26 (85.27,93.26)	94.9 (90.17,99.63)
Skin	hidradenitis	496	357	139	358	256	102	10.84 (8.63,13.06)	6.02 (4.22,7.82)	0.47 (0.3,0.65)	0.38 (0.2,0.55)	1.21 (0.52,1.89)	0.59 (0.18,1.01)	0.38 (0.08,0.68)	0.31 (0.06,0.56)	0.31 (0.09,0.52)	0.25 (0.03,0.47)
Skin	keratitis	2304	1325	979	1910	1088	822	51.39 (46.58,56.21)	48.58 (43.48,53.68)	4.03 (3.51,4.55)	3.15 (2.65,3.66)	3.64 (2.45,4.82)	3.13 (2.19,4.08)	3.25 (2.36,4.14)	2.87 (2.11,3.63)	4.21 (3.42,5.0)	4.24 (3.34,5.15)
Skin	lichen_planus	3695	2392	1303	3024	1946	1078	90.65 (84.27,97.03)	71.12 (64.95,77.28)	7.69 (6.97,8.42)	4.09 (3.52,4.67)	3.74 (2.53,4.94)	5.44 (4.19,6.69)	4.79 (3.7,5.87)	7.09 (5.89,8.29)	6.76 (5.76,7.77)	6.71 (5.56,7.85)
Skin	pilonidal	2025	598	1427	1677	502	1175	33.36 (29.48,37.24)	99.4 (92.12,106.67)	0.49 (0.31,0.68)	1.19 (0.88,1.5)	1.83 (0.98,2.67)	0.82 (0.34,1.31)	0.96 (0.47,1.44)	0.47 (0.16,0.78)	0.66 (0.34,0.97)	0.71 (0.34,1.08)
Skin	psoriasis	15545	7823	7722	10411	5397	5014	312.96 (301.25,324.68)	370.56 (356.71,384.42)	12.94 (11.98,13.89)	13.83 (12.75,14.91)	a	11.52 (9.68,13.36)	13.05 (11.23,14.86)	13.31 (11.64,14.97)	14.07 (12.6,15.54)	14.63 (12.92,16.35)
Skin	rosacea	10968	6669	4299	9981	6165	3816	313.2 (301.48,324.92)	214.75 (204.12,225.38)	21.73 (20.5,22.97)	19.62 (18.34,20.89)	24.74 (21.61,27.88)	19.88 (17.46,22.29)	21.18 (18.87,23.49)	20.99 (18.91,23.07)	21.04 (19.24,22.83)	18.46 (16.54,20.38)
Skin	seb_derm	27880	14687	13193	19762	10307	9455	601.65 (585.64,617.65)	650.41 (632.32,668.49)	26.38 (24.99,27.76)	32.93 (31.23,34.63)	29.25 (25.74,32.75)	29.88 (26.85,32.91)	28.27 (25.55,31.0)	28.13 (25.67,30.59)	30.3 (28.11,32.49)	29.81 (27.32,32.29)
Skin	urticaria	11123	7496	3627	10491	7091	3400	371.43 (358.71,384.16)	206.49 (196.06,216.92)	22.68 (21.41,23.94)	13.84 (12.77,14.91)	18.39 (15.68,21.1)	18.24 (15.92,20.56)	17.77 (15.65,19.88)	19.05 (17.06,21.03)	19.18 (17.47,20.9)	18.47 (16.55,20.39)

Skin	vitiligo	1194	691	503	974	552	422	32.42 (28.59,36.24)	27.3 (23.47,31.13)	1.2 (0.91,1.48)	1.58 (1.22,1.94)	1.41 (0.67,2.15)	1.19 (0.61,1.77)	1.59 (0.97,2.21)	1.62 (1.05,2.18)	1.35 (0.9,1.79)	1.11 (0.64,1.57)

Table S6. Number of participants and cohort characteristics of the UKB cohort compared with the national external population (CALIBER). Sex 0: female; 1: male.

		Missing	Overall	40-49	50-59	60-69
**n**			199373	41560	66630	91183
**sex, n (%)**	0	0	109471 (54.9)	22708 (54.6)	37751 (56.7)	49012 (53.8)
**sex, n (%)**	1		89902 (45.1)	18852 (45.4)	28879 (43.3)	42171 (46.2)
**country, n (%)**	E	0	159289 (79.9)	33487 (80.6)	52371 (78.6)	73431 (80.5)
**country, n (%)**	S		24839 (12.5)	5065 (12.2)	8945 (13.4)	10829 (11.9)
**country, n (%)**	W		15245 (7.6)	3008 (7.2)	5314 (8.0)	6923 (7.6)
**Townsend_q, n (%)**	least_depr	294	39866 (20.0)	7242 (17.5)	13161 (19.8)	19463 (21.4)
**Townsend_q, n (%)**	low_depr		39771 (20.0)	7147 (17.2)	13003 (19.5)	19621 (21.5)
**Townsend_q, n (%)**	medium		39810 (20.0)	7677 (18.5)	13248 (19.9)	18885 (20.7)
**Townsend_q, n (%)**	high_depr		39819 (20.0)	9020 (21.7)	13524 (20.3)	17275 (19.0)
**Townsend_q, n (%)**	most_depr		39813 (20.0)	10386 (25.0)	13582 (20.4)	15845 (17.4)

Table S7. Sex-standardised period prevalence per 10,000 persons obtained in UKB from all sources, EHR sources and estimates reported for CALIBER in the Table S4 in Kuan et al, 2019, stratified on age on 1 April 2010; 95% CI in parenthesis

group	phenotype	condition	UKB_any_4049	UKB_any_5059	UKB_any_6069	UKB_EHR_4049	UKB_EHR_5059	UKB_EHR_6069	Age4049_CALIBER	Age5059_CALIBER	Age6069_CALIBER
Benign Neo	benign_brain	Benign neoplasm of brain and other parts of central nervous system	40 (34,46)	44 (39,49)	56 (52,62)	34 (28,40)	41 (36,46)	53 (48,58)	22 (21,23)	35 (33,36)	46 (44,48)
Benign Neo	benign_colon	Benign neoplasm of colon, rectum, anus and anal canal	265 (250,281)	549 (531,568)	917 (898,937)	262 (247,279)	541 (523,560)	904 (884,924)	174 (170,177)	388 (382,394)	706 (697,714)
Benign Neo	benign_ovary	Benign neoplasm of ovary	290 (275,306)	245 (234,256)	190 (182,199)	290 (275,306)	245 (234,256)	190 (182,199)	678 (668,688)	593 (582,603)	421 (411,430)
Benign Neo	benign_stomach	Benign neoplasm of stomach and duodenum	43 (37,49)	100 (92,108)	178 (169,187)	43 (37,49)	100 (92,108)	178 (169,187)	28 (27,30)	69 (67,72)	129 (126,133)
Benign Neo	benign_uterus	Benign neoplasm and polyp of uterus	174 (162,187)	240 (229,251)	267 (257,277)	168 (157,181)	226 (216,237)	244 (235,254)	249 (243,255)	380 (372,388)	396 (387,405)
Benign Neo	cin_cervical	Carcinoma in situ_cervical	1413 (1379,1448)	1294 (1268,1320)	1080 (1059,1100)	1406 (1371,1441)	1290 (1264,1316)	1075 (1055,1096)	444 (436,452)	315 (308,323)	189 (183,195)
Benign Neo	haemangioma	Haemangioma, any site	140 (129,152)	172 (162,182)	181 (173,190)	140 (129,152)	172 (162,182)	181 (173,190)	130 (127,133)	153 (150,157)	161 (157,165)
Benign Neo	leiomyoma	Leiomyoma of uterus	523 (502,544)	579 (562,596)	509 (495,524)	493 (473,514)	510 (494,527)	386 (374,398)	770 (760,781)	953 (940,966)	699 (687,711)
Cancers	hodgkins	Hodgkin Lymphoma	14 (10,18)	13 (11,16)	13 (11,16)	13 (10,17)	12 (9,15)	12 (10,15)	9 (9,10)	9 (9,10)	12 (11,13)
Cancers	leukaemia	Leukaemia	13 (10,17)	24 (21,28)	47 (43,52)	13 (9,17)	24 (20,28)	47 (42,52)	9 (9,10)	18 (17,20)	41 (39,43)
Cancers	MDS	Myelodysplastic syndromes	3 (1,5)	6 (5,9)	9 (7,11)	3 (1,5)	6 (4,9)	9 (7,11)	2 (2,3)	5 (5,6)	15 (13,16)

Cancers	MGUS	Monoclonal gammopathy of undetermined significance (MGUS)	5 (3,7)	12 (10,15)	30 (27,34)	4 (3,7)	12 (9,15)	30 (26,34)	3 (2,3)	8 (7,9)	24 (22,25)
Cancers	NHL	Non-Hodgkin Lymphoma	26 (21,31)	40 (35,45)	80 (75,86)	24 (20,30)	39 (34,44)	79 (73,85)	16 (15,17)	31 (30,33)	63 (61,66)
Cancers	PCV	Polycythaemia vera	6 (4,9)	13 (11,17)	19 (16,22)	5 (3,8)	13 (10,16)	18 (15,21)	8 (8,9)	16 (15,17)	24 (23,26)
Cancers	plasmacell	Multiple myeloma and malignant plasma cell neoplasms	7 (4,10)	11 (9,14)	24 (21,28)	6 (4,10)	11 (8,14)	24 (21,28)	3 (2,3)	9 (8,9)	23 (21,24)
Cancers	pri_biliary	Primary Malignancy_biliary tract	--	4 (3,6)	10 (8,13)	--	4 (3,6)	10 (8,13)	--	4 (3,5)	9 (8,10)
Cancers	pri_bladder	Primary Malignancy_Bladder	11 (8,15)	32 (28,37)	92 (86,99)	10 (7,14)	30 (26,35)	88 (81,94)	8 (7,8)	25 (23,26)	83 (80,86)
Cancers	pri_bone	Primary Malignancy_Bone and articular cartilage	4 (2,6)	6 (4,8)	8 (7,11)	3 (2,5)	5 (3,7)	6 (5,8)	3 (3,4)	4 (3,4)	6 (5,7)
Cancers	pri_bowel	Primary Malignancy_colorectal and anus	28 (23,34)	92 (85,99)	189 (180,198)	27 (22,33)	88 (81,95)	179 (171,188)	18 (17,19)	60 (58,63)	156 (152,160)
Cancers	pri_brain	Primary Malignancy_Brain, Other CNS and Intracranial	13 (10,17)	17 (14,21)	20 (17,23)	9 (7,13)	14 (11,17)	17 (14,20)	8 (8,9)	12 (11,13)	17 (15,18)
Cancers	pri_breast	Primary Malignancy_Breast	159 (147,171)	265 (254,277)	419 (406,432)	157 (145,168)	261 (249,272)	405 (393,418)	100 (97,102)	209 (205,214)	350 (344,355)
Cancers	pri_cervical	Primary Malignancy_Cervical	43 (37,49)	53 (48,59)	46 (42,50)	15 (11,18)	16 (14,20)	15 (13,18)	23 (22,25)	31 (28,33)	36 (33,39)
Cancers	pri_kidney	Primary Malignancy_Kidney and Ureter	10 (7,13)	25 (22,30)	49 (45,54)	9 (6,12)	24 (21,29)	46 (41,50)	7 (6,7)	17 (16,18)	39 (37,41)
Cancers	pri_liver	Primary Malignancy_Liver	2 (1,3)	7 (5,10)	12 (10,14)	1 (0,2)	5 (3,7)	9 (7,11)	--	5 (5,6)	10 (9,11)

Cancers	pri_lung	Primary Malignancy_Lung and trachea	12 (9,16)	40 (35,45)	80 (74,86)	9 (6,12)	32 (28,37)	67 (62,73)	9 (8,10)	38 (36,40)	108 (105,112)
Cancers	pri_melanoma	Primary Malignancy_Malignant Melanoma	82 (73,91)	125 (117,134)	204 (195,214)	66 (58,74)	90 (83,98)	129 (122,137)	42 (40,44)	65 (62,67)	105 (101,108)
Cancers	pri_mesothelioma	Primary Malignancy_Mesothelioma	--	--	7 (5,9)	--	--	7 (5,9)	--	--	7
Cancers	pri_multindep	Primary Malignancy_Multiple independent sites	--	--	3 (2,5)	--	--	3 (2,5)	--	--	3
Cancers	pri_oesoph	Primary Malignancy_Oesophageal	2 (1,4)	11 (8,14)	25 (22,29)	2 (1,4)	10 (8,13)	24 (21,28)	3 (2,3)	13 (12,14)	30 (28,32)
Cancers	pri_oroph	Primary Malignancy_Oro-pharyngeal	16 (12,20)	31 (26,35)	45 (41,50)	14 (10,18)	26 (22,30)	35 (31,39)	11 (10,12)	27 (25,28)	38 (36,40)
Cancers	pri_other	Primary Malignancy_Other Organs	42 (36,49)	94 (87,102)	158 (150,167)	37 (31,43)	84 (78,92)	143 (135,151)	16 (15,17)	36 (34,38)	68 (66,71)
Cancers	pri_ovarian	Primary Malignancy_Ovarian	18 (15,23)	31 (27,35)	48 (44,53)	16 (12,20)	27 (23,31)	42 (38,46)	20 (18,22)	41 (38,43)	69 (65,73)
Cancers	pri_pancr	Primary Malignancy_Pancreatic	3 (2,5)	8 (6,11)	20 (17,23)	3 (1,5)	8 (6,10)	20 (17,23)	3 (2,3)	9 (8,10)	22 (20,23)
Cancers	pri_prost	Primary Malignancy_Prostate	9 (6,13)	91 (83,99)	322 (310,334)	9 (6,13)	90 (83,98)	317 (305,329)	12 (11,14)	103 (99,107)	432 (422,441)
Cancers	pri_skin	Primary Malignancy_Other Skin and subcutaneous tissue	171 (158,184)	360 (346,375)	781 (763,799)	163 (151,175)	343 (329,358)	757 (740,776)	103 (100,105)	249 (244,254)	584 (576,591)
Cancers	pri_stomach	Primary Malignancy_Stomach	5 (3,7)	12 (9,15)	23 (20,27)	3 (1,5)	11 (8,14)	21 (18,24)	3 (2,3)	9 (8,10)	21 (20,22)
Cancers	pri_testis	Primary Malignancy_Testicular	32 (27,38)	23 (19,27)	17 (14,20)	25 (21,31)	18 (15,22)	11 (9,14)	29 (27,31)	26 (24,28)	19 (17,20)
Cancers	pri_thyroid	Primary Malignancy_Thyroid	12 (9,16)	17 (14,20)	17 (14,20)	11 (8,14)	15 (12,18)	14 (12,17)	8 (8,9)	10 (9,11)	11 (10,12)

Cancers	pri_uterine	Primary Malignancy_Uterine	13 (10,17)	32 (28,36)	64 (59,69)	9 (6,12)	25 (21,29)	52 (48,57)	12 (10,13)	42 (40,45)	100 (96,105)
Cancers	sec_adrenal	Secondary Malignancy_Adrenal gland	--	4 (2,6)	8 (7,11)	--	4 (2,6)	8 (7,11)	--	6 (5,7)	13 (12,14)
Cancers	sec_bone	Secondary Malignancy_Bone	14 (11,18)	33 (29,38)	66 (60,71)	14 (11,18)	33 (29,38)	66 (60,71)	13 (12,14)	33 (31,35)	77 (74,80)
Cancers	sec_bowel	Secondary Malignancy_Bowel	--	4 (2,5)	6 (5,8)	--	4 (2,5)	6 (5,8)	--	3 (3,4)	8 (7,9)
Cancers	sec_brain	Secondary Malignancy_Brain, Other CNS and Intracranial	6 (4,9)	13 (10,16)	24 (21,27)	6 (4,9)	13 (10,16)	24 (21,27)	7 (6,7)	15 (14,16)	29 (27,31)
Cancers	sec_liver	Secondary malignancy_Liver and intrahepatic bile duct	17 (13,21)	39 (35,44)	71 (66,77)	17 (13,21)	39 (35,44)	71 (66,77)	16 (15,17)	42 (41,44)	92 (88,95)
Cancers	sec_LN	Secondary Malignancy_Lymph Nodes	60 (53,68)	109 (101,117)	159 (151,168)	60 (53,68)	109 (101,117)	159 (151,168)	46 (44,48)	96 (93,99)	158 (154,162)
Cancers	sec_lung	Secondary Malignancy_Lung	12 (9,16)	29 (25,33)	57 (52,62)	12 (9,16)	29 (25,33)	57 (52,62)	12 (11,13)	29 (27,30)	62 (60,65)
Cancers	sec_other	Secondary Malignancy_Other organs	18 (15,23)	36 (31,41)	63 (58,69)	18 (15,23)	36 (31,41)	63 (58,69)	8 (7,8)	18 (16,19)	37 (35,38)
Cancers	sec_peritoneum	Secondary Malignancy_retroperitoneum and peritoneum	9 (7,13)	21 (17,24)	37 (33,41)	9 (7,13)	21 (17,24)	37 (33,41)	7 (6,8)	19 (18,20)	39 (37,41)
Cancers	sec_pleura	Secondary Malignancy_Pleura	3 (2,5)	9 (7,12)	15 (12,17)	3 (2,5)	9 (7,12)	15 (12,17)	3 (2,3)	8 (7,9)	17 (16,18)
Cardiovascular	AAA	Abdominal aortic aneurysm	5 (3,7)	14 (11,17)	51 (46,56)	3 (2,6)	11 (8,13)	45 (40,49)	4 (3,4)	13 (12,14)	91 (88,94)
Cardiovascular	AF	Atrial fibrillation	91 (81,100)	246 (234,259)	676 (659,694)	87 (78,96)	239 (227,251)	665 (648,683)	73 (71,75)	213 (209,217)	643 (635,651)
Cardiovascular	av_block_1	Atrioventricular block, first degree	9 (6,13)	21 (17,24)	48 (43,53)	9 (6,13)	21 (17,24)	48 (43,53)	5 (5,6)	13 (12,14)	36 (34,38)

Cardiovascular	av_block_2	Atrioventricular block, second degree	5 (3,8)	8 (6,11)	17 (15,20)	5 (3,8)	8 (6,11)	17 (15,20)	3 (2,3)	6 (5,7)	17 (16,18)
Cardiovascular	av_block_3	Atrioventricular block, complete	4 (2,7)	6 (4,8)	23 (20,27)	4 (2,7)	6 (4,8)	23 (20,27)	3 (2,3)	7 (7,8)	23 (22,25)
Cardiovascular	bifasc_block	Bifascicular block	--	--	3 (2,5)	--	--	3 (2,5)	--	--	3 (3,4)
Cardiovascular	cardiomyo_oth	Other Cardiomyopathy	16 (12,20)	26 (22,30)	44 (40,48)	16 (12,20)	26 (22,30)	44 (40,48)	16 (15,17)	31 (30,33)	59 (57,61)
Cardiovascular	CHD_NOS	Coronary heart disease not otherwise specified	204 (191,219)	561 (542,580)	1260 (1237,1284)	183 (170,197)	539 (520,557)	1237 (1214,1261)	34 (32,35)	94 (91,97)	211 (207,216)
Cardiovascular	dcm	Dilated cardiomyopathy	10 (8,14)	16 (13,20)	26 (23,30)	10 (8,14)	16 (13,20)	26 (23,30)	11 (10,11)	21 (19,22)	35 (33,36)
Cardiovascular	hf	Heart failure	42 (36,49)	113 (105,122)	306 (294,318)	41 (35,48)	111 (103,120)	304 (292,316)	45 (43,46)	133 (130,136)	362 (355,368)
Cardiovascular	hocm	Hypertrophic Cardiomyopathy	7 (4,10)	10 (8,13)	10 (8,13)	6 (4,9)	9 (7,12)	9 (7,11)	4 (4,5)	8 (7,9)	11 (10,12)
Cardiovascular	hypertension	Hypertension	1254 (1220,1289)	2546 (2507,2586)	4212 (4169,4254)	1248 (1214,1283)	2534 (2495,2573)	4201 (4158,4243)	1180 (1171,	2655 (2640,	4551 (4530,
Cardiovascular	Intracereb_haem	Intracerebral haemorrhage	11 (8,14)	25 (21,29)	39 (35,44)	9 (6,12)	22 (18,26)	35 (32,39)	12 (11,13)	25 (23,26)	51 (49,53)
Cardiovascular	Isch_stroke	Ischaemic stroke	32 (27,39)	73 (66,80)	150 (142,159)	32 (27,39)	73 (66,80)	150 (142,158)	32 (30,33)	85 (82,88)	207 (202,211)
Cardiovascular	LBBB	Left bundle branch block	12 (9,16)	27 (24,32)	71 (66,77)	12 (9,16)	27 (24,32)	71 (66,77)	10 (9,11)	29 (28,31)	74 (72,77)
Cardiovascular	mult_valve	Multiple valve dz	9 (7,13)	23 (20,28)	59 (54,64)	9 (7,13)	23 (20,28)	59 (54,64)	11 (10,12)	29 (27,31)	78 (75,81)
Cardiovascular	myocardial_infarction	Myocardial infarction	151 (139,164)	350 (335,365)	728 (711,747)	133 (122,145)	321 (307,336)	675 (658,692)	89 (86,91)	271 (266,275)	558 (550,565)
Cardiovascular	nonRh_aortic	Nonrheumatic aortic valve disorders	24 (19,29)	51 (45,56)	141 (133,149)	24 (19,29)	51 (45,56)	141 (133,149)	19 (18,20)	48 (46,50)	141 (138,145)

Cardiovascular	nonRh_mitral	Nonrheumatic mitral valve disorders	31 (26,36)	59 (53,65)	115 (108,122)	30 (25,36)	58 (53,65)	114 (108,122)	35 (34,37)	68 (65,70)	137 (133,141)
Cardiovascular	PE	Pulmonary embolism	73 (65,81)	122 (114,131)	191 (182,200)	45 (39,52)	87 (80,94)	145 (137,153)	61 (59,63)	100 (97,103)	185 (181,190)
Cardiovascular	pericardial_effusion	Pericardial effusion (noninflammatory)	18 (14,23)	25 (21,29)	42 (38,46)	18 (14,23)	25 (21,29)	42 (37,46)	9 (8,10)	17 (16,18)	32 (30,34)
Cardiovascular	peripheral_arterial_disease	Peripheral arterial disease	49 (42,56)	97 (90,105)	224 (214,234)	32 (27,38)	80 (73,87)	203 (194,213)	36 (34,37)	118 (115,121)	307 (302,313)
Cardiovascular	prim_pulm_htn	Primary pulmonary hypertension	5 (3,8)	7 (5,9)	19 (16,22)	5 (3,8)	7 (5,9)	19 (16,22)	5 (5,6)	11 (10,12)	27 (25,28)
Cardiovascular	raynauds	Raynaud's syndrome	146 (135,158)	142 (133,151)	159 (151,168)	137 (126,148)	137 (128,146)	154 (146,162)	109 (106,112)	131 (128,134)	160 (156,164)
Cardiovascular	RBBB	Right bundle branch block	26 (21,32)	38 (34,44)	83 (77,89)	26 (21,32)	38 (34,44)	83 (77,89)	17 (16,18)	34 (32,35)	72 (69,75)
Cardiovascular	Rh_valve	Rheumatic valve dz	6 (4,9)	14 (11,17)	33 (29,37)	5 (3,8)	14 (11,17)	32 (29,36)	6 (6,7)	14 (12,15)	34 (32,36)
Cardiovascular	sec_pulm_htn	Secondary pulmonary hypertension	4 (2,6)	5 (3,7)	12 (10,15)	4 (2,6)	5 (3,7)	12 (10,15)	3 (3,4)	8 (7,9)	17 (16,18)
Cardiovascular	sick_sinus	Sick sinus syndrome	4 (2,6)	8 (6,11)	20 (17,23)	3 (2,6)	8 (6,10)	20 (17,23)	2 (2,3)	5 (4,6)	15 (14,16)
Cardiovascular	stable_angina	Stable angina	126 (116,138)	396 (380,412)	937 (917,957)	126 (116,138)	396 (380,412)	937 (917,957)	122 (119,125)	403 (397,409)	913 (903,923)
Cardiovascular	Stroke_NOS	Stroke NOS	65 (58,74)	145 (136,155)	311 (299,323)	65 (58,74)	145 (136,155)	311 (299,323)	26 (24,27)	70 (67,72)	173 (169,177)
Cardiovascular	Subarach	Subarachnoid haemorrhage	22 (18,27)	38 (33,43)	46 (41,50)	21 (17,26)	35 (31,40)	42 (38,47)	19 (18,20)	31 (30,33)	43 (41,45)
Cardiovascular	subdural_haem	Subdural haematoma - nontraumatic	6 (4,9)	8 (6,10)	15 (13,18)	4 (2,6)	5 (3,7)	11 (9,14)	4 (3,4)	6 (6,7)	14 (13,15)
Cardiovascular	SVT	Supraventricular tachycardia	52 (45,59)	77 (70,84)	119 (112,126)	49 (42,56)	73 (67,80)	115 (108,122)	55 (53,57)	80 (77,82)	130 (126,133)

Cardiovascular	TIA	Transient ischaemic attack	38 (32,44)	109 (101,117)	279 (268,290)	33 (27,39)	96 (88,104)	246 (236,256)	40 (38,42)	119 (116,122)	305 (300,311)
Cardiovascular	trifasc_block	Trifascicular block	--	--	4 (3,6)	--	--	4 (3,6)	--	--	3 (3,4)
Cardiovascular	unstable_angina	Unstable Angina	42 (36,49)	118 (109,127)	245 (235,256)	42 (36,49)	118 (109,127)	245 (235,256)	56 (54,58)	161 (158,165)	311 (305,316)
Cardiovascular	VT	Ventricular tachycardia	11 (8,15)	17 (14,20)	33 (29,37)	11 (8,15)	17 (14,20)	33 (29,37)	9 (8,10)	19 (17,20)	39 (37,40)
Cardiovascular	vte_ex_pe	Venous thromboembolic disease (Excl PE)	146 (135,159)	239 (227,251)	379 (367,392)	93 (84,102)	146 (137,156)	233 (224,244)	107 (104,110)	166 (162,170)	276 (271,281)
Digestive	anal_fissure	Anal fissure	335 (318,353)	297 (284,310)	259 (248,269)	332 (315,350)	294 (281,308)	255 (245,266)	314 (309,318)	300 (295,305)	266 (260,271)
Digestive	angiodysplasia_colon	Angiodysplasia of colon	3 (2,6)	8 (6,10)	18 (15,21)	3 (2,6)	8 (6,10)	18 (15,21)	3 (2,3)	7 (7,8)	17 (15,18)
Digestive	anorectal_fistula	Anorectal fistula	77 (68,86)	84 (77,91)	69 (64,75)	76 (68,85)	83 (76,91)	69 (64,75)	63 (61,65)	70 (68,73)	65 (62,67)
Digestive	anorectal_prolapse	Anorectal prolapse	20 (16,25)	41 (37,47)	61 (56,66)	20 (16,24)	37 (33,42)	57 (52,62)	19 (18,21)	30 (29,32)	54 (52,56)
Digestive	appendicitis	Appendicitis	443 (423,464)	485 (468,502)	501 (487,516)	379 (360,398)	401 (386,417)	395 (382,408)	689 (682,696)	795 (787,803)	854 (844,863)
Digestive	autoimm_liver	Autoimmune liver disease	8 (6,11)	15 (12,18)	21 (18,24)	8 (5,11)	14 (11,17)	20 (18,23)	5 (5,6)	10 (9,11)	15 (14,16)
Digestive	barretts	Barrett's oesophagus	43 (37,50)	92 (85,100)	152 (144,161)	37 (31,44)	83 (76,91)	139 (132,147)	30 (29,31)	68 (66,71)	122 (119,126)
Digestive	cholangitis	Cholangitis	6 (4,9)	16 (13,20)	27 (24,30)	6 (4,9)	16 (13,20)	27 (23,30)	8 (7,9)	15 (14,17)	31 (29,33)
Digestive	cholecystitis	Cholecystitis	167 (155,179)	216 (205,227)	306 (295,318)	164 (152,176)	209 (199,220)	297 (286,309)	179 (175,182)	234 (230,239)	315 (309,320)
Digestive	cholelithiasis	Cholelithiasis	330 (313,348)	468 (452,484)	667 (651,684)	304 (288,321)	403 (388,418)	553 (538,568)	323 (318,328)	442 (436,449)	621 (613,628)

Digestive	cirrhosis	Liver fibrosis, sclerosis and cirrhosis	31 (25,37)	55 (49,61)	66 (60,71)	29 (24,35)	51 (46,57)	62 (57,67)	40 (38,42)	66 (64,68)	73 (70,76)
Digestive	coeliac	Coeliac disease	111 (101,121)	116 (108,124)	110 (104,117)	62 (54,70)	71 (64,77)	73 (67,79)	37 (35,38)	43 (41,45)	53 (50,55)
Digestive	crohns	Crohn's disease	54 (47,62)	55 (50,61)	57 (52,62)	49 (42,56)	49 (44,54)	51 (47,56)	52 (50,54)	54 (52,56)	56 (54,59)
Digestive	diverticuli	Diverticular disease of intestine (acute and chronic)	209 (195,224)	585 (566,603)	1164 (1142,1186)	200 (187,214)	566 (548,585)	1127 (1105,1149)	149 (146,152)	450 (444,457)	992 (982,
Digestive	fatty_liver	Fatty Liver	87 (78,96)	110 (103,119)	107 (100,114)	87 (78,96)	110 (103,119)	107 (100,114)	52 (50,54)	79 (76,81)	77 (74,80)
Digestive	gastritis_duodenitis	Gastritis and duodenitis	743 (716,769)	989 (965,1014)	1317 (1293,1340)	733 (707,760)	982 (958,1006)	1307 (1284,1331)	615 (608,621)	840 (831,848)	1101 (1090,
Digestive	GORD	Gastro-oesophageal reflux disease	837 (810,866)	1147 (1121,1173)	1473 (1448,1498)	585 (562,609)	835 (813,857)	1071 (1049,1092)	935 (926,943)	1255 (1245,	1614 (1601,
Digestive	hernia_abdo	Abdominal Hernia	623 (598,648)	833 (811,857)	1211 (1188,1234)	608 (584,633)	813 (790,836)	1184 (1161,1208)	625 (618,631)	853 (844,861)	1238 (1227,
Digestive	hernia_diaphragm	Diaphragmatic hernia	358 (340,377)	649 (629,668)	1015 (995,1036)	326 (309,344)	590 (572,609)	932 (912,952)	335 (330,339)	611 (604,618)	1005 (995,
Digestive	IBS	Irritable bowel syndrome	539 (517,562)	576 (558,594)	524 (509,539)	361 (343,379)	402 (387,417)	369 (357,382)	839 (832,847)	865 (856,874)	840 (831,849)
Digestive	liver_alc	Alcoholic liver disease	22 (17,27)	40 (35,45)	36 (32,40)	21 (17,26)	40 (35,45)	36 (32,40)	47 (46,49)	74 (72,77)	72 (69,75)
Digestive	liver_fail	Hepatic failure	9 (7,13)	18 (15,21)	23 (20,26)	6 (4,9)	12 (9,15)	16 (14,19)	15 (14,16)	23 (22,24)	26 (25,28)
Digestive	oesoph_ulc	Oesophagitis and oesophageal ulcer	452 (431,473)	708 (687,729)	971 (951,991)	452 (431,473)	708 (687,729)	971 (951,991)	536 (530,542)	810 (802,819)	1115 (1104,
Digestive	pancreatitis	Pancreatitis	49 (43,57)	67 (61,74)	97 (91,103)	44 (38,51)	63 (57,70)	91 (84,97)	59 (57,61)	77 (74,79)	103 (100,106)

Digestive	peritonitis	Peritonitis	61 (54,69)	91 (84,99)	118 (111,125)	47 (40,54)	66 (60,72)	83 (77,89)	52 (50,54)	77 (74,80)	110 (107,114)
Digestive	portal_htn	Portal hypertension	6 (4,9)	13 (11,17)	16 (13,19)	6 (4,9)	13 (11,17)	16 (13,19)	15 (14,16)	25 (23,26)	26 (24,27)
Digestive	ulc_colitis	Ulcerative colitis	104 (95,115)	121 (113,130)	140 (133,148)	98 (89,108)	115 (107,124)	132 (125,140)	70 (68,73)	89 (86,92)	115 (112,118)
Digestive	ulcer_peptic	Peptic ulcer disease	215 (201,230)	367 (352,382)	539 (524,554)	173 (160,187)	301 (287,315)	468 (453,482)	155 (152,159)	293 (288,298)	471 (464,478)
Digestive	varices	Oesophageal varices	12 (8,15)	19 (16,23)	24 (21,27)	12 (8,15)	19 (16,23)	23 (20,26)	14 (13,15)	25 (24,27)	30 (28,31)
Digestive	volvulus	Volvulus	9 (7,13)	15 (12,18)	21 (18,24)	9 (7,13)	15 (12,18)	21 (18,24)	7 (6,7)	12 (11,13)	21 (20,23)
Ear	deaf	Hearing loss	585 (562,609)	927 (904,951)	1613 (1586,1639)	524 (502,547)	803 (781,825)	1342 (1319,1367)	529 (523,535)	795 (786,803)	1285 (1274,
Ear	meniere	Meniere disease	32 (27,38)	66 (60,72)	97 (91,104)	28 (24,34)	54 (49,60)	82 (77,89)	22 (21,23)	45 (43,47)	76 (73,78)
Ear	tinnitus	Tinnitus	825 (797,853)	1129 (1103,1155)	1284 (1261,1308)	299 (282,316)	467 (450,484)	544 (528,559)	284 (280,288)	492 (486,499)	661 (653,669)
Endocrine	CF	Cystic Fibrosis	5 (3,8)	6 (4,8)	4 (2,5)	5 (3,7)	6 (4,8)	3 (2,5)	5 (4,5)	5 (4,6)	7 (6,8)
Endocrine	diabetes_nos	Diabetes NOS	221 (207,236)	404 (388,420)	629 (613,646)	40 (34,46)	77 (71,85)	121 (114,128)	30 (28,31)	43 (41,45)	61 (58,63)
Endocrine	diabetes_t1	Diabetes Type I	65 (57,73)	75 (68,82)	100 (93,107)	61 (53,69)	74 (67,81)	98 (91,104)	55 (53,57)	49 (47,51)	38 (36,40)
Endocrine	diabetes_t2	Diabetes Type II	270 (254,286)	510 (492,528)	816 (798,835)	252 (237,268)	486 (469,504)	789 (771,808)	382 (376,387)	820 (811,828)	1323 (1311,
Endocrine	obesity	Obesity	845 (817,873)	1053 (1028,1078)	1108 (1087,1130)	844 (817,873)	1052 (1028,1077)	1107 (1086,1129)	2154 (2142,	2639 (2623,	2969 (2952,
Endocrine	PCOS	Polycystic ovarian syndrome	83 (75,92)	26 (22,30)	7 (6,9)	69 (61,77)	20 (17,23)	5 (4,6)	86 (83,90)	20 (18,22)	7 (6,8)

Endocrine	PTH	Hyperparathyroidism	16 (12,20)	28 (25,33)	50 (46,55)	14 (11,18)	27 (23,31)	49 (44,53)	12 (11,13)	23 (21,24)	41 (39,43)
Endocrine	SIADH	Syndrome of inappropriate secretion of antidiuretic hormone	--	2 (1,4)	6 (4,7)	--	2 (1,4)	6 (4,7)	--	3 (2,3)	7 (6,8)
Endocrine	thyroid	Hypo or hyperthyroidism	516 (495,538)	765 (745,786)	957 (937,977)	461 (441,482)	685 (666,704)	874 (855,893)	468 (462,474)	710 (702,718)	943 (934,953)
Eye	actinic_keratosis	Actinic keratosis	101 (92,112)	296 (283,310)	696 (679,714)	101 (92,112)	296 (283,310)	696 (679,714)	56 (54,58)	64 (61,66)	75 (72,77)
Eye	ant_uveitis	Anterior and Intermediate Uveitis	64 (56,72)	88 (81,95)	89 (83,95)	61 (54,69)	85 (78,92)	87 (81,93)	75 (73,78)	105 (102,108)	123 (120,127)
Eye	blind	Visual impairment and blindness	38 (32,44)	56 (50,62)	76 (70,82)	38 (32,44)	56 (50,62)	76 (70,82)	55 (53,57)	82 (79,85)	135 (131,139)
Eye	cataract	Cataract	127 (117,139)	371 (357,386)	1110 (1088,1132)	114 (104,125)	329 (316,344)	1009 (989,1030)	92 (90,95)	297 (292,302)	946 (936,956)
Eye	diab_eye	Diabetic ophthalmic complications	177 (164,190)	297 (283,310)	445 (431,459)	160 (147,173)	278 (266,292)	415 (402,428)	121 (118,124)	252 (248,257)	424 (418,431)
Eye	glaucoma	Glaucoma	88 (79,98)	205 (194,217)	419 (406,433)	57 (50,65)	155 (146,165)	348 (336,361)	48 (46,50)	130 (126,133)	319 (313,325)
Eye	macula_degen	Macular degeneration	41 (35,47)	97 (89,105)	250 (240,261)	20 (16,25)	42 (37,47)	150 (143,159)	18 (17,19)	51 (49,53)	164 (160,168)
Eye	post_uveitis	Posterior Uveitis	8 (5,11)	10 (8,13)	11 (9,13)	8 (5,11)	10 (8,13)	11 (9,13)	6 (6,7)	8 (7,9)	9 (8,10)
Eye	ptosis	Ptosis of eyelid	28 (23,34)	46 (41,51)	73 (68,79)	28 (23,34)	46 (41,51)	73 (67,79)	24 (23,25)	38 (37,40)	63 (61,66)
Eye	retinal_detach	Retinal detachments and breaks	51 (44,59)	102 (94,110)	167 (159,176)	45 (38,52)	89 (82,97)	147 (139,155)	37 (36,39)	72 (70,75)	128 (124,132)
Eye	retinal_vasc_occl	Retinal vascular occlusions	14 (11,19)	32 (28,36)	79 (73,85)	13 (9,17)	30 (26,34)	75 (69,81)	9 (8,10)	27 (25,28)	71 (69,74)

Eye	scleritis	Scleritis and episcleritis	20 (16,25)	30 (26,34)	32 (28,36)	20 (16,25)	30 (26,34)	32 (28,36)	56 (54,58)	75 (72,77)	79 (76,81)
Genitourinary	AKI	Acute Kidney Injury	33 (27,39)	72 (65,79)	157 (149,165)	33 (27,39)	72 (65,79)	157 (149,165)	46 (44,48)	103 (100,106)	249 (244,254)
Genitourinary	BPH	Hyperplasia of prostate	81 (72,91)	321 (307,336)	867 (847,887)	79 (70,89)	316 (301,330)	851 (831,871)	112 (108,116)	546 (537,556)	1636 (1618,
Genitourinary	chr_cystitis	Non-acute cystitis	16 (12,20)	24 (21,28)	39 (35,44)	16 (12,20)	24 (21,28)	39 (35,44)	14 (13,15)	22 (21,23)	39 (37,40)
Genitourinary	CKD	Chronic Kidney Disease	74 (66,83)	164 (154,174)	408 (395,422)	68 (61,77)	153 (144,163)	397 (384,410)	33 (31,34)	140 (136,143)	611 (603,619)
Genitourinary	dysmenorrhoea	Dysmenorrhoea	348 (331,365)	232 (222,244)	96 (90,102)	340 (323,358)	224 (214,235)	88 (83,94)	919 (908,930)	674 (663,685)	282 (274,290)
Genitourinary	ED	Erectile dysfunction	397 (377,418)	729 (707,751)	1090 (1067,1112)	397 (377,417)	728 (706,750)	1088 (1066,1110)	814 (804,825)	1551 (1535,	2306 (2284,
Genitourinary	endometrial_hyper	Endometrial hyperplasia and hypertrophy	57 (51,65)	66 (61,73)	55 (50,60)	57 (51,65)	66 (61,73)	55 (50,60)	124 (120,128)	162 (157,168)	131 (125,136)
Genitourinary	endometriosis	Endometriosis	312 (296,329)	240 (229,251)	144 (137,152)	278 (263,294)	199 (189,209)	104 (98,111)	511 (503,520)	373 (365,381)	187 (181,193)
Genitourinary	ESRD	End stage renal disease	20 (15,24)	32 (28,37)	45 (40,49)	18 (14,22)	27 (23,32)	38 (34,43)	18 (17,19)	30 (28,32)	46 (44,48)
Genitourinary	female_genital_prolapse	Female genital prolapse	216 (202,230)	378 (365,392)	653 (637,669)	212 (198,225)	369 (355,383)	631 (616,647)	400 (392,407)	736 (725,748)	1221 (1205,
Genitourinary	female_infertility	Female infertility	226 (213,241)	119 (112,128)	65 (60,70)	215 (201,229)	107 (100,115)	58 (54,63)	575 (566,585)	305 (297,312)	134 (128,139)
Genitourinary	GN	Glomerulonephritis	42 (36,48)	59 (53,65)	84 (78,90)	40 (34,46)	55 (49,61)	81 (75,87)	36 (34,37)	52 (50,54)	90 (87,93)
Genitourinary	hydrocele	Hydrocoele (incl infected)	71 (62,80)	72 (65,79)	114 (107,121)	71 (62,80)	72 (65,79)	114 (107,121)	131 (127,135)	156 (151,161)	222 (216,229)
Genitourinary	male_infertility	Male infertility	55 (48,63)	37 (32,42)	20 (17,23)	54 (47,62)	36 (31,41)	19 (16,22)	136 (132,140)	85 (81,88)	44 (41,47)

Genitourinary	menorrhagia	Menorrhagia and polymenorrhoea	1185 (1154,1217)	991 (969,1014)	522 (508,536)	1177 (1145,1209)	972 (950,995)	490 (476,504)	2788 (2768,	2654 (2632,	1410 (1393,
Genitourinary	neuro_bladder	Neuromuscular dysfunction of bladder	101 (92,111)	151 (142,161)	216 (206,225)	101 (92,111)	151 (142,161)	216 (206,225)	81 (78,83)	126 (123,129)	191 (187,196)
Genitourinary	obstr_reflux	Obstructive and reflux uropathy	58 (51,65)	71 (64,78)	101 (94,108)	58 (51,65)	71 (64,78)	101 (94,108)	60 (58,62)	77 (75,80)	117 (114,121)
Genitourinary	PCB	Postcoital and contact bleeding	200 (187,214)	150 (142,159)	73 (67,78)	200 (187,214)	150 (142,159)	73 (67,78)	476 (468,484)	342 (334,350)	166 (160,172)
Genitourinary	PMB	Postmenopausal bleeding	93 (84,102)	430 (416,445)	591 (576,607)	93 (84,102)	430 (416,445)	591 (576,607)	127 (123,131)	833 (821,845)	1170 (1155,
Genitourinary	TIN	Tubulo-interstitial nephritis	26 (22,32)	26 (22,30)	26 (23,29)	24 (20,29)	24 (21,28)	22 (19,26)	31 (30,33)	28 (26,29)	28 (26,30)
Genitourinary	undescended_testis	Undescended testicle	29 (24,35)	19 (15,23)	16 (13,18)	23 (18,28)	15 (12,19)	12 (10,15)	50 (47,52)	39 (37,42)	28 (25,30)
Genitourinary	urine_incont	Urinary Incontinence	369 (351,387)	503 (487,520)	631 (616,648)	354 (337,372)	469 (454,485)	583 (568,599)	388 (382,393)	536 (529,543)	673 (665,681)
Genitourinary	urolithiasis	Urolithiasis	227 (213,243)	322 (308,336)	390 (377,403)	211 (197,226)	302 (289,316)	361 (349,374)	270 (266,274)	349 (344,355)	437 (431,444)
Haem/Imm	2ry_polycythaemia	Secondary polycythaemia	7 (4,10)	9 (7,12)	12 (9,14)	7 (4,10)	9 (7,12)	12 (9,14)	9 (8,10)	16 (15,17)	22 (20,23)
Haem/Imm	agranulocytosis	Agranulocytosis	85 (76,94)	123 (114,131)	150 (142,158)	84 (75,93)	121 (113,130)	149 (141,157)	51 (49,53)	85 (82,87)	120 (116,123)
Haem/Imm	aplastic	Aplastic anaemias	7 (5,10)	14 (11,17)	19 (16,22)	7 (5,10)	13 (11,17)	18 (15,21)	6 (6,7)	12 (11,13)	21 (20,23)
Haem/Imm	b12_def	Vitamin B12 deficiency anaemia	109 (100,120)	134 (126,143)	175 (166,183)	104 (95,115)	128 (119,137)	169 (160,177)	32 (30,33)	45 (43,47)	71 (68,73)
Haem/Imm	folatedef	Folate deficiency anaemia	29 (24,35)	25 (22,29)	35 (32,39)	29 (24,35)	25 (22,29)	35 (32,39)	9 (8,10)	14 (13,15)	21 (20,23)
Haem/Imm	hyposplenism	Hyposplenism	18 (14,22)	22 (18,26)	24 (21,28)	18 (14,22)	22 (18,26)	24 (21,28)	16 (15,17)	22 (21,23)	27 (25,28)

Haem/Imm	IDA	Iron deficiency anaemia	395 (377,414)	408 (393,423)	412 (399,426)	321 (305,338)	357 (343,371)	382 (370,395)	434 (429,440)	462 (456,469)	521 (514,528)
Haem/Imm	immunodef	Immunodeficiencies	7 (4,9)	5 (3,7)	7 (6,9)	7 (4,9)	5 (3,7)	7 (6,9)	4 (4,5)	5 (5,6)	8 (7,9)
Haem/Imm	oth_anaemia	Other anaemias	476 (456,497)	537 (520,555)	634 (618,651)	476 (456,497)	537 (520,555)	634 (618,651)	431 (426,437)	489 (482,495)	649 (641,657)
Haem/Imm	oth_haem_anaemia	Other haemolytic anaemias	10 (7,14)	11 (9,14)	13 (11,15)	10 (7,14)	11 (9,14)	13 (11,15)	10 (9,11)	9 (8,10)	12 (11,13)
Haem/Imm	pri_thrombocytopaenia	Primary or Idiopathic Thrombocytopaenia	26 (21,31)	38 (33,43)	49 (45,54)	26 (21,31)	38 (33,43)	49 (45,54)	13 (12,14)	15 (14,16)	20 (18,21)
Haem/Imm	sarcoid	Sarcoidosis	37 (31,43)	40 (36,46)	48 (43,52)	32 (27,38)	35 (30,39)	42 (38,46)	25 (24,27)	36 (35,38)	39 (37,41)
Haem/Imm	sec_oth_thrombocytopaenia	Secondary or other Thrombocytopaenia	35 (29,41)	49 (44,55)	70 (65,76)	35 (29,41)	49 (44,55)	70 (65,76)	31 (30,33)	44 (42,46)	67 (64,70)
Haem/Imm	sickle_cell	Sickle-cell anaemia	4 (2,7)	--	--	3 (2,6)	--	--	3 (2,3)	--	--
Haem/Imm	sickle_trait	Sickle-cell trait	12 (9,16)	9 (6,11)	4 (3,6)	12 (9,16)	9 (6,11)	4 (3,6)	21 (20,22)	10 (9,11)	5 (4,6)
Haem/Imm	splenomegaly	Splenomegaly	8 (5,11)	15 (12,19)	16 (13,19)	8 (5,11)	15 (12,19)	16 (13,19)	13 (12,14)	17 (16,19)	23 (22,25)
Haem/Imm	thal_trait	Thalassaemia trait	17 (13,21)	12 (10,15)	8 (7,10)	17 (13,21)	12 (10,15)	8 (7,10)	17 (16,18)	14 (12,15)	9 (8,10)
Haem/Imm	thala	Thalassaemia	11 (8,14)	8 (6,11)	5 (4,7)	7 (4,10)	7 (5,9)	4 (3,5)	7 (6,8)	5 (4,6)	4 (4,5)
Haem/Imm	thrombophilia	Thrombophilia	39 (33,46)	35 (30,39)	31 (28,35)	39 (33,45)	34 (30,39)	31 (28,35)	19 (18,20)	14 (13,15)	13 (12,14)
Infections	anorectal	Infection of anal and rectal regions	53 (46,60)	48 (43,54)	32 (28,36)	53 (46,60)	48 (43,54)	32 (28,36)	57 (55,59)	49 (47,52)	37 (35,39)
Infections	bacterial	Bacterial Diseases (excl TB)	875 (847,904)	1019 (994,1043)	1261 (1238,1285)	800 (774,828)	896 (873,919)	1102 (1080,1124)	796 (788,803)	857 (848,865)	1149 (1139,
Infections	bone	Infection of bones and joints	27 (22,33)	41 (36,46)	49 (44,54)	19 (15,24)	27 (23,32)	32 (28,36)	22 (21,23)	32 (31,34)	43 (41,45)

Infections	chr_hep	Chronic viral hepatitis	42 (36,49)	40 (35,45)	24 (21,28)	42 (36,49)	40 (35,45)	24 (21,28)	46 (44,48)	40 (38,42)	22 (21,24)
Infections	digestive	Infections of the digestive system	198 (185,212)	264 (252,277)	326 (315,338)	176 (163,189)	231 (220,243)	293 (282,304)	134 (131,137)	186 (182,190)	276 (271,281)
Infections	ear_urti	Ear and Upper Respiratory Tract Infections	302 (286,320)	313 (300,327)	292 (281,303)	160 (148,173)	138 (129,148)	146 (138,154)	154 (151,158)	132 (128,135)	132 (129,136)
Infections	enceph	Encephalitis	6 (4,9)	--	8 (7,11)	1 (0,2)	--	3 (2,4)	2 (2,3)	--	4 (3,4)
Infections	eye	Eye infections	17 (14,22)	24 (21,28)	28 (25,32)	9 (7,13)	14 (11,17)	17 (15,20)	8 (7,8)	10 (9,11)	15 (14,16)
Infections	heart	Infections of the Heart	3 (2,5)	5 (3,7)	9 (7,11)	3 (2,5)	5 (3,7)	9 (7,11)	5 (5,6)	6 (5,7)	9 (8,10)
Infections	hiv	HIV	23 (19,28)	12 (9,15)	3 (2,5)	22 (17,27)	12 (9,15)	3 (2,4)	21 (20,22)	12 (11,13)	5 (4,6)
Infections	liver	Infection of liver	40 (34,47)	58 (52,64)	53 (48,58)	18 (14,23)	23 (20,28)	17 (14,20)	34 (33,36)	32 (30,33)	22 (21,24)
Infections	lrti	Lower Respiratory Tract Infections	324 (307,342)	454 (437,470)	736 (718,754)	231 (217,246)	312 (299,326)	543 (527,558)	270 (266,274)	416 (410,422)	754 (746,763)
Infections	male_GU	Infection of male genital system	32 (27,39)	30 (26,35)	40 (36,44)	32 (27,39)	30 (26,35)	40 (36,44)	59 (57,62)	57 (54,60)	69 (66,73)
Infections	meningitis	Meningitis	47 (40,54)	48 (43,54)	42 (38,46)	12 (9,16)	6 (5,9)	6 (5,8)	12 (11,13)	8 (7,9)	6 (5,7)
Infections	mycoses	Mycoses	43 (37,50)	50 (45,56)	82 (76,88)	43 (37,50)	50 (45,56)	82 (76,88)	44 (43,46)	59 (57,62)	103 (99,106)
Infections	oth_gu	Infection of other or unspecified genitourinary system	31 (26,36)	19 (16,22)	14 (12,17)	31 (26,36)	19 (16,22)	14 (12,17)	26 (25,27)	15 (14,16)	10 (9,12)
Infections	oth_nerv_sys	Other nervous system infections	10 (7,14)	17 (14,20)	20 (17,23)	8 (6,11)	14 (11,17)	17 (14,20)	8 (7,9)	11 (10,12)	18 (17,20)
Infections	oth_organisms	Other or unspecified infectious organisms	523 (502,546)	647 (627,666)	940 (920,960)	523 (502,546)	647 (627,666)	940 (920,960)	456 (450,462)	600 (593,607)	948 (938,958)

Infections	oth_organs	Infections of Other or unspecified organs	306 (289,323)	379 (364,394)	510 (496,525)	306 (289,323)	379 (364,394)	510 (496,525)	208 (204,212)	230 (226,234)	310 (305,316)
Infections	parasitic	Parasitic infections	26 (22,32)	26 (22,30)	23 (20,27)	10 (7,14)	10 (8,13)	10 (8,13)	12 (11,13)	12 (11,13)	11 (10,12)
Infections	PID	Female pelvic inflammatory disease	166 (155,179)	109 (102,117)	64 (59,70)	164 (153,177)	106 (99,114)	63 (58,68)	386 (379,393)	248 (241,254)	136 (131,141)
Infections	rh_fever	Rheumatic fever	18 (15,23)	46 (41,52)	90 (84,97)	12 (9,16)	31 (27,35)	61 (56,67)	13 (12,14)	33 (31,34)	71 (69,74)
Infections	sepsis	Septicaemia	54 (47,62)	81 (75,89)	139 (131,147)	47 (41,54)	76 (70,83)	131 (123,138)	52 (50,54)	89 (86,91)	169 (165,173)
Infections	skin	Infection of skin and subcutaneous tissues	238 (224,254)	290 (277,303)	329 (317,341)	236 (221,251)	286 (273,299)	325 (313,337)	237 (233,241)	270 (265,275)	343 (337,349)
Infections	TB	Tuberculosis	54 (47,62)	73 (66,80)	144 (136,152)	45 (39,52)	57 (52,64)	103 (96,110)	45 (43,47)	64 (61,66)	107 (103,110)
Infections	uti	Urinary Tract Infections	206 (193,220)	299 (286,312)	447 (433,461)	197 (184,211)	286 (273,299)	435 (422,449)	185 (182,189)	257 (252,262)	479 (472,486)
Infections	viral	Viral diseases (excl chronic hepatitis/HIV)	473 (452,494)	512 (495,530)	415 (402,429)	145 (133,156)	156 (147,166)	152 (144,160)	150 (147,154)	132 (128,135)	136 (133,140)
Musculoskeletal	ank_spond	Ankylosing spondylitis	33 (28,39)	47 (42,52)	55 (50,60)	27 (22,32)	35 (30,40)	38 (34,43)	22 (21,24)	30 (28,31)	38 (36,40)
Musculoskeletal	carpal_tunnel	Carpal tunnel syndrome	404 (385,423)	573 (555,591)	650 (634,667)	391 (373,411)	555 (538,573)	625 (609,641)	427 (421,432)	619 (612,627)	719 (710,727)
Musculoskeletal	collapsed_vert	Collapsed vertebra	16 (12,20)	24 (20,28)	44 (40,48)	7 (5,10)	14 (12,18)	35 (31,39)	8 (8,9)	19 (18,21)	43 (41,45)
Musculoskeletal	entero_arthro	Enteropathic arthropathy	--	--	--	--	--	--	--	--	--
Musculoskeletal	enthesopathy	Enthesopathies & synovial disorders	2133 (2089,2178)	2689 (2650,2729)	2924 (2889,2959)	2127 (2082,2172)	2683 (2643,2723)	2919 (2884,2955)	2260 (2248,	3188 (3171,	3517 (3498,
Musculoskeletal	fibromatosis	Fibromatoses	65 (57,73)	174 (164,185)	336 (324,348)	59 (51,67)	165 (155,176)	327 (315,339)	46 (44,48)	141 (138,145)	288 (283,294)

Musculoskeletal	fracture_hip	Fracture of hip	27 (22,33)	44 (39,50)	85 (79,91)	22 (18,28)	41 (36,46)	80 (75,86)	24 (22,25)	48 (46,51)	110 (107,113)
Musculoskeletal	fracture_wrist	Fracture of wrist	229 (215,245)	295 (282,309)	380 (368,393)	183 (170,196)	244 (233,256)	336 (325,348)	221 (217,225)	266 (261,271)	352 (346,358)
Musculoskeletal	GCA	Giant Cell arteritis	3 (2,5)	9 (7,12)	29 (26,33)	3 (2,5)	9 (7,12)	29 (25,32)	3 (2,3)	10 (9,11)	30 (28,32)
Musculoskeletal	gout	Gout	238 (223,254)	416 (400,433)	638 (621,655)	228 (213,244)	395 (379,411)	610 (594,627)	213 (209,217)	408 (402,414)	643 (635,651)
Musculoskeletal	intervert_disc	Intervertebral disc disorders	540 (518,563)	636 (617,656)	723 (706,741)	436 (416,457)	509 (491,526)	597 (582,613)	396 (391,402)	509 (502,516)	587 (579,595)
Musculoskeletal	juv_arth	Juvenile arthritis	4 (2,6)	3 (2,5)	--	4 (2,6)	3 (2,5)	--	5 (5,6)	3 (3,4)	--
Musculoskeletal	OA	Osteoarthritis (excl spine)	781 (754,808)	1776 (1744,1808)	3073 (3037,3109)	618 (595,643)	1421 (1392,1450)	2540 (2507,2573)	586 (580,593)	1561 (1549,	2868 (2851,
Musculoskeletal	osteoporosis	Osteoporosis	111 (101,121)	342 (329,356)	759 (742,777)	99 (90,109)	316 (303,329)	706 (689,723)	61 (59,63)	228 (224,233)	569 (561,576)
Musculoskeletal	PMR	Polymyalgia Rheumatica	8 (5,11)	41 (36,46)	134 (127,142)	7 (5,10)	37 (33,42)	129 (121,136)	9 (8,9)	40 (39,42)	142 (138,146)
Musculoskeletal	PSA	Psoriatic arthropathy	43 (37,50)	55 (50,61)	45 (41,50)	40 (34,47)	51 (45,56)	41 (37,45)	33 (31,34)	43 (42,45)	45 (43,47)
Musculoskeletal	reactive	Postinfective and reactive arthropathies	10 (7,14)	12 (10,15)	12 (10,15)	10 (7,14)	12 (10,15)	12 (10,15)	14 (13,15)	16 (15,17)	15 (14,16)
Musculoskeletal	RhA	Rheumatoid Arthritis	102 (92,112)	191 (181,202)	264 (253,274)	79 (71,88)	149 (140,158)	211 (202,221)	80 (77,82)	149 (146,153)	240 (235,245)
Musculoskeletal	scoliosis	Scoliosis	57 (50,65)	62 (56,68)	80 (74,86)	52 (46,60)	56 (50,62)	75 (69,80)	59 (57,61)	65 (63,67)	91 (88,94)
Musculoskeletal	sjogren	Sjogren's disease	10 (7,13)	22 (19,26)	27 (24,30)	7 (5,11)	18 (15,21)	22 (19,25)	8 (7,9)	16 (15,17)	26 (24,27)

Musculoskeletal	SLE	Lupus erythematosus (local and systemic)	23 (19,28)	25 (22,29)	28 (25,32)	21 (17,25)	23 (20,27)	26 (22,29)	21 (20,23)	26 (25,28)	28 (27,30)
Musculoskeletal	spinal_stenosis	Spinal stenosis	45 (39,52)	86 (79,93)	179 (170,188)	44 (38,51)	84 (77,91)	176 (167,185)	38 (36,39)	83 (80,85)	164 (160,168)
Musculoskeletal	spondylolisthesis	Spondylolisthesis	39 (33,46)	60 (54,66)	86 (80,92)	28 (23,34)	44 (39,50)	72 (67,78)	25 (24,26)	44 (42,46)	74 (71,77)
Musculoskeletal	spondylosis	Spondylosis	306 (289,323)	604 (586,623)	1078 (1057,1100)	290 (274,307)	568 (550,587)	1033 (1012,1054)	230 (226,234)	546 (539,553)	1018 (1008,
Musculoskeletal	sys_sclerosis	Systemic sclerosis	6 (4,8)	7 (6,10)	11 (9,13)	5 (3,7)	7 (5,9)	9 (7,11)	4 (3,4)	7 (6,8)	10 (9,11)
Neurological	autonomic_neuro	Disorders of autonomic nervous system	15 (11,19)	27 (24,32)	32 (28,36)	15 (11,19)	27 (24,32)	32 (28,36)	24 (22,25)	34 (32,35)	46 (44,48)
Neurological	bells	Bell's palsy	68 (60,76)	75 (68,82)	84 (78,90)	64 (57,72)	70 (64,77)	79 (74,85)	79 (77,81)	100 (97,103)	118 (114,121)
Neurological	cerebral_palsy	Cerebral Palsy	11 (8,15)	10 (7,13)	6 (4,8)	10 (7,13)	9 (7,12)	5 (4,7)	14 (13,15)	12 (11,13)	10 (9,11)
Neurological	chronic_fatigue	Postviral fatigue syndrome, neurasthenia and fibromyalgia	269 (254,285)	286 (273,299)	248 (238,259)	250 (235,265)	264 (252,276)	231 (221,241)	209 (206,213)	263 (258,268)	238 (233,243)
Neurological	dm_neuro	Diabetic neurological complications	9 (6,12)	27 (23,31)	42 (38,47)	8 (6,12)	25 (21,29)	41 (37,46)	18 (17,20)	45 (43,47)	84 (81,87)
Neurological	epilepsy	Epilepsy	166 (154,179)	168 (158,179)	166 (157,174)	159 (147,171)	161 (152,171)	159 (151,168)	198 (195,202)	201 (197,205)	212 (208,217)
Neurological	essential_tremor	Essential tremor	19 (15,23)	25 (22,30)	56 (51,61)	16 (13,21)	24 (20,28)	52 (47,57)	12 (11,13)	19 (17,20)	44 (42,46)
Neurological	intracranial_htn	Intracranial hypertension	5 (3,8)	5 (3,7)	2 (2,4)	5 (3,8)	5 (3,7)	2 (2,4)	6 (5,6)	5 (4,5)	3 (2,4)
Neurological	migraine	Migraine	1194 (1161,1227)	1074 (1050,1099)	890 (871,909)	1052 (1022,1083)	944 (922,967)	783 (765,801)	945 (937,953)	895 (886,903)	778 (769,786)

Neurological	MND	Motor neuron disease	1 (1,3)	7 (5,10)	8 (7,10)	1 (1,3)	6 (5,9)	8 (6,10)	2 (2,3)	4 (4,5)	10 (9,11)
Neurological	MS	Multiple sclerosis	53 (46,60)	60 (54,66)	41 (37,45)	50 (44,58)	59 (53,65)	39 (35,43)	38 (37,40)	49 (47,51)	51 (49,53)
Neurological	myasthenia	Myasthenia gravis	3 (2,5)	7 (5,9)	9 (7,11)	3 (2,5)	7 (5,9)	9 (7,11)	3 (2,3)	4 (4,5)	9 (8,9)
Neurological	Parkinsons	Parkinson's disease	6 (4,9)	23 (20,27)	74 (68,80)	5 (3,8)	23 (19,27)	73 (68,79)	4 (4,5)	18 (17,20)	70 (67,73)
Neurological	periph_neuro	Peripheral neuropathies (excl. cranial nerve, carpal tunnel syndromes)	216 (202,231)	323 (309,337)	387 (374,400)	209 (195,224)	313 (300,327)	375 (363,388)	211 (208,215)	326 (321,331)	432 (425,439)
Neurological	trigem_neur	Trigeminal neuralgia	44 (38,51)	71 (65,78)	91 (85,97)	43 (37,50)	69 (63,76)	89 (83,95)	51 (49,53)	80 (78,83)	108 (104,111)
Perinatal	congenital_septal	Congenital malformations of cardiac septa	32 (27,38)	25 (21,29)	22 (19,26)	32 (26,38)	24 (20,28)	22 (19,25)	27 (25,28)	24 (22,25)	22 (21,24)
Perinatal	downs	Down's syndrome	1 (0,3)	1 (1,3)	0 (0,1)	1 (0,3)	1 (1,3)	0 (0,1)	10 (9,11)	8 (7,9)	5 (4,5)
Perinatal	HBW	High birth weight	--	--	--	--	--	--	--	--	--
Perinatal	intrauterine_hypoxia	Intrauterine hypoxia	--	--	--	--	--	--	--	--	--
Perinatal	LBW	Slow fetal growth or low birth weight	--	--	--	--	--	--	--	--	--
Perinatal	neo_jaundice	Neonatal jaundice (excl haemolytic dz of the newborn)	--	--	--	--	--	--	--	--	--
Perinatal	PDA	Patent ductus arteriosus	4 (2,6)	3 (2,5)	3 (2,4)	4 (2,6)	3 (2,5)	3 (2,4)	4 (3,4)	3 (3,4)	3 (2,3)
Perinatal	post_term	Post-term infant	--	--	--	--	--	--	--	--	--
Perinatal	prematurity	Prematurity	52 (45,59)	12 (9,15)	--	52 (45,59)	12 (9,15)	--	12 (11,13)	4 (3,4)	--
Perinatal	RDN	Respiratory distress of newborn	--	--	--	--	--	--	--	--	--

Perinatal	sepsis_newborn	Bacterial sepsis of newborn	--	--	--	--	--	--	--	--	--
Perinatal	spina_bifida	Spina bifida	21 (17,26)	18 (14,21)	14 (12,17)	19 (15,24)	17 (14,20)	14 (11,16)	18 (16,19)	16 (15,17)	14 (12,15)
Psychiatric	ADHD	Hyperkinetic disorders	2 (1,4)	1 (0,2)	1 (0,2)	2 (1,4)	1 (0,2)	1 (0,2)	5 (5,6)	3 (3,4)	--
Psychiatric	alc_problems	Alcohol Problems	417 (397,438)	494 (476,511)	456 (442,470)	414 (394,434)	489 (472,507)	453 (439,467)	436 (431,441)	467 (461,474)	434 (428,441)
Psychiatric	anxiety	Anxiety disorders	2085 (2042,2129)	2153 (2118,2189)	1845 (1818,1873)	1087 (1056,1119)	1118 (1093,1144)	1091 (1070,1113)	1704 (1694,	1773 (1761,	1744 (1730,
Psychiatric	autism	Autism and Asperger's syndrome	14 (10,18)	7 (5,10)	4 (3,5)	6 (4,9)	3 (1,4)	1 (1,2)	12 (11,13)	8 (7,8)	4 (3,4)
Psychiatric	BAD	Bipolar affective disorder and mania	95 (85,104)	86 (79,93)	76 (70,82)	51 (44,58)	49 (44,54)	51 (47,56)	66 (64,68)	62 (60,64)	59 (56,61)
Psychiatric	delirium	Delirium, not induced by alcohol and other psychoactive substances	5 (3,7)	10 (7,12)	25 (22,28)	5 (3,7)	10 (7,12)	25 (22,28)	8 (7,8)	17 (16,18)	47 (45,49)
Psychiatric	dementia	Dementia	8 (5,11)	26 (22,30)	100 (94,107)	7 (5,11)	25 (21,29)	99 (93,106)	6 (5,7)	27 (25,28)	130 (126,134)
Psychiatric	depression	Depression	3563 (3506,3620)	3604 (3558,3649)	2973 (2938,3009)	1499 (1462,1537)	1472 (1443,1501)	1307 (1284,1331)	2492 (2479,	2510 (2496,	2277 (2262,
Psychiatric	eating_dz	Anorexia and bulimia nervosa	49 (43,56)	25 (22,29)	14 (11,16)	41 (36,48)	21 (17,24)	12 (10,14)	50 (48,52)	28 (26,29)	12 (11,14)
Psychiatric	intell_dz	Intellectual disability	17 (14,22)	12 (10,16)	4 (3,6)	17 (14,22)	12 (10,16)	4 (3,6)	70 (68,72)	66 (63,68)	53 (50,55)
Psychiatric	ocd	Obsessive-compulsive disorder	63 (55,71)	47 (42,53)	32 (29,36)	35 (30,42)	30 (26,35)	21 (18,24)	58 (56,60)	47 (45,49)	35 (33,37)
Psychiatric	PD	Personality disorders	43 (37,50)	35 (31,40)	26 (23,30)	43 (37,50)	35 (31,40)	26 (23,30)	95 (92,97)	81 (78,83)	72 (69,75)

Psychiatric	schizo	Schizophrenia, schizotypal and delusional disorders	64 (56,72)	47 (42,53)	39 (35,43)	62 (54,70)	45 (40,51)	37 (34,42)	102 (100,105)	97 (95,100)	91 (88,94)
Psychiatric	substance _misuse	Other psychoactive substance misuse	83 (75,93)	55 (49,61)	41 (37,46)	83 (74,92)	54 (49,61)	41 (37,45)	225 (221,229)	128 (124,131)	95 (92,98)
Respiratory	allergic_rhi nitis	Allergic and chronic rhinitis	3525 (3468,3583)	3194 (3151,32 38)	2735 (2702,2770)	1367 (1332,1404)	1320 (1292,1348)	1296 (1273,1320)	1602 (1591,161 2)	1517 (1506,152 9)	1492 (1480,150 4)
Respiratory	asbestosis	Asbestosis	--	4 (2,5)	18 (16,22)	--	2 (1,4)	15 (13,18)	--	4 (4,5)	25 (24,27)
Respiratory	aspiration_ pneumo	Aspiration pneumonitis	6 (4,9)	13 (10,16)	21 (18,24)	6 (4,9)	13 (10,16)	21 (18,24)	13 (12,14)	22 (21,24)	49 (47,51)
Respiratory	asthma	Asthma	1704 (1664,1744)	1553 (1523,15 83)	1460 (1435,1485)	1332 (1297,1367)	1226 (1199,1253)	1201 (1179,1224)	1408 (1398,141 8)	1333 (1322,134 4)	1362 (1351,137 4)
Respiratory	bronchiect asis	Bronchiectasis	34 (29,41)	65 (59,71)	149 (141,157)	26 (21,31)	55 (49,61)	134 (126,141)	21 (20,22)	52 (49,54)	128 (125,132)
Respiratory	COPD	COPD	97 (88,107)	261 (249,274)	557 (541,572)	89 (80,99)	247 (235,260)	536 (521,552)	127 (124,130)	374 (368,379)	826 (817,835)
Respiratory	hyper_nas al_turbs	Hypertrophy of nasal turbinates	57 (50,65)	50 (45,56)	40 (36,44)	57 (50,65)	50 (45,56)	40 (36,44)	49 (47,51)	44 (42,46)	35 (34,37)
Respiratory	nasal_poly p	Nasal polyp	147 (135,159)	193 (182,204)	258 (247,269)	130 (119,142)	172 (162,183)	229 (219,239)	104 (101,106)	148 (144,152)	205 (201,210)
Respiratory	pleural_eff usion	Pleural effusion	47 (41,54)	93 (85,101)	171 (163,180)	47 (40,54)	93 (85,101)	171 (162,180)	64 (62,66)	123 (119,126)	250 (245,255)
Respiratory	pleural_pla que	Pleural plaque	--	10 (7,13)	46 (41,50)	--	9 (7,12)	43 (39,48)	--	9 (8,9)	42 (40,45)
Respiratory	pneumoth orax	Pneumothorax	57 (49,64)	66 (60,73)	73 (67,79)	44 (38,51)	47 (42,53)	56 (51,61)	49 (47,51)	59 (56,61)	75 (72,78)
Respiratory	pulm_colla pse	Pulmonary collapse (excl pneumothorax)	18 (14,23)	38 (33,43)	64 (59,69)	18 (14,23)	38 (33,43)	64 (59,69)	28 (27,30)	56 (54,58)	101 (98,104)

Respiratory	pulm_fibrosis	Other interstitial pulmonary diseases with fibrosis	8 (5,11)	15 (13,19)	51 (46,56)	7 (4,10)	12 (10,15)	44 (40,49)	6 (5,7)	16 (14,17)	45 (43,47)
Respiratory	resp_failure	Respiratory failure	15 (12,20)	35 (30,40)	69 (64,75)	15 (12,20)	34 (29,38)	69 (64,75)	29 (28,31)	64 (61,66)	147 (143,150)
Respiratory	sinusitis	Chronic sinusitis	1251 (1218,1285)	1309 (1281,1336)	1312 (1288,1335)	1222 (1189,1256)	1266 (1240,1294)	1268 (1245,1291)	285 (280,289)	371 (365,376)	393 (387,399)
Respiratory	sleep_apnoea	Sleep apnoea	157 (145,170)	211 (200,223)	206 (197,216)	153 (141,166)	203 (192,215)	199 (190,209)	122 (119,124)	178 (174,182)	185 (181,190)
Skin	acne	Acne	498 (477,520)	259 (247,272)	130 (122,137)	488 (467,509)	252 (240,264)	125 (117,132)	633 (626,639)	332 (326,337)	153 (149,157)
Skin	alopecia_areata	Alopecia areata	45 (39,52)	40 (36,45)	34 (30,38)	43 (37,49)	38 (34,43)	32 (28,36)	49 (47,51)	46 (44,48)	38 (36,40)
Skin	dermatitis	Dermatitis (atopc/contact/other/unspecified)	1904 (1862,1946)	1913 (1880,1947)	2022 (1993,2052)	1737 (1697,1777)	1757 (1726,1790)	1903 (1874,1931)	2136 (2124,2148)	2290 (2276,2305)	2480 (2464,2496)
Skin	hidradenitis	Hidradenitis suppurativa	24 (19,29)	16 (13,19)	9 (7,11)	24 (19,29)	16 (13,19)	9 (7,11)	48 (46,49)	37 (35,39)	20 (19,22)
Skin	keratitis	Keratitis	63 (55,71)	68 (62,75)	82 (76,88)	63 (55,71)	68 (62,75)	82 (76,88)	78 (76,80)	239 (235,244)	586 (578,594)
Skin	lichen_planus	Lichen planus	66 (59,75)	99 (92,107)	142 (134,150)	66 (58,74)	98 (91,106)	140 (132,148)	52 (50,54)	94 (91,97)	133 (130,137)
Skin	pilonidal	Pilonidal cyst/sinus	108 (98,119)	78 (71,85)	57 (52,63)	108 (98,119)	78 (71,85)	57 (52,63)	120 (118,123)	89 (86,92)	71 (69,74)
Skin	psoriasis	Psoriasis	380 (362,400)	414 (398,430)	443 (430,457)	346 (328,365)	376 (362,392)	411 (398,424)	409 (404,414)	464 (458,470)	521 (513,528)
Skin	rosacea	Rosacea	341 (324,359)	398 (383,414)	418 (405,432)	337 (319,355)	396 (381,411)	415 (402,428)	312 (307,317)	388 (383,394)	417 (411,423)
Skin	seb_derm	Seborrheic dermatitis	896 (867,925)	822 (800,845)	790 (772,809)	567 (545,591)	547 (529,565)	575 (559,591)	440 (435,446)	504 (497,511)	579 (572,587)

Skin	urticaria	Urticaria	404 (385,424)	415 (399,430)	427 (414,440)	398 (380,418)	409 (394,425)	422 (409,436)	489 (483,494)	509 (503,516)	517 (509,524)
Skin	vitiligo	Vitiligo	40 (34,46)	39 (34,44)	39 (35,43)	39 (33,45)	37 (32,42)	36 (33,41)	41 (39,43)	42 (40,44)	43 (41,45)

Table S8. Sex-standardised period prevalence per 10,000 persons obtained in UKB in any source, in UKB in EHR stratified by country of assessment centre

group	phenotype	variable_name	UKB_EHR_4049	UKB_EHR_5059	UKB_EHR_6069	country
Benign neoplasm or Carcinoma in situ	Benign neoplasm and polyp of uterus	benign_uterus	196(152,250)	283(242,329)	259(223,298)	W
Benign neoplasm or Carcinoma in situ	Benign neoplasm and polyp of uterus	benign_uterus	69(49,94)	148(125,174)	165(142,189)	S
Benign neoplasm or Carcinoma in situ	Benign neoplasm and polyp of uterus	benign_uterus	182(168,196)	234(222,247)	255(244,267)	E
Benign neoplasm or Carcinoma in situ	Benign neoplasm of brain and other parts of central nervous system	benign_brain	22(9,46)	34(20,53)	60(43,81)	W
Benign neoplasm or Carcinoma in situ	Benign neoplasm of brain and other parts of central nervous system	benign_brain	48(31,72)	48(35,65)	67(52,84)	S
Benign neoplasm or Carcinoma in situ	Benign neoplasm of brain and other parts of central nervous system	benign_brain	32(27,39)	40(35,46)	50(45,56)	E
Benign neoplasm or Carcinoma in situ	Benign neoplasm of colon, rectum, anus and anal canal	benign_colon	254(199,319)	482(424,546)	835(768,907)	W

Benign neoplasm or Carcinoma in situ	Benign neoplasm of colon, rectum, anus and anal canal	benign_colon	262(218,312)	540(492,592)	911(854,971)	S
Benign neoplasm or Carcinoma in situ	Benign neoplasm of colon, rectum, anus and anal canal	benign_colon	264(246,282)	548(527,568)	910(888,932)	E
Benign neoplasm or Carcinoma in situ	Benign neoplasm of ovary	benign_ovary	299(243,364)	242(204,284)	177(148,210)	W
Benign neoplasm or Carcinoma in situ	Benign neoplasm of ovary	benign_ovary	284(242,331)	256(226,289)	194(170,221)	S
Benign neoplasm or Carcinoma in situ	Benign neoplasm of ovary	benign_ovary	290(273,309)	243(231,256)	191(181,201)	E
Benign neoplasm or Carcinoma in situ	Benign neoplasm of stomach and duodenum	benign_stomach	29(13,56)	77(56,105)	155(127,187)	W
Benign neoplasm or Carcinoma in situ	Benign neoplasm of stomach and duodenum	benign_stomach	28(15,48)	65(50,85)	109(91,131)	S
Benign neoplasm or Carcinoma in situ	Benign neoplasm of stomach and duodenum	benign_stomach	46(39,54)	108(99,117)	190(180,200)	E
Benign neoplasm or	Carcinoma in situ_cervical	cin_cervical	891(792,999)	775(706,849)	581(527,638)	W

Carcinoma in situ						
Benign neoplasm or Carcinoma in situ	Carcinoma in situ_cervical	cin_cervical	285(243,332)	220(192,251)	178(155,203)	S
Benign neoplasm or Carcinoma in situ	Carcinoma in situ_cervical	cin_cervical	1631(1590,1673)	1523(1492,1555)	1260(1235,1285)	E
Benign neoplasm or Carcinoma in situ	Haemangioma, any site	haemangioma	217(168,276)	273(230,321)	228(194,266)	W
Benign neoplasm or Carcinoma in situ	Haemangioma, any site	haemangioma	128(99,163)	165(139,193)	142(121,167)	S
Benign neoplasm or Carcinoma in situ	Haemangioma, any site	haemangioma	134(122,147)	162(152,174)	183(173,193)	E
Benign neoplasm or Carcinoma in situ	Leiomyoma of uterus	leiomyoma	486(414,568)	485(431,544)	352(310,397)	W
Benign neoplasm or Carcinoma in situ	Leiomyoma of uterus	leiomyoma	337(292,388)	447(406,490)	386(352,423)	S
Benign neoplasm or Carcinoma in situ	Leiomyoma of uterus	leiomyoma	518(495,542)	524(506,543)	389(375,403)	E

Cancers	Hodgkin Lymphoma	hodgkins	7(1,27)	5(1,16)	9(3,19)	W
Cancers	Hodgkin Lymphoma	hodgkins	14(5,29)	13(7,22)	8(4,16)	S
Cancers	Hodgkin Lymphoma	hodgkins	13(10,18)	12(9,16)	13(10,16)	E
Cancers	Leukaemia	leukaemia	7(1,24)	21(10,37)	54(38,74)	W
Cancers	Leukaemia	leukaemia	13(5,28)	18(10,30)	47(35,62)	S
Cancers	Leukaemia	leukaemia	13(10,18)	25(21,30)	46(41,51)	E
Cancers	Monoclonal gammopathy of undetermined significance (MGUS)	MGUS	4(0,21)	23(12,41)	31(19,47)	W
Cancers	Monoclonal gammopathy of undetermined significance (MGUS)	MGUS	6(1,19)	3(1,10)	30(20,42)	S
Cancers	Monoclonal gammopathy of undetermined significance (MGUS)	MGUS	4(2,7)	12(9,16)	30(26,34)	E
Cancers	Multiple myeloma and malignant plasma cell neoplasms	plasmacell	6(1,22)	15(7,30)	25(14,40)	W
Cancers	Multiple myeloma and malignant plasma cell neoplasms	plasmacell	13(5,28)	9(4,17)	17(10,26)	S
Cancers	Multiple myeloma and malignant plasma cell neoplasms	plasmacell	6(3,9)	11(8,14)	25(22,29)	E
Cancers	Myelodysplastic syndromes	MDS	0(NaN,NaN)	5(1,15)	12(5,23)	W
Cancers	Myelodysplastic syndromes	MDS	0(NaN,NaN)	6(2,14)	9(4,16)	S
Cancers	Myelodysplastic syndromes	MDS	3(2,6)	6(4,9)	8(6,11)	E
Cancers	Non-Hodgkin Lymphoma	NHL	13(3,33)	35(21,55)	64(47,86)	W
Cancers	Non-Hodgkin Lymphoma	NHL	26(13,45)	44(32,61)	69(55,87)	S
Cancers	Non-Hodgkin Lymphoma	NHL	25(20,31)	38(33,44)	81(75,88)	E
Cancers	Polycythaemia vera	PCV	24(10,49)	35(20,56)	43(28,61)	W
Cancers	Polycythaemia vera	PCV	0(NaN,NaN)	12(6,22)	14(7,23)	S
Cancers	Polycythaemia vera	PCV	4(2,7)	11(8,14)	16(13,19)	E

Cancers	Primary Malignancy_Adrenal gland	pri_adrenal	0(NaN,NaN)	2(0,12)	1(0,8)	W
Cancers	Primary Malignancy_Adrenal gland	pri_adrenal	2(0,10)	0(NaN,NaN)	3(1,9)	S
Cancers	Primary Malignancy_Adrenal gland	pri_adrenal	1(0,2)	2(1,4)	1(0,2)	E
Cancers	Primary Malignancy_biliary tract	pri_biliary	0(NaN,NaN)	6(1,18)	9(3,19)	W
Cancers	Primary Malignancy_biliary tract	pri_biliary	0(NaN,NaN)	4(1,10)	14(8,23)	S
Cancers	Primary Malignancy_biliary tract	pri_biliary	2(0,4)	4(2,6)	10(8,12)	E
Cancers	Primary Malignancy_Bladder	pri_bladder	10(2,31)	33(19,53)	83(63,108)	W
Cancers	Primary Malignancy_Bladder	pri_bladder	10(3,24)	29(18,43)	67(52,85)	S
Cancers	Primary Malignancy_Bladder	pri_bladder	10(7,14)	30(25,35)	91(84,98)	E
Cancers	Primary Malignancy_Bone and articular cartilage	pri_bone	3(0,17)	10(3,24)	10(4,20)	W
Cancers	Primary Malignancy_Bone and articular cartilage	pri_bone	6(1,19)	7(2,15)	5(1,11)	S
Cancers	Primary Malignancy_Bone and articular cartilage	pri_bone	2(1,5)	4(2,6)	6(4,8)	E
Cancers	Primary Malignancy_Brain, Other CNS and Intracranial	pri_brain	24(10,49)	16(7,31)	18(9,31)	W
Cancers	Primary Malignancy_Brain, Other CNS and Intracranial	pri_brain	6(1,17)	10(5,19)	21(13,32)	S
Cancers	Primary Malignancy_Brain, Other CNS and Intracranial	pri_brain	9(6,13)	15(11,18)	16(14,20)	E
Cancers	Primary Malignancy_Breast	pri_breast	133(97,178)	240(203,283)	416(370,465)	W
Cancers	Primary Malignancy_Breast	pri_breast	159(128,195)	239(209,271)	397(362,434)	S
Cancers	Primary Malignancy_Breast	pri_breast	158(146,172)	266(253,280)	406(392,420)	E
Cancers	Primary Malignancy_Cervical	pri_cervical	12(3,31)	13(6,26)	15(7,27)	W
Cancers	Primary Malignancy_Cervical	pri_cervical	26(15,43)	22(14,33)	17(11,27)	S
Cancers	Primary Malignancy_Cervical	pri_cervical	13(10,17)	16(13,19)	15(12,18)	E
Cancers	Primary Malignancy_colorectal and anus	pri_bowel	22(8,48)	103(77,135)	202(169,239)	W

Cancers	Primary Malignancy_colorectal and anus	pri_bowel	37(22,58)	98(78,121)	199(173,228)	S
Cancers	Primary Malignancy_colorectal and anus	pri_bowel	26(21,32)	84(77,93)	174(165,184)	E
Cancers	Primary Malignancy_Kidney and Ureter	pri_kidney	7(1,24)	20(9,36)	52(36,72)	W
Cancers	Primary Malignancy_Kidney and Ureter	pri_kidney	12(4,25)	26(16,39)	57(43,74)	S
Cancers	Primary Malignancy_Kidney and Ureter	pri_kidney	9(6,12)	25(20,29)	44(39,49)	E
Cancers	Primary Malignancy_Liver	pri_liver	0(NaN,NaN)	2(0,12)	16(8,29)	W
Cancers	Primary Malignancy_Liver	pri_liver	0(NaN,NaN)	3(1,10)	8(3,15)	S
Cancers	Primary Malignancy_Liver	pri_liver	1(0,3)	5(3,8)	8(6,10)	E
Cancers	Primary Malignancy_Lung and trachea	pri_lung	10(2,29)	24(13,41)	77(58,101)	W
Cancers	Primary Malignancy_Lung and trachea	pri_lung	9(3,20)	37(25,52)	75(60,93)	S
Cancers	Primary Malignancy_Lung and trachea	pri_lung	8(6,12)	32(27,37)	65(59,71)	E
Cancers	Primary Malignancy_Lymph Nodes	pri_LN	0(NaN,NaN)	0(NaN,NaN)	0(NaN,NaN)	W
Cancers	Primary Malignancy_Lymph Nodes	pri_LN	0(NaN,NaN)	0(NaN,NaN)	1(0,5)	S
Cancers	Primary Malignancy_Lymph Nodes	pri_LN	1(0,3)	1(0,2)	3(2,4)	E
Cancers	Primary Malignancy_Malignant Melanoma	pri_melanoma	64(39,99)	141(111,177)	153(126,186)	W
Cancers	Primary Malignancy_Malignant Melanoma	pri_melanoma	74(52,102)	102(82,126)	144(122,168)	S
Cancers	Primary Malignancy_Malignant Melanoma	pri_melanoma	64(56,74)	83(75,91)	125(117,133)	E
Cancers	Primary Malignancy_Mesothelioma	pri_mesothelioma	3(0,17)	2(0,12)	9(3,20)	W
Cancers	Primary Malignancy_Mesothelioma	pri_mesothelioma	0(NaN,NaN)	0(NaN,NaN)	6(2,13)	S
Cancers	Primary Malignancy_Mesothelioma	pri_mesothelioma	0(0,2)	1(0,3)	7(5,9)	E

Cancers	Primary Malignancy_Multiple independent sites	pri_multindep	3(0,17)	2(0,9)	7(2,17)	W
Cancers	Primary Malignancy_Multiple independent sites	pri_multindep	0(NaN,NaN)	0(NaN,NaN)	1(0,5)	S
Cancers	Primary Malignancy_Multiple independent sites	pri_multindep	1(0,2)	1(0,2)	3(2,5)	E
Cancers	Primary Malignancy_Oesophageal	pri_oesoph	0(NaN,NaN)	12(5,27)	23(13,37)	W
Cancers	Primary Malignancy_Oesophageal	pri_oesoph	5(1,17)	6(2,14)	20(13,31)	S
Cancers	Primary Malignancy_Oesophageal	pri_oesoph	2(1,4)	11(8,14)	25(22,29)	E
Cancers	Primary Malignancy_Oro-pharyngeal	pri_oroph	4(0,21)	36(21,57)	42(28,61)	W
Cancers	Primary Malignancy_Oro-pharyngeal	pri_oroph	23(11,41)	44(31,61)	46(34,61)	S
Cancers	Primary Malignancy_Oro-pharyngeal	pri_oroph	13(10,18)	22(18,27)	33(29,37)	E
Cancers	Primary Malignancy_Other Organs	pri_other	58(34,93)	131(102,166)	166(137,200)	W
Cancers	Primary Malignancy_Other Organs	pri_other	25(13,44)	76(59,97)	160(136,186)	S
Cancers	Primary Malignancy_Other Organs	pri_other	37(31,44)	81(73,89)	139(130,147)	E
Cancers	Primary Malignancy_Other Skin and subcutaneous tissue	pri_skin	218(168,277)	471(414,534)	1001(927,1078)	W
Cancers	Primary Malignancy_Other Skin and subcutaneous tissue	pri_skin	181(146,222)	368(329,411)	810(757,866)	S
Cancers	Primary Malignancy_Other Skin and subcutaneous tissue	pri_skin	155(142,169)	326(311,342)	727(707,747)	E
Cancers	Primary Malignancy_Ovarian	pri_ovarian	24(10,48)	20(10,35)	53(38,73)	W
Cancers	Primary Malignancy_Ovarian	pri_ovarian	7(2,18)	22(14,33)	40(29,53)	S
Cancers	Primary Malignancy_Ovarian	pri_ovarian	17(13,21)	29(24,33)	41(37,46)	E
Cancers	Primary Malignancy_Pancreatic	pri_pancr	0(NaN,NaN)	4(0,14)	29(18,45)	W
Cancers	Primary Malignancy_Pancreatic	pri_pancr	5(1,15)	6(2,15)	18(10,28)	S

Cancers	Primary Malignancy_Pancreatic	pri_pancr	3(1,5)	8(6,11)	19(16,23)	E
Cancers	Primary Malignancy_Prostate	pri_prost	22(8,48)	99(73,133)	340(296,387)	W
Cancers	Primary Malignancy_Prostate	pri_prost	7(1,20)	77(59,99)	315(280,352)	S
Cancers	Primary Malignancy_Prostate	pri_prost	8(5,12)	92(83,101)	315(302,329)	E
Cancers	Primary Malignancy_Stomach	pri_stomach	3(0,17)	21(10,38)	21(11,35)	W
Cancers	Primary Malignancy_Stomach	pri_stomach	4(0,15)	12(6,23)	24(15,36)	S
Cancers	Primary Malignancy_Stomach	pri_stomach	3(1,5)	9(7,13)	21(18,24)	E
Cancers	Primary Malignancy_Testicular	pri_testis	26(10,53)	11(4,25)	8(2,18)	W
Cancers	Primary Malignancy_Testicular	pri_testis	32(18,54)	28(18,43)	12(6,21)	S
Cancers	Primary Malignancy_Testicular	pri_testis	24(19,31)	17(14,22)	11(9,14)	E
Cancers	Primary Malignancy_Thyroid	pri_thyroid	10(2,29)	4(0,14)	18(10,32)	W
Cancers	Primary Malignancy_Thyroid	pri_thyroid	9(3,22)	12(6,21)	16(9,25)	S
Cancers	Primary Malignancy_Thyroid	pri_thyroid	11(8,15)	17(14,21)	14(11,17)	E
Cancers	Primary Malignancy_Uterine	pri_uterine	12(3,31)	27(15,43)	67(49,88)	W
Cancers	Primary Malignancy_Uterine	pri_uterine	16(7,30)	31(21,44)	54(41,69)	S
Cancers	Primary Malignancy_Uterine	pri_uterine	8(5,11)	24(20,28)	51(46,56)	E
Cancers	Secondary Malignancy_Adrenal gland	sec_adrenal	0(NaN,NaN)	4(0,14)	7(2,17)	W
Cancers	Secondary Malignancy_Adrenal gland	sec_adrenal	0(NaN,NaN)	0(NaN,NaN)	7(3,15)	S
Cancers	Secondary Malignancy_Adrenal gland	sec_adrenal	1(0,3)	5(3,7)	9(7,11)	E
Cancers	Secondary Malignancy_Bone	sec_bone	19(7,41)	37(22,57)	71(53,94)	W
Cancers	Secondary Malignancy_Bone	sec_bone	9(3,20)	21(12,32)	56(42,72)	S
Cancers	Secondary Malignancy_Bone	sec_bone	14(10,19)	35(30,40)	66(61,73)	E
Cancers	Secondary Malignancy_Bowel	sec_bowel	3(0,17)	2(0,9)	12(5,23)	W
Cancers	Secondary Malignancy_Bowel	sec_bowel	0(NaN,NaN)	4(1,11)	4(1,10)	S
Cancers	Secondary Malignancy_Bowel	sec_bowel	2(1,4)	4(2,6)	6(4,8)	E

Cancers	Secondary Malignancy_Brain, Other CNS and Intracranial	sec_brain	6(1,22)	11(4,25)	27(16,43)	W
Cancers	Secondary Malignancy_Brain, Other CNS and Intracranial	sec_brain	2(0,13)	13(6,23)	21(13,31)	S
Cancers	Secondary Malignancy_Brain, Other CNS and Intracranial	sec_brain	6(4,10)	13(10,16)	24(20,27)	E
Cancers	Secondary malignancy_Liver and intrahepatic bile duct	sec_liver	21(8,45)	48(31,71)	78(58,101)	W
Cancers	Secondary malignancy_Liver and intrahepatic bile duct	sec_liver	10(3,23)	34(23,48)	58(45,75)	S
Cancers	Secondary malignancy_Liver and intrahepatic bile duct	sec_liver	17(13,22)	39(34,45)	72(66,79)	E
Cancers	Secondary Malignancy_Lung	sec_lung	26(11,50)	32(18,51)	46(32,65)	W
Cancers	Secondary Malignancy_Lung	sec_lung	8(2,21)	30(20,44)	55(42,71)	S
Cancers	Secondary Malignancy_Lung	sec_lung	12(8,16)	28(24,33)	58(53,64)	E
Cancers	Secondary Malignancy_Lymph Nodes	sec_LN	49(28,80)	122(94,155)	184(153,218)	W
Cancers	Secondary Malignancy_Lymph Nodes	sec_LN	55(37,78)	106(86,130)	147(126,172)	S
Cancers	Secondary Malignancy_Lymph Nodes	sec_LN	62(54,71)	108(99,117)	159(150,168)	E
Cancers	Secondary Malignancy_Other organs	sec_other	22(9,45)	37(23,58)	78(58,101)	W
Cancers	Secondary Malignancy_Other organs	sec_other	19(9,35)	42(30,57)	77(62,96)	S
Cancers	Secondary Malignancy_Other organs	sec_other	18(14,23)	35(30,40)	60(54,66)	E
Cancers	Secondary Malignancy_Pleura	sec_pleura	3(0,17)	9(3,21)	12(5,23)	W
Cancers	Secondary Malignancy_Pleura	sec_pleura	2(0,10)	7(2,15)	15(9,25)	S
Cancers	Secondary Malignancy_Pleura	sec_pleura	3(2,6)	10(7,13)	15(12,18)	E
Cancers	Secondary Malignancy_retroperitoneum and peritoneum	sec_peritoneum	10(2,29)	29(17,47)	47(32,66)	W
Cancers	Secondary Malignancy_retroperitoneum and peritoneum	sec_peritoneum	7(2,19)	12(6,21)	37(27,51)	S

Cancers	Secondary Malignancy_retroperitoneum and peritoneum	sec_peritoneum	10(7,13)	21(18,26)	35(31,40)	E
Cardiovascular	Abdominal aortic aneurysm	AAA	11(2,32)	9(2,22)	44(30,64)	W
Cardiovascular	Abdominal aortic aneurysm	AAA	0(NaN,NaN)	12(6,22)	37(26,51)	S
Cardiovascular	Abdominal aortic aneurysm	AAA	3(1,6)	11(8,14)	46(41,51)	E
Cardiovascular	Atrial fibrillation	AF	89(58,132)	236(196,283)	674(613,739)	W
Cardiovascular	Atrial fibrillation	AF	77(54,107)	222(191,257)	646(598,697)	S
Cardiovascular	Atrial fibrillation	AF	88(78,98)	242(228,256)	667(649,687)	E
Cardiovascular	Atrioventricular block, complete	av_block_3	3(0,17)	12(4,26)	39(26,57)	W
Cardiovascular	Atrioventricular block, complete	av_block_3	7(2,18)	0(NaN,NaN)	13(7,22)	S
Cardiovascular	Atrioventricular block, complete	av_block_3	4(2,6)	6(4,9)	23(20,27)	E
Cardiovascular	Atrioventricular block, first degree	av_block_1	7(1,27)	10(3,23)	29(18,45)	W
Cardiovascular	Atrioventricular block, first degree	av_block_1	6(1,17)	18(10,30)	35(25,49)	S
Cardiovascular	Atrioventricular block, first degree	av_block_1	10(7,14)	22(18,27)	52(46,57)	E
Cardiovascular	Atrioventricular block, second degree	av_block_2	0(NaN,NaN)	6(1,18)	17(8,30)	W
Cardiovascular	Atrioventricular block, second degree	av_block_2	6(1,17)	5(1,12)	16(9,25)	S
Cardiovascular	Atrioventricular block, second degree	av_block_2	5(3,8)	9(7,12)	18(15,21)	E
Cardiovascular	Bifascicular block	bifasc_block	0(NaN,NaN)	2(0,12)	3(0,10)	W
Cardiovascular	Bifascicular block	bifasc_block	0(NaN,NaN)	1(0,7)	2(0,8)	S
Cardiovascular	Bifascicular block	bifasc_block	1(0,2)	1(0,2)	3(2,5)	E
Cardiovascular	Coronary heart disease not otherwise specified	CHD_NOS	165(121,220)	487(427,553)	1136(1057,1220<br>)	W
Cardiovascular	Coronary heart disease not otherwise specified	CHD_NOS	215(174,261)	578(527,632)	1289(1220,1361<br>)	S

Cardiovascular	Coronary heart disease not otherwise specified	CHD_NOS	180(166,196)	537(517,558)	1239(1213,1265)	E
Cardiovascular	Dilated cardiomyopathy	dcm	6(1,22)	18(8,34)	28(17,44)	W
Cardiovascular	Dilated cardiomyopathy	dcm	4(0,15)	10(4,19)	28(18,41)	S
Cardiovascular	Dilated cardiomyopathy	dcm	12(8,16)	17(14,21)	26(22,30)	E
Cardiovascular	Heart failure	hf	62(37,98)	110(82,143)	355(311,403)	W
Cardiovascular	Heart failure	hf	39(23,61)	121(98,147)	292(259,327)	S
Cardiovascular	Heart failure	hf	40(33,48)	110(100,119)	301(288,314)	E
Cardiovascular	Hypertension	hypertension	1397(1265,1539)	2904(2759,3056)	4575(4417,4738)	W
Cardiovascular	Hypertension	hypertension	1147(1053,1246)	2348(2247,2452)	3977(3857,4099)	S
Cardiovascular	Hypertension	hypertension	1251(1213,1290)	2528(2484,2572)	4197(4150,4245)	E
Cardiovascular	Hypertrophic Cardiomyopathy	hocm	7(1,27)	11(4,25)	7(2,17)	W
Cardiovascular	Hypertrophic Cardiomyopathy	hocm	5(1,17)	11(5,21)	8(3,16)	S
Cardiovascular	Hypertrophic Cardiomyopathy	hocm	6(4,9)	9(6,12)	10(7,12)	E
Cardiovascular	Intracerebral haemorrhage	Intracereb_haem	13(4,34)	31(18,52)	48(33,68)	W
Cardiovascular	Intracerebral haemorrhage	Intracereb_haem	5(1,17)	35(24,50)	35(25,48)	S
Cardiovascular	Intracerebral haemorrhage	Intracereb_haem	9(6,13)	18(15,22)	34(30,39)	E
Cardiovascular	Ischaemic stroke	Isch_stroke	21(8,47)	77(55,106)	161(132,195)	W
Cardiovascular	Ischaemic stroke	Isch_stroke	32(18,52)	80(62,101)	188(162,216)	S
Cardiovascular	Ischaemic stroke	Isch_stroke	34(28,41)	71(64,79)	143(135,152)	E
Cardiovascular	Left bundle branch block	LBBB	23(9,48)	25(13,43)	68(50,90)	W
Cardiovascular	Left bundle branch block	LBBB	10(3,24)	24(15,37)	67(52,84)	S
Cardiovascular	Left bundle branch block	LBBB	12(8,16)	28(24,33)	72(66,79)	E
Cardiovascular	Multiple valve dz	mult_valve	7(1,24)	20(10,37)	50(34,70)	W

Cardiovascular	Multiple valve dz	mult_valve	11(4,26)	13(7,23)	51(38,66)	S
Cardiovascular	Multiple valve dz	mult_valve	9(6,13)	26(21,30)	61(55,67)	E
Cardiovascular	Myocardial infarction	myocardial_infarction	82(52,123)	283(238,335)	466(415,521)	W
Cardiovascular	Myocardial infarction	myocardial_infarction	148(115,188)	292(256,331)	611(563,661)	S
Cardiovascular	Myocardial infarction	myocardial_infarction	136(123,149)	330(314,347)	704(684,724)	E
Cardiovascular	Nonrheumatic aortic valve disorders	nonRh_aortic	37(19,67)	55(37,79)	161(132,194)	W
Cardiovascular	Nonrheumatic aortic valve disorders	nonRh_aortic	21(10,39)	53(39,71)	159(136,185)	S
Cardiovascular	Nonrheumatic aortic valve disorders	nonRh_aortic	23(18,28)	50(44,56)	136(128,145)	E
Cardiovascular	Nonrheumatic mitral valve disorders	nonRh_mitral	41(22,71)	72(50,99)	131(105,161)	W
Cardiovascular	Nonrheumatic mitral valve disorders	nonRh_mitral	28(15,47)	72(56,92)	163(139,189)	S
Cardiovascular	Nonrheumatic mitral valve disorders	nonRh_mitral	29(24,36)	55(48,61)	106(99,114)	E
Cardiovascular	Other Cardiomyopathy	cardiomyo_oth	20(7,44)	27(15,45)	52(36,72)	W
Cardiovascular	Other Cardiomyopathy	cardiomyo_oth	11(4,26)	22(13,35)	39(28,53)	S
Cardiovascular	Other Cardiomyopathy	cardiomyo_oth	16(12,21)	26(22,31)	44(39,49)	E
Cardiovascular	Pericardial effusion (noninflammatory)	pericardial_effusion	47(25,80)	24(12,42)	29(18,45)	W
Cardiovascular	Pericardial effusion (noninflammatory)	pericardial_effusion	11(4,27)	26(16,39)	46(34,61)	S
Cardiovascular	Pericardial effusion (noninflammatory)	pericardial_effusion	16(12,21)	25(21,30)	42(38,47)	E
Cardiovascular	Peripheral arterial disease	peripheral_arterial_disease	43(23,74)	117(89,151)	239(204,279)	W
Cardiovascular	Peripheral arterial disease	peripheral_arterial_disease	39(23,60)	85(66,107)	241(212,273)	S
Cardiovascular	Peripheral arterial disease	peripheral_arterial_disease	30(24,37)	75(68,84)	194(184,204)	E
Cardiovascular	Primary pulmonary hypertension	prim_pulm_htn	9(2,26)	5(1,16)	17(9,30)	W
Cardiovascular	Primary pulmonary hypertension	prim_pulm_htn	2(0,10)	11(5,20)	21(13,31)	S

Cardiovascular	Primary pulmonary hypertension	prim_pulm_htn	5(3,9)	6(4,9)	18(15,22)	E
Cardiovascular	Pulmonary embolism	PE	64(39,100)	98(73,130)	181(151,216)	W
Cardiovascular	Pulmonary embolism	PE	32(19,52)	92(73,115)	172(148,199)	S
Cardiovascular	Pulmonary embolism	PE	45(38,53)	85(77,93)	137(129,146)	E
Cardiovascular	Raynaud's syndrome	raynauds	122(87,167)	157(125,194)	146(119,177)	W
Cardiovascular	Raynaud's syndrome	raynauds	75(54,101)	80(63,101)	124(104,146)	S
Cardiovascular	Raynaud's syndrome	raynauds	148(135,161)	144(134,155)	159(150,169)	E
Cardiovascular	Rheumatic valve dz	Rh_valve	6(1,22)	15(6,29)	39(26,57)	W
Cardiovascular	Rheumatic valve dz	Rh_valve	3(0,12)	18(10,28)	37(26,50)	S
Cardiovascular	Rheumatic valve dz	Rh_valve	5(3,9)	13(10,16)	31(27,35)	E
Cardiovascular	Right bundle branch block	RBBB	30(14,57)	42(26,65)	74(55,98)	W
Cardiovascular	Right bundle branch block	RBBB	12(4,26)	21(13,34)	61(47,78)	S
Cardiovascular	Right bundle branch block	RBBB	28(22,34)	41(35,47)	87(80,94)	E
Cardiovascular	Secondary pulmonary hypertension	sec_pulm_htn	6(1,22)	7(2,18)	10(4,21)	W
Cardiovascular	Secondary pulmonary hypertension	sec_pulm_htn	0(NaN,NaN)	3(1,10)	9(4,17)	S
Cardiovascular	Secondary pulmonary hypertension	sec_pulm_htn	4(2,7)	5(3,7)	13(11,16)	E
Cardiovascular	Sick sinus syndrome	sick_sinus	4(0,21)	6(1,18)	31(19,47)	W
Cardiovascular	Sick sinus syndrome	sick_sinus	2(0,10)	6(2,14)	22(14,34)	S
Cardiovascular	Sick sinus syndrome	sick_sinus	4(2,6)	8(6,11)	18(15,22)	E
Cardiovascular	Stable angina	stable_angina	100(66,145)	391(337,450)	914(843,989)	W
Cardiovascular	Stable angina	stable_angina	128(98,165)	393(352,438)	976(917,1039)	S
Cardiovascular	Stable angina	stable_angina	129(117,142)	397(379,415)	933(911,956)	E
Cardiovascular	Stroke NOS	Stroke_NOS	54(31,89)	182(147,223)	335(293,381)	W
Cardiovascular	Stroke NOS	Stroke_NOS	102(75,135)	236(204,271)	490(448,534)	S
Cardiovascular	Stroke NOS	Stroke_NOS	61(53,70)	126(116,136)	283(270,295)	E

Cardiovascular	Subarachnoid haemorrhage	Subarach	10(2,29)	14(5,28)	57(40,77)	W
Cardiovascular	Subarachnoid haemorrhage	Subarach	22(11,40)	40(28,56)	33(23,45)	S
Cardiovascular	Subarachnoid haemorrhage	Subarach	22(17,28)	36(31,42)	42(38,47)	E
Cardiovascular	Subdural haematoma - nontraumatic	subdural_haem	4(0,21)	8(2,20)	16(8,29)	W
Cardiovascular	Subdural haematoma - nontraumatic	subdural_haem	11(4,26)	6(2,13)	10(5,19)	S
Cardiovascular	Subdural haematoma - nontraumatic	subdural_haem	3(1,5)	5(3,7)	11(9,13)	E
Cardiovascular	Supraventricular tachycardia	SVT	76(49,113)	72(51,99)	130(105,160)	W
Cardiovascular	Supraventricular tachycardia	SVT	55(37,80)	84(66,105)	122(102,145)	S
Cardiovascular	Supraventricular tachycardia	SVT	45(38,53)	72(65,80)	113(105,121)	E
Cardiovascular	Transient ischaemic attack	TIA	48(26,80)	152(120,190)	374(330,423)	W
Cardiovascular	Transient ischaemic attack	TIA	47(29,70)	119(97,144)	325(291,361)	S
Cardiovascular	Transient ischaemic attack	TIA	29(24,36)	86(78,95)	222(211,233)	E
Cardiovascular	Trifascicular block	trifasc_block	0(NaN,NaN)	4(1,16)	3(0,11)	W
Cardiovascular	Trifascicular block	trifasc_block	0(NaN,NaN)	0(NaN,NaN)	4(1,10)	S
Cardiovascular	Trifascicular block	trifasc_block	0(0,2)	0(0,1)	4(3,6)	E
Cardiovascular	Unstable Angina	unstable_angina	38(19,68)	111(84,144)	234(199,274)	W
Cardiovascular	Unstable Angina	unstable_angina	36(21,57)	106(85,131)	223(195,254)	S
Cardiovascular	Unstable Angina	unstable_angina	43(36,51)	120(111,130)	249(238,261)	E
Cardiovascular	Venous thromboembolic disease (Excl PE)	vte_ex_pe	120(84,165)	191(154,233)	278(240,321)	W
Cardiovascular	Venous thromboembolic disease (Excl PE)	vte_ex_pe	49(31,72)	110(89,134)	168(144,195)	S
Cardiovascular	Venous thromboembolic disease (Excl PE)	vte_ex_pe	97(86,108)	148(137,159)	239(228,250)	E
Cardiovascular	Ventricular tachycardia	VT	22(9,46)	17(7,33)	21(11,35)	W
Cardiovascular	Ventricular tachycardia	VT	7(1,20)	16(9,27)	31(21,44)	S
Cardiovascular	Ventricular tachycardia	VT	11(8,15)	17(14,21)	34(30,39)	E

Digestive	Abdominal Hernia	hernia_abdo	617(529,715)	822(743,906)	1192(1111,1279<br>)	W
Digestive	Abdominal Hernia	hernia_abdo	566(499,640)	791(731,855)	1135(1069,1203<br>)	S
Digestive	Abdominal Hernia	hernia_abdo	614(587,642)	815(790,841)	1191(1165,1217<br>)	E
Digestive	Alcoholic liver disease	liver_alc	14(4,36)	28(15,47)	33(21,50)	W
Digestive	Alcoholic liver disease	liver_alc	24(12,42)	40(27,56)	48(35,64)	S
Digestive	Alcoholic liver disease	liver_alc	21(17,27)	41(35,47)	35(30,39)	E
Digestive	Anal fissure	anal_fissure	392(324,470)	295(250,346)	267(230,309)	W
Digestive	Anal fissure	anal_fissure	207(169,250)	218(188,252)	179(154,207)	S
Digestive	Anal fissure	anal_fissure	346(326,366)	307(292,323)	265(254,278)	E
Digestive	Angiodysplasia of colon	angiodysplasia_colon	6(1,22)	8(2,21)	39(26,57)	W
Digestive	Angiodysplasia of colon	angiodysplasia_colon	2(0,10)	7(2,15)	18(11,28)	S
Digestive	Angiodysplasia of colon	angiodysplasia_colon	4(2,6)	8(6,11)	16(13,19)	E
Digestive	Anorectal fistula	anorectal_fistula	91(59,133)	94(69,125)	61(44,83)	W
Digestive	Anorectal fistula	anorectal_fistula	102(76,135)	124(101,150)	104(85,126)	S
Digestive	Anorectal fistula	anorectal_fistula	70(62,80)	75(68,83)	65(59,71)	E
Digestive	Anorectal prolapse	anorectal_prolapse	34(17,61)	44(28,65)	55(39,75)	W
Digestive	Anorectal prolapse	anorectal_prolapse	16(7,30)	36(25,51)	62(48,78)	S
Digestive	Anorectal prolapse	anorectal_prolapse	19(15,24)	37(32,42)	56(51,62)	E
Digestive	Appendicitis	appendicitis	289(231,358)	252(211,299)	243(208,283)	W
Digestive	Appendicitis	appendicitis	649(580,724)	849(789,912)	915(859,974)	S
Digestive	Appendicitis	appendicitis	347(327,367)	340(324,356)	332(319,346)	E
Digestive	Autoimmune liver disease	autoimm_liver	9(2,26)	16(7,31)	23(13,37)	W
Digestive	Autoimmune liver disease	autoimm_liver	15(6,30)	20(12,32)	14(8,22)	S
Digestive	Autoimmune liver disease	autoimm_liver	6(4,9)	12(10,16)	21(18,25)	E

Digestive	Barrett's oesophagus	barretts	29(12,57)	40(24,62)	114(90,143)	W
Digestive	Barrett's oesophagus	barretts	48(30,72)	81(63,103)	118(97,140)	S
Digestive	Barrett's oesophagus	barretts	36(30,43)	88(80,96)	145(136,154)	E
Digestive	Cholangitis	cholangitis	4(0,21)	19(9,34)	31(19,48)	W
Digestive	Cholangitis	cholangitis	6(1,19)	17(10,28)	23(15,34)	S
Digestive	Cholangitis	cholangitis	6(4,10)	16(13,20)	27(23,31)	E
Digestive	Cholecystitis	cholecystitis	194(149,249)	250(210,295)	327(286,372)	W
Digestive	Cholecystitis	cholecystitis	157(125,195)	187(160,217)	281(251,314)	S
Digestive	Cholecystitis	cholecystitis	162(149,176)	209(197,222)	297(285,310)	E
Digestive	Cholelithiasis	cholelithiasis	353(291,425)	541(481,606)	657(598,720)	W
Digestive	Cholelithiasis	cholelithiasis	392(341,450)	481(437,528)	716(667,768)	S
Digestive	Cholelithiasis	cholelithiasis	286(268,304)	375(359,392)	519(503,536)	E
Digestive	Coeliac disease	coeliac	52(29,84)	49(33,71)	87(66,111)	W
Digestive	Coeliac disease	coeliac	56(37,82)	80(63,100)	79(64,98)	S
Digestive	Coeliac disease	coeliac	63(55,72)	71(64,79)	71(65,77)	E
Digestive	Crohn's disease	crohns	53(30,88)	61(41,86)	59(42,80)	W
Digestive	Crohn's disease	crohns	73(51,101)	61(46,80)	59(45,76)	S
Digestive	Crohn's disease	crohns	45(38,52)	45(40,52)	49(44,55)	E
Digestive	Diaphragmatic hernia	hernia_diaphragm	304(245,373)	524(464,590)	906(837,980)	W
Digestive	Diaphragmatic hernia	hernia_diaphragm	307(260,361)	571(522,624)	879(824,936)	S
Digestive	Diaphragmatic hernia	hernia_diaphragm	331(312,351)	600(579,622)	942(920,964)	E
Digestive	Diverticular disease of intestine (acute and chronic)	diverticuli	172(128,227)	538(477,605)	1214(1133,1299)	W
Digestive	Diverticular disease of intestine (acute and chronic)	diverticuli	235(194,282)	680(627,737)	1317(1250,1388)	S
Digestive	Diverticular disease of intestine (acute and chronic)	diverticuli	198(183,213)	550(530,570)	1090(1066,1115)	E

Digestive	Fatty Liver	fatty_liver	24(10,49)	31(17,50)	39(26,57)	W
Digestive	Fatty Liver	fatty_liver	54(35,79)	57(42,76)	56(43,72)	S
Digestive	Fatty Liver	fatty_liver	97(87,108)	128(118,138)	121(113,129)	E
Digestive	Gastritis and duodenitis	gastritis_duodenitis	843(742,954)	1056(969,1147)	1260(1177,1346)	W
Digestive	Gastritis and duodenitis	gastritis_duodenitis	584(518,655)	819(760,881)	1130(1067,1196)	S
Digestive	Gastritis and duodenitis	gastritis_duodenitis	746(717,776)	1002(975,1030)	1338(1311,1365)	E
Digestive	Gastro-oesophageal reflux disease	GORD	1249(1125,1382)	1495(1392,1603)	1857(1757,1961)	W
Digestive	Gastro-oesophageal reflux disease	GORD	642(573,717)	918(855,983)	1133(1070,1198)	S
Digestive	Gastro-oesophageal reflux disease	GORD	517(493,542)	754(730,778)	988(965,1011)	E
Digestive	Hepatic failure	liver_fail	7(1,24)	10(3,23)	11(4,22)	W
Digestive	Hepatic failure	liver_fail	6(1,17)	13(6,23)	12(7,21)	S
Digestive	Hepatic failure	liver_fail	6(4,9)	12(9,15)	17(14,21)	E
Digestive	Inflammatory bowel disease (IBD)	IBD	143(102,194)	170(136,209)	180(150,215)	W
Digestive	Inflammatory bowel disease (IBD)	IBD	166(132,206)	163(138,193)	186(161,214)	S
Digestive	Inflammatory bowel disease (IBD)	IBD	129(117,142)	146(136,157)	164(155,173)	E
Digestive	Irritable bowel syndrome	IBS	907(803,1019)	952(872,1038)	841(775,912)	W
Digestive	Irritable bowel syndrome	IBS	700(631,776)	739(685,797)	673(625,723)	S
Digestive	Irritable bowel syndrome	IBS	259(243,277)	289(274,303)	279(268,292)	E
Digestive	Liver fibrosis, sclerosis and cirrhosis	cirrhosis	48(26,81)	58(39,84)	73(54,97)	W
Digestive	Liver fibrosis, sclerosis and cirrhosis	cirrhosis	37(22,59)	77(59,98)	89(71,109)	S
Digestive	Liver fibrosis, sclerosis and cirrhosis	cirrhosis	26(21,32)	46(40,53)	57(52,63)	E

Digestive	Oesophageal varices	varices	20(7,44)	21(10,38)	26(15,41)	W
Digestive	Oesophageal varices	varices	23(12,40)	23(14,35)	46(34,61)	S
Digestive	Oesophageal varices	varices	9(6,13)	19(15,23)	19(16,23)	E
Digestive	Oesophagitis and oesophageal ulcer	oesoph_ulc	690(599,791)	819(743,900)	1157(1078,1240<br>)	W
Digestive	Oesophagitis and oesophageal ulcer	oesoph_ulc	370(317,428)	595(544,649)	831(776,888)	S
Digestive	Oesophagitis and oesophageal ulcer	oesoph_ulc	443(420,466)	716(693,739)	975(952,998)	E
Digestive	Pancreatitis	pancreatitis	48(26,80)	80(58,109)	97(75,123)	W
Digestive	Pancreatitis	pancreatitis	45(28,68)	66(50,86)	102(84,123)	S
Digestive	Pancreatitis	pancreatitis	44(37,52)	61(54,68)	88(82,95)	E
Digestive	Peptic ulcer disease	ulcer_peptic	123(87,170)	227(188,273)	365(321,413)	W
Digestive	Peptic ulcer disease	ulcer_peptic	297(250,351)	420(377,466)	643(595,694)	S
Digestive	Peptic ulcer disease	ulcer_peptic	160(146,174)	288(273,303)	452(436,468)	E
Digestive	Peritonitis	peritonitis	53(30,88)	60(40,85)	79(59,103)	W
Digestive	Peritonitis	peritonitis	49(31,73)	64(48,83)	97(79,118)	S
Digestive	Peritonitis	peritonitis	46(39,54)	67(60,74)	81(75,88)	E
Digestive	Portal hypertension	portal_htn	14(4,36)	10(3,24)	18(9,31)	W
Digestive	Portal hypertension	portal_htn	5(1,17)	19(11,31)	22(14,33)	S
Digestive	Portal hypertension	portal_htn	5(3,8)	13(10,17)	15(12,18)	E
Digestive	Ulcerative colitis	ulc_colitis	107(73,152)	126(97,160)	133(107,163)	W
Digestive	Ulcerative colitis	ulc_colitis	115(87,149)	120(98,145)	148(126,173)	S
Digestive	Ulcerative colitis	ulc_colitis	95(85,106)	113(104,123)	130(122,138)	E
Digestive	Volvulus	volvulus	6(1,22)	18(8,33)	11(4,22)	W
Digestive	Volvulus	volvulus	9(3,22)	18(10,29)	27(18,39)	S
Digestive	Volvulus	volvulus	9(6,13)	14(11,18)	21(18,24)	E

Ear	Hearing loss	deaf	611(526,706)	935(853,1022)	1592(1499,1690)	W
Ear	Hearing loss	deaf	473(415,538)	699(645,757)	1056(995,1120)	S
Ear	Hearing loss	deaf	524(499,549)	807(782,832)	1361(1334,1388)	E
Ear	Meniere disease	meniere	62(38,97)	121(94,154)	193(161,228)	W
Ear	Meniere disease	meniere	42(26,64)	40(28,55)	83(66,102)	S
Ear	Meniere disease	meniere	23(19,29)	50(44,56)	72(66,78)	E
Ear	Tinnitus	tinnitus	488(411,575)	632(565,704)	704(642,770)	W
Ear	Tinnitus	tinnitus	215(176,260)	268(235,304)	303(271,338)	S
Ear	Tinnitus	tinnitus	294(276,313)	484(465,504)	564(546,581)	E
Endocrine	Cystic Fibrosis	CF	0(NaN,NaN)	2(0,9)	6(2,14)	W
Endocrine	Cystic Fibrosis	CF	7(2,18)	15(8,25)	6(2,12)	S
Endocrine	Cystic Fibrosis	CF	5(3,7)	4(3,7)	3(2,4)	E
Endocrine	Diabetes NOS	diabetes_nos	28(12,54)	81(58,111)	118(93,146)	W
Endocrine	Diabetes NOS	diabetes_nos	23(11,41)	66(50,86)	93(75,114)	S
Endocrine	Diabetes NOS	diabetes_nos	43(36,51)	79(71,87)	125(117,134)	E
Endocrine	Diabetes Type I	diabetes_t1	52(30,85)	85(61,115)	94(72,120)	W
Endocrine	Diabetes Type I	diabetes_t1	55(36,81)	81(63,102)	92(75,113)	S
Endocrine	Diabetes Type I	diabetes_t1	62(54,71)	71(64,79)	99(92,106)	E
Endocrine	Diabetes Type II	diabetes_t2	370(304,447)	784(708,865)	1166(1086,1250)	W
Endocrine	Diabetes Type II	diabetes_t2	261(217,312)	603(552,657)	905(848,965)	S
Endocrine	Diabetes Type II	diabetes_t2	241(224,258)	436(418,455)	737(717,757)	E
Endocrine	Hyperparathyroidism	PTH	16(5,37)	61(42,86)	77(57,100)	W
Endocrine	Hyperparathyroidism	PTH	11(4,24)	17(10,28)	41(30,54)	S
Endocrine	Hyperparathyroidism	PTH	14(11,19)	25(21,30)	47(43,53)	E

Endocrine	Hyperthyroidism	hyperthyroid	117(82,162)	186(152,225)	196(165,232)	W
Endocrine	Hyperthyroidism	hyperthyroid	105(79,136)	187(161,217)	208(182,236)	S
Endocrine	Hyperthyroidism	hyperthyroid	112(101,124)	144(134,154)	164(155,174)	E
Endocrine	Hypo or hyperthyroidism	thyroid	600(518,691)	848(774,928)	1023(950,1100)	W
Endocrine	Hypo or hyperthyroidism	thyroid	505(447,569)	722(669,778)	932(877,990)	S
Endocrine	Hypo or hyperthyroidism	thyroid	442(420,464)	662(641,684)	851(830,872)	E
Endocrine	Hypothyroidism	hypothyroid	503(428,587)	735(667,809)	879(812,951)	W
Endocrine	Hypothyroidism	hypothyroid	434(380,493)	602(554,653)	822(771,876)	S
Endocrine	Hypothyroidism	hypothyroid	344(325,364)	546(526,565)	720(701,739)	E
Endocrine	Obesity	obesity	994(885,1113)	1304(1209,1405)	1306(1222,1394)	W
Endocrine	Obesity	obesity	1112(1022,1209)	1444(1365,1526)	1634(1558,1713)	S
Endocrine	Obesity	obesity	791(761,822)	960(933,987)	1012(989,1035)	E
Endocrine	Polycystic ovarian syndrome	PCOS	63(39,97)	5(1,15)	0(NaN,NaN)	W
Endocrine	Polycystic ovarian syndrome	PCOS	67(48,92)	22(14,33)	9(5,16)	S
Endocrine	Polycystic ovarian syndrome	PCOS	69(61,78)	21(18,25)	5(3,6)	E
Endocrine	Syndrome of inappropriate secretion of antidiuretic hormone	SIADH	0(NaN,NaN)	0(NaN,NaN)	7(2,17)	W
Endocrine	Syndrome of inappropriate secretion of antidiuretic hormone	SIADH	2(0,13)	2(0,7)	4(1,10)	S
Endocrine	Syndrome of inappropriate secretion of antidiuretic hormone	SIADH	0(0,2)	2(1,4)	6(4,8)	E
Endocrine	Thyroiditis unspecified	thyroid_nos	270(216,334)	340(294,391)	430(384,481)	W
Endocrine	Thyroiditis unspecified	thyroid_nos	179(145,218)	293(259,329)	375(340,412)	S
Endocrine	Thyroiditis unspecified	thyroid_nos	91(81,101)	135(126,145)	182(172,191)	E
Eye	Anterior and Intermediate Uveitis	ant_uveitis	95(63,137)	147(116,184)	119(94,148)	W

Eye	Anterior and Intermediate Uveitis	ant_uveitis	74(52,102)	105(85,128)	103(85,125)	S
Eye	Anterior and Intermediate Uveitis	ant_uveitis	56(48,65)	75(68,83)	82(75,88)	E
Eye	Cataract	cataract	94(62,136)	317(271,369)	945(874,1020)	W
Eye	Cataract	cataract	125(95,160)	305(270,344)	907(851,966)	S
Eye	Cataract	cataract	114(103,126)	335(319,351)	1031(1008,1054)	E
Eye	Diabetic ophthalmic complications	diab_eye	113(78,159)	268(225,318)	422(375,474)	W
Eye	Diabetic ophthalmic complications	diab_eye	135(103,172)	282(247,320)	411(372,452)	S
Eye	Diabetic ophthalmic complications	diab_eye	168(154,182)	279(264,294)	415(400,430)	E
Eye	Glaucoma	glaucoma	77(48,117)	182(147,222)	373(329,421)	W
Eye	Glaucoma	glaucoma	43(26,65)	123(101,148)	295(263,330)	S
Eye	Glaucoma	glaucoma	58(50,67)	158(147,169)	354(340,368)	E
Eye	Macular degeneration	macula_degen	10(2,31)	70(49,96)	175(145,209)	W
Eye	Macular degeneration	macula_degen	38(22,60)	57(42,75)	207(180,236)	S
Eye	Macular degeneration	macula_degen	18(14,23)	36(31,42)	140(132,149)	E
Eye	Posterior Uveitis	post_uveitis	10(2,31)	16(7,30)	14(7,27)	W
Eye	Posterior Uveitis	post_uveitis	12(4,25)	15(8,25)	13(7,21)	S
Eye	Posterior Uveitis	post_uveitis	7(5,11)	9(6,12)	10(8,13)	E
Eye	Ptosis of eyelid	ptosis	16(5,39)	38(23,58)	57(41,78)	W
Eye	Ptosis of eyelid	ptosis	21(10,37)	24(15,36)	56(43,71)	S
Eye	Ptosis of eyelid	ptosis	30(25,37)	50(44,57)	77(71,83)	E
Eye	Retinal detachments and breaks	retinal_detach	61(37,96)	87(64,117)	143(116,175)	W
Eye	Retinal detachments and breaks	retinal_detach	39(23,60)	85(67,106)	122(102,146)	S
Eye	Retinal detachments and breaks	retinal_detach	44(37,52)	90(82,99)	150(142,160)	E

Eye	Retinal vascular occlusions	retinal_vasc_occl	10(2,29)	29(16,49)	81(61,106)	W
Eye	Retinal vascular occlusions	retinal_vasc_occl	10(3,24)	28(18,42)	80(64,100)	S
Eye	Retinal vascular occlusions	retinal_vasc_occl	13(9,18)	30(25,35)	73(67,80)	E
Eye	Scleritis and episcleritis	scleritis	76(48,114)	88(65,117)	82(62,106)	W
Eye	Scleritis and episcleritis	scleritis	29(16,48)	34(23,49)	48(36,62)	S
Eye	Scleritis and episcleritis	scleritis	14(10,18)	23(19,28)	25(21,28)	E
Eye	Visual impairment and blindness	blind	44(23,75)	66(46,92)	80(60,104)	W
Eye	Visual impairment and blindness	blind	37(22,59)	56(41,74)	62(48,80)	S
Eye	Visual impairment and blindness	blind	37(31,44)	55(48,61)	78(71,84)	E
Genitourinary	Acute Kidney Injury	AKI	21(8,45)	64(44,91)	154(126,187)	W
Genitourinary	Acute Kidney Injury	AKI	28(15,47)	71(54,92)	150(127,175)	S
Genitourinary	Acute Kidney Injury	AKI	35(28,42)	73(65,81)	158(149,167)	E
Genitourinary	Chronic Kidney Disease	CKD	161(119,214)	402(349,462)	949(877,1024)	W
Genitourinary	Chronic Kidney Disease	CKD	90(66,120)	189(161,220)	646(599,696)	S
Genitourinary	Chronic Kidney Disease	CKD	57(49,65)	122(112,132)	308(295,321)	E
Genitourinary	Dysmenorrhoea	dysmenorrhoea	502(428,584)	255(216,299)	75(56,98)	W
Genitourinary	Dysmenorrhoea	dysmenorrhoea	195(161,235)	179(153,207)	96(79,115)	S
Genitourinary	Dysmenorrhoea	dysmenorrhoea	349(330,368)	229(217,241)	88(82,95)	E
Genitourinary	End stage renal disease	ESRD	16(5,39)	21(10,37)	61(44,83)	W
Genitourinary	End stage renal disease	ESRD	26(14,44)	29(19,43)	36(26,49)	S
Genitourinary	End stage renal disease	ESRD	17(12,22)	28(23,33)	36(32,41)	E
Genitourinary	Endometrial hyperplasia and hypertrophy	endometrial_hyper	103(71,144)	82(60,108)	53(38,73)	W
Genitourinary	Endometrial hyperplasia and hypertrophy	endometrial_hyper	21(11,36)	26(17,38)	32(23,44)	S
Genitourinary	Endometrial hyperplasia and hypertrophy	endometrial_hyper	59(51,67)	72(65,79)	58(53,64)	E

Genitourinary	Endometriosis	endometriosis	257(205,318)	198(164,237)	76(58,99)	W
Genitourinary	Endometriosis	endometriosis	216(180,258)	197(170,226)	93(76,111)	S
Genitourinary	Endometriosis	endometriosis	290(273,308)	199(188,211)	109(101,116)	E
Genitourinary	Erectile dysfunction	ED	514(432,607)	957(870,1051)	1341(1254,1433<br>)	W
Genitourinary	Erectile dysfunction	ED	235(191,285)	401(357,447)	646(596,699)	S
Genitourinary	Erectile dysfunction	ED	409(387,433)	760(735,786)	1126(1101,1151<br>)	E
Genitourinary	Female genital prolapse	female_genital_prolapse	284(229,348)	457(404,514)	728(667,792)	W
Genitourinary	Female genital prolapse	female_genital_prolapse	152(122,188)	274(243,308)	562(521,606)	S
Genitourinary	Female genital prolapse	female_genital_prolapse	214(200,230)	376(361,392)	633(616,651)	E
Genitourinary	Female infertility	female_infertility	245(194,304)	82(60,108)	20(11,34)	W
Genitourinary	Female infertility	female_infertility	282(240,329)	116(96,139)	67(53,83)	S
Genitourinary	Female infertility	female_infertility	201(187,216)	108(100,117)	61(55,66)	E
Genitourinary	Glomerulonephritis	GN	34(16,62)	51(33,75)	69(50,92)	W
Genitourinary	Glomerulonephritis	GN	81(57,110)	93(74,115)	127(107,151)	S
Genitourinary	Glomerulonephritis	GN	34(28,41)	48(43,55)	75(69,82)	E
Genitourinary	Hydrocoele (incl infected)	hydrocele	41(20,73)	58(38,85)	104(81,132)	W
Genitourinary	Hydrocoele (incl infected)	hydrocele	76(52,107)	70(53,92)	99(80,121)	S
Genitourinary	Hydrocoele (incl infected)	hydrocele	72(63,83)	74(66,82)	117(109,125)	E
Genitourinary	Hyperplasia of prostate	BPH	115(78,163)	417(360,480)	1025(949,1106)	W
Genitourinary	Hyperplasia of prostate	BPH	71(48,101)	315(277,357)	862(804,922)	S
Genitourinary	Hyperplasia of prostate	BPH	77(67,87)	305(290,322)	833(811,855)	E
Genitourinary	Male infertility	male_infertility	52(28,87)	15(6,31)	6(2,16)	W
Genitourinary	Male infertility	male_infertility	60(39,88)	35(23,50)	26(17,39)	S
Genitourinary	Male infertility	male_infertility	54(46,62)	38(33,44)	19(16,23)	E

Genitourinary	Menorrhagia and polymenorrhoea	menorrhagia	1411(1286,1545)	1152(1067,1241)	472(423,524)	W
Genitourinary	Menorrhagia and polymenorrhoea	menorrhagia	624(562,692)	680(630,733)	448(411,488)	S
Genitourinary	Menorrhagia and polymenorrhoea	menorrhagia	1243(1207,1280)	1003(978,1029)	498(482,514)	E
Genitourinary	Neuromuscular dysfunction of bladder	neuro_bladder	80(52,119)	124(97,158)	127(102,157)	W
Genitourinary	Neuromuscular dysfunction of bladder	neuro_bladder	106(80,138)	175(149,205)	237(209,268)	S
Genitourinary	Neuromuscular dysfunction of bladder	neuro_bladder	103(92,114)	150(140,161)	221(210,232)	E
Genitourinary	Non-acute cystitis	chr_cystitis	10(2,29)	25(13,42)	33(21,49)	W
Genitourinary	Non-acute cystitis	chr_cystitis	12(5,25)	21(12,33)	30(21,42)	S
Genitourinary	Non-acute cystitis	chr_cystitis	17(13,21)	25(21,29)	41(37,46)	E
Genitourinary	Obstructive and reflux uropathy	obstr_reflux	36(18,64)	78(56,106)	105(82,132)	W
Genitourinary	Obstructive and reflux uropathy	obstr_reflux	47(29,70)	80(63,101)	94(77,115)	S
Genitourinary	Obstructive and reflux uropathy	obstr_reflux	61(53,70)	68(61,76)	101(94,109)	E
Genitourinary	Postcoital and contact bleeding	PCB	251(200,311)	175(143,212)	97(76,122)	W
Genitourinary	Postcoital and contact bleeding	PCB	57(39,80)	54(40,70)	30(21,41)	S
Genitourinary	Postcoital and contact bleeding	PCB	218(203,234)	164(154,175)	77(71,83)	E
Genitourinary	Postmenopausal bleeding	PMB	151(112,199)	598(538,664)	809(746,877)	W
Genitourinary	Postmenopausal bleeding	PMB	66(47,90)	254(224,287)	450(413,489)	S
Genitourinary	Postmenopausal bleeding	PMB	92(82,102)	443(426,460)	593(576,610)	E
Genitourinary	Tubulo-interstitial nephritis	TIN	25(11,49)	14(5,28)	35(23,52)	W
Genitourinary	Tubulo-interstitial nephritis	TIN	23(12,39)	25(16,37)	20(12,30)	S
Genitourinary	Tubulo-interstitial nephritis	TIN	24(19,30)	25(21,30)	21(18,25)	E
Genitourinary	Undescended testicle	undescended_testis	11(2,32)	2(0,12)	6(2,16)	W

Genitourinary	Undescended testicle	undescended_testis	21(9,39)	13(6,24)	20(12,31)	S
Genitourinary	Undescended testicle	undescended_testis	24(19,30)	17(13,21)	12(9,14)	E
Genitourinary	Urinary Incontinence	urine_incont	399(333,474)	570(510,635)	729(668,794)	W
Genitourinary	Urinary Incontinence	urine_incont	292(249,340)	377(340,418)	497(457,539)	S
Genitourinary	Urinary Incontinence	urine_incont	360(341,380)	475(457,493)	582(565,600)	E
Genitourinary	Urolithiasis	urolithiasis	213(163,273)	265(222,315)	322(280,367)	W
Genitourinary	Urolithiasis	urolithiasis	228(187,275)	343(305,385)	404(366,445)	S
Genitourinary	Urolithiasis	urolithiasis	209(193,225)	299(284,315)	359(345,373)	E
Haematological or immunological	Agranulocytosis	agranulocytosis	96(65,137)	134(105,168)	169(140,203)	W
Haematological or immunological	Agranulocytosis	agranulocytosis	97(72,128)	98(79,120)	143(121,166)	S
Haematological or immunological	Agranulocytosis	agranulocytosis	81(71,91)	124(115,134)	148(139,157)	E
Haematological or immunological	Aplastic anaemias	aplastic	3(0,17)	11(4,25)	15(7,27)	W
Haematological or immunological	Aplastic anaemias	aplastic	6(1,19)	11(5,21)	13(7,22)	S
Haematological or immunological	Aplastic anaemias	aplastic	8(5,11)	14(11,18)	19(16,22)	E
Haematological or immunological	Folate deficiency anaemia	folatedef	31(14,58)	18(9,33)	19(10,33)	W

Haematological or immunological	Folate deficiency anaemia	folatedef	21(11,37)	29(19,43)	40(29,54)	S
Haematological or immunological	Folate deficiency anaemia	folatedef	30(24,36)	25(21,30)	36(32,41)	E
Haematological or immunological	Hyposplenism	hyposplenism	10(2,31)	23(12,39)	24(14,39)	W
Haematological or immunological	Hyposplenism	hyposplenism	10(3,24)	25(15,38)	36(25,50)	S
Haematological or immunological	Hyposplenism	hyposplenism	19(15,25)	21(17,26)	23(19,27)	E
Haematological or immunological	Immunodeficiencies	immunodef	3(0,17)	4(0,14)	4(1,12)	W
Haematological or immunological	Immunodeficiencies	immunodef	5(1,15)	1(0,6)	14(7,23)	S
Haematological or immunological	Immunodeficiencies	immunodef	7(4,10)	6(4,8)	7(5,9)	E
Haematological or immunological	Iron deficiency anaemia	IDA	517(442,602)	457(403,517)	470(420,523)	W
Haematological or immunological	Iron deficiency anaemia	IDA	373(323,428)	454(412,499)	482(442,525)	S
Haematological or immunological	Iron deficiency anaemia	IDA	296(278,314)	330(315,346)	359(346,373)	E

Haematological or immunological	Other anaemias	oth_anaemia	741(650,841)	731(661,805)	816(751,887)	W
Haematological or immunological	Other anaemias	oth_anaemia	444(389,503)	523(477,571)	601(556,649)	S
Haematological or immunological	Other anaemias	oth_anaemia	458(436,480)	520(501,539)	622(604,640)	E
Haematological or immunological	Other haemolytic anaemias	oth_haem_anaemia	10(2,31)	4(0,14)	13(6,25)	W
Haematological or immunological	Other haemolytic anaemias	oth_haem_anaemia	8(2,21)	10(5,20)	7(3,14)	S
Haematological or immunological	Other haemolytic anaemias	oth_haem_anaemia	11(7,15)	12(9,15)	14(11,17)	E
Haematological or immunological	Primary or Idiopathic Thrombocytopaenia	pri_thrombocytopaenia	18(7,39)	22(11,39)	54(38,74)	W
Haematological or immunological	Primary or Idiopathic Thrombocytopaenia	pri_thrombocytopaenia	25(13,44)	40(28,55)	52(39,68)	S
Haematological or immunological	Primary or Idiopathic Thrombocytopaenia	pri_thrombocytopaenia	27(22,33)	39(34,45)	48(43,54)	E
Haematological or immunological	Sarcoidosis	sarcoid	36(17,66)	38(23,59)	39(25,56)	W
Haematological or immunological	Sarcoidosis	sarcoid	37(22,58)	36(25,51)	78(62,97)	S

Haematological or immunological	Sarcoidosis	sarcoid	31(25,38)	34(29,39)	37(33,41)	E
Haematological or immunological	Secondary or other Thrombocytopaenia	sec_oth_thrombocytopaenia	40(21,68)	29(16,48)	72(53,95)	W
Haematological or immunological	Secondary or other Thrombocytopaenia	sec_oth_thrombocytopaenia	37(22,58)	53(39,71)	65(51,83)	S
Haematological or immunological	Secondary or other Thrombocytopaenia	sec_oth_thrombocytopaenia	34(28,41)	51(45,57)	70(64,77)	E
Haematological or immunological	Secondary polycythaemia	2ry_polycythaemia	20(7,44)	25(13,44)	22(12,36)	W
Haematological or immunological	Secondary polycythaemia	2ry_polycythaemia	2(0,13)	7(2,15)	10(5,19)	S
Haematological or immunological	Secondary polycythaemia	2ry_polycythaemia	6(4,10)	8(6,11)	11(9,14)	E
Haematological or immunological	Sickle-cell anaemia	sickle_cell	0(NaN,NaN)	2(0,9)	3(0,10)	W
Haematological or immunological	Sickle-cell anaemia	sickle_cell	2(0,13)	0(NaN,NaN)	0(NaN,NaN)	S
Haematological or immunological	Sickle-cell anaemia	sickle_cell	4(2,7)	3(2,5)	1(0,2)	E
Haematological or immunological	Sickle-cell trait	sickle_trait	13(3,33)	3(0,12)	1(0,8)	W

Haematological or immunological	Sickle-cell trait	sickle_trait	4(0,15)	2(0,7)	0(NaN,NaN)	S
Haematological or immunological	Sickle-cell trait	sickle_trait	13(10,18)	10(8,13)	5(4,7)	E
Haematological or immunological	Splenomegaly	splenomegaly	4(0,21)	16(7,31)	24(13,38)	W
Haematological or immunological	Splenomegaly	splenomegaly	5(1,17)	18(10,30)	15(8,25)	S
Haematological or immunological	Splenomegaly	splenomegaly	8(6,12)	15(12,19)	15(13,19)	E
Haematological or immunological	Thalassaemia	thala	6(1,22)	16(7,30)	9(3,19)	W
Haematological or immunological	Thalassaemia	thala	8(2,21)	6(2,14)	1(0,5)	S
Haematological or immunological	Thalassaemia	thala	7(4,10)	6(4,8)	4(2,5)	E
Haematological or immunological	Thalassaemia trait	thal_trait	19(7,41)	13(5,27)	6(2,15)	W
Haematological or immunological	Thalassaemia trait	thal_trait	10(3,23)	7(3,16)	5(1,11)	S
Haematological or immunological	Thalassaemia trait	thal_trait	17(13,22)	13(10,17)	9(7,12)	E

Haematological or immunological	Thrombophilia	thrombophilia	38(19,66)	32(19,51)	39(26,57)	W
Haematological or immunological	Thrombophilia	thrombophilia	25(13,43)	29(19,42)	18(11,28)	S
Haematological or immunological	Thrombophilia	thrombophilia	41(34,48)	36(31,41)	33(29,37)	E
Haematological or immunological	Vitamin B12 deficiency anaemia	b12_def	130(92,177)	163(131,202)	197(165,233)	W
Haematological or immunological	Vitamin B12 deficiency anaemia	b12_def	100(74,131)	127(105,152)	178(154,206)	S
Haematological or immunological	Vitamin B12 deficiency anaemia	b12_def	103(93,114)	124(115,134)	164(155,174)	E
Infections	Bacterial Diseases (excl TB)	bacterial	788(692,894)	932(851,1018)	1159(1080,1242)	W
Infections	Bacterial Diseases (excl TB)	bacterial	743(670,823)	867(806,931)	1045(984,1108)	S
Infections	Bacterial Diseases (excl TB)	bacterial	811(780,841)	897(872,924)	1105(1081,1129)	E
Infections	Chronic viral hepatitis	chr_hep	30(14,57)	19(9,34)	15(7,27)	W
Infections	Chronic viral hepatitis	chr_hep	44(27,68)	43(30,59)	21(13,31)	S
Infections	Chronic viral hepatitis	chr_hep	43(36,50)	42(36,48)	26(22,30)	E
Infections	Ear and Upper Respiratory Tract Infections	ear_urti	196(149,254)	142(111,179)	141(115,172)	W
Infections	Ear and Upper Respiratory Tract Infections	ear_urti	146(115,183)	132(109,159)	114(95,137)	S
Infections	Ear and Upper Respiratory Tract Infections	ear_urti	159(145,173)	139(129,149)	151(142,160)	E
Infections	Encephalitis	enceph	4(0,21)	2(0,12)	6(2,15)	W

Infections	Encephalitis	enceph	0(NaN,NaN)	4(1,10)	4(1,10)	S
Infections	Encephalitis	enceph	1(0,2)	3(2,5)	3(2,4)	E
Infections	Eye infections	eye	16(5,39)	7(2,18)	21(12,35)	W
Infections	Eye infections	eye	9(2,22)	14(7,24)	15(9,24)	S
Infections	Eye infections	eye	9(6,13)	15(11,18)	17(14,20)	E
Infections	Female pelvic inflammatory disease	PID	193(149,247)	158(128,194)	102(80,128)	W
Infections	Female pelvic inflammatory disease	PID	99(75,128)	50(37,65)	25(17,35)	S
Infections	Female pelvic inflammatory disease	PID	172(159,186)	111(102,120)	66(60,71)	E
Infections	HIV	hiv	16(5,39)	2(0,12)	2(0,9)	W
Infections	HIV	hiv	9(2,22)	11(5,21)	3(1,9)	S
Infections	HIV	hiv	24(19,30)	13(10,16)	3(2,5)	E
Infections	Infection of anal and rectal regions	anorectal	37(19,67)	46(29,69)	24(14,39)	W
Infections	Infection of anal and rectal regions	anorectal	80(57,110)	67(51,87)	38(27,52)	S
Infections	Infection of anal and rectal regions	anorectal	50(43,58)	45(40,52)	32(28,36)	E
Infections	Infection of bones and joints	bone	19(7,42)	26(14,45)	44(30,63)	W
Infections	Infection of bones and joints	bone	6(1,17)	26(16,39)	31(21,44)	S
Infections	Infection of bones and joints	bone	21(16,26)	28(23,33)	31(27,35)	E
Infections	Infection of liver	liver	17(6,40)	18(8,35)	13(6,26)	W
Infections	Infection of liver	liver	17(8,33)	25(15,38)	15(9,24)	S
Infections	Infection of liver	liver	18(14,23)	24(20,28)	17(14,20)	E
Infections	Infection of male genital system	male_GU	22(8,48)	17(7,34)	49(34,69)	W
Infections	Infection of male genital system	male_GU	44(26,68)	40(27,56)	43(31,58)	S
Infections	Infection of male genital system	male_GU	32(26,39)	30(25,35)	38(34,43)	E

Infections	Infection of other or unspecified genitourinary system	oth_gu	21(9,44)	29(17,47)	18(9,30)	W
Infections	Infection of other or unspecified genitourinary system	oth_gu	31(18,49)	12(6,21)	12(6,20)	S
Infections	Infection of other or unspecified genitourinary system	oth_gu	32(26,38)	19(16,23)	14(11,17)	E
Infections	Infection of skin and subcutaneous tissues	skin	225(174,286)	268(225,317)	336(293,382)	W
Infections	Infection of skin and subcutaneous tissues	skin	257(214,306)	270(236,307)	289(257,323)	S
Infections	Infection of skin and subcutaneous tissues	skin	234(218,251)	290(275,305)	329(316,343)	E
Infections	Infections of Other or unspecified organs	oth_organs	323(263,394)	430(375,490)	551(497,610)	W
Infections	Infections of Other or unspecified organs	oth_organs	229(188,275)	322(285,361)	454(414,496)	S
Infections	Infections of Other or unspecified organs	oth_organs	316(297,336)	383(366,400)	514(498,531)	E
Infections	Infections of the digestive system	digestive	131(93,179)	211(174,255)	264(227,306)	W
Infections	Infections of the digestive system	digestive	86(62,116)	134(111,161)	164(141,190)	S
Infections	Infections of the digestive system	digestive	194(179,209)	250(236,264)	314(302,327)	E
Infections	Infections of the Heart	heart	7(1,24)	4(0,14)	7(2,17)	W
Infections	Infections of the Heart	heart	2(0,10)	1(0,6)	9(4,17)	S
Infections	Infections of the Heart	heart	3(1,6)	6(4,8)	9(7,12)	E
Infections	Lower Respiratory Tract Infections	lrti	236(184,298)	351(302,407)	566(511,625)	W
Infections	Lower Respiratory Tract Infections	lrti	213(174,258)	330(293,371)	549(505,595)	S
Infections	Lower Respiratory Tract Infections	lrti	234(217,251)	305(290,321)	539(523,557)	E
Infections	Meningitis	meningitis	6(1,22)	8(2,20)	1(0,8)	W
Infections	Meningitis	meningitis	2(0,10)	8(3,16)	5(2,12)	S

Infections	Meningitis	meningitis	15(11,19)	6(4,8)	7(5,9)	E
Infections	Mycoses	mycoses	44(24,73)	66(46,92)	66(48,88)	W
Infections	Mycoses	mycoses	25(13,44)	31(20,45)	50(37,65)	S
Infections	Mycoses	mycoses	46(39,53)	51(45,58)	88(81,95)	E
Infections	Other nervous system infections	oth_nerv_sys	7(1,24)	10(3,23)	19(10,32)	W
Infections	Other nervous system infections	oth_nerv_sys	3(0,12)	21(12,33)	21(13,32)	S
Infections	Other nervous system infections	oth_nerv_sys	9(6,13)	13(10,17)	16(13,19)	E
Infections	Other or unspecified infectious organisms	oth_organisms	536(457,626)	656(588,730)	970(898,1047)	W
Infections	Other or unspecified infectious organisms	oth_organisms	451(394,514)	618(567,672)	861(806,919)	S
Infections	Other or unspecified infectious organisms	oth_organisms	533(509,559)	651(629,673)	948(926,971)	E
Infections	Parasitic infections	parasitic	3(0,17)	10(3,23)	10(4,21)	W
Infections	Parasitic infections	parasitic	7(1,20)	9(4,19)	10(5,18)	S
Infections	Parasitic infections	parasitic	11(8,16)	10(8,13)	11(8,13)	E
Infections	Rheumatic fever	rh_fever	16(5,37)	15(6,29)	62(45,83)	W
Infections	Rheumatic fever	rh_fever	8(2,21)	41(29,57)	76(60,94)	S
Infections	Rheumatic fever	rh_fever	12(9,16)	30(26,36)	59(54,65)	E
Infections	Septicaemia	sepsis	57(33,92)	54(35,78)	134(108,164)	W
Infections	Septicaemia	sepsis	52(34,76)	61(46,79)	130(110,154)	S
Infections	Septicaemia	sepsis	46(39,54)	81(74,89)	130(122,139)	E
Infections	Tuberculosis	TB	22(9,46)	10(3,24)	32(20,48)	W
Infections	Tuberculosis	TB	55(36,80)	69(53,89)	189(164,217)	S
Infections	Tuberculosis	TB	46(39,54)	60(54,67)	97(90,104)	E
Infections	Urinary Tract Infections	uti	169(127,221)	333(286,386)	473(424,527)	W
Infections	Urinary Tract Infections	uti	221(182,265)	341(303,381)	504(462,548)	S

Infections	Urinary Tract Infections	uti	196(181,211)	272(258,286)	421(407,437)	E
Infections	Viral diseases (excl chronic hepatitis/HIV)	viral	150(110,201)	142(112,179)	163(134,196)	W
Infections	Viral diseases (excl chronic hepatitis/HIV)	viral	137(107,174)	140(116,167)	148(126,172)	S
Infections	Viral diseases (excl chronic hepatitis/HIV)	viral	145(133,159)	161(150,172)	151(143,161)	E
Musculoskeletal	Ankylosing spondylitis	ank_spond	28(12,56)	39(23,61)	42(28,60)	W
Musculoskeletal	Ankylosing spondylitis	ank_spond	20(10,37)	31(20,45)	33(22,46)	S
Musculoskeletal	Ankylosing spondylitis	ank_spond	27(22,34)	35(30,41)	39(34,44)	E
Musculoskeletal	Carpal tunnel syndrome	carpal_tunnel	406(339,483)	604(541,673)	647(589,709)	W
Musculoskeletal	Carpal tunnel syndrome	carpal_tunnel	215(177,260)	335(299,375)	398(362,437)	S
Musculoskeletal	Carpal tunnel syndrome	carpal_tunnel	417(396,440)	588(567,608)	656(638,675)	E
Musculoskeletal	Collapsed vertebra	collapsed_vert	6(1,22)	12(4,26)	37(24,54)	W
Musculoskeletal	Collapsed vertebra	collapsed_vert	12(4,26)	17(9,28)	26(17,38)	S
Musculoskeletal	Collapsed vertebra	collapsed_vert	7(4,10)	14(11,18)	36(32,41)	E
Musculoskeletal	Enteropathic arthropathy	entero_arthro	7(1,24)	2(0,9)	0(NaN,NaN)	W
Musculoskeletal	Enteropathic arthropathy	entero_arthro	0(NaN,NaN)	0(NaN,NaN)	2(0,6)	S
Musculoskeletal	Enteropathic arthropathy	entero_arthro	2(1,4)	1(0,2)	2(1,3)	E

Musculoskeletal	Enthesopathies & synovial disorders	enthesopathy	2819(2632,3016)	3564(3405,3730)	3889(3743,4039)	W
Musculoskeletal	Enthesopathies & synovial disorders	enthesopathy	1156(1063,1255)	1521(1441,1605)	1786(1707,1868)	S
Musculoskeletal	Enthesopathies & synovial disorders	enthesopathy	2211(2161,2262)	2792(2747,2838)	2994(2955,3034)	E
Musculoskeletal	Fibromatoses	fibromatosis	53(29,87)	216(177,261)	444(395,497)	W
Musculoskeletal	Fibromatoses	fibromatosis	64(43,91)	112(90,137)	272(240,306)	S
Musculoskeletal	Fibromatoses	fibromatosis	58(50,67)	169(158,181)	324(311,338)	E
Musculoskeletal	Fracture of hip	fracture_hip	28(12,54)	39(24,60)	71(52,94)	W
Musculoskeletal	Fracture of hip	fracture_hip	45(28,70)	51(37,68)	121(101,144)	S
Musculoskeletal	Fracture of hip	fracture_hip	19(14,24)	40(34,46)	75(69,82)	E
Musculoskeletal	Fracture of wrist	fracture_wrist	213(164,272)	246(206,291)	353(310,399)	W
Musculoskeletal	Fracture of wrist	fracture_wrist	322(273,377)	340(303,380)	455(417,496)	S
Musculoskeletal	Fracture of wrist	fracture_wrist	160(146,174)	228(215,241)	317(304,330)	E
Musculoskeletal	Giant Cell arteritis	GCA	0(NaN,NaN)	5(1,16)	28(17,43)	W
Musculoskeletal	Giant Cell arteritis	GCA	0(NaN,NaN)	11(5,20)	29(20,40)	S
Musculoskeletal	Giant Cell arteritis	GCA	4(2,7)	9(7,12)	29(25,33)	E

Musculoskeletal	Gout	gout	320(257,395)	531(468,601)	809(742,880)	W
Musculoskeletal	Gout	gout	141(108,180)	255(221,292)	404(365,446)	S
Musculoskeletal	Gout	gout	233(216,250)	405(387,423)	621(602,639)	E
Musculoskeletal	Intervertebral disc disorders	intervert_disc	470(396,555)	538(476,605)	537(483,594)	W
Musculoskeletal	Intervertebral disc disorders	intervert_disc	364(313,421)	399(358,443)	512(470,557)	S
Musculoskeletal	Intervertebral disc disorders	intervert_disc	444(422,467)	525(505,545)	616(598,634)	E
Musculoskeletal	Juvenile arthritis	juv_arth	3(0,17)	2(0,9)	0(NaN,NaN)	W
Musculoskeletal	Juvenile arthritis	juv_arth	6(1,19)	5(1,12)	2(0,7)	S
Musculoskeletal	Juvenile arthritis	juv_arth	4(2,6)	3(2,5)	2(1,3)	E
Musculoskeletal	Lupus erythematosus (local and systemic)	SLE	13(4,34)	24(13,41)	24(14,38)	W
Musculoskeletal	Lupus erythematosus (local and systemic)	SLE	16(7,31)	29(19,42)	30(21,42)	S
Musculoskeletal	Lupus erythematosus (local and systemic)	SLE	22(17,27)	22(18,26)	25(22,29)	E
Musculoskeletal	Osteoarthritis (excl spine)	OA	841(740,952)	1881(1766,2001)	3301(3167,3439)	W
Musculoskeletal	Osteoarthritis (excl spine)	OA	547(484,616)	1353(1277,1431)	2469(2376,2565)	S
Musculoskeletal	Osteoarthritis (excl spine)	OA	609(583,637)	1386(1354,1418)	2479(2443,2515)	E

Musculoskeletal	Osteoporosis	osteoporosis	118(83,163)	320(275,369)	797(734,865)	W
Musculoskeletal	Osteoporosis	osteoporosis	84(61,112)	367(330,408)	852(800,907)	S
Musculoskeletal	Osteoporosis	osteoporosis	99(89,110)	307(293,322)	675(657,694)	E
Musculoskeletal	Polymyalgia Rheumatica	PMR	16(5,37)	43(28,64)	181(151,216)	W
Musculoskeletal	Polymyalgia Rheumatica	PMR	7(2,19)	34(23,48)	109(91,131)	S
Musculoskeletal	Polymyalgia Rheumatica	PMR	6(4,9)	37(32,42)	127(119,135)	E
Musculoskeletal	Postinfective and reactive arthropathies	reactive	11(2,32)	10(4,23)	11(5,22)	W
Musculoskeletal	Postinfective and reactive arthropathies	reactive	7(2,19)	4(1,10)	8(4,16)	S
Musculoskeletal	Postinfective and reactive arthropathies	reactive	10(7,15)	14(11,18)	13(11,16)	E
Musculoskeletal	Psoriatic arthropathy	PSA	56(33,90)	29(16,49)	53(37,73)	W
Musculoskeletal	Psoriatic arthropathy	PSA	68(47,97)	57(42,76)	46(34,61)	S
Musculoskeletal	Psoriatic arthropathy	PSA	35(29,42)	52(46,58)	39(35,44)	E
Musculoskeletal	Rheumatoid Arthritis	RhA	107(73,151)	192(157,233)	281(243,323)	W
Musculoskeletal	Rheumatoid Arthritis	RhA	93(69,123)	153(128,180)	201(175,229)	S
Musculoskeletal	Rheumatoid Arthritis	RhA	74(65,84)	144(134,155)	207(196,217)	E

Musculoskeletal	Scoliosis	scoliosis	61(37,96)	52(35,75)	71(53,93)	W
Musculoskeletal	Scoliosis	scoliosis	48(31,71)	56(42,74)	49(36,64)	S
Musculoskeletal	Scoliosis	scoliosis	52(45,60)	56(50,63)	79(73,85)	E
Musculoskeletal	Sjogren's disease	sjogren	10(2,31)	13(6,26)	27(16,42)	W
Musculoskeletal	Sjogren's disease	sjogren	11(4,24)	29(19,41)	27(19,39)	S
Musculoskeletal	Sjogren's disease	sjogren	7(4,10)	16(13,20)	21(18,24)	E
Musculoskeletal	Spinal stenosis	spinal_stenosis	34(16,63)	70(49,97)	153(126,186)	W
Musculoskeletal	Spinal stenosis	spinal_stenosis	12(4,26)	49(36,67)	134(112,157)	S
Musculoskeletal	Spinal stenosis	spinal_stenosis	50(42,58)	91(83,100)	184(174,194)	E
Musculoskeletal	Spondylolisthesis	spondylolisthesis	31(14,58)	35(21,56)	62(45,83)	W
Musculoskeletal	Spondylolisthesis	spondylolisthesis	19(9,36)	23(14,36)	42(31,56)	S
Musculoskeletal	Spondylolisthesis	spondylolisthesis	29(24,36)	49(43,55)	77(71,84)	E
Musculoskeletal	Spondylosis	spondylosis	281(224,347)	625(559,696)	1063(988,1143)	W
Musculoskeletal	Spondylosis	spondylosis	174(140,215)	431(389,477)	907(851,965)	S
Musculoskeletal	Spondylosis	spondylosis	308(290,327)	586(565,607)	1048(1025,1072)	E

Musculoskeletal	Systemic sclerosis	sys_sclerosis	6(1,22)	9(3,22)	10(4,20)	W
Musculoskeletal	Systemic sclerosis	sys_sclerosis	3(0,12)	5(2,12)	10(5,18)	S
Musculoskeletal	Systemic sclerosis	sys_sclerosis	5(3,7)	7(5,9)	9(7,12)	E
Neurological	Bell's palsy	bells	63(38,99)	88(64,118)	71(53,94)	W
Neurological	Bell's palsy	bells	81(57,111)	74(57,94)	99(81,120)	S
Neurological	Bell's palsy	bells	62(54,71)	68(61,75)	77(71,84)	E
Neurological	Cerebral Palsy	cerebral_palsy	6(1,22)	8(2,20)	4(1,13)	W
Neurological	Cerebral Palsy	cerebral_palsy	11(4,26)	14(7,25)	10(5,17)	S
Neurological	Cerebral Palsy	cerebral_palsy	10(7,14)	8(6,11)	5(3,7)	E
Neurological	Diabetic neurological complications	dm_neuro	9(2,26)	54(36,79)	53(37,74)	W
Neurological	Diabetic neurological complications	dm_neuro	2(0,10)	29(19,44)	43(31,57)	S
Neurological	Diabetic neurological complications	dm_neuro	9(6,13)	21(17,25)	40(35,45)	E
Neurological	Disorders of autonomic nervous system	autonomic_neuro	20(7,44)	24(13,41)	40(27,58)	W
Neurological	Disorders of autonomic nervous system	autonomic_neuro	12(5,25)	26(17,39)	30(20,42)	S
Neurological	Disorders of autonomic nervous system	autonomic_neuro	15(11,20)	28(24,33)	31(27,36)	E
Neurological	Epilepsy	epilepsy	169(126,222)	154(122,192)	156(128,189)	W
Neurological	Epilepsy	epilepsy	144(113,182)	162(136,191)	165(141,191)	S
Neurological	Epilepsy	epilepsy	160(147,174)	162(151,173)	159(150,168)	E
Neurological	Essential tremor	essential_tremor	16(5,39)	29(16,49)	53(37,73)	W
Neurological	Essential tremor	essential_tremor	17(7,33)	7(2,14)	30(20,42)	S
Neurological	Essential tremor	essential_tremor	16(12,21)	26(22,31)	55(50,61)	E
Neurological	Intracranial hypertension	intracranial_htn	10(2,29)	5(1,16)	4(1,12)	W

Neurological	Intracranial hypertension	intracranial_htn	4(0,15)	3(1,10)	2(1,7)	S
Neurological	Intracranial hypertension	intracranial_htn	5(3,8)	5(4,8)	2(1,4)	E
Neurological	Migraine	migraine	1134(1019,1257)	1034(951,1122)	704(644,769)	W
Neurological	Migraine	migraine	792(719,871)	778(723,836)	698(650,748)	S
Neurological	Migraine	migraine	1085(1051,1120)	964(938,990)	804(784,824)	E
Neurological	Motor neuron disease	MND	3(0,17)	8(2,20)	6(2,15)	W
Neurological	Motor neuron disease	MND	0(NaN,NaN)	6(2,13)	7(3,14)	S
Neurological	Motor neuron disease	MND	2(0,4)	6(4,9)	8(6,10)	E
Neurological	Multiple sclerosis	MS	43(23,73)	57(39,81)	39(26,57)	W
Neurological	Multiple sclerosis	MS	60(41,86)	71(55,90)	49(37,64)	S
Neurological	Multiple sclerosis	MS	50(42,58)	57(51,63)	37(33,42)	E
Neurological	Myasthenia gravis	myasthenia	0(NaN,NaN)	5(1,16)	7(2,17)	W
Neurological	Myasthenia gravis	myasthenia	6(1,19)	4(1,11)	11(6,19)	S
Neurological	Myasthenia gravis	myasthenia	3(2,6)	7(5,10)	9(7,12)	E
Neurological	Parkinson's disease	Parkinsons	4(0,21)	25(13,44)	87(67,112)	W
Neurological	Parkinson's disease	Parkinsons	6(1,17)	17(10,28)	68(53,85)	S
Neurological	Parkinson's disease	Parkinsons	6(3,9)	23(19,28)	73(66,79)	E
Neurological	Peripheral neuropathies (excl. cranial nerve, carpal tunnel syndromes)	periph_neuro	238(186,300)	429(375,489)	507(456,563)	W
Neurological	Peripheral neuropathies (excl. cranial nerve, carpal tunnel syndromes)	periph_neuro	139(108,177)	248(216,283)	285(254,319)	S
Neurological	Peripheral neuropathies (excl. cranial nerve, carpal tunnel syndromes)	periph_neuro	217(202,234)	312(297,328)	376(362,390)	E
Neurological	Postviral fatigue syndrome, neurasthenia and fibromyalgia	chronic_fatigue	549(470,638)	496(438,559)	428(381,479)	W
Neurological	Postviral fatigue syndrome, neurasthenia and fibromyalgia	chronic_fatigue	213(175,256)	256(225,291)	234(207,264)	S

Neurological	Postviral fatigue syndrome, neurasthenia and fibromyalgia	chronic_fatigue	229(213,245)	242(229,255)	212(201,222)	E
Neurological	Trigeminal neuralgia	trigem_neur	38(19,66)	86(64,115)	126(101,155)	W
Neurological	Trigeminal neuralgia	trigem_neur	29(16,47)	55(41,73)	54(41,69)	S
Neurological	Trigeminal neuralgia	trigem_neur	46(39,53)	70(63,77)	90(84,97)	E
Perinatal	Bacterial sepsis of newborn	sepsis_newborn	--	--	--	W
Perinatal	Bacterial sepsis of newborn	sepsis_newborn	--	--	--	S
Perinatal	Bacterial sepsis of newborn	sepsis_newborn	--	--	--	E
Perinatal	Congenital malformations of cardiac septa	congenital_septal	27(12,53)	17(8,33)	16(8,28)	W
Perinatal	Congenital malformations of cardiac septa	congenital_septal	35(20,55)	29(19,43)	25(17,37)	S
Perinatal	Congenital malformations of cardiac septa	congenital_septal	32(26,38)	24(20,28)	22(18,25)	E
Perinatal	Down's syndrome	downs	--	--	--	W
Perinatal	Down's syndrome	downs	--	--	--	S
Perinatal	Down's syndrome	downs	--	--	--	E
Perinatal	High birth weight	HBW	--	--	--	W
Perinatal	High birth weight	HBW	--	--	--	S
Perinatal	High birth weight	HBW	--	--	--	E
Perinatal	Intrauterine hypoxia	intrauterine_hypoxia	--	--	--	W
Perinatal	Intrauterine hypoxia	intrauterine_hypoxia	--	--	--	S
Perinatal	Intrauterine hypoxia	intrauterine_hypoxia	--	--	--	E
Perinatal	Neonatal jaundice (excl haemolytic dz of the newborn)	neo_jaundice	--	--	--	W
Perinatal	Neonatal jaundice (excl haemolytic dz of the newborn)	neo_jaundice	--	--	--	S
Perinatal	Neonatal jaundice (excl haemolytic dz of the newborn)	neo_jaundice	--	--	--	E
Perinatal	Patent ductus arteriosus	PDA	--	--	--	W
Perinatal	Patent ductus arteriosus	PDA	--	--	--	S

Perinatal	Patent ductus arteriosus	PDA	--	--	--	E
Perinatal	Post-term infant	post_term	--	--	--	W
Perinatal	Post-term infant	post_term	--	--	--	S
Perinatal	Post-term infant	post_term	--	--	--	E
Perinatal	Prematurity	prematurity	--	--	--	W
Perinatal	Prematurity	prematurity	--	--	--	S
Perinatal	Prematurity	prematurity	--	--	--	E
Perinatal	Respiratory distress of newborn	RDN	--	--	--	W
Perinatal	Respiratory distress of newborn	RDN	--	--	--	S
Perinatal	Respiratory distress of newborn	RDN	--	--	--	E
Perinatal	Slow fetal growth or low birth weight	LBW	--	--	--	W
Perinatal	Slow fetal growth or low birth weight	LBW	--	--	--	S
Perinatal	Slow fetal growth or low birth weight	LBW	--	--	--	E
Perinatal	Spina bifida	spina_bifida	23(9,48)	9(3,21)	2(0,9)	W
Perinatal	Spina bifida	spina_bifida	16(7,32)	23(14,35)	20(12,30)	S
Perinatal	Spina bifida	spina_bifida	19(15,25)	17(13,20)	14(11,17)	E
Psychiatric	Alcohol Problems	alc_problems	173(128,228)	220(181,266)	195(163,232)	W
Psychiatric	Alcohol Problems	alc_problems	511(449,580)	737(680,798)	731(679,786)	S
Psychiatric	Alcohol Problems	alc_problems	421(399,444)	474(455,493)	437(422,453)	E
Psychiatric	Alzheimer's disease	alzheimer	3(0,17)	3(0,12)	44(30,63)	W
Psychiatric	Alzheimer's disease	alzheimer	0(NaN,NaN)	8(3,17)	53(41,69)	S
Psychiatric	Alzheimer's disease	alzheimer	1(0,3)	8(6,11)	39(35,44)	E
Psychiatric	Anorexia and bulimia nervosa	eating_dz	36(19,63)	3(0,12)	1(0,8)	W
Psychiatric	Anorexia and bulimia nervosa	eating_dz	65(46,90)	29(19,42)	15(9,24)	S

Psychiatric	Anorexia and bulimia nervosa	eating_dz	38(32,45)	21(17,25)	12(10,15)	E
Psychiatric	Anxiety disorders	anxiety	1592(1453,1741)	1464(1363,1570)	1440(1353,1532)	W
Psychiatric	Anxiety disorders	anxiety	1267(1171,1369)	1302(1228,1378)	1232(1167,1299)	S
Psychiatric	Anxiety disorders	anxiety	1016(982,1050)	1051(1024,1079)	1038(1015,1061)	E
Psychiatric	Autism and Asperger's syndrome	autism	13(4,34)	2(0,12)	2(0,9)	W
Psychiatric	Autism and Asperger's syndrome	autism	13(5,28)	8(3,17)	4(1,11)	S
Psychiatric	Autism and Asperger's syndrome	autism	4(2,7)	2(1,3)	1(0,2)	E
Psychiatric	Bipolar affective disorder and mania	BAD	64(38,100)	35(21,55)	57(41,78)	W
Psychiatric	Bipolar affective disorder and mania	BAD	51(33,75)	47(34,63)	65(51,83)	S
Psychiatric	Bipolar affective disorder and mania	BAD	50(42,58)	50(44,57)	49(44,54)	E
Psychiatric	Delirium, not induced by alcohol and other psychoactive substances	delirium	4(0,21)	9(2,22)	27(17,43)	W
Psychiatric	Delirium, not induced by alcohol and other psychoactive substances	delirium	6(1,19)	6(2,14)	23(15,35)	S
Psychiatric	Delirium, not induced by alcohol and other psychoactive substances	delirium	4(2,7)	10(8,14)	25(21,29)	E
Psychiatric	Dementia	dementia	6(1,22)	17(8,32)	111(88,139)	W
Psychiatric	Dementia	dementia	2(0,13)	21(13,34)	131(110,154)	S
Psychiatric	Dementia	dementia	8(5,12)	26(22,31)	94(87,101)	E
Psychiatric	Dementia (excluding Alzheimer's)	dementia_ex_Alz	6(1,22)	15(7,30)	106(83,133)	W
Psychiatric	Dementia (excluding Alzheimer's)	dementia_ex_Alz	2(0,13)	21(13,34)	124(104,147)	S
Psychiatric	Dementia (excluding Alzheimer's)	dementia_ex_Alz	8(5,12)	25(21,30)	88(81,95)	E

Psychiatric	Depression	depression	2218(2053,2391)	2229(2104,2359)	1879(1779,1983)	W
Psychiatric	Depression	depression	1714(1603,1832)	1740(1655,1829)	1618(1543,1695)	S
Psychiatric	Depression	depression	1402(1363,1443)	1349(1318,1381)	1207(1183,1233)	E
Psychiatric	Hyperkinetic disorders	ADHD	0(NaN,NaN)	2(0,12)	2(0,9)	W
Psychiatric	Hyperkinetic disorders	ADHD	5(1,17)	1(0,7)	1(0,5)	S
Psychiatric	Hyperkinetic disorders	ADHD	2(1,4)	1(0,1)	1(0,2)	E
Psychiatric	Intellectual disability	intell_dz	17(6,40)	11(4,25)	1(0,8)	W
Psychiatric	Intellectual disability	intell_dz	10(3,24)	18(10,29)	8(3,15)	S
Psychiatric	Intellectual disability	intell_dz	18(14,24)	12(9,15)	4(3,6)	E
Psychiatric	Obsessive-compulsive disorder	ocd	34(16,62)	37(22,57)	31(19,47)	W
Psychiatric	Obsessive-compulsive disorder	ocd	35(20,55)	14(8,24)	20(12,30)	S
Psychiatric	Obsessive-compulsive disorder	ocd	36(30,43)	32(28,37)	20(17,23)	E
Psychiatric	Other psychoactive substance misuse	substance_misuse	65(39,102)	62(42,88)	42(28,61)	W
Psychiatric	Other psychoactive substance misuse	substance_misuse	106(79,141)	59(44,78)	42(31,56)	S
Psychiatric	Other psychoactive substance misuse	substance_misuse	81(72,91)	53(47,60)	41(36,46)	E
Psychiatric	Personality disorders	PD	26(11,52)	30(17,50)	20(11,34)	W
Psychiatric	Personality disorders	PD	47(30,70)	61(46,80)	58(45,75)	S
Psychiatric	Personality disorders	PD	44(37,52)	31(26,36)	22(19,26)	E
Psychiatric	Schizophrenia, schizotypal and delusional disorders	schizo	48(26,80)	56(38,81)	27(17,43)	W
Psychiatric	Schizophrenia, schizotypal and delusional disorders	schizo	69(47,96)	58(43,76)	41(30,55)	S
Psychiatric	Schizophrenia, schizotypal and delusional disorders	schizo	62(54,71)	42(37,48)	38(33,43)	E

Respiratory	Allergic and chronic rhinitis	allergic_rhinitis	1847(1696,2007)	1581(1476,1692)	1582(1490,1679)	W
Respiratory	Allergic and chronic rhinitis	allergic_rhinitis	1297(1198,1402)	1128(1059,1201)	969(911,1030)	S
Respiratory	Allergic and chronic rhinitis	allergic_rhinitis	1336(1297,1376)	1326(1295,1358)	1317(1291,1344)	E
Respiratory	Asbestosis	asbestosis	0(NaN,NaN)	8(2,21)	35(22,53)	W
Respiratory	Asbestosis	asbestosis	0(NaN,NaN)	4(1,11)	24(15,36)	S
Respiratory	Asbestosis	asbestosis	0(0,2)	1(0,3)	12(10,15)	E
Respiratory	Aspiration pneumonitis	aspiration_pneumo	7(1,27)	11(4,25)	18(9,32)	W
Respiratory	Aspiration pneumonitis	aspiration_pneumo	5(1,17)	9(4,19)	27(18,39)	S
Respiratory	Aspiration pneumonitis	aspiration_pneumo	7(4,10)	14(11,18)	21(17,24)	E
Respiratory	Asthma	asthma	1509(1373,1654)	1290(1195,1391)	1483(1394,1577)	W
Respiratory	Asthma	asthma	1199(1104,1299)	1131(1062,1203)	1027(967,1089)	S
Respiratory	Asthma	asthma	1336(1297,1376)	1235(1205,1266)	1200(1175,1225)	E
Respiratory	Bronchiectasis	bronchiectasis	19(7,41)	38(23,59)	117(93,146)	W
Respiratory	Bronchiectasis	bronchiectasis	28(16,47)	49(35,66)	109(90,131)	S
Respiratory	Bronchiectasis	bronchiectasis	26(21,32)	58(51,65)	139(131,148)	E
Respiratory	Chronic sinusitis	sinusitis	2115(1955,2285)	1885(1771,2004)	1948(1846,2055)	W
Respiratory	Chronic sinusitis	sinusitis	893(812,979)	862(802,925)	811(758,866)	S
Respiratory	Chronic sinusitis	sinusitis	1193(1156,1230)	1273(1243,1303)	1272(1246,1298)	E
Respiratory	COPD	COPD	85(55,124)	204(166,247)	586(530,646)	W
Respiratory	COPD	COPD	66(45,93)	251(219,287)	499(457,543)	S
Respiratory	COPD	COPD	93(83,104)	251(238,265)	537(520,554)	E

Respiratory	COPD_excl_bronchitis_NOS	COPD_excl_bronchitis_NOS	78(50,116)	196(159,238)	576(520,636)	W
Respiratory	COPD_excl_bronchitis_NOS	COPD_excl_bronchitis_NOS	63(42,89)	248(216,283)	495(454,540)	S
Respiratory	COPD_excl_bronchitis_NOS	COPD_excl_bronchitis_NOS	86(77,97)	243(229,257)	522(505,539)	E
Respiratory	Hypertrophy of nasal turbinates	hyper_nasal_turbs	48(26,80)	45(29,68)	20(11,34)	W
Respiratory	Hypertrophy of nasal turbinates	hyper_nasal_turbs	21(10,38)	38(26,53)	18(10,28)	S
Respiratory	Hypertrophy of nasal turbinates	hyper_nasal_turbs	63(55,72)	53(47,60)	45(40,50)	E
Respiratory	Nasal polyp	nasal_polyp	142(102,193)	194(158,237)	230(196,269)	W
Respiratory	Nasal polyp	nasal_polyp	112(83,146)	157(131,186)	221(193,251)	S
Respiratory	Nasal polyp	nasal_polyp	132(120,145)	173(161,185)	230(219,242)	E
Respiratory	Other interstitial pulmonary diseases with fibrosis	pulm_fibrosis	10(2,29)	17(7,33)	61(44,82)	W
Respiratory	Other interstitial pulmonary diseases with fibrosis	pulm_fibrosis	6(1,19)	14(7,24)	51(38,67)	S
Respiratory	Other interstitial pulmonary diseases with fibrosis	pulm_fibrosis	6(4,10)	11(9,15)	42(37,47)	E
Respiratory	Pleural effusion	pleural_effusion	44(23,75)	103(77,135)	178(148,213)	W
Respiratory	Pleural effusion	pleural_effusion	32(18,53)	72(56,92)	172(148,199)	S
Respiratory	Pleural effusion	pleural_effusion	49(42,57)	95(87,104)	170(161,180)	E
Respiratory	Pleural plaque	pleural_plaque	4(0,21)	12(5,27)	49(33,69)	W
Respiratory	Pleural plaque	pleural_plaque	0(NaN,NaN)	7(3,16)	33(22,46)	S
Respiratory	Pleural plaque	pleural_plaque	3(1,5)	10(7,13)	44(39,49)	E
Respiratory	Pneumothorax	pneumothorax	29(13,56)	23(12,41)	33(21,50)	W
Respiratory	Pneumothorax	pneumothorax	52(34,78)	63(47,83)	81(65,101)	S
Respiratory	Pneumothorax	pneumothorax	45(38,53)	47(41,53)	54(49,60)	E
Respiratory	Pulmonary collapse (excl pneumothorax)	pulm_collapse	22(9,46)	27(15,46)	48(33,67)	W
Respiratory	Pulmonary collapse (excl pneumothorax)	pulm_collapse	16(6,32)	42(30,59)	67(53,85)	S

Respiratory	Pulmonary collapse (excl pneumothorax)	pulm_collapse	18(14,24)	38(33,44)	65(59,71)	E
Respiratory	Respiratory failure	resp_failure	13(4,34)	24(12,42)	78(59,102)	W
Respiratory	Respiratory failure	resp_failure	10(3,24)	18(10,29)	32(22,45)	S
Respiratory	Respiratory failure	resp_failure	16(12,21)	37(32,43)	73(67,80)	E
Respiratory	Sleep apnoea	sleep_apnoea	122(85,170)	175(140,216)	179(148,214)	W
Respiratory	Sleep apnoea	sleep_apnoea	108(80,142)	174(146,204)	174(149,202)	S
Respiratory	Sleep apnoea	sleep_apnoea	163(149,177)	211(199,225)	205(194,216)	E
Skin	Acne	acne	464(392,547)	219(181,262)	116(92,144)	W
Skin	Acne	acne	522(461,589)	294(260,332)	137(116,161)	S
Skin	Acne	acne	485(462,509)	248(235,262)	124(116,132)	E
Skin	Actinic keratosis	actinic_keratosis	126(89,174)	396(343,454)	1068(992,1148)	W
Skin	Actinic keratosis	actinic_keratosis	73(51,101)	154(128,182)	313(280,349)	S
Skin	Actinic keratosis	actinic_keratosis	104(93,115)	311(295,326)	717(697,737)	E
Skin	Alopecia areata	alopecia_areata	33(16,61)	24(13,40)	27(17,43)	W
Skin	Alopecia areata	alopecia_areata	40(24,62)	26(17,39)	34(24,47)	S
Skin	Alopecia areata	alopecia_areata	44(37,52)	42(36,48)	32(28,36)	E
Skin	Dermatitis (atopc/contact/other/unspecified)	dermatitis	2768(2584,2962)	2751(2612,2896)	3049(2920,3182)	W
Skin	Dermatitis (atopc/contact/other/unspecified)	dermatitis	1403(1301,1511)	1284(1210,1360)	1319(1251,1390)	S
Skin	Dermatitis (atopc/contact/other/unspecified)	dermatitis	1696(1652,1740)	1738(1702,1774)	1881(1849,1912)	E
Skin	Hidradenitis suppurativa	hidradenitis	47(26,77)	50(34,72)	17(9,29)	W
Skin	Hidradenitis suppurativa	hidradenitis	45(29,67)	20(12,31)	11(6,19)	S
Skin	Hidradenitis suppurativa	hidradenitis	18(14,23)	12(9,15)	8(6,11)	E
Skin	Keratitis	keratitis	77(49,116)	114(87,146)	101(78,127)	W
Skin	Keratitis	keratitis	87(63,117)	64(48,82)	77(61,96)	S

Skin	Keratitis	keratitis	58(50,67)	65(58,72)	81(75,88)	E
Skin	Lichen planus	lichen_planus	54(31,89)	73(52,99)	117(93,145)	W
Skin	Lichen planus	lichen_planus	39(23,60)	66(50,85)	89(72,108)	S
Skin	Lichen planus	lichen_planus	71(62,81)	107(98,116)	149(141,158)	E
Skin	Pilonidal cyst/sinus	pilonidal	138(98,188)	60(41,85)	48(33,67)	W
Skin	Pilonidal cyst/sinus	pilonidal	165(129,206)	97(77,121)	66(51,84)	S
Skin	Pilonidal cyst/sinus	pilonidal	98(87,109)	76(69,84)	57(51,63)	E
Skin	Psoriasis	psoriasis	424(353,505)	397(345,455)	449(400,502)	W
Skin	Psoriasis	psoriasis	421(365,483)	356(318,398)	390(354,430)	S
Skin	Psoriasis	psoriasis	328(309,348)	378(361,395)	410(395,425)	E
Skin	Rosacea	rosacea	472(398,556)	494(437,557)	505(454,561)	W
Skin	Rosacea	rosacea	309(262,361)	331(295,371)	337(303,373)	S
Skin	Rosacea	rosacea	329(310,349)	397(380,414)	418(403,433)	E
Skin	Seborrheic dermatitis	seb_derm	780(683,886)	751(679,829)	839(772,910)	W
Skin	Seborrheic dermatitis	seb_derm	290(244,343)	268(234,304)	237(209,269)	S
Skin	Seborrheic dermatitis	seb_derm	591(565,617)	574(553,595)	599(582,617)	E
Skin	Urticaria	urticaria	527(449,615)	472(416,533)	548(495,606)	W
Skin	Urticaria	urticaria	191(155,232)	238(207,271)	211(185,240)	S
Skin	Urticaria	urticaria	419(397,441)	432(415,450)	442(427,457)	E
Skin	Vitiligo	vitiligo	54(31,87)	42(27,64)	38(25,56)	W
Skin	Vitiligo	vitiligo	26(14,45)	25(15,38)	21(13,32)	S
Skin	Vitiligo	vitiligo	39(33,47)	38(33,44)	39(34,43)	E

Table S9. Sex-standardised period prevalence per 10,000 persons obtained in UKB in any source, in UKB in EHR stratified by deprivation quintile at UKB entry

group	phenotype	variable_name	UKB_EHR_4049	UKB_EHR_5059	UKB_EHR_6069	Townsend_q
Benign neoplasm or Carcinoma in situ	Benign neoplasm and polyp of uterus	benign_uterus	177(149,209)	230(206,255)	234(214,256)	least_depr
Benign neoplasm or Carcinoma in situ	Benign neoplasm and polyp of uterus	benign_uterus	167(140,198)	222(198,247)	247(226,269)	low_depr
Benign neoplasm or Carcinoma in situ	Benign neoplasm and polyp of uterus	benign_uterus	157(132,186)	234(210,260)	253(232,276)	medium
Benign neoplasm or Carcinoma in situ	Benign neoplasm and polyp of uterus	benign_uterus	165(141,192)	225(202,250)	240(219,263)	high_depr
Benign neoplasm or Carcinoma in situ	Benign neoplasm and polyp of uterus	benign_uterus	175(151,202)	224(201,249)	249(226,274)	most_depr
Benign neoplasm or Carcinoma in situ	Benign neoplasm of brain and other parts of central nervous system	benign_brain	24(14,38)	38(28,50)	49(40,60)	least_depr
Benign neoplasm or Carcinoma in situ	Benign neoplasm of brain and other parts of central nervous system	benign_brain	31(19,47)	38(28,50)	57(47,68)	low_depr
Benign neoplasm or Carcinoma in situ	Benign neoplasm of brain and other parts of central nervous system	benign_brain	34(22,50)	49(38,62)	56(46,67)	medium
Benign neoplasm or Carcinoma in situ	Benign neoplasm of brain and other parts of central nervous system	benign_brain	39(27,54)	39(29,51)	52(42,64)	high_depr
Benign neoplasm or Carcinoma in situ	Benign neoplasm of brain and other parts of central nervous system	benign_brain	37(27,51)	40(30,52)	50(39,62)	most_depr
Benign neoplasm or Carcinoma in situ	Benign neoplasm of colon, rectum, anus and anal canal	benign_colon	242(207,281)	502(464,543)	792(752,832)	least_depr

Benign neoplasm or Carcinoma in situ	Benign neoplasm of colon, rectum, anus and anal canal	benign_colon	233(198,271)	490(452,530)	880(839,923)	low_depr
Benign neoplasm or Carcinoma in situ	Benign neoplasm of colon, rectum, anus and anal canal	benign_colon	252(217,291)	532(493,574)	845(804,888)	medium
Benign neoplasm or Carcinoma in situ	Benign neoplasm of colon, rectum, anus and anal canal	benign_colon	267(234,304)	559(519,601)	941(896,989)	high_depr
Benign neoplasm or Carcinoma in situ	Benign neoplasm of colon, rectum, anus and anal canal	benign_colon	300(268,335)	619(578,663)	1101(1049,1154)	most_depr
Benign neoplasm or Carcinoma in situ	Benign neoplasm of ovary	benign_ovary	291(254,331)	238(213,264)	184(166,204)	least_depr
Benign neoplasm or Carcinoma in situ	Benign neoplasm of ovary	benign_ovary	270(235,308)	214(191,239)	189(171,208)	low_depr
Benign neoplasm or Carcinoma in situ	Benign neoplasm of ovary	benign_ovary	277(243,315)	239(215,265)	188(169,207)	medium
Benign neoplasm or Carcinoma in situ	Benign neoplasm of ovary	benign_ovary	311(278,348)	253(228,279)	179(160,199)	high_depr
Benign neoplasm or Carcinoma in situ	Benign neoplasm of ovary	benign_ovary	296(265,331)	281(254,309)	215(193,238)	most_depr
Benign neoplasm or Carcinoma in situ	Benign neoplasm of stomach and duodenum	benign_stomach	27(16,41)	92(76,110)	161(144,180)	least_depr
Benign neoplasm or Carcinoma in situ	Benign neoplasm of stomach and duodenum	benign_stomach	41(27,58)	101(84,119)	179(161,199)	low_depr
Benign neoplasm or Carcinoma in situ	Benign neoplasm of stomach and duodenum	benign_stomach	28(17,42)	97(81,115)	182(164,203)	medium
Benign neoplasm or Carcinoma in situ	Benign neoplasm of stomach and duodenum	benign_stomach	59(44,77)	99(83,117)	179(159,200)	high_depr

Benign neoplasm or Carcinoma in situ	Benign neoplasm of stomach and duodenum	benign_stomach	52(39,68)	109(92,128)	192(171,215)	most_depr
Benign neoplasm or Carcinoma in situ	Carcinoma in situ_cervical	cin_cervical	1504(1419,1591)	1294(1237,1353)	1050(1006,1095)	least_depr
Benign neoplasm or Carcinoma in situ	Carcinoma in situ_cervical	cin_cervical	1515(1431,1603)	1371(1312,1431)	1114(1069,1160)	low_depr
Benign neoplasm or Carcinoma in situ	Carcinoma in situ_cervical	cin_cervical	1357(1280,1437)	1284(1228,1342)	1048(1004,1093)	medium
Benign neoplasm or Carcinoma in situ	Carcinoma in situ_cervical	cin_cervical	1410(1338,1486)	1273(1218,1331)	1116(1069,1164)	high_depr
Benign neoplasm or Carcinoma in situ	Carcinoma in situ_cervical	cin_cervical	1289(1222,1359)	1234(1179,1292)	1050(1002,1100)	most_depr
Benign neoplasm or Carcinoma in situ	Haemangioma, any site	haemangioma	172(143,205)	196(173,221)	196(176,216)	least_depr
Benign neoplasm or Carcinoma in situ	Haemangioma, any site	haemangioma	152(125,183)	170(148,194)	180(161,199)	low_depr
Benign neoplasm or Carcinoma in situ	Haemangioma, any site	haemangioma	142(116,171)	197(174,223)	201(181,222)	medium
Benign neoplasm or Carcinoma in situ	Haemangioma, any site	haemangioma	131(109,157)	166(145,189)	164(145,184)	high_depr
Benign neoplasm or Carcinoma in situ	Haemangioma, any site	haemangioma	114(95,137)	131(112,151)	162(142,183)	most_depr
Benign neoplasm or Carcinoma in situ	Leiomyoma of uterus	leiomyoma	443(398,492)	503(468,541)	400(373,428)	least_depr
Benign neoplasm or Carcinoma in situ	Leiomyoma of uterus	leiomyoma	463(417,513)	479(444,515)	384(358,412)	low_depr

Benign neoplasm or Carcinoma in situ	Leiomyoma of uterus	leiomyoma	456(412,504)	503(468,540)	394(367,422)	medium
Benign neoplasm or Carcinoma in situ	Leiomyoma of uterus	leiomyoma	502(459,547)	555(518,593)	388(361,417)	high_depr
Benign neoplasm or Carcinoma in situ	Leiomyoma of uterus	leiomyoma	574(529,620)	509(474,547)	358(330,387)	most_depr
Cancers	Hodgkin Lymphoma	hodgkins	15(8,28)	12(7,20)	16(11,23)	least_depr
Cancers	Hodgkin Lymphoma	hodgkins	12(5,23)	15(9,24)	14(10,21)	low_depr
Cancers	Hodgkin Lymphoma	hodgkins	12(5,22)	10(5,17)	7(4,12)	medium
Cancers	Hodgkin Lymphoma	hodgkins	13(7,24)	10(5,17)	9(5,15)	high_depr
Cancers	Hodgkin Lymphoma	hodgkins	12(7,21)	12(7,19)	14(8,21)	most_depr
Cancers	Leukaemia	leukaemia	16(8,29)	25(17,35)	45(36,56)	least_depr
Cancers	Leukaemia	leukaemia	19(10,32)	23(16,33)	43(34,53)	low_depr
Cancers	Leukaemia	leukaemia	13(6,24)	19(12,29)	48(39,60)	medium
Cancers	Leukaemia	leukaemia	11(5,20)	29(21,40)	51(41,63)	high_depr
Cancers	Leukaemia	leukaemia	8(3,16)	23(16,33)	47(37,59)	most_depr
Cancers	Monoclonal gammopathy of undetermined significance (MGUS)	MGUS	7(2,17)	13(8,21)	25(18,33)	least_depr
Cancers	Monoclonal gammopathy of undetermined significance (MGUS)	MGUS	4(1,12)	12(7,20)	34(27,43)	low_depr
Cancers	Monoclonal gammopathy of undetermined significance (MGUS)	MGUS	3(0,11)	11(6,18)	28(21,37)	medium
Cancers	Monoclonal gammopathy of undetermined significance (MGUS)	MGUS	2(0,8)	13(8,22)	28(20,37)	high_depr
Cancers	Monoclonal gammopathy of undetermined significance (MGUS)	MGUS	6(2,13)	10(6,17)	35(26,46)	most_depr
Cancers	Multiple myeloma and malignant plasma cell neoplasms	plasmacell	19(10,33)	10(5,17)	24(17,31)	least_depr
Cancers	Multiple myeloma and malignant plasma cell neoplasms	plasmacell	3(0,10)	11(6,19)	32(25,41)	low_depr

Cancers	Multiple myeloma and malignant plasma cell neoplasms	plasmacell	6(2,15)	11(6,18)	21(15,29)	medium
Cancers	Multiple myeloma and malignant plasma cell neoplasms	plasmacell	2(0,8)	8(4,15)	22(16,31)	high_depr
Cancers	Multiple myeloma and malignant plasma cell neoplasms	plasmacell	3(1,9)	14(9,22)	22(15,30)	most_depr
Cancers	Myelodysplastic syndromes	MDS	3(0,10)	3(1,8)	7(4,12)	least_depr
Cancers	Myelodysplastic syndromes	MDS	3(0,9)	10(5,17)	8(4,13)	low_depr
Cancers	Myelodysplastic syndromes	MDS	3(0,10)	7(3,13)	9(5,15)	medium
Cancers	Myelodysplastic syndromes	MDS	3(1,10)	6(3,12)	10(6,16)	high_depr
Cancers	Myelodysplastic syndromes	MDS	2(0,7)	6(2,11)	9(5,16)	most_depr
Cancers	Non-Hodgkin Lymphoma	NHL	19(10,32)	38(28,51)	83(71,97)	least_depr
Cancers	Non-Hodgkin Lymphoma	NHL	21(12,35)	32(23,44)	78(66,92)	low_depr
Cancers	Non-Hodgkin Lymphoma	NHL	22(13,36)	41(30,53)	72(60,85)	medium
Cancers	Non-Hodgkin Lymphoma	NHL	23(14,35)	39(29,51)	80(67,95)	high_depr
Cancers	Non-Hodgkin Lymphoma	NHL	33(23,46)	44(34,57)	79(66,94)	most_depr
Cancers	Polycythaemia vera	PCV	3(0,10)	11(6,19)	12(8,18)	least_depr
Cancers	Polycythaemia vera	PCV	3(0,11)	11(6,19)	18(12,25)	low_depr
Cancers	Polycythaemia vera	PCV	8(3,17)	11(6,19)	17(12,24)	medium
Cancers	Polycythaemia vera	PCV	5(2,12)	12(7,20)	23(16,31)	high_depr
Cancers	Polycythaemia vera	PCV	6(2,13)	18(12,27)	19(13,28)	most_depr
Cancers	Primary Malignancy_Adrenal gland	pri_adrenal	1(0,7)	0(NaN,NaN)	1(0,4)	least_depr
Cancers	Primary Malignancy_Adrenal gland	pri_adrenal	0(NaN,NaN)	2(0,6)	1(0,4)	low_depr
Cancers	Primary Malignancy_Adrenal gland	pri_adrenal	1(0,8)	2(0,7)	1(0,3)	medium
Cancers	Primary Malignancy_Adrenal gland	pri_adrenal	0(NaN,NaN)	2(0,5)	2(1,6)	high_depr
Cancers	Primary Malignancy_Adrenal gland	pri_adrenal	1(0,5)	4(1,9)	2(0,5)	most_depr

Cancers	Primary Malignancy_biliary tract	pri_biliary	0(NaN,NaN)	6(2,11)	9(5,14)	least_depr
Cancers	Primary Malignancy_biliary tract	pri_biliary	2(0,9)	2(0,7)	12(8,18)	low_depr
Cancers	Primary Malignancy_biliary tract	pri_biliary	1(0,8)	5(2,10)	9(5,14)	medium
Cancers	Primary Malignancy_biliary tract	pri_biliary	2(0,8)	4(1,9)	10(6,16)	high_depr
Cancers	Primary Malignancy_biliary tract	pri_biliary	1(0,5)	4(2,9)	12(7,19)	most_depr
Cancers	Primary Malignancy_Bladder	pri_bladder	4(1,13)	29(20,40)	83(70,97)	least_depr
Cancers	Primary Malignancy_Bladder	pri_bladder	8(3,17)	30(20,41)	92(79,107)	low_depr
Cancers	Primary Malignancy_Bladder	pri_bladder	9(4,19)	22(15,32)	94(80,109)	medium
Cancers	Primary Malignancy_Bladder	pri_bladder	13(6,23)	35(26,47)	80(67,95)	high_depr
Cancers	Primary Malignancy_Bladder	pri_bladder	15(8,24)	35(25,47)	89(74,105)	most_depr
Cancers	Primary Malignancy_Bone and articular cartilage	pri_bone	2(0,9)	7(3,13)	4(2,8)	least_depr
Cancers	Primary Malignancy_Bone and articular cartilage	pri_bone	0(NaN,NaN)	5(2,11)	5(2,10)	low_depr
Cancers	Primary Malignancy_Bone and articular cartilage	pri_bone	2(0,8)	8(4,15)	5(3,10)	medium
Cancers	Primary Malignancy_Bone and articular cartilage	pri_bone	4(1,11)	2(0,6)	10(6,15)	high_depr
Cancers	Primary Malignancy_Bone and articular cartilage	pri_bone	5(2,12)	2(0,6)	7(4,13)	most_depr
Cancers	Primary Malignancy_Brain, Other CNS and Intracranial	pri_brain	13(6,24)	17(10,26)	18(13,25)	least_depr
Cancers	Primary Malignancy_Brain, Other CNS and Intracranial	pri_brain	12(5,24)	10(5,18)	14(9,20)	low_depr
Cancers	Primary Malignancy_Brain,	pri_brain	2(0,8)	17(10,25)	17(11,24)	medium

	Other CNS and Intracranial					
Cancers	Primary Malignancy_Brain, Other CNS and Intracranial	pri_brain	11(5,21)	14(9,22)	16(11,24)	high_depr
Cancers	Primary Malignancy_Brain, Other CNS and Intracranial	pri_brain	9(4,17)	13(8,20)	21(15,30)	most_depr
Cancers	Primary Malignancy_Breast	pri_breast	170(142,201)	285(258,313)	416(389,445)	least_depr
Cancers	Primary Malignancy_Breast	pri_breast	163(136,194)	261(235,288)	403(377,432)	low_depr
Cancers	Primary Malignancy_Breast	pri_breast	167(140,196)	238(214,264)	392(366,421)	medium
Cancers	Primary Malignancy_Breast	pri_breast	161(137,188)	265(240,292)	417(388,447)	high_depr
Cancers	Primary Malignancy_Breast	pri_breast	128(108,152)	254(229,281)	396(367,427)	most_depr
Cancers	Primary Malignancy_Cervical	pri_cervical	15(8,26)	15(9,22)	11(7,16)	least_depr
Cancers	Primary Malignancy_Cervical	pri_cervical	14(7,25)	20(14,29)	17(12,23)	low_depr
Cancers	Primary Malignancy_Cervical	pri_cervical	8(3,17)	17(11,25)	13(8,19)	medium
Cancers	Primary Malignancy_Cervical	pri_cervical	17(10,27)	13(8,20)	16(11,23)	high_depr
Cancers	Primary Malignancy_Cervical	pri_cervical	18(11,27)	18(11,26)	22(15,30)	most_depr
Cancers	Primary Malignancy_colorectal and anus	pri_bowel	30(18,45)	99(83,118)	171(153,190)	least_depr
Cancers	Primary Malignancy_colorectal and anus	pri_bowel	32(20,48)	83(68,101)	193(174,214)	low_depr
Cancers	Primary Malignancy_colorectal and anus	pri_bowel	23(13,36)	85(69,102)	158(141,178)	medium
Cancers	Primary Malignancy_colorectal and anus	pri_bowel	26(17,39)	88(73,106)	163(144,184)	high_depr
Cancers	Primary Malignancy_colorectal and anus	pri_bowel	26(17,38)	84(69,101)	217(194,241)	most_depr
Cancers	Primary Malignancy_Kidney and Ureter	pri_kidney	2(0,9)	28(19,39)	43(34,53)	least_depr
Cancers	Primary Malignancy_Kidney and Ureter	pri_kidney	4(1,13)	21(14,31)	50(40,61)	low_depr

Cancers	Primary Malignancy_Kidney and Ureter	pri_kidney	14(6,25)	21(14,31)	56(46,68)	medium
Cancers	Primary Malignancy_Kidney and Ureter	pri_kidney	13(7,24)	20(13,29)	41(32,52)	high_depr
Cancers	Primary Malignancy_Kidney and Ureter	pri_kidney	10(5,18)	31(22,43)	37(28,48)	most_depr
Cancers	Primary Malignancy_Liver	pri_liver	0(NaN,NaN)	2(0,7)	7(4,12)	least_depr
Cancers	Primary Malignancy_Liver	pri_liver	0(NaN,NaN)	3(1,8)	6(3,10)	low_depr
Cancers	Primary Malignancy_Liver	pri_liver	1(0,6)	7(3,13)	9(5,14)	medium
Cancers	Primary Malignancy_Liver	pri_liver	0(NaN,NaN)	6(2,12)	12(7,19)	high_depr
Cancers	Primary Malignancy_Liver	pri_liver	3(1,8)	6(3,12)	9(5,15)	most_depr
Cancers	Primary Malignancy_Lung and trachea	pri_lung	5(1,14)	21(14,30)	46(37,57)	least_depr
Cancers	Primary Malignancy_Lung and trachea	pri_lung	6(2,15)	20(13,29)	40(31,50)	low_depr
Cancers	Primary Malignancy_Lung and trachea	pri_lung	8(3,17)	26(18,37)	60(50,73)	medium
Cancers	Primary Malignancy_Lung and trachea	pri_lung	15(8,25)	29(20,40)	78(66,93)	high_depr
Cancers	Primary Malignancy_Lung and trachea	pri_lung	8(3,15)	63(50,78)	124(107,142)	most_depr
Cancers	Primary Malignancy_Lymph Nodes	pri_LN	0(NaN,NaN)	0(NaN,NaN)	2(0,5)	least_depr
Cancers	Primary Malignancy_Lymph Nodes	pri_LN	0(NaN,NaN)	1(0,4)	2(0,4)	low_depr
Cancers	Primary Malignancy_Lymph Nodes	pri_LN	1(0,6)	1(0,5)	2(0,5)	medium
Cancers	Primary Malignancy_Lymph Nodes	pri_LN	2(0,8)	0(NaN,NaN)	2(0,5)	high_depr
Cancers	Primary Malignancy_Lymph Nodes	pri_LN	0(NaN,NaN)	1(0,4)	5(2,10)	most_depr

Cancers	Primary Malignancy_Malignant Melanoma	pri_melanoma	83(64,106)	104(88,123)	146(129,164)	least_depr
Cancers	Primary Malignancy_Malignant Melanoma	pri_melanoma	81(61,104)	111(93,131)	151(134,169)	low_depr
Cancers	Primary Malignancy_Malignant Melanoma	pri_melanoma	65(48,85)	87(72,104)	133(117,150)	medium
Cancers	Primary Malignancy_Malignant Melanoma	pri_melanoma	66(50,85)	86(71,104)	111(96,127)	high_depr
Cancers	Primary Malignancy_Malignant Melanoma	pri_melanoma	44(32,58)	63(51,78)	97(83,114)	most_depr
Cancers	Primary Malignancy_Mesothelioma	pri_mesothelioma	1(0,7)	2(0,6)	7(4,12)	least_depr
Cancers	Primary Malignancy_Mesothelioma	pri_mesothelioma	0(NaN,NaN)	0(NaN,NaN)	5(2,9)	low_depr
Cancers	Primary Malignancy_Mesothelioma	pri_mesothelioma	0(NaN,NaN)	4(1,9)	8(5,14)	medium
Cancers	Primary Malignancy_Mesothelioma	pri_mesothelioma	0(NaN,NaN)	0(NaN,NaN)	6(3,11)	high_depr
Cancers	Primary Malignancy_Mesothelioma	pri_mesothelioma	1(0,5)	1(0,4)	8(4,14)	most_depr
Cancers	Primary Malignancy_Multiple independent sites	pri_multindep	1(0,7)	1(0,4)	1(0,4)	least_depr
Cancers	Primary Malignancy_Multiple independent sites	pri_multindep	3(0,9)	1(0,4)	6(3,10)	low_depr
Cancers	Primary Malignancy_Multiple independent sites	pri_multindep	0(NaN,NaN)	1(0,4)	4(2,8)	medium
Cancers	Primary Malignancy_Multiple independent sites	pri_multindep	0(NaN,NaN)	1(0,4)	2(0,5)	high_depr
Cancers	Primary Malignancy_Multiple independent sites	pri_multindep	1(0,5)	2(0,7)	5(2,10)	most_depr
Cancers	Primary Malignancy_Oesophageal	pri_oesoph	3(0,10)	5(2,10)	24(17,32)	least_depr
Cancers	Primary Malignancy_Oesophageal	pri_oesoph	2(0,9)	11(6,18)	24(18,33)	low_depr

Cancers	Primary Malignancy_Oesophageal	pri_oesoph	0(NaN,NaN)	15(9,24)	19(13,27)	medium
Cancers	Primary Malignancy_Oesophageal	pri_oesoph	2(0,7)	5(2,10)	27(20,37)	high_depr
Cancers	Primary Malignancy_Oesophageal	pri_oesoph	3(1,9)	16(10,25)	29(21,39)	most_depr
Cancers	Primary Malignancy_Oro-pharyngeal	pri_oroph	13(6,24)	16(10,25)	27(20,36)	least_depr
Cancers	Primary Malignancy_Oro-pharyngeal	pri_oroph	14(6,26)	22(14,32)	34(26,43)	low_depr
Cancers	Primary Malignancy_Oro-pharyngeal	pri_oroph	14(7,26)	26(18,36)	33(26,43)	medium
Cancers	Primary Malignancy_Oro-pharyngeal	pri_oroph	12(6,22)	27(19,38)	34(26,44)	high_depr
Cancers	Primary Malignancy_Oro-pharyngeal	pri_oroph	16(9,25)	38(28,50)	48(37,60)	most_depr
Cancers	Primary Malignancy_Other Organs	pri_other	30(18,45)	81(66,98)	123(108,140)	least_depr
Cancers	Primary Malignancy_Other Organs	pri_other	42(28,60)	86(71,104)	133(117,150)	low_depr
Cancers	Primary Malignancy_Other Organs	pri_other	35(23,51)	85(70,103)	134(118,152)	medium
Cancers	Primary Malignancy_Other Organs	pri_other	38(27,54)	83(68,100)	146(128,165)	high_depr
Cancers	Primary Malignancy_Other Organs	pri_other	38(27,53)	87(72,104)	187(166,210)	most_depr
Cancers	Primary Malignancy_Other Skin and subcutaneous tissue	pri_skin	185(155,219)	393(359,428)	881(839,924)	least_depr
Cancers	Primary Malignancy_Other Skin and subcutaneous tissue	pri_skin	190(159,225)	387(354,423)	794(755,835)	low_depr
Cancers	Primary Malignancy_Other Skin and subcutaneous tissue	pri_skin	153(126,183)	364(332,398)	791(751,833)	medium

Cancers	Primary Malignancy_Other Skin and subcutaneous tissue	pri_skin	175(149,205)	311(281,343)	689(650,730)	high_depr
Cancers	Primary Malignancy_Other Skin and subcutaneous tissue	pri_skin	125(105,149)	267(240,296)	594(556,633)	most_depr
Cancers	Primary Malignancy_Ovarian	pri_ovarian	19(11,31)	22(15,31)	43(34,52)	least_depr
Cancers	Primary Malignancy_Ovarian	pri_ovarian	14(7,25)	28(20,38)	45(37,55)	low_depr
Cancers	Primary Malignancy_Ovarian	pri_ovarian	15(8,26)	33(24,43)	45(36,55)	medium
Cancers	Primary Malignancy_Ovarian	pri_ovarian	14(8,24)	29(21,39)	37(29,47)	high_depr
Cancers	Primary Malignancy_Ovarian	pri_ovarian	18(11,27)	23(16,32)	40(31,50)	most_depr
Cancers	Primary Malignancy_Pancreatic	pri_pancr	3(0,9)	4(2,10)	20(14,27)	least_depr
Cancers	Primary Malignancy_Pancreatic	pri_pancr	0(NaN,NaN)	4(1,8)	18(13,25)	low_depr
Cancers	Primary Malignancy_Pancreatic	pri_pancr	1(0,8)	11(6,19)	14(9,21)	medium
Cancers	Primary Malignancy_Pancreatic	pri_pancr	6(2,14)	9(4,16)	20(14,28)	high_depr
Cancers	Primary Malignancy_Pancreatic	pri_pancr	4(1,10)	10(5,17)	28(20,37)	most_depr
Cancers	Primary Malignancy_Prostate	pri_prost	9(3,20)	110(91,131)	310(285,336)	least_depr
Cancers	Primary Malignancy_Prostate	pri_prost	5(1,14)	81(65,100)	327(301,354)	low_depr
Cancers	Primary Malignancy_Prostate	pri_prost	12(5,23)	87(70,106)	342(315,371)	medium
Cancers	Primary Malignancy_Prostate	pri_prost	10(4,20)	99(81,118)	299(273,328)	high_depr
Cancers	Primary Malignancy_Prostate	pri_prost	9(4,17)	76(62,93)	302(275,331)	most_depr
Cancers	Primary Malignancy_Stomach	pri_stomach	6(2,14)	6(3,13)	19(13,26)	least_depr
Cancers	Primary Malignancy_Stomach	pri_stomach	3(0,9)	9(5,17)	26(19,34)	low_depr
Cancers	Primary Malignancy_Stomach	pri_stomach	1(0,8)	15(9,24)	14(9,20)	medium

Cancers	Primary Malignancy_Stomach	pri_stomach	0(NaN,NaN)	6(3,12)	23(17,32)	high_depr
Cancers	Primary Malignancy_Stomach	pri_stomach	5(2,11)	16(10,25)	25(18,34)	most_depr
Cancers	Primary Malignancy_Testicular	pri_testis	26(15,42)	22(14,32)	15(10,22)	least_depr
Cancers	Primary Malignancy_Testicular	pri_testis	30(18,47)	18(11,28)	9(5,15)	low_depr
Cancers	Primary Malignancy_Testicular	pri_testis	35(23,53)	18(11,27)	8(4,13)	medium
Cancers	Primary Malignancy_Testicular	pri_testis	25(15,38)	19(12,29)	7(4,13)	high_depr
Cancers	Primary Malignancy_Testicular	pri_testis	16(9,26)	15(9,24)	16(10,24)	most_depr
Cancers	Primary Malignancy_Thyroid	pri_thyroid	12(5,22)	17(11,25)	11(7,17)	least_depr
Cancers	Primary Malignancy_Thyroid	pri_thyroid	13(6,24)	14(9,22)	16(11,22)	low_depr
Cancers	Primary Malignancy_Thyroid	pri_thyroid	9(3,18)	14(8,21)	14(9,20)	medium
Cancers	Primary Malignancy_Thyroid	pri_thyroid	9(4,18)	15(9,23)	16(11,23)	high_depr
Cancers	Primary Malignancy_Thyroid	pri_thyroid	10(5,18)	15(10,23)	15(10,23)	most_depr
Cancers	Primary Malignancy_Uterine	pri_uterine	6(2,15)	22(15,31)	50(41,61)	least_depr
Cancers	Primary Malignancy_Uterine	pri_uterine	1(0,7)	31(23,41)	53(44,64)	low_depr
Cancers	Primary Malignancy_Uterine	pri_uterine	8(3,17)	24(17,34)	54(45,65)	medium
Cancers	Primary Malignancy_Uterine	pri_uterine	17(10,27)	26(19,36)	50(41,61)	high_depr
Cancers	Primary Malignancy_Uterine	pri_uterine	10(5,18)	21(14,30)	53(42,65)	most_depr
Cancers	Secondary Malignancy_Adrenal gland	sec_adrenal	0(NaN,NaN)	7(3,13)	6(3,11)	least_depr
Cancers	Secondary Malignancy_Adrenal gland	sec_adrenal	2(0,9)	4(1,10)	7(4,12)	low_depr
Cancers	Secondary Malignancy_Adrenal gland	sec_adrenal	0(NaN,NaN)	2(0,6)	7(4,12)	medium
Cancers	Secondary Malignancy_Adrenal gland	sec_adrenal	1(0,6)	2(0,5)	10(6,16)	high_depr
Cancers	Secondary Malignancy_Adrenal gland	sec_adrenal	1(0,6)	6(2,11)	12(7,19)	most_depr

Cancers	Secondary Malignancy_Bone	sec_bone	10(4,20)	36(27,48)	61(50,73)	least_depr
Cancers	Secondary Malignancy_Bone	sec_bone	9(4,20)	29(21,40)	54(44,65)	low_depr
Cancers	Secondary Malignancy_Bone	sec_bone	14(7,25)	30(21,41)	60(50,73)	medium
Cancers	Secondary Malignancy_Bone	sec_bone	22(13,33)	34(25,46)	76(63,90)	high_depr
Cancers	Secondary Malignancy_Bone	sec_bone	11(6,20)	35(25,46)	80(67,96)	most_depr
Cancers	Secondary Malignancy_Bowel	sec_bowel	0(NaN,NaN)	4(1,9)	7(4,12)	least_depr
Cancers	Secondary Malignancy_Bowel	sec_bowel	2(0,9)	4(1,8)	7(4,11)	low_depr
Cancers	Secondary Malignancy_Bowel	sec_bowel	1(0,6)	5(2,10)	6(3,11)	medium
Cancers	Secondary Malignancy_Bowel	sec_bowel	4(1,11)	3(1,8)	3(1,7)	high_depr
Cancers	Secondary Malignancy_Bowel	sec_bowel	2(0,7)	2(0,6)	7(3,12)	most_depr
Cancers	Secondary Malignancy_Brain, Other CNS and Intracranial	sec_brain	3(0,9)	12(7,19)	22(16,30)	least_depr
Cancers	Secondary Malignancy_Brain, Other CNS and Intracranial	sec_brain	9(4,20)	11(6,19)	16(11,22)	low_depr
Cancers	Secondary Malignancy_Brain, Other CNS and Intracranial	sec_brain	6(2,14)	9(5,16)	23(17,31)	medium
Cancers	Secondary Malignancy_Brain, Other CNS and Intracranial	sec_brain	10(5,19)	14(9,22)	22(16,30)	high_depr
Cancers	Secondary Malignancy_Brain, Other CNS and Intracranial	sec_brain	2(0,7)	18(12,27)	37(28,47)	most_depr
Cancers	Secondary malignancy_Liver and intrahepatic bile duct	sec_liver	12(6,24)	38(28,51)	74(62,87)	least_depr
Cancers	Secondary malignancy_Liver and intrahepatic bile duct	sec_liver	16(8,29)	34(25,46)	56(46,67)	low_depr
Cancers	Secondary malignancy_Liver and intrahepatic bile duct	sec_liver	11(5,22)	42(32,55)	65(54,77)	medium

Cancers	Secondary malignancy_Liver and intrahepatic bile duct	sec_liver	25(16,37)	32(23,44)	78(65,92)	high_depr
Cancers	Secondary malignancy_Liver and intrahepatic bile duct	sec_liver	16(9,26)	49(38,62)	88(74,103)	most_depr
Cancers	Secondary Malignancy_Lung	sec_lung	8(3,18)	28(19,38)	56(46,68)	least_depr
Cancers	Secondary Malignancy_Lung	sec_lung	20(11,34)	29(20,40)	55(45,67)	low_depr
Cancers	Secondary Malignancy_Lung	sec_lung	11(5,21)	26(18,36)	55(45,67)	medium
Cancers	Secondary Malignancy_Lung	sec_lung	14(8,24)	27(19,37)	55(45,68)	high_depr
Cancers	Secondary Malignancy_Lung	sec_lung	9(5,17)	34(25,46)	63(51,76)	most_depr
Cancers	Secondary Malignancy_Lymph Nodes	sec_LN	60(44,81)	108(92,127)	160(143,179)	least_depr
Cancers	Secondary Malignancy_Lymph Nodes	sec_LN	64(47,85)	99(83,117)	155(138,173)	low_depr
Cancers	Secondary Malignancy_Lymph Nodes	sec_LN	62(46,81)	114(96,133)	140(124,158)	medium
Cancers	Secondary Malignancy_Lymph Nodes	sec_LN	68(52,86)	113(96,132)	174(155,195)	high_depr
Cancers	Secondary Malignancy_Lymph Nodes	sec_LN	50(37,65)	110(94,129)	170(150,191)	most_depr
Cancers	Secondary Malignancy_Other organs	sec_other	20(11,33)	35(25,46)	65(54,78)	least_depr
Cancers	Secondary Malignancy_Other organs	sec_other	17(9,29)	33(24,44)	57(47,69)	low_depr
Cancers	Secondary Malignancy_Other organs	sec_other	19(10,31)	40(30,52)	58(48,70)	medium
Cancers	Secondary Malignancy_Other organs	sec_other	22(14,34)	30(22,41)	62(51,75)	high_depr
Cancers	Secondary Malignancy_Other organs	sec_other	14(8,23)	40(30,53)	76(63,90)	most_depr
Cancers	Secondary Malignancy_Pleura	sec_pleura	1(0,7)	8(4,14)	13(9,20)	least_depr
Cancers	Secondary Malignancy_Pleura	sec_pleura	3(0,9)	10(5,17)	17(11,23)	low_depr

Cancers	Secondary Malignancy_Pleura	sec_pleura	5(1,13)	9(4,15)	13(8,19)	medium
Cancers	Secondary Malignancy_Pleura	sec_pleura	4(1,11)	8(4,14)	12(7,18)	high_depr
Cancers	Secondary Malignancy_Pleura	sec_pleura	2(0,7)	12(7,20)	19(13,28)	most_depr
Cancers	Secondary Malignancy_retroperitoneum and peritoneum	sec_peritoneum	15(8,27)	16(10,25)	45(36,55)	least_depr
Cancers	Secondary Malignancy_retroperitoneum and peritoneum	sec_peritoneum	7(2,17)	19(12,27)	42(34,52)	low_depr
Cancers	Secondary Malignancy_retroperitoneum and peritoneum	sec_peritoneum	5(1,13)	24(17,34)	29(22,38)	medium
Cancers	Secondary Malignancy_retroperitoneum and peritoneum	sec_peritoneum	14(8,24)	20(13,29)	31(23,40)	high_depr
Cancers	Secondary Malignancy_retroperitoneum and peritoneum	sec_peritoneum	6(2,12)	24(17,34)	35(27,46)	most_depr
Cardiovascular	Abdominal aortic aneurysm	AAA	3(0,11)	4(1,9)	38(30,48)	least_depr
Cardiovascular	Abdominal aortic aneurysm	AAA	3(0,10)	9(4,16)	50(40,61)	low_depr
Cardiovascular	Abdominal aortic aneurysm	AAA	6(2,15)	13(8,22)	40(31,50)	medium
Cardiovascular	Abdominal aortic aneurysm	AAA	4(1,11)	9(5,17)	44(35,56)	high_depr
Cardiovascular	Abdominal aortic aneurysm	AAA	1(0,5)	17(10,26)	52(41,65)	most_depr
Cardiovascular	Atrial fibrillation	AF	101(79,129)	202(177,229)	627(591,663)	least_depr
Cardiovascular	Atrial fibrillation	AF	76(57,100)	234(207,263)	643(608,680)	low_depr
Cardiovascular	Atrial fibrillation	AF	84(64,108)	229(203,258)	632(596,670)	medium
Cardiovascular	Atrial fibrillation	AF	82(64,104)	249(222,278)	650(612,690)	high_depr
Cardiovascular	Atrial fibrillation	AF	87(70,108)	277(250,308)	797(753,843)	most_depr
Cardiovascular	Atrioventricular block, complete	av_block_3	3(0,9)	5(2,11)	20(15,28)	least_depr
Cardiovascular	Atrioventricular block, complete	av_block_3	0(NaN,NaN)	5(2,11)	24(18,32)	low_depr
Cardiovascular	Atrioventricular block, complete	av_block_3	5(1,13)	6(3,13)	26(19,35)	medium
Cardiovascular	Atrioventricular block, complete	av_block_3	7(2,15)	5(2,11)	22(15,30)	high_depr

Cardiovascular	Atrioventricular block, complete	av_block_3	5(2,11)	7(3,13)	24(16,33)	most_depr
Cardiovascular	Atrioventricular block, first degree	av_block_1	12(5,24)	9(5,17)	37(29,46)	least_depr
Cardiovascular	Atrioventricular block, first degree	av_block_1	5(1,14)	21(14,31)	50(40,61)	low_depr
Cardiovascular	Atrioventricular block, first degree	av_block_1	8(3,18)	30(21,41)	38(29,48)	medium
Cardiovascular	Atrioventricular block, first degree	av_block_1	10(5,20)	16(10,25)	54(44,67)	high_depr
Cardiovascular	Atrioventricular block, first degree	av_block_1	10(5,18)	27(19,37)	65(53,79)	most_depr
Cardiovascular	Atrioventricular block, second degree	av_block_2	4(1,13)	6(2,12)	17(12,24)	least_depr
Cardiovascular	Atrioventricular block, second degree	av_block_2	8(2,18)	7(3,14)	15(10,22)	low_depr
Cardiovascular	Atrioventricular block, second degree	av_block_2	0(NaN,NaN)	11(6,19)	22(15,29)	medium
Cardiovascular	Atrioventricular block, second degree	av_block_2	6(2,13)	7(3,14)	15(10,22)	high_depr
Cardiovascular	Atrioventricular block, second degree	av_block_2	7(3,14)	9(5,16)	18(12,26)	most_depr
Cardiovascular	Bifascicular block	bifasc_block	0(NaN,NaN)	1(0,5)	3(1,7)	least_depr
Cardiovascular	Bifascicular block	bifasc_block	0(NaN,NaN)	0(NaN,NaN)	4(2,8)	low_depr
Cardiovascular	Bifascicular block	bifasc_block	1(0,6)	2(0,6)	3(1,7)	medium
Cardiovascular	Bifascicular block	bifasc_block	1(0,7)	2(0,5)	2(0,5)	high_depr
Cardiovascular	Bifascicular block	bifasc_block	0(NaN,NaN)	2(0,6)	3(1,8)	most_depr
Cardiovascular	Coronary heart disease not otherwise specified	CHD_NOS	123(98,153)	388(353,425)	1005(961,1052)	least_depr
Cardiovascular	Coronary heart disease not otherwise specified	CHD_NOS	136(109,167)	445(408,484)	1096(1049,1144)	low_depr
Cardiovascular	Coronary heart disease not otherwise specified	CHD_NOS	147(120,178)	505(466,547)	1190(1140,1241)	medium
Cardiovascular	Coronary heart disease not otherwise specified	CHD_NOS	164(138,193)	548(507,590)	1281(1227,1337)	high_depr
Cardiovascular	Coronary heart disease not otherwise specified	CHD_NOS	299(266,334)	791(744,841)	1704(1640,1771)	most_depr
Cardiovascular	Dilated cardiomyopathy	dcm	6(2,15)	14(8,23)	22(15,30)	least_depr
Cardiovascular	Dilated cardiomyopathy	dcm	9(3,20)	10(5,18)	26(19,34)	low_depr
Cardiovascular	Dilated cardiomyopathy	dcm	5(1,13)	15(9,24)	30(22,39)	medium

Cardiovascular	Dilated cardiomyopathy	dcm	9(4,18)	24(16,34)	26(19,35)	high_depr
Cardiovascular	Dilated cardiomyopathy	dcm	19(11,29)	17(11,26)	30(22,40)	most_depr
Cardiovascular	Heart failure	hf	18(10,31)	76(61,93)	221(200,243)	least_depr
Cardiovascular	Heart failure	hf	29(17,45)	76(61,93)	275(251,299)	low_depr
Cardiovascular	Heart failure	hf	28(17,42)	100(83,120)	278(254,304)	medium
Cardiovascular	Heart failure	hf	43(30,59)	128(109,150)	295(270,323)	high_depr
Cardiovascular	Heart failure	hf	72(57,91)	171(149,195)	483(449,519)	most_depr
Cardiovascular	Hypertension	hypertension	1056(982,1135)	2125(2046,2208)	3809(3722,3897)	least_depr
Cardiovascular	Hypertension	hypertension	1069(993,1149)	2248(2165,2333)	3971(3883,4061)	low_depr
Cardiovascular	Hypertension	hypertension	1105(1031,1184)	2481(2395,2569)	4152(4060,4246)	medium
Cardiovascular	Hypertension	hypertension	1226(1154,1302)	2635(2547,2725)	4318(4220,4419)	high_depr
Cardiovascular	Hypertension	hypertension	1627(1550,1707)	3142(3048,3239)	4895(4786,5005)	most_depr
Cardiovascular	Hypertrophic Cardiomyopathy	hocm	4(1,13)	8(4,15)	8(4,12)	least_depr
Cardiovascular	Hypertrophic Cardiomyopathy	hocm	2(0,9)	7(3,13)	12(7,18)	low_depr
Cardiovascular	Hypertrophic Cardiomyopathy	hocm	7(2,17)	5(2,11)	8(4,13)	medium
Cardiovascular	Hypertrophic Cardiomyopathy	hocm	6(2,13)	10(5,17)	8(5,14)	high_depr
Cardiovascular	Hypertrophic Cardiomyopathy	hocm	9(4,17)	16(9,24)	11(6,17)	most_depr
Cardiovascular	Intracerebral haemorrhage	Intracereb_haem	2(0,9)	15(9,23)	32(24,41)	least_depr
Cardiovascular	Intracerebral haemorrhage	Intracereb_haem	5(1,14)	11(6,19)	26(19,35)	low_depr
Cardiovascular	Intracerebral haemorrhage	Intracereb_haem	8(3,18)	26(18,36)	38(30,48)	medium
Cardiovascular	Intracerebral haemorrhage	Intracereb_haem	12(6,22)	25(17,36)	40(31,51)	high_depr
Cardiovascular	Intracerebral haemorrhage	Intracereb_haem	14(7,23)	30(22,42)	42(32,53)	most_depr
Cardiovascular	Ischaemic stroke	Isch_stroke	18(9,31)	52(40,66)	112(98,128)	least_depr
Cardiovascular	Ischaemic stroke	Isch_stroke	23(13,38)	54(42,69)	133(117,151)	low_depr
Cardiovascular	Ischaemic stroke	Isch_stroke	17(9,29)	67(54,83)	141(124,159)	medium
Cardiovascular	Ischaemic stroke	Isch_stroke	43(30,59)	62(49,78)	145(127,164)	high_depr
Cardiovascular	Ischaemic stroke	Isch_stroke	51(38,67)	123(105,144)	233(210,258)	most_depr
Cardiovascular	Left bundle branch block	LBBB	13(6,25)	28(20,39)	57(47,69)	least_depr

Cardiovascular	Left bundle branch block	LBBB	9(3,19)	25(17,36)	66(55,79)	low_depr
Cardiovascular	Left bundle branch block	LBBB	3(0,10)	29(21,40)	79(67,93)	medium
Cardiovascular	Left bundle branch block	LBBB	17(9,28)	28(20,39)	66(54,80)	high_depr
Cardiovascular	Left bundle branch block	LBBB	18(11,28)	26(18,36)	92(78,109)	most_depr
Cardiovascular	Multiple valve dz	mult_valve	6(2,15)	14(8,23)	59(48,71)	least_depr
Cardiovascular	Multiple valve dz	mult_valve	3(0,10)	17(11,26)	60(50,72)	low_depr
Cardiovascular	Multiple valve dz	mult_valve	8(3,17)	22(14,32)	51(41,62)	medium
Cardiovascular	Multiple valve dz	mult_valve	10(5,19)	30(21,41)	55(44,67)	high_depr
Cardiovascular	Multiple valve dz	mult_valve	16(9,26)	33(24,44)	72(59,86)	most_depr
Cardiovascular	Myocardial infarction	myocardial_infarction	83(63,107)	239(212,268)	538(505,572)	least_depr
Cardiovascular	Myocardial infarction	myocardial_infarction	100(78,128)	242(214,271)	597(562,632)	low_depr
Cardiovascular	Myocardial infarction	myocardial_infarction	97(75,123)	308(277,341)	626(590,664)	medium
Cardiovascular	Myocardial infarction	myocardial_infarction	122(100,148)	305(275,338)	709(669,751)	high_depr
Cardiovascular	Myocardial infarction	myocardial_infarction	224(196,255)	499(461,539)	963(914,1013)	most_depr
Cardiovascular	Nonrheumatic aortic valve disorders	nonRh_aortic	19(10,33)	36(26,48)	124(109,141)	least_depr
Cardiovascular	Nonrheumatic aortic valve disorders	nonRh_aortic	24(14,38)	38(28,51)	152(135,171)	low_depr
Cardiovascular	Nonrheumatic aortic valve disorders	nonRh_aortic	31(19,46)	49(37,63)	119(104,136)	medium
Cardiovascular	Nonrheumatic aortic valve disorders	nonRh_aortic	20(12,32)	60(47,76)	125(108,143)	high_depr
Cardiovascular	Nonrheumatic aortic valve disorders	nonRh_aortic	24(16,36)	69(55,84)	189(168,212)	most_depr
Cardiovascular	Nonrheumatic mitral valve disorders	nonRh_mitral	24(14,38)	49(38,63)	113(98,129)	least_depr
Cardiovascular	Nonrheumatic mitral valve disorders	nonRh_mitral	16(8,29)	48(37,61)	116(101,132)	low_depr
Cardiovascular	Nonrheumatic mitral valve disorders	nonRh_mitral	45(32,63)	57(45,72)	106(92,122)	medium
Cardiovascular	Nonrheumatic mitral valve disorders	nonRh_mitral	34(23,48)	69(56,85)	109(94,126)	high_depr
Cardiovascular	Nonrheumatic mitral valve disorders	nonRh_mitral	30(20,42)	67(54,82)	130(113,149)	most_depr
Cardiovascular	Other Cardiomyopathy	cardiomyo_oth	10(4,21)	20(13,30)	34(26,43)	least_depr
Cardiovascular	Other Cardiomyopathy	cardiomyo_oth	11(5,22)	15(9,23)	42(33,53)	low_depr

Cardiovascular	Other Cardiomyopathy	cardiomyo_oth	7(3,16)	24(16,34)	48(39,60)	medium
Cardiovascular	Other Cardiomyopathy	cardiomyo_oth	17(9,28)	31(22,42)	46(36,57)	high_depr
Cardiovascular	Other Cardiomyopathy	cardiomyo_oth	27(18,40)	37(28,49)	51(40,63)	most_depr
Cardiovascular	Pericardial effusion (noninflammatory)	pericardial_effusion	16(8,28)	17(11,26)	43(34,53)	least_depr
Cardiovascular	Pericardial effusion (noninflammatory)	pericardial_effusion	6(2,15)	28(19,39)	41(32,51)	low_depr
Cardiovascular	Pericardial effusion (noninflammatory)	pericardial_effusion	26(16,41)	26(18,37)	47(38,58)	medium
Cardiovascular	Pericardial effusion (noninflammatory)	pericardial_effusion	15(8,26)	25(17,36)	30(23,40)	high_depr
Cardiovascular	Pericardial effusion (noninflammatory)	pericardial_effusion	24(16,36)	27(19,38)	46(36,58)	most_depr
Cardiovascular	Peripheral arterial disease	peripheral_arterial_disease	18(10,31)	47(36,61)	135(119,152)	least_depr
Cardiovascular	Peripheral arterial disease	peripheral_arterial_disease	19(10,32)	53(41,68)	144(127,162)	low_depr
Cardiovascular	Peripheral arterial disease	peripheral_arterial_disease	26(15,40)	56(43,71)	176(157,196)	medium
Cardiovascular	Peripheral arterial disease	peripheral_arterial_disease	27(17,40)	86(70,104)	210(189,234)	high_depr
Cardiovascular	Peripheral arterial disease	peripheral_arterial_disease	60(46,77)	153(133,176)	383(353,416)	most_depr
Cardiovascular	Primary pulmonary hypertension	prim_pulm_htn	0(NaN,NaN)	4(1,9)	14(9,20)	least_depr
Cardiovascular	Primary pulmonary hypertension	prim_pulm_htn	4(1,12)	8(4,15)	19(13,26)	low_depr
Cardiovascular	Primary pulmonary hypertension	prim_pulm_htn	8(3,17)	2(0,7)	16(11,23)	medium
Cardiovascular	Primary pulmonary hypertension	prim_pulm_htn	3(1,10)	6(2,12)	16(10,23)	high_depr
Cardiovascular	Primary pulmonary hypertension	prim_pulm_htn	10(5,18)	13(7,20)	30(22,40)	most_depr
Cardiovascular	Pulmonary embolism	PE	34(22,51)	67(53,83)	128(112,145)	least_depr
Cardiovascular	Pulmonary embolism	PE	38(25,56)	79(64,96)	131(116,148)	low_depr
Cardiovascular	Pulmonary embolism	PE	33(21,49)	89(73,107)	152(135,171)	medium
Cardiovascular	Pulmonary embolism	PE	44(31,60)	93(77,111)	133(116,151)	high_depr
Cardiovascular	Pulmonary embolism	PE	69(54,87)	105(89,124)	185(165,208)	most_depr
Cardiovascular	Raynaud's syndrome	raynauds	157(130,188)	136(118,158)	152(135,170)	least_depr
Cardiovascular	Raynaud's syndrome	raynauds	135(110,165)	139(119,160)	145(129,163)	low_depr
Cardiovascular	Raynaud's syndrome	raynauds	147(122,176)	139(120,160)	152(135,171)	medium
Cardiovascular	Raynaud's syndrome	raynauds	137(115,163)	130(111,150)	159(141,178)	high_depr
Cardiovascular	Raynaud's syndrome	raynauds	116(96,138)	138(119,159)	164(145,185)	most_depr

Cardiovascular	Rheumatic valve dz	Rh_valve	5(1,14)	12(7,19)	28(21,36)	least_depr
Cardiovascular	Rheumatic valve dz	Rh_valve	0(NaN,NaN)	10(5,17)	30(23,39)	low_depr
Cardiovascular	Rheumatic valve dz	Rh_valve	5(1,14)	10(5,17)	29(22,38)	medium
Cardiovascular	Rheumatic valve dz	Rh_valve	5(2,13)	12(7,19)	29(21,38)	high_depr
Cardiovascular	Rheumatic valve dz	Rh_valve	9(4,16)	25(17,34)	47(37,59)	most_depr
Cardiovascular	Right bundle branch block	RBBB	26(15,41)	33(24,45)	70(58,83)	least_depr
Cardiovascular	Right bundle branch block	RBBB	24(13,39)	37(27,50)	76(64,90)	low_depr
Cardiovascular	Right bundle branch block	RBBB	26(16,41)	41(30,54)	88(75,102)	medium
Cardiovascular	Right bundle branch block	RBBB	35(23,50)	36(26,49)	81(68,96)	high_depr
Cardiovascular	Right bundle branch block	RBBB	19(11,29)	45(34,58)	102(86,119)	most_depr
Cardiovascular	Secondary pulmonary hypertension	sec_pulm_htn	2(0,9)	4(1,8)	6(3,11)	least_depr
Cardiovascular	Secondary pulmonary hypertension	sec_pulm_htn	2(0,9)	6(3,12)	13(8,19)	low_depr
Cardiovascular	Secondary pulmonary hypertension	sec_pulm_htn	5(1,14)	2(0,6)	13(8,19)	medium
Cardiovascular	Secondary pulmonary hypertension	sec_pulm_htn	5(2,13)	7(3,14)	12(7,19)	high_depr
Cardiovascular	Secondary pulmonary hypertension	sec_pulm_htn	5(2,11)	5(2,10)	20(14,29)	most_depr
Cardiovascular	Sick sinus syndrome	sick_sinus	7(2,15)	9(4,15)	21(15,29)	least_depr
Cardiovascular	Sick sinus syndrome	sick_sinus	3(0,10)	9(4,16)	18(13,25)	low_depr
Cardiovascular	Sick sinus syndrome	sick_sinus	4(1,11)	7(3,13)	16(11,23)	medium
Cardiovascular	Sick sinus syndrome	sick_sinus	1(0,7)	6(3,12)	21(14,29)	high_depr
Cardiovascular	Sick sinus syndrome	sick_sinus	2(0,7)	8(4,15)	24(17,33)	most_depr
Cardiovascular	Stable angina	stable_angina	83(63,107)	285(255,316)	764(725,805)	least_depr
Cardiovascular	Stable angina	stable_angina	101(78,128)	320(289,354)	821(781,863)	low_depr
Cardiovascular	Stable angina	stable_angina	90(70,115)	358(325,393)	887(844,931)	medium
Cardiovascular	Stable angina	stable_angina	121(98,146)	391(357,427)	982(935,1031)	high_depr
Cardiovascular	Stable angina	stable_angina	207(180,236)	612(571,656)	1305(1249,1363)	most_depr
Cardiovascular	Stroke NOS	Stroke_NOS	39(26,57)	91(75,110)	243(221,266)	least_depr
Cardiovascular	Stroke NOS	Stroke_NOS	44(30,63)	120(101,141)	272(249,296)	low_depr
Cardiovascular	Stroke NOS	Stroke_NOS	48(33,66)	130(110,151)	295(270,321)	medium
Cardiovascular	Stroke NOS	Stroke_NOS	65(49,84)	139(119,161)	302(276,329)	high_depr
Cardiovascular	Stroke NOS	Stroke_NOS	110(91,133)	242(216,270)	472(438,507)	most_depr
Cardiovascular	Subarachnoid haemorrhage	Subarach	17(9,29)	27(19,37)	30(22,38)	least_depr
Cardiovascular	Subarachnoid haemorrhage	Subarach	20(11,34)	28(20,39)	43(34,53)	low_depr

Cardiovascular	Subarachnoid haemorrhage	Subarach	18(10,30)	36(26,48)	42(33,52)	medium
Cardiovascular	Subarachnoid haemorrhage	Subarach	21(12,33)	35(25,46)	50(40,61)	high_depr
Cardiovascular	Subarachnoid haemorrhage	Subarach	28(19,40)	50(39,63)	49(39,62)	most_depr
Cardiovascular	Subdural haematoma - nontraumatic	subdural_haem	2(0,9)	5(2,11)	11(7,17)	least_depr
Cardiovascular	Subdural haematoma - nontraumatic	subdural_haem	2(0,9)	2(0,7)	11(7,17)	low_depr
Cardiovascular	Subdural haematoma - nontraumatic	subdural_haem	1(0,8)	5(2,11)	7(4,12)	medium
Cardiovascular	Subdural haematoma - nontraumatic	subdural_haem	7(3,16)	6(3,12)	9(5,15)	high_depr
Cardiovascular	Subdural haematoma - nontraumatic	subdural_haem	4(1,10)	6(3,12)	18(12,26)	most_depr
Cardiovascular	Supraventricular tachycardia	SVT	44(30,62)	64(51,80)	117(103,134)	least_depr
Cardiovascular	Supraventricular tachycardia	SVT	57(41,77)	89(74,107)	116(101,132)	low_depr
Cardiovascular	Supraventricular tachycardia	SVT	52(37,71)	57(45,71)	112(98,128)	medium
Cardiovascular	Supraventricular tachycardia	SVT	49(36,65)	79(65,96)	111(96,128)	high_depr
Cardiovascular	Supraventricular tachycardia	SVT	44(32,59)	77(63,94)	120(104,139)	most_depr
Cardiovascular	Transient ischaemic attack	TIA	20(11,33)	73(59,90)	205(185,226)	least_depr
Cardiovascular	Transient ischaemic attack	TIA	23(13,37)	85(70,103)	227(207,250)	low_depr
Cardiovascular	Transient ischaemic attack	TIA	27(16,41)	82(67,100)	255(233,279)	medium
Cardiovascular	Transient ischaemic attack	TIA	31(21,46)	109(92,129)	230(208,254)	high_depr
Cardiovascular	Transient ischaemic attack	TIA	52(39,68)	128(110,149)	324(296,354)	most_depr
Cardiovascular	Trifascicular block	trifasc_block	0(NaN,NaN)	1(0,4)	4(2,8)	least_depr
Cardiovascular	Trifascicular block	trifasc_block	1(0,7)	1(0,5)	3(1,7)	low_depr
Cardiovascular	Trifascicular block	trifasc_block	0(NaN,NaN)	1(0,5)	3(1,7)	medium
Cardiovascular	Trifascicular block	trifasc_block	0(NaN,NaN)	0(NaN,NaN)	3(1,7)	high_depr
Cardiovascular	Trifascicular block	trifasc_block	0(NaN,NaN)	1(0,5)	7(3,12)	most_depr
Cardiovascular	Unstable Angina	unstable_angin a	37(24,54)	65(51,81)	180(161,200)	least_depr
Cardiovascular	Unstable Angina	unstable_angin a	34(21,51)	85(69,104)	206(186,228)	low_depr
Cardiovascular	Unstable Angina	unstable_angin a	14(6,25)	120(101,141)	220(199,242)	medium

Cardiovascular	Unstable Angina	unstable_angina	33(22,48)	111(94,131)	267(242,293)	high_depr
Cardiovascular	Unstable Angina	unstable_angina	78(62,97)	203(179,229)	380(350,413)	most_depr
Cardiovascular	Venous thromboembolic disease (Excl PE)	vte_ex_pe	66(49,88)	123(104,144)	222(201,244)	least_depr
Cardiovascular	Venous thromboembolic disease (Excl PE)	vte_ex_pe	87(67,111)	127(108,149)	215(195,237)	low_depr
Cardiovascular	Venous thromboembolic disease (Excl PE)	vte_ex_pe	85(65,108)	156(135,179)	225(204,248)	medium
Cardiovascular	Venous thromboembolic disease (Excl PE)	vte_ex_pe	83(65,104)	164(143,188)	227(205,251)	high_depr
Cardiovascular	Venous thromboembolic disease (Excl PE)	vte_ex_pe	129(108,153)	160(139,183)	284(259,312)	most_depr
Cardiovascular	Ventricular tachycardia	VT	7(2,17)	14(8,22)	24(17,32)	least_depr
Cardiovascular	Ventricular tachycardia	VT	9(3,20)	16(9,24)	35(27,44)	low_depr
Cardiovascular	Ventricular tachycardia	VT	14(7,26)	18(11,27)	28(21,37)	medium
Cardiovascular	Ventricular tachycardia	VT	11(5,21)	14(8,23)	40(31,51)	high_depr
Cardiovascular	Ventricular tachycardia	VT	13(7,22)	22(15,32)	39(30,51)	most_depr
Digestive	Abdominal Hernia	hernia_abdo	610(553,672)	821(770,874)	1168(1119,1218)	least_depr
Digestive	Abdominal Hernia	hernia_abdo	619(560,682)	768(718,820)	1187(1138,1238)	low_depr
Digestive	Abdominal Hernia	hernia_abdo	629(572,690)	800(749,852)	1139(1089,1189)	medium
Digestive	Abdominal Hernia	hernia_abdo	587(536,641)	873(821,927)	1197(1144,1251)	high_depr
Digestive	Abdominal Hernia	hernia_abdo	607(560,657)	804(756,855)	1240(1185,1297)	most_depr
Digestive	Alcoholic liver disease	liver_alc	14(6,26)	13(7,21)	25(18,33)	least_depr
Digestive	Alcoholic liver disease	liver_alc	5(1,14)	20(13,30)	20(14,28)	low_depr
Digestive	Alcoholic liver disease	liver_alc	18(10,31)	23(15,33)	33(25,43)	medium
Digestive	Alcoholic liver disease	liver_alc	16(9,28)	47(36,61)	39(30,50)	high_depr
Digestive	Alcoholic liver disease	liver_alc	43(32,58)	92(76,110)	70(57,85)	most_depr
Digestive	Anal fissure	anal_fissure	331(290,375)	272(244,302)	255(233,279)	least_depr
Digestive	Anal fissure	anal_fissure	361(319,408)	283(255,314)	264(241,287)	low_depr
Digestive	Anal fissure	anal_fissure	303(266,345)	309(280,341)	240(218,263)	medium
Digestive	Anal fissure	anal_fissure	331(295,371)	301(272,332)	246(223,271)	high_depr

Digestive	Anal fissure	anal_fissure	331(296,367)	304(275,335)	275(249,302)	most_depr
Digestive	Angiodysplasia of colon	angiodysplasia_colon	0(NaN,NaN)	8(4,15)	15(10,21)	least_depr
Digestive	Angiodysplasia of colon	angiodysplasia_colon	3(0,9)	8(4,15)	14(9,20)	low_depr
Digestive	Angiodysplasia of colon	angiodysplasia_colon	6(2,15)	4(1,10)	16(11,23)	medium
Digestive	Angiodysplasia of colon	angiodysplasia_colon	3(1,10)	9(5,16)	22(16,31)	high_depr
Digestive	Angiodysplasia of colon	angiodysplasia_colon	5(2,11)	8(4,15)	25(18,35)	most_depr
Digestive	Anorectal fistula	anorectal_fistula	76(57,99)	79(64,96)	61(50,73)	least_depr
Digestive	Anorectal fistula	anorectal_fistula	72(53,95)	69(56,86)	66(55,78)	low_depr
Digestive	Anorectal fistula	anorectal_fistula	62(45,83)	78(63,95)	68(57,81)	medium
Digestive	Anorectal fistula	anorectal_fistula	94(75,117)	85(70,103)	71(59,85)	high_depr
Digestive	Anorectal fistula	anorectal_fistula	73(57,91)	104(87,123)	82(69,98)	most_depr
Digestive	Anorectal prolapse	anorectal_prolapse	20(11,33)	32(23,43)	56(46,67)	least_depr
Digestive	Anorectal prolapse	anorectal_prolapse	18(10,31)	41(31,53)	53(44,64)	low_depr
Digestive	Anorectal prolapse	anorectal_prolapse	22(13,35)	30(22,41)	50(41,61)	medium
Digestive	Anorectal prolapse	anorectal_prolapse	16(9,27)	42(32,54)	62(51,75)	high_depr
Digestive	Anorectal prolapse	anorectal_prolapse	20(12,31)	43(33,55)	64(52,77)	most_depr
Digestive	Appendicitis	appendicitis	385(341,433)	403(369,439)	420(392,450)	least_depr
Digestive	Appendicitis	appendicitis	399(354,449)	406(372,443)	385(358,413)	low_depr
Digestive	Appendicitis	appendicitis	367(325,413)	401(367,437)	377(349,405)	medium
Digestive	Appendicitis	appendicitis	391(351,434)	381(349,416)	384(355,415)	high_depr
Digestive	Appendicitis	appendicitis	357(322,395)	413(379,449)	411(380,444)	most_depr
Digestive	Autoimmune liver disease	autoimm_liver	8(3,18)	15(9,23)	15(10,21)	least_depr
Digestive	Autoimmune liver disease	autoimm_liver	9(4,19)	9(5,16)	21(15,28)	low_depr
Digestive	Autoimmune liver disease	autoimm_liver	11(5,21)	11(6,18)	19(14,27)	medium
Digestive	Autoimmune liver disease	autoimm_liver	3(1,9)	16(10,24)	25(18,33)	high_depr
Digestive	Autoimmune liver disease	autoimm_liver	8(3,15)	18(12,26)	23(16,31)	most_depr
Digestive	Barrett's oesophagus	barretts	20(11,33)	89(73,108)	124(108,140)	least_depr

Digestive	Barrett's oesophagus	barretts	26(15,41)	68(54,84)	129(113,146)	low_depr
Digestive	Barrett's oesophagus	barretts	43(29,61)	81(66,98)	141(124,159)	medium
Digestive	Barrett's oesophagus	barretts	37(25,52)	82(67,99)	140(122,159)	high_depr
Digestive	Barrett's oesophagus	barretts	52(39,68)	95(79,114)	169(150,191)	most_depr
Digestive	Cholangitis	cholangitis	5(1,14)	4(1,10)	23(17,31)	least_depr
Digestive	Cholangitis	cholangitis	6(2,16)	13(7,21)	22(16,29)	low_depr
Digestive	Cholangitis	cholangitis	4(1,12)	17(10,25)	27(20,36)	medium
Digestive	Cholangitis	cholangitis	6(2,14)	27(18,37)	29(22,39)	high_depr
Digestive	Cholangitis	cholangitis	8(3,16)	21(14,30)	34(25,44)	most_depr
Digestive	Cholecystitis	cholecystitis	120(97,148)	190(168,215)	245(224,268)	least_depr
Digestive	Cholecystitis	cholecystitis	160(133,192)	198(175,223)	279(256,303)	low_depr
Digestive	Cholecystitis	cholecystitis	149(123,178)	198(175,223)	294(270,319)	medium
Digestive	Cholecystitis	cholecystitis	164(139,192)	225(201,252)	329(303,357)	high_depr
Digestive	Cholecystitis	cholecystitis	209(182,238)	234(209,261)	355(326,385)	most_depr
Digestive	Cholelithiasis	cholelithiasis	259(224,298)	353(322,386)	485(455,517)	least_depr
Digestive	Cholelithiasis	cholelithiasis	279(242,319)	358(327,391)	511(480,543)	low_depr
Digestive	Cholelithiasis	cholelithiasis	264(230,303)	386(354,420)	549(516,583)	medium
Digestive	Cholelithiasis	cholelithiasis	325(290,364)	418(385,454)	583(548,620)	high_depr
Digestive	Cholelithiasis	cholelithiasis	366(330,404)	494(457,532)	662(622,703)	most_depr
Digestive	Coeliac disease	coeliac	62(45,83)	74(60,90)	72(61,85)	least_depr
Digestive	Coeliac disease	coeliac	77(58,100)	69(56,85)	74(63,87)	low_depr
Digestive	Coeliac disease	coeliac	50(36,69)	66(53,81)	71(60,84)	medium
Digestive	Coeliac disease	coeliac	61(46,80)	79(65,96)	68(56,82)	high_depr
Digestive	Coeliac disease	coeliac	60(46,77)	66(53,81)	79(66,94)	most_depr
Digestive	Crohn's disease	crohns	37(24,54)	40(30,53)	48(38,58)	least_depr
Digestive	Crohn's disease	crohns	35(23,51)	54(42,69)	46(37,56)	low_depr
Digestive	Crohn's disease	crohns	44(30,62)	43(33,56)	47(37,57)	medium
Digestive	Crohn's disease	crohns	50(36,67)	53(41,67)	55(45,68)	high_depr
Digestive	Crohn's disease	crohns	69(54,86)	53(42,67)	63(51,76)	most_depr
Digestive	Diaphragmatic hernia	hernia_diaphragm	277(239,318)	488(451,528)	833(793,874)	least_depr
Digestive	Diaphragmatic hernia	hernia_diaphragm	235(201,273)	535(495,576)	861(821,903)	low_depr
Digestive	Diaphragmatic hernia	hernia_diaphragm	309(271,351)	545(506,587)	894(851,937)	medium
Digestive	Diaphragmatic hernia	hernia_diaphragm	338(301,378)	601(560,644)	962(916,1010)	high_depr
Digestive	Diaphragmatic hernia	hernia_diaphragm	423(384,464)	772(726,820)	1150(1098,1204)	most_depr
Digestive	Diverticular disease of intestine (acute and chronic)	diverticuli	174(145,207)	546(506,588)	1014(970,1060)	least_depr

Digestive	Diverticular disease of intestine (acute and chronic)	diverticuli	177(148,211)	562(521,605)	1079(1033,1126)	low_depr
Digestive	Diverticular disease of intestine (acute and chronic)	diverticuli	209(177,244)	533(493,574)	1075(1028,1123)	medium
Digestive	Diverticular disease of intestine (acute and chronic)	diverticuli	199(171,231)	583(543,626)	1180(1129,1233)	high_depr
Digestive	Diverticular disease of intestine (acute and chronic)	diverticuli	231(203,262)	609(568,652)	1326(1270,1384)	most_depr
Digestive	Fatty Liver	fatty_liver	72(53,95)	64(51,80)	77(65,90)	least_depr
Digestive	Fatty Liver	fatty_liver	69(51,92)	86(70,104)	100(87,115)	low_depr
Digestive	Fatty Liver	fatty_liver	71(53,94)	95(79,114)	95(82,110)	medium
Digestive	Fatty Liver	fatty_liver	91(72,114)	131(112,153)	121(105,139)	high_depr
Digestive	Fatty Liver	fatty_liver	116(96,139)	169(148,193)	151(132,171)	most_depr
Digestive	Gastritis and duodenitis	gastritis_duodenitis	607(551,667)	798(750,848)	1136(1089,1184)	least_depr
Digestive	Gastritis and duodenitis	gastritis_duodenitis	609(552,669)	807(758,858)	1197(1149,1246)	low_depr
Digestive	Gastritis and duodenitis	gastritis_duodenitis	703(644,766)	967(914,1023)	1289(1238,1341)	medium
Digestive	Gastritis and duodenitis	gastritis_duodenitis	733(678,792)	982(929,1037)	1352(1298,1409)	high_depr
Digestive	Gastritis and duodenitis	gastritis_duodenitis	928(870,988)	1337(1276,1401)	1629(1567,1693)	most_depr
Digestive	Gastro-oesophageal reflux disease	GORD	483(433,537)	774(727,824)	985(942,1031)	least_depr
Digestive	Gastro-oesophageal reflux disease	GORD	492(442,547)	742(696,791)	1016(972,1061)	low_depr
Digestive	Gastro-oesophageal reflux disease	GORD	560(508,616)	842(793,893)	1072(1026,1120)	medium
Digestive	Gastro-oesophageal reflux disease	GORD	608(558,662)	830(782,881)	1111(1062,1162)	high_depr
Digestive	Gastro-oesophageal reflux disease	GORD	720(669,774)	982(930,1037)	1199(1146,1254)	most_depr
Digestive	Hepatic failure	liver_fail	4(1,11)	6(2,11)	9(5,14)	least_depr
Digestive	Hepatic failure	liver_fail	4(1,12)	7(4,14)	12(8,18)	low_depr
Digestive	Hepatic failure	liver_fail	3(0,11)	10(5,18)	13(8,19)	medium
Digestive	Hepatic failure	liver_fail	7(3,15)	14(8,23)	24(17,33)	high_depr
Digestive	Hepatic failure	liver_fail	11(5,19)	20(13,30)	26(19,35)	most_depr
Digestive	Inflammatory bowel disease (IBD)	IBD	131(106,160)	144(124,167)	160(143,179)	least_depr
Digestive	Inflammatory bowel disease (IBD)	IBD	117(94,146)	157(136,180)	151(135,170)	low_depr

Digestive	Inflammatory bowel disease (IBD)	IBD	126(102,154)	144(124,166)	151(134,170)	medium
Digestive	Inflammatory bowel disease (IBD)	IBD	132(109,158)	160(140,183)	198(177,220)	high_depr
Digestive	Inflammatory bowel disease (IBD)	IBD	157(134,183)	148(129,171)	183(162,205)	most_depr
Digestive	Irritable bowel syndrome	IBS	345(304,390)	413(380,449)	376(350,404)	least_depr
Digestive	Irritable bowel syndrome	IBS	333(293,377)	359(328,392)	368(341,395)	low_depr
Digestive	Irritable bowel syndrome	IBS	351(311,395)	412(379,448)	344(318,371)	medium
Digestive	Irritable bowel syndrome	IBS	349(312,389)	399(366,433)	370(343,400)	high_depr
Digestive	Irritable bowel syndrome	IBS	411(373,451)	427(393,463)	391(361,423)	most_depr
Digestive	Liver fibrosis, sclerosis and cirrhosis	cirrhosis	19(10,32)	27(18,37)	49(39,60)	least_depr
Digestive	Liver fibrosis, sclerosis and cirrhosis	cirrhosis	21(11,34)	32(23,44)	44(35,54)	low_depr
Digestive	Liver fibrosis, sclerosis and cirrhosis	cirrhosis	28(17,43)	40(30,53)	54(44,66)	medium
Digestive	Liver fibrosis, sclerosis and cirrhosis	cirrhosis	26(17,40)	57(44,72)	70(57,84)	high_depr
Digestive	Liver fibrosis, sclerosis and cirrhosis	cirrhosis	44(32,58)	98(82,117)	102(87,120)	most_depr
Digestive	Oesophageal varices	varices	8(3,18)	10(6,18)	17(12,24)	least_depr
Digestive	Oesophageal varices	varices	8(3,18)	12(7,20)	15(10,21)	low_depr
Digestive	Oesophageal varices	varices	8(3,17)	19(12,29)	22(16,30)	medium
Digestive	Oesophageal varices	varices	17(10,28)	25(17,35)	29(22,39)	high_depr
Digestive	Oesophageal varices	varices	14(8,23)	28(20,39)	34(25,45)	most_depr
Digestive	Oesophagitis and oesophageal ulcer	oesoph_ulc	396(351,445)	617(575,662)	883(841,926)	least_depr
Digestive	Oesophagitis and oesophageal ulcer	oesoph_ulc	378(333,426)	658(614,704)	909(867,953)	low_depr
Digestive	Oesophagitis and oesophageal ulcer	oesoph_ulc	439(393,490)	697(651,744)	968(924,1014)	medium
Digestive	Oesophagitis and oesophageal ulcer	oesoph_ulc	447(403,493)	702(657,749)	1009(961,1058)	high_depr
Digestive	Oesophagitis and oesophageal ulcer	oesoph_ulc	549(505,597)	859(810,910)	1120(1069,1174)	most_depr
Digestive	Pancreatitis	pancreatitis	35(22,51)	45(34,59)	77(65,90)	least_depr
Digestive	Pancreatitis	pancreatitis	26(15,41)	51(39,65)	72(61,85)	low_depr
Digestive	Pancreatitis	pancreatitis	39(26,57)	44(33,57)	87(74,102)	medium
Digestive	Pancreatitis	pancreatitis	59(44,78)	64(51,79)	92(78,107)	high_depr
Digestive	Pancreatitis	pancreatitis	55(41,71)	111(93,130)	131(113,150)	most_depr

Digestive	Peptic ulcer disease	ulcer_peptic	114(90,142)	224(198,252)	373(346,401)	least_depr
Digestive	Peptic ulcer disease	ulcer_peptic	143(116,175)	234(207,262)	415(386,445)	low_depr
Digestive	Peptic ulcer disease	ulcer_peptic	146(119,176)	276(248,307)	436(406,467)	medium
Digestive	Peptic ulcer disease	ulcer_peptic	187(159,218)	309(279,341)	470(438,504)	high_depr
Digestive	Peptic ulcer disease	ulcer_peptic	241(212,273)	450(415,488)	684(643,726)	most_depr
Digestive	Peritonitis	peritonitis	38(25,55)	54(42,68)	70(59,83)	least_depr
Digestive	Peritonitis	peritonitis	41(27,59)	43(33,56)	82(70,96)	low_depr
Digestive	Peritonitis	peritonitis	38(25,55)	64(50,79)	70(59,84)	medium
Digestive	Peritonitis	peritonitis	44(31,60)	78(63,94)	89(75,104)	high_depr
Digestive	Peritonitis	peritonitis	66(51,84)	89(73,106)	108(92,126)	most_depr
Digestive	Portal hypertension	portal_htn	1(0,7)	6(3,12)	9(5,15)	least_depr
Digestive	Portal hypertension	portal_htn	3(0,10)	5(2,10)	11(7,17)	low_depr
Digestive	Portal hypertension	portal_htn	3(0,10)	11(6,18)	17(12,24)	medium
Digestive	Portal hypertension	portal_htn	8(3,16)	16(10,25)	20(14,28)	high_depr
Digestive	Portal hypertension	portal_htn	11(5,20)	29(21,40)	24(17,34)	most_depr
Digestive	Ulcerative colitis	ulc_colitis	102(80,128)	115(97,135)	125(110,142)	least_depr
Digestive	Ulcerative colitis	ulc_colitis	90(70,116)	118(99,138)	119(104,135)	low_depr
Digestive	Ulcerative colitis	ulc_colitis	94(74,119)	115(97,136)	123(107,140)	medium
Digestive	Ulcerative colitis	ulc_colitis	96(76,118)	120(102,140)	157(139,177)	high_depr
Digestive	Ulcerative colitis	ulc_colitis	108(89,130)	109(92,128)	141(123,161)	most_depr
Digestive	Volvulus	volvulus	13(6,25)	14(9,22)	17(12,24)	least_depr
Digestive	Volvulus	volvulus	7(2,15)	13(8,21)	15(10,22)	low_depr
Digestive	Volvulus	volvulus	10(4,20)	15(9,23)	25(18,33)	medium
Digestive	Volvulus	volvulus	11(5,20)	17(10,25)	20(13,27)	high_depr
Digestive	Volvulus	volvulus	6(2,13)	16(10,25)	29(21,39)	most_depr
Ear	Hearing loss	deaf	489(439,543)	755(708,804)	1276(1225,1327)	least_depr
Ear	Hearing loss	deaf	522(470,578)	791(742,842)	1333(1282,1385)	low_depr
Ear	Hearing loss	deaf	505(456,559)	763(716,812)	1343(1291,1397)	medium
Ear	Hearing loss	deaf	497(451,545)	802(754,852)	1341(1286,1397)	high_depr
Ear	Hearing loss	deaf	585(539,634)	900(850,953)	1430(1372,1491)	most_depr
Ear	Meniere disease	meniere	28(17,43)	52(41,65)	77(65,90)	least_depr
Ear	Meniere disease	meniere	30(19,45)	57(45,71)	80(68,94)	low_depr
Ear	Meniere disease	meniere	26(16,40)	56(45,70)	88(75,102)	medium
Ear	Meniere disease	meniere	26(17,39)	49(38,62)	83(70,97)	high_depr
Ear	Meniere disease	meniere	31(22,44)	56(44,70)	85(71,100)	most_depr
Ear	Tinnitus	tinnitus	313(273,357)	485(447,525)	528(496,562)	least_depr
Ear	Tinnitus	tinnitus	284(246,326)	455(418,494)	533(501,566)	low_depr

Ear	Tinnitus	tinnitus	293(256,335)	475(438,515)	560(527,595)	medium
Ear	Tinnitus	tinnitus	296(261,335)	454(419,493)	560(525,597)	high_depr
Ear	Tinnitus	tinnitus	304(271,339)	467(431,505)	539(503,577)	most_depr
Endocrine	Cystic Fibrosis	CF	3(0,9)	8(4,14)	2(1,5)	least_depr
Endocrine	Cystic Fibrosis	CF	7(2,15)	2(0,6)	2(1,6)	low_depr
Endocrine	Cystic Fibrosis	CF	8(3,17)	6(3,12)	3(1,6)	medium
Endocrine	Cystic Fibrosis	CF	3(1,9)	3(1,8)	3(1,7)	high_depr
Endocrine	Cystic Fibrosis	CF	4(1,9)	9(5,15)	7(3,12)	most_depr
Endocrine	Diabetes NOS	diabetes_nos	19(10,33)	34(25,46)	82(69,96)	least_depr
Endocrine	Diabetes NOS	diabetes_nos	27(16,43)	50(38,65)	86(73,100)	low_depr
Endocrine	Diabetes NOS	diabetes_nos	35(22,51)	58(46,73)	110(96,127)	medium
Endocrine	Diabetes NOS	diabetes_nos	37(25,52)	84(69,102)	131(114,149)	high_depr
Endocrine	Diabetes NOS	diabetes_nos	68(53,86)	155(134,178)	215(192,239)	most_depr
Endocrine	Diabetes Type I	diabetes_t1	45(31,63)	43(33,56)	73(61,86)	least_depr
Endocrine	Diabetes Type I	diabetes_t1	44(30,63)	56(44,71)	81(68,94)	low_depr
Endocrine	Diabetes Type I	diabetes_t1	55(40,75)	60(47,75)	85(72,99)	medium
Endocrine	Diabetes Type I	diabetes_t1	61(46,79)	83(68,100)	96(82,113)	high_depr
Endocrine	Diabetes Type I	diabetes_t1	87(70,107)	122(104,143)	166(146,187)	most_depr
Endocrine	Diabetes Type II	diabetes_t2	121(96,150)	302(272,334)	561(528,596)	least_depr
Endocrine	Diabetes Type II	diabetes_t2	155(127,188)	340(307,374)	657(621,694)	low_depr
Endocrine	Diabetes Type II	diabetes_t2	194(164,229)	440(403,478)	693(656,733)	medium
Endocrine	Diabetes Type II	diabetes_t2	270(236,306)	506(467,546)	867(823,913)	high_depr
Endocrine	Diabetes Type II	diabetes_t2	437(397,479)	824(776,874)	1261(1206,1318)	most_depr
Endocrine	Hyperparathyroidism	PTH	10(4,20)	23(15,32)	39(31,49)	least_depr
Endocrine	Hyperparathyroidism	PTH	13(6,24)	22(15,32)	50(41,61)	low_depr
Endocrine	Hyperparathyroidism	PTH	4(1,11)	29(21,40)	44(35,55)	medium
Endocrine	Hyperparathyroidism	PTH	22(14,34)	32(23,43)	51(41,62)	high_depr
Endocrine	Hyperparathyroidism	PTH	18(11,28)	30(21,40)	62(51,76)	most_depr
Endocrine	Hyperthyroidism	hyperthyroid	100(79,126)	133(115,154)	158(141,176)	least_depr
Endocrine	Hyperthyroidism	hyperthyroid	103(82,129)	134(115,154)	167(150,186)	low_depr
Endocrine	Hyperthyroidism	hyperthyroid	110(88,136)	149(130,170)	169(151,188)	medium
Endocrine	Hyperthyroidism	hyperthyroid	114(93,138)	173(153,196)	183(164,204)	high_depr
Endocrine	Hyperthyroidism	hyperthyroid	124(104,147)	175(154,198)	188(168,210)	most_depr
Endocrine	Hypo or hyperthyroidism	thyroid	391(348,438)	617(577,659)	792(754,832)	least_depr
Endocrine	Hypo or hyperthyroidism	thyroid	470(422,521)	618(577,660)	848(809,889)	low_depr
Endocrine	Hypo or hyperthyroidism	thyroid	404(361,450)	677(635,721)	847(806,888)	medium
Endocrine	Hypo or hyperthyroidism	thyroid	475(432,521)	724(681,770)	905(862,950)	high_depr

Endocrine	Hypo or hyperthyroidism	thyroid	534(491,580)	784(738,831)	1001(953,1050)	most_depr
Endocrine	Hypothyroidism	hypothyroid	297(260,338)	507(471,545)	675(640,712)	least_depr
Endocrine	Hypothyroidism	hypothyroid	386(343,433)	513(476,552)	711(675,749)	low_depr
Endocrine	Hypothyroidism	hypothyroid	312(275,353)	563(524,603)	713(676,751)	medium
Endocrine	Hypothyroidism	hypothyroid	373(335,413)	590(552,631)	781(741,822)	high_depr
Endocrine	Hypothyroidism	hypothyroid	438(399,479)	663(622,707)	863(819,909)	most_depr
Endocrine	Obesity	obesity	541(489,598)	744(697,792)	828(788,869)	least_depr
Endocrine	Obesity	obesity	630(574,691)	845(796,897)	923(881,967)	low_depr
Endocrine	Obesity	obesity	752(692,816)	966(913,1020)	1029(984,1076)	medium
Endocrine	Obesity	obesity	846(787,908)	1097(1042,1155)	1194(1143,1247)	high_depr
Endocrine	Obesity	obesity	1270(1203,1340)	1582(1516,1651)	1679(1616,1744)	most_depr
Endocrine	Polycystic ovarian syndrome	PCOS	67(50,87)	19(12,27)	3(1,6)	least_depr
Endocrine	Polycystic ovarian syndrome	PCOS	54(39,73)	21(14,29)	3(1,7)	low_depr
Endocrine	Polycystic ovarian syndrome	PCOS	69(52,89)	13(8,20)	6(3,10)	medium
Endocrine	Polycystic ovarian syndrome	PCOS	64(49,82)	24(17,33)	5(2,9)	high_depr
Endocrine	Polycystic ovarian syndrome	PCOS	85(68,104)	23(16,32)	8(4,13)	most_depr
Endocrine	Syndrome of inappropriate secretion of antidiuretic hormone	SIADH	0(NaN,NaN)	2(0,6)	4(2,8)	least_depr
Endocrine	Syndrome of inappropriate secretion of antidiuretic hormone	SIADH	0(NaN,NaN)	3(1,9)	5(2,9)	low_depr
Endocrine	Syndrome of inappropriate secretion of antidiuretic hormone	SIADH	0(NaN,NaN)	2(0,6)	6(3,10)	medium
Endocrine	Syndrome of inappropriate secretion of antidiuretic hormone	SIADH	0(NaN,NaN)	3(1,8)	4(1,8)	high_depr
Endocrine	Syndrome of inappropriate secretion of antidiuretic hormone	SIADH	2(0,7)	1(0,5)	10(6,17)	most_depr
Endocrine	Thyroiditis unspecified	thyroid_nos	122(98,149)	173(152,196)	204(185,225)	least_depr
Endocrine	Thyroiditis unspecified	thyroid_nos	129(105,158)	151(131,173)	213(194,234)	low_depr
Endocrine	Thyroiditis unspecified	thyroid_nos	103(82,127)	178(156,201)	225(205,247)	medium
Endocrine	Thyroiditis unspecified	thyroid_nos	108(88,131)	179(158,202)	225(203,247)	high_depr

Endocrine	Thyroiditis unspecified	thyroid_nos	116(96,138)	182(161,206)	258(234,284)	most_depr
Eye	Anterior and Intermediate Uveitis	ant_uveitis	44(30,62)	75(61,92)	90(77,104)	least_depr
Eye	Anterior and Intermediate Uveitis	ant_uveitis	63(46,84)	81(67,98)	92(79,106)	low_depr
Eye	Anterior and Intermediate Uveitis	ant_uveitis	69(52,90)	81(66,98)	86(73,100)	medium
Eye	Anterior and Intermediate Uveitis	ant_uveitis	65(49,84)	92(76,109)	87(73,102)	high_depr
Eye	Anterior and Intermediate Uveitis	ant_uveitis	64(49,81)	95(79,113)	79(66,95)	most_depr
Eye	Cataract	cataract	96(74,121)	282(253,312)	928(886,972)	least_depr
Eye	Cataract	cataract	92(71,118)	267(240,297)	916(874,959)	low_depr
Eye	Cataract	cataract	114(91,141)	348(317,382)	952(909,997)	medium
Eye	Cataract	cataract	111(90,136)	323(293,355)	1079(1030,1129)	high_depr
Eye	Cataract	cataract	142(120,167)	420(386,457)	1220(1166,1275)	most_depr
Eye	Diabetic ophthalmic complications	diab_eye	89(68,114)	182(159,208)	306(282,332)	least_depr
Eye	Diabetic ophthalmic complications	diab_eye	105(82,132)	202(177,229)	339(314,367)	low_depr
Eye	Diabetic ophthalmic complications	diab_eye	126(102,155)	242(215,271)	377(349,406)	medium
Eye	Diabetic ophthalmic complications	diab_eye	178(151,209)	296(267,328)	448(416,481)	high_depr
Eye	Diabetic ophthalmic complications	diab_eye	254(224,286)	458(422,496)	649(610,691)	most_depr
Eye	Glaucoma	glaucoma	43(29,61)	151(130,174)	346(321,374)	least_depr
Eye	Glaucoma	glaucoma	54(38,74)	135(116,157)	348(322,375)	low_depr
Eye	Glaucoma	glaucoma	46(32,65)	147(126,169)	330(304,357)	medium
Eye	Glaucoma	glaucoma	58(43,76)	155(134,178)	348(320,377)	high_depr
Eye	Glaucoma	glaucoma	77(61,96)	186(164,211)	374(344,405)	most_depr
Eye	Macular degeneration	macula_degen	22(13,36)	42(32,55)	148(131,166)	least_depr
Eye	Macular degeneration	macula_degen	18(10,31)	33(24,45)	119(104,136)	low_depr
Eye	Macular degeneration	macula_degen	15(7,27)	43(32,56)	149(132,167)	medium
Eye	Macular degeneration	macula_degen	26(16,39)	43(33,56)	165(147,186)	high_depr
Eye	Macular degeneration	macula_degen	18(11,28)	45(35,58)	178(158,200)	most_depr
Eye	Posterior Uveitis	post_uveitis	6(2,14)	6(3,12)	9(5,14)	least_depr
Eye	Posterior Uveitis	post_uveitis	4(1,11)	10(5,17)	11(7,17)	low_depr
Eye	Posterior Uveitis	post_uveitis	3(0,10)	13(8,21)	10(6,16)	medium
Eye	Posterior Uveitis	post_uveitis	9(4,17)	9(5,16)	11(6,17)	high_depr
Eye	Posterior Uveitis	post_uveitis	16(9,25)	12(7,20)	15(10,23)	most_depr
Eye	Ptosis of eyelid	ptosis	19(11,32)	38(28,50)	74(62,87)	least_depr
Eye	Ptosis of eyelid	ptosis	22(12,36)	45(34,57)	71(59,83)	low_depr

Eye	Ptosis of eyelid	ptosis	22(13,36)	40(30,52)	72(61,85)	medium
Eye	Ptosis of eyelid	ptosis	34(23,48)	49(38,62)	73(61,87)	high_depr
Eye	Ptosis of eyelid	ptosis	37(27,51)	56(45,70)	76(63,90)	most_depr
Eye	Retinal detachments and breaks	retinal_detach	50(35,69)	79(64,96)	141(125,159)	least_depr
Eye	Retinal detachments and breaks	retinal_detach	48(33,67)	90(74,109)	150(134,169)	low_depr
Eye	Retinal detachments and breaks	retinal_detach	40(27,58)	95(79,114)	157(140,176)	medium
Eye	Retinal detachments and breaks	retinal_detach	43(30,59)	94(78,112)	156(138,176)	high_depr
Eye	Retinal detachments and breaks	retinal_detach	44(32,59)	88(72,105)	125(108,144)	most_depr
Eye	Retinal vascular occlusions	retinal_vasc_occl	13(6,25)	26(18,36)	78(66,91)	least_depr
Eye	Retinal vascular occlusions	retinal_vasc_occl	12(5,24)	29(21,41)	80(68,94)	low_depr
Eye	Retinal vascular occlusions	retinal_vasc_occl	11(5,22)	28(19,39)	67(56,80)	medium
Eye	Retinal vascular occlusions	retinal_vasc_occl	17(9,29)	28(20,39)	74(61,88)	high_depr
Eye	Retinal vascular occlusions	retinal_vasc_occl	10(5,18)	37(27,49)	73(61,88)	most_depr
Eye	Scleritis and episcleritis	scleritis	23(13,37)	29(21,40)	39(31,49)	least_depr
Eye	Scleritis and episcleritis	scleritis	21(12,35)	33(24,44)	27(20,35)	low_depr
Eye	Scleritis and episcleritis	scleritis	16(8,27)	31(22,42)	28(21,37)	medium
Eye	Scleritis and episcleritis	scleritis	24(15,37)	31(22,42)	31(23,40)	high_depr
Eye	Scleritis and episcleritis	scleritis	17(10,27)	25(17,34)	34(26,44)	most_depr
Eye	Visual impairment and blindness	blind	20(11,33)	37(27,49)	65(54,78)	least_depr
Eye	Visual impairment and blindness	blind	20(10,34)	36(26,48)	69(58,82)	low_depr
Eye	Visual impairment and blindness	blind	38(26,55)	60(47,75)	66(55,78)	medium
Eye	Visual impairment and blindness	blind	25(16,38)	57(44,71)	76(64,91)	high_depr
Eye	Visual impairment and blindness	blind	72(57,91)	87(72,105)	110(94,127)	most_depr
Genitourinary	Acute Kidney Injury	AKI	20(11,33)	47(36,61)	110(96,126)	least_depr
Genitourinary	Acute Kidney Injury	AKI	29(18,45)	50(38,64)	127(111,144)	low_depr
Genitourinary	Acute Kidney Injury	AKI	26(16,41)	56(44,71)	131(115,149)	medium
Genitourinary	Acute Kidney Injury	AKI	31(20,45)	76(61,92)	173(153,194)	high_depr

Genitourinary	Acute Kidney Injury	AKI	50(37,65)	126(108,147)	266(241,293)	most_depr
Genitourinary	Chronic Kidney Disease	CKD	66(48,87)	114(96,134)	323(298,349)	least_depr
Genitourinary	Chronic Kidney Disease	CKD	49(34,68)	114(96,134)	362(336,390)	low_depr
Genitourinary	Chronic Kidney Disease	CKD	46(32,65)	141(121,163)	381(353,410)	medium
Genitourinary	Chronic Kidney Disease	CKD	65(49,84)	197(174,223)	396(366,427)	high_depr
Genitourinary	Chronic Kidney Disease	CKD	102(83,123)	197(174,222)	552(516,590)	most_depr
Genitourinary	Dysmenorrhoea	dysmenorrhoea	297(260,337)	222(199,247)	86(73,99)	least_depr
Genitourinary	Dysmenorrhoea	dysmenorrhoea	337(298,380)	209(186,233)	79(68,92)	low_depr
Genitourinary	Dysmenorrhoea	dysmenorrhoea	312(276,352)	220(197,245)	92(80,106)	medium
Genitourinary	Dysmenorrhoea	dysmenorrhoea	348(313,387)	232(209,257)	91(78,106)	high_depr
Genitourinary	Dysmenorrhoea	dysmenorrhoea	389(353,428)	239(215,266)	95(81,111)	most_depr
Genitourinary	End stage renal disease	ESRD	14(7,25)	19(12,28)	25(18,33)	least_depr
Genitourinary	End stage renal disease	ESRD	16(8,28)	12(7,20)	35(27,45)	low_depr
Genitourinary	End stage renal disease	ESRD	10(4,21)	26(18,36)	31(24,40)	medium
Genitourinary	End stage renal disease	ESRD	17(9,28)	39(29,51)	38(29,48)	high_depr
Genitourinary	End stage renal disease	ESRD	27(18,40)	40(30,53)	68(55,82)	most_depr
Genitourinary	Endometrial hyperplasia and hypertrophy	endometrial_hyper	54(39,73)	66(53,80)	54(44,65)	least_depr
Genitourinary	Endometrial hyperplasia and hypertrophy	endometrial_hyper	55(40,74)	70(57,85)	56(47,67)	low_depr
Genitourinary	Endometrial hyperplasia and hypertrophy	endometrial_hyper	47(33,63)	67(55,81)	55(45,66)	medium
Genitourinary	Endometrial hyperplasia and hypertrophy	endometrial_hyper	54(41,71)	67(54,81)	52(42,63)	high_depr
Genitourinary	Endometrial hyperplasia and hypertrophy	endometrial_hyper	73(58,91)	63(51,78)	58(47,70)	most_depr
Genitourinary	Endometriosis	endometriosis	273(238,312)	195(173,218)	103(90,118)	least_depr
Genitourinary	Endometriosis	endometriosis	277(242,316)	208(185,232)	116(102,131)	low_depr
Genitourinary	Endometriosis	endometriosis	249(217,285)	201(179,225)	111(97,127)	medium
Genitourinary	Endometriosis	endometriosis	287(255,323)	206(184,230)	93(80,108)	high_depr
Genitourinary	Endometriosis	endometriosis	295(263,329)	181(160,204)	94(80,110)	most_depr
Genitourinary	Erectile dysfunction	ED	320(278,366)	676(629,725)	1042(995,1090)	least_depr

Genitourinary	Erectile dysfunction	ED	335(292,384)	633(587,682)	1071(1024,1119)	low_depr
Genitourinary	Erectile dysfunction	ED	330(288,376)	700(652,751)	1091(1043,1142)	medium
Genitourinary	Erectile dysfunction	ED	426(382,474)	746(697,798)	1115(1063,1168)	high_depr
Genitourinary	Erectile dysfunction	ED	508(465,554)	869(817,922)	1135(1081,1191)	most_depr
Genitourinary	Female genital prolapse	female_genital_prolapse	242(209,278)	389(358,422)	626(593,662)	least_depr
Genitourinary	Female genital prolapse	female_genital_prolapse	218(187,253)	383(352,416)	650(616,685)	low_depr
Genitourinary	Female genital prolapse	female_genital_prolapse	215(185,249)	362(333,394)	625(591,660)	medium
Genitourinary	Female genital prolapse	female_genital_prolapse	189(163,218)	369(339,400)	626(591,663)	high_depr
Genitourinary	Female genital prolapse	female_genital_prolapse	202(176,231)	339(310,370)	626(589,665)	most_depr
Genitourinary	Female infertility	female_infertility	244(211,281)	127(110,147)	68(57,80)	least_depr
Genitourinary	Female infertility	female_infertility	215(184,249)	109(93,127)	60(50,72)	low_depr
Genitourinary	Female infertility	female_infertility	222(192,256)	103(87,120)	57(47,68)	medium
Genitourinary	Female infertility	female_infertility	229(200,261)	109(93,127)	56(46,68)	high_depr
Genitourinary	Female infertility	female_infertility	174(150,200)	88(73,104)	49(39,60)	most_depr
Genitourinary	Glomerulonephritis	GN	42(28,59)	42(31,55)	70(59,83)	least_depr
Genitourinary	Glomerulonephritis	GN	41(27,59)	34(25,46)	70(59,83)	low_depr
Genitourinary	Glomerulonephritis	GN	29(18,44)	54(42,69)	66(55,79)	medium
Genitourinary	Glomerulonephritis	GN	37(25,52)	69(56,85)	89(76,105)	high_depr
Genitourinary	Glomerulonephritis	GN	49(37,65)	71(57,87)	115(99,133)	most_depr
Genitourinary	Hydrocoele (incl infected)	hydrocele	81(61,106)	65(51,82)	107(93,124)	least_depr
Genitourinary	Hydrocoele (incl infected)	hydrocele	85(64,111)	88(71,107)	108(94,125)	low_depr
Genitourinary	Hydrocoele (incl infected)	hydrocele	74(55,97)	73(58,90)	120(104,137)	medium
Genitourinary	Hydrocoele (incl infected)	hydrocele	66(49,86)	67(53,83)	122(106,141)	high_depr
Genitourinary	Hydrocoele (incl infected)	hydrocele	56(43,73)	69(55,85)	113(96,131)	most_depr
Genitourinary	Hyperplasia of prostate	BPH	78(58,103)	330(297,365)	834(793,877)	least_depr
Genitourinary	Hyperplasia of prostate	BPH	65(47,88)	311(279,345)	885(842,929)	low_depr

Genitourinary	Hyperplasia of prostate	BPH	78(59,102)	315(283,350)	865(821,910)	medium
Genitourinary	Hyperplasia of prostate	BPH	91(71,114)	310(279,344)	834(789,881)	high_depr
Genitourinary	Hyperplasia of prostate	BPH	80(64,100)	313(282,346)	832(786,880)	most_depr
Genitourinary	Male infertility	male_infertility	67(49,90)	38(27,51)	29(21,37)	least_depr
Genitourinary	Male infertility	male_infertility	59(41,81)	38(27,51)	13(8,20)	low_depr
Genitourinary	Male infertility	male_infertility	41(27,60)	38(28,51)	18(13,26)	medium
Genitourinary	Male infertility	male_infertility	63(47,83)	35(25,48)	18(12,26)	high_depr
Genitourinary	Male infertility	male_infertility	44(32,59)	30(21,41)	15(9,22)	most_depr
Genitourinary	Menorrhagia and polymenorrhoea	menorrhagia	1132(1060,1209)	980(930,1031)	526(496,559)	least_depr
Genitourinary	Menorrhagia and polymenorrhoea	menorrhagia	1175(1101,1253)	974(925,1026)	475(446,505)	low_depr
Genitourinary	Menorrhagia and polymenorrhoea	menorrhagia	1136(1066,1210)	999(949,1050)	499(469,530)	medium
Genitourinary	Menorrhagia and polymenorrhoea	menorrhagia	1172(1106,1241)	953(905,1003)	470(440,502)	high_depr
Genitourinary	Menorrhagia and polymenorrhoea	menorrhagia	1250(1184,1318)	954(905,1005)	476(443,510)	most_depr
Genitourinary	Neuromuscular dysfunction of bladder	neuro_bladder	93(72,118)	133(114,154)	217(197,239)	least_depr
Genitourinary	Neuromuscular dysfunction of bladder	neuro_bladder	83(63,107)	126(107,146)	193(174,213)	low_depr
Genitourinary	Neuromuscular dysfunction of bladder	neuro_bladder	92(72,115)	163(142,186)	191(171,211)	medium
Genitourinary	Neuromuscular dysfunction of bladder	neuro_bladder	90(71,111)	137(118,158)	225(203,248)	high_depr
Genitourinary	Neuromuscular dysfunction of bladder	neuro_bladder	137(115,161)	199(176,224)	262(237,288)	most_depr
Genitourinary	Non-acute cystitis	chr_cystitis	10(4,20)	16(10,24)	38(30,47)	least_depr
Genitourinary	Non-acute cystitis	chr_cystitis	10(4,20)	13(7,20)	40(32,50)	low_depr
Genitourinary	Non-acute cystitis	chr_cystitis	20(12,33)	27(19,38)	38(30,48)	medium
Genitourinary	Non-acute cystitis	chr_cystitis	17(10,28)	34(26,46)	35(27,45)	high_depr
Genitourinary	Non-acute cystitis	chr_cystitis	19(11,29)	30(22,40)	46(36,58)	most_depr
Genitourinary	Obstructive and reflux uropathy	obstr_reflux	66(48,88)	70(56,86)	82(69,96)	least_depr
Genitourinary	Obstructive and reflux uropathy	obstr_reflux	55(39,75)	64(51,79)	104(90,119)	low_depr
Genitourinary	Obstructive and reflux uropathy	obstr_reflux	47(32,65)	64(51,79)	100(86,116)	medium
Genitourinary	Obstructive and reflux uropathy	obstr_reflux	42(30,58)	64(51,79)	98(84,115)	high_depr
Genitourinary	Obstructive and reflux uropathy	obstr_reflux	75(60,94)	90(74,108)	124(107,142)	most_depr

Genitourinary	Postcoital and contact bleeding	PCB	215(184,250)	190(169,213)	76(64,89)	least_depr
Genitourinary	Postcoital and contact bleeding	PCB	188(159,221)	148(129,169)	86(74,99)	low_depr
Genitourinary	Postcoital and contact bleeding	PCB	235(204,270)	154(135,175)	71(60,84)	medium
Genitourinary	Postcoital and contact bleeding	PCB	191(165,220)	132(115,152)	76(64,90)	high_depr
Genitourinary	Postcoital and contact bleeding	PCB	179(155,206)	128(111,148)	50(40,62)	most_depr
Genitourinary	Postmenopausal bleeding	PMB	104(83,129)	426(393,460)	584(551,618)	least_depr
Genitourinary	Postmenopausal bleeding	PMB	84(65,107)	432(399,466)	591(559,625)	low_depr
Genitourinary	Postmenopausal bleeding	PMB	73(56,94)	418(386,451)	611(578,646)	medium
Genitourinary	Postmenopausal bleeding	PMB	86(69,106)	434(402,468)	579(545,615)	high_depr
Genitourinary	Postmenopausal bleeding	PMB	113(94,135)	442(409,477)	591(555,629)	most_depr
Genitourinary	Tubulo-interstitial nephritis	TIN	17(9,28)	23(15,33)	22(16,29)	least_depr
Genitourinary	Tubulo-interstitial nephritis	TIN	12(5,22)	18(12,27)	19(14,26)	low_depr
Genitourinary	Tubulo-interstitial nephritis	TIN	16(9,28)	25(17,35)	17(12,25)	medium
Genitourinary	Tubulo-interstitial nephritis	TIN	29(19,42)	24(16,33)	24(18,33)	high_depr
Genitourinary	Tubulo-interstitial nephritis	TIN	41(29,55)	31(23,42)	30(22,40)	most_depr
Genitourinary	Undescended testicle	undescended_testis	23(13,38)	18(11,27)	12(7,18)	least_depr
Genitourinary	Undescended testicle	undescended_testis	35(22,53)	18(11,28)	13(8,19)	low_depr
Genitourinary	Undescended testicle	undescended_testis	28(17,44)	13(7,22)	13(8,20)	medium
Genitourinary	Undescended testicle	undescended_testis	17(10,29)	13(7,21)	12(7,19)	high_depr
Genitourinary	Undescended testicle	undescended_testis	16(9,26)	15(9,23)	11(6,18)	most_depr
Genitourinary	Urinary Incontinence	urine_incont	311(273,352)	411(379,445)	539(508,572)	least_depr
Genitourinary	Urinary Incontinence	urine_incont	331(292,373)	424(392,459)	541(509,573)	low_depr
Genitourinary	Urinary Incontinence	urine_incont	310(273,349)	481(447,518)	552(520,585)	medium
Genitourinary	Urinary Incontinence	urine_incont	351(315,390)	468(434,503)	599(565,636)	high_depr
Genitourinary	Urinary Incontinence	urine_incont	437(398,478)	561(523,601)	707(667,748)	most_depr
Genitourinary	Urolithiasis	urolithiasis	185(154,220)	282(253,313)	326(301,353)	least_depr
Genitourinary	Urolithiasis	urolithiasis	210(177,248)	299(268,332)	364(337,392)	low_depr

Genitourinary	Urolithiasis	urolithiasis	196(164,231)	306(276,338)	370(343,400)	medium
Genitourinary	Urolithiasis	urolithiasis	203(174,236)	281(252,312)	367(339,398)	high_depr
Genitourinary	Urolithiasis	urolithiasis	249(219,282)	344(313,377)	383(353,416)	most_depr
Haematological or immunological	Agranulocytosis	agranulocytosi s	80(61,104)	122(104,142)	156(140,175)	least_depr
Haematological or immunological	Agranulocytosis	agranulocytosi s	72(54,94)	117(99,137)	142(126,160)	low_depr
Haematological or immunological	Agranulocytosis	agranulocytosi s	77(59,99)	110(93,130)	149(132,168)	medium
Haematological or immunological	Agranulocytosis	agranulocytosi s	106(86,129)	115(98,134)	148(130,167)	high_depr
Haematological or immunological	Agranulocytosis	agranulocytosi s	80(64,99)	143(124,164)	151(132,171)	most_depr
Haematological or immunological	Aplastic anaemias	aplastic	5(1,13)	10(5,17)	20(14,27)	least_depr
Haematological or immunological	Aplastic anaemias	aplastic	11(5,21)	13(8,21)	16(10,22)	low_depr
Haematological or immunological	Aplastic anaemias	aplastic	7(2,16)	12(7,19)	18(12,25)	medium
Haematological or immunological	Aplastic anaemias	aplastic	5(2,12)	11(6,19)	17(11,24)	high_depr
Haematological or immunological	Aplastic anaemias	aplastic	7(3,14)	21(13,30)	20(14,29)	most_depr
Haematological or immunological	Folate deficiency anaemia	folatedef	9(4,18)	14(8,21)	21(15,29)	least_depr
Haematological or immunological	Folate deficiency anaemia	folatedef	24(14,38)	14(9,23)	35(27,45)	low_depr
Haematological or immunological	Folate deficiency anaemia	folatedef	29(18,44)	19(12,28)	26(19,34)	medium
Haematological or immunological	Folate deficiency anaemia	folatedef	29(19,42)	31(22,42)	37(28,47)	high_depr
Haematological or immunological	Folate deficiency anaemia	folatedef	46(34,61)	47(36,60)	63(51,76)	most_depr
Haematological or immunological	Hyposplenism	hyposplenism	14(7,27)	19(12,28)	22(16,30)	least_depr

Haematological or immunological	Hyposplenism	hyposplenism	21(11,34)	22(15,32)	25(18,33)	low_depr
Haematological or immunological	Hyposplenism	hyposplenism	21(12,34)	17(11,26)	23(17,31)	medium
Haematological or immunological	Hyposplenism	hyposplenism	12(6,22)	28(19,38)	20(14,28)	high_depr
Haematological or immunological	Hyposplenism	hyposplenism	20(13,31)	23(16,33)	33(24,43)	most_depr
Haematological or immunological	Immunodeficiencies	immunodef	7(2,17)	2(0,7)	7(3,11)	least_depr
Haematological or immunological	Immunodeficiencies	immunodef	1(0,7)	6(3,12)	9(5,15)	low_depr
Haematological or immunological	Immunodeficiencies	immunodef	5(1,12)	3(1,8)	5(3,10)	medium
Haematological or immunological	Immunodeficiencies	immunodef	10(5,19)	4(2,9)	5(2,9)	high_depr
Haematological or immunological	Immunodeficiencies	immunodef	8(4,16)	9(4,15)	12(7,19)	most_depr
Haematological or immunological	Iron deficiency anaemia	IDA	295(258,335)	302(274,332)	314(289,339)	least_depr
Haematological or immunological	Iron deficiency anaemia	IDA	266(231,305)	338(308,370)	345(319,372)	low_depr
Haematological or immunological	Iron deficiency anaemia	IDA	332(294,374)	359(328,392)	369(343,398)	medium
Haematological or immunological	Iron deficiency anaemia	IDA	323(288,361)	350(320,382)	402(372,433)	high_depr
Haematological or immunological	Iron deficiency anaemia	IDA	371(335,409)	435(401,471)	508(473,544)	most_depr
Haematological or immunological	Other anaemias	oth_anaemia	431(386,480)	459(424,496)	526(494,559)	least_depr
Haematological or immunological	Other anaemias	oth_anaemia	404(360,451)	494(458,533)	564(532,598)	low_depr
Haematological or immunological	Other anaemias	oth_anaemia	468(423,518)	520(483,560)	603(569,639)	medium

Haematological or immunological	Other anaemias	oth_anaemia	502(458,548)	552(514,592)	684(646,724)	high_depr
Haematological or immunological	Other anaemias	oth_anaemia	544(500,590)	654(612,698)	840(795,886)	most_depr
Haematological or immunological	Other haemolytic anaemias	oth_haem_anaemia	13(6,25)	11(6,19)	13(8,19)	least_depr
Haematological or immunological	Other haemolytic anaemias	oth_haem_anaemia	11(5,22)	10(5,17)	12(8,18)	low_depr
Haematological or immunological	Other haemolytic anaemias	oth_haem_anaemia	6(2,15)	11(6,19)	9(5,15)	medium
Haematological or immunological	Other haemolytic anaemias	oth_haem_anaemia	8(3,16)	9(5,16)	14(9,21)	high_depr
Haematological or immunological	Other haemolytic anaemias	oth_haem_anaemia	12(7,21)	14(8,22)	17(11,25)	most_depr
Haematological or immunological	Primary or Idiopathic Thrombocytopaenia	pri_thrombocytopaenia	13(6,24)	26(18,36)	50(40,61)	least_depr
Haematological or immunological	Primary or Idiopathic Thrombocytopaenia	pri_thrombocytopaenia	32(21,48)	35(26,48)	51(42,63)	low_depr
Haematological or immunological	Primary or Idiopathic Thrombocytopaenia	pri_thrombocytopaenia	27(16,41)	34(24,45)	44(35,55)	medium
Haematological or immunological	Primary or Idiopathic Thrombocytopaenia	pri_thrombocytopaenia	21(12,32)	45(34,58)	52(41,64)	high_depr
Haematological or immunological	Primary or Idiopathic Thrombocytopaenia	pri_thrombocytopaenia	35(25,49)	49(38,63)	49(39,61)	most_depr
Haematological or immunological	Sarcoidosis	sarcoid	33(21,49)	33(24,44)	44(35,55)	least_depr
Haematological or immunological	Sarcoidosis	sarcoid	24(13,39)	33(23,44)	32(25,41)	low_depr
Haematological or immunological	Sarcoidosis	sarcoid	24(14,38)	41(31,54)	36(28,45)	medium
Haematological or immunological	Sarcoidosis	sarcoid	34(23,49)	31(22,42)	50(40,61)	high_depr
Haematological or immunological	Sarcoidosis	sarcoid	42(30,56)	37(27,48)	49(39,61)	most_depr

Haematological or immunological	Secondary or other Thrombocytopaenia	sec_oth_thrombocytopaenia	21(12,35)	37(27,48)	65(54,78)	least_depr
Haematological or immunological	Secondary or other Thrombocytopaenia	sec_oth_thrombocytopaenia	34(22,50)	53(41,68)	72(60,85)	low_depr
Haematological or immunological	Secondary or other Thrombocytopaenia	sec_oth_thrombocytopaenia	31(20,46)	39(29,51)	62(51,74)	medium
Haematological or immunological	Secondary or other Thrombocytopaenia	sec_oth_thrombocytopaenia	37(26,52)	55(43,69)	73(60,87)	high_depr
Haematological or immunological	Secondary or other Thrombocytopaenia	sec_oth_thrombocytopaenia	44(32,59)	62(50,77)	80(67,96)	most_depr
Haematological or immunological	Secondary polycythaemia	2ry_polycythaemia	6(2,14)	8(4,15)	9(5,14)	least_depr
Haematological or immunological	Secondary polycythaemia	2ry_polycythaemia	4(1,13)	7(3,13)	13(9,20)	low_depr
Haematological or immunological	Secondary polycythaemia	2ry_polycythaemia	6(2,14)	9(5,16)	8(4,13)	medium
Haematological or immunological	Secondary polycythaemia	2ry_polycythaemia	4(1,12)	6(3,12)	11(7,18)	high_depr
Haematological or immunological	Secondary polycythaemia	2ry_polycythaemia	12(6,21)	15(9,24)	17(12,25)	most_depr
Haematological or immunological	Sickle-cell anaemia	sickle_cell	1(0,7)	0(NaN,NaN)	0(NaN,NaN)	least_depr
Haematological or immunological	Sickle-cell anaemia	sickle_cell	0(NaN,NaN)	2(0,7)	1(0,3)	low_depr
Haematological or immunological	Sickle-cell anaemia	sickle_cell	3(1,10)	2(0,6)	0(0,3)	medium
Haematological or immunological	Sickle-cell anaemia	sickle_cell	2(0,8)	3(1,8)	2(1,6)	high_depr
Haematological or immunological	Sickle-cell anaemia	sickle_cell	9(4,16)	6(2,11)	2(1,6)	most_depr
Haematological or immunological	Sickle-cell trait	sickle_trait	6(2,14)	1(0,4)	0(0,3)	least_depr
Haematological or immunological	Sickle-cell trait	sickle_trait	3(0,9)	4(1,9)	3(1,7)	low_depr

Haematological or immunological	Sickle-cell trait	sickle_trait	8(3,17)	3(1,8)	3(1,7)	medium
Haematological or immunological	Sickle-cell trait	sickle_trait	8(4,17)	6(3,11)	5(2,9)	high_depr
Haematological or immunological	Sickle-cell trait	sickle_trait	30(21,43)	29(21,39)	12(7,18)	most_depr
Haematological or immunological	Splenomegaly	splenomegaly	7(2,17)	9(4,16)	10(6,16)	least_depr
Haematological or immunological	Splenomegaly	splenomegaly	9(3,19)	7(3,14)	15(10,21)	low_depr
Haematological or immunological	Splenomegaly	splenomegaly	4(1,12)	15(9,23)	18(12,26)	medium
Haematological or immunological	Splenomegaly	splenomegaly	11(5,21)	18(11,27)	20(14,28)	high_depr
Haematological or immunological	Splenomegaly	splenomegaly	7(3,14)	26(18,37)	18(12,26)	most_depr
Haematological or immunological	Thalassaemia	thala	8(3,18)	8(4,15)	4(1,8)	least_depr
Haematological or immunological	Thalassaemia	thala	2(0,9)	6(3,12)	2(1,5)	low_depr
Haematological or immunological	Thalassaemia	thala	5(1,14)	3(1,8)	3(1,7)	medium
Haematological or immunological	Thalassaemia	thala	7(3,15)	11(6,19)	5(2,9)	high_depr
Haematological or immunological	Thalassaemia	thala	9(5,17)	4(2,9)	6(3,10)	most_depr
Haematological or immunological	Thalassaemia trait	thal_trait	11(5,21)	7(3,13)	5(3,10)	least_depr
Haematological or immunological	Thalassaemia trait	thal_trait	5(1,14)	5(2,10)	4(2,8)	low_depr
Haematological or immunological	Thalassaemia trait	thal_trait	15(8,27)	7(3,13)	9(5,15)	medium
Haematological or immunological	Thalassaemia trait	thal_trait	22(14,34)	20(13,30)	12(7,19)	high_depr

Haematological or immunological	Thalassaemia trait	thal_trait	25(16,36)	23(15,32)	12(7,19)	most_depr
Haematological or immunological	Thrombophilia	thrombophilia	34(22,50)	32(24,43)	30(23,39)	least_depr
Haematological or immunological	Thrombophilia	thrombophilia	40(27,58)	24(16,33)	32(24,41)	low_depr
Haematological or immunological	Thrombophilia	thrombophilia	36(24,52)	44(34,57)	28(21,37)	medium
Haematological or immunological	Thrombophilia	thrombophilia	39(28,54)	35(26,47)	31(23,40)	high_depr
Haematological or immunological	Thrombophilia	thrombophilia	42(30,56)	36(27,48)	35(27,46)	most_depr
Haematological or immunological	Vitamin B12 deficiency anaemia	b12_def	69(52,91)	85(70,102)	136(120,154)	least_depr
Haematological or immunological	Vitamin B12 deficiency anaemia	b12_def	76(57,98)	93(77,111)	145(129,163)	low_depr
Haematological or immunological	Vitamin B12 deficiency anaemia	b12_def	80(61,102)	109(92,128)	144(127,162)	medium
Haematological or immunological	Vitamin B12 deficiency anaemia	b12_def	108(88,131)	138(119,159)	167(149,188)	high_depr
Haematological or immunological	Vitamin B12 deficiency anaemia	b12_def	163(140,190)	209(185,235)	269(244,296)	most_depr
Infections	Bacterial Diseases (excl TB)	bacterial	677(619,739)	742(696,790)	923(881,967)	least_depr
Infections	Bacterial Diseases (excl TB)	bacterial	670(612,733)	733(687,782)	1005(961,1050)	low_depr
Infections	Bacterial Diseases (excl TB)	bacterial	752(692,816)	854(805,906)	1003(958,1049)	medium
Infections	Bacterial Diseases (excl TB)	bacterial	805(748,866)	879(830,931)	1170(1119,1223)	high_depr
Infections	Bacterial Diseases (excl TB)	bacterial	1011(951,1074)	1256(1197,1317)	1484(1425,1546)	most_depr
Infections	Chronic viral hepatitis	chr_hep	24(14,38)	28(20,39)	18(13,25)	least_depr
Infections	Chronic viral hepatitis	chr_hep	24(14,38)	27(19,38)	16(11,22)	low_depr
Infections	Chronic viral hepatitis	chr_hep	33(22,49)	32(23,43)	22(15,29)	medium
Infections	Chronic viral hepatitis	chr_hep	34(23,48)	41(30,53)	31(23,40)	high_depr
Infections	Chronic viral hepatitis	chr_hep	79(63,99)	71(57,87)	39(30,51)	most_depr

Infections	Ear and Upper Respiratory Tract Infections	ear_urti	133(108,163)	116(98,136)	129(114,146)	least_depr
Infections	Ear and Upper Respiratory Tract Infections	ear_urti	117(93,145)	119(101,140)	144(128,162)	low_depr
Infections	Ear and Upper Respiratory Tract Infections	ear_urti	157(130,188)	137(118,159)	131(115,148)	medium
Infections	Ear and Upper Respiratory Tract Infections	ear_urti	162(137,191)	138(119,160)	146(129,165)	high_depr
Infections	Ear and Upper Respiratory Tract Infections	ear_urti	210(183,240)	179(157,203)	185(165,208)	most_depr
Infections	Encephalitis	enceph	2(0,9)	5(2,10)	5(2,9)	least_depr
Infections	Encephalitis	enceph	2(0,9)	2(0,6)	4(2,8)	low_depr
Infections	Encephalitis	enceph	1(0,6)	2(0,7)	1(0,4)	medium
Infections	Encephalitis	enceph	0(NaN,NaN)	2(0,5)	2(0,5)	high_depr
Infections	Encephalitis	enceph	0(NaN,NaN)	4(2,10)	3(1,8)	most_depr
Infections	Eye infections	eye	6(2,15)	10(5,18)	16(11,23)	least_depr
Infections	Eye infections	eye	4(1,12)	11(6,19)	16(11,23)	low_depr
Infections	Eye infections	eye	11(5,22)	11(6,18)	16(10,22)	medium
Infections	Eye infections	eye	12(6,21)	16(10,24)	18(13,26)	high_depr
Infections	Eye infections	eye	12(6,20)	21(14,30)	20(14,28)	most_depr
Infections	Female pelvic inflammatory disease	PID	143(118,172)	102(86,119)	66(55,78)	least_depr
Infections	Female pelvic inflammatory disease	PID	134(110,162)	98(83,115)	64(54,76)	low_depr
Infections	Female pelvic inflammatory disease	PID	160(134,189)	102(87,119)	55(45,66)	medium
Infections	Female pelvic inflammatory disease	PID	171(146,199)	101(86,118)	68(57,81)	high_depr
Infections	Female pelvic inflammatory disease	PID	199(174,228)	131(113,151)	63(52,77)	most_depr
Infections	HIV	hiv	9(3,19)	2(0,6)	0(NaN,NaN)	least_depr
Infections	HIV	hiv	5(1,14)	5(2,11)	3(1,6)	low_depr
Infections	HIV	hiv	13(6,25)	6(2,12)	2(1,6)	medium
Infections	HIV	hiv	18(10,30)	11(6,19)	3(1,7)	high_depr
Infections	HIV	hiv	52(39,68)	33(23,44)	9(5,15)	most_depr
Infections	Infection of anal and rectal regions	anorectal	50(35,70)	43(32,56)	28(21,36)	least_depr
Infections	Infection of anal and rectal regions	anorectal	37(24,55)	35(26,47)	22(16,30)	low_depr
Infections	Infection of anal and rectal regions	anorectal	53(38,72)	41(30,54)	27(20,36)	medium

Infections	Infection of anal and rectal regions	anorectal	56(41,74)	49(38,63)	39(30,50)	high_depr
Infections	Infection of anal and rectal regions	anorectal	63(49,80)	72(58,88)	46(36,58)	most_depr
Infections	Infection of bones and joints	bone	6(2,15)	14(8,22)	25(18,33)	least_depr
Infections	Infection of bones and joints	bone	16(8,28)	24(16,34)	29(22,38)	low_depr
Infections	Infection of bones and joints	bone	22(12,35)	25(17,35)	21(15,28)	medium
Infections	Infection of bones and joints	bone	16(9,28)	27(18,37)	30(22,40)	high_depr
Infections	Infection of bones and joints	bone	31(21,43)	46(35,60)	57(46,70)	most_depr
Infections	Infection of liver	liver	10(4,20)	16(10,24)	9(5,15)	least_depr
Infections	Infection of liver	liver	13(6,25)	12(7,20)	12(8,18)	low_depr
Infections	Infection of liver	liver	11(5,21)	12(7,20)	22(15,30)	medium
Infections	Infection of liver	liver	20(12,32)	27(19,38)	15(10,22)	high_depr
Infections	Infection of liver	liver	30(21,43)	48(37,62)	27(20,37)	most_depr
Infections	Infection of male genital system	male_GU	28(16,44)	24(16,35)	32(24,41)	least_depr
Infections	Infection of male genital system	male_GU	30(18,47)	31(21,43)	42(33,52)	low_depr
Infections	Infection of male genital system	male_GU	27(16,42)	27(18,38)	34(25,43)	medium
Infections	Infection of male genital system	male_GU	31(20,46)	25(17,36)	43(33,55)	high_depr
Infections	Infection of male genital system	male_GU	42(30,57)	43(32,56)	51(40,64)	most_depr
Infections	Infection of other or unspecified genitourinary system	oth_gu	29(18,43)	18(11,26)	14(9,20)	least_depr
Infections	Infection of other or unspecified genitourinary system	oth_gu	24(14,37)	16(10,23)	11(7,17)	low_depr
Infections	Infection of other or unspecified genitourinary system	oth_gu	26(16,39)	18(12,27)	14(9,20)	medium
Infections	Infection of other or unspecified genitourinary system	oth_gu	30(20,43)	21(14,30)	17(12,24)	high_depr
Infections	Infection of other or unspecified genitourinary system	oth_gu	43(31,57)	21(14,30)	15(10,22)	most_depr
Infections	Infection of skin and subcutaneous tissues	skin	192(161,228)	227(201,254)	243(222,267)	least_depr
Infections	Infection of skin and subcutaneous tissues	skin	213(180,251)	237(210,265)	299(275,324)	low_depr

Infections	Infection of skin and subcutaneous tissues	skin	215(183,252)	263(236,293)	299(274,325)	medium
Infections	Infection of skin and subcutaneous tissues	skin	215(185,248)	296(267,327)	356(328,386)	high_depr
Infections	Infection of skin and subcutaneous tissues	skin	317(283,353)	401(367,436)	453(420,488)	most_depr
Infections	Infections of Other or unspecified organs	oth_organs	263(227,303)	301(272,333)	410(382,440)	least_depr
Infections	Infections of Other or unspecified organs	oth_organs	263(226,303)	301(271,332)	463(433,494)	low_depr
Infections	Infections of Other or unspecified organs	oth_organs	276(240,316)	339(308,372)	452(422,484)	medium
Infections	Infections of Other or unspecified organs	oth_organs	298(264,336)	388(355,423)	541(507,577)	high_depr
Infections	Infections of Other or unspecified organs	oth_organs	393(356,433)	555(516,597)	727(685,770)	most_depr
Infections	Infections of the digestive system	digestive	144(117,174)	172(150,196)	223(203,245)	least_depr
Infections	Infections of the digestive system	digestive	148(121,179)	201(177,227)	272(249,296)	low_depr
Infections	Infections of the digestive system	digestive	136(111,165)	215(191,242)	282(258,307)	medium
Infections	Infections of the digestive system	digestive	193(165,223)	243(217,271)	316(290,344)	high_depr
Infections	Infections of the digestive system	digestive	231(203,262)	321(292,353)	392(361,424)	most_depr
Infections	Infections of the Heart	heart	3(0,9)	5(2,11)	8(4,13)	least_depr
Infections	Infections of the Heart	heart	6(2,15)	4(1,9)	13(8,19)	low_depr
Infections	Infections of the Heart	heart	3(0,11)	6(3,13)	6(3,11)	medium
Infections	Infections of the Heart	heart	2(0,8)	2(0,6)	8(4,13)	high_depr
Infections	Infections of the Heart	heart	3(1,9)	8(4,14)	12(7,19)	most_depr
Infections	Lower Respiratory Tract Infections	lrti	181(151,215)	204(180,230)	385(358,414)	least_depr
Infections	Lower Respiratory Tract Infections	lrti	181(151,215)	233(207,262)	475(445,507)	low_depr
Infections	Lower Respiratory Tract Infections	lrti	170(142,203)	299(270,331)	486(455,519)	medium
Infections	Lower Respiratory Tract Infections	lrti	216(187,249)	310(280,342)	562(527,599)	high_depr
Infections	Lower Respiratory Tract Infections	lrti	354(319,392)	506(468,545)	865(819,912)	most_depr
Infections	Meningitis	meningitis	14(7,26)	9(4,16)	7(4,12)	least_depr
Infections	Meningitis	meningitis	11(5,21)	3(1,8)	7(4,12)	low_depr
Infections	Meningitis	meningitis	16(8,27)	5(2,11)	6(3,10)	medium
Infections	Meningitis	meningitis	10(5,20)	8(4,15)	7(3,12)	high_depr
Infections	Meningitis	meningitis	12(7,21)	6(2,11)	4(1,8)	most_depr

Infections	Mycoses	mycoses	36(24,53)	33(24,44)	63(52,75)	least_depr
Infections	Mycoses	mycoses	45(31,63)	52(40,66)	77(65,91)	low_depr
Infections	Mycoses	mycoses	27(17,42)	42(31,54)	78(66,92)	medium
Infections	Mycoses	mycoses	49(36,66)	47(36,61)	91(78,107)	high_depr
Infections	Mycoses	mycoses	51(39,67)	74(60,90)	104(89,121)	most_depr
Infections	Other nervous system infections	oth_nerv_sys	5(1,14)	12(7,20)	17(11,24)	least_depr
Infections	Other nervous system infections	oth_nerv_sys	4(1,11)	12(7,20)	15(10,21)	low_depr
Infections	Other nervous system infections	oth_nerv_sys	13(6,23)	15(9,24)	14(9,20)	medium
Infections	Other nervous system infections	oth_nerv_sys	8(3,16)	12(7,20)	14(9,21)	high_depr
Infections	Other nervous system infections	oth_nerv_sys	10(5,18)	19(12,28)	24(17,33)	most_depr
Infections	Other or unspecified infectious organisms	oth_organisms	437(390,488)	495(458,535)	739(702,779)	least_depr
Infections	Other or unspecified infectious organisms	oth_organisms	427(381,478)	538(498,579)	871(830,913)	low_depr
Infections	Other or unspecified infectious organisms	oth_organisms	462(415,513)	598(557,642)	863(821,906)	medium
Infections	Other or unspecified infectious organisms	oth_organisms	504(459,553)	666(623,712)	966(920,1014)	high_depr
Infections	Other or unspecified infectious organisms	oth_organisms	708(657,761)	924(873,977)	1333(1276,1391)	most_depr
Infections	Parasitic infections	parasitic	7(2,15)	8(4,14)	5(2,9)	least_depr
Infections	Parasitic infections	parasitic	5(1,14)	5(2,11)	12(7,18)	low_depr
Infections	Parasitic infections	parasitic	8(3,17)	9(5,16)	8(4,13)	medium
Infections	Parasitic infections	parasitic	11(5,21)	11(6,19)	15(10,22)	high_depr
Infections	Parasitic infections	parasitic	17(10,27)	16(10,25)	14(9,22)	most_depr
Infections	Rheumatic fever	rh_fever	9(4,19)	22(14,31)	54(45,66)	least_depr
Infections	Rheumatic fever	rh_fever	2(0,9)	25(17,36)	60(49,71)	low_depr
Infections	Rheumatic fever	rh_fever	12(6,24)	28(20,39)	57(47,69)	medium
Infections	Rheumatic fever	rh_fever	15(8,26)	32(24,43)	57(47,70)	high_depr
Infections	Rheumatic fever	rh_fever	17(10,27)	45(35,58)	83(69,98)	most_depr
Infections	Septicaemia	sepsis	28(17,43)	64(51,80)	111(96,127)	least_depr
Infections	Septicaemia	sepsis	37(24,54)	71(57,87)	118(103,134)	low_depr
Infections	Septicaemia	sepsis	52(37,71)	62(49,77)	117(102,134)	medium
Infections	Septicaemia	sepsis	52(38,69)	78(64,95)	146(128,165)	high_depr
Infections	Septicaemia	sepsis	60(46,77)	106(89,125)	170(150,192)	most_depr
Infections	Tuberculosis	TB	22(13,37)	37(27,50)	86(74,101)	least_depr
Infections	Tuberculosis	TB	25(15,40)	50(38,64)	89(76,103)	low_depr
Infections	Tuberculosis	TB	33(21,49)	48(37,62)	86(73,101)	medium
Infections	Tuberculosis	TB	49(35,66)	53(41,66)	111(95,128)	high_depr

Infections	Tuberculosis	TB	79(63,98)	97(81,115)	150(131,170)	most_depr
Infections	Urinary Tract Infections	uti	171(143,204)	253(227,282)	388(361,417)	least_depr
Infections	Urinary Tract Infections	uti	155(128,187)	225(200,253)	387(360,415)	low_depr
Infections	Urinary Tract Infections	uti	175(147,206)	272(245,302)	399(371,429)	medium
Infections	Urinary Tract Infections	uti	205(177,236)	277(250,306)	447(416,480)	high_depr
Infections	Urinary Tract Infections	uti	255(225,287)	395(363,430)	583(546,622)	most_depr
Infections	Viral diseases (excl chronic hepatitis/HIV)	viral	125(100,153)	143(124,165)	137(121,154)	least_depr
Infections	Viral diseases (excl chronic hepatitis/HIV)	viral	137(111,167)	124(106,145)	144(128,162)	low_depr
Infections	Viral diseases (excl chronic hepatitis/HIV)	viral	124(101,152)	128(109,149)	135(119,153)	medium
Infections	Viral diseases (excl chronic hepatitis/HIV)	viral	145(121,172)	166(145,190)	166(147,186)	high_depr
Infections	Viral diseases (excl chronic hepatitis/HIV)	viral	178(154,206)	217(193,244)	183(163,206)	most_depr
Musculoskeletal	Ankylosing spondylitis	ank_spond	16(8,28)	38(27,50)	44(35,55)	least_depr
Musculoskeletal	Ankylosing spondylitis	ank_spond	24(14,39)	33(23,45)	41(32,51)	low_depr
Musculoskeletal	Ankylosing spondylitis	ank_spond	21(11,34)	30(21,41)	35(27,44)	medium
Musculoskeletal	Ankylosing spondylitis	ank_spond	36(25,51)	34(25,46)	30(22,39)	high_depr
Musculoskeletal	Ankylosing spondylitis	ank_spond	32(22,45)	39(29,51)	42(32,53)	most_depr
Musculoskeletal	Carpal tunnel syndrome	carpal_tunnel	323(283,367)	473(437,511)	556(524,590)	least_depr
Musculoskeletal	Carpal tunnel syndrome	carpal_tunnel	384(341,432)	519(481,560)	601(568,636)	low_depr
Musculoskeletal	Carpal tunnel syndrome	carpal_tunnel	401(358,448)	535(497,575)	631(596,667)	medium
Musculoskeletal	Carpal tunnel syndrome	carpal_tunnel	380(341,422)	572(533,613)	645(608,683)	high_depr
Musculoskeletal	Carpal tunnel syndrome	carpal_tunnel	451(411,493)	674(632,719)	710(670,753)	most_depr
Musculoskeletal	Collapsed vertebra	collapsed_vert	3(0,10)	11(6,18)	27(21,36)	least_depr
Musculoskeletal	Collapsed vertebra	collapsed_vert	10(4,20)	10(6,17)	30(23,39)	low_depr
Musculoskeletal	Collapsed vertebra	collapsed_vert	10(4,20)	8(4,14)	44(35,55)	medium

Musculoskeletal	Collapsed vertebra	collapsed_vert	2(0,8)	16(10,25)	36(27,46)	high_depr
Musculoskeletal	Collapsed vertebra	collapsed_vert	9(5,17)	26(18,36)	38(29,49)	most_depr
Musculoskeletal	Enteropathic arthropathy	entero_arthro	0(NaN,NaN)	1(0,4)	3(1,6)	least_depr
Musculoskeletal	Enteropathic arthropathy	entero_arthro	3(0,10)	0(NaN,NaN)	2(0,4)	low_depr
Musculoskeletal	Enteropathic arthropathy	entero_arthro	1(0,6)	1(0,4)	1(0,4)	medium
Musculoskeletal	Enteropathic arthropathy	entero_arthro	3(1,10)	1(0,5)	2(1,6)	high_depr
Musculoskeletal	Enteropathic arthropathy	entero_arthro	3(1,8)	1(0,5)	1(0,3)	most_depr
Musculoskeletal	Enthesopathies & synovial disorders	enthesopathy	2149(2042,2259)	2743(2653,2835)	3000(2924,3079)	least_depr
Musculoskeletal	Enthesopathies & synovial disorders	enthesopathy	2336(2224,2452)	2665(2576,2756)	2953(2878,3031)	low_depr
Musculoskeletal	Enthesopathies & synovial disorders	enthesopathy	2146(2043,2254)	2819(2728,2912)	2981(2903,3060)	medium
Musculoskeletal	Enthesopathies & synovial disorders	enthesopathy	2066(1973,2163)	2661(2574,2750)	2867(2788,2949)	high_depr
Musculoskeletal	Enthesopathies & synovial disorders	enthesopathy	2009(1923,2097)	2536(2452,2623)	2763(2681,2846)	most_depr
Musculoskeletal	Fibromatoses	fibromatosis	69(51,91)	167(145,191)	331(306,358)	least_depr
Musculoskeletal	Fibromatoses	fibromatosis	55(38,76)	176(153,202)	336(311,363)	low_depr
Musculoskeletal	Fibromatoses	fibromatosis	56(40,76)	181(158,207)	336(310,364)	medium
Musculoskeletal	Fibromatoses	fibromatosis	56(41,74)	160(138,184)	331(303,360)	high_depr
Musculoskeletal	Fibromatoses	fibromatosis	59(45,76)	143(123,165)	299(272,327)	most_depr
Musculoskeletal	Fracture of hip	fracture_hip	19(10,33)	32(23,43)	67(56,79)	least_depr
Musculoskeletal	Fracture of hip	fracture_hip	23(13,38)	37(27,48)	71(60,84)	low_depr
Musculoskeletal	Fracture of hip	fracture_hip	15(8,27)	35(26,47)	82(70,96)	medium
Musculoskeletal	Fracture of hip	fracture_hip	29(19,43)	46(35,59)	77(65,92)	high_depr
Musculoskeletal	Fracture of hip	fracture_hip	24(15,35)	54(42,69)	111(95,129)	most_depr
Musculoskeletal	Fracture of wrist	fracture_wrist	201(169,237)	227(202,254)	348(323,375)	least_depr
Musculoskeletal	Fracture of wrist	fracture_wrist	178(148,212)	244(218,272)	320(296,346)	low_depr

Musculoskeletal	Fracture of wrist	fracture_wrist	203(172,238)	254(228,283)	331(306,358)	medium
Musculoskeletal	Fracture of wrist	fracture_wrist	191(163,222)	252(226,280)	320(294,347)	high_depr
Musculoskeletal	Fracture of wrist	fracture_wrist	152(129,178)	244(219,272)	367(338,398)	most_depr
Musculoskeletal	Giant Cell arteritis	GCA	5(1,13)	10(5,16)	28(21,37)	least_depr
Musculoskeletal	Giant Cell arteritis	GCA	0(NaN,NaN)	7(4,14)	23(17,30)	low_depr
Musculoskeletal	Giant Cell arteritis	GCA	2(0,8)	8(4,15)	25(18,33)	medium
Musculoskeletal	Giant Cell arteritis	GCA	1(0,6)	5(2,11)	35(27,45)	high_depr
Musculoskeletal	Giant Cell arteritis	GCA	6(2,12)	15(9,23)	33(25,43)	most_depr
Musculoskeletal	Gout	gout	226(191,265)	361(327,397)	576(542,611)	least_depr
Musculoskeletal	Gout	gout	237(200,277)	378(343,415)	566(532,601)	low_depr
Musculoskeletal	Gout	gout	211(178,249)	403(367,441)	599(564,636)	medium
Musculoskeletal	Gout	gout	231(199,266)	423(386,461)	615(577,654)	high_depr
Musculoskeletal	Gout	gout	236(207,268)	408(373,444)	717(675,761)	most_depr
Musculoskeletal	Intervertebral disc disorders	intervert_disc	396(351,444)	527(488,568)	591(557,626)	least_depr
Musculoskeletal	Intervertebral disc disorders	intervert_disc	451(403,504)	512(473,553)	593(559,628)	low_depr
Musculoskeletal	Intervertebral disc disorders	intervert_disc	452(405,503)	510(472,551)	597(562,633)	medium
Musculoskeletal	Intervertebral disc disorders	intervert_disc	421(379,465)	480(443,519)	602(566,640)	high_depr
Musculoskeletal	Intervertebral disc disorders	intervert_disc	460(420,504)	513(476,553)	605(568,645)	most_depr
Musculoskeletal	Juvenile arthritis	juv_arth	0(NaN,NaN)	2(0,7)	1(0,4)	least_depr
Musculoskeletal	Juvenile arthritis	juv_arth	7(2,15)	2(0,6)	3(1,7)	low_depr
Musculoskeletal	Juvenile arthritis	juv_arth	5(1,12)	2(0,6)	2(1,5)	medium
Musculoskeletal	Juvenile arthritis	juv_arth	4(1,11)	5(2,11)	2(1,6)	high_depr
Musculoskeletal	Juvenile arthritis	juv_arth	4(1,10)	4(2,9)	1(0,4)	most_depr
Musculoskeletal	Lupus erythematosus (local and systemic)	SLE	15(8,27)	14(8,21)	21(15,28)	least_depr

Musculoskeletal	Lupus erythematosus (local and systemic)	SLE	17(9,28)	19(13,28)	21(15,28)	low_depr
Musculoskeletal	Lupus erythematosus (local and systemic)	SLE	13(7,24)	26(19,36)	29(22,38)	medium
Musculoskeletal	Lupus erythematosus (local and systemic)	SLE	20(12,32)	24(17,33)	30(22,39)	high_depr
Musculoskeletal	Lupus erythematosus (local and systemic)	SLE	34(24,46)	31(23,42)	28(21,38)	most_depr
Musculoskeletal	Osteoarthritis (excl spine)	OA	559(505,616)	1307(1246,1370)	2350(2283,2420)	least_depr
Musculoskeletal	Osteoarthritis (excl spine)	OA	612(556,673)	1309(1248,1373)	2430(2361,2499)	low_depr
Musculoskeletal	Osteoarthritis (excl spine)	OA	593(539,650)	1376(1313,1441)	2543(2472,2616)	medium
Musculoskeletal	Osteoarthritis (excl spine)	OA	592(543,645)	1457(1393,1523)	2594(2518,2671)	high_depr
Musculoskeletal	Osteoarthritis (excl spine)	OA	711(661,764)	1646(1578,1715)	2849(2766,2933)	most_depr
Musculoskeletal	Osteoporosis	osteoporosis	86(67,110)	266(240,294)	682(647,719)	least_depr
Musculoskeletal	Osteoporosis	osteoporosis	87(67,111)	307(279,338)	657(623,693)	low_depr
Musculoskeletal	Osteoporosis	osteoporosis	86(67,109)	298(271,328)	692(656,729)	medium
Musculoskeletal	Osteoporosis	osteoporosis	94(75,116)	307(279,336)	713(675,753)	high_depr
Musculoskeletal	Osteoporosis	osteoporosis	130(109,154)	399(366,433)	806(763,850)	most_depr
Musculoskeletal	Polymyalgia Rheumatica	PMR	9(4,19)	41(31,53)	133(118,151)	least_depr
Musculoskeletal	Polymyalgia Rheumatica	PMR	7(2,15)	39(29,51)	129(114,146)	low_depr
Musculoskeletal	Polymyalgia Rheumatica	PMR	3(0,10)	39(29,52)	140(124,158)	medium
Musculoskeletal	Polymyalgia Rheumatica	PMR	7(3,15)	34(25,46)	126(110,144)	high_depr
Musculoskeletal	Polymyalgia Rheumatica	PMR	8(4,16)	32(23,42)	111(95,128)	most_depr
Musculoskeletal	Postinfective and reactive arthropathies	reactive	17(8,30)	9(5,16)	12(8,18)	least_depr
Musculoskeletal	Postinfective and reactive arthropathies	reactive	9(3,20)	10(5,17)	15(10,21)	low_depr
Musculoskeletal	Postinfective and reactive arthropathies	reactive	5(1,13)	13(7,21)	13(8,19)	medium
Musculoskeletal	Postinfective and reactive arthropathies	reactive	8(3,17)	11(6,19)	12(7,19)	high_depr
Musculoskeletal	Postinfective and reactive arthropathies	reactive	12(6,21)	18(11,27)	10(5,16)	most_depr

Musculoskeletal	Psoriatic arthropathy	PSA	43(29,60)	45(34,58)	36(28,46)	least_depr
Musculoskeletal	Psoriatic arthropathy	PSA	43(29,62)	52(40,66)	39(31,49)	low_depr
Musculoskeletal	Psoriatic arthropathy	PSA	36(23,52)	44(33,57)	40(32,51)	medium
Musculoskeletal	Psoriatic arthropathy	PSA	39(27,55)	54(42,68)	38(30,49)	high_depr
Musculoskeletal	Psoriatic arthropathy	PSA	42(31,57)	59(47,74)	52(41,64)	most_depr
Musculoskeletal	Rheumatoid Arthritis	RhA	74(56,96)	115(98,135)	180(162,199)	least_depr
Musculoskeletal	Rheumatoid Arthritis	RhA	69(52,91)	121(103,141)	183(165,203)	low_depr
Musculoskeletal	Rheumatoid Arthritis	RhA	60(44,79)	150(130,172)	193(174,214)	medium
Musculoskeletal	Rheumatoid Arthritis	RhA	84(66,104)	149(130,171)	233(211,257)	high_depr
Musculoskeletal	Rheumatoid Arthritis	RhA	100(82,121)	208(185,234)	284(259,312)	most_depr
Musculoskeletal	Scoliosis	scoliosis	39(26,56)	43(33,56)	71(60,84)	least_depr
Musculoskeletal	Scoliosis	scoliosis	53(37,72)	57(45,71)	68(57,81)	low_depr
Musculoskeletal	Scoliosis	scoliosis	58(43,78)	52(40,65)	81(69,95)	medium
Musculoskeletal	Scoliosis	scoliosis	51(38,68)	60(47,74)	77(65,91)	high_depr
Musculoskeletal	Scoliosis	scoliosis	58(44,74)	66(53,81)	76(64,91)	most_depr
Musculoskeletal	Sjogren's disease	sjogren	4(1,11)	15(10,23)	19(13,25)	least_depr
Musculoskeletal	Sjogren's disease	sjogren	6(2,15)	15(9,23)	27(20,35)	low_depr
Musculoskeletal	Sjogren's disease	sjogren	9(3,18)	18(12,26)	19(14,27)	medium
Musculoskeletal	Sjogren's disease	sjogren	7(3,15)	16(10,23)	22(16,30)	high_depr
Musculoskeletal	Sjogren's disease	sjogren	10(5,18)	26(19,36)	23(16,31)	most_depr
Musculoskeletal	Spinal stenosis	spinal_stenosis	31(20,47)	75(61,92)	155(138,173)	least_depr
Musculoskeletal	Spinal stenosis	spinal_stenosis	46(31,65)	76(62,93)	173(155,192)	low_depr
Musculoskeletal	Spinal stenosis	spinal_stenosis	51(36,70)	73(59,89)	184(165,205)	medium
Musculoskeletal	Spinal stenosis	spinal_stenosis	41(29,57)	79(64,95)	171(152,192)	high_depr

Musculoskeletal	Spinal stenosis	spinal_stenosis	50(38,66)	117(99,137)	200(178,223)	most_depr
Musculoskeletal	Spondylolisthesis	spondylolisthesis	25(15,39)	42(32,55)	61(51,73)	least_depr
Musculoskeletal	Spondylolisthesis	spondylolisthesis	20(11,33)	46(35,60)	72(60,84)	low_depr
Musculoskeletal	Spondylolisthesis	spondylolisthesis	37(24,53)	36(26,48)	73(61,86)	medium
Musculoskeletal	Spondylolisthesis	spondylolisthesis	24(15,37)	38(28,50)	78(65,92)	high_depr
Musculoskeletal	Spondylolisthesis	spondylolisthesis	34(23,47)	59(47,74)	78(65,93)	most_depr
Musculoskeletal	Spondylosis	spondylosis	216(184,253)	497(459,536)	957(914,1001)	least_depr
Musculoskeletal	Spondylosis	spondylosis	251(215,291)	477(440,516)	990(947,1035)	low_depr
Musculoskeletal	Spondylosis	spondylosis	283(247,323)	563(523,605)	977(933,1022)	medium
Musculoskeletal	Spondylosis	spondylosis	286(252,323)	573(533,615)	1043(996,1092)	high_depr
Musculoskeletal	Spondylosis	spondylosis	376(339,415)	726(681,773)	1234(1180,1290)	most_depr
Musculoskeletal	Systemic sclerosis	sys_sclerosis	3(0,10)	4(1,8)	11(7,17)	least_depr
Musculoskeletal	Systemic sclerosis	sys_sclerosis	5(1,14)	9(5,16)	7(4,12)	low_depr
Musculoskeletal	Systemic sclerosis	sys_sclerosis	4(1,11)	6(3,12)	6(3,10)	medium
Musculoskeletal	Systemic sclerosis	sys_sclerosis	5(2,12)	8(4,14)	13(9,20)	high_depr
Musculoskeletal	Systemic sclerosis	sys_sclerosis	6(2,12)	6(2,11)	9(5,15)	most_depr
Neurological	Bell's palsy	bells	67(49,90)	56(44,71)	68(57,81)	least_depr
Neurological	Bell's palsy	bells	69(51,91)	67(54,83)	79(67,93)	low_depr
Neurological	Bell's palsy	bells	53(38,72)	70(57,86)	64(53,76)	medium
Neurological	Bell's palsy	bells	62(47,81)	66(53,82)	84(71,99)	high_depr
Neurological	Bell's palsy	bells	69(54,87)	90(75,108)	106(90,123)	most_depr
Neurological	Cerebral Palsy	cerebral_palsy	5(1,13)	5(2,10)	2(1,5)	least_depr
Neurological	Cerebral Palsy	cerebral_palsy	5(1,14)	2(0,7)	3(1,7)	low_depr
Neurological	Cerebral Palsy	cerebral_palsy	9(4,19)	11(6,18)	6(3,11)	medium
Neurological	Cerebral Palsy	cerebral_palsy	6(2,13)	9(4,16)	7(4,12)	high_depr
Neurological	Cerebral Palsy	cerebral_palsy	20(13,31)	18(12,27)	10(5,16)	most_depr
Neurological	Diabetic neurological complications	dm_neuro	6(2,14)	9(5,17)	29(22,38)	least_depr
Neurological	Diabetic neurological complications	dm_neuro	1(0,7)	18(11,27)	27(20,36)	low_depr

Neurological	Diabetic neurological complications	dm_neuro	7(2,16)	19(12,28)	37(29,47)	medium
Neurological	Diabetic neurological complications	dm_neuro	7(2,15)	27(18,37)	41(32,52)	high_depr
Neurological	Diabetic neurological complications	dm_neuro	18(10,28)	49(37,62)	78(64,93)	most_depr
Neurological	Disorders of autonomic nervous system	autonomic_neuro	11(5,21)	15(9,23)	28(21,37)	least_depr
Neurological	Disorders of autonomic nervous system	autonomic_neuro	11(5,21)	23(15,32)	29(22,37)	low_depr
Neurological	Disorders of autonomic nervous system	autonomic_neuro	13(6,24)	29(21,40)	29(22,38)	medium
Neurological	Disorders of autonomic nervous system	autonomic_neuro	16(9,26)	22(15,32)	31(23,41)	high_depr
Neurological	Disorders of autonomic nervous system	autonomic_neuro	21(13,32)	47(36,60)	43(33,55)	most_depr
Neurological	Epilepsy	epilepsy	108(85,135)	126(107,147)	132(116,149)	least_depr
Neurological	Epilepsy	epilepsy	123(98,151)	128(109,149)	142(126,160)	low_depr
Neurological	Epilepsy	epilepsy	130(106,159)	154(133,177)	158(141,177)	medium
Neurological	Epilepsy	epilepsy	161(136,189)	156(135,179)	154(136,173)	high_depr
Neurological	Epilepsy	epilepsy	233(205,265)	239(214,267)	221(198,246)	most_depr
Neurological	Essential tremor	essential_tremor	10(4,20)	25(17,35)	44(35,55)	least_depr
Neurological	Essential tremor	essential_tremor	23(13,37)	19(12,29)	51(42,62)	low_depr
Neurological	Essential tremor	essential_tremor	13(6,24)	22(15,32)	52(42,64)	medium
Neurological	Essential tremor	essential_tremor	22(13,34)	26(18,36)	47(37,59)	high_depr
Neurological	Essential tremor	essential_tremor	15(9,25)	27(19,38)	68(56,82)	most_depr
Neurological	Intracranial hypertension	intracranial_htn	3(0,9)	4(1,9)	1(0,3)	least_depr
Neurological	Intracranial hypertension	intracranial_htn	7(2,16)	3(1,7)	4(2,8)	low_depr
Neurological	Intracranial hypertension	intracranial_htn	7(3,16)	4(1,8)	1(0,4)	medium
Neurological	Intracranial hypertension	intracranial_htn	4(1,10)	6(3,12)	2(0,5)	high_depr
Neurological	Intracranial hypertension	intracranial_htn	6(2,12)	9(4,15)	5(2,10)	most_depr
Neurological	Migraine	migraine	1027(956,1102)	984(933,1038)	829(790,870)	least_depr

Neurological	Migraine	migraine	1091(1018,1169)	949(898,1002)	767(729,806)	low_depr
Neurological	Migraine	migraine	1073(1003,1147)	935(885,987)	788(750,829)	medium
Neurological	Migraine	migraine	1027(963,1093)	921(872,972)	803(762,845)	high_depr
Neurological	Migraine	migraine	1050(990,1114)	928(879,980)	722(681,764)	most_depr
Neurological	Motor neuron disease	MND	3(0,10)	2(0,7)	7(4,13)	least_depr
Neurological	Motor neuron disease	MND	1(0,7)	6(2,12)	9(5,14)	low_depr
Neurological	Motor neuron disease	MND	0(NaN,NaN)	5(2,10)	8(4,13)	medium
Neurological	Motor neuron disease	MND	2(0,8)	8(3,14)	10(6,16)	high_depr
Neurological	Motor neuron disease	MND	0(NaN,NaN)	11(6,19)	5(2,9)	most_depr
Neurological	Multiple sclerosis	MS	58(42,78)	65(52,80)	40(32,50)	least_depr
Neurological	Multiple sclerosis	MS	55(39,74)	53(42,67)	38(30,47)	low_depr
Neurological	Multiple sclerosis	MS	34(22,50)	66(53,82)	39(30,48)	medium
Neurological	Multiple sclerosis	MS	53(39,70)	55(43,69)	45(35,55)	high_depr
Neurological	Multiple sclerosis	MS	53(40,69)	55(44,69)	33(25,43)	most_depr
Neurological	Myasthenia gravis	myasthenia	4(1,13)	5(2,11)	4(2,8)	least_depr
Neurological	Myasthenia gravis	myasthenia	1(0,7)	10(5,17)	9(6,15)	low_depr
Neurological	Myasthenia gravis	myasthenia	3(1,10)	5(2,9)	9(6,15)	medium
Neurological	Myasthenia gravis	myasthenia	1(0,6)	3(1,8)	11(6,17)	high_depr
Neurological	Myasthenia gravis	myasthenia	6(2,12)	10(5,17)	13(8,20)	most_depr
Neurological	Parkinson's disease	Parkinsons	5(1,14)	29(20,40)	79(67,92)	least_depr
Neurological	Parkinson's disease	Parkinsons	2(0,9)	19(12,28)	70(59,83)	low_depr
Neurological	Parkinson's disease	Parkinsons	7(2,16)	17(10,26)	73(61,87)	medium
Neurological	Parkinson's disease	Parkinsons	7(3,16)	18(12,27)	69(57,83)	high_depr
Neurological	Parkinson's disease	Parkinsons	6(2,13)	30(21,41)	75(62,90)	most_depr
Neurological	Peripheral neuropathies (excl. cranial nerve, carpal tunnel syndromes)	periph_neuro	205(173,241)	282(254,312)	352(326,379)	least_depr
Neurological	Peripheral neuropathies (excl. cranial nerve, carpal tunnel syndromes)	periph_neuro	204(172,240)	286(257,317)	341(316,368)	low_depr
Neurological	Peripheral neuropathies (excl. cranial nerve, carpal tunnel syndromes)	periph_neuro	221(189,257)	306(276,337)	377(350,406)	medium
Neurological	Peripheral neuropathies (excl. cranial nerve, carpal tunnel syndromes)	periph_neuro	177(150,207)	300(271,330)	370(341,400)	high_depr
Neurological	Peripheral neuropathies (excl. cranial nerve, carpal tunnel syndromes)	periph_neuro	236(207,267)	389(357,424)	453(420,487)	most_depr

Neurological	Postviral fatigue syndrome, neurasthenia and fibromyalgia	chronic_fatigue	218(186,255)	221(197,247)	227(207,249)	least_depr
Neurological	Postviral fatigue syndrome, neurasthenia and fibromyalgia	chronic_fatigue	240(206,278)	251(226,279)	213(194,235)	low_depr
Neurological	Postviral fatigue syndrome, neurasthenia and fibromyalgia	chronic_fatigue	230(198,265)	249(224,277)	243(222,266)	medium
Neurological	Postviral fatigue syndrome, neurasthenia and fibromyalgia	chronic_fatigue	275(243,311)	293(265,322)	219(198,242)	high_depr
Neurological	Postviral fatigue syndrome, neurasthenia and fibromyalgia	chronic_fatigue	273(243,307)	304(276,335)	254(230,280)	most_depr
Neurological	Trigeminal neuralgia	trigem_neur	29(18,44)	67(54,83)	89(76,103)	least_depr
Neurological	Trigeminal neuralgia	trigem_neur	48(34,67)	67(54,82)	89(77,103)	low_depr
Neurological	Trigeminal neuralgia	trigem_neur	40(27,56)	68(54,83)	92(79,106)	medium
Neurological	Trigeminal neuralgia	trigem_neur	49(36,65)	68(55,83)	81(69,96)	high_depr
Neurological	Trigeminal neuralgia	trigem_neur	47(35,62)	76(62,92)	92(78,108)	most_depr
Perinatal	Bacterial sepsis of newborn	sepsis_newborn	--	--	--	least_depr
Perinatal	Bacterial sepsis of newborn	sepsis_newborn	--	--	--	low_depr
Perinatal	Bacterial sepsis of newborn	sepsis_newborn	--	--	--	medium
Perinatal	Bacterial sepsis of newborn	sepsis_newborn	--	--	--	high_depr
Perinatal	Bacterial sepsis of newborn	sepsis_newborn	--	--	--	most_depr
Perinatal	Congenital malformations of cardiac septa	congenital_septal	28(17,44)	21(14,31)	23(17,31)	least_depr
Perinatal	Congenital malformations of cardiac septa	congenital_septal	20(11,34)	20(13,30)	19(13,26)	low_depr
Perinatal	Congenital malformations of cardiac septa	congenital_septal	32(20,47)	29(21,40)	19(13,27)	medium
Perinatal	Congenital malformations of cardiac septa	congenital_septal	35(24,50)	26(18,36)	23(17,32)	high_depr
Perinatal	Congenital malformations of cardiac septa	congenital_septal	38(27,52)	24(17,34)	24(17,33)	most_depr

Perinatal	Down's syndrome	downs	--	--	--	least_depr
Perinatal	Down's syndrome	downs	--	--	--	low_depr
Perinatal	Down's syndrome	downs	--	--	--	medium
Perinatal	Down's syndrome	downs	--	--	--	high_depr
Perinatal	Down's syndrome	downs	--	--	--	most_depr
Perinatal	High birth weight	HBW	--	--	--	least_depr
Perinatal	High birth weight	HBW	--	--	--	low_depr
Perinatal	High birth weight	HBW	--	--	--	medium
Perinatal	High birth weight	HBW	--	--	--	high_depr
Perinatal	High birth weight	HBW	--	--	--	most_depr
Perinatal	Intrauterine hypoxia	intrauterine_hypoxia	--	--	--	least_depr
Perinatal	Intrauterine hypoxia	intrauterine_hypoxia	--	--	--	low_depr
Perinatal	Intrauterine hypoxia	intrauterine_hypoxia	--	--	--	medium
Perinatal	Intrauterine hypoxia	intrauterine_hypoxia	--	--	--	high_depr
Perinatal	Intrauterine hypoxia	intrauterine_hypoxia	--	--	--	most_depr
Perinatal	Neonatal jaundice (excl haemolytic dz of the newborn)	neo_jaundice	--	--	--	least_depr
Perinatal	Neonatal jaundice (excl haemolytic dz of the newborn)	neo_jaundice	--	--	--	low_depr
Perinatal	Neonatal jaundice (excl haemolytic dz of the newborn)	neo_jaundice	--	--	--	medium
Perinatal	Neonatal jaundice (excl haemolytic dz of the newborn)	neo_jaundice	--	--	--	high_depr
Perinatal	Neonatal jaundice (excl haemolytic dz of the newborn)	neo_jaundice	--	--	--	most_depr
Perinatal	Patent ductus arteriosus	PDA	--	--	--	least_depr
Perinatal	Patent ductus arteriosus	PDA	--	--	--	low_depr
Perinatal	Patent ductus arteriosus	PDA	--	--	--	medium
Perinatal	Patent ductus arteriosus	PDA	--	--	--	high_depr
Perinatal	Patent ductus arteriosus	PDA	--	--	--	most_depr
Perinatal	Post-term infant	post_term	--	--	--	least_depr
Perinatal	Post-term infant	post_term	--	--	--	low_depr

Perinatal	Post-term infant	post_term	--	--	--	medium
Perinatal	Post-term infant	post_term	--	--	--	high_depr
Perinatal	Post-term infant	post_term	--	--	--	most_depr
Perinatal	Prematurity	prematurity	--	--	--	least_depr
Perinatal	Prematurity	prematurity	--	--	--	low_depr
Perinatal	Prematurity	prematurity	--	--	--	medium
Perinatal	Prematurity	prematurity	--	--	--	high_depr
Perinatal	Prematurity	prematurity	--	--	--	most_depr
Perinatal	Respiratory distress of newborn	RDN	--	--	--	least_depr
Perinatal	Respiratory distress of newborn	RDN	--	--	--	low_depr
Perinatal	Respiratory distress of newborn	RDN	--	--	--	medium
Perinatal	Respiratory distress of newborn	RDN	--	--	--	high_depr
Perinatal	Respiratory distress of newborn	RDN	--	--	--	most_depr
Perinatal	Slow fetal growth or low birth weight	LBW	--	--	--	least_depr
Perinatal	Slow fetal growth or low birth weight	LBW	--	--	--	low_depr
Perinatal	Slow fetal growth or low birth weight	LBW	--	--	--	medium
Perinatal	Slow fetal growth or low birth weight	LBW	--	--	--	high_depr
Perinatal	Slow fetal growth or low birth weight	LBW	--	--	--	most_depr
Perinatal	Spina bifida	spina_bifida	17(9,29)	18(11,27)	14(9,20)	least_depr
Perinatal	Spina bifida	spina_bifida	11(5,22)	16(10,25)	13(9,19)	low_depr
Perinatal	Spina bifida	spina_bifida	21(12,34)	12(7,20)	13(8,19)	medium
Perinatal	Spina bifida	spina_bifida	18(10,30)	20(13,29)	14(9,22)	high_depr
Perinatal	Spina bifida	spina_bifida	24(15,35)	17(11,26)	14(9,21)	most_depr
Psychiatric	Alcohol Problems	alc_problems	365(322,412)	347(315,381)	359(333,387)	least_depr
Psychiatric	Alcohol Problems	alc_problems	338(296,385)	367(334,403)	351(325,379)	low_depr
Psychiatric	Alcohol Problems	alc_problems	324(284,368)	431(395,469)	416(386,446)	medium
Psychiatric	Alcohol Problems	alc_problems	391(350,435)	517(478,558)	478(445,513)	high_depr
Psychiatric	Alcohol Problems	alc_problems	581(536,630)	762(715,811)	707(665,750)	most_depr
Psychiatric	Alzheimer's disease	alzheimer	1(0,7)	7(3,14)	41(32,51)	least_depr
Psychiatric	Alzheimer's disease	alzheimer	0(NaN,NaN)	6(3,12)	36(28,46)	low_depr
Psychiatric	Alzheimer's disease	alzheimer	0(NaN,NaN)	10(5,17)	41(32,51)	medium
Psychiatric	Alzheimer's disease	alzheimer	1(0,6)	9(5,16)	38(30,49)	high_depr
Psychiatric	Alzheimer's disease	alzheimer	1(0,6)	6(3,12)	52(41,64)	most_depr

Psychiatric	Anorexia and bulimia nervosa	eating_dz	31(20,46)	18(12,27)	9(5,14)	least_depr
Psychiatric	Anorexia and bulimia nervosa	eating_dz	32(21,48)	23(16,32)	12(8,18)	low_depr
Psychiatric	Anorexia and bulimia nervosa	eating_dz	48(34,65)	16(10,23)	11(7,16)	medium
Psychiatric	Anorexia and bulimia nervosa	eating_dz	47(35,63)	15(10,23)	14(9,20)	high_depr
Psychiatric	Anorexia and bulimia nervosa	eating_dz	45(33,60)	30(22,41)	14(9,21)	most_depr
Psychiatric	Anxiety disorders	anxiety	923(855,995)	1046(991,1102)	1033(989,1079)	least_depr
Psychiatric	Anxiety disorders	anxiety	1034(962,1110)	1034(980,1091)	1070(1025,1117)	low_depr
Psychiatric	Anxiety disorders	anxiety	1040(969,1114)	1110(1054,1168)	1039(994,1086)	medium
Psychiatric	Anxiety disorders	anxiety	1046(981,1115)	1067(1012,1123)	1089(1040,1139)	high_depr
Psychiatric	Anxiety disorders	anxiety	1305(1236,1376)	1324(1263,1386)	1256(1201,1312)	most_depr
Psychiatric	Autism and Asperger's syndrome	autism	4(1,13)	1(0,5)	2(0,5)	least_depr
Psychiatric	Autism and Asperger's syndrome	autism	3(0,11)	0(NaN,NaN)	1(0,4)	low_depr
Psychiatric	Autism and Asperger's syndrome	autism	3(0,11)	1(0,4)	1(0,3)	medium
Psychiatric	Autism and Asperger's syndrome	autism	7(2,15)	3(1,8)	1(0,3)	high_depr
Psychiatric	Autism and Asperger's syndrome	autism	10(5,18)	8(4,14)	3(1,8)	most_depr
Psychiatric	Bipolar affective disorder and mania	BAD	31(19,47)	30(22,41)	37(29,46)	least_depr
Psychiatric	Bipolar affective disorder and mania	BAD	21(12,35)	34(25,45)	39(30,48)	low_depr
Psychiatric	Bipolar affective disorder and mania	BAD	33(22,49)	40(30,52)	47(38,58)	medium
Psychiatric	Bipolar affective disorder and mania	BAD	50(36,67)	41(31,54)	59(48,71)	high_depr
Psychiatric	Bipolar affective disorder and mania	BAD	97(79,118)	98(82,116)	81(67,96)	most_depr
Psychiatric	Delirium, not induced by alcohol and other psychoactive substances	delirium	3(0,10)	10(5,18)	19(13,26)	least_depr
Psychiatric	Delirium, not induced by alcohol and other psychoactive substances	delirium	3(0,10)	7(3,14)	17(12,24)	low_depr
Psychiatric	Delirium, not induced by alcohol and other	delirium	1(0,8)	7(3,13)	23(17,32)	medium

	psychoactive substances					
Psychiatric	Delirium, not induced by alcohol and other psychoactive substances	delirium	6(2,13)	7(3,14)	21(15,29)	high_depr
Psychiatric	Delirium, not induced by alcohol and other psychoactive substances	delirium	8(3,16)	16(10,24)	48(37,60)	most_depr
Psychiatric	Dementia	dementia	4(1,11)	18(11,27)	79(67,92)	least_depr
Psychiatric	Dementia	dementia	2(0,9)	14(8,22)	78(66,91)	low_depr
Psychiatric	Dementia	dementia	4(1,12)	22(15,32)	97(84,113)	medium
Psychiatric	Dementia	dementia	7(2,15)	22(15,32)	105(90,121)	high_depr
Psychiatric	Dementia	dementia	16(9,25)	47(36,60)	148(130,169)	most_depr
Psychiatric	Dementia (excluding Alzheimer's)	dementia_ex_Alz	4(1,11)	16(10,25)	74(62,87)	least_depr
Psychiatric	Dementia (excluding Alzheimer's)	dementia_ex_Alz	2(0,9)	13(8,22)	73(61,86)	low_depr
Psychiatric	Dementia (excluding Alzheimer's)	dementia_ex_Alz	4(1,12)	22(15,32)	95(81,110)	medium
Psychiatric	Dementia (excluding Alzheimer's)	dementia_ex_Alz	7(2,15)	22(14,32)	97(83,113)	high_depr
Psychiatric	Dementia (excluding Alzheimer's)	dementia_ex_Alz	16(9,25)	45(34,57)	139(121,159)	most_depr
Psychiatric	Depression	depression	1122(1047,1201)	1244(1185,1305)	1145(1098,1193)	least_depr
Psychiatric	Depression	depression	1238(1159,1322)	1255(1196,1317)	1155(1109,1204)	low_depr
Psychiatric	Depression	depression	1456(1373,1543)	1390(1327,1455)	1245(1196,1297)	medium
Psychiatric	Depression	depression	1453(1376,1534)	1480(1417,1546)	1363(1309,1419)	high_depr
Psychiatric	Depression	depression	2011(1926,2099)	1968(1894,2043)	1704(1641,1769)	most_depr
Psychiatric	Hyperkinetic disorders	ADHD	3(0,10)	0(NaN,NaN)	1(0,4)	least_depr
Psychiatric	Hyperkinetic disorders	ADHD	0(NaN,NaN)	2(0,7)	1(0,3)	low_depr
Psychiatric	Hyperkinetic disorders	ADHD	0(NaN,NaN)	1(0,5)	1(0,4)	medium
Psychiatric	Hyperkinetic disorders	ADHD	5(1,12)	1(0,4)	1(0,3)	high_depr
Psychiatric	Hyperkinetic disorders	ADHD	1(0,5)	0(NaN,NaN)	1(0,4)	most_depr
Psychiatric	Intellectual disability	intell_dz	3(0,10)	2(0,7)	1(0,4)	least_depr
Psychiatric	Intellectual disability	intell_dz	9(3,19)	3(1,9)	1(0,4)	low_depr
Psychiatric	Intellectual disability	intell_dz	6(2,14)	7(3,13)	3(1,6)	medium
Psychiatric	Intellectual disability	intell_dz	13(6,23)	10(5,17)	4(2,9)	high_depr
Psychiatric	Intellectual disability	intell_dz	46(34,61)	38(28,50)	14(8,21)	most_depr

Psychiatric	Obsessive-compulsive disorder	ocd	24(14,38)	28(20,39)	21(15,28)	least_depr
Psychiatric	Obsessive-compulsive disorder	ocd	34(22,51)	26(18,36)	19(13,26)	low_depr
Psychiatric	Obsessive-compulsive disorder	ocd	21(12,34)	25(17,35)	21(15,29)	medium
Psychiatric	Obsessive-compulsive disorder	ocd	36(25,51)	30(21,41)	20(14,28)	high_depr
Psychiatric	Obsessive-compulsive disorder	ocd	52(39,68)	41(31,53)	23(16,32)	most_depr
Psychiatric	Other psychoactive substance misuse	substance_misuse	25(15,40)	20(13,29)	25(19,33)	least_depr
Psychiatric	Other psychoactive substance misuse	substance_misuse	21(12,35)	29(20,40)	24(18,32)	low_depr
Psychiatric	Other psychoactive substance misuse	substance_misuse	62(45,83)	35(26,47)	35(27,44)	medium
Psychiatric	Other psychoactive substance misuse	substance_misuse	59(44,78)	63(50,78)	45(35,56)	high_depr
Psychiatric	Other psychoactive substance misuse	substance_misuse	197(171,226)	122(103,142)	85(72,101)	most_depr
Psychiatric	Personality disorders	PD	17(9,29)	18(12,28)	16(10,22)	least_depr
Psychiatric	Personality disorders	PD	13(6,24)	16(10,25)	14(9,20)	low_depr
Psychiatric	Personality disorders	PD	18(10,31)	35(26,47)	21(15,29)	medium
Psychiatric	Personality disorders	PD	37(25,52)	30(21,41)	24(17,32)	high_depr
Psychiatric	Personality disorders	PD	105(86,127)	71(57,87)	63(51,77)	most_depr
Psychiatric	Schizophrenia, schizotypal and delusional disorders	schizo	15(7,26)	10(5,17)	19(14,27)	least_depr
Psychiatric	Schizophrenia, schizotypal and delusional disorders	schizo	11(5,22)	18(11,26)	25(18,33)	low_depr
Psychiatric	Schizophrenia, schizotypal and delusional disorders	schizo	28(17,43)	30(22,42)	23(17,31)	medium
Psychiatric	Schizophrenia, schizotypal and delusional disorders	schizo	56(42,75)	38(28,51)	45(35,56)	high_depr
Psychiatric	Schizophrenia, schizotypal and delusional disorders	schizo	156(132,182)	126(107,146)	85(71,100)	most_depr
Respiratory	Allergic and chronic rhinitis	allergic_rhinitis	1419(1333,1509)	1398(1334,1464)	1323(1272,1375)	least_depr
Respiratory	Allergic and chronic rhinitis	allergic_rhinitis	1390(1304,1480)	1368(1305,1434)	1352(1301,1404)	low_depr
Respiratory	Allergic and chronic rhinitis	allergic_rhinitis	1333(1253,1418)	1345(1283,1409)	1260(1210,1312)	medium
Respiratory	Allergic and chronic rhinitis	allergic_rhinitis	1393(1317,1472)	1274(1214,1336)	1266(1214,1321)	high_depr

Respiratory	Allergic and chronic rhinitis	allergic_rhinitis	1318(1249,1389)	1224(1166,1284)	1271(1216,1328)	most_depr
Respiratory	Asbestosis	asbestosis	0(NaN,NaN)	3(1,8)	13(8,20)	least_depr
Respiratory	Asbestosis	asbestosis	0(NaN,NaN)	2(0,7)	9(5,15)	low_depr
Respiratory	Asbestosis	asbestosis	0(NaN,NaN)	3(1,8)	10(6,16)	medium
Respiratory	Asbestosis	asbestosis	0(NaN,NaN)	0(NaN,NaN)	15(9,22)	high_depr
Respiratory	Asbestosis	asbestosis	1(0,5)	3(1,8)	31(23,41)	most_depr
Respiratory	Aspiration pneumonitis	aspiration_pneumo	6(2,15)	6(3,13)	16(10,22)	least_depr
Respiratory	Aspiration pneumonitis	aspiration_pneumo	6(2,15)	11(6,18)	16(11,23)	low_depr
Respiratory	Aspiration pneumonitis	aspiration_pneumo	5(1,13)	15(9,23)	16(11,23)	medium
Respiratory	Aspiration pneumonitis	aspiration_pneumo	5(1,12)	12(7,20)	23(16,31)	high_depr
Respiratory	Aspiration pneumonitis	aspiration_pneumo	10(5,18)	22(14,31)	38(29,49)	most_depr
Respiratory	Asthma	asthma	1290(1209,1376)	1136(1079,1196)	1096(1050,1144)	least_depr
Respiratory	Asthma	asthma	1267(1185,1353)	1169(1110,1229)	1136(1089,1184)	low_depr
Respiratory	Asthma	asthma	1263(1184,1345)	1167(1109,1227)	1120(1073,1169)	medium
Respiratory	Asthma	asthma	1325(1251,1403)	1252(1193,1313)	1248(1196,1302)	high_depr
Respiratory	Asthma	asthma	1465(1393,1541)	1400(1338,1465)	1453(1394,1513)	most_depr
Respiratory	Bronchiectasis	bronchiectasis	25(15,39)	49(38,62)	115(101,131)	least_depr
Respiratory	Bronchiectasis	bronchiectasis	22(13,36)	49(38,62)	135(119,152)	low_depr
Respiratory	Bronchiectasis	bronchiectasis	21(12,34)	55(43,69)	137(121,155)	medium
Respiratory	Bronchiectasis	bronchiectasis	21(13,34)	57(45,71)	122(106,139)	high_depr
Respiratory	Bronchiectasis	bronchiectasis	37(26,50)	65(52,80)	164(144,185)	most_depr
Respiratory	Chronic sinusitis	sinusitis	1233(1154,1316)	1353(1292,1417)	1315(1264,1366)	least_depr
Respiratory	Chronic sinusitis	sinusitis	1333(1250,1420)	1286(1226,1349)	1325(1275,1377)	low_depr
Respiratory	Chronic sinusitis	sinusitis	1224(1147,1304)	1321(1260,1384)	1274(1224,1325)	medium
Respiratory	Chronic sinusitis	sinusitis	1208(1138,1282)	1239(1181,1300)	1202(1151,1255)	high_depr
Respiratory	Chronic sinusitis	sinusitis	1146(1082,1213)	1142(1087,1200)	1205(1151,1260)	most_depr
Respiratory	COPD	COPD	32(20,48)	109(92,129)	298(274,323)	least_depr
Respiratory	COPD	COPD	45(30,63)	129(110,150)	372(345,400)	low_depr
Respiratory	COPD	COPD	56(40,76)	168(146,192)	448(418,479)	medium
Respiratory	COPD	COPD	81(63,102)	277(249,307)	611(574,649)	high_depr

Respiratory	COPD	COPD	193(167,222)	538(499,579)	1058(1007,1110)	most_depr
Respiratory	COPD_excl_bronchitis_NOS	COPD_excl_bronchitis_NOS	25(15,40)	104(87,124)	288(264,313)	least_depr
Respiratory	COPD_excl_bronchitis_NOS	COPD_excl_bronchitis_NOS	38(25,56)	126(108,148)	361(334,388)	low_depr
Respiratory	COPD_excl_bronchitis_NOS	COPD_excl_bronchitis_NOS	52(37,72)	164(142,187)	439(409,470)	medium
Respiratory	COPD_excl_bronchitis_NOS	COPD_excl_bronchitis_NOS	73(56,93)	264(237,293)	595(558,633)	high_depr
Respiratory	COPD_excl_bronchitis_NOS	COPD_excl_bronchitis_NOS	186(160,214)	524(486,564)	1036(986,1087)	most_depr
Respiratory	Hypertrophy of nasal turbinates	hyper_nasal_turbs	46(32,65)	39(29,51)	34(27,44)	least_depr
Respiratory	Hypertrophy of nasal turbinates	hyper_nasal_turbs	51(35,70)	49(38,64)	44(35,55)	low_depr
Respiratory	Hypertrophy of nasal turbinates	hyper_nasal_turbs	59(43,80)	47(36,61)	41(32,52)	medium
Respiratory	Hypertrophy of nasal turbinates	hyper_nasal_turbs	59(44,78)	54(42,69)	35(26,45)	high_depr
Respiratory	Hypertrophy of nasal turbinates	hyper_nasal_turbs	64(49,81)	62(50,77)	44(34,56)	most_depr
Respiratory	Nasal polyp	nasal_polyp	161(133,193)	183(160,209)	233(212,256)	least_depr
Respiratory	Nasal polyp	nasal_polyp	121(97,150)	183(160,209)	251(229,275)	low_depr
Respiratory	Nasal polyp	nasal_polyp	128(103,156)	184(161,209)	208(188,230)	medium
Respiratory	Nasal polyp	nasal_polyp	146(122,174)	168(146,193)	231(208,255)	high_depr
Respiratory	Nasal polyp	nasal_polyp	103(84,125)	144(124,166)	219(196,243)	most_depr
Respiratory	Other interstitial pulmonary diseases with fibrosis	pulm_fibrosis	6(2,15)	5(2,11)	45(36,55)	least_depr
Respiratory	Other interstitial pulmonary diseases with fibrosis	pulm_fibrosis	4(1,12)	11(6,18)	40(31,50)	low_depr
Respiratory	Other interstitial pulmonary diseases with fibrosis	pulm_fibrosis	7(2,17)	9(5,17)	44(35,54)	medium
Respiratory	Other interstitial pulmonary diseases with fibrosis	pulm_fibrosis	6(2,14)	12(6,19)	43(33,54)	high_depr
Respiratory	Other interstitial pulmonary diseases with fibrosis	pulm_fibrosis	9(4,17)	24(16,34)	50(40,63)	most_depr
Respiratory	Pleural effusion	pleural_effusion	30(19,46)	66(52,81)	134(118,152)	least_depr
Respiratory	Pleural effusion	pleural_effusion	45(30,64)	82(66,99)	174(156,194)	low_depr
Respiratory	Pleural effusion	pleural_effusion	43(30,61)	99(82,117)	163(145,183)	medium
Respiratory	Pleural effusion	pleural_effusion	45(32,62)	96(80,115)	162(143,182)	high_depr
Respiratory	Pleural effusion	pleural_effusion	63(48,80)	119(102,140)	232(208,257)	most_depr

Respiratory	Pleural plaque	pleural_plaque	0(NaN,NaN)	3(1,8)	30(23,39)	least_depr
Respiratory	Pleural plaque	pleural_plaque	2(0,9)	7(3,13)	42(33,52)	low_depr
Respiratory	Pleural plaque	pleural_plaque	1(0,6)	14(8,23)	38(30,49)	medium
Respiratory	Pleural plaque	pleural_plaque	4(1,11)	7(3,14)	48(38,60)	high_depr
Respiratory	Pleural plaque	pleural_plaque	5(2,12)	16(10,25)	60(48,73)	most_depr
Respiratory	Pneumothorax	pneumothorax	45(31,64)	44(33,57)	61(50,73)	least_depr
Respiratory	Pneumothorax	pneumothorax	37(24,55)	38(28,51)	49(39,60)	low_depr
Respiratory	Pneumothorax	pneumothorax	34(22,51)	48(36,62)	48(39,60)	medium
Respiratory	Pneumothorax	pneumothorax	48(35,65)	44(33,57)	59(48,72)	high_depr
Respiratory	Pneumothorax	pneumothorax	53(40,70)	62(49,77)	63(51,77)	most_depr
Respiratory	Pulmonary collapse (excl pneumothorax)	pulm_collapse	8(3,17)	22(15,32)	48(39,59)	least_depr
Respiratory	Pulmonary collapse (excl pneumothorax)	pulm_collapse	18(10,31)	33(24,45)	62(52,74)	low_depr
Respiratory	Pulmonary collapse (excl pneumothorax)	pulm_collapse	17(9,30)	34(24,45)	59(49,71)	medium
Respiratory	Pulmonary collapse (excl pneumothorax)	pulm_collapse	14(7,24)	40(30,53)	71(59,85)	high_depr
Respiratory	Pulmonary collapse (excl pneumothorax)	pulm_collapse	30(21,43)	58(46,73)	85(71,101)	most_depr
Respiratory	Respiratory failure	resp_failure	10(4,21)	17(11,26)	41(32,51)	least_depr
Respiratory	Respiratory failure	resp_failure	10(4,22)	25(17,36)	52(42,63)	low_depr
Respiratory	Respiratory failure	resp_failure	16(8,29)	29(20,40)	52(42,64)	medium
Respiratory	Respiratory failure	resp_failure	6(2,14)	21(14,31)	76(63,90)	high_depr
Respiratory	Respiratory failure	resp_failure	28(19,41)	74(60,90)	137(119,156)	most_depr
Respiratory	Sleep apnoea	sleep_apnoea	74(55,98)	154(133,178)	156(139,175)	least_depr
Respiratory	Sleep apnoea	sleep_apnoea	125(100,155)	164(141,189)	172(153,191)	low_depr
Respiratory	Sleep apnoea	sleep_apnoea	148(121,179)	177(154,203)	195(175,217)	medium
Respiratory	Sleep apnoea	sleep_apnoea	158(132,187)	216(191,244)	226(204,251)	high_depr
Respiratory	Sleep apnoea	sleep_apnoea	227(198,258)	296(267,327)	261(236,288)	most_depr
Skin	Acne	acne	545(493,600)	278(250,307)	121(106,138)	least_depr
Skin	Acne	acne	503(453,558)	265(237,294)	125(110,142)	low_depr
Skin	Acne	acne	487(439,538)	253(227,282)	121(106,138)	medium
Skin	Acne	acne	478(435,525)	234(209,261)	131(114,149)	high_depr
Skin	Acne	acne	450(410,492)	231(206,258)	125(108,144)	most_depr
Skin	Actinic keratosis	actinic_keratosis	139(113,169)	347(316,382)	803(763,844)	least_depr
Skin	Actinic keratosis	actinic_keratosis	116(92,145)	358(325,392)	764(725,804)	low_depr
Skin	Actinic keratosis	actinic_keratosis	108(86,134)	317(287,350)	730(691,770)	medium
Skin	Actinic keratosis	actinic_keratosis	92(73,115)	287(259,318)	635(597,674)	high_depr

Skin	Actinic keratosis	actinic_keratosis	69(54,87)	178(156,202)	508(473,545)	most_depr
Skin	Alopecia areata	alopecia_areata	36(23,53)	39(29,52)	32(24,41)	least_depr
Skin	Alopecia areata	alopecia_areata	30(18,46)	32(23,43)	27(20,35)	low_depr
Skin	Alopecia areata	alopecia_areata	37(25,54)	43(32,55)	35(28,45)	medium
Skin	Alopecia areata	alopecia_areata	61(46,80)	34(25,45)	29(21,38)	high_depr
Skin	Alopecia areata	alopecia_areata	45(33,60)	44(34,56)	37(28,48)	most_depr
Skin	Dermatitis (atopc/contact/other/unspecified)	dermatitis	1788(1692,1888)	1761(1690,1834)	1919(1858,1981)	least_depr
Skin	Dermatitis (atopc/contact/other/unspecified)	dermatitis	1712(1618,1811)	1779(1707,1853)	1908(1847,1970)	low_depr
Skin	Dermatitis (atopc/contact/other/unspecified)	dermatitis	1829(1734,1927)	1779(1707,1852)	1925(1863,1989)	medium
Skin	Dermatitis (atopc/contact/other/unspecified)	dermatitis	1698(1614,1785)	1732(1662,1804)	1903(1838,1969)	high_depr
Skin	Dermatitis (atopc/contact/other/unspecified)	dermatitis	1684(1606,1764)	1740(1670,1812)	1849(1783,1917)	most_depr
Skin	Hidradenitis suppurativa	hidradenitis	22(12,35)	13(7,21)	8(5,13)	least_depr
Skin	Hidradenitis suppurativa	hidradenitis	12(5,23)	13(7,20)	7(4,12)	low_depr
Skin	Hidradenitis suppurativa	hidradenitis	18(10,31)	18(12,27)	10(6,16)	medium
Skin	Hidradenitis suppurativa	hidradenitis	21(13,33)	19(13,27)	7(4,12)	high_depr
Skin	Hidradenitis suppurativa	hidradenitis	40(29,54)	17(11,25)	15(10,23)	most_depr
Skin	Keratitis	keratitis	60(43,81)	67(54,83)	75(63,88)	least_depr
Skin	Keratitis	keratitis	60(43,80)	67(53,83)	85(73,99)	low_depr
Skin	Keratitis	keratitis	79(60,103)	78(64,94)	83(71,97)	medium
Skin	Keratitis	keratitis	60(45,78)	71(57,87)	81(68,95)	high_depr
Skin	Keratitis	keratitis	57(43,73)	59(47,74)	87(73,103)	most_depr
Skin	Lichen planus	lichen_planus	63(46,84)	95(79,113)	132(117,149)	least_depr
Skin	Lichen planus	lichen_planus	69(52,91)	100(83,118)	146(130,164)	low_depr
Skin	Lichen planus	lichen_planus	62(46,83)	93(77,111)	140(124,158)	medium
Skin	Lichen planus	lichen_planus	63(48,82)	111(94,130)	133(117,152)	high_depr
Skin	Lichen planus	lichen_planus	69(54,87)	94(79,112)	147(129,168)	most_depr

Skin	Pilonidal cyst/sinus	pilonidal	102(79,129)	80(65,98)	63(52,75)	least_depr
Skin	Pilonidal cyst/sinus	pilonidal	107(84,135)	74(59,91)	52(42,64)	low_depr
Skin	Pilonidal cyst/sinus	pilonidal	98(76,123)	76(61,93)	54(44,66)	medium
Skin	Pilonidal cyst/sinus	pilonidal	127(105,154)	85(70,103)	62(51,75)	high_depr
Skin	Pilonidal cyst/sinus	pilonidal	106(87,128)	74(60,90)	55(44,68)	most_depr
Skin	Psoriasis	psoriasis	320(280,365)	348(317,382)	391(364,420)	least_depr
Skin	Psoriasis	psoriasis	345(303,391)	365(332,400)	396(369,425)	low_depr
Skin	Psoriasis	psoriasis	345(304,390)	340(309,374)	383(356,413)	medium
Skin	Psoriasis	psoriasis	347(309,388)	417(383,454)	416(386,448)	high_depr
Skin	Psoriasis	psoriasis	365(329,403)	408(375,444)	481(448,517)	most_depr
Skin	Rosacea	rosacea	395(351,443)	452(416,489)	432(403,462)	least_depr
Skin	Rosacea	rosacea	327(287,371)	394(361,430)	436(407,466)	low_depr
Skin	Rosacea	rosacea	329(290,372)	421(387,457)	419(391,450)	medium
Skin	Rosacea	rosacea	349(312,389)	383(351,418)	408(378,439)	high_depr
Skin	Rosacea	rosacea	299(267,334)	329(299,361)	369(340,400)	most_depr
Skin	Seborrheic dermatitis	seb_derm	603(547,663)	536(496,577)	623(589,660)	least_depr
Skin	Seborrheic dermatitis	seb_derm	578(523,638)	529(490,571)	585(552,620)	low_depr
Skin	Seborrheic dermatitis	seb_derm	553(501,608)	552(512,595)	558(525,593)	medium
Skin	Seborrheic dermatitis	seb_derm	526(480,576)	548(509,590)	559(524,596)	high_depr
Skin	Seborrheic dermatitis	seb_derm	584(538,632)	568(529,610)	539(503,576)	most_depr
Skin	Urticaria	urticaria	406(361,454)	421(387,457)	463(434,494)	least_depr
Skin	Urticaria	urticaria	410(365,459)	413(379,449)	418(390,448)	low_depr
Skin	Urticaria	urticaria	395(352,441)	415(381,451)	416(387,446)	medium
Skin	Urticaria	urticaria	362(324,403)	400(368,435)	422(392,454)	high_depr
Skin	Urticaria	urticaria	421(382,462)	398(366,433)	384(354,415)	most_depr
Skin	Vitiligo	vitiligo	41(27,58)	33(24,45)	38(30,48)	least_depr
Skin	Vitiligo	vitiligo	35(23,52)	37(28,49)	41(33,51)	low_depr
Skin	Vitiligo	vitiligo	40(27,57)	34(25,46)	38(30,48)	medium
Skin	Vitiligo	vitiligo	48(35,65)	41(30,53)	34(25,44)	high_depr
Skin	Vitiligo	vitiligo	30(20,42)	38(29,50)	30(22,40)	most_depr

Table S10. Age and sex adjusted hazard ratio of smoking association with incident disease (reference value is smoking status = never); all p < 0.0002

group	phenotype_descr	phenotype	factor	levels	HR	ci_left	ci_right	se	pvalue_sci	n	n_events	median_follow_up	IQR_follow_up
Benign neoplasm or Carcinoma in situ	Benign neoplasm of colon, rectum, anus and anal canal	benign_colon	smoking	previous	1.33	1.26	1.4	0.03	2.36E-26	149732	6630	6.9	1.43
Benign neoplasm or Carcinoma in situ	Benign neoplasm of colon, rectum, anus and anal canal	benign_colon	smoking	current	1.86	1.73	2	0.04	7.46E-62	149732	6630	6.9	1.43
Cancers	Primary Malignancy_Bladder	pri_bladder	smoking	previous	1.69	1.39	2.07	0.1	2.31E-07	153595	496	6.96	1.31
Cancers	Primary Malignancy_Bladder	pri_bladder	smoking	current	2.38	1.81	3.12	0.14	4.19E-10	153595	496	6.96	1.31
Cancers	Primary Malignancy_Kidney and Ureter	pri_kidney	smoking	previous	1.69	1.33	2.15	0.12	2.13E-05	153828	331	6.96	1.31
Cancers	Primary Malignancy_Kidney and Ureter	pri_kidney	smoking	current	2.39	1.73	3.29	0.16	9.97E-08	153828	331	6.96	1.31
Cancers	Primary Malignancy_Lung and trachea	pri_lung	smoking	previous	3.68	2.96	4.58	0.11	1.45E-31	153791	684	6.97	1.31
Cancers	Primary Malignancy_Lung and trachea	pri_lung	smoking	current	14.71	11.78	18.36	0.11	1.71E-124	153791	684	6.97	1.31
Cancers	Primary Malignancy_Oesophageal	pri_oesoph	smoking	current	2.63	1.73	3.98	0.21	5.30E-06	153982	209	6.98	1.31
Cancers	Primary Malignancy_Oesophageal	pri_oesoph	smoking	previous	1.88	1.38	2.56	0.16	6.48E-05	153982	209	6.98	1.31
Cancers	Primary Malignancy_Oro-pharyngeal	pri_oroph	smoking	current	3.71	2.58	5.35	0.19	1.90E-12	153728	216	6.98	1.31
Cancers	Primary Malignancy_Oro-pharyngeal	pri_oroph	smoking	previous	1.89	1.39	2.59	0.16	6.00E-05	153728	216	6.98	1.31
Cancers	Primary Malignancy_Other Organs	pri_other	smoking	current	1.97	1.64	2.36	0.09	3.55E-13	153204	1085	6.96	1.31
Cancers	Primary Malignancy_Other Organs	pri_other	smoking	previous	1.35	1.18	1.54	0.07	7.05E-06	153204	1085	6.96	1.31

Cancers	Primary Malignancy_Pancreatic	pri_pancr	smoking	current	2.24	1.49	3.35	0.21	9.35E-05	154034	217	6.98	1.31
Cancers	Primary Malignancy_Other Skin and subcutaneous tissue	pri_skin	smoking	current	0.73	0.65	0.83	0.06	2.18E-07	149842	4488	6.92	1.41
Cancers	Secondary Malignancy_Lymph Nodes	sec_LN	smoking	previous	1.45	1.3	1.62	0.06	3.41E-11	153395	1508	6.96	1.32
Cancers	Secondary Malignancy_Lymph Nodes	sec_LN	smoking	current	1.9	1.63	2.23	0.08	6.64E-16	153395	1508	6.96	1.32
Cancers	Secondary Malignancy_Adrenal gland	sec_adrenal	smoking	current	5.39	3.29	8.84	0.25	2.31E-11	154071	107	6.98	1.31
Cancers	Secondary Malignancy_Bone	sec_bone	smoking	current	2.1	1.7	2.6	0.11	8.79E-12	153994	756	6.98	1.31
Cancers	Secondary Malignancy_Brain, Other CNS and Intracranial	sec_brain	smoking	current	3.52	2.62	4.73	0.15	7.31E-17	154057	319	6.98	1.31
Cancers	Secondary malignancy_Liver and intrahepatic bile duct	sec_liver	smoking	current	1.76	1.43	2.15	0.1	7.40E-08	153975	865	6.98	1.31
Cancers	Secondary Malignancy_Lung	sec_lung	smoking	current	1.85	1.46	2.35	0.12	4.80E-07	154003	683	6.98	1.31
Cancers	Secondary Malignancy_Lung	sec_lung	smoking	previous	1.57	1.33	1.85	0.08	6.82E-08	154003	683	6.98	1.31
Cancers	Secondary Malignancy_Other organs	sec_other	smoking	previous	1.37	1.16	1.61	0.08	1.44E-04	153903	701	6.97	1.31
Cancers	Secondary Malignancy_Other organs	sec_other	smoking	current	1.89	1.5	2.38	0.12	5.57E-08	153903	701	6.97	1.31
Cancers	Secondary Malignancy_Pleura	sec_pleura	smoking	previous	1.75	1.31	2.35	0.15	1.82E-04	154060	218	6.98	1.31
Cancers	Secondary Malignancy_Pleura	sec_pleura	smoking	current	2.37	1.58	3.55	0.21	3.07E-05	154060	218	6.98	1.31
Cardiovascular	Abdominal aortic aneurysm	AAA	smoking	previous	2.32	1.76	3.06	0.14	2.73E-09	153866	329	6.97	1.31
Cardiovascular	Abdominal aortic aneurysm	AAA	smoking	current	5.97	4.38	8.13	0.16	9.45E-30	153866	329	6.97	1.31
Cardiovascular	Atrial fibrillation	AF	smoking	previous	1.28	1.19	1.37	0.04	5.65E-12	150562	3624	6.93	1.34
Cardiovascular	Atrial fibrillation	AF	smoking	current	1.38	1.24	1.54	0.06	7.08E-09	150562	3624	6.93	1.34
Cardiovascular	Coronary heart disease not otherwise specified	CHD_NOS	smoking	previous	1.31	1.22	1.41	0.04	3.08E-14	144817	3776	6.93	1.35

Cardiovascular	Coronary heart disease not otherwise specified	CHD_NOS	smoking	current	1.9	1.73	2.1	0.05	5.14E-39	144817	3776	6.93	1.35
Cardiovascular	Ischaemic stroke	Isch_stroke	smoking	current	2.22	1.85	2.65	0.09	5.79E-18	153345	958	6.96	1.31
Cardiovascular	Left bundle branch block	LBBB	smoking	current	1.68	1.3	2.17	0.13	8.25E-05	153851	574	6.96	1.31
Cardiovascular	Pulmonary embolism	PE	smoking	current	1.58	1.29	1.94	0.1	1.21E-05	152697	918	6.96	1.31
Cardiovascular	Stroke NOS	Stroke_NOS	smoking	current	1.99	1.48	2.68	0.15	5.20E-06	151124	362	6.96	1.31
Cardiovascular	Subarachnoid haemorrhage	Subarach	smoking	current	2.16	1.45	3.24	0.21	1.76E-04	153669	189	6.98	1.31
Cardiovascular	Transient ischaemic attack	TIA	smoking	current	1.73	1.45	2.05	0.09	5.42E-10	152422	1252	6.95	1.32
Cardiovascular	Heart failure	hf	smoking	current	2.33	2.04	2.66	0.07	1.33E-35	152718	1836	6.95	1.32
Cardiovascular	Heart failure	hf	smoking	previous	1.31	1.18	1.45	0.05	2.54E-07	152718	1836	6.95	1.32
Cardiovascular	Myocardial infarction	myocardial_infarction	smoking	current	2.52	2.07	3.07	0.1	3.95E-20	147230	709	6.96	1.31
Cardiovascular	Nonrheumatic aortic valve disorders	nonRh_aortic	smoking	current	1.61	1.3	1.98	0.11	1.03E-05	153451	881	6.96	1.32
Cardiovascular	Pericardial effusion (noninflammatory)	pericardial_effusion	smoking	current	2.22	1.53	3.2	0.19	2.26E-05	153777	249	6.98	1.31
Cardiovascular	Peripheral arterial disease	peripheral_arterial_disease	smoking	previous	1.82	1.56	2.14	0.08	1.61E-13	152668	959	6.96	1.31
Cardiovascular	Peripheral arterial disease	peripheral_arterial_disease	smoking	current	6.85	5.8	8.08	0.08	6.39E-114	152668	959	6.96	1.31

Cardiovascular	Stable angina	stable_angina	smoking	previous	1.19	1.11	1.28	0.04	3.15E-06	148093	3508	6.93	1.35
Cardiovascular	Stable angina	stable_angina	smoking	current	1.61	1.45	1.79	0.05	9.19E-20	148093	3508	6.93	1.35
Cardiovascular	Unstable Angina	unstable_angina	smoking	current	1.93	1.6	2.33	0.1	4.78E-12	152534	963	6.96	1.32
Digestive	Gastro-oesophageal reflux disease	GORD	smoking	previous	1.15	1.09	1.21	0.03	1.31E-07	141700	6892	6.88	1.43
Digestive	Inflammatory bowel disease (IBD)	IBD	smoking	previous	1.78	1.45	2.19	0.1	3.34E-08	151928	441	6.96	1.31
Digestive	Inflammatory bowel disease (IBD)	IBD	smoking	current	1.85	1.38	2.48	0.15	3.47E-05	151928	441	6.96	1.31
Digestive	Liver fibrosis, sclerosis and cirrhosis	cirrhosis	smoking	current	2.47	1.87	3.26	0.14	1.43E-10	153646	402	6.96	1.31
Digestive	Diverticular disease of intestine (acute and chronic)	diverticuli	smoking	previous	1.29	1.23	1.36	0.02	1.06E-25	148653	7761	6.88	1.44
Digestive	Diverticular disease of intestine (acute and chronic)	diverticuli	smoking	current	1.46	1.36	1.58	0.04	3.88E-24	148653	7761	6.88	1.44
Digestive	Fatty Liver	fatty_liver	smoking	previous	1.45	1.29	1.62	0.06	3.99E-10	153645	1367	6.96	1.32
Digestive	Fatty Liver	fatty_liver	smoking	current	1.42	1.2	1.69	0.09	6.24E-05	153645	1367	6.96	1.32
Digestive	Gastritis and duodenitis	gastritis_duodenitis	smoking	current	1.56	1.45	1.68	0.04	4.55E-32	144186	7105	6.89	1.44
Digestive	Gastritis and duodenitis	gastritis_duodenitis	smoking	previous	1.21	1.15	1.28	0.03	9.36E-14	144186	7105	6.89	1.44
Digestive	Diaphragmatic hernia	hernia_diaphragm	smoking	current	1.21	1.11	1.33	0.05	3.80E-05	146847	5267	6.9	1.42
Digestive	Diaphragmatic hernia	hernia_diaphragm	smoking	previous	1.14	1.08	1.21	0.03	7.23E-06	146847	5267	6.9	1.42
Digestive	Alcoholic liver disease	liver_alc	smoking	previous	1.96	1.44	2.66	0.16	1.56E-05	153782	247	6.98	1.31
Digestive	Alcoholic liver disease	liver_alc	smoking	current	4.44	3.19	6.17	0.17	9.04E-19	153782	247	6.98	1.31
Digestive	Hepatic failure	liver_fail	smoking	current	2.38	1.56	3.62	0.21	5.10E-05	153934	167	6.98	1.31

Digestive	Oesophagitis and oesophageal ulcer	oesoph_ulc	smoking	previous	1.22	1.14	1.3	0.03	2.09E-09	146523	4429	6.92	1.41
Digestive	Oesophagitis and oesophageal ulcer	oesoph_ulc	smoking	current	1.27	1.15	1.4	0.05	2.69E-06	146523	4429	6.92	1.41
Digestive	Peritonitis	peritonitis	smoking	current	2.28	1.8	2.88	0.12	5.62E-12	153043	568	6.96	1.31
Digestive	Portal hypertension	portal_htn	smoking	current	3.07	2.01	4.68	0.22	1.91E-07	154015	148	6.98	1.31
Digestive	Ulcerative colitis	ulc_colitis	smoking	previous	2.1	1.65	2.66	0.12	1.00E-09	152450	337	6.96	1.31
Digestive	Ulcerative colitis	ulc_colitis	smoking	current	2.15	1.54	3	0.17	6.80E-06	152450	337	6.96	1.31
Digestive	Peptic ulcer disease	ulcer_peptic	smoking	current	1.95	1.66	2.28	0.08	1.53E-16	149047	1368	6.95	1.32
Endocrine	Diabetes Type II	diabetes_t2	smoking	previous	1.23	1.16	1.31	0.03	1.53E-11	149744	4989	6.91	1.41
Endocrine	Diabetes Type II	diabetes_t2	smoking	current	1.57	1.44	1.71	0.04	3.89E-24	149744	4989	6.91	1.41
Endocrine	Hyperthyroidism	hyperthyroid	smoking	current	1.65	1.28	2.12	0.13	1.27E-04	151683	536	6.96	1.32
Endocrine	Hypothyroidism	hypothyroid	smoking	current	1.52	1.32	1.74	0.07	2.42E-09	144663	1962	6.95	1.33
Endocrine	Obesity	obesity	smoking	previous	1.24	1.17	1.31	0.03	3.31E-14	144148	6009	6.88	1.43
Endocrine	Obesity	obesity	smoking	current	1.26	1.16	1.36	0.04	8.24E-08	144148	6009	6.88	1.43
Endocrine	Hypo or hyperthyroidism	thyroid	smoking	current	1.44	1.26	1.66	0.07	2.08E-07	142846	1971	6.94	1.33
Eye	Visual impairment and blindness	blind	smoking	current	2.14	1.6	2.85	0.15	2.26E-07	153508	407	6.96	1.31
Eye	Diabetic ophthalmic complications	diab_eye	smoking	current	1.31	1.16	1.48	0.06	1.03E-05	151709	2959	6.94	1.34
Eye	Diabetic ophthalmic complications	diab_eye	smoking	previous	1.27	1.18	1.38	0.04	1.28E-09	151709	2959	6.94	1.34
Genitourinary	Acute Kidney Injury	AKI	smoking	current	2.2	1.91	2.52	0.07	2.68E-29	153834	1770	6.96	1.32
Genitourinary	Acute Kidney Injury	AKI	smoking	previous	1.31	1.18	1.45	0.05	4.91E-07	153834	1770	6.96	1.32

Genitourinary	Chronic Kidney Disease	CKD	smoking	previous	1.19	1.09	1.29	0.04	4.91E-05	152307	2647	6.93	1.33
Genitourinary	Chronic Kidney Disease	CKD	smoking	current	1.39	1.22	1.58	0.07	5.42E-07	152307	2647	6.93	1.33
Genitourinary	Erectile dysfunction	ED	smoking	current	1.29	1.19	1.4	0.04	1.63E-09	147822	5621	6.91	1.43
Genitourinary	Erectile dysfunction	ED	smoking	previous	1.23	1.16	1.3	0.03	2.94E-12	147822	5621	6.91	1.43
Haematological or immunological	Aplastic anaemias	aplastic	smoking	current	2.47	1.54	3.96	0.24	1.66E-04	154007	133	6.98	1.31
Haematological or immunological	Vitamin B12 deficiency anaemia	b12_def	smoking	current	2	1.68	2.38	0.09	3.56E-15	152741	1071	6.96	1.32
Haematological or immunological	Folate deficiency anaemia	folatedef	smoking	current	3.59	2.74	4.7	0.14	2.07E-20	153895	352	6.96	1.31
Haematological or immunological	Other anaemias	oth_anaemia	smoking	current	1.38	1.24	1.53	0.05	2.21E-09	148350	3670	6.93	1.34
Infections	Infection of anal and rectal regions	anorectal	smoking	current	3.06	2.12	4.4	0.19	2.15E-09	153653	189	6.98	1.31
Infections	Bacterial Diseases (excl TB)	bacterial	smoking	previous	1.18	1.12	1.25	0.03	2.47E-10	143690	6922	6.9	1.42
Infections	Bacterial Diseases (excl TB)	bacterial	smoking	current	1.6	1.49	1.72	0.04	2.52E-36	143690	6922	6.9	1.42
Infections	Infections of the digestive system	digestive	smoking	current	1.55	1.39	1.73	0.05	1.08E-15	152729	3297	6.94	1.34
Infections	Infections of the digestive system	digestive	smoking	previous	1.22	1.13	1.31	0.04	2.23E-07	152729	3297	6.94	1.34
Infections	Infection of liver	liver	smoking	current	3.14	1.93	5.12	0.25	4.30E-06	153413	114	6.98	1.31
Infections	Lower Respiratory Tract Infections	lrti	smoking	current	3.1	2.86	3.37	0.04	1.29E-157	149287	4282	6.94	1.34

Infections	Lower Respiratory Tract Infections	lrti	smoking	previous	1.51	1.41	1.62	0.03	2.25E-32	149287	4282	6.94	1.34
Infections	Mycoses	mycoses	smoking	current	2.63	2.15	3.21	0.1	3.58E-21	153774	753	6.96	1.31
Infections	Other or unspecified infectious organisms	oth_orga nisms	smoking	previous	1.34	1.27	1.4	0.02	1.78E-31	149636	8008	6.89	1.43
Infections	Other or unspecified infectious organisms	oth_orga nisms	smoking	current	2.17	2.03	2.31	0.03	8.61E-12 3	149636	8008	6.89	1.43
Infections	Infections of Other or unspecified organs	oth_orga ns	smoking	current	1.78	1.61	1.97	0.05	1.21E-29	150859	3565	6.94	1.34
Infections	Infections of Other or unspecified organs	oth_orga ns	smoking	previous	1.24	1.15	1.33	0.04	7.14E-09	150859	3565	6.94	1.34
Infections	Septicaemia	sepsis	smoking	current	1.73	1.46	2.04	0.08	1.14E-10	153580	1379	6.96	1.32
Infections	Septicaemia	sepsis	smoking	previous	1.35	1.2	1.52	0.06	3.48E-07	153580	1379	6.96	1.32
Infections	Infection of skin and subcutaneous tissues	skin	smoking	current	1.7	1.5	1.93	0.06	8.48E-17	151756	2174	6.95	1.33
Infections	Urinary Tract Infections	uti	smoking	current	1.43	1.27	1.6	0.06	2.86E-09	151487	3064	6.94	1.33
Infections	Urinary Tract Infections	uti	smoking	previous	1.22	1.13	1.32	0.04	3.45E-07	151487	3064	6.94	1.33
Infections	Viral diseases (excl chronic hepatitis/HIV)	viral	smoking	current	1.44	1.2	1.72	0.09	7.91E-05	147978	1102	6.96	1.32
Musculoskel etal	Osteoarthritis (excl spine)	OA	smoking	previous	1.15	1.11	1.2	0.02	3.42E-12	130200	11491	6.8	1.51
Musculoskel etal	Rheumatoid Arthritis	RhA	smoking	previous	1.37	1.18	1.58	0.07	2.40E-05	151660	896	6.96	1.32
Musculoskel etal	Rheumatoid Arthritis	RhA	smoking	current	2.22	1.84	2.69	0.1	3.08E-16	151660	896	6.96	1.32
Musculoskel etal	Carpal tunnel syndrome	carpal_tu nnel	smoking	current	1.32	1.17	1.48	0.06	5.79E-06	147905	2854	6.94	1.34
Musculoskel etal	Collapsed vertebra	collapsed _vert	smoking	current	2.22	1.58	3.13	0.18	5.27E-06	153793	299	6.98	1.31
Musculoskel etal	Enthesopathies & synovial disorders	enthesop athy	smoking	previous	1.14	1.1	1.18	0.02	7.52E-13	126805	13951	6.8	1.58

Musculoskeletal	Fracture of hip	fracture_hip	smoking	current	2	1.54	2.58	0.13	1.34E-07	153621	538	6.96	1.31
Musculoskeletal	Gout	gout	smoking	previous	1.31	1.21	1.42	0.04	8.33E-11	149466	2656	6.94	1.34
Musculoskeletal	Intervertebral disc disorders	intervert_disc	smoking	current	1.46	1.29	1.64	0.06	5.40E-10	146841	2710	6.94	1.34
Musculoskeletal	Intervertebral disc disorders	intervert_disc	smoking	previous	1.21	1.11	1.31	0.04	7.13E-06	146841	2710	6.94	1.34
Musculoskeletal	Osteoporosis	osteoporosis	smoking	current	1.43	1.3	1.59	0.05	3.09E-12	149639	4270	6.92	1.41
Musculoskeletal	Spinal stenosis	spinal_stenosis	smoking	current	1.71	1.44	2.03	0.09	1.35E-09	153352	1299	6.95	1.33
Musculoskeletal	Spinal stenosis	spinal_stenosis	smoking	previous	1.31	1.16	1.48	0.06	7.78E-06	153352	1299	6.95	1.33
Musculoskeletal	Spondylosis	spondylosis	smoking	previous	1.15	1.07	1.24	0.04	1.61E-04	145448	3556	6.92	1.4
Musculoskeletal	Spondylosis	spondylosis	smoking	current	1.53	1.38	1.7	0.05	1.66E-15	145448	3556	6.92	1.4
Neurological	Postviral fatigue syndrome, neurasthenia and fibromyalgia	chronic_fatigue	smoking	current	1.49	1.21	1.83	0.11	1.59E-04	151083	840	6.95	1.32
Neurological	Epilepsy	epilepsy	smoking	current	1.9	1.41	2.56	0.15	2.20E-05	151976	369	6.96	1.31
Neurological	Peripheral neuropathies (excl. cranial nerve, carpal tunnel syndromes)	periph_neuro	smoking	previous	1.23	1.12	1.34	0.05	1.13E-05	151144	2225	6.94	1.33
Neurological	Peripheral neuropathies (excl. cranial nerve, carpal tunnel syndromes)	periph_neuro	smoking	current	1.36	1.19	1.55	0.07	8.10E-06	151144	2225	6.94	1.33
Psychiatric	Bipolar affective disorder and mania	BAD	smoking	current	3.45	2.37	5.02	0.19	8.66E-11	152960	163	6.98	1.31
Psychiatric	Alcohol Problems	alc_problems	smoking	previous	1.67	1.58	1.77	0.03	7.21E-70	151939	5819	6.89	1.42
Psychiatric	Alcohol Problems	alc_problems	smoking	current	2.11	1.95	2.28	0.04	8.42E-82	151939	5819	6.89	1.42
Psychiatric	Anxiety disorders	anxiety	smoking	previous	1.2	1.12	1.3	0.04	1.18E-06	127169	3421	6.91	1.41

Psychiatric	Anxiety disorders	anxiety	smoking	current	1.59	1.43	1.76	0.05	1.28E-18	127169	3421	6.91	1.41
Psychiatric	Delirium, not induced by alcohol and other psychoactive substances	delirium	smoking	current	2.16	1.5	3.12	0.19	3.65E-05	154029	260	6.98	1.31
Psychiatric	Depression	depression	smoking	current	2.17	1.96	2.4	0.05	2.54E-50	106004	3004	7	1.34
Psychiatric	Depression	depression	smoking	previous	1.29	1.19	1.4	0.04	7.07E-10	106004	3004	7	1.34
Psychiatric	Other psychoactive substance misuse	substance_misuse	smoking	current	5.83	4.36	7.79	0.15	1.37E-32	153575	266	6.98	1.31
Respiratory	COPD	COPD	smoking	current	14.98	13.59	16.51	0.05	0.00E+00	151345	3256	6.94	1.34
Respiratory	COPD	COPD	smoking	previous	3.05	2.77	3.37	0.05	1.51E-110	151345	3256	6.94	1.34
Respiratory	COPD_excl_bronchitis_NOS	COPD_excl_bronchitis_NOS	smoking	current	15.86	14.36	17.53	0.05	0.00E+00	151407	3171	6.94	1.34
Respiratory	COPD_excl_bronchitis_NOS	COPD_excl_bronchitis_NOS	smoking	previous	3.17	2.87	3.51	0.05	5.33E-112	151407	3171	6.94	1.34
Respiratory	Aspiration pneumonitis	aspiration_pneumo	smoking	current	3.09	2.13	4.49	0.19	3.26E-09	154032	229	6.98	1.31
Respiratory	Aspiration pneumonitis	aspiration_pneumo	smoking	previous	1.82	1.35	2.45	0.15	7.62E-05	154032	229	6.98	1.31
Respiratory	Asthma	asthma	smoking	current	1.48	1.3	1.68	0.07	2.57E-09	133123	2221	6.94	1.33
Respiratory	Bronchiectasis	bronchiectasis	smoking	current	1.76	1.4	2.22	0.12	1.61E-06	153099	734	6.96	1.32
Respiratory	Bronchiectasis	bronchiectasis	smoking	previous	1.38	1.18	1.61	0.08	6.78E-05	153099	734	6.96	1.32
Respiratory	Pleural effusion	pleural_effusion	smoking	current	2.04	1.75	2.38	0.08	5.63E-20	153405	1481	6.96	1.32
Respiratory	Pleural effusion	pleural_effusion	smoking	previous	1.36	1.22	1.52	0.06	8.16E-08	153405	1481	6.96	1.32

Respiratory	Pleural plaque	pleural_plaque	smoking	previous	1.73	1.35	2.22	0.13	1.72E-05	153972	322	6.96	1.31
Respiratory	Pleural plaque	pleural_plaque	smoking	current	2.38	1.69	3.33	0.17	5.23E-07	153972	322	6.96	1.31
Respiratory	Pneumothorax	pneumothorax	smoking	current	3.48	2.47	4.9	0.18	1.09E-12	153290	230	6.98	1.31
Respiratory	Pulmonary collapse (excl pneumothorax)	pulm_collapse	smoking	current	2.24	1.77	2.84	0.12	2.60E-11	153850	578	6.96	1.31
Respiratory	Other interstitial pulmonary diseases with fibrosis	pulm_fibrosis	smoking	previous	2.16	1.68	2.78	0.13	1.60E-09	153842	330	6.96	1.31
Respiratory	Other interstitial pulmonary diseases with fibrosis	pulm_fibrosis	smoking	current	3.31	2.38	4.6	0.17	8.66E-13	153842	330	6.96	1.31
Respiratory	Respiratory failure	resp_failure	smoking	previous	1.77	1.48	2.11	0.09	2.01E-10	153944	719	6.96	1.31
Respiratory	Respiratory failure	resp_failure	smoking	current	4.76	3.92	5.79	0.1	2.91E-55	153944	719	6.96	1.31
Respiratory	Chronic sinusitis	sinusitis	smoking	previous	1.12	1.06	1.2	0.03	1.76E-04	139467	4868	6.91	1.43
Respiratory	Sleep apnoea	sleep_apnoea	smoking	current	1.45	1.22	1.71	0.08	1.30E-05	152546	1400	6.95	1.32
Respiratory	Sleep apnoea	sleep_apnoea	smoking	previous	1.45	1.29	1.63	0.06	2.53E-10	152546	1400	6.95	1.32
Skin	Actinic keratosis	actinic_keratosis	smoking	current	0.66	0.58	0.74	0.06	9.17E-12	151055	4592	6.92	1.42
Skin	Dermatitis (atopc/contact/other/unspecified)	dermatitis	smoking	previous	1.17	1.11	1.23	0.03	2.70E-09	131299	7152	6.89	1.46
Skin	Dermatitis (atopc/contact/other/unspecified)	dermatitis	smoking	current	1.17	1.08	1.26	0.04	8.70E-05	131299	7152	6.89	1.46
Skin	Psoriasis	psoriasis	smoking	current	2.04	1.74	2.38	0.08	3.32E-19	148816	1340	6.95	1.33
Skin	Psoriasis	psoriasis	smoking	previous	1.36	1.21	1.53	0.06	4.32E-07	148816	1340	6.95	1.33
Skin	Seborrheic dermatitis	seb_derm	smoking	previous	1.17	1.08	1.27	0.04	1.49E-04	144426	2845	6.94	1.35

Benign neoplasm or Carcinoma in situ	Benign neoplasm of brain and other parts of central nervous system	benign_brain	smoking	previous	0.95	0.75	1.22	0.12	6.99E-01	153561	310	6.96	1.31
Benign neoplasm or Carcinoma in situ	Benign neoplasm of brain and other parts of central nervous system	benign_brain	smoking	current	1.05	0.72	1.55	0.2	7.89E-01	153561	310	6.96	1.31
Benign neoplasm or Carcinoma in situ	Benign neoplasm of ovary	benign_ovary	smoking	previous	0.96	0.82	1.12	0.08	5.88E-01	151115	802	6.96	1.32
Benign neoplasm or Carcinoma in situ	Benign neoplasm of ovary	benign_ovary	smoking	current	1.06	0.83	1.35	0.12	6.46E-01	151115	802	6.96	1.32
Benign neoplasm or Carcinoma in situ	Benign neoplasm of stomach and duodenum	benign_stomach	smoking	previous	0.96	0.86	1.07	0.06	4.81E-01	153375	1456	6.95	1.33
Benign neoplasm or Carcinoma in situ	Benign neoplasm of stomach and duodenum	benign_stomach	smoking	current	0.68	0.55	0.85	0.11	5.00E-04	153375	1456	6.95	1.33
Benign neoplasm or Carcinoma in situ	Benign neoplasm and polyp of uterus	benign_uterus	smoking	current	0.85	0.69	1.06	0.11	1.46E-01	151265	1170	6.96	1.32
Benign neoplasm or Carcinoma in situ	Benign neoplasm and polyp of uterus	benign_uterus	smoking	previous	0.92	0.81	1.05	0.07	2.19E-01	151265	1170	6.96	1.32
Benign neoplasm or	Carcinoma in situ_cervical	cin_cervical	smoking	previous	1.02	0.93	1.12	0.05	6.51E-01	135261	2395	6.96	1.36

Carcinoma in situ													
Benign neoplasm or Carcinoma in situ	Carcinoma in situ_cervical	cin_cervical	smoking	current	0.98	0.85	1.12	0.07	7.61E-01	135261	2395	6.96	1.36
Benign neoplasm or Carcinoma in situ	Haemangioma, any site	haemangioma	smoking	current	0.88	0.69	1.12	0.12	2.97E-01	152349	877	6.96	1.32
Benign neoplasm or Carcinoma in situ	Haemangioma, any site	haemangioma	smoking	previous	1.06	0.92	1.22	0.07	4.46E-01	152349	877	6.96	1.32
Benign neoplasm or Carcinoma in situ	Leiomyoma of uterus	leiomyoma	smoking	previous	0.87	0.77	0.98	0.06	1.74E-02	146721	1558	6.95	1.33
Benign neoplasm or Carcinoma in situ	Leiomyoma of uterus	leiomyoma	smoking	current	0.77	0.65	0.93	0.09	6.22E-03	146721	1558	6.95	1.33
Cancers	Monoclonal gammopathy of undetermined significance (MGUS)	MGUS	smoking	current	1.49	0.98	2.26	0.21	5.93E-02	153949	235	6.96	1.31
Cancers	Monoclonal gammopathy of undetermined significance (MGUS)	MGUS	smoking	previous	1.05	0.79	1.39	0.14	7.46E-01	153949	235	6.96	1.31
Cancers	Non-Hodgkin Lymphoma	NHL	smoking	previous	1.17	0.97	1.43	0.1	1.03E-01	153610	493	6.96	1.31
Cancers	Non-Hodgkin Lymphoma	NHL	smoking	current	1.6	1.21	2.11	0.14	9.22E-04	153610	493	6.96	1.31
Cancers	Leukaemia	leukaemia	smoking	previous	1.13	0.89	1.43	0.12	3.21E-01	153820	317	6.96	1.31
Cancers	Leukaemia	leukaemia	smoking	current	1.13	0.77	1.66	0.2	5.29E-01	153820	317	6.96	1.31

Cancers	Multiple myeloma and malignant plasma cell neoplasms	plasmacell	smoking	current	1.41	0.91	2.19	0.23	1.27E-01	153970	198	6.98	1.31
Cancers	Multiple myeloma and malignant plasma cell neoplasms	plasmacell	smoking	previous	0.98	0.72	1.32	0.16	8.71E-01	153970	198	6.98	1.31
Cancers	Primary Malignancy_biliary tract	pri_biliary	smoking	previous	0.83	0.54	1.26	0.21	3.78E-01	154049	103	6.98	1.31
Cancers	Primary Malignancy_biliary tract	pri_biliary	smoking	current	0.91	0.45	1.85	0.36	7.99E-01	154049	103	6.98	1.31
Cancers	Primary Malignancy_colorectal and anus	pri_bowel	smoking	current	1.14	0.93	1.39	0.1	2.15E-01	153096	1198	6.96	1.32
Cancers	Primary Malignancy_colorectal and anus	pri_bowel	smoking	previous	1.23	1.09	1.38	0.06	9.98E-04	153096	1198	6.96	1.32
Cancers	Primary Malignancy_Brain, Other CNS and Intracranial	pri_brain	smoking	current	1.16	0.66	2.02	0.29	6.11E-01	153944	165	6.98	1.31
Cancers	Primary Malignancy_Brain, Other CNS and Intracranial	pri_brain	smoking	previous	1.57	1.13	2.18	0.17	6.49E-03	153944	165	6.98	1.31
Cancers	Primary Malignancy_Breast	pri_breast	smoking	previous	1.07	0.97	1.18	0.05	1.69E-01	150427	2019	6.95	1.33
Cancers	Primary Malignancy_Breast	pri_breast	smoking	current	0.97	0.83	1.15	0.08	7.58E-01	150427	2019	6.95	1.33
Cancers	Primary Malignancy_Malignant Melanoma	pri_melanoma	smoking	current	0.56	0.41	0.76	0.16	2.78E-04	152254	748	6.96	1.32
Cancers	Primary Malignancy_Malignant Melanoma	pri_melanoma	smoking	previous	0.93	0.8	1.08	0.08	3.57E-01	152254	748	6.96	1.32
Cancers	Primary Malignancy_Ovarian	pri_ovarian	smoking	previous	0.85	0.65	1.12	0.14	2.43E-01	153666	260	6.98	1.31
Cancers	Primary Malignancy_Ovarian	pri_ovarian	smoking	current	0.93	0.59	1.49	0.24	7.77E-01	153666	260	6.98	1.31
Cancers	Primary Malignancy_Pancreatic	pri_pancr	smoking	previous	1.48	1.1	1.98	0.15	8.73E-03	154034	217	6.98	1.31
Cancers	Primary Malignancy_Prostate	pri_prost	smoking	current	0.89	0.76	1.04	0.08	1.47E-01	152908	1895	6.95	1.33
Cancers	Primary Malignancy_Prostate	pri_prost	smoking	previous	0.89	0.81	0.98	0.05	2.32E-02	152908	1895	6.95	1.33

Cancers	Primary Malignancy_Other Skin and subcutaneous tissue	pri_skin	smoking	previous	0.92	0.87	0.98	0.03	1.49E-02	149842	4488	6.92	1.41
Cancers	Primary Malignancy_Stomach	pri_stomach	smoking	previous	1.46	1.06	2	0.16	1.91E-02	153976	185	6.98	1.31
Cancers	Primary Malignancy_Stomach	pri_stomach	smoking	current	1.69	1.06	2.69	0.24	2.63E-02	153976	185	6.98	1.31
Cancers	Primary Malignancy_Uterine	pri_uterine	smoking	current	0.71	0.44	1.15	0.25	1.65E-01	153595	314	6.96	1.31
Cancers	Primary Malignancy_Uterine	pri_uterine	smoking	previous	0.92	0.72	1.17	0.12	4.92E-01	153595	314	6.96	1.31
Cancers	Secondary Malignancy_Adrenal gland	sec_adrenal	smoking	previous	1.71	1.07	2.71	0.24	2.34E-02	154071	107	6.98	1.31
Cancers	Secondary Malignancy_Bone	sec_bone	smoking	previous	1.34	1.15	1.57	0.08	2.56E-04	153994	756	6.98	1.31
Cancers	Secondary Malignancy_Brain, Other CNS and Intracranial	sec_brain	smoking	previous	1.56	1.21	2	0.13	5.70E-04	154057	319	6.98	1.31
Cancers	Secondary malignancy_Liver and intrahepatic bile duct	sec_liver	smoking	previous	1.2	1.04	1.39	0.07	1.50E-02	153975	865	6.98	1.31
Cancers	Secondary Malignancy_retroperitoneum and peritoneum	sec_peritoneum	smoking	current	1.24	0.91	1.7	0.16	1.79E-01	154025	454	6.98	1.31
Cancers	Secondary Malignancy_retroperitoneum and peritoneum	sec_peritoneum	smoking	previous	1.11	0.91	1.35	0.1	3.07E-01	154025	454	6.98	1.31
Cardiovascular	Intracerebral haemorrhage	Intracereb_haem	smoking	current	1.44	0.96	2.18	0.21	8.06E-02	153816	226	6.98	1.31
Cardiovascular	Intracerebral haemorrhage	Intracereb_haem	smoking	previous	0.97	0.73	1.29	0.15	8.24E-01	153816	226	6.98	1.31
Cardiovascular	Ischaemic stroke	Isch_stroke	smoking	previous	1.11	0.97	1.28	0.07	1.36E-01	153345	958	6.96	1.31
Cardiovascular	Left bundle branch block	LBBB	smoking	previous	1.15	0.96	1.38	0.09	1.20E-01	153851	574	6.96	1.31

Cardiovascular	Pulmonary embolism	PE	smoking	previous	1.19	1.03	1.37	0.07	1.53E-02	152697	918	6.96	1.31
Cardiovascular	Right bundle branch block	RBBB	smoking	previous	1.2	1.01	1.43	0.09	3.35E-02	153748	614	6.96	1.31
Cardiovascular	Right bundle branch block	RBBB	smoking	current	1.32	1.01	1.72	0.13	3.95E-02	153748	614	6.96	1.31
Cardiovascular	Rheumatic valve dz	Rh_valve	smoking	current	1.4	0.87	2.26	0.24	1.69E-01	153877	171	6.98	1.31
Cardiovascular	Rheumatic valve dz	Rh_valve	smoking	previous	0.94	0.67	1.3	0.17	6.90E-01	153877	171	6.98	1.31
Cardiovascular	Supraventricular tachycardia	SVT	smoking	current	1.33	1	1.76	0.15	5.25E-02	153125	517	6.96	1.31
Cardiovascular	Supraventricular tachycardia	SVT	smoking	previous	1.19	0.99	1.44	0.1	6.52E-02	153125	517	6.96	1.31
Cardiovascular	Stroke NOS	Stroke_NOS	smoking	previous	0.94	0.75	1.19	0.12	6.23E-01	151124	362	6.96	1.31
Cardiovascular	Subarachnoid haemorrhage	Subarach	smoking	previous	1.21	0.88	1.67	0.16	2.47E-01	153669	189	6.98	1.31
Cardiovascular	Transient ischaemic attack	TIA	smoking	previous	1.2	1.06	1.35	0.06	3.31E-03	152422	1252	6.95	1.32
Cardiovascular	Ventricular tachycardia	VT	smoking	current	1.41	0.92	2.17	0.22	1.16E-01	153897	224	6.98	1.31
Cardiovascular	Ventricular tachycardia	VT	smoking	previous	1.3	0.98	1.73	0.15	6.90E-02	153897	224	6.98	1.31
Cardiovascular	Atrioventricular block, first degree	av_block_1	smoking	previous	0.89	0.72	1.09	0.1	2.54E-01	153969	428	6.96	1.31
Cardiovascular	Atrioventricular block, first degree	av_block_1	smoking	current	1.04	0.76	1.44	0.16	7.99E-01	153969	428	6.96	1.31
Cardiovascular	Atrioventricular block, second degree	av_block_2	smoking	previous	0.97	0.68	1.37	0.18	8.49E-01	154018	147	6.98	1.31

Cardiovascular	Atrioventricular block, second degree	av_block_2	smoking	current	1.03	0.59	1.8	0.28	9.15E-01	154018	147	6.98	1.31
Cardiovascular	Atrioventricular block, complete	av_block_3	smoking	current	1.53	0.9	2.59	0.27	1.16E-01	153996	147	6.98	1.31
Cardiovascular	Atrioventricular block, complete	av_block_3	smoking	previous	1.24	0.87	1.76	0.18	2.31E-01	153996	147	6.98	1.31
Cardiovascular	Other Cardiomyopathy	cardiomyo_oth	smoking	previous	1.47	1.15	1.87	0.12	1.81E-03	153857	312	6.96	1.31
Cardiovascular	Other Cardiomyopathy	cardiomyo_oth	smoking	current	1.42	0.98	2.07	0.19	6.46E-02	153857	312	6.96	1.31
Cardiovascular	Dilated cardiomyopathy	dcm	smoking	previous	1.75	1.28	2.39	0.16	4.65E-04	153932	183	6.98	1.31
Cardiovascular	Dilated cardiomyopathy	dcm	smoking	current	1.09	0.63	1.91	0.29	7.52E-01	153932	183	6.98	1.31
Cardiovascular	Multiple valve dz	mult_valve	smoking	current	1.21	0.9	1.64	0.15	2.11E-01	153909	518	6.96	1.31
Cardiovascular	Multiple valve dz	mult_valve	smoking	previous	1.2	1	1.44	0.09	5.62E-02	153909	518	6.96	1.31
Cardiovascular	Myocardial infarction	myocardial_infarction	smoking	previous	1.11	0.94	1.32	0.09	2.17E-01	147230	709	6.96	1.31
Cardiovascular	Nonrheumatic aortic valve disorders	nonRh_aortic	smoking	previous	1.2	1.04	1.39	0.07	1.24E-02	153451	881	6.96	1.32
Cardiovascular	Nonrheumatic mitral valve disorders	nonRh_mitral	smoking	current	1.23	0.95	1.6	0.13	1.12E-01	153359	649	6.96	1.31
Cardiovascular	Nonrheumatic mitral valve disorders	nonRh_mitral	smoking	previous	1	0.85	1.18	0.09	9.87E-01	153359	649	6.96	1.31
Cardiovascular	Pericardial effusion (noninflammatory)	pericardial_effusion	smoking	previous	1.52	1.15	2	0.14	2.86E-03	153777	249	6.98	1.31
Cardiovascular	Primary pulmonary hypertension	prim_pulm_htn	smoking	current	1.06	0.57	1.96	0.31	8.55E-01	153998	134	6.98	1.31

Cardiovascular	Primary pulmonary hypertension	prim_pulm_htn	smoking	previous	0.98	0.68	1.41	0.19	9.15E-01	153998	134	6.98	1.31
Cardiovascular	Raynaud's syndrome	raynauds	smoking	previous	1.05	0.91	1.21	0.07	5.19E-01	152528	912	6.96	1.32
Cardiovascular	Raynaud's syndrome	raynauds	smoking	current	1.06	0.85	1.33	0.11	5.87E-01	152528	912	6.96	1.32
Cardiovascular	Secondary pulmonary hypertension	sec_pulm_htn	smoking	previous	1.35	0.93	1.96	0.19	1.14E-01	154058	139	6.98	1.31
Cardiovascular	Secondary pulmonary hypertension	sec_pulm_htn	smoking	current	2.39	1.46	3.89	0.25	4.86E-04	154058	139	6.98	1.31
Cardiovascular	Sick sinus syndrome	sick_sinus	smoking	previous	0.89	0.63	1.27	0.18	5.31E-01	153979	141	6.98	1.31
Cardiovascular	Sick sinus syndrome	sick_sinus	smoking	current	0.83	0.44	1.56	0.32	5.61E-01	153979	141	6.98	1.31
Cardiovascular	Unstable Angina	unstable_angina	smoking	previous	1.2	1.04	1.38	0.07	1.17E-02	152534	963	6.96	1.32
Cardiovascular	Venous thromboembolic disease (Excl PE)	vte_ex_pe	smoking	previous	1.09	0.95	1.24	0.07	2.09E-01	150703	1091	6.96	1.32
Cardiovascular	Venous thromboembolic disease (Excl PE)	vte_ex_pe	smoking	current	1.3	1.07	1.58	0.1	7.96E-03	150703	1091	6.96	1.32
Digestive	Gastro-oesophageal reflux disease	GORD	smoking	current	1.16	1.07	1.26	0.04	2.46E-04	141700	6892	6.88	1.43
Digestive	Irritable bowel syndrome	IBS	smoking	current	1.22	1.02	1.45	0.09	3.00E-02	147371	1325	6.93	1.32
Digestive	Irritable bowel syndrome	IBS	smoking	previous	1.11	0.98	1.25	0.06	9.49E-02	147371	1325	6.93	1.32
Digestive	Anal fissure	anal_fissure	smoking	current	0.8	0.64	0.99	0.11	3.95E-02	150960	1117	6.96	1.32
Digestive	Anal fissure	anal_fissure	smoking	previous	1.12	0.99	1.28	0.06	7.30E-02	150960	1117	6.96	1.32
Digestive	Angiodysplasia of colon	angiodysplasia_colon	smoking	previous	1.3	0.9	1.9	0.19	1.66E-01	154022	131	6.98	1.31

Digestive	Angiodysplasia of colon	angiodysplasia_colon	smoking	current	1.71	0.99	2.95	0.28	5.44E-02	154022	131	6.98	1.31
Digestive	Anorectal fistula	anorectal_fistula	smoking	previous	1.22	0.92	1.62	0.14	1.65E-01	153236	247	6.96	1.31
Digestive	Anorectal fistula	anorectal_fistula	smoking	current	1.7	1.19	2.43	0.18	3.57E-03	153236	247	6.96	1.31
Digestive	Anorectal prolapse	anorectal_prolapse	smoking	current	1.24	0.87	1.79	0.19	2.37E-01	153650	340	6.96	1.31
Digestive	Anorectal prolapse	anorectal_prolapse	smoking	previous	1.03	0.82	1.3	0.12	8.01E-01	153650	340	6.96	1.31
Digestive	Appendicitis	appendicitis	smoking	previous	1.15	0.96	1.37	0.09	1.23E-01	146680	588	6.96	1.31
Digestive	Appendicitis	appendicitis	smoking	current	1.27	0.98	1.65	0.13	6.87E-02	146680	588	6.96	1.31
Digestive	Barrett's oesophagus	barretts	smoking	current	1.29	1.04	1.59	0.11	1.89E-02	153220	932	6.96	1.32
Digestive	Barrett's oesophagus	barretts	smoking	previous	1.18	1.02	1.35	0.07	2.27E-02	153220	932	6.96	1.32
Digestive	Cholangitis	cholangitis	smoking	previous	1.29	0.97	1.72	0.14	7.52E-02	153950	223	6.98	1.31
Digestive	Cholangitis	cholangitis	smoking	current	1.46	0.95	2.25	0.22	8.68E-02	153950	223	6.98	1.31
Digestive	Cholecystitis	cholecystitis	smoking	previous	1.09	0.98	1.2	0.05	1.25E-01	151776	1638	6.95	1.33
Digestive	Cholecystitis	cholecystitis	smoking	current	0.89	0.75	1.07	0.09	2.25E-01	151776	1638	6.95	1.33
Digestive	Cholelithiasis	cholelithiasis	smoking	current	1.09	0.96	1.23	0.06	1.78E-01	148499	3014	6.93	1.34
Digestive	Cholelithiasis	cholelithiasis	smoking	previous	1.08	1	1.17	0.04	4.87E-02	148499	3014	6.93	1.34
Digestive	Liver fibrosis, sclerosis and cirrhosis	cirrhosis	smoking	previous	1.48	1.19	1.85	0.11	4.98E-04	153646	402	6.96	1.31
Digestive	Coeliac disease	coeliac	smoking	previous	1.1	0.84	1.45	0.14	4.80E-01	152543	244	6.97	1.31

Digestive	Coeliac disease	coeliac	smoking	current	0.89	0.55	1.42	0.24	6.16E-01	152543	244	6.97	1.31
Digestive	Crohn's disease	crohns	smoking	previous	1.36	0.99	1.88	0.16	6.08E-02	153368	180	6.97	1.31
Digestive	Crohn's disease	crohns	smoking	current	1.79	1.16	2.78	0.22	8.81E-03	153368	180	6.97	1.31
Digestive	Abdominal Hernia	hernia_abdo	smoking	previous	1.08	1.02	1.15	0.03	1.23E-02	143859	4763	6.92	1.41
Digestive	Abdominal Hernia	hernia_abdo	smoking	current	1.06	0.97	1.17	0.05	1.95E-01	143859	4763	6.92	1.41
Digestive	Hepatic failure	liver_fail	smoking	previous	1.18	0.84	1.67	0.18	3.43E-01	153934	167	6.98	1.31
Digestive	Pancreatitis	pancreatitis	smoking	previous	1.12	0.92	1.38	0.1	2.55E-01	153343	460	6.96	1.31
Digestive	Pancreatitis	pancreatitis	smoking	current	1.66	1.26	2.18	0.14	3.53E-04	153343	460	6.96	1.31
Digestive	Peritonitis	peritonitis	smoking	previous	1.29	1.08	1.56	0.09	6.23E-03	153043	568	6.96	1.31
Digestive	Portal hypertension	portal_htn	smoking	previous	1.27	0.88	1.85	0.19	2.06E-01	154015	148	6.98	1.31
Digestive	Peptic ulcer disease	ulcer_peptic	smoking	previous	1.24	1.1	1.39	0.06	3.93E-04	149047	1368	6.95	1.32
Digestive	Oesophageal varices	varices	smoking	previous	1.26	0.91	1.76	0.17	1.61E-01	153922	173	6.98	1.31
Digestive	Oesophageal varices	varices	smoking	current	1.71	1.09	2.69	0.23	2.05E-02	153922	173	6.98	1.31
Digestive	Volvulus	volvulus	smoking	current	1.38	0.87	2.2	0.24	1.71E-01	153952	179	6.97	1.31
Digestive	Volvulus	volvulus	smoking	previous	0.88	0.63	1.22	0.17	4.51E-01	153952	179	6.97	1.31
Ear	Hearing loss	deaf	smoking	previous	1.11	1.05	1.17	0.03	2.44E-04	140976	5897	6.9	1.43
Ear	Hearing loss	deaf	smoking	current	1.03	0.94	1.13	0.05	5.23E-01	140976	5897	6.9	1.43
Ear	Meniere disease	meniere	smoking	previous	1.15	0.85	1.55	0.15	3.68E-01	153131	197	6.96	1.31
Ear	Meniere disease	meniere	smoking	current	0.82	0.47	1.4	0.28	4.62E-01	153131	197	6.96	1.31
Ear	Tinnitus	tinnitus	smoking	current	0.96	0.82	1.11	0.08	5.65E-01	137398	2177	7.04	1.31
Ear	Tinnitus	tinnitus	smoking	previous	1.14	1.04	1.25	0.05	5.75E-03	137398	2177	7.04	1.31
Endocrine	Hyperparathyroidism	PTH	smoking	current	0.81	0.55	1.21	0.2	3.12E-01	153837	399	6.96	1.31

Endocrine	Hyperparathyroidism	PTH	smoking	previous	0.97	0.78	1.2	0.11	7.49E-01	153837	399	6.96	1.31
Endocrine	Diabetes NOS	diabetes_nos	smoking	previous	1.16	0.98	1.37	0.09	7.87E-02	147594	655	6.98	1.31
Endocrine	Diabetes NOS	diabetes_nos	smoking	current	1.38	1.08	1.76	0.12	9.28E-03	147594	655	6.98	1.31
Endocrine	Diabetes Type I	diabetes_t1	smoking	previous	1.41	1.14	1.74	0.11	1.22E-03	153297	421	6.96	1.31
Endocrine	Diabetes Type I	diabetes_t1	smoking	current	1.39	1.01	1.9	0.16	4.33E-02	153297	421	6.96	1.31
Endocrine	Hyperthyroidism	hyperthyroid	smoking	previous	1.02	0.84	1.23	0.1	8.54E-01	151683	536	6.96	1.32
Endocrine	Hypothyroidism	hypothyroid	smoking	previous	1.08	0.98	1.2	0.05	1.09E-01	144663	1962	6.95	1.33
Endocrine	Hypo or hyperthyroidism	thyroid	smoking	previous	1.04	0.94	1.15	0.05	4.11E-01	142846	1971	6.94	1.33
Endocrine	Thyroiditis unspecified	thyroid_nos	smoking	previous	1.31	0.88	1.94	0.2	1.85E-01	151208	114	6.96	1.31
Endocrine	Thyroiditis unspecified	thyroid_nos	smoking	current	0.96	0.49	1.89	0.34	9.16E-01	151208	114	6.96	1.31
Eye	Anterior and Intermediate Uveitis	ant_uveitis	smoking	current	1.24	0.85	1.8	0.19	2.58E-01	153081	290	6.96	1.31
Eye	Anterior and Intermediate Uveitis	ant_uveitis	smoking	previous	1.04	0.8	1.34	0.13	7.83E-01	153081	290	6.96	1.31
Eye	Visual impairment and blindness	blind	smoking	previous	1.37	1.1	1.7	0.11	4.38E-03	153508	407	6.96	1.31
Eye	Cataract	cataract	smoking	previous	1.04	0.98	1.09	0.03	1.71E-01	148730	7052	6.9	1.43
Eye	Cataract	cataract	smoking	current	1.1	1.01	1.2	0.04	2.61E-02	148730	7052	6.9	1.43
Eye	Glaucoma	glaucoma	smoking	previous	0.88	0.79	0.97	0.05	1.23E-02	150951	1683	6.95	1.33
Eye	Glaucoma	glaucoma	smoking	current	0.97	0.82	1.15	0.09	7.13E-01	150951	1683	6.95	1.33
Eye	Macular degeneration	macula_degen	smoking	previous	1.08	0.95	1.22	0.07	2.66E-01	152253	1080	6.96	1.32

Eye	Macular degeneration	macula_degen	smoking	current	1.12	0.9	1.39	0.11	3.15E-01	152253	1080	6.96	1.32
Eye	Ptosis of eyelid	ptosis	smoking	current	1.25	0.91	1.73	0.16	1.70E-01	153571	429	6.96	1.31
Eye	Ptosis of eyelid	ptosis	smoking	previous	1.1	0.9	1.35	0.1	3.61E-01	153571	429	6.96	1.31
Eye	Retinal detachments and breaks	retinal_detach	smoking	previous	0.89	0.76	1.05	0.08	1.60E-01	152795	736	6.96	1.32
Eye	Retinal detachments and breaks	retinal_detach	smoking	current	0.72	0.54	0.95	0.14	2.00E-02	152795	736	6.96	1.32
Eye	Retinal vascular occlusions	retinal_vasc_occl	smoking	previous	1.05	0.85	1.29	0.11	6.35E-01	153633	411	6.96	1.31
Eye	Retinal vascular occlusions	retinal_vasc_occl	smoking	current	1.04	0.73	1.47	0.18	8.26E-01	153633	411	6.96	1.31
Eye	Scleritis and episcleritis	scleritis	smoking	previous	1.25	0.82	1.91	0.21	2.92E-01	153754	102	6.98	1.31
Eye	Scleritis and episcleritis	scleritis	smoking	current	1.19	0.62	2.28	0.33	6.06E-01	153754	102	6.98	1.31
Genitourinary	Hyperplasia of prostate	BPH	smoking	previous	1.05	0.98	1.12	0.03	1.52E-01	149793	3910	6.92	1.4
Genitourinary	Hyperplasia of prostate	BPH	smoking	current	0.99	0.89	1.1	0.05	8.46E-01	149793	3910	6.92	1.4
Genitourinary	End stage renal disease	ESRD	smoking	current	1.05	0.65	1.7	0.25	8.34E-01	153721	198	6.98	1.31
Genitourinary	End stage renal disease	ESRD	smoking	previous	0.98	0.72	1.32	0.15	8.69E-01	153721	198	6.98	1.31
Genitourinary	Glomerulonephritis	GN	smoking	previous	1.25	1.02	1.54	0.11	3.06E-02	153431	428	6.96	1.31
Genitourinary	Glomerulonephritis	GN	smoking	current	1.58	1.16	2.13	0.15	3.26E-03	153431	428	6.96	1.31
Genitourinary	Postcoital and contact bleeding	PCB	smoking	previous	1.07	0.84	1.37	0.12	5.78E-01	152510	336	6.96	1.31
Genitourinary	Postcoital and contact bleeding	PCB	smoking	current	0.9	0.62	1.31	0.19	5.85E-01	152510	336	6.96	1.31

Genitourinary	Postmenopausal bleeding	PMB	smoking	current	0.8	0.67	0.94	0.09	7.79E-03	148776	2149	6.95	1.33
Genitourinary	Postmenopausal bleeding	PMB	smoking	previous	1	0.91	1.1	0.05	9.47E-01	148776	2149	6.95	1.33
Genitourinary	Tubulo-interstitial nephritis	TIN	smoking	current	1.67	1.07	2.6	0.23	2.43E-02	153859	201	6.98	1.31
Genitourinary	Tubulo-interstitial nephritis	TIN	smoking	previous	1.54	1.14	2.09	0.15	4.70E-03	153859	201	6.98	1.31
Genitourinary	Non-acute cystitis	chr_cystitis	smoking	previous	1.17	0.87	1.56	0.15	3.02E-01	153788	217	6.97	1.31
Genitourinary	Non-acute cystitis	chr_cystitis	smoking	current	1.57	1.03	2.4	0.22	3.77E-02	153788	217	6.97	1.31
Genitourinary	Dysmenorrhoea	dysmenorrhoea	smoking	previous	1.2	0.86	1.68	0.17	2.88E-01	151328	183	6.98	1.31
Genitourinary	Dysmenorrhoea	dysmenorrhoea	smoking	current	1.23	0.79	1.93	0.23	3.64E-01	151328	183	6.98	1.31
Genitourinary	Endometrial hyperplasia and hypertrophy	endometrial_hyper	smoking	current	0.75	0.46	1.22	0.25	2.50E-01	153321	259	6.96	1.31
Genitourinary	Endometrial hyperplasia and hypertrophy	endometrial_hyper	smoking	previous	1.06	0.81	1.39	0.14	6.76E-01	153321	259	6.96	1.31
Genitourinary	Endometriosis	endometriosis	smoking	previous	0.92	0.71	1.19	0.13	5.13E-01	151057	320	6.96	1.32
Genitourinary	Endometriosis	endometriosis	smoking	current	1.04	0.72	1.5	0.19	8.55E-01	151057	320	6.96	1.32
Genitourinary	Female genital prolapse	female_genital_prolapse	smoking	previous	1.06	0.98	1.15	0.04	1.41E-01	148909	2832	6.94	1.35
Genitourinary	Female genital prolapse	female_genital_prolapse	smoking	current	0.79	0.68	0.92	0.08	2.66E-03	148909	2832	6.94	1.35
Genitourinary	Hydrocoele (incl infected)	hydrocele	smoking	current	1.17	0.87	1.57	0.15	2.92E-01	153146	440	6.96	1.31

Genitourinary	Hydrocoele (incl infected)	hydrocele	smoking	previous	0.98	0.8	1.21	0.1	8.81E-01	153146	440	6.96	1.31
Genitourinary	Menorrhagia and polymenorrhoea	menorrhagia	smoking	previous	0.88	0.78	0.99	0.06	3.77E-02	142650	1520	6.95	1.33
Genitourinary	Menorrhagia and polymenorrhoea	menorrhagia	smoking	current	0.72	0.6	0.87	0.09	5.44E-04	142650	1520	6.95	1.33
Genitourinary	Neuromuscular dysfunction of bladder	neuro_bladder	smoking	previous	1.08	0.95	1.22	0.06	2.63E-01	152401	1133	6.95	1.32
Genitourinary	Neuromuscular dysfunction of bladder	neuro_bladder	smoking	current	1.12	0.91	1.37	0.1	2.75E-01	152401	1133	6.95	1.32
Genitourinary	Obstructive and reflux uropathy	obstr_reflux	smoking	previous	1.21	1.04	1.41	0.08	1.50E-02	153469	776	6.96	1.31
Genitourinary	Obstructive and reflux uropathy	obstr_reflux	smoking	current	1.33	1.06	1.67	0.12	1.53E-02	153469	776	6.96	1.31
Genitourinary	Urinary Incontinence	urine_incont	smoking	previous	1.09	1.01	1.17	0.04	3.42E-02	148453	3185	6.93	1.35
Genitourinary	Urinary Incontinence	urine_incont	smoking	current	1.19	1.05	1.34	0.06	5.17E-03	148453	3185	6.93	1.35
Genitourinary	Urolithiasis	urolithiasis	smoking	previous	1.13	1.01	1.28	0.06	3.43E-02	150319	1327	6.95	1.33
Genitourinary	Urolithiasis	urolithiasis	smoking	current	1.08	0.9	1.3	0.09	3.80E-01	150319	1327	6.95	1.33
Haematological or immunological	Iron deficiency anaemia	IDA	smoking	previous	1.03	0.95	1.13	0.04	4.24E-01	150047	2610	6.94	1.34
Haematological or immunological	Iron deficiency anaemia	IDA	smoking	current	1.19	1.05	1.36	0.07	6.18E-03	150047	2610	6.94	1.34
Haematological or	Agranulocytosis	agranulocytosis	smoking	current	1.17	0.96	1.42	0.1	1.14E-01	153144	1229	6.96	1.32

immunological													
Haematological or immunological	Agranulocytosis	agranulocytosis	smoking	previous	1.2	1.06	1.35	0.06	3.61E-03	153144	1229	6.96	1.32
Haematological or immunological	Aplastic anaemias	aplastic	smoking	previous	1.21	0.82	1.78	0.2	3.38E-01	154007	133	6.98	1.31
Haematological or immunological	Vitamin B12 deficiency anaemia	b12_def	smoking	previous	1.17	1.02	1.34	0.07	2.16E-02	152741	1071	6.96	1.32
Haematological or immunological	Folate deficiency anaemia	folatedef	smoking	previous	1.4	1.1	1.8	0.13	6.79E-03	153895	352	6.96	1.31
Haematological or immunological	Other anaemias	oth_anaemia	smoking	previous	1.13	1.05	1.22	0.04	5.91E-04	148350	3670	6.93	1.34
Haematological or immunological	Primary or Idiopathic Thrombocytopaenia	pri_thrombocytopaenia	smoking	current	1.34	0.91	1.98	0.2	1.42E-01	153669	255	6.96	1.31
Haematological or immunological	Primary or Idiopathic Thrombocytopaenia	pri_thrombocytopaenia	smoking	previous	1	0.76	1.31	0.14	9.77E-01	153669	255	6.96	1.31
Haematological or immunological	Secondary or other Thrombocytopaenia	sec_oth_thrombocytopaenia	smoking	current	1.49	1.14	1.94	0.13	3.14E-03	153665	530	6.96	1.31

Haematological or immunological	Secondary or other Thrombocytopaenia	sec_oth_thrombocytopaenia	smoking	previous	1.05	0.87	1.27	0.1	5.82E-01	153665	530	6.96	1.31
Haematological or immunological	Splenomegaly	splenomegaly	smoking	previous	1.29	0.88	1.87	0.19	1.89E-01	153969	129	6.98	1.31
Haematological or immunological	Splenomegaly	splenomegaly	smoking	current	1.41	0.81	2.45	0.28	2.30E-01	153969	129	6.98	1.31
Haematological or immunological	Thrombophilia	thrombophilia	smoking	previous	1.02	0.73	1.41	0.17	9.13E-01	153732	174	6.98	1.31
Haematological or immunological	Thrombophilia	thrombophilia	smoking	current	1	0.6	1.65	0.26	9.95E-01	153732	174	6.98	1.31
Infections	Female pelvic inflammatory disease	PID	smoking	previous	1.13	0.92	1.39	0.11	2.49E-01	152924	420	6.96	1.31
Infections	Female pelvic inflammatory disease	PID	smoking	current	0.9	0.62	1.29	0.19	5.51E-01	152924	420	6.96	1.31
Infections	Infection of anal and rectal regions	anorectal	smoking	previous	1.38	0.98	1.93	0.17	6.32E-02	153653	189	6.98	1.31
Infections	Infection of bones and joints	bone	smoking	current	1.36	0.89	2.06	0.21	1.55E-01	153667	235	6.98	1.31
Infections	Infection of bones and joints	bone	smoking	previous	1.36	1.03	1.79	0.14	3.15E-02	153667	235	6.98	1.31
Infections	Ear and Upper Respiratory Tract Infections	ear_urti	smoking	previous	1.17	1.02	1.35	0.07	2.59E-02	150069	940	6.98	1.31
Infections	Ear and Upper Respiratory Tract Infections	ear_urti	smoking	current	1.34	1.09	1.64	0.1	5.42E-03	150069	940	6.98	1.31
Infections	Eye infections	eye	smoking	current	1.55	0.92	2.62	0.27	1.02E-01	153828	124	6.98	1.31
Infections	Eye infections	eye	smoking	previous	0.92	0.62	1.37	0.2	6.82E-01	153828	124	6.98	1.31
Infections	Infection of liver	liver	smoking	previous	1.53	1	2.33	0.22	4.93E-02	153413	114	6.98	1.31

Infections	Infection of male genital system	male_GU	smoking	previous	0.8	0.6	1.07	0.15	1.28E-01	153788	231	6.96	1.31
Infections	Infection of male genital system	male_GU	smoking	current	1.17	0.79	1.72	0.2	4.33E-01	153788	231	6.96	1.31
Infections	Mycoses	mycoses	smoking	previous	1.35	1.15	1.59	0.08	2.32E-04	153774	753	6.96	1.31
Infections	Other nervous system infections	oth_nerv_sys	smoking	previous	1.26	0.87	1.83	0.19	2.28E-01	153930	129	6.98	1.31
Infections	Other nervous system infections	oth_nerv_sys	smoking	current	1.29	0.72	2.31	0.3	3.92E-01	153930	129	6.98	1.31
Infections	Rheumatic fever	rh_fever	smoking	current	1.41	0.87	2.28	0.24	1.61E-01	153241	167	6.97	1.31
Infections	Rheumatic fever	rh_fever	smoking	previous	0.93	0.67	1.3	0.17	6.79E-01	153241	167	6.97	1.31
Infections	Infection of skin and subcutaneous tissues	skin	smoking	previous	1.17	1.07	1.28	0.05	9.96E-04	151756	2174	6.95	1.33
Infections	Viral diseases (excl chronic hepatitis/HIV)	viral	smoking	previous	0.99	0.87	1.13	0.07	8.61E-01	147978	1102	6.96	1.32
Musculoskeletal	Giant Cell arteritis	GCA	smoking	current	1.89	1.24	2.88	0.21	3.09E-03	153956	194	6.98	1.31
Musculoskeletal	Giant Cell arteritis	GCA	smoking	previous	0.88	0.64	1.21	0.16	4.42E-01	153956	194	6.98	1.31
Musculoskeletal	Osteoarthritis (excl spine)	OA	smoking	current	1.09	1.02	1.16	0.03	9.89E-03	130200	11491	6.8	1.51
Musculoskeletal	Polymyalgia Rheumatica	PMR	smoking	current	0.96	0.75	1.23	0.13	7.54E-01	153501	895	6.96	1.32
Musculoskeletal	Polymyalgia Rheumatica	PMR	smoking	previous	1.02	0.89	1.18	0.07	7.66E-01	153501	895	6.96	1.32
Musculoskeletal	Psoriatic arthropathy	PSA	smoking	current	1.45	0.93	2.27	0.23	9.86E-02	153520	195	6.96	1.31
Musculoskeletal	Psoriatic arthropathy	PSA	smoking	previous	1.5	1.1	2.03	0.16	9.88E-03	153520	195	6.96	1.31
Musculoskeletal	Carpal tunnel syndrome	carpal_tunnel	smoking	previous	1.16	1.07	1.25	0.04	4.36E-04	147905	2854	6.94	1.34

Musculoskeletal	Collapsed vertebra	collapsed_vert	smoking	previous	1.44	1.12	1.84	0.13	4.50E-03	153793	299	6.98	1.31
Musculoskeletal	Enthesopathies & synovial disorders	enthesopathy	smoking	current	1.09	1.03	1.15	0.03	2.83E-03	126805	13951	6.8	1.58
Musculoskeletal	Fibromatoses	fibromatosis	smoking	previous	1.18	1.06	1.31	0.05	1.56E-03	152148	1726	6.95	1.33
Musculoskeletal	Fibromatoses	fibromatosis	smoking	current	1.17	1	1.38	0.08	5.30E-02	152148	1726	6.95	1.33
Musculoskeletal	Fracture of hip	fracture_hip	smoking	previous	1.18	0.98	1.42	0.1	8.50E-02	153621	538	6.96	1.31
Musculoskeletal	Fracture of wrist	fracture_wrist	smoking	current	0.96	0.8	1.15	0.09	6.78E-01	150487	1656	6.95	1.33
Musculoskeletal	Fracture of wrist	fracture_wrist	smoking	previous	1	0.9	1.11	0.05	9.50E-01	150487	1656	6.95	1.33
Musculoskeletal	Gout	gout	smoking	current	1.05	0.92	1.2	0.07	4.85E-01	149466	2656	6.94	1.34
Musculoskeletal	Osteoporosis	osteoporosis	smoking	previous	1.07	1	1.15	0.03	3.70E-02	149639	4270	6.92	1.41
Musculoskeletal	Scoliosis	scoliosis	smoking	current	1.57	1.1	2.22	0.18	1.21E-02	153292	351	6.97	1.31
Musculoskeletal	Scoliosis	scoliosis	smoking	previous	1.4	1.12	1.76	0.11	3.16E-03	153292	351	6.97	1.31
Musculoskeletal	Sjogren's disease	sjogren	smoking	current	0.55	0.24	1.26	0.43	1.59E-01	153824	116	6.98	1.31
Musculoskeletal	Sjogren's disease	sjogren	smoking	previous	0.88	0.59	1.31	0.21	5.24E-01	153824	116	6.98	1.31
Musculoskeletal	Spondylolisthesis	spondylolisthesis	smoking	current	1.65	1.22	2.24	0.15	1.22E-03	153400	447	6.96	1.31
Musculoskeletal	Spondylolisthesis	spondylolisthesis	smoking	previous	1.37	1.12	1.67	0.1	2.24E-03	153400	447	6.96	1.31
Neurological	Multiple sclerosis	MS	smoking	current	2.27	1.45	3.57	0.23	3.55E-04	153420	132	6.98	1.31

Neurological	Multiple sclerosis	MS	smoking	previous	1.16	0.78	1.73	0.2	4.62E-01	153420	132	6.98	1.31
Neurological	Parkinson's disease	Parkinsons	smoking	previous	0.89	0.74	1.08	0.1	2.48E-01	153787	481	6.96	1.31
Neurological	Parkinson's disease	Parkinsons	smoking	current	0.44	0.28	0.69	0.23	2.94E-04	153787	481	6.96	1.31
Neurological	Disorders of autonomic nervous system	autonomic_neuro	smoking	previous	0.8	0.58	1.11	0.16	1.86E-01	153810	191	6.97	1.31
Neurological	Disorders of autonomic nervous system	autonomic_neuro	smoking	current	1.21	0.77	1.9	0.23	4.14E-01	153810	191	6.97	1.31
Neurological	Bell's palsy	bells	smoking	current	1.13	0.8	1.6	0.18	4.94E-01	153187	334	6.96	1.31
Neurological	Bell's palsy	bells	smoking	previous	0.93	0.73	1.18	0.12	5.63E-01	153187	334	6.96	1.31
Neurological	Postviral fatigue syndrome, neurasthenia and fibromyalgia	chronic_fatigue	smoking	previous	1.08	0.92	1.25	0.08	3.51E-01	151083	840	6.95	1.32
Neurological	Diabetic neurological complications	dm_neuro	smoking	current	1.67	1.11	2.53	0.21	1.47E-02	153829	221	6.98	1.31
Neurological	Diabetic neurological complications	dm_neuro	smoking	previous	1.34	1.01	1.79	0.15	4.59E-02	153829	221	6.98	1.31
Neurological	Epilepsy	epilepsy	smoking	previous	1.16	0.93	1.46	0.12	1.90E-01	151976	369	6.96	1.31
Neurological	Essential tremor	essential_tremor	smoking	previous	0.91	0.73	1.14	0.11	4.11E-01	153805	376	6.96	1.31
Neurological	Essential tremor	essential_tremor	smoking	current	1.14	0.81	1.6	0.17	4.62E-01	153805	376	6.96	1.31
Neurological	Migraine	migraine	smoking	previous	1.13	1.03	1.25	0.05	1.30E-02	140282	1926	6.94	1.34
Neurological	Migraine	migraine	smoking	current	0.94	0.8	1.1	0.08	4.51E-01	140282	1926	6.94	1.34
Neurological	Trigeminal neuralgia	trigem_neur	smoking	current	1.18	0.87	1.6	0.16	2.90E-01	153335	474	6.96	1.31
Neurological	Trigeminal neuralgia	trigem_neur	smoking	previous	0.98	0.8	1.19	0.1	8.16E-01	153335	474	6.96	1.31
Perinatal	Congenital malformations of cardiac septa	congenital_septal	smoking	previous	1.21	0.81	1.81	0.21	3.51E-01	153800	110	6.98	1.31

Perinatal	Congenital malformations of cardiac septa	congenital_septal	smoking	current	0.82	0.41	1.67	0.36	5.89E-01	153800	110	6.98	1.31
Psychiatric	Bipolar affective disorder and mania	BAD	smoking	previous	1.02	0.7	1.49	0.19	9.04E-01	152960	163	6.98	1.31
Psychiatric	Alzheimer's disease	alzheimer	smoking	current	1.13	0.79	1.63	0.18	4.97E-01	154053	390	6.96	1.31
Psychiatric	Alzheimer's disease	alzheimer	smoking	previous	0.99	0.8	1.23	0.11	9.30E-01	154053	390	6.96	1.31
Psychiatric	Delirium, not induced by alcohol and other psychoactive substances	delirium	smoking	previous	1.3	1	1.7	0.14	5.29E-02	154029	260	6.98	1.31
Psychiatric	Dementia	dementia	smoking	current	1.91	1.3	2.82	0.2	1.05E-03	153204	228	6.98	1.3
Psychiatric	Dementia	dementia	smoking	previous	0.91	0.68	1.22	0.15	5.31E-01	153204	228	6.98	1.3
Psychiatric	Dementia (excluding Alzheimer's)	dementia_ex_Alz	smoking	previous	0.95	0.67	1.36	0.18	7.85E-01	153217	146	6.98	1.31
Psychiatric	Dementia (excluding Alzheimer's)	dementia_ex_Alz	smoking	current	1.92	1.19	3.13	0.25	8.12E-03	153217	146	6.98	1.31
Psychiatric	Schizophrenia, schizotypal and delusional disorders	schizo	smoking	current	1.79	1.09	2.95	0.25	2.15E-02	153513	116	6.98	1.31
Psychiatric	Schizophrenia, schizotypal and delusional disorders	schizo	smoking	previous	0.82	0.53	1.27	0.22	3.67E-01	153513	116	6.98	1.31
Psychiatric	Other psychoactive substance misuse	substance_misuse	smoking	previous	1.44	1.06	1.96	0.16	1.93E-02	153575	266	6.98	1.31
Respiratory	Allergic and chronic rhinitis	allergic_rhinitis	smoking	current	1.09	0.97	1.23	0.06	1.62E-01	110387	2952	6.93	1.41
Respiratory	Allergic and chronic rhinitis	allergic_rhinitis	smoking	previous	1.13	1.04	1.22	0.04	2.40E-03	110387	2952	6.93	1.41
Respiratory	Asthma	asthma	smoking	previous	1.15	1.05	1.26	0.05	3.11E-03	133123	2221	6.94	1.33
Respiratory	Hypertrophy of nasal turbinates	hyper_nasal_turbs	smoking	current	0.79	0.45	1.39	0.29	4.11E-01	153544	158	6.98	1.31
Respiratory	Hypertrophy of nasal turbinates	hyper_nasal_turbs	smoking	previous	1.06	0.76	1.49	0.17	7.33E-01	153544	158	6.98	1.31
Respiratory	Nasal polyp	nasal_polyp	smoking	current	1.18	0.92	1.51	0.13	2.04E-01	151531	687	6.96	1.32

Respiratory	Nasal polyp	nasal_polyp	smoking	previous	1.28	1.09	1.5	0.08	3.15E-03	151531	687	6.96	1.32
Respiratory	Pneumothorax	pneumothorax	smoking	previous	1.44	1.07	1.95	0.15	1.72E-02	153290	230	6.98	1.31
Respiratory	Pulmonary collapse (excl pneumothorax)	pulm_collapse	smoking	previous	1.32	1.1	1.58	0.09	2.88E-03	153850	578	6.96	1.31
Respiratory	Chronic sinusitis	sinusitis	smoking	current	1.13	1.03	1.24	0.05	1.07E-02	139467	4868	6.91	1.43
Skin	Acne	acne	smoking	previous	1.24	0.94	1.63	0.14	1.23E-01	150556	248	6.98	1.31
Skin	Acne	acne	smoking	current	0.91	0.59	1.41	0.22	6.74E-01	150556	248	6.98	1.31
Skin	Actinic keratosis	actinic_keratosis	smoking	previous	1.01	0.95	1.07	0.03	7.81E-01	151055	4592	6.92	1.42
Skin	Alopecia areata	alopecia_areata	smoking	current	1.31	0.76	2.25	0.28	3.39E-01	153595	126	6.98	1.31
Skin	Alopecia areata	alopecia_areata	smoking	previous	1.05	0.71	1.56	0.2	8.04E-01	153595	126	6.98	1.31
Skin	Keratitis	keratitis	smoking	current	0.79	0.54	1.16	0.2	2.26E-01	153298	377	6.96	1.31
Skin	Keratitis	keratitis	smoking	previous	0.96	0.77	1.2	0.11	7.50E-01	153298	377	6.96	1.31
Skin	Lichen planus	lichen_planus	smoking	current	0.87	0.64	1.19	0.16	3.91E-01	152800	626	6.96	1.32
Skin	Lichen planus	lichen_planus	smoking	previous	1.33	1.13	1.57	0.08	8.08E-04	152800	626	6.96	1.32
Skin	Rosacea	rosacea	smoking	previous	1.19	1.08	1.3	0.05	2.68E-04	149906	2095	6.95	1.33
Skin	Rosacea	rosacea	smoking	current	0.8	0.67	0.94	0.08	6.27E-03	149906	2095	6.95	1.33
Skin	Seborrheic dermatitis	seb_derm	smoking	current	1.15	1.02	1.3	0.06	2.75E-02	144426	2845	6.94	1.35
Skin	Urticaria	urticaria	smoking	current	1.05	0.9	1.23	0.08	5.26E-01	149475	1874	6.95	1.34
Skin	Urticaria	urticaria	smoking	previous	1.02	0.93	1.13	0.05	6.31E-01	149475	1874	6.95	1.34
Skin	Vitiligo	vitiligo	smoking	previous	0.87	0.6	1.26	0.19	4.53E-01	153604	143	6.98	1.31
Skin	Vitiligo	vitiligo	smoking	current	1.02	0.6	1.75	0.27	9.37E-01	153604	143	6.98	1.31

Table S11. Age and sex adjusted hazard ratio of hypertension association with incident disease (reference value is no hypertension); all $p < 0.0002$

group	phenotype_descr	phenotype	factor	levels	HR	ci_left	ci_right	se	pvalue_sci	n	n_events	median_follow_up	IQR_follow_up
**Benign neoplasm or Carcinoma in situ**	Benign neoplasm of colon, rectum, anus and anal canal	benign_colon	hypertension	yes	1.35	1.28	1.42	0.03	9.39E-29	149732	6630	6.9	1.43
**Benign neoplasm or Carcinoma in situ**	Benign neoplasm of stomach and duodenum	benign_stomach	hypertension	yes	1.58	1.42	1.77	0.06	3.42E-16	153375	1456	6.95	1.33
**Cancers**	Primary Malignancy_Kidney and Ureter	pri_kidney	hypertension	yes	1.61	1.28	2.02	0.12	4.80E-05	153828	331	6.96	1.31
**Cancers**	Primary Malignancy_Lung and trachea	pri_lung	hypertension	yes	1.36	1.16	1.6	0.08	1.39E-04	153791	684	6.97	1.31
**Cardiovascular**	Abdominal aortic aneurysm	AAA	hypertension	yes	2.01	1.61	2.5	0.11	6.54E-10	153866	329	6.97	1.31
**Cardiovascular**	Atrial fibrillation	AF	hypertension	yes	1.64	1.53	1.76	0.03	1.12E-46	150562	3624	6.93	1.34
**Cardiovascular**	Coronary heart disease not	CHD_NOS	hypertension	yes	1.84	1.72	1.96	0.03	1.05E-70	144817	3776	6.93	1.35

	otherwise specified												
**Cardiovascular**	Intracerebral haemorrhage	Intracereb_haem	hypertension	yes	1.85	1.41	2.42	0.14	8.93E-06	153816	226	6.98	1.31
**Cardiovascular**	Ischaemic stroke	Isch_stroke	hypertension	yes	1.77	1.55	2.02	0.07	1.79E-17	153345	958	6.96	1.31
**Cardiovascular**	Left bundle branch block	LBBB	hypertension	yes	1.97	1.66	2.33	0.09	3.52E-15	153851	574	6.96	1.31
**Cardiovascular**	Right bundle branch block	RBBB	hypertension	yes	1.54	1.31	1.82	0.08	3.42E-07	153748	614	6.96	1.31
**Cardiovascular**	Rheumatic valve dz	Rh_valve	hypertension	yes	2.56	1.88	3.49	0.16	2.75E-09	153877	171	6.98	1.31
**Cardiovascular**	Stroke NOS	Stroke_NOS	hypertension	yes	1.75	1.42	2.17	0.11	2.55E-07	151124	362	6.96	1.31
**Cardiovascular**	Transient ischaemic attack	TIA	hypertension	yes	1.57	1.4	1.76	0.06	3.22E-14	152422	1252	6.95	1.32
**Cardiovascular**	Ventricular tachycardia	VT	hypertension	yes	1.69	1.28	2.22	0.14	1.84E-04	153897	224	6.98	1.31
**Cardiovascular**	Atrioventricular block, first degree	av_block_1	hypertension	yes	1.93	1.59	2.34	0.1	4.79E-11	153969	428	6.96	1.31
**Cardiovascular**	Other Cardiomyopathy	cardiomyo_oth	hypertension	yes	1.78	1.41	2.24	0.12	1.25E-06	153857	312	6.96	1.31
**Cardiovascular**	Heart failure	hf	hypertension	yes	2.23	2.03	2.45	0.05	8.66E-63	152718	1836	6.95	1.32

**Cardiovascular**	Multiple valve dz	mult_valve	hypertension	yes	1.73	1.45	2.07	0.09	1.47E-09	153909	518	6.96	1.31
**Cardiovascular**	Myocardial infarction	myocardial_infarction	hypertension	yes	1.42	1.21	1.67	0.08	1.57E-05	147230	709	6.96	1.31
**Cardiovascular**	Nonrheumatic aortic valve disorders	nonRh_aortic	hypertension	yes	2.08	1.81	2.38	0.07	5.89E-26	153451	881	6.96	1.32
**Cardiovascular**	Nonrheumatic mitral valve disorders	nonRh_mitral	hypertension	yes	1.6	1.36	1.88	0.08	1.33E-08	153359	649	6.96	1.31
**Cardiovascular**	Pericardial effusion (noninflammatory)	pericardial_effusion	hypertension	yes	1.83	1.4	2.38	0.13	7.58E-06	153777	249	6.98	1.31
**Cardiovascular**	Peripheral arterial disease	peripheral_arterial_disease	hypertension	yes	2.72	2.38	3.1	0.07	1.70E-50	152668	959	6.96	1.31
**Cardiovascular**	Primary pulmonary hypertension	prim_pulm_htn	hypertension	yes	2.16	1.53	3.06	0.18	1.42E-05	153998	134	6.98	1.31
**Cardiovascular**	Secondary pulmonary hypertension	sec_pulm_htn	hypertension	yes	2.18	1.55	3.07	0.18	8.89E-06	154058	139	6.98	1.31
**Cardiovascular**	Stable angina	stable_angina	hypertension	yes	1.77	1.65	1.89	0.04	5.50E-58	148093	3508	6.93	1.35
**Cardiovascular**	Unstable Angina	unstable_angina	hypertension	yes	2.19	1.92	2.5	0.07	1.51E-31	152534	963	6.96	1.32
**Digestive**	Gastro-oesophageal	GORD	hypertension	yes	1.32	1.25	1.39	0.03	1.06E-23	141700	6892	6.88	1.43

	reflux disease												
**Digestive**	Angiodysplasia of colon	angiodysplasia_colon	hypertension	yes	1.97	1.38	2.82	0.18	1.71E-04	154022	131	6.98	1.31
**Digestive**	Cholecystitis	cholecystitis	hypertension	yes	1.44	1.3	1.61	0.06	3.46E-11	151776	1638	6.95	1.33
**Digestive**	Cholelithiasis	cholelithiasis	hypertension	yes	1.42	1.31	1.54	0.04	1.69E-17	148499	3014	6.93	1.34
**Digestive**	Liver fibrosis, sclerosis and cirrhosis	cirrhosis	hypertension	yes	2.21	1.8	2.72	0.11	5.76E-14	153646	402	6.96	1.31
**Digestive**	Diverticular disease of intestine (acute and chronic)	diverticuli	hypertension	yes	1.33	1.27	1.4	0.02	4.55E-31	148653	7761	6.88	1.44
**Digestive**	Fatty Liver	fatty_liver	hypertension	yes	2.22	1.98	2.49	0.06	1.19E-42	153645	1367	6.96	1.32
**Digestive**	Gastritis and duodenitis	gastritis_duodenitis	hypertension	yes	1.38	1.31	1.45	0.03	1.90E-33	144186	7105	6.89	1.44
**Digestive**	Diaphragmatic hernia	hernia_diaphragm	hypertension	yes	1.34	1.26	1.43	0.03	1.10E-21	146847	5267	6.9	1.42
**Digestive**	Alcoholic liver disease	liver_alc	hypertension	yes	2.56	1.96	3.33	0.13	3.41E-12	153782	247	6.98	1.31
**Digestive**	Hepatic failure	liver_fail	hypertension	yes	2.12	1.54	2.92	0.16	4.01E-06	153934	167	6.98	1.31
**Digestive**	Oesophagitis and oesophageal ulcer	oesoph_ulc	hypertension	yes	1.28	1.2	1.37	0.03	4.94E-13	146523	4429	6.92	1.41

**Digestive**	Pancreatitis	pancreatitis	hypertension	yes	1.8	1.48	2.19	0.1	3.57E-09	153343	460	6.96	1.31
**Digestive**	Portal hypertension	portal_htn	hypertension	yes	2.48	1.77	3.48	0.17	1.34E-07	154015	148	6.98	1.31
**Digestive**	Peptic ulcer disease	ulcer_peptic	hypertension	yes	1.61	1.44	1.81	0.06	1.87E-16	149047	1368	6.95	1.32
**Digestive**	Oesophageal varices	varices	hypertension	yes	1.92	1.4	2.64	0.16	4.98E-05	153922	173	6.98	1.31
**Endocrine**	Hyperparathyroidism	PTH	hypertension	yes	2.99	2.43	3.66	0.1	9.96E-26	153837	399	6.96	1.31
**Endocrine**	Diabetes NOS	diabetes_nos	hypertension	yes	2.62	2.23	3.08	0.08	1.60E-31	147594	655	6.98	1.31
**Endocrine**	Diabetes Type I	diabetes_t1	hypertension	yes	3.35	2.74	4.1	0.1	6.18E-32	153297	421	6.96	1.31
**Endocrine**	Diabetes Type II	diabetes_t2	hypertension	yes	3.09	2.92	3.27	0.03	0.00E-02	149744	4989	6.91	1.41
**Endocrine**	Hyperthyroidism	hyperthyroid	hypertension	yes	1.65	1.37	2	0.1	2.55E-07	151683	536	6.96	1.32
**Endocrine**	Obesity	obesity	hypertension	yes	2.26	2.13	2.39	0.03	1.39E-180	144148	6009	6.88	1.43
**Eye**	Visual impairment and blindness	blind	hypertension	yes	1.88	1.53	2.31	0.1	1.47E-09	153508	407	6.96	1.31
**Eye**	Cataract	cataract	hypertension	yes	1.31	1.25	1.38	0.03	5.33E-27	148730	7052	6.9	1.43
**Eye**	Diabetic ophthalmic complications	diab_eye	hypertension	yes	3.78	3.5	4.08	0.04	1.46E-256	151709	2959	6.94	1.34
**Eye**	Macular degeneration	macula_degen	hypertension	yes	1.5	1.33	1.7	0.06	1.51E-10	152253	1080	6.96	1.32

**Eye**	Retinal vascular occlusions	retinal_vasc_occl	hypertension	yes	1.68	1.37	2.06	0.1	5.17E-07	153633	411	6.96	1.31
**Genitourinary**	Acute Kidney Injury	AKI	hypertension	yes	2.69	2.44	2.96	0.05	1.89E-89	153834	1770	6.96	1.32
**Genitourinary**	Hyperplasia of prostate	BPH	hypertension	yes	1.17	1.09	1.25	0.03	7.55E-06	149793	3910	6.92	1.4
**Genitourinary**	Chronic Kidney Disease	CKD	hypertension	yes	3.05	2.82	3.3	0.04	1.38E-168	152307	2647	6.93	1.33
**Genitourinary**	Erectile dysfunction	ED	hypertension	yes	1.64	1.55	1.73	0.03	4.38E-66	147822	5621	6.91	1.43
**Genitourinary**	End stage renal disease	ESRD	hypertension	yes	3.81	2.84	5.12	0.15	7.40E-19	153721	198	6.98	1.31
**Genitourinary**	Glomerulonephritis	GN	hypertension	yes	3.55	2.9	4.33	0.1	1.82E-35	153431	428	6.96	1.31
**Genitourinary**	Tubulo-interstitial nephritis	TIN	hypertension	yes	2.12	1.57	2.86	0.15	9.20E-07	153859	201	6.98	1.31
**Genitourinary**	Urinary Incontinence	urine_incont	hypertension	yes	1.25	1.15	1.35	0.04	1.07E-07	148453	3185	6.93	1.35
**Genitourinary**	Urolithiasis	urolithiasis	hypertension	yes	1.4	1.24	1.58	0.06	4.29E-08	150319	1327	6.95	1.33
**Haematological or immunological**	Iron deficiency anaemia	IDA	hypertension	yes	1.78	1.64	1.94	0.04	3.91E-42	150047	2610	6.94	1.34
**Haematological or**	Vitamin B12 deficiency anaemia	b12_def	hypertension	yes	2.01	1.76	2.28	0.07	2.40E-26	152741	1071	6.96	1.32

**immunological**													
**Haematological or immunological**	Folate deficiency anaemia	folatedef	hypertension	yes	1.72	1.37	2.16	0.12	3.19E-06	153895	352	6.96	1.31
**Haematological or immunological**	Other anaemias	oth_anaemia	hypertension	yes	1.8	1.68	1.93	0.04	1.38E-61	148350	3670	6.93	1.34
**Infections**	Bacterial Diseases (excl TB)	bacterial	hypertension	yes	1.62	1.54	1.7	0.03	3.75E-75	143690	6922	6.9	1.42
**Infections**	Infection of bones and joints	bone	hypertension	yes	2.61	2	3.42	0.14	2.25E-12	153667	235	6.98	1.31
**Infections**	Infections of the digestive system	digestive	hypertension	yes	1.38	1.28	1.49	0.04	4.23E-17	152729	3297	6.94	1.34
**Infections**	Lower Respiratory Tract Infections	lrti	hypertension	yes	1.47	1.38	1.57	0.03	1.28E-31	149287	4282	6.94	1.34
**Infections**	Other or unspecified infectious organisms	oth_organisms	hypertension	yes	1.41	1.34	1.48	0.02	1.48E-44	149636	8008	6.89	1.43
**Infections**	Infections of Other or unspecified organs	oth_organs	hypertension	yes	1.61	1.5	1.73	0.04	1.87E-39	150859	3565	6.94	1.34
**Infections**	Rheumatic fever	rh_fever	hypertension	yes	2.67	1.95	3.66	0.16	9.70E-10	153241	167	6.97	1.31

**Infections**	Septicaemia	sepsis	hypertension	yes	1.54	1.38	1.72	0.06	5.75E-14	153580	1379	6.96	1.32
**Infections**	Infection of skin and subcutaneous tissues	skin	hypertension	yes	1.92	1.76	2.11	0.05	1.33E-45	151756	2174	6.95	1.33
**Infections**	Urinary Tract Infections	uti	hypertension	yes	1.61	1.5	1.74	0.04	2.14E-35	151487	3064	6.94	1.33
**Infections**	Viral diseases (excl chronic hepatitis/HIV)	viral	hypertension	yes	1.48	1.3	1.69	0.07	5.27E-09	147978	1102	6.96	1.32
**Musculoskeletal**	Osteoarthritis (excl spine)	OA	hypertension	yes	1.34	1.28	1.39	0.02	7.11E-43	130200	11491	6.8	1.51
**Musculoskeletal**	Rheumatoid Arthritis	RhA	hypertension	yes	1.61	1.39	1.86	0.07	7.40E-11	151660	896	6.96	1.32
**Musculoskeletal**	Carpal tunnel syndrome	carpal_tunnel	hypertension	yes	1.5	1.38	1.63	0.04	3.11E-21	147905	2854	6.94	1.34
**Musculoskeletal**	Enthesopathies & synovial disorders	enthesopathy	hypertension	yes	1.17	1.12	1.21	0.02	6.27E-14	126805	13951	6.8	1.58
**Musculoskeletal**	Gout	gout	hypertension	yes	2.38	2.2	2.58	0.04	4.33E-101	149466	2656	6.94	1.34
**Musculoskeletal**	Intervertebral disc disorders	intervert_disc	hypertension	yes	1.27	1.17	1.39	0.04	4.62E-08	146841	2710	6.94	1.34
**Musculoskeletal**	Spinal stenosis	spinal_stenosis	hypertension	yes	1.66	1.48	1.86	0.06	4.07E-18	153352	1299	6.95	1.33

**Musculoskeletal**	Spondylosis	spondylosis	hypertension	yes	1.38	1.28	1.48	0.04	8.12E-18	145448	3556	6.92	1.4
**Neurological**	Disorders of autonomic nervous system	autonomic_neuro	hypertension	yes	2.18	1.61	2.94	0.15	4.47E-07	153810	191	6.97	1.31
**Neurological**	Postviral fatigue syndrome, neurasthenia and fibromyalgia	chronic_fatigue	hypertension	yes	1.45	1.23	1.71	0.08	9.42E-06	151083	840	6.95	1.32
**Neurological**	Diabetic neurological complications	dm_neuro	hypertension	yes	7.09	5.23	9.61	0.16	1.42E-36	153829	221	6.98	1.31
**Neurological**	Epilepsy	epilepsy	hypertension	yes	1.6	1.28	1.99	0.11	3.65E-05	151976	369	6.96	1.31
**Neurological**	Peripheral neuropathies (excl. cranial nerve, carpal tunnel syndromes)	periph_neuro	hypertension	yes	1.53	1.4	1.68	0.05	1.51E-19	151144	2225	6.94	1.33
**Psychiatric**	Alcohol Problems	alc_problems	hypertension	yes	1.37	1.29	1.45	0.03	1.38E-25	151939	5819	6.89	1.42
**Psychiatric**	Anxiety disorders	anxiety	hypertension	yes	1.3	1.2	1.41	0.04	1.33E-10	127169	3421	6.91	1.41
**Psychiatric**	Delirium, not induced by alcohol and other	delirium	hypertension	yes	1.9	1.48	2.43	0.13	4.61E-07	154029	260	6.98	1.31

	psychoactive substances												
**Psychiatric**	Depression	depression	hypertension	yes	1.49	1.37	1.62	0.04	2.66E-21	106004	3004	7	1.34
**Psychiatric**	Other psychoactive substance misuse	substance_misuse	hypertension	yes	1.74	1.33	2.28	0.14	4.96E-05	153575	266	6.98	1.31
**Respiratory**	COPD	COPD	hypertension	yes	1.4	1.3	1.5	0.04	6.40E-19	151345	3256	6.94	1.34
**Respiratory**	COPD_excl_bronchitis_NOS	COPD_excl_bronchitis_NOS	hypertension	yes	1.42	1.32	1.53	0.04	3.82E-20	151407	3171	6.94	1.34
**Respiratory**	Allergic and chronic rhinitis	allergic_rhinitis	hypertension	yes	1.18	1.08	1.28	0.04	1.53E-04	110387	2952	6.93	1.41
**Respiratory**	Asthma	asthma	hypertension	yes	1.45	1.32	1.6	0.05	9.69E-15	133123	2221	6.94	1.33
**Respiratory**	Pleural effusion	pleural_effusion	hypertension	yes	1.43	1.28	1.59	0.06	1.32E-10	153405	1481	6.96	1.32
**Respiratory**	Pulmonary collapse (excl pneumothorax)	pulm_collapse	hypertension	yes	1.43	1.2	1.71	0.09	5.91E-05	153850	578	6.96	1.31
**Respiratory**	Respiratory failure	resp_failure	hypertension	yes	1.8	1.55	2.1	0.08	4.00E-14	153944	719	6.96	1.31
**Respiratory**	Chronic sinusitis	sinusitis	hypertension	yes	1.21	1.13	1.29	0.03	2.94E-08	139467	4868	6.91	1.43
**Respiratory**	Sleep apnoea	sleep_apnoea	hypertension	yes	2.58	2.3	2.88	0.06	3.71E-61	152546	1400	6.95	1.32
**Skin**	Actinic keratosis	actinic_keratosis	hypertension	yes	0.82	0.77	0.88	0.03	1.38E-08	151055	4592	6.92	1.42

**Skin**	Dermatitis (atopc/contact/other/unspecified)	dermatitis	hypertension	yes	1.24	1.18	1.31	0.03	2.64E-15	131299	7152	6.89	1.46
**Benign neoplasm or Carcinoma in situ**	Benign neoplasm of brain and other parts of central nervous system	benign_brain	hypertension	yes	1.12	0.87	1.45	0.13	3.86E-01	153561	310	6.96	1.31
**Benign neoplasm or Carcinoma in situ**	Benign neoplasm of ovary	benign_ovary	hypertension	yes	1.2	1.01	1.43	0.09	4.19E-02	151115	802	6.96	1.32
**Benign neoplasm or Carcinoma in situ**	Benign neoplasm and polyp of uterus	benign_uterus	hypertension	yes	1.22	1.05	1.41	0.07	7.68E-03	151265	1170	6.96	1.32
**Benign neoplasm or Carcinoma in situ**	Carcinoma in situ_cervical	cin_cervical	hypertension	yes	0.95	0.84	1.07	0.06	4.07E-01	135261	2395	6.96	1.36
**Benign neoplasm or Carcinoma in situ**	Haemangioma, any site	haemangioma	hypertension	yes	1.03	0.87	1.2	0.08	7.56E-01	152349	877	6.96	1.32
**Benign neoplasm or**	Leiomyoma of uterus	leiomyoma	hypertension	yes	1.28	1.11	1.47	0.07	6.86E-04	146721	1558	6.95	1.33

**Carcinoma in situ**													
**Cancers**	Monoclonal gammopathy of undetermined significance (MGUS)	MGUS	hypertension	yes	1.03	0.78	1.37	0.14	8.28E-01	153949	235	6.96	1.31
**Cancers**	Non-Hodgkin Lymphoma	NHL	hypertension	yes	0.99	0.81	1.21	0.1	9.52E-01	153610	493	6.96	1.31
**Cancers**	Leukaemia	leukaemia	hypertension	yes	0.98	0.77	1.26	0.13	9.02E-01	153820	317	6.96	1.31
**Cancers**	Multiple myeloma and malignant plasma cell neoplasms	plasmacell	hypertension	yes	1.19	0.88	1.6	0.15	2.68E-01	153970	198	6.98	1.31
**Cancers**	Primary Malignancy_ biliary tract	pri_biliary	hypertension	yes	1.29	0.85	1.94	0.21	2.30E-01	154049	103	6.98	1.31
**Cancers**	Primary Malignancy_ Bladder	pri_bladder	hypertension	yes	1.15	0.96	1.39	0.1	1.39E-01	153595	496	6.96	1.31
**Cancers**	Primary Malignancy_ colorectal and anus	pri_bowel	hypertension	yes	1.09	0.96	1.23	0.06	1.91E-01	153096	1198	6.96	1.32
**Cancers**	Primary Malignancy_ Brain, Other CNS and Intracranial	pri_brain	hypertension	yes	0.69	0.47	1.01	0.19	5.33E-02	153944	165	6.98	1.31

**Cancers**	Primary Malignancy_ Breast	pri_breast	hypertension	yes	1.2	1.08	1.33	0.05	8.09E-04	150427	2019	6.95	1.33
**Cancers**	Primary Malignancy_ Malignant Melanoma	pri_melano ma	hypertension	yes	1.15	0.98	1.35	0.08	9.51E-02	152254	748	6.96	1.32
**Cancers**	Primary Malignancy_ Oesophage al	pri_oesoph	hypertension	yes	1.09	0.82	1.46	0.15	5.42E-01	153982	209	6.98	1.31
**Cancers**	Primary Malignancy_ Oro-pharyn geal	pri_oroph	hypertension	yes	1.36	1.01	1.82	0.15	4.16E-02	153728	216	6.98	1.31
**Cancers**	Primary Malignancy_ Other Organs	pri_other	hypertension	yes	1.13	0.99	1.29	0.07	6.27E-02	153204	1085	6.96	1.31
**Cancers**	Primary Malignancy_ Ovarian	pri_ovarian	hypertension	yes	1.1	0.83	1.46	0.15	5.15E-01	153666	260	6.98	1.31
**Cancers**	Primary Malignancy_ Pancreatic	pri_pancr	hypertension	yes	1.35	1.02	1.79	0.14	3.55E-02	154034	217	6.98	1.31
**Cancers**	Primary Malignancy_ Prostate	pri_prost	hypertension	yes	0.96	0.87	1.06	0.05	4.15E-01	152908	1895	6.95	1.33
**Cancers**	Primary Malignancy_ Other Skin and subcutaneo us tissue	pri_skin	hypertension	yes	0.96	0.9	1.03	0.03	2.73E-01	149842	4488	6.92	1.41

**Cancers**	Primary Malignancy_ Stomach	pri_stomach	hypertension	yes	1.3	0.95	1.76	0.16	9.69E-02	153976	185	6.98	1.31
**Cancers**	Primary Malignancy_ Uterine	pri_uterine	hypertension	yes	1.29	1	1.65	0.13	4.94E-02	153595	314	6.96	1.31
**Cancers**	Secondary Malignancy_ Lymph Nodes	sec_LN	hypertension	yes	1.16	1.03	1.3	0.06	1.14E-02	153395	1508	6.96	1.32
**Cancers**	Secondary Malignancy_ Adrenal gland	sec_adrenal	hypertension	yes	1.43	0.96	2.14	0.2	8.01E-02	154071	107	6.98	1.31
**Cancers**	Secondary Malignancy_ Bone	sec_bone	hypertension	yes	1.02	0.87	1.19	0.08	8.24E-01	153994	756	6.98	1.31
**Cancers**	Secondary Malignancy_ Brain, Other CNS and Intracranial	sec_brain	hypertension	yes	1.28	1	1.63	0.12	4.77E-02	154057	319	6.98	1.31
**Cancers**	Secondary malignancy_ Liver and intrahepatic bile duct	sec_liver	hypertension	yes	1.2	1.04	1.39	0.07	1.45E-02	153975	865	6.98	1.31
**Cancers**	Secondary Malignancy_ Lung	sec_lung	hypertension	yes	1.14	0.97	1.35	0.08	1.20E-01	154003	683	6.98	1.31
**Cancers**	Secondary Malignancy_ Other organs	sec_other	hypertension	yes	1.12	0.95	1.32	0.08	1.84E-01	153903	701	6.97	1.31

**Cancers**	Secondary Malignancy_ retroperitoneum and peritoneum	sec_peritoneum	hypertension	yes	0.99	0.8	1.22	0.11	9.13E-01	154025	454	6.98	1.31
**Cancers**	Secondary Malignancy_ Pleura	sec_pleura	hypertension	yes	1	0.74	1.36	0.16	9.97E-01	154060	218	6.98	1.31
**Cardiovascular**	Pulmonary embolism	PE	hypertension	yes	1.08	0.94	1.25	0.07	2.72E-01	152697	918	6.96	1.31
**Cardiovascular**	Supraventricular tachycardia	SVT	hypertension	yes	1.19	0.98	1.44	0.1	8.48E-02	153125	517	6.96	1.31
**Cardiovascular**	Subarachnoid haemorrhage	Subarach	hypertension	yes	1.26	0.91	1.74	0.17	1.72E-01	153669	189	6.98	1.31
**Cardiovascular**	Atrioventricular block, second degree	av_block_2	hypertension	yes	1.64	1.17	2.3	0.17	4.24E-03	154018	147	6.98	1.31
**Cardiovascular**	Atrioventricular block, complete	av_block_3	hypertension	yes	1.65	1.18	2.3	0.17	3.22E-03	153996	147	6.98	1.31
**Cardiovascular**	Dilated cardiomyopathy	dcm	hypertension	yes	1.25	0.91	1.71	0.16	1.66E-01	153932	183	6.98	1.31
**Cardiovascular**	Raynaud's syndrome	raynauds	hypertension	yes	0.84	0.71	0.99	0.08	3.89E-02	152528	912	6.96	1.32
**Cardiovascular**	Sick sinus syndrome	sick_sinus	hypertension	yes	1.5	1.06	2.12	0.18	2.07E-02	153979	141	6.98	1.31
**Cardiovascular**	Venous thromboem	vte_ex_pe	hypertension	yes	1.2	1.05	1.37	0.07	6.65E-03	150703	1091	6.96	1.32

	bolic disease (Excl PE)												
**Digestive**	Inflammatory bowel disease (IBD)	IBD	hypertension	yes	1.44	1.16	1.77	0.11	7.40E-04	151928	441	6.96	1.31
**Digestive**	Irritable bowel syndrome	IBS	hypertension	yes	1.23	1.08	1.4	0.07	1.47E-03	147371	1325	6.93	1.32
**Digestive**	Anal fissure	anal_fissure	hypertension	yes	1.06	0.92	1.23	0.07	4.16E-01	150960	1117	6.96	1.32
**Digestive**	Anorectal fistula	anorectal_fistula	hypertension	yes	1.14	0.84	1.55	0.16	3.95E-01	153236	247	6.96	1.31
**Digestive**	Anorectal prolapse	anorectal_prolapse	hypertension	yes	0.95	0.74	1.22	0.13	6.93E-01	153650	340	6.96	1.31
**Digestive**	Appendicitis	appendicitis	hypertension	yes	1.06	0.87	1.3	0.1	5.54E-01	146680	588	6.96	1.31
**Digestive**	Barrett's oesophagus	barretts	hypertension	yes	1.14	0.98	1.31	0.07	8.14E-02	153220	932	6.96	1.32
**Digestive**	Cholangitis	cholangitis	hypertension	yes	1.59	1.21	2.1	0.14	1.03E-03	153950	223	6.98	1.31
**Digestive**	Coeliac disease	coeliac	hypertension	yes	0.96	0.71	1.29	0.15	7.82E-01	152543	244	6.97	1.31
**Digestive**	Crohn's disease	crohns	hypertension	yes	1.55	1.11	2.15	0.17	9.19E-03	153368	180	6.97	1.31
**Digestive**	Abdominal Hernia	hernia_abdo	hypertension	yes	1.03	0.97	1.1	0.03	3.07E-01	143859	4763	6.92	1.41
**Digestive**	Peritonitis	peritonitis	hypertension	yes	1.35	1.12	1.62	0.09	1.42E-03	153043	568	6.96	1.31
**Digestive**	Ulcerative colitis	ulc_colitis	hypertension	yes	1.32	1.04	1.69	0.12	2.46E-02	152450	337	6.96	1.31
**Digestive**	Volvulus	volvulus	hypertension	yes	1.58	1.15	2.16	0.16	4.34E-03	153952	179	6.97	1.31
**Ear**	Hearing loss	deaf	hypertension	yes	1.11	1.05	1.17	0.03	5.08E-04	140976	5897	6.9	1.43

**Ear**	Meniere disease	meniere	hypertension	yes	1.03	0.74	1.45	0.17	8.44E-01	153131	197	6.96	1.31
**Ear**	Tinnitus	tinnitus	hypertension	yes	1.06	0.95	1.17	0.05	2.94E-01	137398	2177	7.04	1.31
**Endocrine**	Hypothyroidism	hypothyroid	hypertension	yes	1	0.9	1.12	0.06	9.42E-01	144663	1962	6.95	1.33
**Endocrine**	Hypo or hyperthyroidism	thyroid	hypertension	yes	1.05	0.94	1.17	0.06	4.06E-01	142846	1971	6.94	1.33
**Endocrine**	Thyroiditis unspecified	thyroid_nos	hypertension	yes	1.4	0.9	2.18	0.23	1.33E-01	151208	114	6.96	1.31
**Eye**	Anterior and Intermediate Uveitis	ant_uveitis	hypertension	yes	1.24	0.94	1.62	0.14	1.25E-01	153081	290	6.96	1.31
**Eye**	Glaucoma	glaucoma	hypertension	yes	1.09	0.98	1.22	0.05	9.55E-02	150951	1683	6.95	1.33
**Eye**	Ptosis of eyelid	ptosis	hypertension	yes	1.38	1.12	1.7	0.11	2.45E-03	153571	429	6.96	1.31
**Eye**	Retinal detachments and breaks	retinal_detach	hypertension	yes	1.21	1.03	1.43	0.08	2.17E-02	152795	736	6.96	1.32
**Eye**	Scleritis and episcleritis	scleritis	hypertension	yes	0.95	0.58	1.57	0.25	8.45E-01	153754	102	6.98	1.31
**Genitourinary**	Postcoital and contact bleeding	PCB	hypertension	yes	0.6	0.4	0.91	0.21	1.51E-02	152510	336	6.96	1.31
**Genitourinary**	Postmenopausal bleeding	PMB	hypertension	yes	1.15	1.03	1.28	0.05	1.13E-02	148776	2149	6.95	1.33
**Genitourinary**	Non-acute cystitis	chr_cystitis	hypertension	yes	1.4	1.05	1.86	0.15	2.35E-02	153788	217	6.97	1.31
**Genitourinary**	Dysmenorrhoea	dysmenorrhoea	hypertension	yes	1.34	0.82	2.2	0.25	2.45E-01	151328	183	6.98	1.31

**Genitourinary**	Endometrial hyperplasia and hypertrophy	endometrial_hyper	hypertension	yes	0.83	0.58	1.19	0.18	3.10E-01	153321	259	6.96	1.31
**Genitourinary**	Endometriosis	endometriosis	hypertension	yes	1.57	1.18	2.1	0.15	2.10E-03	151057	320	6.96	1.32
**Genitourinary**	Female genital prolapse	female_genital_prolapse	hypertension	yes	1.11	1.02	1.22	0.04	1.62E-02	148909	2832	6.94	1.35
**Genitourinary**	Hydrocoele (incl infected)	hydrocele	hypertension	yes	1.38	1.13	1.68	0.1	1.77E-03	153146	440	6.96	1.31
**Genitourinary**	Menorrhagia and polymenorrhoea	menorrhagia	hypertension	yes	1.1	0.91	1.33	0.1	3.16E-01	142650	1520	6.95	1.33
**Genitourinary**	Neuromuscular dysfunction of bladder	neuro_bladder	hypertension	yes	1.15	1.01	1.31	0.07	3.94E-02	152401	1133	6.95	1.32
**Genitourinary**	Obstructive and reflux uropathy	obstr_reflux	hypertension	yes	1.34	1.15	1.57	0.08	2.24E-04	153469	776	6.96	1.31
**Haematological or immunological**	Agranulocytosis	agranulocytosis	hypertension	yes	1.05	0.92	1.2	0.07	4.70E-01	153144	1229	6.96	1.32
**Haematological or immunological**	Aplastic anaemias	aplastic	hypertension	yes	1.52	1.06	2.18	0.18	2.17E-02	154007	133	6.98	1.31
**Haematological or**	Primary or Idiopathic	pri_thrombocytopaenia	hypertension	yes	1.29	0.99	1.69	0.14	6.26E-02	153669	255	6.96	1.31

**immunological**	Thrombocytopaenia												
**Haematological or immunological**	Secondary or other Thrombocytopaenia	sec_oth_thrombocytopaenia	hypertension	yes	1.35	1.12	1.62	0.09	1.63E-03	153665	530	6.96	1.31
**Haematological or immunological**	Splenomegaly	splenomegaly	hypertension	yes	1.93	1.34	2.77	0.19	4.03E-04	153969	129	6.98	1.31
**Haematological or immunological**	Thrombophilia	thrombophilia	hypertension	yes	1.67	1.19	2.35	0.17	3.17E-03	153732	174	6.98	1.31
**Infections**	Female pelvic inflammatory disease	PID	hypertension	yes	1.34	1.05	1.7	0.12	1.76E-02	152924	420	6.96	1.31
**Infections**	Infection of anal and rectal regions	anorectal	hypertension	yes	1.18	0.84	1.67	0.18	3.46E-01	153653	189	6.98	1.31
**Infections**	Ear and Upper Respiratory Tract Infections	ear_urti	hypertension	yes	1.23	1.06	1.43	0.08	5.81E-03	150069	940	6.98	1.31
**Infections**	Eye infections	eye	hypertension	yes	1.07	0.72	1.61	0.21	7.31E-01	153828	124	6.98	1.31
**Infections**	Infection of liver	liver	hypertension	yes	1.21	0.79	1.86	0.22	3.71E-01	153413	114	6.98	1.31
**Infections**	Infection of male genital system	male_GU	hypertension	yes	1.41	1.07	1.87	0.14	1.53E-02	153788	231	6.96	1.31

**Infections**	Mycoses	mycoses	hypertension	yes	1.34	1.15	1.57	0.08	2.12E-04	153774	753	6.96	1.31
**Infections**	Other nervous system infections	oth_nerv_sys	hypertension	yes	1.36	0.93	1.99	0.2	1.16E-01	153930	129	6.98	1.31
**Musculoskeletal**	Giant Cell arteritis	GCA	hypertension	yes	1.02	0.75	1.39	0.16	8.89E-01	153956	194	6.98	1.31
**Musculoskeletal**	Polymyalgia Rheumatica	PMR	hypertension	yes	1.16	1.01	1.33	0.07	4.25E-02	153501	895	6.96	1.32
**Musculoskeletal**	Psoriatic arthropathy	PSA	hypertension	yes	1.38	0.98	1.93	0.17	6.24E-02	153520	195	6.96	1.31
**Musculoskeletal**	Collapsed vertebra	collapsed_vert	hypertension	yes	1.13	0.88	1.45	0.13	3.22E-01	153793	299	6.98	1.31
**Musculoskeletal**	Fibromatoses	fibromatosis	hypertension	yes	0.92	0.83	1.03	0.06	1.56E-01	152148	1726	6.95	1.33
**Musculoskeletal**	Fracture of hip	fracture_hip	hypertension	yes	1.11	0.92	1.34	0.1	2.68E-01	153621	538	6.96	1.31
**Musculoskeletal**	Fracture of wrist	fracture_wrist	hypertension	yes	0.95	0.85	1.07	0.06	3.90E-01	150487	1656	6.95	1.33
**Musculoskeletal**	Osteoporosis	osteoporosis	hypertension	yes	0.95	0.89	1.02	0.04	1.52E-01	149639	4270	6.92	1.41
**Musculoskeletal**	Scoliosis	scoliosis	hypertension	yes	1.29	1.02	1.62	0.12	3.18E-02	153292	351	6.97	1.31
**Musculoskeletal**	Sjogren's disease	sjogren	hypertension	yes	1.81	1.22	2.71	0.2	3.55E-03	153824	116	6.98	1.31
**Musculoskeletal**	Spondylolisthesis	spondylolisthesis	hypertension	yes	1.38	1.13	1.69	0.1	1.72E-03	153400	447	6.96	1.31
**Neurological**	Multiple sclerosis	MS	hypertension	yes	1.19	0.76	1.85	0.23	4.49E-01	153420	132	6.98	1.31
**Neurological**	Parkinson's disease	Parkinsons	hypertension	yes	1.03	0.85	1.26	0.1	7.30E-01	153787	481	6.96	1.31

**Neurological**	Bell's palsy	bells	hypertension	yes	1.41	1.11	1.8	0.12	5.60E-03	153187	334	6.96	1.31
**Neurological**	Essential tremor	essential_tremor	hypertension	yes	1.15	0.92	1.43	0.11	2.16E-01	153805	376	6.96	1.31
**Neurological**	Migraine	migraine	hypertension	yes	1.12	1	1.26	0.06	5.68E-02	140282	1926	6.94	1.34
**Neurological**	Trigeminal neuralgia	trigem_neur	hypertension	yes	1.13	0.92	1.39	0.11	2.54E-01	153335	474	6.96	1.31
**Perinatal**	Congenital malformations of cardiac septa	congenital_septal	hypertension	yes	1.28	0.83	1.99	0.22	2.65E-01	153800	110	6.98	1.31
**Psychiatric**	Bipolar affective disorder and mania	BAD	hypertension	yes	1.36	0.94	1.95	0.19	1.01E-01	152960	163	6.98	1.31
**Psychiatric**	Alzheimer's disease	alzheimer	hypertension	yes	0.98	0.79	1.21	0.11	8.54E-01	154053	390	6.96	1.31
**Psychiatric**	Dementia	dementia	hypertension	yes	1	0.76	1.32	0.14	9.84E-01	153204	228	6.98	1.3
**Psychiatric**	Dementia (excluding Alzheimer's)	dementia_ex_Alz	hypertension	yes	1.11	0.79	1.56	0.17	5.40E-01	153217	146	6.98	1.31
**Psychiatric**	Schizophrenia, schizotypal and delusional disorders	schizo	hypertension	yes	1.55	1.01	2.37	0.22	4.32E-02	153513	116	6.98	1.31
**Respiratory**	Aspiration pneumonitis	aspiration_pneumo	hypertension	yes	1.32	1	1.74	0.14	4.87E-02	154032	229	6.98	1.31
**Respiratory**	Bronchiectasis	bronchiectasis	hypertension	yes	1.26	1.07	1.47	0.08	4.44E-03	153099	734	6.96	1.32

**Respiratory**	Hypertrophy of nasal turbinates	hyper_nasal_turbs	hypertension	yes	1.23	0.85	1.8	0.19	2.73E-01	153544	158	6.98	1.31
**Respiratory**	Nasal polyp	nasal_polyp	hypertension	yes	1.11	0.93	1.32	0.09	2.41E-01	151531	687	6.96	1.32
**Respiratory**	Pleural plaque	pleural_plaque	hypertension	yes	1.06	0.84	1.33	0.12	6.48E-01	153972	322	6.96	1.31
**Respiratory**	Pneumothorax	pneumothorax	hypertension	yes	1.05	0.78	1.4	0.15	7.63E-01	153290	230	6.98	1.31
**Respiratory**	Other interstitial pulmonary diseases with fibrosis	pulm_fibrosis	hypertension	yes	1.25	1	1.57	0.12	5.50E-02	153842	330	6.96	1.31
**Skin**	Acne	acne	hypertension	yes	1.66	1.22	2.27	0.16	1.35E-03	150556	248	6.98	1.31
**Skin**	Alopecia areata	alopecia_areata	hypertension	yes	1.64	1.09	2.47	0.21	1.86E-02	153595	126	6.98	1.31
**Skin**	Keratitis	keratitis	hypertension	yes	1.25	0.99	1.59	0.12	5.80E-02	153298	377	6.96	1.31
**Skin**	Lichen planus	lichen_planus	hypertension	yes	1.1	0.91	1.33	0.09	3.10E-01	152800	626	6.96	1.32
**Skin**	Psoriasis	psoriasis	hypertension	yes	1.19	1.05	1.35	0.06	6.09E-03	148816	1340	6.95	1.33
**Skin**	Rosacea	rosacea	hypertension	yes	1.02	0.91	1.13	0.05	7.77E-01	149906	2095	6.95	1.33
**Skin**	Seborrheic dermatitis	seb_derm	hypertension	yes	1.16	1.06	1.27	0.04	7.69E-04	144426	2845	6.94	1.35
**Skin**	Urticaria	urticaria	hypertension	yes	1.18	1.06	1.32	0.06	2.36E-03	149475	1874	6.95	1.34
**Skin**	Vitiligo	vitiligo	hypertension	yes	1.14	0.77	1.68	0.2	5.23E-01	153604	143	6.98	1.31

Table S12. Age and sex adjusted hazard ratio of BMI association with incident disease (*P* < 0.002) (reference value is healthy BMI)

group	phenotype_descr	phenotype	factor	levels	HR	ci_left	ci_right	se	pvalue_sci	n	n_events	median_follow_up	IQR_follow_up
**Benign neoplasm or Carcinoma in situ**	Benign neoplasm of colon, rectum, anus and anal canal	benign_colon	BMI	overweight	1.19	1.12	1.27	0.03	1.60E-08	149732	6630	6.9	1.43
**Benign neoplasm or Carcinoma in situ**	Benign neoplasm of colon, rectum, anus and anal canal	benign_colon	BMI	obese	1.56	1.46	1.67	0.03	9.59E-41	149732	6630	6.9	1.43
**Benign neoplasm or Carcinoma in situ**	Benign neoplasm of stomach and duodenum	benign_stomach	BMI	obese	1.6	1.4	1.84	0.07	1.91E-11	153375	1456	6.95	1.33
**Benign neoplasm or Carcinoma in situ**	Benign neoplasm and polyp of uterus	benign_uterus	BMI	obese	2	1.74	2.31	0.07	1.70E-21	151265	1170	6.96	1.32
**Benign neoplasm or Carcinoma in situ**	Leiomyoma of uterus	leiomyoma	BMI	obese	1.45	1.28	1.64	0.06	6.86E-09	146721	1558	6.95	1.33
**Cancers**	Primary Malignancy_Kidney and Ureter	pri_kidney	BMI	obese	1.88	1.4	2.53	0.15	3.22E-05	153828	331	6.96	1.31
**Cancers**	Primary Malignancy_Other	pri_skin	BMI	obese	0.8	0.74	0.87	0.04	8.30E-08	149842	4488	6.92	1.41

	Skin and subcutaneous tissue												
**Cancers**	Primary Malignancy_Uterine	pri_uterine	BMI	obese	3.16	2.37	4.22	0.15	6.59E-15	153595	314	6.96	1.31
**Cardiovascular**	Atrial fibrillation	AF	BMI	obese	1.76	1.61	1.92	0.04	8.42E-37	150562	3624	6.93	1.34
**Cardiovascular**	Coronary heart disease not otherwise specified	CHD_NOS	BMI	obese	1.99	1.82	2.17	0.05	1.84E-51	144817	3776	6.93	1.35
**Cardiovascular**	Coronary heart disease not otherwise specified	CHD_NOS	BMI	overweight	1.35	1.24	1.47	0.04	5.87E-12	144817	3776	6.93	1.35
**Cardiovascular**	Left bundle branch block	LBBB	BMI	obese	1.66	1.33	2.08	0.12	1.02E-05	153851	574	6.96	1.31
**Cardiovascular**	Pulmonary embolism	PE	BMI	overweight	1.6	1.34	1.92	0.09	2.15E-07	152697	918	6.96	1.31
**Cardiovascular**	Pulmonary embolism	PE	BMI	obese	2.36	1.96	2.83	0.09	6.11E-20	152697	918	6.96	1.31
**Cardiovascular**	Rheumatic valve dz	Rh_valve	BMI	obese	2.54	1.67	3.88	0.22	1.52E-05	153877	171	6.98	1.31
**Cardiovascular**	Atrioventricular block, first degree	av_block_1	BMI	obese	1.95	1.49	2.57	0.14	1.48E-06	153969	428	6.96	1.31
**Cardiovascular**	Heart failure	hf	BMI	obese	2.43	2.13	2.76	0.07	6.33E-42	152718	1836	6.95	1.32
**Cardiovascular**	Nonrheumatic aortic valve disorders	nonRh_aortic	BMI	obese	2.32	1.92	2.81	0.1	3.06E-18	153451	881	6.96	1.32
**Cardiovascular**	Nonrheumatic aortic valve disorders	nonRh_aortic	BMI	overweight	1.48	1.23	1.78	0.09	3.20E-05	153451	881	6.96	1.32
**Cardiovascular**	Peripheral arterial disease	peripheral_arterial_disease	BMI	obese	1.52	1.29	1.8	0.09	8.94E-07	152668	959	6.96	1.31

**Cardiovascular**	Raynaud's syndrome	raynauds	BMI	obese	0.45	0.37	0.54	0.1	3.76E-16	152528	912	6.96	1.32
**Cardiovascular**	Raynaud's syndrome	raynauds	BMI	underweight	2.9	1.83	4.6	0.23	5.73E-06	152528	912	6.96	1.32
**Cardiovascular**	Raynaud's syndrome	raynauds	BMI	overweight	0.63	0.55	0.73	0.07	6.94E-10	152528	912	6.96	1.32
**Cardiovascular**	Stable angina	stable_angina	BMI	overweight	1.56	1.42	1.71	0.05	1.18E-21	148093	3508	6.93	1.35
**Cardiovascular**	Stable angina	stable_angina	BMI	obese	2.23	2.03	2.45	0.05	4.29E-61	148093	3508	6.93	1.35
**Cardiovascular**	Unstable Angina	unstable_angina	BMI	obese	2.03	1.7	2.42	0.09	4.10E-15	152534	963	6.96	1.32
**Cardiovascular**	Venous thromboembolic disease (Excl PE)	vte_ex_pe	BMI	overweight	1.37	1.17	1.6	0.08	1.23E-04	150703	1091	6.96	1.32
**Cardiovascular**	Venous thromboembolic disease (Excl PE)	vte_ex_pe	BMI	obese	2.11	1.79	2.48	0.08	5.29E-19	150703	1091	6.96	1.32
**Digestive**	Gastro-oesophageal reflux disease	GORD	BMI	obese	1.48	1.38	1.57	0.03	1.32E-32	141700	6892	6.88	1.43
**Digestive**	Gastro-oesophageal reflux disease	GORD	BMI	overweight	1.28	1.21	1.35	0.03	1.93E-16	141700	6892	6.88	1.43
**Digestive**	Angiodysplasia of colon	angiodysplasia_colon	BMI	obese	2.62	1.63	4.22	0.24	6.84E-05	154022	131	6.98	1.31
**Digestive**	Anorectal fistula	anorectal_fistula	BMI	obese	2.09	1.47	2.98	0.18	4.32E-05	153236	247	6.96	1.31
**Digestive**	Anorectal prolapse	anorectal_prolapse	BMI	overweight	0.61	0.48	0.78	0.13	1.06E-04	153650	340	6.96	1.31
**Digestive**	Cholangitis	cholangitis	BMI	overweight	2.67	1.77	4.03	0.21	2.59E-06	153950	223	6.98	1.31
**Digestive**	Cholangitis	cholangitis	BMI	obese	3.21	2.09	4.93	0.22	9.98E-08	153950	223	6.98	1.31

**Digestive**	Cholecystitis	cholecystitis	BMI	obese	3.28	2.85	3.76	0.07	3.96E-64	151776	1638	6.95	1.33
**Digestive**	Cholecystitis	cholecystitis	BMI	overweight	1.77	1.54	2.03	0.07	7.64E-16	151776	1638	6.95	1.33
**Digestive**	Cholelithiasis	cholelithiasis	BMI	obese	3.29	2.97	3.65	0.05	1.39E-116	148499	3014	6.93	1.34
**Digestive**	Cholelithiasis	cholelithiasis	BMI	overweight	1.88	1.7	2.08	0.05	7.28E-34	148499	3014	6.93	1.34
**Digestive**	Liver fibrosis, sclerosis and cirrhosis	cirrhosis	BMI	obese	2.13	1.65	2.77	0.13	1.06E-08	153646	402	6.96	1.31
**Digestive**	Diverticular disease of intestine (acute and chronic)	diverticuli	BMI	obese	1.68	1.58	1.79	0.03	1.48E-63	148653	7761	6.88	1.44
**Digestive**	Diverticular disease of intestine (acute and chronic)	diverticuli	BMI	overweight	1.31	1.23	1.38	0.03	4.07E-20	148653	7761	6.88	1.44
**Digestive**	Fatty Liver	fatty_liver	BMI	obese	8.25	6.77	10.06	0.1	1.21E-96	153645	1367	6.96	1.32
**Digestive**	Fatty Liver	fatty_liver	BMI	overweight	3.32	2.71	4.07	0.1	6.85E-31	153645	1367	6.96	1.32
**Digestive**	Gastritis and duodenitis	gastritis_duodenitis	BMI	obese	1.34	1.26	1.43	0.03	3.04E-20	144186	7105	6.89	1.44
**Digestive**	Gastritis and duodenitis	gastritis_duodenitis	BMI	overweight	1.16	1.09	1.23	0.03	4.02E-07	144186	7105	6.89	1.44
**Digestive**	Diaphragmatic hernia	hernia_diaphragm	BMI	overweight	1.44	1.35	1.54	0.03	1.33E-25	146847	5267	6.9	1.42
**Digestive**	Diaphragmatic hernia	hernia_diaphragm	BMI	obese	1.62	1.5	1.75	0.04	1.34E-36	146847	5267	6.9	1.42
**Digestive**	Oesophagitis and oesophageal ulcer	oesoph_ulc	BMI	obese	1.42	1.31	1.54	0.04	5.13E-17	146523	4429	6.92	1.41
**Digestive**	Oesophagitis and oesophageal ulcer	oesoph_ulc	BMI	overweight	1.36	1.26	1.46	0.04	6.41E-16	146523	4429	6.92	1.41
**Digestive**	Pancreatitis	pancreatitis	BMI	obese	1.99	1.56	2.55	0.13	4.55E-08	153343	460	6.96	1.31

**Digestive**	Peptic ulcer disease	ulcer_peptic	BMI	obese	1.61	1.39	1.86	0.07	8.31E-11	149047	1368	6.95	1.32
**Digestive**	Oesophageal varices	varices	BMI	obese	2.18	1.47	3.25	0.2	1.21E-04	153922	173	6.98	1.31
**Endocrine**	Hyperparathyroidism	PTH	BMI	obese	1.81	1.41	2.33	0.13	4.03E-06	153837	399	6.96	1.31
**Endocrine**	Diabetes NOS	diabetes_nos	BMI	obese	7.01	5.39	9.11	0.13	4.13E-48	147594	655	6.98	1.31
**Endocrine**	Diabetes NOS	diabetes_nos	BMI	overweight	2.27	1.73	2.99	0.14	4.20E-09	147594	655	6.98	1.31
**Endocrine**	Diabetes Type I	diabetes_t1	BMI	obese	3.27	2.52	4.24	0.13	6.42E-19	153297	421	6.96	1.31
**Endocrine**	Diabetes Type II	diabetes_t2	BMI	obese	9.47	8.53	10.52	0.05	0.00E+00	149744	4989	6.91	1.41
**Endocrine**	Diabetes Type II	diabetes_t2	BMI	overweight	2.88	2.58	3.21	0.06	1.95E-80	149744	4989	6.91	1.41
**Endocrine**	Hypothyroidism	hypothyroid	BMI	overweight	1.3	1.16	1.45	0.06	4.43E-06	144663	1962	6.95	1.33
**Endocrine**	Hypothyroidism	hypothyroid	BMI	obese	1.73	1.54	1.94	0.06	4.86E-20	144663	1962	6.95	1.33
**Endocrine**	Obesity	obesity	BMI	obese	105.81	85.37	131.15	0.11	0.00E+00	144148	6009	6.88	1.43
**Endocrine**	Obesity	obesity	BMI	overweight	11.38	9.13	14.19	0.11	5.48E-104	144148	6009	6.88	1.43
**Endocrine**	Hypo or hyperthyroidism	thyroid	BMI	obese	1.58	1.41	1.77	0.06	9.84E-15	142846	1971	6.94	1.33
**Eye**	Cataract	cataract	BMI	obese	1.23	1.16	1.31	0.03	8.29E-11	148730	7052	6.9	1.43
**Eye**	Diabetic ophthalmic complications	diab_eye	BMI	overweight	2.41	2.11	2.75	0.07	3.50E-38	151709	2959	6.94	1.34
**Eye**	Diabetic ophthalmic complications	diab_eye	BMI	obese	7.39	6.51	8.4	0.07	4.82E-207	151709	2959	6.94	1.34
**Genitourinary**	Acute Kidney Injury	AKI	BMI	obese	2.61	2.29	2.97	0.07	4.77E-47	153834	1770	6.96	1.32
**Genitourinary**	Chronic Kidney Disease	CKD	BMI	obese	2.56	2.3	2.85	0.05	1.80E-66	152307	2647	6.93	1.33
**Genitourinary**	Chronic Kidney Disease	CKD	BMI	overweight	1.44	1.29	1.6	0.05	2.19E-11	152307	2647	6.93	1.33

**Genitourinary**	Erectile dysfunction	ED	BMI	overweight	1.4	1.3	1.51	0.04	2.17E-19	147822	5621	6.91	1.43
**Genitourinary**	Erectile dysfunction	ED	BMI	obese	2.06	1.9	2.22	0.04	6.93E-75	147822	5621	6.91	1.43
**Genitourinary**	End stage renal disease	ESRD	BMI	obese	2.52	1.72	3.69	0.2	2.12E-06	153721	198	6.98	1.31
**Genitourinary**	Glomerulonephritis	GN	BMI	obese	2.64	2.04	3.41	0.13	1.37E-13	153431	428	6.96	1.31
**Genitourinary**	Postmenopausal bleeding	PMB	BMI	obese	1.44	1.29	1.6	0.05	3.74E-11	148776	2149	6.95	1.33
**Genitourinary**	Endometrial hyperplasia and hypertrophy	endometrial_hyper	BMI	obese	2.46	1.81	3.35	0.16	1.19E-08	153321	259	6.96	1.31
**Genitourinary**	Female genital prolapse	female_genital_prolapse	BMI	obese	1.24	1.12	1.36	0.05	2.38E-05	148909	2832	6.94	1.35
**Genitourinary**	Female genital prolapse	female_genital_prolapse	BMI	overweight	1.31	1.21	1.43	0.04	4.93E-10	148909	2832	6.94	1.35
**Genitourinary**	Menorrhagia and polymenorrhoea	menorrhagia	BMI	obese	1.5	1.32	1.71	0.07	7.40E-10	142650	1520	6.95	1.33
**Genitourinary**	Neuromuscular dysfunction of bladder	neuro_bladder	BMI	obese	1.64	1.4	1.92	0.08	8.88E-10	152401	1133	6.95	1.32
**Genitourinary**	Obstructive and reflux uropathy	obstr_reflux	BMI	obese	1.65	1.36	2	0.1	2.92E-07	153469	776	6.96	1.31
**Genitourinary**	Urinary Incontinence	urine_incont	BMI	overweight	1.28	1.18	1.4	0.04	1.42E-08	148453	3185	6.93	1.35
**Genitourinary**	Urinary Incontinence	urine_incont	BMI	obese	1.76	1.6	1.92	0.05	3.78E-34	148453	3185	6.93	1.35
**Genitourinary**	Urolithiasis	urolithiasis	BMI	obese	1.74	1.5	2.01	0.07	1.65E-13	150319	1327	6.95	1.33

**Haematological or immunological**	Iron deficiency anaemia	IDA	BMI	obese	1.59	1.44	1.76	0.05	4.68E-20	150047	2610	6.94	1.34
**Haematological or immunological**	Vitamin B12 deficiency anaemia	b12_def	BMI	obese	1.92	1.64	2.24	0.08	1.06E-16	152741	1071	6.96	1.32
**Haematological or immunological**	Other anaemias	oth_anaemia	BMI	obese	1.58	1.45	1.72	0.04	1.93E-26	148350	3670	6.93	1.34
**Haematological or immunological**	Other anaemias	oth_anaemia	BMI	underweight	2.23	1.58	3.15	0.18	5.95E-06	148350	3670	6.93	1.34
**Infections**	Female pelvic inflammatory disease	PID	BMI	obese	1.61	1.27	2.05	0.12	1.09E-04	152924	420	6.96	1.31
**Infections**	Infection of anal and rectal regions	anorectal	BMI	obese	2.66	1.79	3.94	0.2	1.21E-06	153653	189	6.98	1.31
**Infections**	Bacterial Diseases (excl TB)	bacterial	BMI	underweight	1.98	1.49	2.62	0.14	2.12E-06	143690	6922	6.9	1.42
**Infections**	Bacterial Diseases (excl TB)	bacterial	BMI	overweight	1.15	1.09	1.23	0.03	3.84E-06	143690	6922	6.9	1.42
**Infections**	Bacterial Diseases (excl TB)	bacterial	BMI	obese	1.79	1.68	1.91	0.03	4.40E-74	143690	6922	6.9	1.42
**Infections**	Infection of bones and joints	bone	BMI	obese	2.52	1.78	3.58	0.18	2.25E-07	153667	235	6.98	1.31
**Infections**	Infections of the digestive system	digestive	BMI	obese	1.29	1.18	1.42	0.05	3.40E-08	152729	3297	6.94	1.34
**Infections**	Ear and Upper Respiratory Tract Infections	ear_urti	BMI	obese	1.39	1.17	1.65	0.09	1.57E-04	150069	940	6.98	1.31

**Infections**	Lower Respiratory Tract Infections	lrti	BMI	obese	1.62	1.5	1.76	0.04	2.16E-32	149287	4282	6.94	1.34
**Infections**	Lower Respiratory Tract Infections	lrti	BMI	underweigh t	2.94	2.17	3.99	0.16	4.12E-12	149287	4282	6.94	1.34
**Infections**	Other or unspecified infectious organisms	oth_organis ms	BMI	obese	1.49	1.4	1.57	0.03	1.53E-40	149636	8008	6.89	1.43
**Infections**	Other or unspecified infectious organisms	oth_organis ms	BMI	underweigh t	1.97	1.53	2.54	0.13	1.74E-07	149636	8008	6.89	1.43
**Infections**	Infections of Other or unspecified organs	oth_organs	BMI	obese	1.57	1.44	1.72	0.04	4.72E-24	150859	3565	6.94	1.34
**Infections**	Infections of Other or unspecified organs	oth_organs	BMI	underweigh t	2.44	1.72	3.45	0.18	4.96E-07	150859	3565	6.94	1.34
**Infections**	Rheumatic fever	rh_fever	BMI	obese	2.45	1.61	3.73	0.21	2.86E-05	153241	167	6.97	1.31
**Infections**	Septicaemia	sepsis	BMI	obese	1.52	1.32	1.74	0.07	3.55E-09	153580	1379	6.96	1.32
**Infections**	Infection of skin and subcutaneous tissues	skin	BMI	obese	2.33	2.08	2.61	0.06	5.22E-49	151756	2174	6.95	1.33
**Infections**	Urinary Tract Infections	uti	BMI	obese	1.65	1.5	1.82	0.05	2.56E-25	151487	3064	6.94	1.33
**Infections**	Urinary Tract Infections	uti	BMI	underweigh t	2.45	1.68	3.57	0.19	3.20E-06	151487	3064	6.94	1.33
**Musculosk eletal**	Osteoarthritis (excl spine)	OA	BMI	overweight	1.36	1.3	1.42	0.02	2.29E-37	130200	11491	6.8	1.51
**Musculosk eletal**	Osteoarthritis (excl spine)	OA	BMI	obese	2.15	2.05	2.26	0.02	8.15E-206	130200	11491	6.8	1.51
**Musculosk eletal**	Rheumatoid Arthritis	RhA	BMI	overweight	1.39	1.17	1.65	0.09	1.51E-04	151660	896	6.96	1.32
**Musculosk eletal**	Rheumatoid Arthritis	RhA	BMI	obese	1.94	1.63	2.32	0.09	2.23E-13	151660	896	6.96	1.32
**Musculosk eletal**	Carpal tunnel syndrome	carpal_tunn el	BMI	overweight	1.34	1.22	1.47	0.05	1.41E-09	147905	2854	6.94	1.34

**Musculoskeletal**	Carpal tunnel syndrome	carpal_tunnel	BMI	obese	2.05	1.86	2.26	0.05	2.95E-47	147905	2854	6.94	1.34
**Musculoskeletal**	Enthesopathies & synovial disorders	enthesopathy	BMI	overweight	1.16	1.11	1.21	0.02	9.05E-13	126805	13951	6.8	1.58
**Musculoskeletal**	Enthesopathies & synovial disorders	enthesopathy	BMI	obese	1.32	1.26	1.38	0.02	9.37E-34	126805	13951	6.8	1.58
**Musculoskeletal**	Fibromatoses	fibromatosis	BMI	obese	0.61	0.53	0.69	0.07	6.43E-13	152148	1726	6.95	1.33
**Musculoskeletal**	Fracture of hip	fracture_hip	BMI	obese	0.51	0.4	0.65	0.12	4.33E-08	153621	538	6.96	1.31
**Musculoskeletal**	Fracture of hip	fracture_hip	BMI	overweight	0.65	0.53	0.78	0.1	6.29E-06	153621	538	6.96	1.31
**Musculoskeletal**	Fracture of wrist	fracture_wrist	BMI	obese	0.73	0.64	0.83	0.07	4.33E-06	150487	1656	6.95	1.33
**Musculoskeletal**	Gout	gout	BMI	overweight	2.12	1.87	2.39	0.06	1.90E-33	149466	2656	6.94	1.34
**Musculoskeletal**	Gout	gout	BMI	obese	4.05	3.58	4.57	0.06	3.57E-111	149466	2656	6.94	1.34
**Musculoskeletal**	Intervertebral disc disorders	intervert_disc	BMI	obese	1.6	1.45	1.77	0.05	1.42E-19	146841	2710	6.94	1.34
**Musculoskeletal**	Intervertebral disc disorders	intervert_disc	BMI	overweight	1.31	1.19	1.44	0.05	2.80E-08	146841	2710	6.94	1.34
**Musculoskeletal**	Osteoporosis	osteoporosis	BMI	obese	0.5	0.46	0.55	0.04	2.25E-55	149639	4270	6.92	1.41
**Musculoskeletal**	Osteoporosis	osteoporosis	BMI	overweight	0.65	0.61	0.7	0.03	2.52E-35	149639	4270	6.92	1.41
**Musculoskeletal**	Osteoporosis	osteoporosis	BMI	underweight	2.45	1.94	3.09	0.12	5.89E-14	149639	4270	6.92	1.41
**Musculoskeletal**	Spinal stenosis	spinal_stenosis	BMI	overweight	1.37	1.19	1.59	0.07	2.08E-05	153352	1299	6.95	1.33

**Musculoskeletal**	Spinal stenosis	spinal_stenosis	BMI	obese	2.16	1.86	2.51	0.08	5.28E-24	153352	1299	6.95	1.33
**Musculoskeletal**	Spondylolisthesis	spondylolisthesis	BMI	obese	1.74	1.37	2.21	0.12	5.00E-06	153400	447	6.96	1.31
**Musculoskeletal**	Spondylosis	spondylosis	BMI	obese	1.54	1.41	1.68	0.04	2.18E-22	145448	3556	6.92	1.4
**Neurological**	Bell's palsy	bells	BMI	obese	2.6	1.92	3.51	0.15	5.80E-10	153187	334	6.96	1.31
**Neurological**	Postviral fatigue syndrome, neurasthenia and fibromyalgia	chronic_fatigue	BMI	obese	1.9	1.59	2.26	0.09	7.53E-13	151083	840	6.95	1.32
**Neurological**	Diabetic neurological complications	dm_neuro	BMI	obese	7.09	4.51	11.14	0.23	2.28E-17	153829	221	6.98	1.31
**Neurological**	Peripheral neuropathies (excl. cranial nerve, carpal tunnel syndromes)	periph_neuro	BMI	obese	1.87	1.68	2.09	0.06	1.86E-28	151144	2225	6.94	1.33
**Neurological**	Peripheral neuropathies (excl. cranial nerve, carpal tunnel syndromes)	periph_neuro	BMI	overweight	1.25	1.12	1.39	0.06	5.37E-05	151144	2225	6.94	1.33
**Psychiatric**	Alcohol Problems	alc_problems	BMI	overweight	1.13	1.06	1.2	0.03	1.65E-04	151939	5819	6.89	1.42
**Psychiatric**	Alcohol Problems	alc_problems	BMI	obese	1.21	1.13	1.3	0.04	9.75E-08	151939	5819	6.89	1.42
**Psychiatric**	Anxiety disorders	anxiety	BMI	obese	1.31	1.2	1.43	0.05	2.51E-09	127169	3421	6.91	1.41
**Psychiatric**	Depression	depression	BMI	obese	1.65	1.5	1.81	0.05	1.45E-24	106004	3004	7	1.34
**Psychiatric**	Depression	depression	BMI	overweight	1.23	1.12	1.34	0.05	9.99E-06	106004	3004	7	1.34
**Respiratory**	COPD	COPD	BMI	underweight	3.82	2.85	5.1	0.15	1.55E-19	151345	3256	6.94	1.34

**Respiratory**	COPD	COPD	BMI	obese	1.23	1.13	1.35	0.05	5.60E-06	151345	3256	6.94	1.34
**Respiratory**	COPD_excl_bronchitis_NOS	COPD_excl_bronchitis_NOS	BMI	obese	1.22	1.12	1.34	0.05	1.39E-05	151407	3171	6.94	1.34
**Respiratory**	COPD_excl_bronchitis_NOS	COPD_excl_bronchitis_NOS	BMI	underweight	3.9	2.92	5.22	0.15	4.06E-20	151407	3171	6.94	1.34
**Respiratory**	Asthma	asthma	BMI	overweight	1.35	1.21	1.5	0.06	5.66E-08	133123	2221	6.94	1.33
**Respiratory**	Asthma	asthma	BMI	obese	2	1.79	2.24	0.06	7.06E-34	133123	2221	6.94	1.33
**Respiratory**	Bronchiectasis	bronchiectasis	BMI	underweight	4.94	2.97	8.22	0.26	7.19E-10	153099	734	6.96	1.32
**Respiratory**	Pleural effusion	pleural_effusion	BMI	obese	1.37	1.2	1.57	0.07	4.57E-06	153405	1481	6.96	1.32
**Respiratory**	Pulmonary collapse (excl pneumothorax)	pulm_collapse	BMI	obese	1.67	1.34	2.08	0.11	5.02E-06	153850	578	6.96	1.31
**Respiratory**	Other interstitial pulmonary diseases with fibrosis	pulm_fibrosis	BMI	underweight	7.3	3.35	15.9	0.4	5.48E-07	153842	330	6.96	1.31
**Respiratory**	Respiratory failure	resp_failure	BMI	underweight	8.08	4.89	13.34	0.26	3.17E-16	153944	719	6.96	1.31
**Respiratory**	Respiratory failure	resp_failure	BMI	obese	1.82	1.49	2.23	0.1	7.12E-09	153944	719	6.96	1.31
**Respiratory**	Sleep apnoea	sleep_apnoea	BMI	overweight	2.22	1.82	2.71	0.1	2.37E-15	152546	1400	6.95	1.32
**Respiratory**	Sleep apnoea	sleep_apnoea	BMI	obese	8	6.64	9.63	0.1	3.93E-106	152546	1400	6.95	1.32
**Skin**	Actinic keratosis	actinic_keratosis	BMI	obese	0.6	0.55	0.65	0.04	2.10E-33	151055	4592	6.92	1.42

**Skin**	Actinic keratosis	actinic_keratosis	BMI	overweight	0.79	0.74	0.85	0.03	4.33E-12	151055	4592	6.92	1.42
**Skin**	Dermatitis (atopc/contact/other/unspecified)	dermatitis	BMI	obese	1.22	1.15	1.3	0.03	1.66E-10	131299	7152	6.89	1.46
**Benign neoplasm or Carcinoma in situ**	Benign neoplasm of brain and other parts of central nervous system	benign_brain	BMI	overweight	0.89	0.68	1.17	0.14	4.06E-01	153561	310	6.96	1.31
**Benign neoplasm or Carcinoma in situ**	Benign neoplasm of brain and other parts of central nervous system	benign_brain	BMI	obese	1.13	0.84	1.51	0.15	4.16E-01	153561	310	6.96	1.31
**Benign neoplasm or Carcinoma in situ**	Benign neoplasm of brain and other parts of central nervous system	benign_brain	BMI	underweight	0	0	Inf	874.11	9.87E-01	153561	310	6.96	1.31
**Benign neoplasm or Carcinoma in situ**	Benign neoplasm of colon, rectum, anus and anal canal	benign_colon	BMI	underweight	1.03	0.69	1.54	0.21	8.93E-01	149732	6630	6.9	1.43
**Benign neoplasm or Carcinoma in situ**	Benign neoplasm of ovary	benign_ovary	BMI	underweight	0.65	0.24	1.74	0.5	3.88E-01	151115	802	6.96	1.32
**Benign neoplasm or Carcinoma in situ**	Benign neoplasm of ovary	benign_ovary	BMI	overweight	1.05	0.89	1.23	0.08	5.59E-01	151115	802	6.96	1.32

**Benign neoplasm or Carcinoma in situ**	Benign neoplasm of ovary	benign_ovary	BMI	obese	0.99	0.82	1.19	0.09	8.96E-01	151115	802	6.96	1.32
**Benign neoplasm or Carcinoma in situ**	Benign neoplasm of stomach and duodenum	benign_stomach	BMI	overweight	1.27	1.11	1.44	0.07	3.18E-04	153375	1456	6.95	1.33
**Benign neoplasm or Carcinoma in situ**	Benign neoplasm of stomach and duodenum	benign_stomach	BMI	underweight	0.66	0.25	1.76	0.5	4.05E-01	153375	1456	6.95	1.33
**Benign neoplasm or Carcinoma in situ**	Benign neoplasm and polyp of uterus	benign_uterus	BMI	overweight	1.24	1.07	1.43	0.07	3.84E-03	151265	1170	6.96	1.32
**Benign neoplasm or Carcinoma in situ**	Benign neoplasm and polyp of uterus	benign_uterus	BMI	underweight	0.71	0.29	1.71	0.45	4.45E-01	151265	1170	6.96	1.32
**Benign neoplasm or Carcinoma in situ**	Carcinoma in situ_cervical	cin_cervical	BMI	underweight	0.65	0.38	1.09	0.27	1.04E-01	135261	2395	6.96	1.36
**Benign neoplasm or Carcinoma in situ**	Carcinoma in situ_cervical	cin_cervical	BMI	overweight	0.93	0.84	1.02	0.05	1.11E-01	135261	2395	6.96	1.36

**Benign neoplasm or Carcinoma in situ**	Carcinoma in situ_cervical	cin_cervical	BMI	obese	0.98	0.88	1.09	0.05	7.26E-01	135261	2395	6.96	1.36
**Benign neoplasm or Carcinoma in situ**	Haemangioma, any site	haemangioma	BMI	overweight	1.07	0.92	1.26	0.08	3.64E-01	152349	877	6.96	1.32
**Benign neoplasm or Carcinoma in situ**	Haemangioma, any site	haemangioma	BMI	underweight	0.65	0.21	2.04	0.58	4.66E-01	152349	877	6.96	1.32
**Benign neoplasm or Carcinoma in situ**	Haemangioma, any site	haemangioma	BMI	obese	0.96	0.8	1.16	0.09	6.91E-01	152349	877	6.96	1.32
**Benign neoplasm or Carcinoma in situ**	Leiomyoma of uterus	leiomyoma	BMI	overweight	1.14	1.01	1.28	0.06	3.08E-02	146721	1558	6.95	1.33
**Benign neoplasm or Carcinoma in situ**	Leiomyoma of uterus	leiomyoma	BMI	underweight	0.75	0.39	1.45	0.34	3.90E-01	146721	1558	6.95	1.33
**Cancers**	Monoclonal gammopathy of undetermined significance (MGUS)	MGUS	BMI	underweight	1.81	0.44	7.38	0.72	4.08E-01	153949	235	6.96	1.31

**Cancers**	Monoclonal gammopathy of undetermined significance (MGUS)	MGUS	BMI	overweight	0.92	0.68	1.25	0.16	5.92E-01	153949	235	6.96	1.31
**Cancers**	Monoclonal gammopathy of undetermined significance (MGUS)	MGUS	BMI	obese	1.04	0.74	1.46	0.17	8.17E-01	153949	235	6.96	1.31
**Cancers**	Non-Hodgkin Lymphoma	NHL	BMI	overweight	0.95	0.77	1.18	0.11	6.69E-01	153610	493	6.96	1.31
**Cancers**	Non-Hodgkin Lymphoma	NHL	BMI	obese	1.05	0.83	1.33	0.12	6.72E-01	153610	493	6.96	1.31
**Cancers**	Non-Hodgkin Lymphoma	NHL	BMI	underweight	0.96	0.24	3.86	0.71	9.51E-01	153610	493	6.96	1.31
**Cancers**	Leukaemia	leukaemia	BMI	obese	1.04	0.77	1.41	0.15	7.77E-01	153820	317	6.96	1.31
**Cancers**	Leukaemia	leukaemia	BMI	underweight	0.82	0.11	5.86	1.01	8.40E-01	153820	317	6.96	1.31
**Cancers**	Leukaemia	leukaemia	BMI	overweight	0.99	0.76	1.29	0.14	9.22E-01	153820	317	6.96	1.31
**Cancers**	Multiple myeloma and malignant plasma cell neoplasms	plasmacell	BMI	overweight	0.77	0.55	1.07	0.17	1.15E-01	153970	198	6.98	1.31
**Cancers**	Multiple myeloma and malignant plasma cell neoplasms	plasmacell	BMI	obese	0.95	0.66	1.37	0.19	7.90E-01	153970	198	6.98	1.31
**Cancers**	Multiple myeloma and malignant plasma cell neoplasms	plasmacell	BMI	underweight	1.11	0.15	8.03	1.01	9.15E-01	153970	198	6.98	1.31
**Cancers**	Primary Malignancy_biliary tract	pri_biliary	BMI	underweight	2.59	0.35	19.05	1.02	3.51E-01	154049	103	6.98	1.31

**Cancers**	Primary Malignancy_biliary tract	pri_biliary	BMI	obese	1.28	0.76	2.17	0.27	3.54E-01	154049	103	6.98	1.31
**Cancers**	Primary Malignancy_biliary tract	pri_biliary	BMI	overweight	1.08	0.67	1.76	0.25	7.41E-01	154049	103	6.98	1.31
**Cancers**	Primary Malignancy_Bladder	pri_bladder	BMI	obese	1.42	1.1	1.82	0.13	6.42E-03	153595	496	6.96	1.31
**Cancers**	Primary Malignancy_Bladder	pri_bladder	BMI	overweight	1.23	0.98	1.55	0.12	7.44E-02	153595	496	6.96	1.31
**Cancers**	Primary Malignancy_Bladder	pri_bladder	BMI	underweigh t	0.76	0.11	5.47	1	7.88E-01	153595	496	6.96	1.31
**Cancers**	Primary Malignancy_colorectal and anus	pri_bowel	BMI	underweigh t	0.43	0.11	1.73	0.71	2.36E-01	153096	1198	6.96	1.32
**Cancers**	Primary Malignancy_colorectal and anus	pri_bowel	BMI	obese	1.26	1.08	1.46	0.08	3.38E-03	153096	1198	6.96	1.32
**Cancers**	Primary Malignancy_colorectal and anus	pri_bowel	BMI	overweight	1.04	0.91	1.2	0.07	5.39E-01	153096	1198	6.96	1.32
**Cancers**	Primary Malignancy_Brain, Other CNS and Intracranial	pri_brain	BMI	overweight	0.93	0.64	1.33	0.19	6.83E-01	153944	165	6.98	1.31
**Cancers**	Primary Malignancy_Brain, Other CNS and Intracranial	pri_brain	BMI	obese	0.99	0.66	1.49	0.21	9.54E-01	153944	165	6.98	1.31
**Cancers**	Primary Malignancy_Brain, Other CNS and Intracranial	pri_brain	BMI	underweigh t	0	0	Inf	1969.23	9.94E-01	153944	165	6.98	1.31

**Cancers**	Primary Malignancy_Breast	pri_breast	BMI	obese	1.2	1.07	1.34	0.06	2.19E-03	150427	2019	6.95	1.33
**Cancers**	Primary Malignancy_Breast	pri_breast	BMI	overweight	1.17	1.05	1.29	0.05	2.96E-03	150427	2019	6.95	1.33
**Cancers**	Primary Malignancy_Breast	pri_breast	BMI	underweight	0.98	0.57	1.7	0.28	9.52E-01	150427	2019	6.95	1.33
**Cancers**	Primary Malignancy_Kidney and Ureter	pri_kidney	BMI	overweight	1.24	0.93	1.66	0.15	1.37E-01	153828	331	6.96	1.31
**Cancers**	Primary Malignancy_Kidney and Ureter	pri_kidney	BMI	underweight	3.07	0.97	9.76	0.59	5.73E-02	153828	331	6.96	1.31
**Cancers**	Primary Malignancy_Lung and trachea	pri_lung	BMI	overweight	0.88	0.73	1.05	0.09	1.62E-01	153791	684	6.97	1.31
**Cancers**	Primary Malignancy_Lung and trachea	pri_lung	BMI	obese	1.05	0.86	1.28	0.1	6.46E-01	153791	684	6.97	1.31
**Cancers**	Primary Malignancy_Lung and trachea	pri_lung	BMI	underweight	0.96	0.31	2.99	0.58	9.37E-01	153791	684	6.97	1.31
**Cancers**	Primary Malignancy_Malignant Melanoma	pri_melanoma	BMI	obese	0.95	0.78	1.15	0.1	5.94E-01	152254	748	6.96	1.32
**Cancers**	Primary Malignancy_Malignant Melanoma	pri_melanoma	BMI	underweight	0.85	0.27	2.66	0.58	7.81E-01	152254	748	6.96	1.32
**Cancers**	Primary Malignancy_Malignant Melanoma	pri_melanoma	BMI	overweight	1	0.85	1.19	0.09	9.61E-01	152254	748	6.96	1.32
**Cancers**	Primary Malignancy_Oesophageal	pri_oesoph	BMI	obese	1.6	1.09	2.34	0.19	1.52E-02	153982	209	6.98	1.31

**Cancers**	Primary Malignancy_Oesophageal	pri_oesoph	BMI	underweight	5.61	1.74	18.07	0.6	3.89E-03	153982	209	6.98	1.31
**Cancers**	Primary Malignancy_Oesophageal	pri_oesoph	BMI	overweight	1.16	0.81	1.66	0.18	4.12E-01	153982	209	6.98	1.31
**Cancers**	Primary Malignancy_Oro-pharyngeal	pri_oroph	BMI	overweight	0.77	0.56	1.06	0.16	1.10E-01	153728	216	6.98	1.31
**Cancers**	Primary Malignancy_Oro-pharyngeal	pri_oroph	BMI	obese	0.86	0.6	1.23	0.18	4.04E-01	153728	216	6.98	1.31
**Cancers**	Primary Malignancy_Oro-pharyngeal	pri_oroph	BMI	underweight	3.07	0.96	9.74	0.59	5.75E-02	153728	216	6.98	1.31
**Cancers**	Primary Malignancy_Other Organs	pri_other	BMI	underweight	1.29	0.57	2.89	0.41	5.39E-01	153204	1085	6.96	1.31
**Cancers**	Primary Malignancy_Other Organs	pri_other	BMI	obese	1.16	0.99	1.36	0.08	6.71E-02	153204	1085	6.96	1.31
**Cancers**	Primary Malignancy_Other Organs	pri_other	BMI	overweight	1	0.87	1.16	0.07	9.54E-01	153204	1085	6.96	1.31
**Cancers**	Primary Malignancy_Ovarian	pri_ovarian	BMI	overweight	1.16	0.87	1.56	0.15	3.07E-01	153666	260	6.98	1.31
**Cancers**	Primary Malignancy_Ovarian	pri_ovarian	BMI	obese	1.39	1.02	1.9	0.16	3.91E-02	153666	260	6.98	1.31
**Cancers**	Primary Malignancy_Ovarian	pri_ovarian	BMI	underweight	1.26	0.31	5.1	0.72	7.50E-01	153666	260	6.98	1.31
**Cancers**	Primary Malignancy_Pancreatic	pri_pancr	BMI	obese	1.15	0.81	1.64	0.18	4.25E-01	154034	217	6.98	1.31

**Cancers**	Primary Malignancy_Pancreatic	pri_pancr	BMI	overweight	0.92	0.66	1.27	0.17	6.02E-01	154034	217	6.98	1.31
**Cancers**	Primary Malignancy_Pancreatic	pri_pancr	BMI	underweight	1.11	0.15	8.03	1.01	9.16E-01	154034	217	6.98	1.31
**Cancers**	Primary Malignancy_Prostate	pri_prost	BMI	overweight	1.05	0.94	1.18	0.06	3.59E-01	152908	1895	6.95	1.33
**Cancers**	Primary Malignancy_Prostate	pri_prost	BMI	obese	0.87	0.77	1	0.07	4.79E-02	152908	1895	6.95	1.33
**Cancers**	Primary Malignancy_Prostate	pri_prost	BMI	underweight	0.78	0.25	2.42	0.58	6.62E-01	152908	1895	6.95	1.33
**Cancers**	Primary Malignancy_Other Skin and subcutaneous tissue	pri_skin	BMI	overweight	0.9	0.84	0.96	0.03	1.68E-03	149842	4488	6.92	1.41
**Cancers**	Primary Malignancy_Other Skin and subcutaneous tissue	pri_skin	BMI	underweight	0.72	0.44	1.18	0.25	1.98E-01	149842	4488	6.92	1.41
**Cancers**	Primary Malignancy_Stomach	pri_stomach	BMI	obese	1.5	1.02	2.19	0.19	3.85E-02	153976	185	6.98	1.31
**Cancers**	Primary Malignancy_Stomach	pri_stomach	BMI	underweight	1.77	0.24	12.84	1.01	5.73E-01	153976	185	6.98	1.31
**Cancers**	Primary Malignancy_Stomach	pri_stomach	BMI	overweight	0.93	0.64	1.34	0.19	6.89E-01	153976	185	6.98	1.31
**Cancers**	Primary Malignancy_Uterine	pri_uterine	BMI	underweight	2.31	0.73	7.34	0.59	1.56E-01	153595	314	6.96	1.31
**Cancers**	Primary Malignancy_Uterine	pri_uterine	BMI	overweight	1.42	1.04	1.93	0.16	2.53E-02	153595	314	6.96	1.31

**Cancers**	Secondary Malignancy_Lymph Nodes	sec_LN	BMI	underweight	0.42	0.13	1.3	0.58	1.30E-01	153395	1508	6.96	1.32
**Cancers**	Secondary Malignancy_Lymph Nodes	sec_LN	BMI	obese	1.21	1.06	1.39	0.07	4.53E-03	153395	1508	6.96	1.32
**Cancers**	Secondary Malignancy_Lymph Nodes	sec_LN	BMI	overweight	1.03	0.91	1.17	0.06	5.92E-01	153395	1508	6.96	1.32
**Cancers**	Secondary Malignancy_Adrenal gland	sec_adrenal	BMI	obese	1.3	0.79	2.12	0.25	3.02E-01	154071	107	6.98	1.31
**Cancers**	Secondary Malignancy_Adrenal gland	sec_adrenal	BMI	overweight	0.86	0.53	1.38	0.24	5.27E-01	154071	107	6.98	1.31
**Cancers**	Secondary Malignancy_Adrenal gland	sec_adrenal	BMI	underweight	0	0	Inf	1498.12	9.93E-01	154071	107	6.98	1.31
**Cancers**	Secondary Malignancy_Bone	sec_bone	BMI	obese	1.28	1.06	1.55	0.1	1.17E-02	153994	756	6.98	1.31
**Cancers**	Secondary Malignancy_Bone	sec_bone	BMI	overweight	1.05	0.88	1.25	0.09	5.87E-01	153994	756	6.98	1.31
**Cancers**	Secondary Malignancy_Bone	sec_bone	BMI	underweight	0	0	Inf	556.76	9.80E-01	153994	756	6.98	1.31
**Cancers**	Secondary Malignancy_Brain, Other CNS and Intracranial	sec_brain	BMI	obese	1.11	0.83	1.49	0.15	4.65E-01	154057	319	6.98	1.31
**Cancers**	Secondary Malignancy_Brain, Other CNS and Intracranial	sec_brain	BMI	overweight	0.95	0.73	1.23	0.13	6.91E-01	154057	319	6.98	1.31

**Cancers**	Secondary Malignancy_Brain, Other CNS and Intracranial	sec_brain	BMI	underweight	0	0	Inf	1414.1	9.92E-01	154057	319	6.98	1.31
**Cancers**	Secondary malignancy_Liver and intrahepatic bile duct	sec_liver	BMI	underweight	1.87	0.88	3.97	0.38	1.02E-01	153975	865	6.98	1.31
**Cancers**	Secondary malignancy_Liver and intrahepatic bile duct	sec_liver	BMI	obese	1.2	1	1.43	0.09	4.73E-02	153975	865	6.98	1.31
**Cancers**	Secondary malignancy_Liver and intrahepatic bile duct	sec_liver	BMI	overweight	0.95	0.81	1.12	0.08	5.35E-01	153975	865	6.98	1.31
**Cancers**	Secondary Malignancy_Lung	sec_lung	BMI	obese	1.33	1.09	1.62	0.1	5.26E-03	154003	683	6.98	1.31
**Cancers**	Secondary Malignancy_Lung	sec_lung	BMI	underweight	0.71	0.18	2.85	0.71	6.28E-01	154003	683	6.98	1.31
**Cancers**	Secondary Malignancy_Lung	sec_lung	BMI	overweight	0.98	0.82	1.19	0.1	8.63E-01	154003	683	6.98	1.31
**Cancers**	Secondary Malignancy_Other organs	sec_other	BMI	overweight	0.96	0.8	1.15	0.09	6.33E-01	153903	701	6.97	1.31
**Cancers**	Secondary Malignancy_Other organs	sec_other	BMI	obese	1.19	0.98	1.45	0.1	7.82E-02	153903	701	6.97	1.31
**Cancers**	Secondary Malignancy_Other organs	sec_other	BMI	underweight	0.91	0.29	2.85	0.58	8.75E-01	153903	701	6.97	1.31
**Cancers**	Secondary Malignancy_retroperitoneum and peritoneum	sec_peritoneum	BMI	obese	1.18	0.93	1.5	0.12	1.80E-01	154025	454	6.98	1.31

**Cancers**	Secondary Malignancy_retroperitoneum and peritoneum	sec_peritoneum	BMI	underweight	1.32	0.42	4.15	0.58	6.31E-01	154025	454	6.98	1.31
**Cancers**	Secondary Malignancy_retroperitoneum and peritoneum	sec_peritoneum	BMI	overweight	0.96	0.77	1.21	0.11	7.51E-01	154025	454	6.98	1.31
**Cancers**	Secondary Malignancy_Pleura	sec_pleura	BMI	obese	1.16	0.82	1.63	0.18	4.04E-01	154060	218	6.98	1.31
**Cancers**	Secondary Malignancy_Pleura	sec_pleura	BMI	overweight	0.89	0.65	1.23	0.16	4.88E-01	154060	218	6.98	1.31
**Cancers**	Secondary Malignancy_Pleura	sec_pleura	BMI	underweight	0	0	Inf	1038.3	9.89E-01	154060	218	6.98	1.31
**Cardiovascular**	Abdominal aortic aneurysm	AAA	BMI	obese	1.57	1.14	2.18	0.17	6.44E-03	153866	329	6.97	1.31
**Cardiovascular**	Abdominal aortic aneurysm	AAA	BMI	overweight	1.5	1.12	2.02	0.15	7.01E-03	153866	329	6.97	1.31
**Cardiovascular**	Abdominal aortic aneurysm	AAA	BMI	underweight	4.66	1.46	14.87	0.59	9.36E-03	153866	329	6.97	1.31
**Cardiovascular**	Atrial fibrillation	AF	BMI	underweight	0.81	0.42	1.57	0.34	5.35E-01	150562	3624	6.93	1.34
**Cardiovascular**	Atrial fibrillation	AF	BMI	overweight	1.07	0.99	1.17	0.04	9.73E-02	150562	3624	6.93	1.34
**Cardiovascular**	Coronary heart disease not otherwise specified	CHD_NOS	BMI	underweight	1.52	0.92	2.49	0.25	9.89E-02	144817	3776	6.93	1.35
**Cardiovascular**	Intracerebral haemorrhage	Intracereb_haem	BMI	obese	1.22	0.86	1.73	0.18	2.61E-01	153816	226	6.98	1.31
**Cardiovascular**	Intracerebral haemorrhage	Intracereb_haem	BMI	overweight	0.95	0.69	1.32	0.17	7.74E-01	153816	226	6.98	1.31

**Cardiovascular**	Intracerebral haemorrhage	Intracereb_haem	BMI	underweight	0	0	Inf	1081.7	9.90E-01	153816	226	6.98	1.31
**Cardiovascular**	Ischaemic stroke	Isch_stroke	BMI	underweight	1.82	0.81	4.09	0.41	1.49E-01	153345	958	6.96	1.31
**Cardiovascular**	Ischaemic stroke	Isch_stroke	BMI	obese	1.28	1.08	1.52	0.09	4.75E-03	153345	958	6.96	1.31
**Cardiovascular**	Ischaemic stroke	Isch_stroke	BMI	overweight	1.02	0.87	1.19	0.08	8.25E-01	153345	958	6.96	1.31
**Cardiovascular**	Left bundle branch block	LBBB	BMI	underweight	2.71	1.11	6.62	0.46	2.90E-02	153851	574	6.96	1.31
**Cardiovascular**	Left bundle branch block	LBBB	BMI	overweight	1.26	1.02	1.56	0.11	3.08E-02	153851	574	6.96	1.31
**Cardiovascular**	Pulmonary embolism	PE	BMI	underweight	0.38	0.05	2.71	1	3.34E-01	152697	918	6.96	1.31
**Cardiovascular**	Right bundle branch block	RBBB	BMI	underweight	2.9	1.28	6.56	0.42	1.05E-02	153748	614	6.96	1.31
**Cardiovascular**	Right bundle branch block	RBBB	BMI	overweight	0.95	0.77	1.16	0.1	5.87E-01	153748	614	6.96	1.31
**Cardiovascular**	Right bundle branch block	RBBB	BMI	obese	1.34	1.09	1.66	0.11	5.95E-03	153748	614	6.96	1.31
**Cardiovascular**	Rheumatic valve dz	Rh_valve	BMI	underweight	2.13	0.29	15.57	1.02	4.58E-01	153877	171	6.98	1.31
**Cardiovascular**	Rheumatic valve dz	Rh_valve	BMI	overweight	1.44	0.94	2.19	0.22	9.18E-02	153877	171	6.98	1.31
**Cardiovascular**	Supraventricular tachycardia	SVT	BMI	underweight	2.07	0.85	5.04	0.45	1.10E-01	153125	517	6.96	1.31
**Cardiovascular**	Supraventricular tachycardia	SVT	BMI	obese	1.35	1.08	1.68	0.11	7.38E-03	153125	517	6.96	1.31
**Cardiovascular**	Supraventricular tachycardia	SVT	BMI	overweight	0.83	0.67	1.03	0.11	9.22E-02	153125	517	6.96	1.31

**Cardiovascular**	Stroke NOS	Stroke_NOS	BMI	obese	1.17	0.9	1.53	0.14	2.44E-01	151124	362	6.96	1.31
**Cardiovascular**	Stroke NOS	Stroke_NOS	BMI	underweight	0.68	0.09	4.87	1	7.00E-01	151124	362	6.96	1.31
**Cardiovascular**	Stroke NOS	Stroke_NOS	BMI	overweight	0.8	0.62	1.02	0.13	7.67E-02	151124	362	6.96	1.31
**Cardiovascular**	Subarachnoid haemorrhage	Subarach	BMI	overweight	0.88	0.63	1.22	0.17	4.52E-01	153669	189	6.98	1.31
**Cardiovascular**	Subarachnoid haemorrhage	Subarach	BMI	obese	0.71	0.47	1.06	0.21	9.24E-02	153669	189	6.98	1.31
**Cardiovascular**	Subarachnoid haemorrhage	Subarach	BMI	underweight	3.81	1.39	10.46	0.52	9.43E-03	153669	189	6.98	1.31
**Cardiovascular**	Transient ischaemic attack	TIA	BMI	underweight	0.4	0.1	1.59	0.71	1.93E-01	152422	1252	6.95	1.32
**Cardiovascular**	Transient ischaemic attack	TIA	BMI	obese	1.08	0.93	1.26	0.08	3.04E-01	152422	1252	6.95	1.32
**Cardiovascular**	Transient ischaemic attack	TIA	BMI	overweight	1.07	0.93	1.22	0.07	3.31E-01	152422	1252	6.95	1.32
**Cardiovascular**	Ventricular tachycardia	VT	BMI	underweight	2.87	0.7	11.79	0.72	1.43E-01	153897	224	6.98	1.31
**Cardiovascular**	Ventricular tachycardia	VT	BMI	obese	1.2	0.83	1.73	0.19	3.28E-01	153897	224	6.98	1.31
**Cardiovascular**	Ventricular tachycardia	VT	BMI	overweight	1.08	0.78	1.5	0.17	6.48E-01	153897	224	6.98	1.31
**Cardiovascular**	Atrioventricular block, first degree	av_block_1	BMI	overweight	1.36	1.05	1.77	0.13	1.97E-02	153969	428	6.96	1.31
**Cardiovascular**	Atrioventricular block, first degree	av_block_1	BMI	underweight	1.01	0.14	7.27	1.01	9.92E-01	153969	428	6.96	1.31
**Cardiovascular**	Atrioventricular block, second degree	av_block_2	BMI	obese	1.23	0.78	1.91	0.23	3.72E-01	154018	147	6.98	1.31

**Cardiovascular**	Atrioventricular block, second degree	av_block_2	BMI	underweight	2.15	0.29	15.7	1.01	4.50E-01	154018	147	6.98	1.31
**Cardiovascular**	Atrioventricular block, second degree	av_block_2	BMI	overweight	1.06	0.71	1.59	0.21	7.77E-01	154018	147	6.98	1.31
**Cardiovascular**	Atrioventricular block, complete	av_block_3	BMI	overweight	0.93	0.63	1.37	0.2	6.95E-01	153996	147	6.98	1.31
**Cardiovascular**	Atrioventricular block, complete	av_block_3	BMI	obese	1	0.64	1.55	0.22	9.87E-01	153996	147	6.98	1.31
**Cardiovascular**	Atrioventricular block, complete	av_block_3	BMI	underweight	0	0	Inf	2085.45	9.94E-01	153996	147	6.98	1.31
**Cardiovascular**	Other Cardiomyopathy	cardiomyo_oth	BMI	obese	1.53	1.13	2.06	0.15	5.57E-03	153857	312	6.96	1.31
**Cardiovascular**	Other Cardiomyopathy	cardiomyo_oth	BMI	overweight	1.08	0.81	1.43	0.15	6.11E-01	153857	312	6.96	1.31
**Cardiovascular**	Other Cardiomyopathy	cardiomyo_oth	BMI	underweight	0.99	0.14	7.09	1.01	9.89E-01	153857	312	6.96	1.31
**Cardiovascular**	Dilated cardiomyopathy	dcm	BMI	obese	1.75	1.18	2.6	0.2	5.24E-03	153932	183	6.98	1.31
**Cardiovascular**	Dilated cardiomyopathy	dcm	BMI	underweight	3.81	0.92	15.76	0.72	6.51E-02	153932	183	6.98	1.31
**Cardiovascular**	Dilated cardiomyopathy	dcm	BMI	overweight	1.08	0.73	1.58	0.2	7.10E-01	153932	183	6.98	1.31
**Cardiovascular**	Heart failure	hf	BMI	underweight	2.23	1.19	4.19	0.32	1.23E-02	152718	1836	6.95	1.32
**Cardiovascular**	Heart failure	hf	BMI	overweight	1.24	1.09	1.41	0.07	9.73E-04	152718	1836	6.95	1.32
**Cardiovascular**	Multiple valve dz	mult_valve	BMI	obese	1.52	1.2	1.92	0.12	4.20E-04	153909	518	6.96	1.31
**Cardiovascular**	Multiple valve dz	mult_valve	BMI	overweight	1.07	0.86	1.33	0.11	5.60E-01	153909	518	6.96	1.31

**Cardiovascular**	Multiple valve dz	mult_valve	BMI	underweight	2.4	0.89	6.51	0.51	8.43E-02	153909	518	6.96	1.31
**Cardiovascular**	Myocardial infarction	myocardial_infarction	BMI	obese	1.42	1.15	1.75	0.11	1.10E-03	147230	709	6.96	1.31
**Cardiovascular**	Myocardial infarction	myocardial_infarction	BMI	overweight	1.31	1.08	1.58	0.1	5.11E-03	147230	709	6.96	1.31
**Cardiovascular**	Myocardial infarction	myocardial_infarction	BMI	underweight	0	0	Inf	581.38	9.82E-01	147230	709	6.96	1.31
**Cardiovascular**	Nonrheumatic aortic valve disorders	nonRh_aortic	BMI	underweight	2.31	0.95	5.63	0.45	6.54E-02	153451	881	6.96	1.32
**Cardiovascular**	Nonrheumatic mitral valve disorders	nonRh_mitral	BMI	underweight	1.17	0.37	3.65	0.58	7.91E-01	153359	649	6.96	1.31
**Cardiovascular**	Nonrheumatic mitral valve disorders	nonRh_mitral	BMI	obese	1.2	0.98	1.47	0.11	8.48E-02	153359	649	6.96	1.31
**Cardiovascular**	Nonrheumatic mitral valve disorders	nonRh_mitral	BMI	overweight	1	0.83	1.21	0.1	9.68E-01	153359	649	6.96	1.31
**Cardiovascular**	Pericardial effusion (noninflammatory)	pericardial_effusion	BMI	obese	1.3	0.92	1.82	0.17	1.33E-01	153777	249	6.98	1.31
**Cardiovascular**	Pericardial effusion (noninflammatory)	pericardial_effusion	BMI	overweight	1.13	0.83	1.53	0.16	4.45E-01	153777	249	6.98	1.31
**Cardiovascular**	Pericardial effusion (noninflammatory)	pericardial_effusion	BMI	underweight	2.97	0.93	9.46	0.59	6.50E-02	153777	249	6.98	1.31
**Cardiovascular**	Peripheral arterial disease	peripheral_arterial_disease	BMI	overweight	0.92	0.78	1.08	0.08	3.03E-01	152668	959	6.96	1.31
**Cardiovascular**	Peripheral arterial disease	peripheral_arterial_disease	BMI	underweight	2.58	1.27	5.22	0.36	8.42E-03	152668	959	6.96	1.31
**Cardiovascular**	Primary pulmonary hypertension	prim_pulm_htn	BMI	underweight	6.35	1.94	20.75	0.6	2.24E-03	153998	134	6.98	1.31
**Cardiovascular**	Primary pulmonary hypertension	prim_pulm_htn	BMI	obese	1.83	1.17	2.86	0.23	8.60E-03	153998	134	6.98	1.31

**Cardiovascular**	Primary pulmonary hypertension	prim_pulm_htn	BMI	overweight	1.02	0.65	1.6	0.23	9.19E-01	153998	134	6.98	1.31
**Cardiovascular**	Secondary pulmonary hypertension	sec_pulm_htn	BMI	underweight	2.22	0.3	16.31	1.02	4.31E-01	154058	139	6.98	1.31
**Cardiovascular**	Secondary pulmonary hypertension	sec_pulm_htn	BMI	obese	2.18	1.4	3.38	0.22	5.33E-04	154058	139	6.98	1.31
**Cardiovascular**	Secondary pulmonary hypertension	sec_pulm_htn	BMI	overweight	1.06	0.68	1.67	0.23	7.89E-01	154058	139	6.98	1.31
**Cardiovascular**	Sick sinus syndrome	sick_sinus	BMI	overweight	1.41	0.92	2.17	0.22	1.13E-01	153979	141	6.98	1.31
**Cardiovascular**	Sick sinus syndrome	sick_sinus	BMI	obese	1.52	0.95	2.43	0.24	8.32E-02	153979	141	6.98	1.31
**Cardiovascular**	Sick sinus syndrome	sick_sinus	BMI	underweight	0	0	Inf	1292.09	9.92E-01	153979	141	6.98	1.31
**Cardiovascular**	Stable angina	stable_angina	BMI	underweight	0.67	0.3	1.5	0.41	3.32E-01	148093	3508	6.93	1.35
**Cardiovascular**	Unstable Angina	unstable_angina	BMI	overweight	1.32	1.11	1.57	0.09	1.42E-03	152534	963	6.96	1.32
**Cardiovascular**	Unstable Angina	unstable_angina	BMI	underweight	1.93	0.79	4.68	0.45	1.48E-01	152534	963	6.96	1.32
**Cardiovascular**	Venous thromboembolic disease (Excl PE)	vte_ex_pe	BMI	underweight	0.3	0.04	2.12	1	2.26E-01	150703	1091	6.96	1.32
**Digestive**	Gastro-oesophageal reflux disease	GORD	BMI	underweight	0.99	0.69	1.42	0.18	9.62E-01	141700	6892	6.88	1.43
**Digestive**	Inflammatory bowel disease (IBD)	IBD	BMI	overweight	1.29	1.02	1.63	0.12	3.08E-02	151928	441	6.96	1.31
**Digestive**	Inflammatory bowel disease (IBD)	IBD	BMI	underweight	1.73	0.55	5.43	0.59	3.51E-01	151928	441	6.96	1.31
**Digestive**	Inflammatory bowel disease (IBD)	IBD	BMI	obese	1.27	0.98	1.64	0.13	7.63E-02	151928	441	6.96	1.31

**Digestive**	Irritable bowel syndrome	IBS	BMI	obese	1.1	0.95	1.27	0.07	1.96E-01	147371	1325	6.93	1.32
**Digestive**	Irritable bowel syndrome	IBS	BMI	underweigh t	1.47	0.81	2.67	0.31	2.08E-01	147371	1325	6.93	1.32
**Digestive**	Irritable bowel syndrome	IBS	BMI	overweight	0.97	0.85	1.1	0.07	6.58E-01	147371	1325	6.93	1.32
**Digestive**	Anal fissure	anal_fissure	BMI	obese	1.21	1.04	1.42	0.08	1.65E-02	150960	1117	6.96	1.32
**Digestive**	Anal fissure	anal_fissure	BMI	overweight	1.06	0.92	1.22	0.07	4.33E-01	150960	1117	6.96	1.32
**Digestive**	Anal fissure	anal_fissure	BMI	underweigh t	0.95	0.39	2.31	0.45	9.18E-01	150960	1117	6.96	1.32
**Digestive**	Angiodysplasia of colon	angiodyspl asia_colon	BMI	overweight	1.27	0.78	2.07	0.25	3.33E-01	154022	131	6.98	1.31
**Digestive**	Angiodysplasia of colon	angiodyspl asia_colon	BMI	underweigh t	0	0	Inf	1418.88	9.92E-01	154022	131	6.98	1.31
**Digestive**	Anorectal fistula	anorectal_fi stula	BMI	overweight	1.55	1.11	2.18	0.17	1.04E-02	153236	247	6.96	1.31
**Digestive**	Anorectal fistula	anorectal_fi stula	BMI	underweigh t	1.36	0.19	9.87	1.01	7.59E-01	153236	247	6.96	1.31
**Digestive**	Anorectal prolapse	anorectal_p rolapse	BMI	obese	0.71	0.53	0.93	0.14	1.46E-02	153650	340	6.96	1.31
**Digestive**	Anorectal prolapse	anorectal_p rolapse	BMI	underweigh t	0.39	0.05	2.81	1	3.51E-01	153650	340	6.96	1.31
**Digestive**	Appendicitis	appendiciti s	BMI	obese	1.2	0.96	1.5	0.11	1.01E-01	146680	588	6.96	1.31
**Digestive**	Appendicitis	appendiciti s	BMI	overweight	1.17	0.96	1.42	0.1	1.15E-01	146680	588	6.96	1.31
**Digestive**	Appendicitis	appendiciti s	BMI	underweigh t	1.39	0.52	3.75	0.51	5.14E-01	146680	588	6.96	1.31
**Digestive**	Barrett's oesophagus	barretts	BMI	obese	1.24	1.03	1.49	0.09	2.21E-02	153220	932	6.96	1.32
**Digestive**	Barrett's oesophagus	barretts	BMI	overweight	1.35	1.15	1.58	0.08	2.91E-04	153220	932	6.96	1.32

**Digestive**	Barrett's oesophagus	barretts	BMI	underweight	0.63	0.16	2.54	0.71	5.17E-01	153220	932	6.96	1.32
**Digestive**	Cholangitis	cholangitis	BMI	underweight	2.35	0.32	17.25	1.02	4.01E-01	153950	223	6.98	1.31
**Digestive**	Cholecystitis	cholecystitis	BMI	underweight	0.82	0.31	2.2	0.5	6.97E-01	151776	1638	6.95	1.33
**Digestive**	Cholelithiasis	cholelithiasis	BMI	underweight	1.09	0.58	2.04	0.32	7.85E-01	148499	3014	6.93	1.34
**Digestive**	Liver fibrosis, sclerosis and cirrhosis	cirrhosis	BMI	overweight	1.07	0.82	1.39	0.14	6.37E-01	153646	402	6.96	1.31
**Digestive**	Liver fibrosis, sclerosis and cirrhosis	cirrhosis	BMI	underweight	0	0	Inf	804.08	9.86E-01	153646	402	6.96	1.31
**Digestive**	Coeliac disease	coeliac	BMI	overweight	0.82	0.61	1.1	0.15	1.87E-01	152543	244	6.97	1.31
**Digestive**	Coeliac disease	coeliac	BMI	obese	0.84	0.6	1.18	0.17	3.25E-01	152543	244	6.97	1.31
**Digestive**	Coeliac disease	coeliac	BMI	underweight	1.48	0.36	6	0.72	5.86E-01	152543	244	6.97	1.31
**Digestive**	Crohn's disease	crohns	BMI	obese	1.24	0.83	1.84	0.2	2.86E-01	153368	180	6.97	1.31
**Digestive**	Crohn's disease	crohns	BMI	overweight	1.15	0.8	1.64	0.18	4.45E-01	153368	180	6.97	1.31
**Digestive**	Crohn's disease	crohns	BMI	underweight	1.27	0.18	9.18	1.01	8.14E-01	153368	180	6.97	1.31
**Digestive**	Diverticular disease of intestine (acute and chronic)	diverticuli	BMI	underweight	0.63	0.39	1	0.24	4.77E-02	148653	7761	6.88	1.44
**Digestive**	Fatty Liver	fatty_liver	BMI	underweight	2.3	0.85	6.23	0.51	1.02E-01	153645	1367	6.96	1.32
**Digestive**	Gastritis and duodenitis	gastritis_duodenitis	BMI	underweight	1.16	0.83	1.62	0.17	3.79E-01	144186	7105	6.89	1.44
**Digestive**	Abdominal Hernia	hernia_abdo	BMI	obese	0.88	0.81	0.95	0.04	1.32E-03	143859	4763	6.92	1.41
**Digestive**	Abdominal Hernia	hernia_abdo	BMI	overweight	0.93	0.87	0.99	0.03	3.10E-02	143859	4763	6.92	1.41

**Digestive**	Abdominal Hernia	hernia_abdo	BMI	underweight	0.95	0.58	1.55	0.25	8.27E-01	143859	4763	6.92	1.41
**Digestive**	Diaphragmatic hernia	hernia_diaphragm	BMI	underweight	0.82	0.51	1.33	0.24	4.24E-01	146847	5267	6.9	1.42
**Digestive**	Alcoholic liver disease	liver_alc	BMI	obese	1.86	1.32	2.61	0.17	3.61E-04	153782	247	6.98	1.31
**Digestive**	Alcoholic liver disease	liver_alc	BMI	overweight	1.06	0.75	1.48	0.17	7.50E-01	153782	247	6.98	1.31
**Digestive**	Alcoholic liver disease	liver_alc	BMI	underweight	4.86	1.52	15.57	0.59	7.79E-03	153782	247	6.98	1.31
**Digestive**	Hepatic failure	liver_fail	BMI	overweight	1.22	0.82	1.83	0.21	3.26E-01	153934	167	6.98	1.31
**Digestive**	Hepatic failure	liver_fail	BMI	obese	1.85	1.22	2.81	0.21	3.67E-03	153934	167	6.98	1.31
**Digestive**	Hepatic failure	liver_fail	BMI	underweight	1.9	0.26	13.88	1.01	5.26E-01	153934	167	6.98	1.31
**Digestive**	Oesophagitis and oesophageal ulcer	oesoph_ulc	BMI	underweight	1	0.62	1.61	0.24	9.95E-01	146523	4429	6.92	1.41
**Digestive**	Pancreatitis	pancreatitis	BMI	underweight	3.93	1.73	8.96	0.42	1.12E-03	153343	460	6.96	1.31
**Digestive**	Pancreatitis	pancreatitis	BMI	overweight	1.21	0.95	1.54	0.12	1.29E-01	153343	460	6.96	1.31
**Digestive**	Peritonitis	peritonitis	BMI	obese	1.28	1.03	1.59	0.11	2.74E-02	153043	568	6.96	1.31
**Digestive**	Peritonitis	peritonitis	BMI	overweight	1.03	0.84	1.26	0.1	7.79E-01	153043	568	6.96	1.31
**Digestive**	Peritonitis	peritonitis	BMI	underweight	0.83	0.2	3.33	0.71	7.88E-01	153043	568	6.96	1.31
**Digestive**	Portal hypertension	portal_htn	BMI	obese	1.71	1.11	2.64	0.22	1.41E-02	154015	148	6.98	1.31
**Digestive**	Portal hypertension	portal_htn	BMI	underweight	4.3	1.03	17.9	0.73	4.51E-02	154015	148	6.98	1.31
**Digestive**	Portal hypertension	portal_htn	BMI	overweight	1.01	0.66	1.55	0.22	9.61E-01	154015	148	6.98	1.31
**Digestive**	Ulcerative colitis	ulc_colitis	BMI	underweight	2.28	0.72	7.21	0.59	1.61E-01	152450	337	6.96	1.31
**Digestive**	Ulcerative colitis	ulc_colitis	BMI	obese	1.21	0.89	1.64	0.15	2.23E-01	152450	337	6.96	1.31
**Digestive**	Ulcerative colitis	ulc_colitis	BMI	overweight	1.32	1.01	1.72	0.13	3.93E-02	152450	337	6.96	1.31

**Digestive**	Peptic ulcer disease	ulcer_peptic	BMI	overweight	1.19	1.04	1.36	0.07	1.39E-02	149047	1368	6.95	1.32
**Digestive**	Peptic ulcer disease	ulcer_peptic	BMI	underweight	2.31	1.26	4.2	0.31	6.45E-03	149047	1368	6.95	1.32
**Digestive**	Oesophageal varices	varices	BMI	underweight	1.86	0.26	13.56	1.01	5.40E-01	153922	173	6.98	1.31
**Digestive**	Oesophageal varices	varices	BMI	overweight	1.12	0.75	1.68	0.21	5.71E-01	153922	173	6.98	1.31
**Digestive**	Volvulus	volvulus	BMI	obese	1.02	0.71	1.47	0.19	9.16E-01	153952	179	6.97	1.31
**Digestive**	Volvulus	volvulus	BMI	underweight	0.93	0.13	6.73	1.01	9.46E-01	153952	179	6.97	1.31
**Digestive**	Volvulus	volvulus	BMI	overweight	0.62	0.44	0.89	0.18	9.68E-03	153952	179	6.97	1.31
**Ear**	Hearing loss	deaf	BMI	underweight	1.25	0.88	1.77	0.18	2.13E-01	140976	5897	6.9	1.43
**Ear**	Hearing loss	deaf	BMI	obese	1.11	1.04	1.19	0.04	2.68E-03	140976	5897	6.9	1.43
**Ear**	Hearing loss	deaf	BMI	overweight	1.01	0.95	1.08	0.03	7.30E-01	140976	5897	6.9	1.43
**Ear**	Meniere disease	meniere	BMI	underweight	3.52	1.28	9.65	0.51	1.45E-02	153131	197	6.96	1.31
**Ear**	Meniere disease	meniere	BMI	overweight	0.87	0.62	1.21	0.17	4.00E-01	153131	197	6.96	1.31
**Ear**	Meniere disease	meniere	BMI	obese	1.03	0.71	1.49	0.19	8.77E-01	153131	197	6.96	1.31
**Ear**	Tinnitus	tinnitus	BMI	obese	0.92	0.82	1.03	0.06	1.53E-01	137398	2177	7.04	1.31
**Ear**	Tinnitus	tinnitus	BMI	overweight	1.04	0.95	1.15	0.05	3.84E-01	137398	2177	7.04	1.31
**Ear**	Tinnitus	tinnitus	BMI	underweight	1.09	0.62	1.93	0.29	7.65E-01	137398	2177	7.04	1.31
**Endocrine**	Hyperparathyroidism	PTH	BMI	underweight	2.13	0.79	5.79	0.51	1.37E-01	153837	399	6.96	1.31
**Endocrine**	Hyperparathyroidism	PTH	BMI	overweight	1.13	0.88	1.46	0.13	3.22E-01	153837	399	6.96	1.31
**Endocrine**	Diabetes NOS	diabetes_nos	BMI	underweight	1.04	0.14	7.49	1.01	9.69E-01	147594	655	6.98	1.31
**Endocrine**	Diabetes Type I	diabetes_t1	BMI	overweight	1.02	0.77	1.36	0.15	8.75E-01	153297	421	6.96	1.31

**Endocrine**	Diabetes Type I	diabetes_t1	BMI	underweight	2.73	0.86	8.65	0.59	8.82E-02	153297	421	6.96	1.31
**Endocrine**	Diabetes Type II	diabetes_t2	BMI	underweight	1.05	0.47	2.35	0.41	9.08E-01	149744	4989	6.91	1.41
**Endocrine**	Hyperthyroidism	hyperthyroid	BMI	overweight	1.05	0.86	1.29	0.1	6.18E-01	151683	536	6.96	1.32
**Endocrine**	Hyperthyroidism	hyperthyroid	BMI	obese	1.35	1.09	1.68	0.11	6.65E-03	151683	536	6.96	1.32
**Endocrine**	Hyperthyroidism	hyperthyroid	BMI	underweight	0.99	0.32	3.1	0.58	9.85E-01	151683	536	6.96	1.32
**Endocrine**	Hypothyroidism	hypothyroid	BMI	underweight	1.37	0.79	2.38	0.28	2.62E-01	144663	1962	6.95	1.33
**Endocrine**	Obesity	obesity	BMI	underweight	0.72	0.1	5.2	1.01	7.48E-01	144148	6009	6.88	1.43
**Endocrine**	Hypo or hyperthyroidism	thyroid	BMI	overweight	1.18	1.06	1.32	0.06	2.50E-03	142846	1971	6.94	1.33
**Endocrine**	Hypo or hyperthyroidism	thyroid	BMI	underweight	1.58	0.96	2.59	0.25	7.17E-02	142846	1971	6.94	1.33
**Endocrine**	Thyroiditis unspecified	thyroid_nos	BMI	obese	1.44	0.88	2.35	0.25	1.51E-01	151208	114	6.96	1.31
**Endocrine**	Thyroiditis unspecified	thyroid_nos	BMI	overweight	1.3	0.83	2.04	0.23	2.51E-01	151208	114	6.96	1.31
**Endocrine**	Thyroiditis unspecified	thyroid_nos	BMI	underweight	1.73	0.24	12.67	1.02	5.88E-01	151208	114	6.96	1.31
**Eye**	Anterior and Intermediate Uveitis	ant_uveitis	BMI	obese	1.26	0.93	1.69	0.15	1.32E-01	153081	290	6.96	1.31
**Eye**	Anterior and Intermediate Uveitis	ant_uveitis	BMI	overweight	0.94	0.71	1.25	0.14	6.80E-01	153081	290	6.96	1.31
**Eye**	Anterior and Intermediate Uveitis	ant_uveitis	BMI	underweight	1.33	0.33	5.4	0.71	6.91E-01	153081	290	6.96	1.31
**Eye**	Visual impairment and blindness	blind	BMI	obese	1.36	1.06	1.76	0.13	1.67E-02	153508	407	6.96	1.31

**Eye**	Visual impairment and blindness	blind	BMI	overweight	0.96	0.75	1.22	0.12	7.16E-01	153508	407	6.96	1.31
**Eye**	Visual impairment and blindness	blind	BMI	underweight	0	0	Inf	765.05	9.86E-01	153508	407	6.96	1.31
**Eye**	Cataract	cataract	BMI	overweight	1.06	1	1.12	0.03	4.18E-02	148730	7052	6.9	1.43
**Eye**	Cataract	cataract	BMI	underweight	1.34	0.97	1.83	0.16	7.37E-02	148730	7052	6.9	1.43
**Eye**	Diabetic ophthalmic complications	diab_eye	BMI	underweight	0.77	0.25	2.41	0.58	6.55E-01	151709	2959	6.94	1.34
**Eye**	Glaucoma	glaucoma	BMI	obese	0.86	0.75	0.98	0.07	2.13E-02	150951	1683	6.95	1.33
**Eye**	Glaucoma	glaucoma	BMI	overweight	0.88	0.79	0.98	0.06	2.52E-02	150951	1683	6.95	1.33
**Eye**	Glaucoma	glaucoma	BMI	underweight	1.32	0.73	2.39	0.3	3.65E-01	150951	1683	6.95	1.33
**Eye**	Macular degeneration	macula_degen	BMI	obese	1.3	1.11	1.53	0.08	1.06E-03	152253	1080	6.96	1.32
**Eye**	Macular degeneration	macula_degen	BMI	underweight	1.48	0.7	3.14	0.38	3.02E-01	152253	1080	6.96	1.32
**Eye**	Macular degeneration	macula_degen	BMI	overweight	1.03	0.89	1.19	0.07	7.09E-01	152253	1080	6.96	1.32
**Eye**	Ptosis of eyelid	ptosis	BMI	underweight	0.5	0.07	3.59	1	4.93E-01	153571	429	6.96	1.31
**Eye**	Ptosis of eyelid	ptosis	BMI	obese	1.26	0.97	1.63	0.13	8.03E-02	153571	429	6.96	1.31
**Eye**	Ptosis of eyelid	ptosis	BMI	overweight	1.22	0.97	1.53	0.12	9.19E-02	153571	429	6.96	1.31
**Eye**	Retinal detachments and breaks	retinal_detach	BMI	obese	0.86	0.71	1.05	0.1	1.43E-01	152795	736	6.96	1.32
**Eye**	Retinal detachments and breaks	retinal_detach	BMI	overweight	0.93	0.78	1.1	0.09	3.79E-01	152795	736	6.96	1.32
**Eye**	Retinal detachments and breaks	retinal_detach	BMI	underweight	0.84	0.27	2.62	0.58	7.64E-01	152795	736	6.96	1.32

**Eye**	Retinal vascular occlusions	retinal_vasc_occl	BMI	obese	1.39	1.06	1.83	0.14	1.67E-02	153633	411	6.96	1.31
**Eye**	Retinal vascular occlusions	retinal_vasc_occl	BMI	overweight	1.32	1.03	1.68	0.13	2.81E-02	153633	411	6.96	1.31
**Eye**	Retinal vascular occlusions	retinal_vasc_occl	BMI	underweight	3.55	1.44	8.72	0.46	5.80E-03	153633	411	6.96	1.31
**Eye**	Scleritis and episcleritis	scleritis	BMI	underweight	1.67	0.23	12.2	1.01	6.13E-01	153754	102	6.98	1.31
**Eye**	Scleritis and episcleritis	scleritis	BMI	obese	1.03	0.62	1.73	0.26	9.04E-01	153754	102	6.98	1.31
**Eye**	Scleritis and episcleritis	scleritis	BMI	overweight	0.98	0.62	1.55	0.23	9.45E-01	153754	102	6.98	1.31
**Genitourinary**	Acute Kidney Injury	AKI	BMI	overweight	1.26	1.1	1.44	0.07	7.11E-04	153834	1770	6.96	1.32
**Genitourinary**	Acute Kidney Injury	AKI	BMI	underweight	0.91	0.34	2.43	0.5	8.45E-01	153834	1770	6.96	1.32
**Genitourinary**	Hyperplasia of prostate	BPH	BMI	obese	1.12	1.03	1.23	0.05	1.21E-02	149793	3910	6.92	1.4
**Genitourinary**	Hyperplasia of prostate	BPH	BMI	overweight	1.11	1.02	1.2	0.04	1.42E-02	149793	3910	6.92	1.4
**Genitourinary**	Hyperplasia of prostate	BPH	BMI	underweight	1.44	0.79	2.6	0.3	2.33E-01	149793	3910	6.92	1.4
**Genitourinary**	Chronic Kidney Disease	CKD	BMI	underweight	0.96	0.45	2.02	0.38	9.06E-01	152307	2647	6.93	1.33
**Genitourinary**	Erectile dysfunction	ED	BMI	underweight	0.64	0.29	1.44	0.41	2.83E-01	147822	5621	6.91	1.43
**Genitourinary**	End stage renal disease	ESRD	BMI	overweight	1.2	0.81	1.78	0.2	3.60E-01	153721	198	6.98	1.31
**Genitourinary**	End stage renal disease	ESRD	BMI	underweight	1.85	0.25	13.45	1.01	5.45E-01	153721	198	6.98	1.31

**Genitourinary**	Glomerulonephritis	GN	BMI	underweight	2.39	0.76	7.56	0.59	1.38E-01	153431	428	6.96	1.31
**Genitourinary**	Glomerulonephritis	GN	BMI	overweight	1.2	0.92	1.57	0.14	1.76E-01	153431	428	6.96	1.31
**Genitourinary**	Postcoital and contact bleeding	PCB	BMI	underweight	2.46	1.21	4.99	0.36	1.32E-02	152510	336	6.96	1.31
**Genitourinary**	Postcoital and contact bleeding	PCB	BMI	obese	0.62	0.45	0.85	0.16	2.77E-03	152510	336	6.96	1.31
**Genitourinary**	Postcoital and contact bleeding	PCB	BMI	overweight	0.91	0.71	1.15	0.12	4.32E-01	152510	336	6.96	1.31
**Genitourinary**	Postmenopausal bleeding	PMB	BMI	overweight	1.12	1.01	1.24	0.05	2.49E-02	148776	2149	6.95	1.33
**Genitourinary**	Postmenopausal bleeding	PMB	BMI	underweight	0.48	0.23	1.02	0.38	5.66E-02	148776	2149	6.95	1.33
**Genitourinary**	Tubulo-interstitial nephritis	TIN	BMI	overweight	1.31	0.92	1.86	0.18	1.35E-01	153859	201	6.98	1.31
**Genitourinary**	Tubulo-interstitial nephritis	TIN	BMI	obese	1.59	1.09	2.32	0.19	1.56E-02	153859	201	6.98	1.31
**Genitourinary**	Tubulo-interstitial nephritis	TIN	BMI	underweight	1.14	0.16	8.24	1.01	8.98E-01	153859	201	6.98	1.31
**Genitourinary**	Non-acute cystitis	chr_cystitis	BMI	underweight	2.17	0.53	8.87	0.72	2.83E-01	153788	217	6.97	1.31
**Genitourinary**	Non-acute cystitis	chr_cystitis	BMI	overweight	1.18	0.84	1.64	0.17	3.35E-01	153788	217	6.97	1.31
**Genitourinary**	Non-acute cystitis	chr_cystitis	BMI	obese	1.47	1.03	2.1	0.18	3.42E-02	153788	217	6.97	1.31
**Genitourinary**	Dysmenorrhoea	dysmenorrhoea	BMI	overweight	1.66	1.2	2.29	0.17	2.23E-03	151328	183	6.98	1.31
**Genitourinary**	Dysmenorrhoea	dysmenorrhoea	BMI	underweight	0.68	0.09	4.88	1.01	7.00E-01	151328	183	6.98	1.31

**Genitourinary**	Dysmenorrhoea	dysmenorrhoea	BMI	obese	1.07	0.7	1.63	0.21	7.42E-01	151328	183	6.98	1.31
**Genitourinary**	Endometrial hyperplasia and hypertrophy	endometrial_hyper	BMI	overweight	1.47	1.07	2.01	0.16	1.65E-02	153321	259	6.96	1.31
**Genitourinary**	Endometrial hyperplasia and hypertrophy	endometrial_hyper	BMI	underweight	0	0	Inf	8097.46	9.98E-01	153321	259	6.96	1.31
**Genitourinary**	Endometriosis	endometriosis	BMI	obese	1.55	1.17	2.05	0.14	2.32E-03	151057	320	6.96	1.32
**Genitourinary**	Endometriosis	endometriosis	BMI	overweight	1.32	1.01	1.71	0.13	3.97E-02	151057	320	6.96	1.32
**Genitourinary**	Endometriosis	endometriosis	BMI	underweight	0.9	0.22	3.66	0.71	8.88E-01	151057	320	6.96	1.32
**Genitourinary**	Female genital prolapse	female_genital_prolapse	BMI	underweight	0.11	0.03	0.43	0.71	1.70E-03	148909	2832	6.94	1.35
**Genitourinary**	Hydrocoele (incl infected)	hydrocele	BMI	obese	1.38	1.07	1.79	0.13	1.48E-02	153146	440	6.96	1.31
**Genitourinary**	Hydrocoele (incl infected)	hydrocele	BMI	underweight	1.13	0.16	8.08	1.01	9.06E-01	153146	440	6.96	1.31
**Genitourinary**	Hydrocoele (incl infected)	hydrocele	BMI	overweight	0.99	0.78	1.27	0.12	9.67E-01	153146	440	6.96	1.31
**Genitourinary**	Menorrhagia and polymenorrhoea	menorrhagia	BMI	overweight	1.24	1.11	1.4	0.06	2.59E-04	142650	1520	6.95	1.33
**Genitourinary**	Menorrhagia and polymenorrhoea	menorrhagia	BMI	underweight	0.51	0.24	1.07	0.38	7.67E-02	142650	1520	6.95	1.33
**Genitourinary**	Neuromuscular dysfunction of bladder	neuro_bladder	BMI	underweight	0.22	0.03	1.56	1	1.29E-01	152401	1133	6.95	1.32
**Genitourinary**	Neuromuscular dysfunction of bladder	neuro_bladder	BMI	overweight	1.29	1.11	1.5	0.08	7.48E-04	152401	1133	6.95	1.32

**Genitourinary**	Obstructive and reflux uropathy	obstr_reflux	BMI	underweight	1.91	0.79	4.66	0.45	1.52E-01	153469	776	6.96	1.31
**Genitourinary**	Obstructive and reflux uropathy	obstr_reflux	BMI	overweight	1.18	0.98	1.42	0.09	7.36E-02	153469	776	6.96	1.31
**Genitourinary**	Urinary Incontinence	urine_incont	BMI	underweight	0.92	0.55	1.54	0.26	7.54E-01	148453	3185	6.93	1.35
**Genitourinary**	Urolithiasis	urolithiasis	BMI	overweight	1.19	1.03	1.37	0.07	1.54E-02	150319	1327	6.95	1.33
**Genitourinary**	Urolithiasis	urolithiasis	BMI	underweight	1.86	0.92	3.75	0.36	8.40E-02	150319	1327	6.95	1.33
**Haematological or immunological**	Iron deficiency anaemia	IDA	BMI	underweight	1.61	1.01	2.56	0.24	4.73E-02	150047	2610	6.94	1.34
**Haematological or immunological**	Iron deficiency anaemia	IDA	BMI	overweight	1.02	0.92	1.12	0.05	7.34E-01	150047	2610	6.94	1.34
**Haematological or immunological**	Agranulocytosis	agranulocytosis	BMI	obese	0.82	0.71	0.96	0.08	1.21E-02	153144	1229	6.96	1.32
**Haematological or immunological**	Agranulocytosis	agranulocytosis	BMI	underweight	1.26	0.65	2.43	0.34	4.95E-01	153144	1229	6.96	1.32
**Haematological or immunological**	Agranulocytosis	agranulocytosis	BMI	overweight	0.84	0.74	0.96	0.07	9.31E-03	153144	1229	6.96	1.32
**Haematological or**	Aplastic anaemias	aplastic	BMI	overweight	0.74	0.48	1.14	0.22	1.70E-01	154007	133	6.98	1.31

**immunological**													
**Haematological or immunological**	Aplastic anaemias	aplastic	BMI	obese	1.33	0.86	2.05	0.22	2.00E-01	154007	133	6.98	1.31
**Haematological or immunological**	Aplastic anaemias	aplastic	BMI	underweight	2.04	0.28	14.9	1.01	4.81E-01	154007	133	6.98	1.31
**Haematological or immunological**	Vitamin B12 deficiency anaemia	b12_def	BMI	underweight	1.91	0.95	3.86	0.36	7.14E-02	152741	1071	6.96	1.32
**Haematological or immunological**	Vitamin B12 deficiency anaemia	b12_def	BMI	overweight	0.97	0.83	1.14	0.08	7.47E-01	152741	1071	6.96	1.32
**Haematological or immunological**	Folate deficiency anaemia	folatedef	BMI	overweight	0.82	0.63	1.07	0.13	1.51E-01	153895	352	6.96	1.31
**Haematological or immunological**	Folate deficiency anaemia	folatedef	BMI	obese	1.38	1.06	1.8	0.14	1.67E-02	153895	352	6.96	1.31
**Haematological or immunological**	Folate deficiency anaemia	folatedef	BMI	underweight	1.88	0.6	5.92	0.59	2.82E-01	153895	352	6.96	1.31
**Haematological or immunological**	Other anaemias	oth_anaemia	BMI	overweight	1.03	0.95	1.12	0.04	4.84E-01	148350	3670	6.93	1.34

**Haematological or immunological**	Primary or Idiopathic Thrombocytopaenia	pri_thrombocytopaenia	BMI	overweight	1.18	0.85	1.63	0.16	3.18E-01	153669	255	6.96	1.31
**Haematological or immunological**	Primary or Idiopathic Thrombocytopaenia	pri_thrombocytopaenia	BMI	obese	1.8	1.29	2.51	0.17	4.88E-04	153669	255	6.96	1.31
**Haematological or immunological**	Primary or Idiopathic Thrombocytopaenia	pri_thrombocytopaenia	BMI	underweight	1.19	0.16	8.58	1.01	8.65E-01	153669	255	6.96	1.31
**Haematological or immunological**	Secondary or other Thrombocytopaenia	sec_oth_thrombocytopaenia	BMI	obese	1.44	1.15	1.79	0.11	1.27E-03	153665	530	6.96	1.31
**Haematological or immunological**	Secondary or other Thrombocytopaenia	sec_oth_thrombocytopaenia	BMI	overweight	0.91	0.74	1.13	0.11	4.12E-01	153665	530	6.96	1.31
**Haematological or immunological**	Secondary or other Thrombocytopaenia	sec_oth_thrombocytopaenia	BMI	underweight	0.48	0.07	3.43	1	4.65E-01	153665	530	6.96	1.31
**Haematological or immunological**	Splenomegaly	splenomegaly	BMI	overweight	1.18	0.74	1.86	0.23	4.89E-01	153969	129	6.98	1.31
**Haematological or immunological**	Splenomegaly	splenomegaly	BMI	obese	1.91	1.2	3.06	0.24	6.85E-03	153969	129	6.98	1.31
**Haematological or**	Splenomegaly	splenomegaly	BMI	underweight	0	0	Inf	2306.05	9.95E-01	153969	129	6.98	1.31

**immunological**													
**Haematological or immunological**	Thrombophilia	thrombophilia	BMI	overweight	1.22	0.85	1.77	0.19	2.83E-01	153732	174	6.98	1.31
**Haematological or immunological**	Thrombophilia	thrombophilia	BMI	obese	1.5	1.01	2.23	0.2	4.58E-02	153732	174	6.98	1.31
**Haematological or immunological**	Thrombophilia	thrombophilia	BMI	underweight	1.26	0.17	9.14	1.01	8.19E-01	153732	174	6.98	1.31
**Infections**	Female pelvic inflammatory disease	PID	BMI	overweight	1.19	0.94	1.5	0.12	1.44E-01	152924	420	6.96	1.31
**Infections**	Female pelvic inflammatory disease	PID	BMI	underweight	1.44	0.53	3.9	0.51	4.68E-01	152924	420	6.96	1.31
**Infections**	Infection of anal and rectal regions	anorectal	BMI	overweight	1.34	0.9	2	0.2	1.53E-01	153653	189	6.98	1.31
**Infections**	Infection of anal and rectal regions	anorectal	BMI	underweight	3.66	0.88	15.22	0.73	7.39E-02	153653	189	6.98	1.31
**Infections**	Infection of bones and joints	bone	BMI	overweight	1.1	0.76	1.58	0.19	6.16E-01	153667	235	6.98	1.31
**Infections**	Infection of bones and joints	bone	BMI	underweight	1.65	0.23	11.95	1.01	6.22E-01	153667	235	6.98	1.31
**Infections**	Infections of the digestive system	digestive	BMI	underweight	1.6	1.06	2.42	0.21	2.62E-02	152729	3297	6.94	1.34
**Infections**	Infections of the digestive system	digestive	BMI	overweight	1.08	1	1.18	0.04	6.16E-02	152729	3297	6.94	1.34

**Infections**	Ear and Upper Respiratory Tract Infections	ear_urti	BMI	overweight	1.17	1	1.37	0.08	4.81E-02	150069	940	6.98	1.31
**Infections**	Ear and Upper Respiratory Tract Infections	ear_urti	BMI	underweigh t	1.2	0.49	2.9	0.45	6.92E-01	150069	940	6.98	1.31
**Infections**	Eye infections	eye	BMI	underweigh t	3.13	0.76	12.95	0.72	1.15E-01	153828	124	6.98	1.31
**Infections**	Eye infections	eye	BMI	overweight	0.69	0.45	1.05	0.22	8.63E-02	153828	124	6.98	1.31
**Infections**	Eye infections	eye	BMI	obese	1.01	0.65	1.58	0.23	9.64E-01	153828	124	6.98	1.31
**Infections**	Infection of liver	liver	BMI	underweigh t	2.39	0.32	17.57	1.02	3.92E-01	153413	114	6.98	1.31
**Infections**	Infection of liver	liver	BMI	obese	1.94	1.2	3.14	0.24	6.56E-03	153413	114	6.98	1.31
**Infections**	Infection of liver	liver	BMI	overweight	1.03	0.63	1.67	0.25	9.17E-01	153413	114	6.98	1.31
**Infections**	Lower Respiratory Tract Infections	lrti	BMI	overweight	1.07	0.99	1.16	0.04	8.83E-02	149287	4282	6.94	1.34
**Infections**	Infection of male genital system	male_GU	BMI	overweight	1.17	0.82	1.65	0.18	3.82E-01	153788	231	6.96	1.31
**Infections**	Infection of male genital system	male_GU	BMI	underweigh t	2.42	0.33	17.57	1.01	3.82E-01	153788	231	6.96	1.31
**Infections**	Infection of male genital system	male_GU	BMI	obese	1.63	1.13	2.36	0.19	9.04E-03	153788	231	6.96	1.31
**Infections**	Mycoses	mycoses	BMI	obese	1.15	0.95	1.39	0.1	1.44E-01	153774	753	6.96	1.31
**Infections**	Mycoses	mycoses	BMI	overweight	0.92	0.77	1.1	0.09	3.51E-01	153774	753	6.96	1.31
**Infections**	Mycoses	mycoses	BMI	underweigh t	2.65	1.36	5.17	0.34	4.10E-03	153774	753	6.96	1.31
**Infections**	Other nervous system infections	oth_nerv_s ys	BMI	obese	1.39	0.87	2.22	0.24	1.69E-01	153930	129	6.98	1.31
**Infections**	Other nervous system infections	oth_nerv_s ys	BMI	overweight	1.11	0.72	1.72	0.22	6.28E-01	153930	129	6.98	1.31

**Infections**	Other nervous system infections	oth_nerv_sys	BMI	underweight	3.84	0.92	16.01	0.73	6.45E-02	153930	129	6.98	1.31
**Infections**	Other or unspecified infectious organisms	oth_organisms	BMI	overweight	1.07	1.01	1.13	0.03	2.35E-02	149636	8008	6.89	1.43
**Infections**	Infections of Other or unspecified organs	oth_organs	BMI	overweight	1.09	1.01	1.19	0.04	3.53E-02	150859	3565	6.94	1.34
**Infections**	Rheumatic fever	rh_fever	BMI	overweight	1.33	0.87	2.03	0.22	1.86E-01	153241	167	6.97	1.31
**Infections**	Rheumatic fever	rh_fever	BMI	underweight	2.06	0.28	15.05	1.02	4.78E-01	153241	167	6.97	1.31
**Infections**	Septicaemia	sepsis	BMI	underweight	1.69	0.87	3.28	0.34	1.20E-01	153580	1379	6.96	1.32
**Infections**	Septicaemia	sepsis	BMI	overweight	0.97	0.85	1.11	0.07	6.57E-01	153580	1379	6.96	1.32
**Infections**	Infection of skin and subcutaneous tissues	skin	BMI	overweight	1.17	1.04	1.31	0.06	6.96E-03	151756	2174	6.95	1.33
**Infections**	Infection of skin and subcutaneous tissues	skin	BMI	underweight	1	0.48	2.12	0.38	9.94E-01	151756	2174	6.95	1.33
**Infections**	Urinary Tract Infections	uti	BMI	overweight	1.12	1.03	1.23	0.05	1.19E-02	151487	3064	6.94	1.33
**Infections**	Viral diseases (excl chronic hepatitis/HIV)	viral	BMI	underweight	1.41	0.67	2.99	0.38	3.64E-01	147978	1102	6.96	1.32
**Infections**	Viral diseases (excl chronic hepatitis/HIV)	viral	BMI	obese	1.26	1.07	1.47	0.08	4.39E-03	147978	1102	6.96	1.32
**Infections**	Viral diseases (excl chronic hepatitis/HIV)	viral	BMI	overweight	1.03	0.89	1.19	0.07	6.58E-01	147978	1102	6.96	1.32
**Musculoskeletal**	Giant Cell arteritis	GCA	BMI	obese	1.23	0.85	1.77	0.19	2.67E-01	153956	194	6.98	1.31
**Musculoskeletal**	Giant Cell arteritis	GCA	BMI	underweight	3.06	0.96	9.77	0.59	5.87E-02	153956	194	6.98	1.31
**Musculoskeletal**	Giant Cell arteritis	GCA	BMI	overweight	0.92	0.65	1.3	0.18	6.46E-01	153956	194	6.98	1.31

**Musculoskeletal**	Osteoarthritis (excl spine)	OA	BMI	underweight	0.84	0.61	1.15	0.16	2.77E-01	130200	11491	6.8	1.51
**Musculoskeletal**	Polymyalgia Rheumatica	PMR	BMI	obese	1.28	1.08	1.52	0.09	5.02E-03	153501	895	6.96	1.32
**Musculoskeletal**	Polymyalgia Rheumatica	PMR	BMI	overweight	1.04	0.89	1.23	0.08	5.90E-01	153501	895	6.96	1.32
**Musculoskeletal**	Polymyalgia Rheumatica	PMR	BMI	underweight	0	0	Inf	509.98	9.78E-01	153501	895	6.96	1.32
**Musculoskeletal**	Psoriatic arthropathy	PSA	BMI	obese	1.63	1.11	2.4	0.2	1.26E-02	153520	195	6.96	1.31
**Musculoskeletal**	Psoriatic arthropathy	PSA	BMI	overweight	1.39	0.98	1.99	0.18	6.80E-02	153520	195	6.96	1.31
**Musculoskeletal**	Psoriatic arthropathy	PSA	BMI	underweight	0	0	Inf	1112.47	9.90E-01	153520	195	6.96	1.31
**Musculoskeletal**	Rheumatoid Arthritis	RhA	BMI	underweight	0.87	0.28	2.72	0.58	8.11E-01	151660	896	6.96	1.32
**Musculoskeletal**	Carpal tunnel syndrome	carpal_tunnel	BMI	underweight	0.57	0.27	1.2	0.38	1.41E-01	147905	2854	6.94	1.34
**Musculoskeletal**	Collapsed vertebra	collapsed_vert	BMI	underweight	2.46	0.78	7.79	0.59	1.26E-01	153793	299	6.98	1.31
**Musculoskeletal**	Collapsed vertebra	collapsed_vert	BMI	overweight	1.22	0.93	1.62	0.14	1.53E-01	153793	299	6.98	1.31
**Musculoskeletal**	Collapsed vertebra	collapsed_vert	BMI	obese	1.16	0.84	1.59	0.16	3.68E-01	153793	299	6.98	1.31
**Musculoskeletal**	Enthesopathies & synovial disorders	enthesopathy	BMI	underweight	0.71	0.54	0.95	0.15	2.08E-02	126805	13951	6.8	1.58
**Musculoskeletal**	Fibromatoses	fibromatosis	BMI	underweight	0.74	0.33	1.65	0.41	4.63E-01	152148	1726	6.95	1.33
**Musculoskeletal**	Fibromatoses	fibromatosis	BMI	overweight	0.83	0.75	0.93	0.05	7.36E-04	152148	1726	6.95	1.33

**Musculoskeletal**	Fracture of hip	fracture_hip	BMI	underweight	1.91	0.9	4.05	0.38	9.26E-02	153621	538	6.96	1.31
**Musculoskeletal**	Fracture of wrist	fracture_wrist	BMI	overweight	0.92	0.83	1.03	0.06	1.34E-01	150487	1656	6.95	1.33
**Musculoskeletal**	Fracture of wrist	fracture_wrist	BMI	underweight	0.87	0.47	1.62	0.32	6.61E-01	150487	1656	6.95	1.33
**Musculoskeletal**	Gout	gout	BMI	underweight	0.95	0.36	2.55	0.5	9.23E-01	149466	2656	6.94	1.34
**Musculoskeletal**	Intervertebral disc disorders	intervert_disc	BMI	underweight	0.89	0.48	1.67	0.32	7.23E-01	146841	2710	6.94	1.34
**Musculoskeletal**	Scoliosis	scoliosis	BMI	obese	0.82	0.61	1.1	0.15	1.84E-01	153292	351	6.97	1.31
**Musculoskeletal**	Scoliosis	scoliosis	BMI	underweight	1.44	0.46	4.54	0.58	5.30E-01	153292	351	6.97	1.31
**Musculoskeletal**	Scoliosis	scoliosis	BMI	overweight	0.94	0.74	1.2	0.12	6.12E-01	153292	351	6.97	1.31
**Musculoskeletal**	Sjogren's disease	sjogren	BMI	overweight	0.57	0.36	0.9	0.23	1.61E-02	153824	116	6.98	1.31
**Musculoskeletal**	Sjogren's disease	sjogren	BMI	obese	1.07	0.69	1.66	0.22	7.50E-01	153824	116	6.98	1.31
**Musculoskeletal**	Sjogren's disease	sjogren	BMI	underweight	0	0	Inf	1518.13	9.92E-01	153824	116	6.98	1.31
**Musculoskeletal**	Spinal stenosis	spinal_stenosis	BMI	underweight	1.2	0.5	2.91	0.45	6.82E-01	153352	1299	6.95	1.33
**Musculoskeletal**	Spondylolisthesis	spondylolisthesis	BMI	overweight	1.04	0.82	1.32	0.12	7.19E-01	153400	447	6.96	1.31
**Musculoskeletal**	Spondylolisthesis	spondylolisthesis	BMI	underweight	1	0.25	4.05	0.71	9.99E-01	153400	447	6.96	1.31
**Musculoskeletal**	Spondylosis	spondylosis	BMI	underweight	0.51	0.26	1.03	0.36	6.03E-02	145448	3556	6.92	1.4

**Musculoskeletal**	Spondylosis	spondylosis	BMI	overweight	1.15	1.06	1.25	0.04	8.57E-04	145448	3556	6.92	1.4
**Neurological**	Multiple sclerosis	MS	BMI	obese	1.08	0.69	1.7	0.23	7.35E-01	153420	132	6.98	1.31
**Neurological**	Multiple sclerosis	MS	BMI	overweight	0.98	0.66	1.47	0.21	9.28E-01	153420	132	6.98	1.31
**Neurological**	Multiple sclerosis	MS	BMI	underweight	0	0	Inf	1338.6	9.92E-01	153420	132	6.98	1.31
**Neurological**	Parkinson's disease	Parkinsons	BMI	overweight	1.31	1.05	1.63	0.11	1.82E-02	153787	481	6.96	1.31
**Neurological**	Parkinson's disease	Parkinsons	BMI	obese	1.15	0.89	1.49	0.13	2.93E-01	153787	481	6.96	1.31
**Neurological**	Parkinson's disease	Parkinsons	BMI	underweight	0.62	0.09	4.42	1	6.31E-01	153787	481	6.96	1.31
**Neurological**	Disorders of autonomic nervous system	autonomic_neuro	BMI	underweight	3.97	1.24	12.75	0.6	2.06E-02	153810	191	6.97	1.31
**Neurological**	Disorders of autonomic nervous system	autonomic_neuro	BMI	obese	1.7	1.16	2.48	0.19	6.04E-03	153810	191	6.97	1.31
**Neurological**	Disorders of autonomic nervous system	autonomic_neuro	BMI	overweight	1.1	0.76	1.59	0.19	6.20E-01	153810	191	6.97	1.31
**Neurological**	Bell's palsy	bells	BMI	overweight	1.52	1.13	2.05	0.15	6.29E-03	153187	334	6.96	1.31
**Neurological**	Bell's palsy	bells	BMI	underweight	1.02	0.14	7.36	1.01	9.84E-01	153187	334	6.96	1.31
**Neurological**	Postviral fatigue syndrome, neurasthenia and fibromyalgia	chronic_fatigue	BMI	underweight	0.23	0.03	1.67	1	1.48E-01	151083	840	6.95	1.32

**Neurological**	Postviral fatigue syndrome, neurasthenia and fibromyalgia	chronic_fatigue	BMI	overweight	1.34	1.13	1.59	0.09	6.96E-04	151083	840	6.95	1.32
**Neurological**	Diabetic neurological complications	dm_neuro	BMI	underweight	6.61	1.55	28.13	0.74	1.06E-02	153829	221	6.98	1.31
**Neurological**	Diabetic neurological complications	dm_neuro	BMI	overweight	1.8	1.1	2.93	0.25	1.89E-02	153829	221	6.98	1.31
**Neurological**	Epilepsy	epilepsy	BMI	underweight	2.22	0.7	7.01	0.59	1.75E-01	151976	369	6.96	1.31
**Neurological**	Epilepsy	epilepsy	BMI	overweight	1.35	1.05	1.75	0.13	2.02E-02	151976	369	6.96	1.31
**Neurological**	Epilepsy	epilepsy	BMI	obese	1.33	0.99	1.77	0.15	5.62E-02	151976	369	6.96	1.31
**Neurological**	Essential tremor	essential_tremor	BMI	obese	1.17	0.89	1.54	0.14	2.48E-01	153805	376	6.96	1.31
**Neurological**	Essential tremor	essential_tremor	BMI	underweight	0.64	0.09	4.62	1	6.62E-01	153805	376	6.96	1.31
**Neurological**	Essential tremor	essential_tremor	BMI	overweight	0.99	0.78	1.27	0.13	9.68E-01	153805	376	6.96	1.31
**Neurological**	Migraine	migraine	BMI	obese	0.98	0.87	1.11	0.06	7.88E-01	140282	1926	6.94	1.34
**Neurological**	Migraine	migraine	BMI	underweight	1.05	0.61	1.82	0.28	8.61E-01	140282	1926	6.94	1.34
**Neurological**	Migraine	migraine	BMI	overweight	0.99	0.89	1.1	0.05	8.72E-01	140282	1926	6.94	1.34
**Neurological**	Peripheral neuropathies (excl. cranial nerve, carpal tunnel syndromes)	periph_neuro	BMI	underweight	1.08	0.56	2.08	0.34	8.28E-01	151144	2225	6.94	1.33
**Neurological**	Trigeminal neuralgia	trigem_neur	BMI	obese	1.35	1.07	1.71	0.12	1.27E-02	153335	474	6.96	1.31

**Neurological**	Trigeminal neuralgia	trigem_neur	BMI	overweight	1.11	0.89	1.38	0.11	3.61E-01	153335	474	6.96	1.31
**Neurological**	Trigeminal neuralgia	trigem_neur	BMI	underweight	0.84	0.21	3.38	0.71	8.04E-01	153335	474	6.96	1.31
**Perinatal**	Congenital malformations of cardiac septa	congenital_septal	BMI	underweight	3.08	0.74	12.71	0.72	1.21E-01	153800	110	6.98	1.31
**Perinatal**	Congenital malformations of cardiac septa	congenital_septal	BMI	obese	0.75	0.46	1.23	0.25	2.51E-01	153800	110	6.98	1.31
**Perinatal**	Congenital malformations of cardiac septa	congenital_septal	BMI	overweight	0.66	0.43	1.03	0.22	6.60E-02	153800	110	6.98	1.31
**Psychiatric**	Bipolar affective disorder and mania	BAD	BMI	overweight	1.3	0.88	1.92	0.2	1.85E-01	152960	163	6.98	1.31
**Psychiatric**	Bipolar affective disorder and mania	BAD	BMI	obese	1.75	1.16	2.63	0.21	7.38E-03	152960	163	6.98	1.31
**Psychiatric**	Bipolar affective disorder and mania	BAD	BMI	underweight	0	0	Inf	1212.97	9.91E-01	152960	163	6.98	1.31
**Psychiatric**	Alcohol Problems	alc_problems	BMI	underweight	1.4	0.98	2.01	0.18	6.61E-02	151939	5819	6.89	1.42
**Psychiatric**	Alzheimer's disease	alzheimer	BMI	obese	0.73	0.55	0.98	0.15	3.32E-02	154053	390	6.96	1.31
**Psychiatric**	Alzheimer's disease	alzheimer	BMI	underweight	0.57	0.08	4.1	1	5.79E-01	154053	390	6.96	1.31
**Psychiatric**	Alzheimer's disease	alzheimer	BMI	overweight	1.01	0.8	1.27	0.12	9.53E-01	154053	390	6.96	1.31
**Psychiatric**	Anxiety disorders	anxiety	BMI	overweight	1.07	0.99	1.16	0.04	1.04E-01	127169	3421	6.91	1.41
**Psychiatric**	Anxiety disorders	anxiety	BMI	underweight	1.93	1.36	2.74	0.18	2.52E-04	127169	3421	6.91	1.41
**Psychiatric**	Delirium, not induced by alcohol and other psychoactive substances	delirium	BMI	obese	1.7	1.23	2.35	0.17	1.45E-03	154029	260	6.98	1.31

**Psychiatric**	Delirium, not induced by alcohol and other psychoactive substances	delirium	BMI	underweight	2.39	0.58	9.78	0.72	2.26E-01	154029	260	6.98	1.31
**Psychiatric**	Delirium, not induced by alcohol and other psychoactive substances	delirium	BMI	overweight	0.99	0.72	1.36	0.16	9.44E-01	154029	260	6.98	1.31
**Psychiatric**	Dementia	dementia	BMI	obese	0.75	0.53	1.07	0.18	1.16E-01	153204	228	6.98	1.3
**Psychiatric**	Dementia	dementia	BMI	overweight	0.72	0.53	0.98	0.16	3.50E-02	153204	228	6.98	1.3
**Psychiatric**	Dementia	dementia	BMI	underweight	1.81	0.44	7.36	0.72	4.09E-01	153204	228	6.98	1.3
**Psychiatric**	Dementia (excluding Alzheimer's)	dementia_ex_Alz	BMI	underweight	2.99	0.73	12.32	0.72	1.29E-01	153217	146	6.98	1.31
**Psychiatric**	Dementia (excluding Alzheimer's)	dementia_ex_Alz	BMI	overweight	0.63	0.42	0.93	0.2	2.00E-02	153217	146	6.98	1.31
**Psychiatric**	Dementia (excluding Alzheimer's)	dementia_ex_Alz	BMI	obese	0.92	0.6	1.4	0.21	6.90E-01	153217	146	6.98	1.31
**Psychiatric**	Depression	depression	BMI	underweight	1.74	1.14	2.66	0.22	1.03E-02	106004	3004	7	1.34
**Psychiatric**	Schizophrenia, schizotypal and delusional disorders	schizo	BMI	overweight	1.85	1.14	2.99	0.25	1.28E-02	153513	116	6.98	1.31
**Psychiatric**	Schizophrenia, schizotypal and delusional disorders	schizo	BMI	obese	1.9	1.12	3.22	0.27	1.74E-02	153513	116	6.98	1.31
**Psychiatric**	Schizophrenia, schizotypal and delusional disorders	schizo	BMI	underweight	0	0	Inf	1472.21	9.93E-01	153513	116	6.98	1.31
**Psychiatric**	Other psychoactive substance misuse	substance_misuse	BMI	obese	1.29	0.94	1.78	0.16	1.14E-01	153575	266	6.98	1.31

**Psychiatric**	Other psychoactive substance misuse	substance_misuse	BMI	underweight	1.74	0.43	7.11	0.72	4.38E-01	153575	266	6.98	1.31
**Psychiatric**	Other psychoactive substance misuse	substance_misuse	BMI	overweight	0.99	0.73	1.33	0.15	9.25E-01	153575	266	6.98	1.31
**Respiratory**	COPD	COPD	BMI	overweight	0.87	0.8	0.95	0.04	1.79E-03	151345	3256	6.94	1.34
**Respiratory**	COPD_excl_bronchitis_NOS	COPD_excl_bronchitis_NOS	BMI	overweight	0.86	0.79	0.94	0.04	5.67E-04	151407	3171	6.94	1.34
**Respiratory**	Allergic and chronic rhinitis	allergic_rhinitis	BMI	overweight	1.05	0.96	1.14	0.04	2.96E-01	110387	2952	6.93	1.41
**Respiratory**	Allergic and chronic rhinitis	allergic_rhinitis	BMI	obese	1.04	0.95	1.15	0.05	3.90E-01	110387	2952	6.93	1.41
**Respiratory**	Allergic and chronic rhinitis	allergic_rhinitis	BMI	underweight	0.97	0.57	1.65	0.27	9.12E-01	110387	2952	6.93	1.41
**Respiratory**	Aspiration pneumonitis	aspiration_pneumo	BMI	overweight	0.8	0.57	1.1	0.17	1.70E-01	154032	229	6.98	1.31
**Respiratory**	Aspiration pneumonitis	aspiration_pneumo	BMI	underweight	3.49	1.09	11.1	0.59	3.46E-02	154032	229	6.98	1.31
**Respiratory**	Aspiration pneumonitis	aspiration_pneumo	BMI	obese	1.17	0.83	1.65	0.17	3.62E-01	154032	229	6.98	1.31
**Respiratory**	Asthma	asthma	BMI	underweight	1.07	0.55	2.06	0.34	8.51E-01	133123	2221	6.94	1.33
**Respiratory**	Bronchiectasis	bronchiectasis	BMI	overweight	1.01	0.85	1.2	0.09	9.37E-01	153099	734	6.96	1.32
**Respiratory**	Bronchiectasis	bronchiectasis	BMI	obese	1	0.82	1.22	0.1	9.76E-01	153099	734	6.96	1.32
**Respiratory**	Hypertrophy of nasal turbinates	hyper_nasal_turbs	BMI	obese	1.69	1.06	2.7	0.24	2.80E-02	153544	158	6.98	1.31
**Respiratory**	Hypertrophy of nasal turbinates	hyper_nasal_turbs	BMI	overweight	1.88	1.24	2.85	0.21	3.09E-03	153544	158	6.98	1.31

**Respiratory**	Hypertrophy of nasal turbinates	hyper_nasal_turbs	BMI	underweight	2.17	0.3	15.94	1.02	4.45E-01	153544	158	6.98	1.31
**Respiratory**	Nasal polyp	nasal_polyp	BMI	obese	1.03	0.84	1.26	0.1	7.81E-01	151531	687	6.96	1.32
**Respiratory**	Nasal polyp	nasal_polyp	BMI	underweight	1.07	0.34	3.33	0.58	9.13E-01	151531	687	6.96	1.32
**Respiratory**	Nasal polyp	nasal_polyp	BMI	overweight	1	0.83	1.19	0.09	9.63E-01	151531	687	6.96	1.32
**Respiratory**	Pleural effusion	pleural_effusion	BMI	underweight	2.02	1.11	3.69	0.31	2.11E-02	153405	1481	6.96	1.32
**Respiratory**	Pleural effusion	pleural_effusion	BMI	overweight	0.98	0.86	1.11	0.07	7.21E-01	153405	1481	6.96	1.32
**Respiratory**	Pleural plaque	pleural_plaque	BMI	underweight	2.89	0.71	11.78	0.72	1.40E-01	153972	322	6.96	1.31
**Respiratory**	Pleural plaque	pleural_plaque	BMI	overweight	1.13	0.85	1.5	0.14	4.03E-01	153972	322	6.96	1.31
**Respiratory**	Pleural plaque	pleural_plaque	BMI	obese	1.31	0.96	1.78	0.16	9.17E-02	153972	322	6.96	1.31
**Respiratory**	Pneumothorax	pneumothorax	BMI	obese	0.7	0.49	1.01	0.18	5.45E-02	153290	230	6.98	1.31
**Respiratory**	Pneumothorax	pneumothorax	BMI	underweight	3.92	1.43	10.73	0.51	7.76E-03	153290	230	6.98	1.31
**Respiratory**	Pneumothorax	pneumothorax	BMI	overweight	0.76	0.56	1.03	0.15	7.89E-02	153290	230	6.98	1.31
**Respiratory**	Pulmonary collapse (excl pneumothorax)	pulm_collapse	BMI	overweight	1.15	0.93	1.42	0.11	1.90E-01	153850	578	6.96	1.31
**Respiratory**	Pulmonary collapse (excl pneumothorax)	pulm_collapse	BMI	underweight	0.49	0.07	3.49	1	4.75E-01	153850	578	6.96	1.31
**Respiratory**	Other interstitial pulmonary diseases with fibrosis	pulm_fibrosis	BMI	overweight	1.29	0.97	1.73	0.15	7.88E-02	153842	330	6.96	1.31

**Respiratory**	Other interstitial pulmonary diseases with fibrosis	pulm_fibrosis	BMI	obese	1.68	1.24	2.28	0.16	8.86E-04	153842	330	6.96	1.31
**Respiratory**	Respiratory failure	resp_failure	BMI	overweight	1.17	0.96	1.42	0.1	1.29E-01	153944	719	6.96	1.31
**Respiratory**	Chronic sinusitis	sinusitis	BMI	underweight	0.73	0.47	1.15	0.23	1.75E-01	139467	4868	6.91	1.43
**Respiratory**	Chronic sinusitis	sinusitis	BMI	overweight	1.11	1.04	1.19	0.03	2.06E-03	139467	4868	6.91	1.43
**Respiratory**	Chronic sinusitis	sinusitis	BMI	obese	1.15	1.06	1.24	0.04	3.99E-04	139467	4868	6.91	1.43
**Respiratory**	Sleep apnoea	sleep_apnoea	BMI	underweight	0.55	0.08	3.95	1	5.55E-01	152546	1400	6.95	1.32
**Skin**	Acne	acne	BMI	overweight	0.83	0.62	1.11	0.15	2.20E-01	150556	248	6.98	1.31
**Skin**	Acne	acne	BMI	underweight	1.73	0.55	5.46	0.59	3.50E-01	150556	248	6.98	1.31
**Skin**	Acne	acne	BMI	obese	0.86	0.61	1.2	0.17	3.64E-01	150556	248	6.98	1.31
**Skin**	Actinic keratosis	actinic_keratosis	BMI	underweight	0.79	0.5	1.26	0.24	3.23E-01	151055	4592	6.92	1.42
**Skin**	Alopecia areata	alopecia_areata	BMI	obese	0.53	0.31	0.88	0.26	1.40E-02	153595	126	6.98	1.31
**Skin**	Alopecia areata	alopecia_areata	BMI	overweight	0.75	0.51	1.1	0.2	1.43E-01	153595	126	6.98	1.31
**Skin**	Alopecia areata	alopecia_areata	BMI	underweight	0	0	Inf	1394.54	9.92E-01	153595	126	6.98	1.31
**Skin**	Dermatitis (atopc/contact/other/unspecified)	dermatitis	BMI	overweight	1.06	1	1.12	0.03	3.34E-02	131299	7152	6.89	1.46
**Skin**	Dermatitis (atopc/contact/other/unspecified)	dermatitis	BMI	underweight	0.94	0.66	1.33	0.18	7.35E-01	131299	7152	6.89	1.46

**Skin**	Keratitis	keratitis	BMI	obese	0.87	0.66	1.15	0.14	3.31E-01	153298	377	6.96	1.31
**Skin**	Keratitis	keratitis	BMI	underweight	1.45	0.46	4.55	0.58	5.27E-01	153298	377	6.96	1.31
**Skin**	Keratitis	keratitis	BMI	overweight	1.01	0.8	1.27	0.12	9.56E-01	153298	377	6.96	1.31
**Skin**	Lichen planus	lichen_planus	BMI	overweight	1.08	0.89	1.3	0.1	4.38E-01	152800	626	6.96	1.32
**Skin**	Lichen planus	lichen_planus	BMI	obese	1.21	0.99	1.49	0.11	6.62E-02	152800	626	6.96	1.32
**Skin**	Lichen planus	lichen_planus	BMI	underweight	0.9	0.29	2.81	0.58	8.53E-01	152800	626	6.96	1.32
**Skin**	Psoriasis	psoriasis	BMI	obese	1.24	1.07	1.43	0.07	4.14E-03	148816	1340	6.95	1.33
**Skin**	Psoriasis	psoriasis	BMI	overweight	1.14	1	1.3	0.07	4.19E-02	148816	1340	6.95	1.33
**Skin**	Psoriasis	psoriasis	BMI	underweight	1.32	0.65	2.65	0.36	4.43E-01	148816	1340	6.95	1.33
**Skin**	Rosacea	rosacea	BMI	obese	1.08	0.96	1.22	0.06	2.02E-01	149906	2095	6.95	1.33
**Skin**	Rosacea	rosacea	BMI	overweight	1.16	1.05	1.29	0.05	4.26E-03	149906	2095	6.95	1.33
**Skin**	Rosacea	rosacea	BMI	underweight	1.56	0.95	2.57	0.25	7.73E-02	149906	2095	6.95	1.33
**Skin**	Seborrheic dermatitis	seb_derm	BMI	obese	1.16	1.05	1.28	0.05	3.67E-03	144426	2845	6.94	1.35
**Skin**	Seborrheic dermatitis	seb_derm	BMI	underweight	1.13	0.68	1.89	0.26	6.34E-01	144426	2845	6.94	1.35
**Skin**	Seborrheic dermatitis	seb_derm	BMI	overweight	1	0.91	1.09	0.05	9.37E-01	144426	2845	6.94	1.35
**Skin**	Urticaria	urticaria	BMI	obese	1.22	1.08	1.37	0.06	1.45E-03	149475	1874	6.95	1.34
**Skin**	Urticaria	urticaria	BMI	overweight	1.1	0.99	1.23	0.06	7.47E-02	149475	1874	6.95	1.34
**Skin**	Urticaria	urticaria	BMI	underweight	0.9	0.47	1.75	0.34	7.63E-01	149475	1874	6.95	1.34
**Skin**	Vitiligo	vitiligo	BMI	obese	1.24	0.79	1.94	0.23	3.44E-01	153604	143	6.98	1.31
**Skin**	Vitiligo	vitiligo	BMI	overweight	1.15	0.77	1.72	0.2	5.00E-01	153604	143	6.98	1.31

**Skin**	Vitiligo	vitiligo	BMI	underweigh t	1.62	0.22	11.82	1.01	6.32E-01	153604	143	6.98	1.31
