# Supplementary figures and images for: A computational framework for defining and validating reproducible phenotyping algorithms of 313 diseases in the UK Biobank
Source: Sci Rep. 2025 Jul 9;15:24607. doi: 10.1038/s41598-025-05838-9 (PMC12241469; doi:10.1038/s41598-025-05838-9)

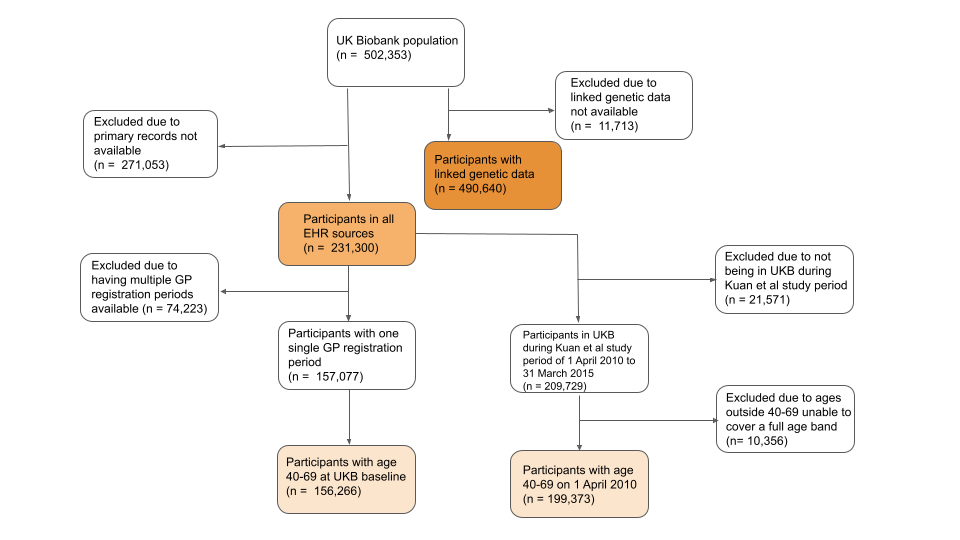

Supplement: Supplementary file 1 — Supplementary Information. [file 41598_2025_5838_MOESM1_ESM.zip › Supplementary/FigureS1.png]

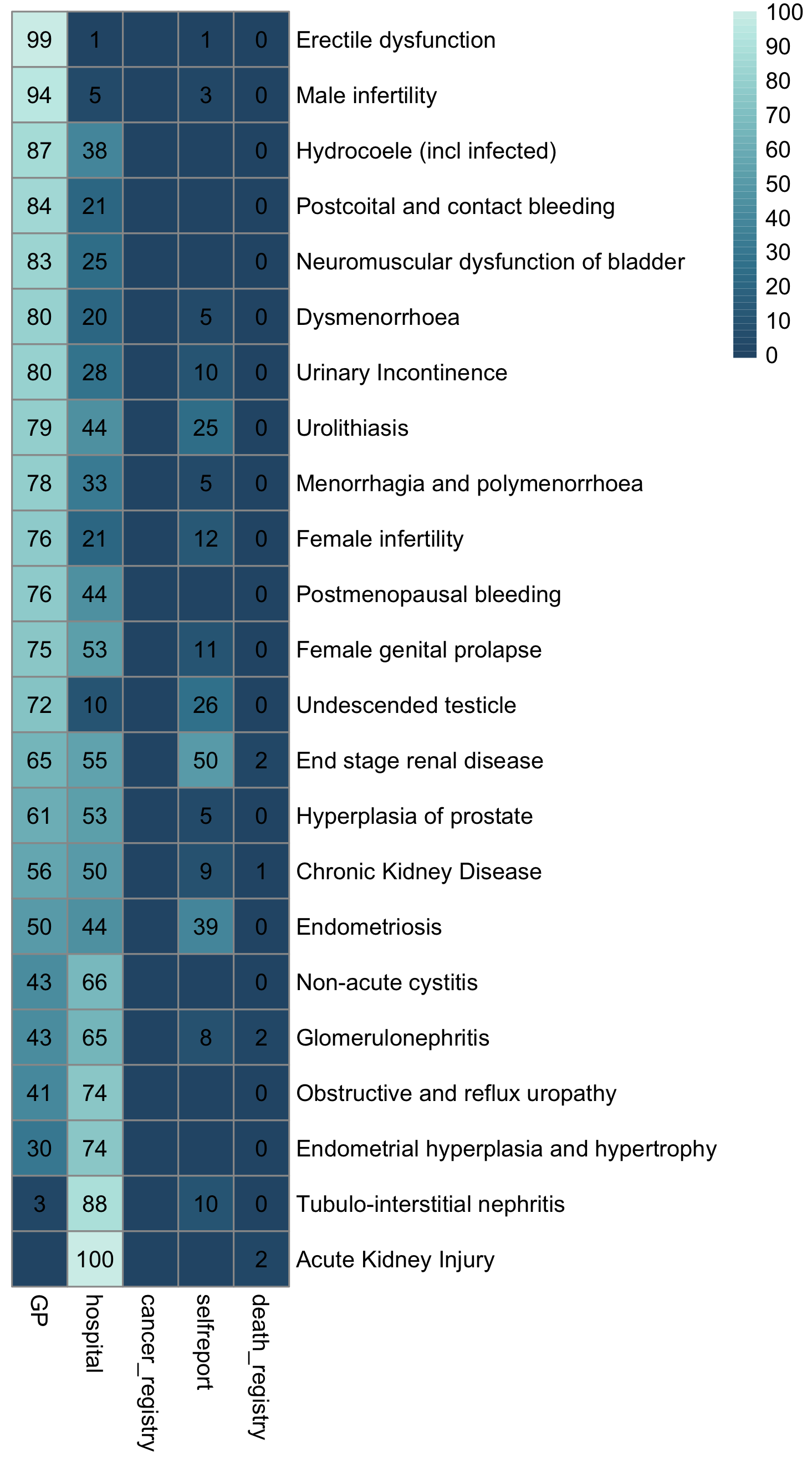

Supplement: Supplementary file 1 — Supplementary Information. [file 41598_2025_5838_MOESM1_ESM.zip › Supplementary/FigureS10_heatmap_source_Genitourinary.png]

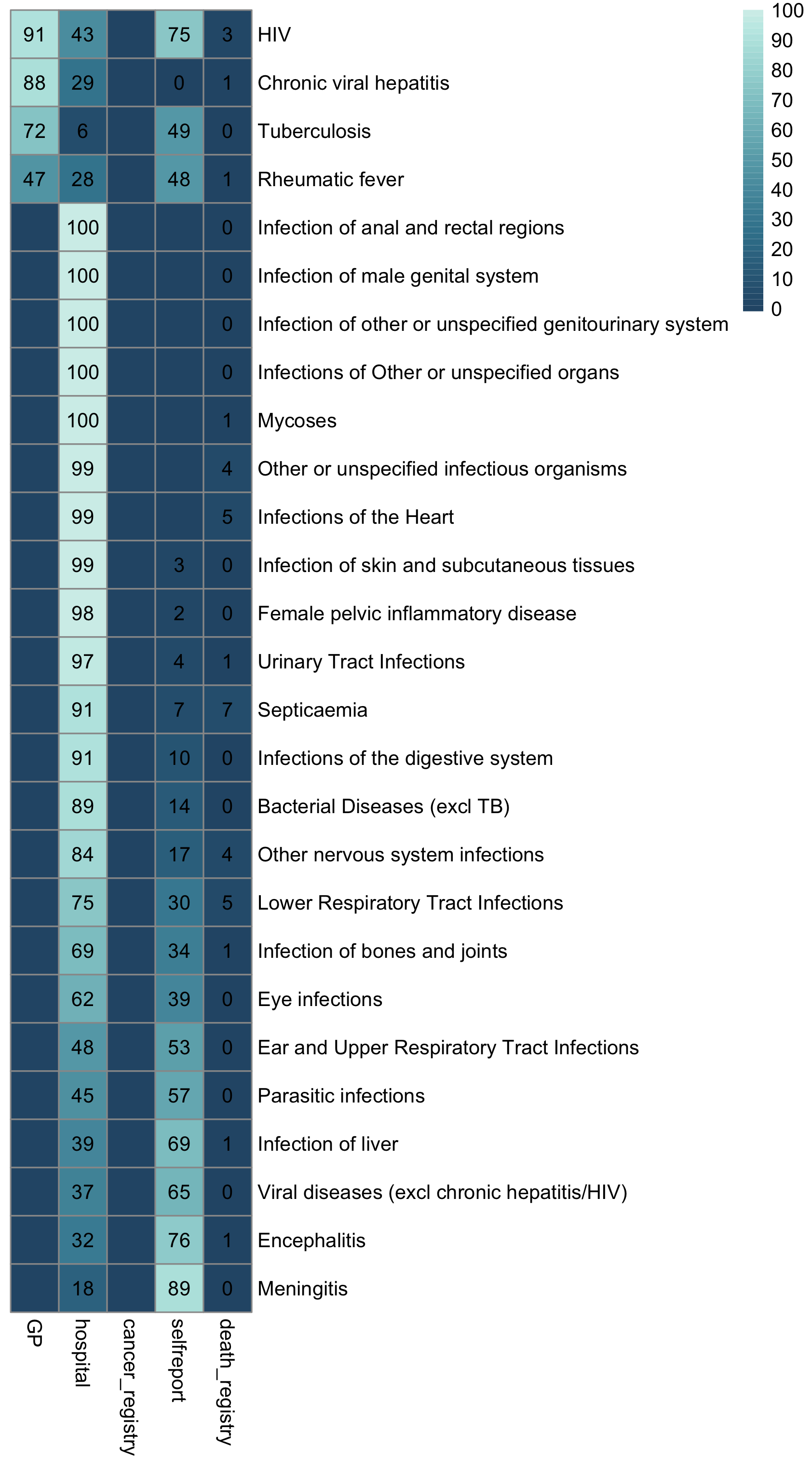

Supplement: Supplementary file 1 — Supplementary Information. [file 41598_2025_5838_MOESM1_ESM.zip › Supplementary/FigureS11_heatmap_source_Infections.png]

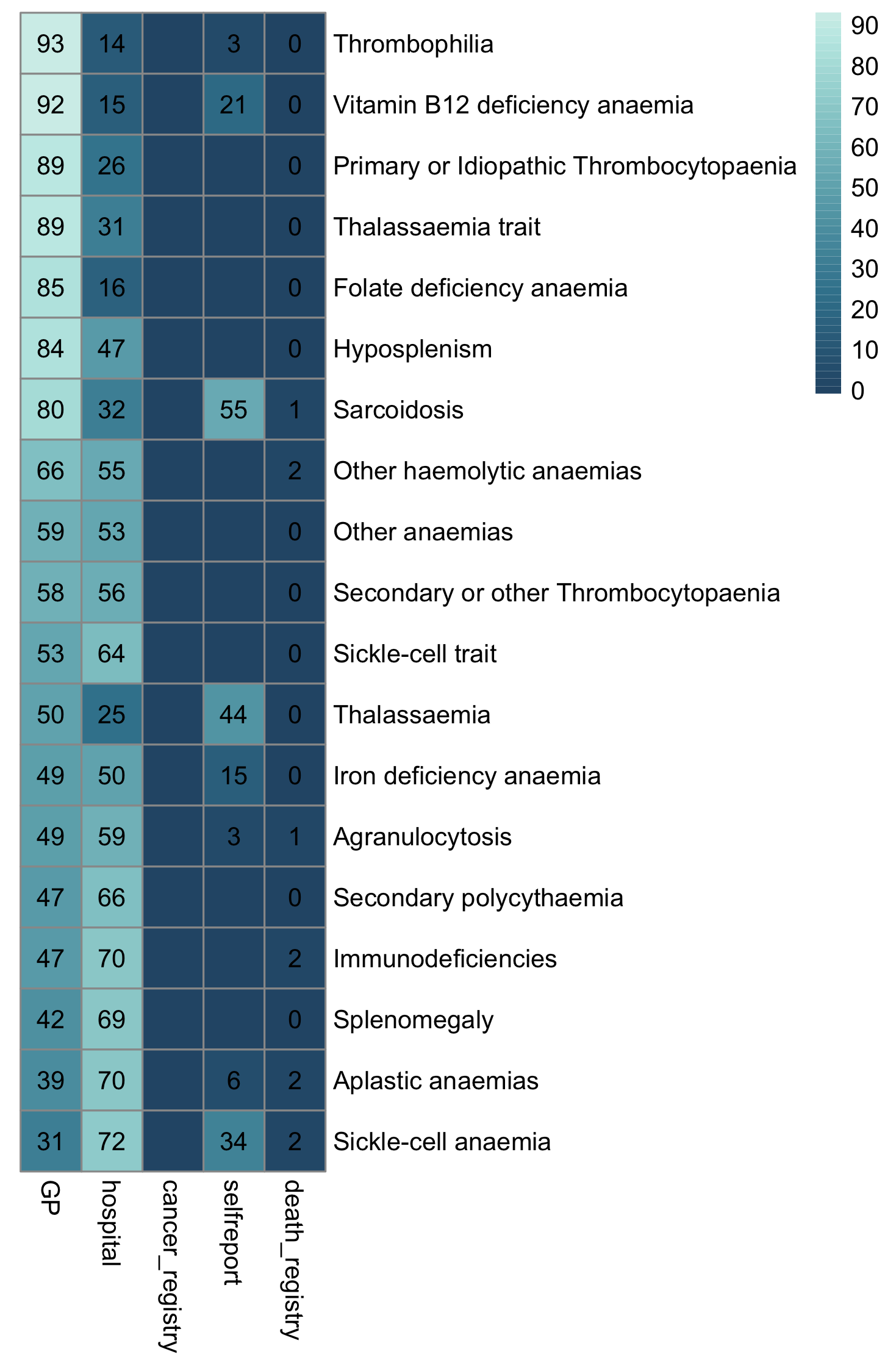

Supplement: Supplementary file 1 — Supplementary Information. [file 41598_2025_5838_MOESM1_ESM.zip › Supplementary/FigureS12_heatmap_source_Haematological or immunological.png]

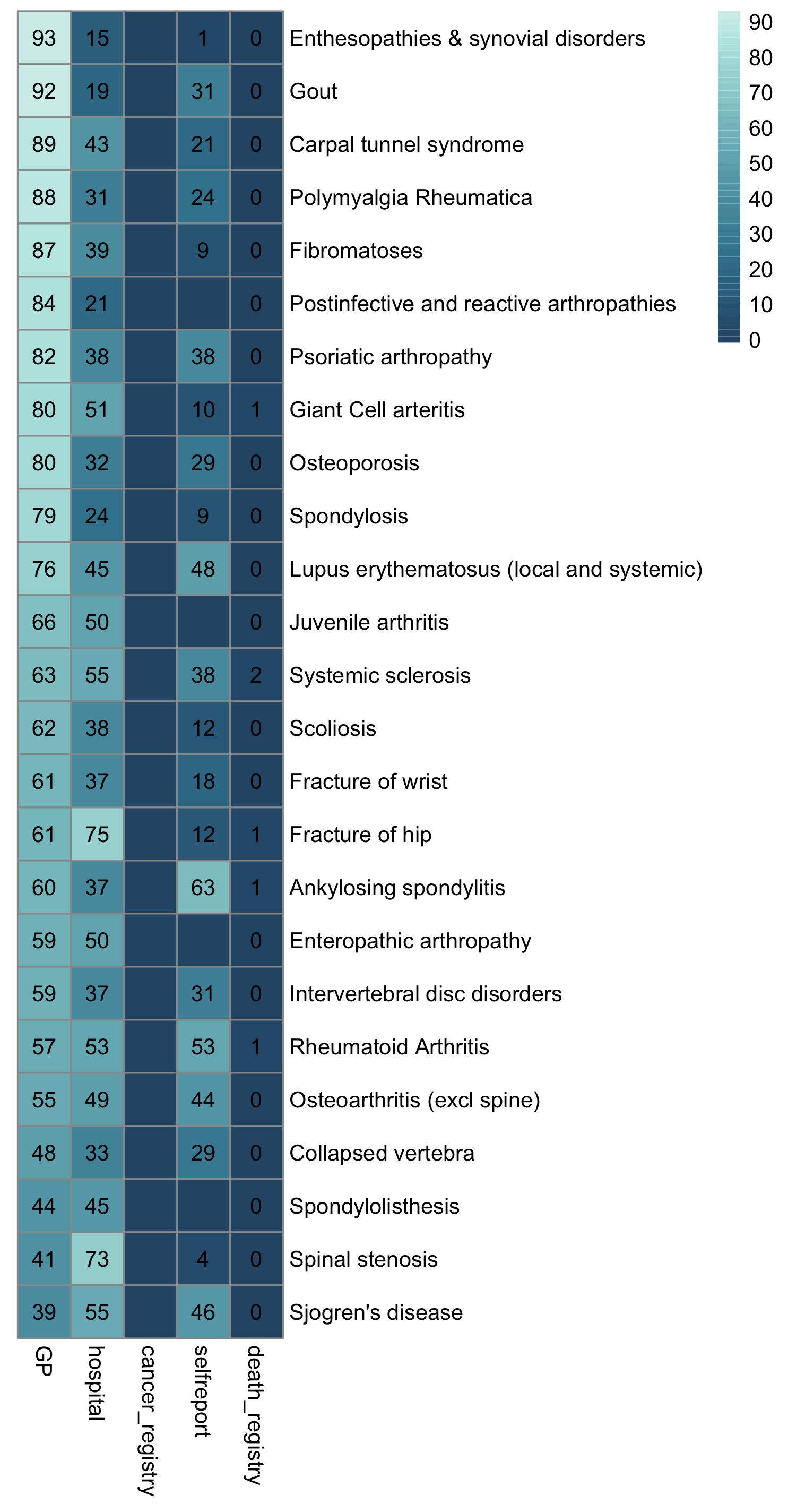

Supplement: Supplementary file 1 — Supplementary Information. [file 41598_2025_5838_MOESM1_ESM.zip › Supplementary/FigureS13_heatmap_source_Musculoskeletal.png]

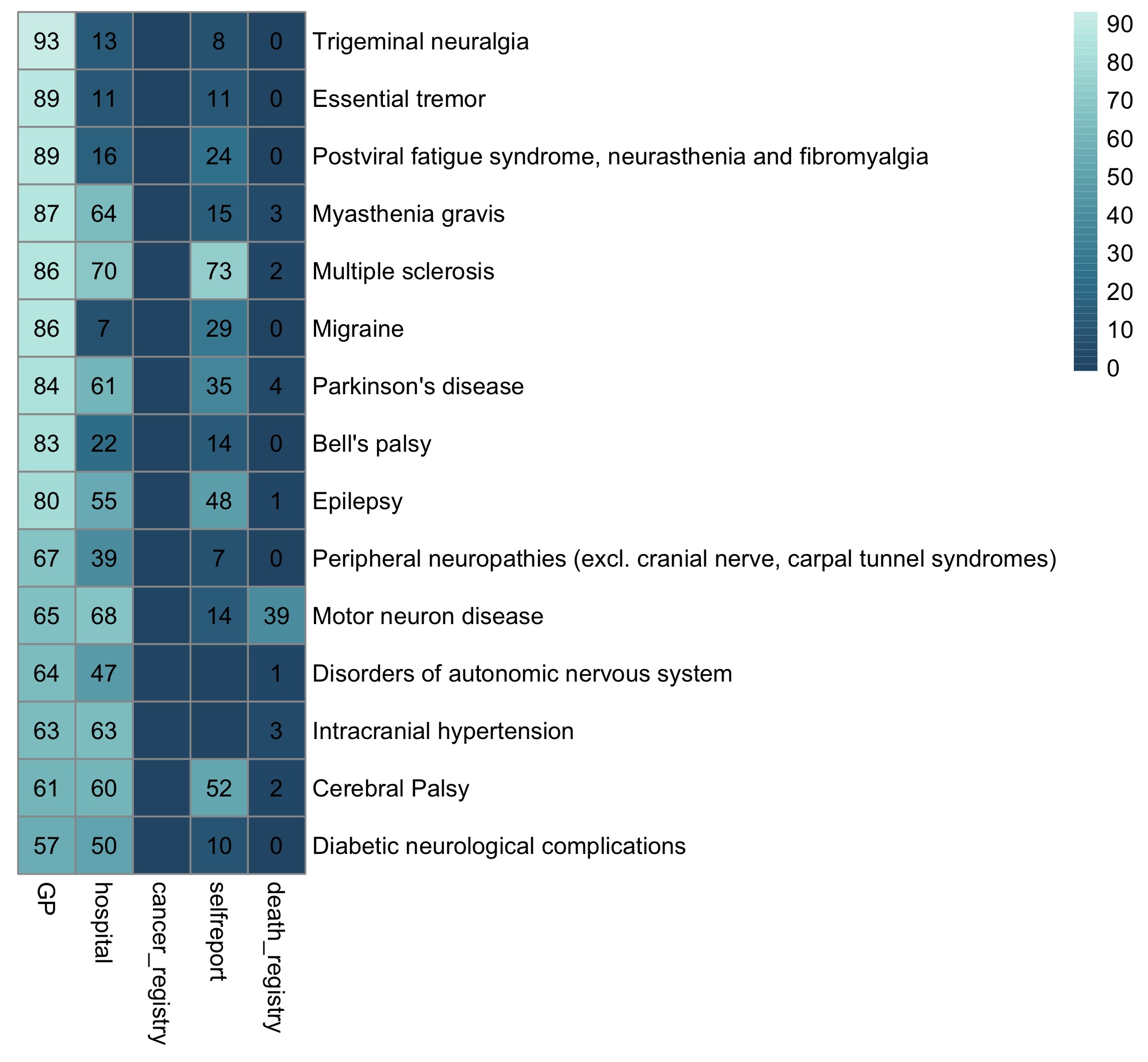

Supplement: Supplementary file 1 — Supplementary Information. [file 41598_2025_5838_MOESM1_ESM.zip › Supplementary/FigureS14_heatmap_source_Neurological.png]

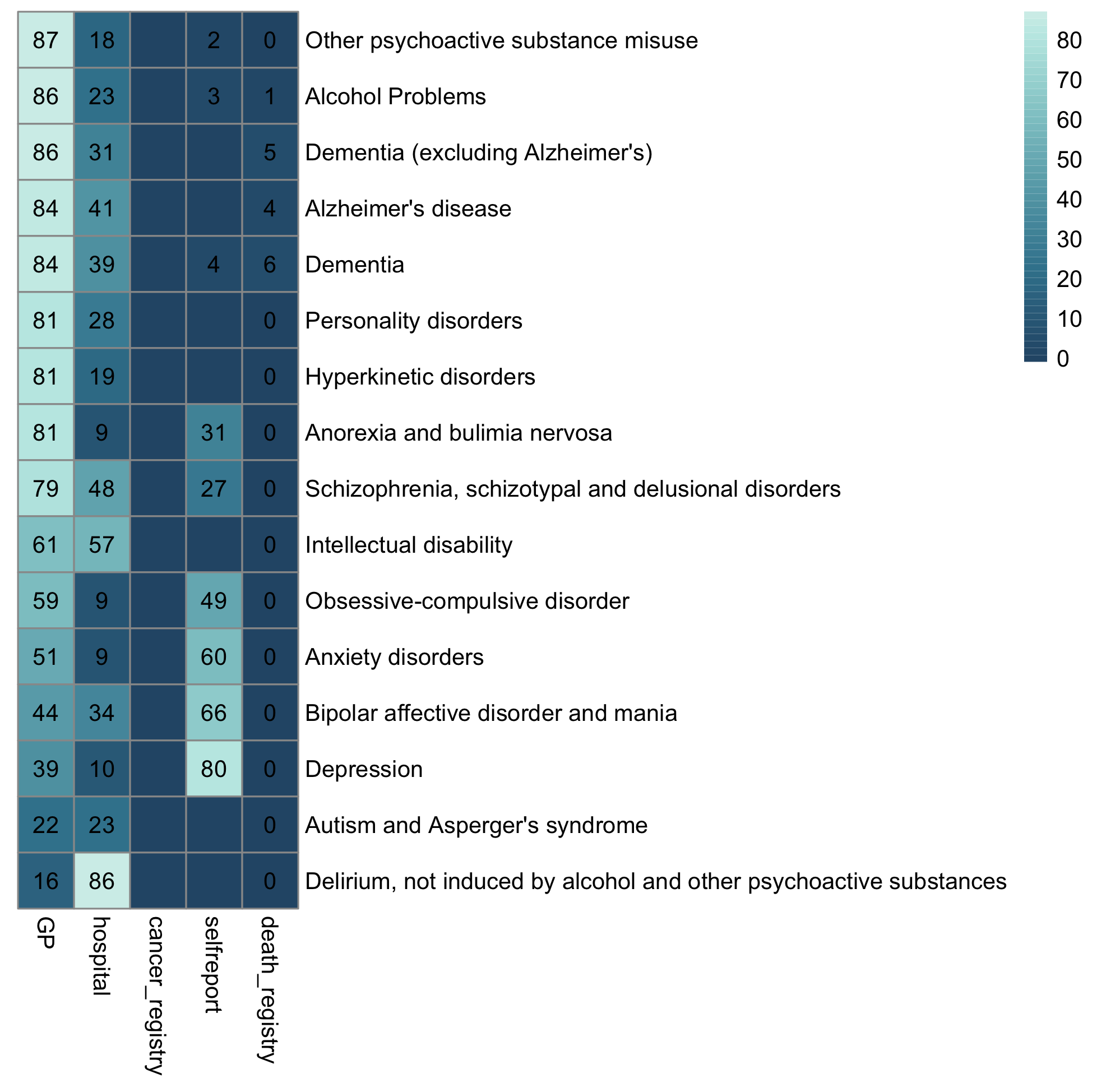

Supplement: Supplementary file 1 — Supplementary Information. [file 41598_2025_5838_MOESM1_ESM.zip › Supplementary/FigureS15_heatmap_source_Psychiatric.png]

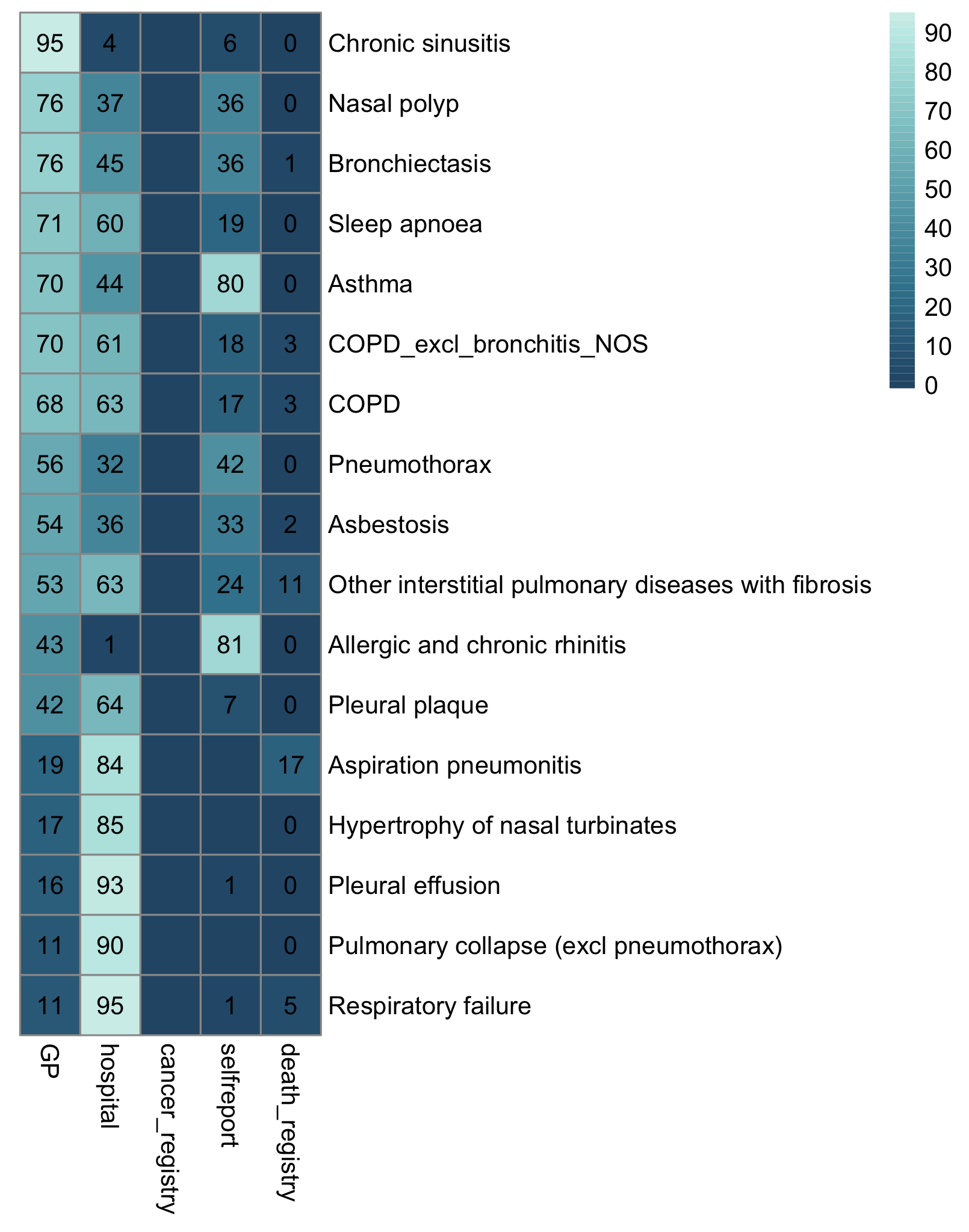

Supplement: Supplementary file 1 — Supplementary Information. [file 41598_2025_5838_MOESM1_ESM.zip › Supplementary/FigureS16_heatmap_source_Respiratory.png]

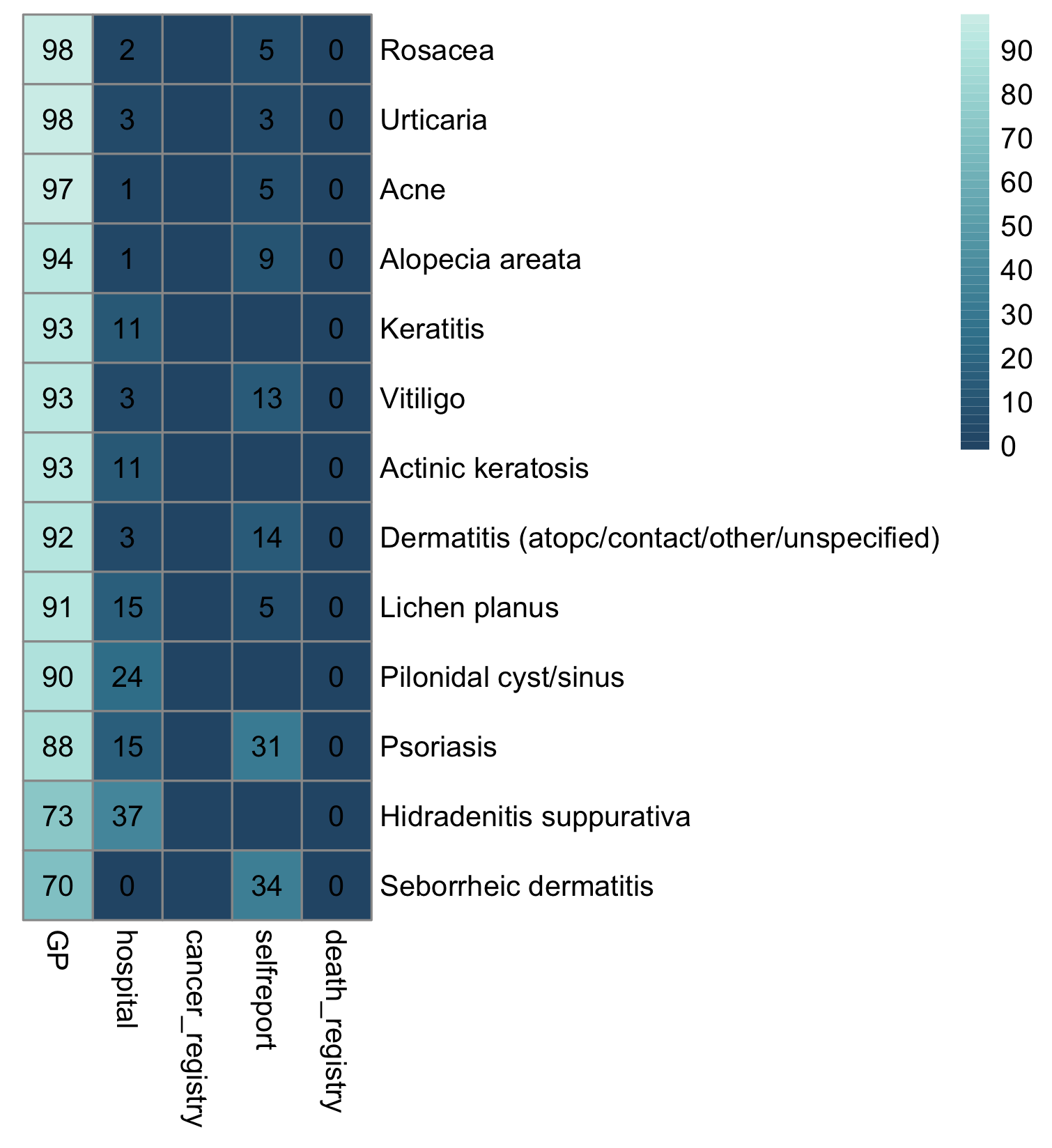

Supplement: Supplementary file 1 — Supplementary Information. [file 41598_2025_5838_MOESM1_ESM.zip › Supplementary/FigureS17_heatmap_source_Skin.png]

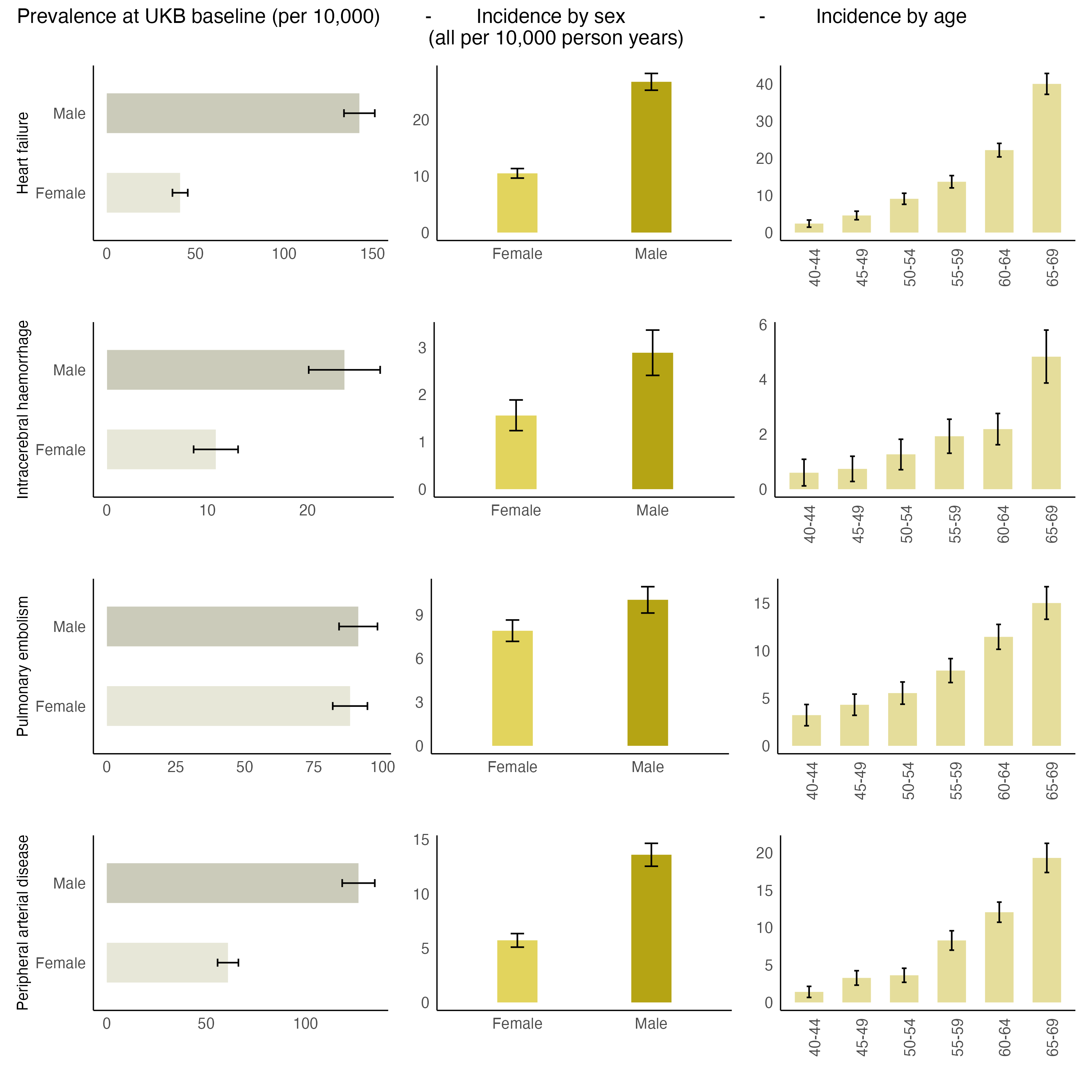

Supplement: Supplementary file 1 — Supplementary Information. [file 41598_2025_5838_MOESM1_ESM.zip › Supplementary/FigureS18_compo_all_plots_cardiov.png]

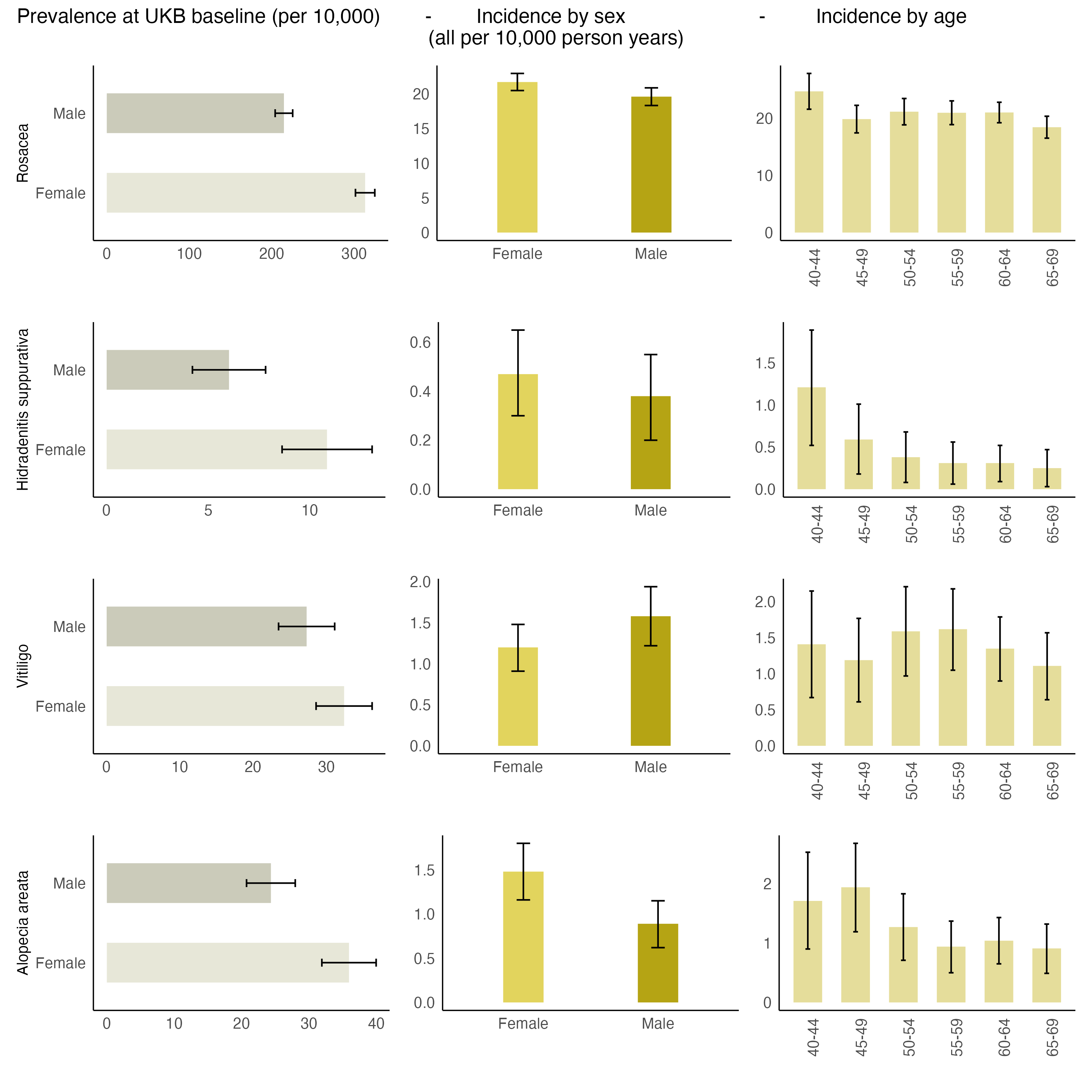

Supplement: Supplementary file 1 — Supplementary Information. [file 41598_2025_5838_MOESM1_ESM.zip › Supplementary/FigureS19_compo_all_plots_skin.png]

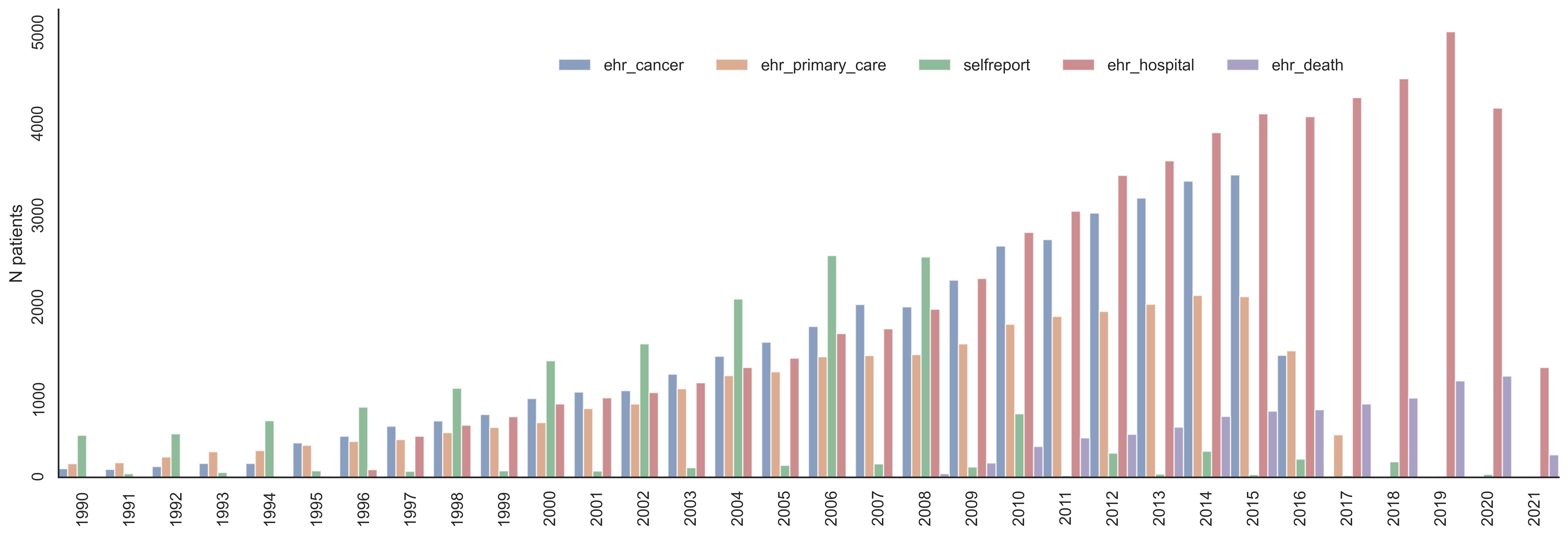

Supplement: Supplementary file 1 — Supplementary Information. [file 41598_2025_5838_MOESM1_ESM.zip › Supplementary/FigureS2.png]

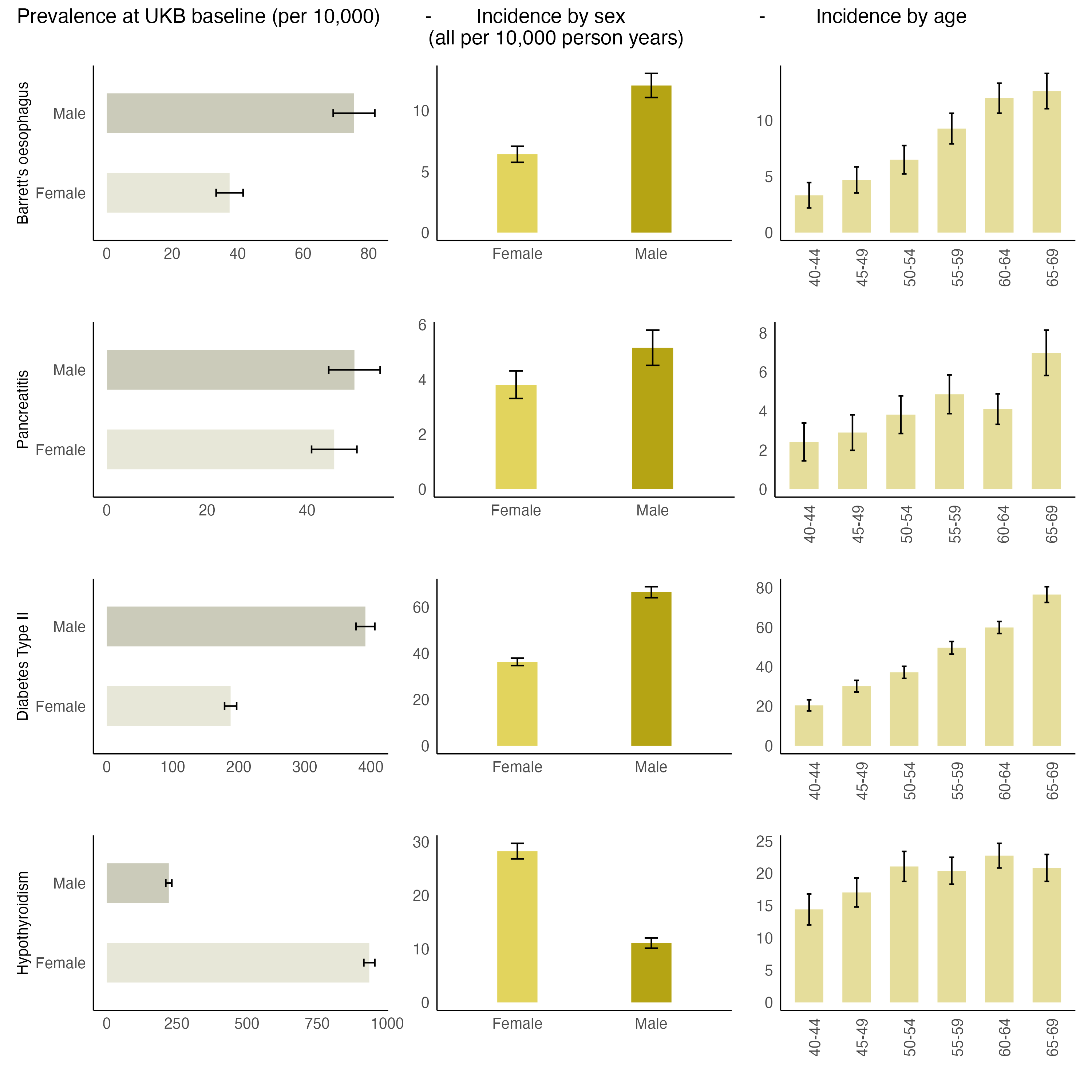

Supplement: Supplementary file 1 — Supplementary Information. [file 41598_2025_5838_MOESM1_ESM.zip › Supplementary/FigureS20_compo_all_plots_endo_dig.png]

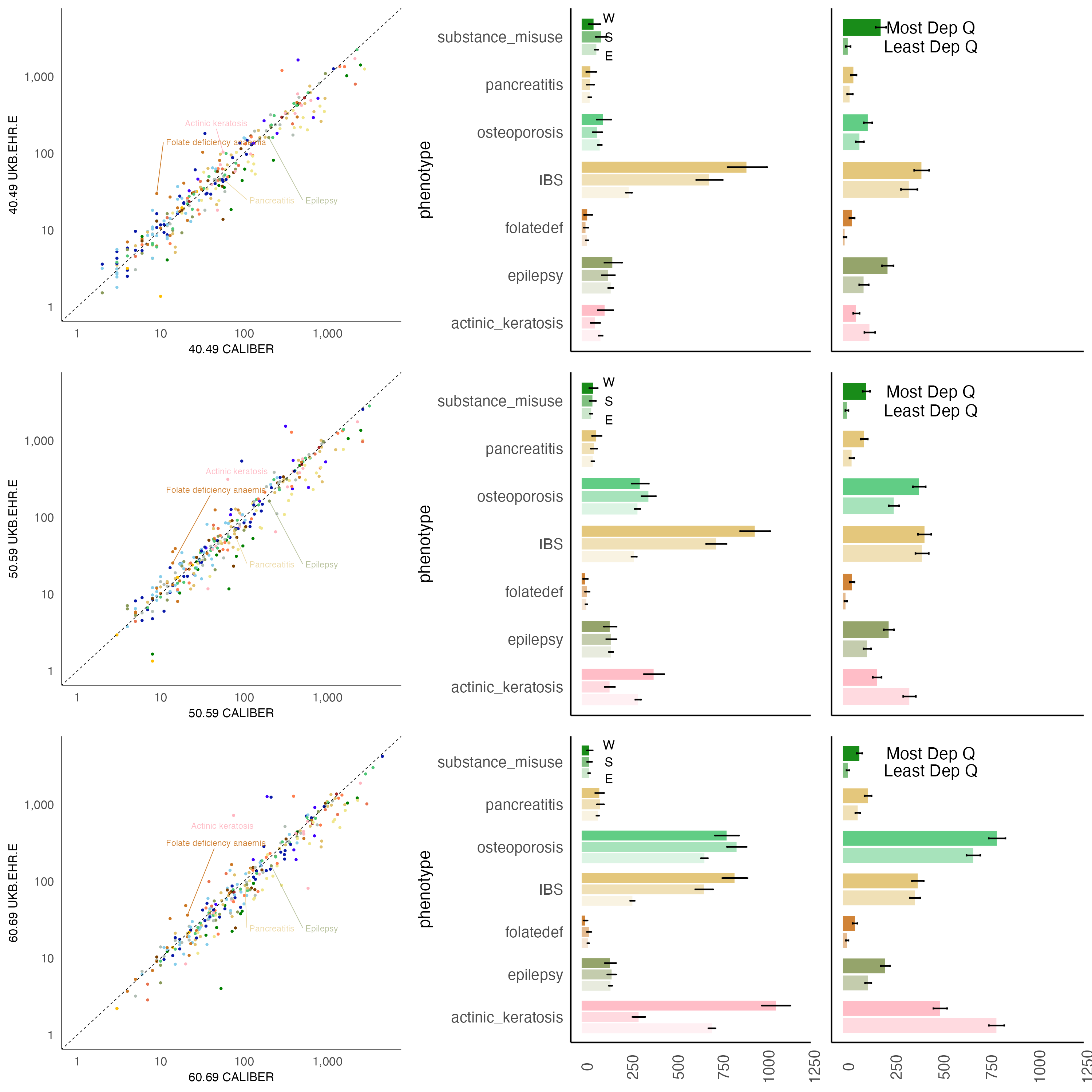

Supplement: Supplementary file 1 — Supplementary Information. [file 41598_2025_5838_MOESM1_ESM.zip › Supplementary/FigureS21.png]

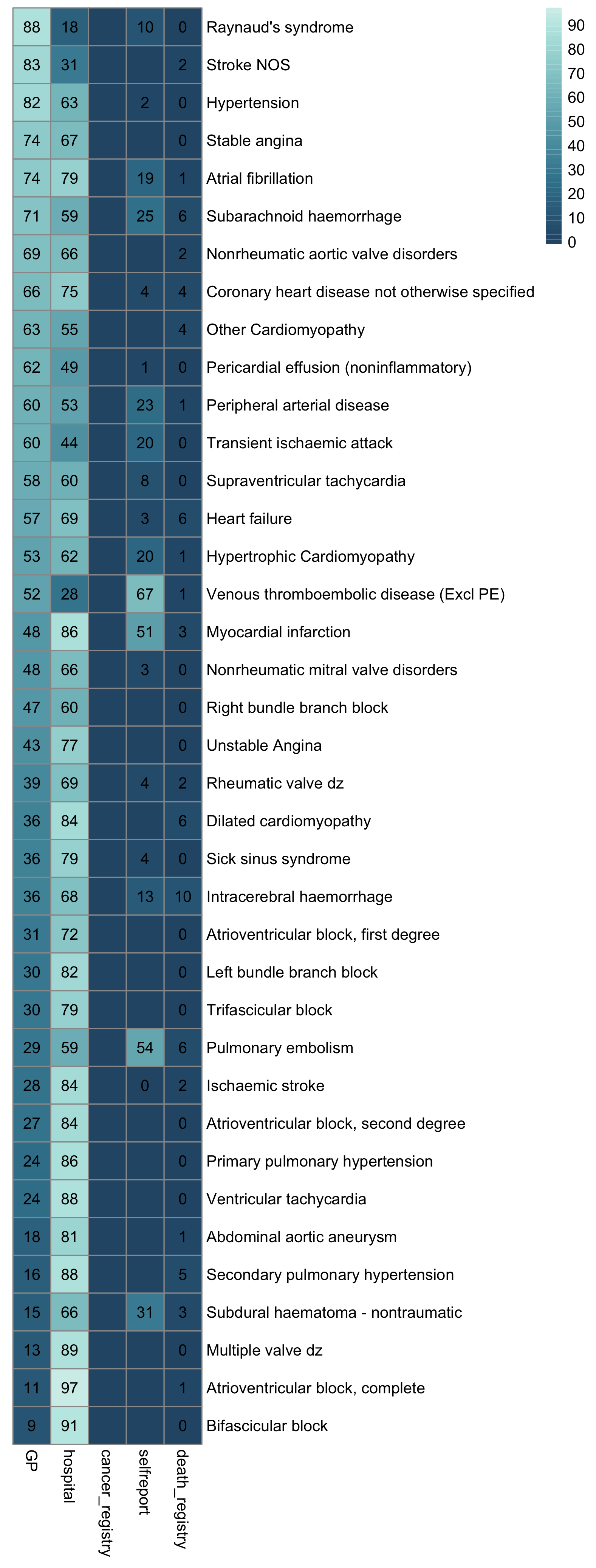

Supplement: Supplementary file 1 — Supplementary Information. [file 41598_2025_5838_MOESM1_ESM.zip › Supplementary/FigureS3_heatmap_source_Cardiovascular.png]

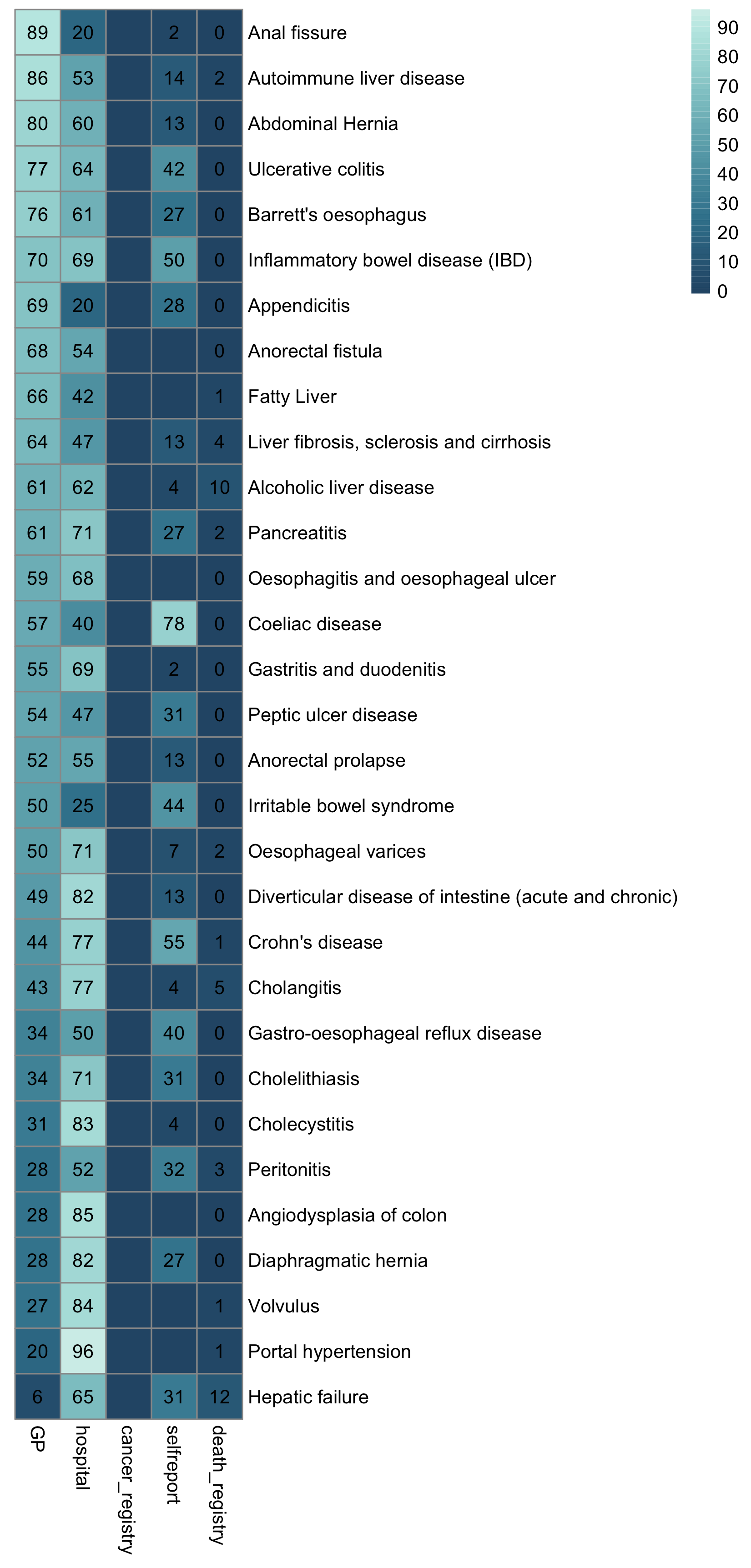

Supplement: Supplementary file 1 — Supplementary Information. [file 41598_2025_5838_MOESM1_ESM.zip › Supplementary/FigureS4_heatmap_source_Digestive.png]

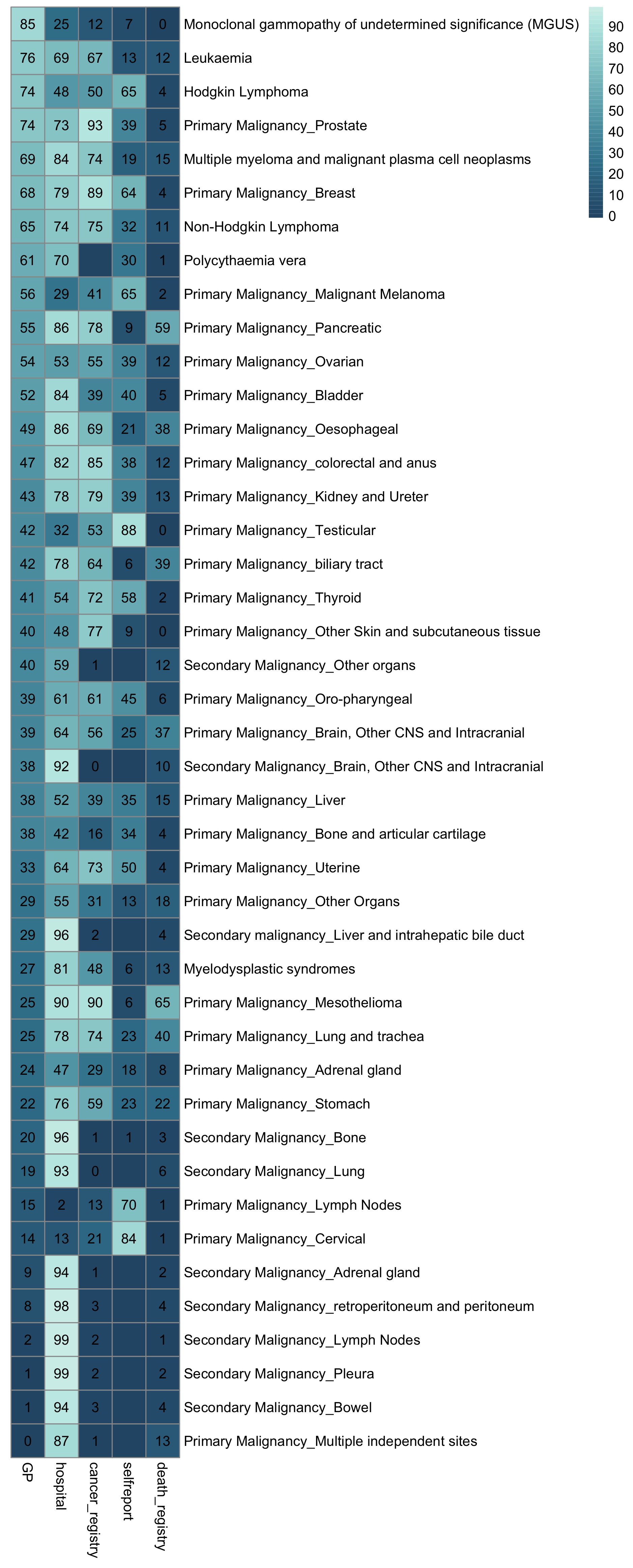

Supplement: Supplementary file 1 — Supplementary Information. [file 41598_2025_5838_MOESM1_ESM.zip › Supplementary/FigureS5_heatmap_source_Cancers.png]

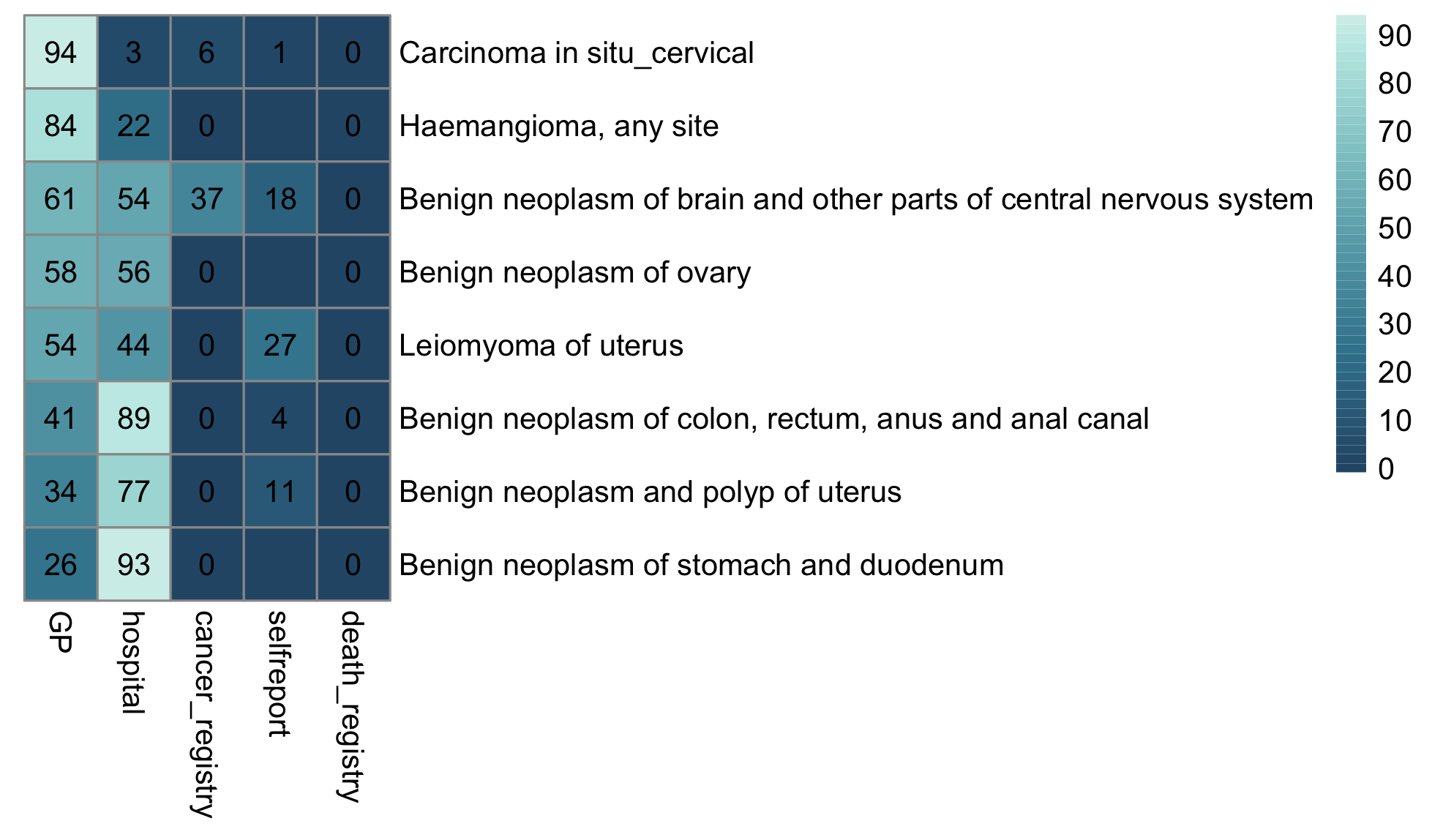

Supplement: Supplementary file 1 — Supplementary Information. [file 41598_2025_5838_MOESM1_ESM.zip › Supplementary/FigureS6_heatmap_source_Benign neoplasm or Carcinoma in situ.png]

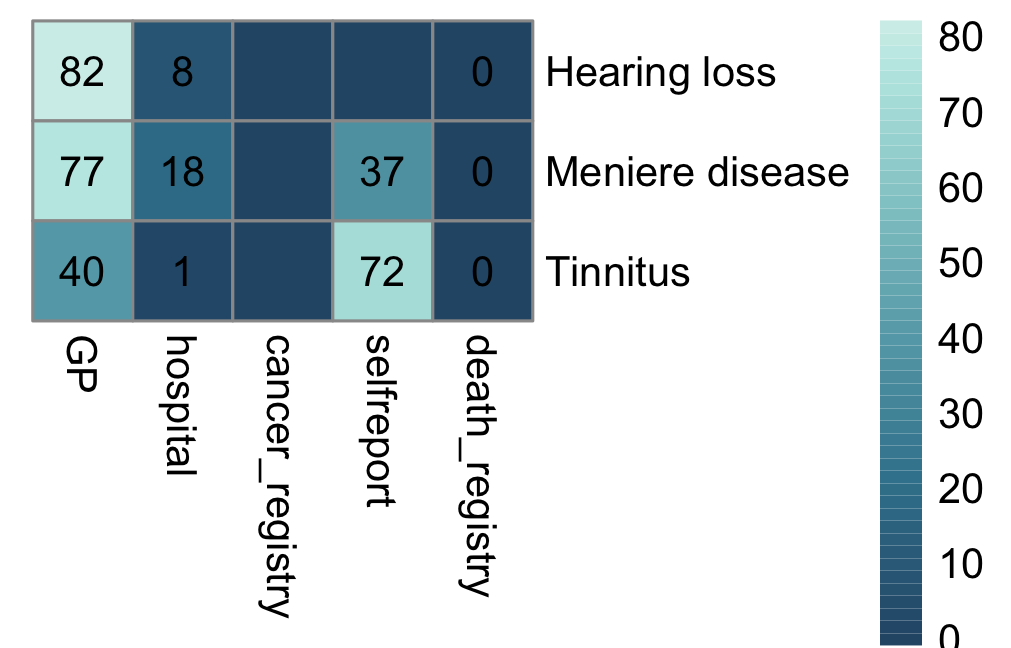

Supplement: Supplementary file 1 — Supplementary Information. [file 41598_2025_5838_MOESM1_ESM.zip › Supplementary/FigureS7_heatmap_source_Ear.png]

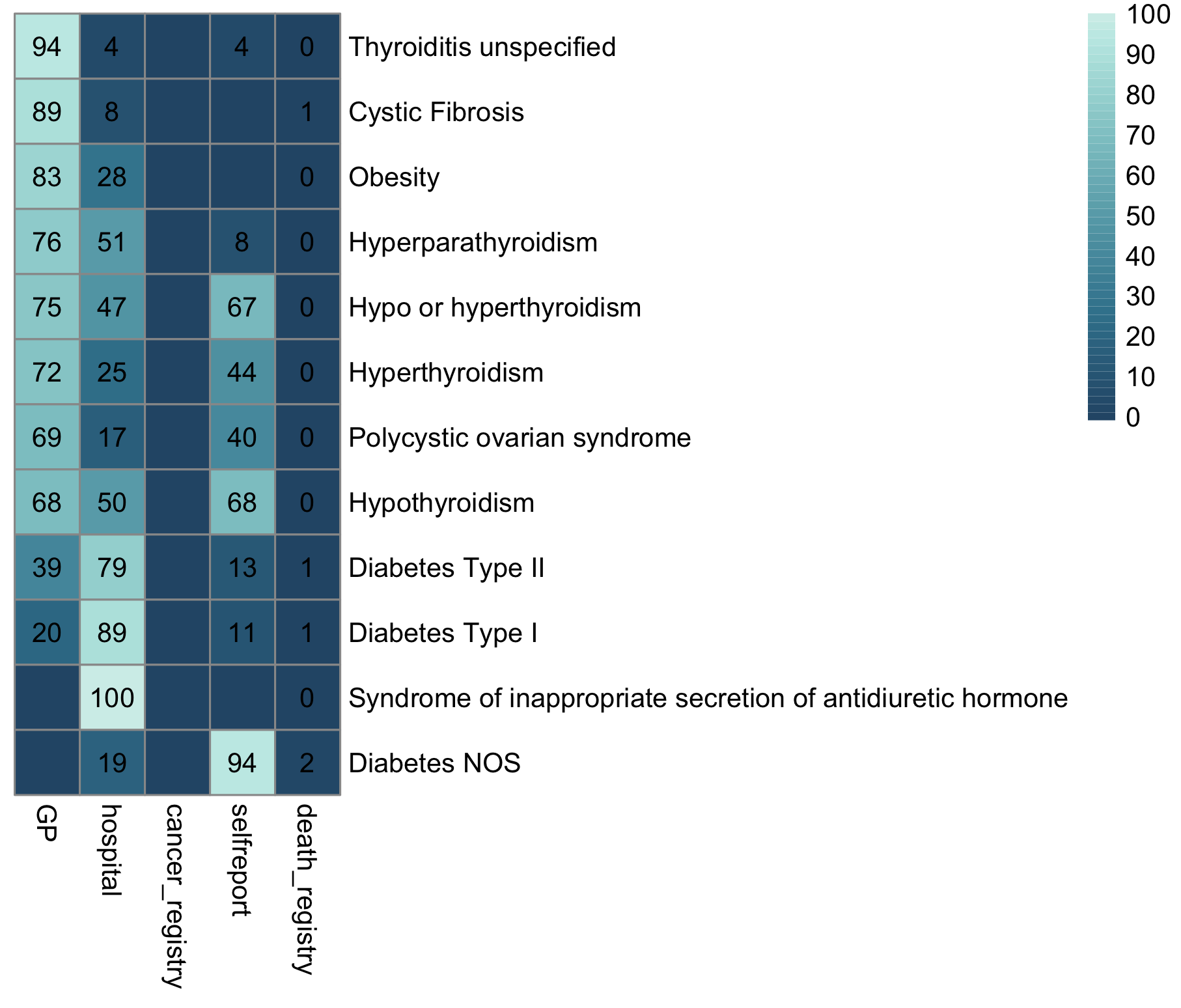

Supplement: Supplementary file 1 — Supplementary Information. [file 41598_2025_5838_MOESM1_ESM.zip › Supplementary/FigureS8_heatmap_source_Endocrine.png]

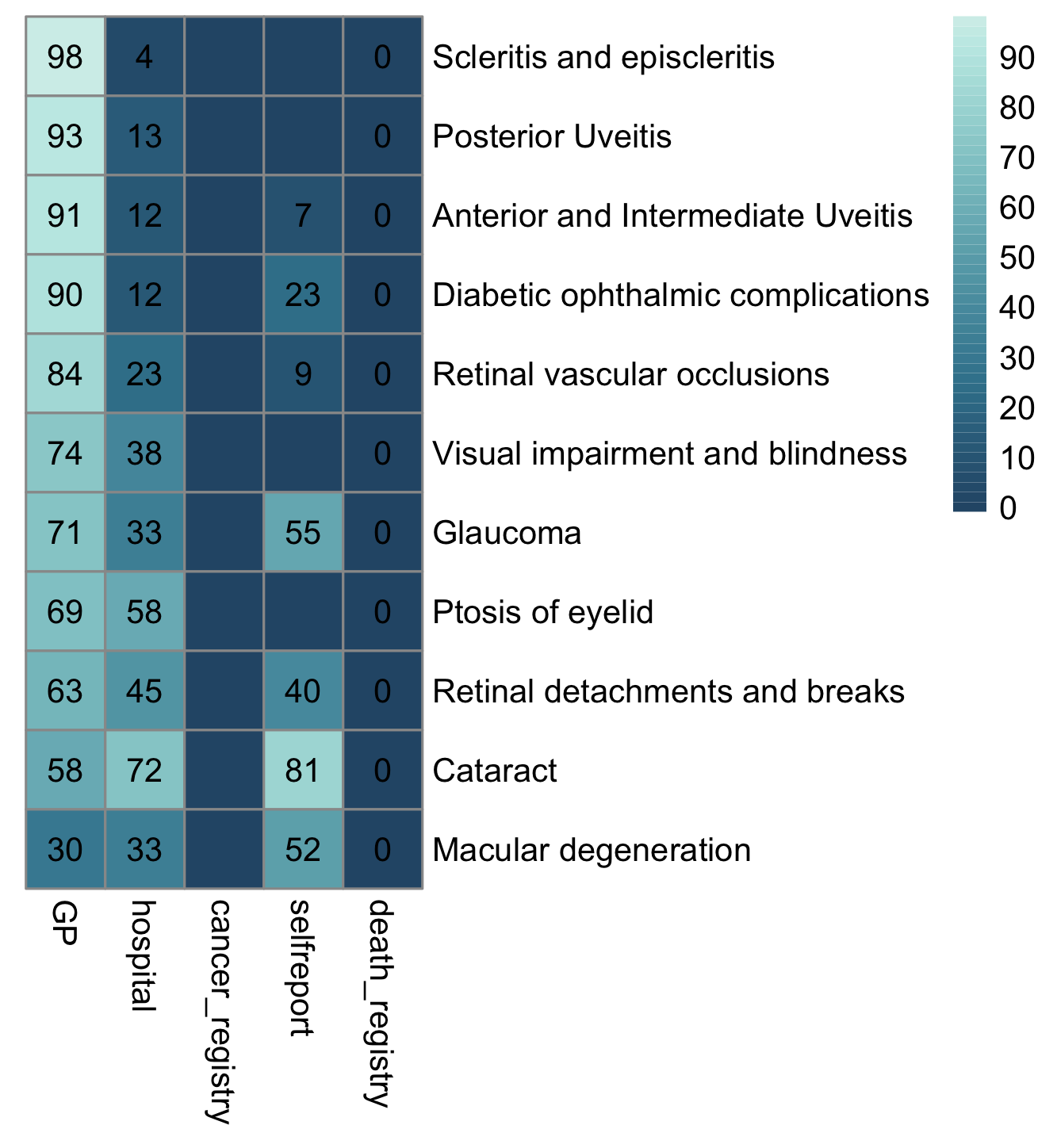

Supplement: Supplementary file 1 — Supplementary Information. [file 41598_2025_5838_MOESM1_ESM.zip › Supplementary/FigureS9_heatmap_source_Eye.png]
